# From Fragment Hit to Clinical Candidate: Discovery of Dual H_1_R/H_4_R Ligands with Superior Efficacy in Allergic Conjunctivitis

**DOI:** 10.1021/acs.jmedchem.6c01913

**Published:** 2026-08-29

**Authors:** Peter Weber, Rogier Smits, Herman D. Lim, Mounir Andaloussi, Jac Wijkmans, Bas de Boer, Thuy Duong Tran, Barbara Zarzycka, Mabel E. Dekker, Tiffany van der Meer, Rick Riemens, Niels Hauwert, Gabor Wagner, Henry F. Vischer, Matthew J. Chapin, Paul Gomes, Andy Whitlock, Maikel Wijtmans, Rob Leurs, Iwan J. P. de Esch

**Affiliations:** † Division of Medicinal Chemistry, Amsterdam Institute of Molecular and Life Sciences (AIMMS), Department of Chemistry & Pharmaceutical Sciences, 1190 Vrije Universiteit Amsterdam, De Boelelaan 1083, Amsterdam 1081 HV, The Netherlands; ‡ Griffin Discoveries BV, De Boelelaan 1083, KA-323, Amsterdam 1081 HV, The Netherlands; § Ora, LLC, 138 Haverhill Street, Suite 102, Andover, Massachusetts 01810, United States

## Abstract

Dual inhibition of the histamine H_1_ and H_4_ receptors (H_1_R and H_4_R) has been shown to provide superior anti-inflammatory efficacy in preclinical models of allergic disease compared to selective inhibition of either receptor alone. Building on this concept, we initiated a fragment-based discovery program and previously identified a quinazoline-containing fragment hit. Here, we describe the lead optimization toward compounds with unique dual H_1_R/H_4_R activity. Structure–activity relationship studies yielded potent and balanced ligands with nanomolar affinities for both receptors, as well as pharmacokinetic properties suitable for ocular administration. Among these, quinazoline **35.HCl** (GD136) and tetrahydroquinazoline **72.HCl** (GD134) demonstrate robust *in vivo* efficacy in a ragweed-induced mouse model of allergic conjunctivitis, significantly reducing hyperemia and outperforming selective H_1_R or H_4_R antagonists. Based on its pharmacological profile, ADME properties, ease of formulation, and *in vivo* efficacy, GD134 was selected as the clinical development candidate for a first-in-class dual-targeted therapy of allergic conjunctivitis.

## Graphical abstract

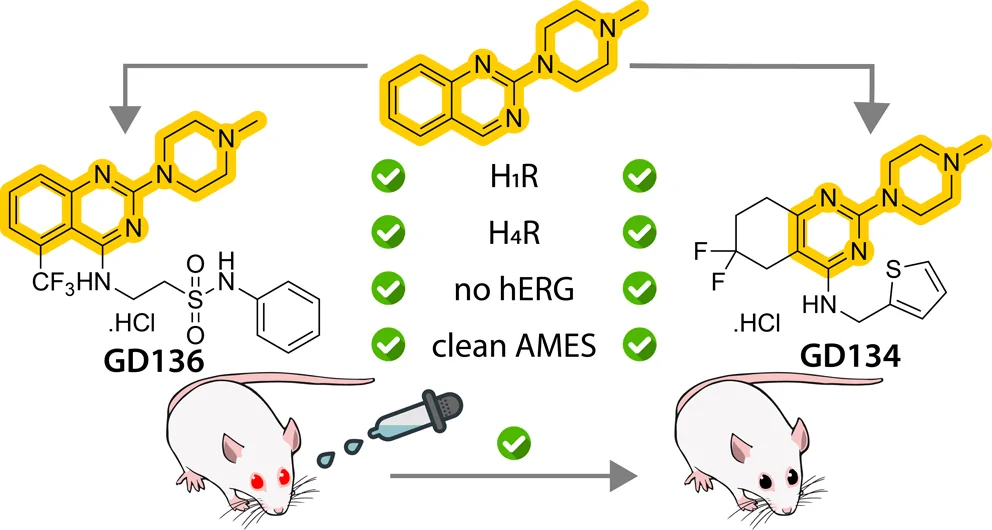

## Introduction

The endogenous amine histamine has been the subject of extensive research since its discovery in 1907.^1^ Its endogenous target proteins are four histamine receptor subtypes of the G protein-coupled receptor (GPCR) type, namely H_1_, H_2_, H_3_ and H_4_ receptors. The histamine H_1_ receptor (H_1_R) is a well-established drug target and a plethora of H_1_R antihistamines have been marketed to alleviate, with some success, various pruritic and allergic conditions, including for example allergic rhinitis (AR).^2^ H_2_R and H_3_R ligands are being used clinically to treat duodenal ulcers and narcolepsy, respectively.^3,4^ H_4_R is a key mediator of immune cell chemotaxis to the site of inflammation and is hence intensively studied in preclinical and clinical research for its role in inflammation,^5,6^ but so far none of the selective H_4_R ligands developed has reached the market.^7–15^ Two H_4_R-selective antagonists, ZPL-3893787 (adriforant) and JNJ39758979, have been in clinical studies for the treatment of atopic dermatitis (AD).^14,16^ Despite neither surpassing the Phase 2a clinical stage, these studies confirm the relevance of H_4_R as a target for the treatment of chronic inflammatory conditions such as AD. Hypothesizing com-plementary roles for H_1_R and H_4_R in pathophysiology, preclinical studies of multiple allergic conditions have confirmed that simultaneous blockade of H_1_R and H_4_R produces more robust effects than selective inhibition of either receptor alone.^10^ This dual action has traditionally been achieved by simultaneously administering selective H_1_R and H_4_R antagonists, yielding promising outcomes in preclinical models of allergic diseases. For example, in a well-established animal model of itch, the combined administration of the H_1_R antagonist diphenhydramine and the H_4_R antagonist JNJ7777120 results in a significantly greater reduction in scratching bouts compared to the separate administration of either compound.^17^ Matsushita et al. have suggested that combined H_1_R and H_4_R antagonist therapy may be superior to monotherapy with either antagonist for the management of contact dermatitis and AD.^18^ Their work found that the administration of the H_1_R antagonist olopatadine and JNJ7777120 results in a greater reduction in serum IgE and Th2 cytokines than when olopatadine was administered alone. Köchling and coworkers also reported superior results of combined inhibition of H_1_R and H_4_R in a mouse model of AD.^19^ A study by Wang and coworkers showed that inhibition of both H_1_R and H_4_R has additive effects on peanut-induced intestinal allergic responses, particularly by reducing dendritic cell accumulation in the intestine. These authors showed that combined treatment of the H_1_R antagonist loratadine and JNJ7777120 in sensitized mice significantly prevents the development of diarrhea and intestinal inflammation.^20^ Additionally, Ohsawa and coworkers claim a synergistic antipruritic and inflammatory effect upon coadministration of a selective H_1_R antagonist and a selective H_4_R antagonist in a murine AD model.^21^ These and other observations underscore the potential of dual H_1_R/H_4_R antagonism in mitigating allergic responses and inflammation. However, all presented preclinical evidence exclusively used combination treatment of established H_1_R-selective and H_4_R-selective antagonists to elicit the observed effects. We believe that a polypharmacological approach, in which H_1_R and H_4_R antagonism is unified in one ligand, is superior to a combined approach, due to a lack of potential drug–drug interactions and, in principle, less complex pharmacokinetics.

The H_1_R and H_4_R antagonists developed to date exhibit high affinity for only one of the two histamine receptor subtypes, suggesting significant differences in the ligand-binding sites of H_1_R and H_4_R. Indeed, sequence alignments of the human H_1_R and human H_4_R reveal a low sequence identity.^22,23^ Chemogenomic analyses further affirm the divergence of these receptor subtypes, as evidenced by the minimal overlap in hit sets from our fragment library screenings, indicating that these receptors bind highly distinct chemotypes.^24^ Indeed, the number of disclosed compounds with affinities for both the H_1_R and H_4_R is extremely small. Only a few imidazole-containing histamine derivatives have moderate affinities for both the H_1_R and H_4_R.^25,26^ In addition, two patent disclosures describe nonimidazole compounds with affinities for both receptors.^27,28^ However, most nonimidazole H_1_R antagonists exhibit negligible affinity for the H_4_R, except for promiscuous benzodiazepine derivatives, such as clozapine, which bind to many GPCR targets.^29–33^ Although a few targeted efforts to design dual-activity antagonists have been reported, such studies remain rare. Those that do exist often yield compounds with imbalanced activity, typically displaying high affinity for H_1_R but markedly lower affinity for H_4_R,^34,35^ or result in compounds with a more balanced profile but with affinities in the single-digit micromolar range.^36^ Thus, the development of potent dual-activity ligands for H_1_R/H_4_R remains a challenge. We previously described a fragment library screen that identified a promising H_4_R quinazoline-containing fragment hit, VUF10249 (Figure 1).^37^ This fragment hit was optimized using a pharmacophore model based on the selective H_4_R ligand JNJ7777120 and a clozapine derivative with dual H_1_R and H_4_R activity. Based on the hypothesis of a hydrophobic pocket that could be targeted by growing the quinazoline hit fragment VUF10249 from the 4-position (Figure 1), VUF10497 was developed as a first potent dual-active H_1_R/H_4_R antagonist (Figure 1).^38^ To improve the solubility of VUF10497, more polar substituents were introduced to increase the topological polar surface area (TPSA). This led to VUF10519, a highly potent H_4_R antagonist with considerable affinity for the H_1_R, featuring a sulfonamide moiety linked via an ethylene linker (Figure 1).^39^

**Figure 1:**
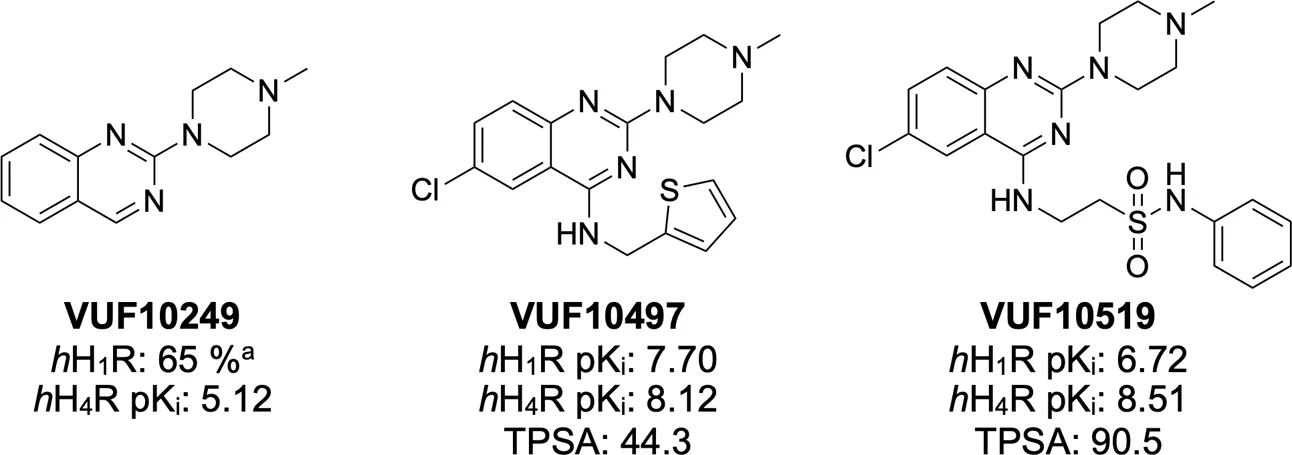
Structure, binding affinity, and TPSA (Å²) of VUF10249, VUF10497, and VUF10519.^a^Percentage displacement of radioligand by 10 μM of VUF10249.

Having found this unique activity profile, we focused on clinical translation. Allergic conjunctivitis (AC) is an ophthalmic disease affecting around 30% of the global population. It is mediated by a complex network of immune cells that is, among others, mediated by the histamine signaling cascade.^40^ Reports in literature indicate an additive effect of H_1_R and H_4_R antagonists in an AC mouse model,^41^ and because both the H_1_R and H_4_R are expressed in the conjunctiva,^42^ AC can be targeted via topical ocular delivery. Additionally, we have access to validated preclinical animal models that translate to clinical data. However, initial leads VUF10497 and VUF10519 did not meet our requirements, having suboptimal solubility for VUF10497 (*vide supra*) and an unbalanced affinity profile for VUF10519 (Figure 1). Therefore, lead optimization of these structures was necessary, particularly with respect to dual H_1_R and H_4_R activity. To minimize the risk of preclinical failure, a quinazoline and tetrahydroquinazoline series were developed in parallel. Synthesis and pharmacological evaluation of a range of new compounds from both series enabled critical structure–activity relationship (SAR) analyses for H_1_R and H_4_R. The affinity of compounds with dual activity for H_1_R and H_4_R, alongside the comprehensive *in vitro* and *in vivo* pharmacological profiling of leads from both compound series, will be presented here. In addition to the challenge of maintaining affinity for both receptor subtypes, the development of clinical candidates was complicated by severe species differences (details of which will be reported elsewhere). For *in vivo* studies, we prioritized compounds with a balanced affinity profile for the H_1_R and H_4_R, and that demonstrate favorable pharmacodynamic and pharmacokinetic properties. Ultimately, our efforts afforded GD134 as a clinical candidate.

## Results and Discussion

### Chemistry

#### Quinazolines

Quinazoline-based ligands having either an amide (**8**), sulfone (**9**) or sulfonamide (**10–35**) were synthesized by convergent syntheses. This strategy required a 2,4-dichloroquinazoline and precursors bearing the amide, sulfone or sulfonamide functional group. Sulfonamide precursors **5a**–**p** were synthesized based on literature procedures (Scheme 1) by a four-step synthesis, starting with the protection of the potassium salts of taurine (**1a**) or homotaurine (**1b**) by a *N*-phtalimide moiety.^39^ The resulting sulfonates **2a**–**b** were converted to the corresponding sulfonyl chlorides **3a**–**b** using PCl_5_. Sulfonamides **4a**–**p** were synthesized by reacting **3a**–**b** with the desired amine HNR_6_R_7_, and subsequent phthalimide deprotection using NH_2_NH_2_·H_2_O yielded primary amines **5a**–**p**. In parallel, the respective 2,4-dichloroquinazoline precursors were synthesized in a two-step sequence, following literature procedures (Scheme 1).^39^ This sequence started from the corresponding anthranilic acid, which was reacted with urea to yield quinazoline-2,4­(1H,3H)-diones **6a**–**g**. These were chlorinated using POCl_3_ to yield 2,4-dichloroquinazolines **7a–g.** Final ligands **8–35** were prepared from the sulfonamide and 2,4-dichloroquinazoline precursors using two subsequent S_N_Ar reactions (Scheme 1).^39^ Specifically, the 2,4-dichloroquinazolines were substituted at the 4-position with primary amines **5a**–**p** at RT. Upon completion of the reaction (as assessed by TLC/LC analysis), *N*-methylpiperazine was added in a one-pot fashion, after which microwave-assisted heating yielded target compounds **8–35** in varying yields (4–99%). Regiochemistry was established based on a key through-space fluorine-proton correlation in a ^19^F–^1^H HOESY 2D NMR analysis of compound **35** and extrapolated to other compounds based on consistent spectral features in non-HOESY NMR analyses, including HSQC and HMBC. Ligand **35** was transformed into its solid HCl salt (**35.HCl**, GD136) for further *in vitro* and *in vivo* studies. The in-depth 2D NMR analysis of **35** and **35.HCl** is provided in the SI (Supporting Information S13–S26).

**Scheme 1:**
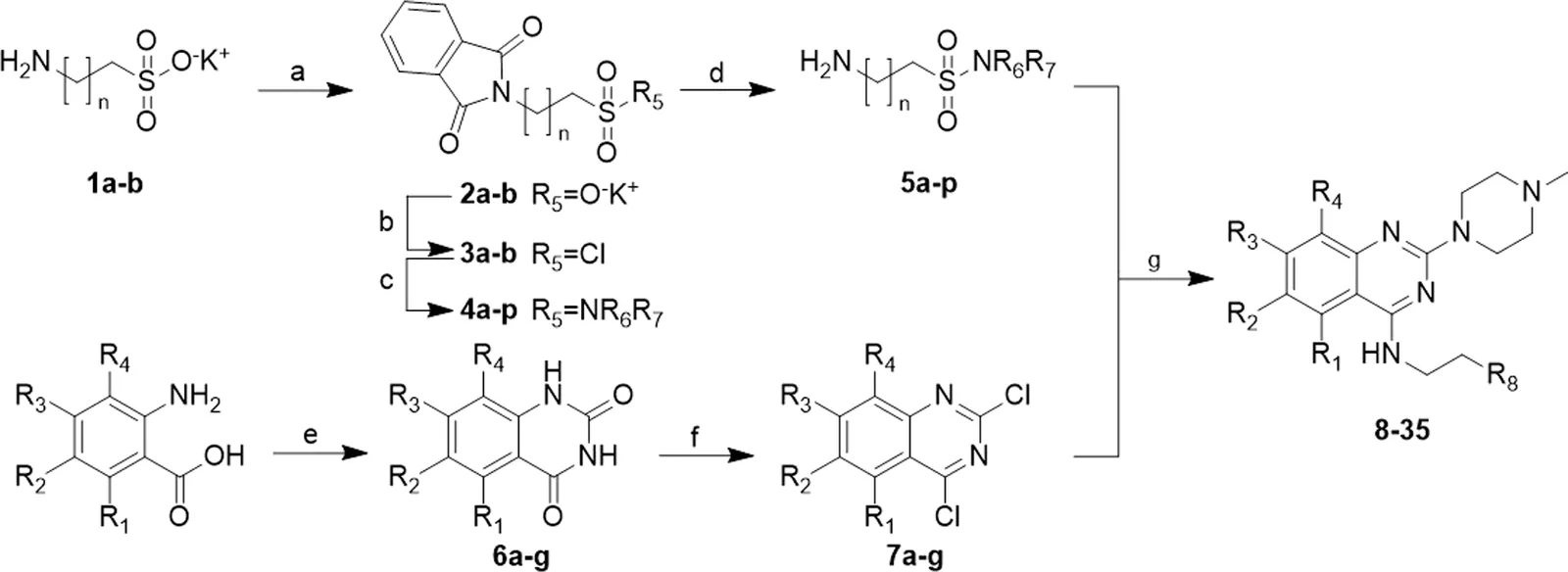
Reagents and Conditions: a) KOAc, phtalic anhydride, AcOH, 95 °C, 4 h, 81%-quant.; b) PCl_5_, PhMe, reflux, 2.5 h, 79–94%; c) HNR_6_R_7_, CHCl_3_ or DCM, RT, 16 h, 35%-quant.; d) NH_2_NH_2_·H_2_O, EtOH, reflux, 3 h, 33%-quant.; e) Urea, 140–150 °C, 6–16 h, 58–98%; f) POCl_3_, DIPEA or Et_2_NPh, reflux, 3–16 h, 13–91%; g) 1) H_2_NCH_2_CH_2_R_8_, DIPEA, EtOAc or THF, RT, 3–6 h, 2) *N*-Methylpiperazine, EtOAc or THF, 120 °C, μW, 10 min, 4–99%.

#### Tetrahydroquinazolines

6,6-Difluoro-5,6,7,8-tetrahydroquinazoline-based ligands having either N-based heteroaliphatic rings or different (hetero)­aromatic side chains were synthesized by convergent synthesis as well (Scheme 2), using similar key steps as outlined in Scheme 1. The strategy required dichloride **43** and different amine precursors bearing the N-based heteroaliphatic rings or (hetero)­aromatic side chains. These amine precursors included heterocyclic precursors **37a**–**b** and **39a**–**b**, which were synthesized from their corresponding aldehydes. To obtain **37a**–**b**, the aldehydes were reacted in a Henry reaction with NH_4_OAc and CH_3_NO_2_ to yield nitroalkenes **36a**–**b**, which were reduced to primary amines **37a**–**b** using LiAlH_4_.^43^ To obtain **39a**–**b**, the corresponding aldehydes were reacted with H_2_NOH·HCl to yield oximes **38a**–**b**, which were reduced using Zn in AcOH to yield primary amines **39a**–**b** in good yields (60–78%). Dichloride **43** was synthesized in a four-step synthesis starting from 4,4-difluorocyclohexan-1-one.^44^ Reaction of this ketone with Me_2_CO_3_ and NaH yielded enol-ester **40**, which upon reacting with NH_4_OAc afforded enamine **41** in quantitative yield over two steps. Ring closure was achieved by reacting **41** with COCl_2_ under basic conditions to yield tetrahydroquinazoline **42**, which was chlorinated using POCl_3_ to provide dichloride **43** in good yield (73%). Target compounds **60**–**75** were prepared from **43** and the respective amine precursors in a two-step sequence. As in Scheme 1, both steps were S_N_Ar reactions, although for this series the monochloro intermediates **44–59** were isolated and purified. First, the 4-position of **43** underwent substitution with various amines under microwave irradiation to accelerate this step, yielding **44–59** with varying yields (4–97%). For the synthesis of intermediates **57–59** (final products **73–75**), primary amines **37a**–**b** and commercially available 2-(thiophen-2-yl)­ethan-1-amine were cyclized with formaldehyde to bicyclic amine intermediates that were used without further purification in the first S_N_Ar reaction. For all compounds, the second S_N_Ar reaction used *N*-methylpiperazine to yield target compounds **60**–**75** in a range of yields (13–88%). The regiochemistry was determined using a hallmark through-space proton–proton correlation observed in the ^1^H–^1^H NOESY 2D NMR spectrum of **72**. This trend was extended to other compounds by comparing consistent spectral features across 2D NMR methods, such as HSQC and HMBC. Compound **72** was converted into its hydrochloride salt form (**72.HCl**, GD134) and used in this form for subsequent *in vitro* and *in vivo* experiments. Detailed 2D NMR data for both **72** and **72.HCl** is available in the SI (Supporting Information S27–S39).

**Scheme 2:**
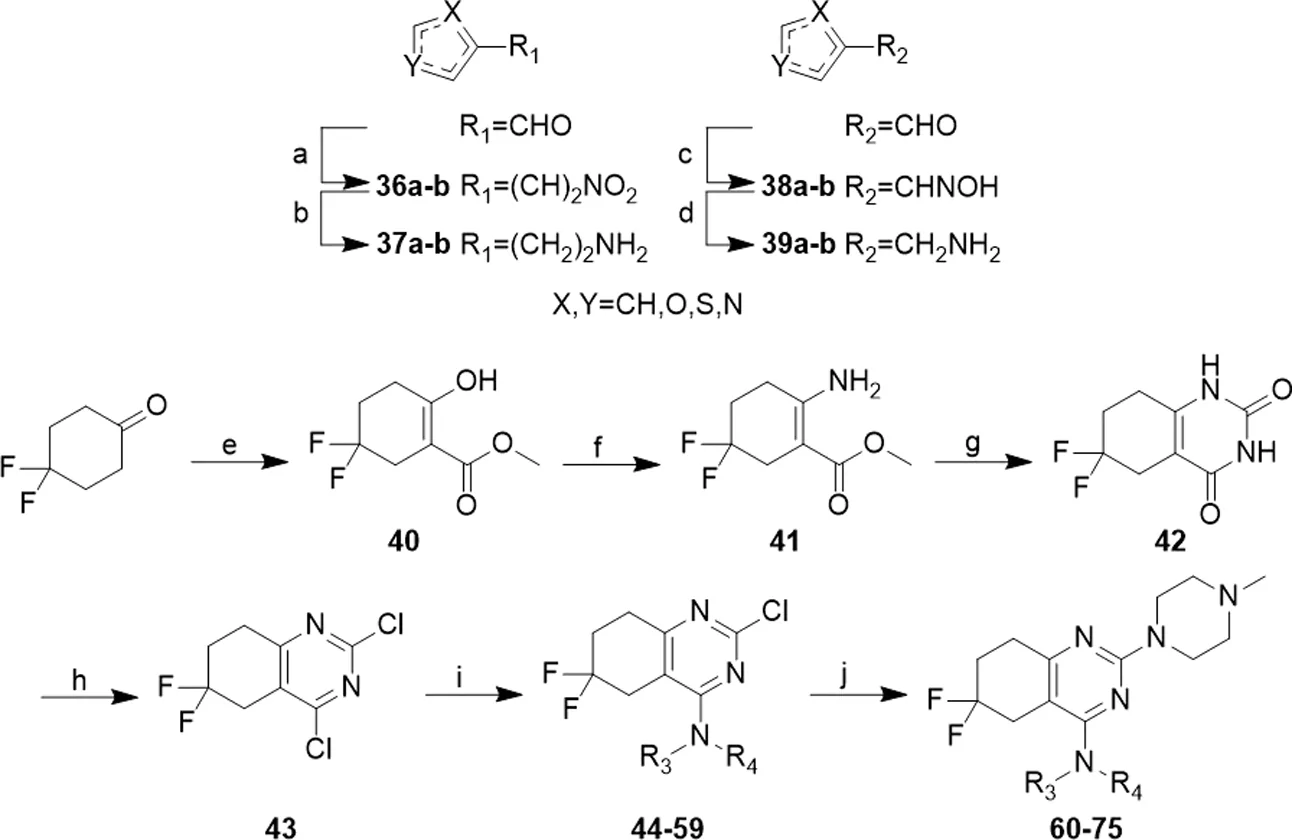
Reagents and Conditions: a) NH_4_OAc, MeNO_2_, 90 °C, 1 h, 66–82%; b) LiAlH_4_, Et_2_O/THF, RT, 16 h, 22–38%; c) NH_2_OH·HCl, NaOAc, EtOH, RT, 72 h, 76–90%; d) Zn, AcOH, 90 °C, 6 h, 60–78%; e) Me_2_CO_3_, NaH, THF, reflux, 2 h, quant.; f) NH_4_OAc, MeOH, RT, 14 h, quant.; g) 1) Pyridine, COCl_2_, DCE, 0 °C, 2 h, 2) Aq. NH_4_OH, 50 °C, 16 h, 83%; h) POCl_3_, DIPEA, reflux, 1 h, 73%; (i) HNR_3_R_4_, DIPEA, MeCN or dioxane, 70 °C, μW, 30–60 min, 4–97%; j) *N*-Methylpiperazine, DIPEA, dioxane, 150 °C, μW, 1–2 h, 13–88%.

#### Structure–Activity Relationship

Previous studies for indole, benzimidazole, quinoxaline, and quinazoline scaffolds showed that the *N*-methylpiperazine moiety is an essential structural element for H_4_R activity,^37,45–47^ and it was hence maintained constant in the present series. Here, diverse substituents were introduced at the 4-quinazoline position to explore SAR (Table 1) and replace the thiophene moiety of VUF10497.^43^ Ethyl-amide derivative (**8**) has a moderate affinity for the H_4_R and displays a more than 10-fold selectivity over the H_1_R subtype. Remarkably, introducing a sulfone group (**9**) significantly enhances H_4_R affinity while minimally affecting H_1_R affinity. Subsequent exploration led to sulfonamide **10**, which exhibits high H_4_R affinity without a notable effect on H_1_R affinity. Monomethylation of the sulfonamide nitrogen atom in sulfonamide (**11**) is tolerated for H_4_R affinity, indicating a promising vector for further optimization. Extending the linker between the quinazoline and the sulfonamide by introduction of an extra methylene unit (**12**) does not impact the low H_1_R affinity but results in an 8-fold decrease in H_4_R affinity compared to **11**. Consequently, the ethylene linker was retained in subsequent compounds. Additional substituents on the sulfonamide nitrogen atom were also explored. Dimethylated compound **13** affords a 5-fold improvement in H_1_R affinity compared to **11** but also leads to a significant 7-fold decrease in H_4_R affinity. Increasing the size of one of the substituents, as in **14**, which has methyl and cyclohexyl groups attached to the sulfonamide nitrogen atom, leads to a more significant improvement for H_4_R than for H_1_R affinity when compared to **13**. The diethyl derivative **15** exhibits a similar affinity profile as **14**. Introduction of a phenyl ring (**16**) is tolerated for H_4_R affinity and, encouragingly, boosts the H_1_R affinity by 10-fold when compared to nonaromatic sulfonamide **14**. This made **16** (p*K*_i_ H_1_R = 7.21; p*K*_i_ H_4_R = 8.15) the first compound in the series to show considerable affinity for both H_1_R and H_4_R. To evaluate the effects of different phenyl substituents, compounds **17**–**20** were examined. While **17** (*m*-Cl) and **18** (*m*-Me) gain or maintain H_1_R affinity, respectively, a substantial drop in H_4_R affinity accompanies this. Compounds **19** (*p*-OMe) and **20** (*p*-Me) exhibit reduced affinities for both H_1_R and H_4_R compared to **16**. These results discouraged exploration of additional substitutions on the phenyl group, since no clear benefit for dual H_1_R/H_4_R affinity was observed. Instead, compounds **13**–**15** suggest that increasing bulk is beneficial for H_4_R affinity, prompting the study of **21**–**23** to assess the size dependence of the second sulfonamide substituent in **16**. Compound **21**, which contains an ethyl group, shows a 3-fold reduction in H_1_R affinity and a 9-fold decrease in H_4_R affinity compared to the methyl-analog **16**. The constrained analogs **22** and **23**, featuring an indoline and a 1,2,3,4-tetrahydroquinoline moiety, respectively, reveal improved H_1_R affinity, but the H_4_R affinity decreases 4-fold and 10-fold, respectively. The smaller benzazetidine analog was not synthesized due to the tendency of this moiety to form *aza-ortho-xylenes* via electrocyclic ring opening.^48^ Interestingly, **22** has a balanced affinity profile (p*K*_i_ H_1_R = 7.66, p*K*_i_ H_4_R = 7.72). However, since it is not known what affinity profile is ideal for the foreseen clinical applications, we prioritized high H_4_R affinity over a balanced H_1_R/H_4_R profile at this stage. Reducing the size of the second sulfonamide substituent as in previously reported compound **24** (VUF10519) results in slightly higher H_4_R affinity than **16**, suggesting that an unmethylated sulfonamide nitrogen could enhance H_4_R affinity. Replacing the phenyl ring of **24** by a benzyl group (**25**) results in an approximate 5-fold decrease in affinity for both H_1_R and H_4_R.

**Table 1:**
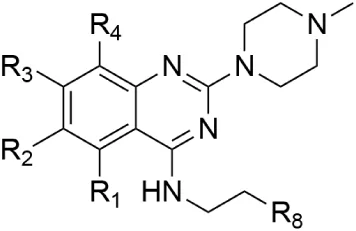

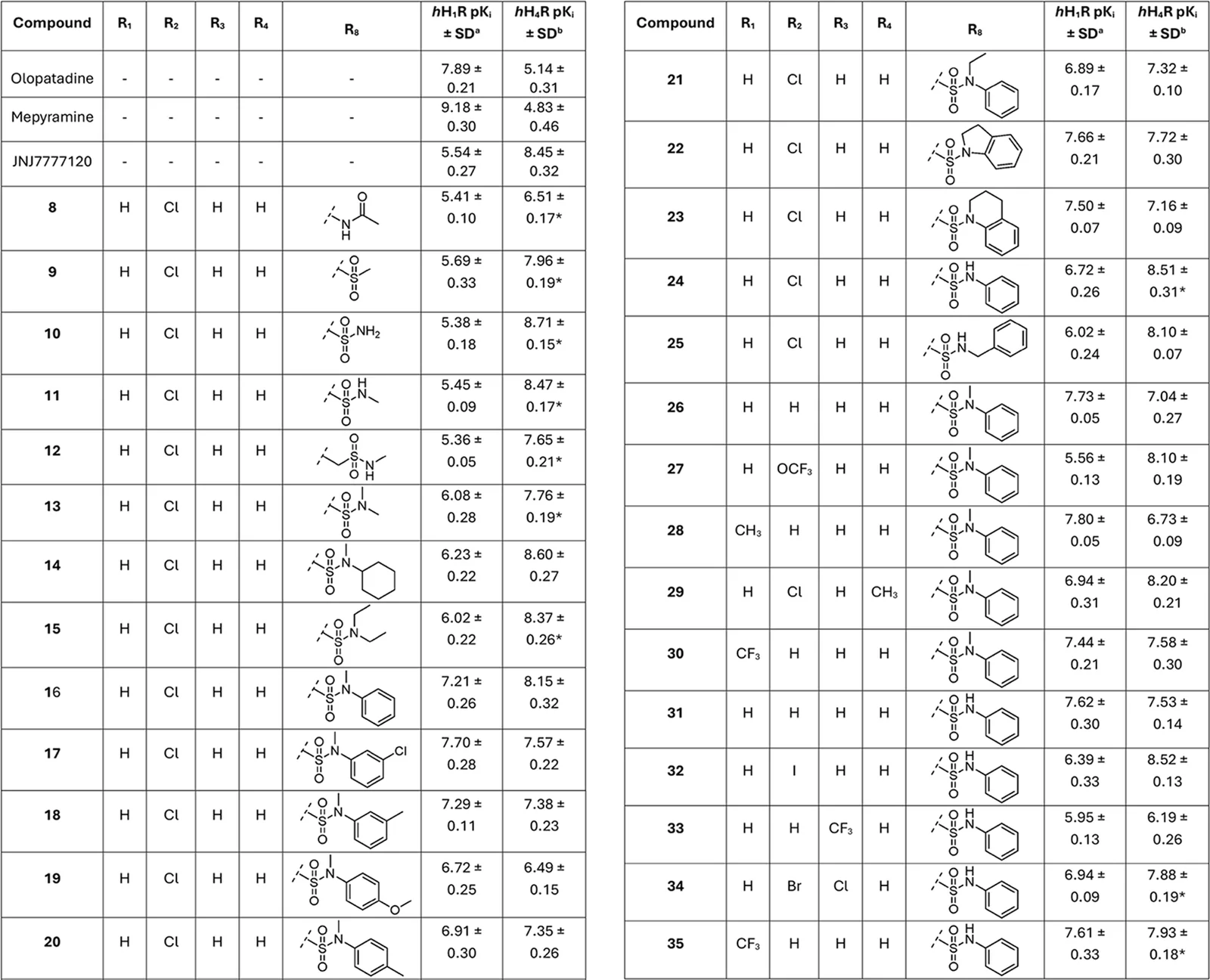
*h*H_1_R/*h*H_4_R Affinity Data for Ligands **8**–**35.** ^a^ ^,^ b

a Measured by displacement of [^3^H]­mepyramine binding using *h*H_1_R transiently expressed in HEK 293T cells.

b Measured by displacement of [^3^H]­histamine binding using *h*H_4_R transiently expressed in HEK 293T cells. pK_i_ values are calculated from at least three independent measurements as the mean ± SD. *H_4_R affinity values have been published before with minimal nonsignificant differences to the values presented herein.^39^

We next turned our attention to substituents on the quinazoline moiety. Removal of the Cl atom (**26**) from **16** shows a 5-fold increase in H_1_R affinity but a more than 10-fold decrease in H_4_R affinity. Analogs **27**–**30** with varying R_1_, R_2_, and R_4_ substituents were explored. Compound **27**, featuring an electron-withdrawing −OCF_3_ substituent at the R_2_ position, demonstrates a more than 100-fold reduction in H_1_R affinity compared to **26**, with an approximately 10-fold increase in H_4_R affinity. The 5-methyl-substituted analog **28** exhibits a bias toward H_1_R, maintaining H_1_R affinity while showing a slight reduction in H_4_R affinity compared to **26**. Compound **29**, with 6-chloro and 8-methyl substituents, shows improved H_4_R affinity but a significant reduction in H_1_R affinity compared to **26**. Derivative **30**, with a 5-CF_3_ substituent, has reduced H_1_R affinity compared to **26**, yet owing to its appreciable H_4_R affinity, exhibits the most balanced affinity pattern of *N*-methylated analogs. Given that an unmethylated sulfonamide nitrogen appears beneficial for H_4_R affinity (*vide supra*), we also assessed quinazoline substituents on **24** using compounds **31**–**35**. Compound **31**, having no substituents on the quinazoline core, displays a more balanced affinity pattern compared to **24**, with a 9-fold improvement in H_1_R affinity and, however, a 10-fold decrease in H_4_R affinity. When comparing **31** to its *N*-methylated analog **26**, the beneficial effect of a sulfonamide N–H moiety was reinforced, since **31** maintains H_1_R affinity and gains 5-fold in H_4_R affinity compared to **26**. Moreover, N–H analogs **24** and **31 e**xhibit lower calculated logD (cLogD) values (2.0 and 2.5, respectively) than their *N*-methylated counterparts **16** and **26** (2.4 and 3.2, respectively). This suggests improved solubility for these N–H analogs, which was an additional argument for pursuing N–H analogs **32**–**35**. The affinity profile of **32**, featuring a 6-iodo substituent, is less balanced, with approximately 100-fold higher potency at H_4_R than at H_1_R. Interestingly, its H_4_R affinity surpasses the affinity of reference compound JNJ7777120. Introducing a 7-CF_3_ substituent (**33**) reduces both H_1_R and H_4_R affinities compared to **31**. Derivatives with more than one substituent on the aryl ring of the quinazoline core, i.e., **29** and **34**, do not lead to promising results (e.g., compare **28** and **29**). However, introducing a 5-CF_3_ substituent (**35**) results in a balanced affinity profile with an H_1_R affinity similar to reference compound olopatadine (Table 1), while maintaining high H_4_R affinity.

Encouraged by the suggestion that increased molecular saturation may improve clinical success,^49^ we have maintained an ongoing interest in exploring three-dimensional (3D) scaffolds.^49–51^ As part of this strategy, we considered alternative scaffolds to the quinazoline scaffold. A broad Japanese patent application disclosed a variety of pyrimidine derivatives as H_4_R ligands,^44^ among many others featuring a 6,6-difluoro-5,6,7,8-tetrahydroquinazoline scaffold. It is noted that this patent does not contain any claims or pharmacological data related to H_1_R. In these examples, the 3D scaffold was equipped with sulfonamide substituents, which were also employed in earlier patent disclosures of our own group and are illustrated in Table 1.^52^ We decided to explore this scaffold, but to introduce novelty, we used smaller, more hydrophobic substituents, inspired by our previously published H_4_R series.^36^ The structures and resulting H_1_R and H_4_R affinities are summarized in Table 2.

**Table 2:**
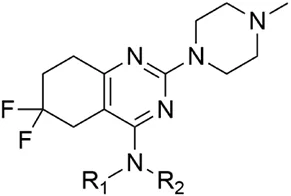

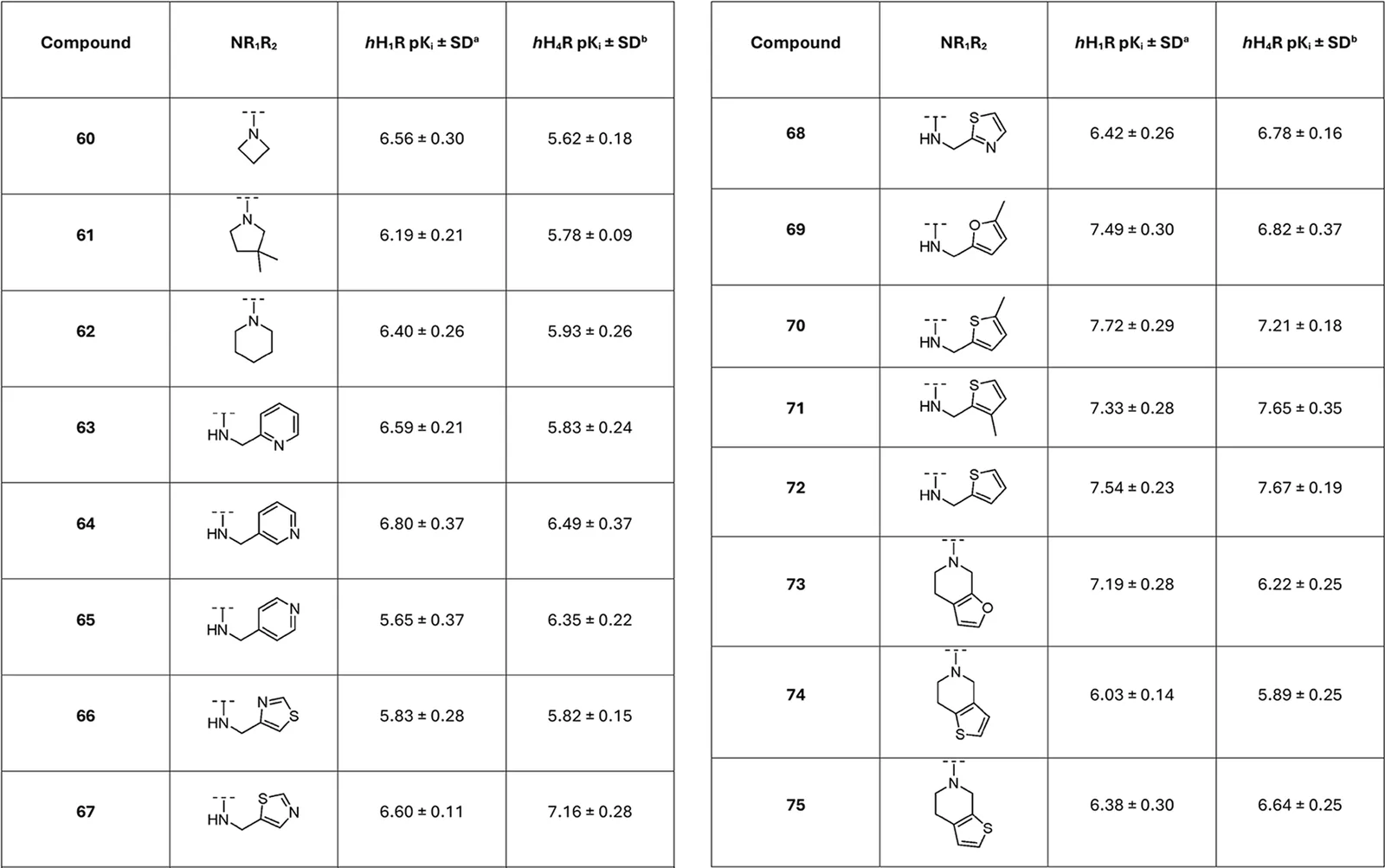
*h*H_1_R/*h*H_4_R Affinity Data for Ligands **60**–**77.** ^a^ ^,^ ^b^

a Measured by displacement of [^3^H]­mepyramine binding using *h*H_1_R transiently expressed in HEK 293T cells.

b Measured by displacement of [^3^H]­histamine binding using *h*H_4_R transiently expressed in HEK 293T cells. p*K*_i_ values are calculated from at least three independent measurements as the mean ± SD.

The SAR detailed in Table 2 focuses on a difluoro-5,6,7,8-tetrahydroquinazoline scaffold, representing a distinct chemical series of dual-activity H_1_R/H_4_R ligands compared to the series examined in Table 1. To expand the SAR scope, a diverse array of substituents was introduced at the 4-position of the scaffold to modulate affinity toward both H_1_R and H_4_R. Initial modifications involved the incorporation of nitrogen-containing aliphatic heterocycles, such as azetidine, 3,3-dimethylpyrrolidine, and piperidine, yielding analogs **60**–**62**. These compounds exhibited low binding affinity toward either receptor subtype, suggesting that this subclass of amine substituents may not be favorable for receptor engagement.

Subsequently, derivatives **63**–**65** bearing 2-, 3-, and 4-pyridinylmethanamine substituents were synthesized to investigate the effect of pyridyl nitrogen positioning. While affinities remained essentially unchanged compared to derivatives **60**–**62**, relocation of the nitrogen from the 2- or 3-position (**63** and **64**, respectively) to the 4-position (**65**) led to a notable 10-fold reduction in H_1_R binding. Returning to five-membered heterocyclic substituents that have been used successfully in earlier studies (Figure 1),^38^ thiazole isomers **66**–**68** were synthesized, exhibiting distinct affinity patterns depending on their heteroatom position. Derivative **66**, featuring a 2-(thiazol-4-yl) substituent with a nitrogen at the 2-position, demonstrated the lowest affinity for both receptors within this subset. Notably, shifting the sulfur atom to the 2-position, as in derivatives **67** and **68**, resulted in a more than 5- and 10-fold increase in H_1_R and H_4_R affinity compared to **66**, respectively. This was encouraging, as it indicated the potential to achieve high H_1_R and H_4_R affinities with a reduced linker length compared to the hit compound **35** in Table 1.

Subsequently, derivatives **69**–**72**, incorporating either a furan (**69**) or thiophene (**70**–**72**) heterocycle, were synthesized. These derivatives, with their respective heteroatom at the 2-position, were designed based on the promising results from compounds **67** and **68**. Compared to the thiazole derivatives, compounds **69**–**72** exhibit significantly improved affinity for both H_1_R and H_4_R. Specifically, thiophene derivatives **70**–**72** demonstrated high dual affinity. Methylation of the thiophene ring at the 2- or 5-position had minimal impact on the overall affinity pattern, leading to the selection of the nonmethylated derivative **72** for further exploration.

To assess whether rigidification through cyclization would enhance receptor affinity, derivatives **73**–**75** were synthesized. However, for the furan-containing derivative **73** and the thiophene-containing derivatives **74** and **75**, rigidification decreased affinity for both receptors. Consequently, derivative **72** was identified as the best compound from this series of difluoro-5,6,7,8-tetrahydroquinazoline-based derivatives. Its affinity profile (p*K*_i_ H_1_R = 7.54, p*K*_i_ H_4_R = 7.67) closely resembled that of **35** (p*K*_i_ H_1_R = 7.61, p*K*_i_ H_4_R = 7.93). Both compounds **35** and **72** exhibit favorable ligand-lipophilicity efficiency (LLE), with LLE_H1R_ values of 3.84 and 3.70, respectively, and LLE_H4R_ values of 4.43 and 3.79, respectively. Following comprehensive preclinical evaluation of compounds exhibiting comparable affinity profiles (**17**, **22**, **30**, **31**, **71**), **35** and **72** were identified as our lead candidates as they demonstrate the most favorable properties (*vide infra*) for targeting AC.

### Molecular Modeling of **35** and **72**

Achieving high affinity at both H_1_R and H_4_R is intrinsically challenging due to substantial differences in their orthosteric binding sites. To propose a plausible rationale for the dual activity of **35** and **72**, molecular docking followed by short 100 ns molecular dynamics (MD) simulations was performed using the doxepin-bound H_1_R structure (PDB: 3RZE)^53^ and the recently reported adriforant-bound H_4_R structure (PDB: 9JG1).^54^ In all systems, the compounds remained stable in the binding pocket with compounds displaying a maximum RMSD of ∼2 Å relative to the initial docking pose after alignment to the receptor backbone (Figures S1–S4). Molecular dynamics further revealed additional protein–ligand and water-mediated interactions, and minor induced-fit rearrangements within the extracellular loop (ECL) region relative to the experimental structures, particularly involving H_1_R residues Y185^ECL2^ and F184^ECL2^. Representative binding poses were obtained by clustering MD trajectories using protein–ligand interaction fingerprints.

In H_1_R, **35** is anchored by a persistent ionic interaction between the protonated *N*-methylpiperazine and D107^3.32^ (100%) (Figure 2A). The quinazoline moiety is deeply buried at the base of the orthosteric binding pocket, where it engages in π-π interactions with F432^6.52^ (97%) and W428^6.48^ (70%). Additional hydrophobic contacts within the lower aromatic region involve I115^3.40^, F432^6.52^, F199^5.47^, and W428^6.48^. In this binding mode, the −CF_3_ group is oriented toward the backbone of TM5. The phenyl ring of the quinazoline aligns well with the lower aromatic ring of doxepin in the deep hydrophobic cavity (Figure S5A), a feature observed for other H_1_R antagonists.^55^ The deep and spatially constrained positioning of the quinazoline moiety in H_1_R provides a rationale for the 100-fold loss in affinity observed upon introduction of a bulky -OCF_3_ group (**27**) relative to the unsubstituted analog (**26**). The sulfonamide NH forms a hydrogen bond with T194^5.42^ (99%), while the sulfonamide oxygens participate in water-mediated interactions with Y431^6.51^ (47%) and Y185^ECL2^ (28%). The phenyl ring of **35** occupies a subpocket beneath ECL2 formed by Y108^3.33^, F184^ECL2^, Y185^ECL2^, and F190^5.38^ and engages in transient π-π interactions with Y185^ECL2^ (35%), Y108^3.33^ (18%), and F184^ECL2^ (11%). These contacts are consistent with the beneficial contribution of an aromatic substituent to H_1_R affinity, as reflected by the higher affinity of the phenyl analog **16** relative to the nonaromatic analog **14**. Para-substitution of the phenyl ring would directly clash with the ECL2 backbone, which explains the slightly lower affinity of compounds **19** and **20** compared to the nonsubstituted phenyl analog (**16**).

**Figure 2:**
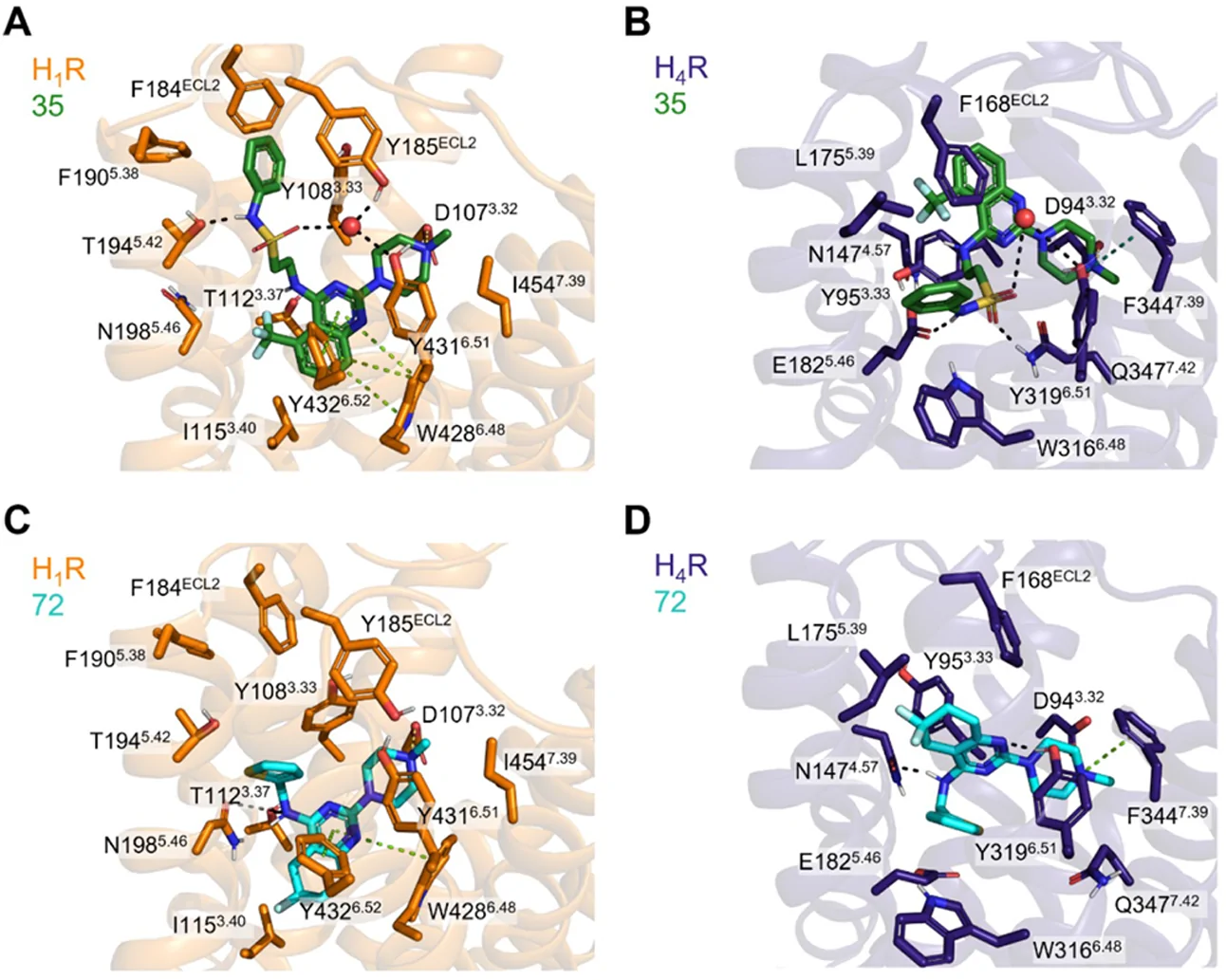
Representative binding pose of **35** (green sticks) in H_1_R (A) and H_4_R (B) and of **72** (cyan sticks) in H_1_R (C) and H_4_R (D), obtained from medoid structures from MD simulations. Hydrogen bonds are shown as black dashed lines, ionic interactions as yellow dashed lines, and π-π/cation-π interactions as light-green dashed lines. Red spheres denote positions of water molecules participating in water-mediated interactions observed during MD simulations. H_1_R PDB: 3RZE, H_4_R PDB: 9JG1.

In H_4_R, **35** adopts a flipped orientation in which the quinazoline is directed toward ECL2 and positioned between Y95^3.33^ and F168^ECL2^ (Figure 2B). Throughout the simulation, the ionic interaction between the *N*-methylpiperazine moiety and D94^3.32^ remains stable (100%). In addition, a cation-π interaction with F344^7.39^ is established, representing an H_4_R specific contact that is absent in H_1_R due to the presence of an isoleucine at that position. The sulfonamide forms hydrogen bonds with E182^5.46^ (98%) and Q347^7.42^ (82%). A similar interaction with E182^5.46^ is observed in the adriforant-H_4_R cryo-EM structure. The slightly higher H_4_R affinity of **35** compared to the methylated sulfonamide **30** can therefore be rationalized by the additional hydrogen bond with E182^5.46^. However, methylated sulfonamides retain appreciable affinity for H_4_R, indicating that this interaction is not strictly required for binding. This is consistent with the report that disruption of the E182^5.46^ interaction for H_4_R agonist clobenpropit shifts activity toward inverse agonism, suggesting that E182^5.46^ is not an essential interaction for H_4_R antagonists.^56^ Similar to H_1_R, water-mediated interactions between one of the sulfonamide oxygens and Y319^6.51^ (66%) is also observed. The phenyl ring is located in between TM5 and TM6 where it only engages in hydrophobic contacts with L175^5.39^ and W316^6.48^. The lack of π-π interactions with the phenyl ring support the observation that for H_4_R there is no additional benefit of an aromatic ring attached to the sulfonamide.

In H_1_R, **72** adopts a binding pose similar to **35** but binds ∼1.5 Å lower within the pocket (Figure 2C). The ionic interaction with D107^3.32^ is preserved during simulation. The lower binding of **72** also allows for the formation of transient hydrogen bonds with T112^3.37^ and N198^5.46^. Comparing the rotamer of N198^5.46^ in the presence of **72**, the residue adopts a more inward rotamer forming hydrogen bonds with W158^4.56^ and T112^3.37^. The pyrimidine ring forms π-π interactions with F432^6.52^ (72%) and W428^6.48^ (36%). The thiophene moiety only forms hydrophobic interactions with surrounding residues Y108^3.33^, W158^4.56^, Y185^ECL2^, and F435^6.55^. Available space around the thiophene is consistent with the minimal effect of methyl substitution (**69**–**71**) on H_1_R affinity. Comparison of the predicted binding pose of **72** with that of doxepin in H_1_R reveals a high degree of similarity, particularly in the arched conformation of the ligand around F432^6.52^ (Figure S5B).

In H_4_R, **72** adopts a flipped orientation of the difluoro-5,6,7,8-tetrahydroquinazoline similar to **35** (Figure 2D). The ionic interaction with D94^3.32^ and cation-π interaction with F344^7.39^ are maintained throughout the simulation. The difluoro substituent is located in the same place as the CF_3_-group of **35**, orientated toward L175^5.39^. Hydrogen bonds between the N–H attached to the pyrimidine ring and N147^4.57^ (81%) and one of the pyrimidine nitrogen atoms and Y319^6.51^ (63%) are also observed. The thiophene moiety sits in between TM5 and TM6, forming hydrophobic interactions with E182^5.46^, L175^5.39^, and Y319^6.51^. Similar to H_1_R, additional space around the thiophene moiety is available, consistent with the experimental observation that methyl substitution on the heterocycle (**69**–**71**) is tolerated in both receptors and has minimal impact on affinity. In both receptors, the predicted binding pose of **72** adopts a conformation in which the thiophene is oriented approximately orthogonal to the difluoro-5,6,7,8-tetrahydroquinazoline scaffold. While this geometry is readily accessible with a flexible linker, rigidification through incorporation of nitrogen-containing aliphatic heterocycles as linker replacements is likely to disfavor this conformation, in agreement with the reduced affinities observed for the rigidified compounds (**60**–**62**, **73**–**75**). In addition, rigidification removes the hydrogen-bond donor functionality that forms hydrogen bonds to T112^3.37^ and N198^5.46^ in H_1_R and N147^4.57^ in H_4_R. The combined loss of conformational adaptability and polar interactions provides rationale for the reduced affinities of the rigidified derivatives.

Overall, these molecular modeling studies suggest that the dual H_1_R/H_4_R potency of compounds **35** and **72** arises from their ability to engage the conserved D^3.32^ residue while adopting distinct, receptor-specific binding poses. In H_1_R, affinity is primarily driven by deep insertion of the core scaffolds into the lower aromatic region, forming π-π interactions. In H_4_R, compounds adopt a flipped orientation forming a cation-π interaction and additional polar contacts.

#### Physicochemical Properties

A preliminary stability assessment in water at room temperature for two months at a concentration of 1.8 mM showed no degradation of **72.HCl** but significant degradation of **35.HCl**. To investigate the stability of **35.HCl** more thoroughly, an industry-standard forced degradation study was conducted (SEPS Pharma), revealing that the ligand remains stable across various pH levels and when stored as a solid in the dark. The compound exhibits some decomposition under light and oxidative stress conditions, but this does not hinder further development. The LogD_7.4_ of **35.HCl** and **72.HCl** was determined using a shake-flask protocol to be 3.96 and 3.20, respectively. Furthermore, the thermodynamic solubility of **35.HCl** and **72.HCl** was determined using a shake-flask method and 24 h incubation at RT. The measured solubility at pH 7.4 of 161.9 μM (= 0.09186 mg/mL) for **35.HCl** and 177.9 (0.08047 mg/mL) for **72.HCl** indicates a relatively low propensity of these compounds to dissolve at this pH. Even though **35.HCl** and **72.HCl** exhibit limited solubility, based on their strong affinity and activity profiles for histamine receptor subtypes across relevant species for preclinical development (*vide infra*), both **35.HCl** and **72.HCl** were selected for detailed ADMET profiling and preclinical studies.

#### *In Vitro* Pharmacology

The binding affinities of **35.HCl** and **72.HCl** for the histamine receptor subtypes were determined in radioligand binding assays (Table 3). Both compounds exhibit nanomolar (nM) affinities at *h*H_1_R and *h*H_4_R, confirming their dual-target profiles. However, their overall selectivity profiles differ notably. Compound **35.HCl** exhibits high selectivity for the H_1_R/H_4_R subtypes, with approximately 100-fold lower affinity for *h*H_2_R and *h*H_3_R . In contrast, compound **72.HCl** maintains good selectivity over H_2_R but displays only 5-fold selectivity over *h*H_3_R. Given the intended pharmacological application, this H_3_R affinity is not expected to be detrimental. The *h*H_3_R is mainly expressed in the central nervous system (CNS). However, there is evidence that H_3_R is also expressed in the back segment of the eye and plays a role in *posterior* eye diseases such as glaucoma.^57,58^ AC is associated with the *anterior* segment, and the low selectivity over *h*H_3_R is not deemed to be a concern in the present study, as drugs administered as eye drops are effective for treating anterior eye diseases but are highly unlikely to reach the posterior segments.^59^

**Table 3:** Binding Affinities of **35.HCl**, **72.HCl**, and Olopatadine at Different Histamine Receptor Subtypes.

Subtype	p*K*_i_ ± SD (**35.HCl)**	p*K*_i_ ± SD (**72.HCl)**	p*K*_i_ ± SD (Olopatadine)
Human H_1_R^a^	7.61 ± 0.33	7.54 ± 0.23	7.89 ± 0.21
Human H_2_R^b^	5.63 ± 0.13^e^	5.92 ± 0.09^e^	n.d.^f^
Human H_3_R^c^	5.31 ± 0.02^e^	7.08 ± 0.30^e^	<5
Human H_4_R^d^	7.93 ± 0.18	7.67 ± 0.19	5.14 ± 0.31
Mouse H_1_R^a^	7.80 ± 0.15	7.62 ± 0.10	8.46 ± 0.11
Mouse H_2_R^b^	5.64 ± 0.39	6.09 ± 0.28	n.d^.^
Mouse H_3_R^c^	5.85 ± 0.16^e^	8.22 ± 0.00^e^	n.d.^f^
Mouse H_4_R^d^	7.38 ± 0.14^e^	7.68 ± 0.08	5.11 ± 0.23
Rat H_1_R^a^	7.44 ± 0.19^e^	7.72 ± 0.10^e^	8.15 ± 0.09
Rat H_2_R^b^	6.16 ± 0.31^e^	6.30 ± 0.25	n.d^f^
Rat H_3_R^c^	5.93 ± 0.00^e^	7.70 ± 0.12^e^	n.d.^f^
Rat H_4_R^d^	7.28 ± 0.14	6.81 ± 0.20^e^	n.d.^f^
Monkey H_4_R^d^	7.38 ± 0.14^e^	7.52 ± 0.06	n.d.^f^

a Measured by displacement of [^3^H]­mepyramine binding at H_1_R orthologs.

b Measured by displacement of [^3^H]­tiotidine binding at H_2_R orthologs.

c Measured by displacement of [^3^H]­Nα-methylhistamine binding at H_3_R orthologs.

d Measured by displacement of [^3^H]­histamine binding at H_4_R orthologs. All receptors were transiently expressed in HEK 293T cells. p*K*_i_ values are calculated from at least two independent measurements as the mean ± SD.

e Data obtained from the free base of **35** and **72**, respectively.

f n.d. = not determined.

Next to **35.HCl** and **72.HCl**, olopatadine, an H_1_R anti-histamine and mast cell stabilizer, was included as a reference compound, as it is the current standard of care for ocular allergy. Olopatadine exhibits high affinity and selective binding to the H_1_R, consistent with published data.^60^ Navigating species differences in histamine receptors has been notoriously difficult when developing histaminergic ligands, especially for the H_4_R subtype.^61^ Therefore, the binding affinity of **35.HCl** and **72.HCl** at the mouse and rat histamine receptor subtypes, as well as the cynomolgus monkey (*Macaca fascicularis*) H_4_R (Table 3) was determined. Both compounds maintain affinity at rodent receptors and show high affinity at monkey H_4_R, while the affinity of **72.HCl** for the H_3_R remains high in rodent species.

The antagonistic activity of **35.HCl** and **72.HCl** on H_1_R and H_4_R was evaluated in a cAMP response element (CRE)-luciferase reporter gene assay.^62,63^ The pEC_50_ values of histamine are 6.03 ± 0.08 and 5.84 ± 0.10 at human H_1_R and mouse H_1_R, respectively (Figure S6A). Histamine reduced luciferase activity induced by 1 μM forskolin with pEC_50_ values of 8.00 ± 0.03 at human H_4_R and 6.25 ± 0.04 at mouse H_4_R (Figure S6). Compounds **35.HCl** and **72.HCl** can reverse luciferase activity induced by 30 μM histamine at human and mouse H_1_Rs (Figure 3A). The effects of 0.1 μM histamine at human H_4_R or 10 μM histamine at mouse H_4_R were inhibited concentration-dependently by **35.HCl** and **72.HCl** (Figure 3B), clearly underlining their antagonism on H_4_R. Noticeably, **72.HCl** and, to a much lower extent, **35.HCl** inhibited histamine activity beyond the basal level of human H_4_R (Figure 3B), indicating its inverse agonistic activity at the human H_4_R. Potencies (p*K*_b_ values) of **35** **.HCl** and **72.HCl** from these functional assays are presented in Table 4. Noticeable differences in p*K*_b_ and p*K*_i_ values at H_1_R are observed, a phenomenon which has been observed before in GPCR pharmacology.^64^ Furthermore, differences in the intrinsic activity of probe molecules (in this case, an antagonist radioligand in a binding assay versus an agonist (histamine) in a functional assay) can result in varying determined affinity values, as has been reported for the 5-HT1A receptor.^65^ However, olopatadine does not follow the trend of **35.HCl** and **72.HCl** as its p*K*_b_ values (8.42 and 8.72 for human and mouse H_1_R, respectively) are greater than its p*K*_i_ values. Therefore, the observed discrepancies are unlikely attributable to intrinsic activities of probe molecules. Possible underlying mechanisms could include ligand binding kinetics or differential inhibition of multiple signaling pathways leading to distal downstream CRE-luc activation.

**Figure 3:**
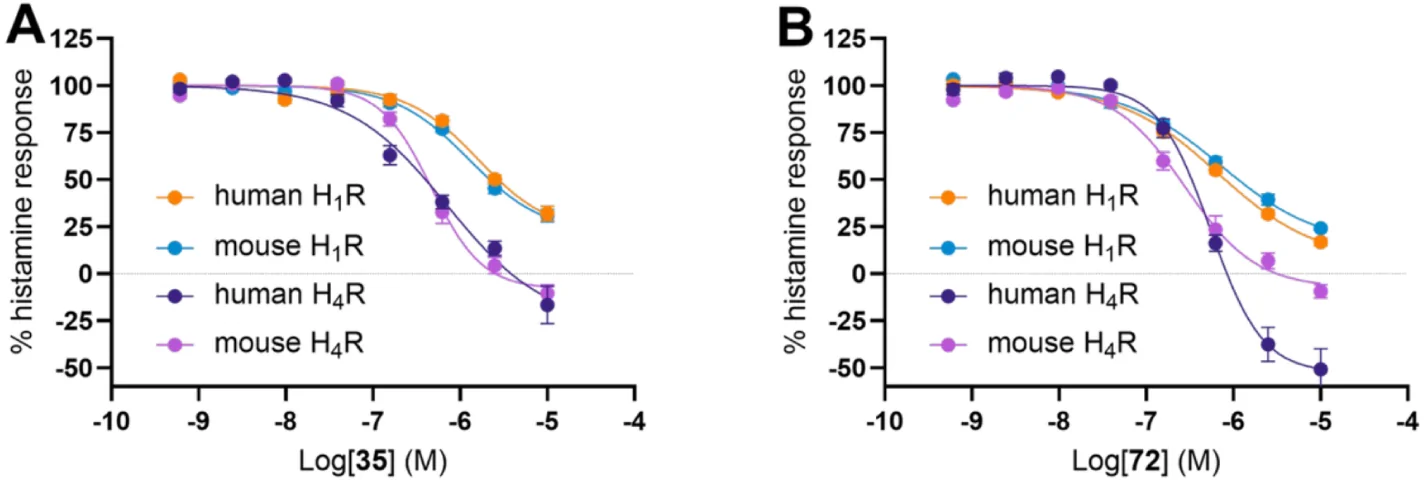
Inhibition of histamine-induced activities at the H_1_Rs and H_4_Rs by **35** **HCl** (A) and **72** **HCl** (B) as measured by CRE-luciferase assays. Initial histamine response (100%) is defined by the level of histamine activity at 30 μM (human H_1_R and mouse H_1_R), 0.1 μM (human H_4_R), or 10 μM (mouse H_4_R), and 0% is the level of basal activity in the absence of agonist. The data were normalized and combined from at least seven experiments.

**Table 4:** Inhibition Constants of **35** **HCl** and **72.HCl** for Different Histamine Receptor Subtypes in Cell-Based CRE-Luciferase Reporter Gene Assays.

Subtype	p*K*_b_ ± SEM (**35.HCl**)	p*K*_b_ ± SEM (**72.HCl)**
Human H_1_R	6.25 ± 0.11	6.64 ± 0.08
Mouse H_1_R	6.28 ± 0.13	6.53 ± 0.10
Human H_4_R	7.19 ± 0.14	7.31 ± 0.05
Mouse H_4_R	7.60 ± 0.04	7.83 ± 0.07

### ADMET Profiling of **35.HCl** and **72.HCl**

Ocularly administered compounds can undergo significant systemic absorption via the nasolacrimal drainage pathway, with estimates suggesting that up to 80% of the instilled dose may be absorbed through the highly vascularized nasal mucosa.^66,67^ Once in systemic circulation, the compound undergoes metabolism and clearance, underscoring the importance of considering pharmacokinetic properties even for topically administered ophthalmic drugs. Therefore, key *in vitro* ADMET properties for **35.HCl** and **72** **.HCl** were evaluated, including metabolic stability, clearance, permeability, plasma protein binding, hERG binding, and mutagenic potential (Table 5). Given that topical ocular administration is typically associated with some systemic exposure, rapid systemic clearance of any absorbed compound is considered beneficial. The rate of hepatic metabolism was assessed via incubation with human, rat, and mouse liver microsomes. Compound **35.HCl** exhibits low metabolic stability across all species, characterized by a short half-life (*t*_1/2_ < 10 min) and a high apparent intrinsic clearance (CL_int_ 1000 μL/min/mg). This indicates rapid hepatic metabolism, which we consider an advantage for topical treatment of ocular diseases. Interestingly, **72.HCl** exhibits increased half-life and high apparent intrinsic clearance in human and rat (*t*_1/2_ = 66 min, *t*_1/2_ = 96 min, respectively; and CL_int_ < 116 μL/min/mg), but extremely rapid hepatic metabolism in mice (*t*_1/2_ < 1 min, CL_int_ 2105 μL/min/mg). Both **35.HCl** and **72.HCl** exhibit high plasma protein binding (>98%), indicating a free unbound fraction of approximately 1%. The observed recovery rate (121% and 109% for **35.HCl** and **72.HCl**, respectively) suggests that the measured value for the matrix was higher than the nominal value of the spike. Permeability was assessed using the Caco-2 monolayer model to predict intestinal absorption.^68^ Compound **35** **.HCl** has a low to moderate permeability (P_app_ 5.6 × 10^–6^ cm/s), while **72** **.HCl** has moderate permeability (P_app_ 25.6 × 10^–6^ cm/s). Both **35** **.HCl** and **72** **.HCl** exhibit a low recovery rate (28% and 55%, respectively), which may result from poor solubility, nonspecific binding of assay plates, metabolism by the Caco-2 cells and/or accumulation of the compound in the cell monolayer. To evaluate potential off-target effects, an outsourced receptor selectivity panel was performed for **72.HCl** (Figure S7). At 10 μM, **72** **.HCl** exhibits measurable activity at α1A-, α2A-, D2S-, 5-HT1A-, 5-HT2A-receptors, as well as the GABAA­(α1β2γ2) receptor and DAT. This off-target profile is broadly consistent with those reported for other ophthalmically administered antihistamines, including epinastine^69^ and azelastine.^70^ Notably, in this selectivity panel, **72** **.HCl** also demonstrated hERG inhibition in an automatic patch-clamp assay. However, in-house hERG binding assays performed for both **35** **.HCl** and **72.HCl** indicate negligible affinity (p*K*_i_ < 5). This apparent discrepancy may reflect differences in assay format, as the selectivity panel employed a functional assay whereas the in-house assay measured binding affinity. To assess the relevance of the observed off-target activities, the pharmacokinetic profiles of **35** **.HCl** and **72** **.HCl** were further characterized following single-dose oral (PO) and intravenous (IV) administration in mice (Figure S8). Both derivatives quickly reach peak plasma concentrations and are rapidly eliminated from the systemic circulation. The low systemic exposures of **35** **.HCl** and **72.HCl** indicate that the risks of pharmacologically relevant off-target effects outside the eyes are very limited. In cytotoxicity assays, **35.HCl** shows only moderate toxicity at high concentrations and with prolonged exposure, whereas **72.HCl** shows no cytotoxicity up to 1000 μM.^70–71^ Furthermore, genotoxicity assessment using the Ames assay revealed no evidence of mutagenic potential for either **35** **.HCl** or **72.HCl,** nor for their metabolites generated in the presence of rat liver S9 fraction, at a concentration of 10 μM. Furthermore, both **35** **.HCl** and **72.HCl** were found to be safe in the herein described CAC mouse model.

**Table 5:** *In Vitro* Assessment of ADMET Properties of **35.HCl** and **72.HCl.**

ADMET property^a^	**35.HCl**	**72.HCl**
Microsomal half-life (min)	8 (human), 6 (rat), 5 (mouse)	66 (human), 96 (rat), 0 (mouse)
CL_int_ (μL/min/mg)	9.02 × 10^2^ (human), 1.10 × 10^3^ (rat), 1.38 × 10^3^ (mouse)	<1.16 × 10^2^ (human), < 1.16 × 10^2^ (rat), 2.11 × 10^3^ (mouse)
Plasma protein binding	99.4%	97.6%
Caco-2 P_app_ (cm/s)	5.60 × 10^–6^	25.6 × 10^–6^
Ames test	Clean at 10 μM	Clean at 10 μM

a The methodology of the specific assays is described in the Experimental section.

#### *In Vivo* Studies

To determine the therapeutic potential of dual-activity H_1_R/H_4_R antagonists for their use in AC, **35.HCl** and **72.HCl** were studied in a mouse conjunctival allergen challenge (CAC) model, which was conducted by Ora (Andover, Massachusetts; Study Approval Number: 12-130-0025 GFN-AG-0.1). This animal disease model has been used frequently to evaluate antiallergic drugs and is relatively predictive of clinical efficacy. The CAC model has been used to develop and approve over 19 marketed products^72^ and offers a relatively rapid and efficient path to first-in-clinic proof-of-concept Phase 2 studies.^72–74^ Compounds **35.HCl** and **72.HCl** were tested as a phosphate-buffered solution at pH 6.6. Initially, **35.HCl** dissolved as a clear solution but subsequently precipitated and was consequently formulated as a suspension (Figure S9) from day one of the *in vivo* model. Compound **72.HCl** was formulated as a clear solution (Figure S9). Control treatments included the vehicle (phosphate-buffered saline at pH 6.6), GD135 (0.1%), a quinazoline-containing selective H_4_R antagonist that lacks H_1_R affinity (Tables S1 and S40–S42), as well as clinically used AC treatments olopatadine (0.1%), a selective H_1_R antagonist/mast cell stabilizer, and the steroid prednisolone acetate (1.0%).

All animals remained in good health throughout the study. Hyperemia and squinting scores (Figure 4 and Figure S10, respectively) were assessed using a 4-point scale with 0.5-unit increments (0 = none; 4 = extremely severe). Both **35.HCl** and **72.HCl** significantly reduce hyperemia from the first challenge onward, compared with the vehicle and the selective H_4_R antagonist GD135 (Figure 4 and Figure S11, respectively), with the latter not reducing signs of AC in this model. Moreover, **35.HCl** and **72.HCl** outperform the standard ocular allergy treatments olopatadine and prednisolone acetate in the mouse CAC model after repeated challenges. A notable differentiation is that **35.HCl** and **72.HCl** can reduce eye redness, whereas existing treatments are unable to do so.

**Figure 4:**
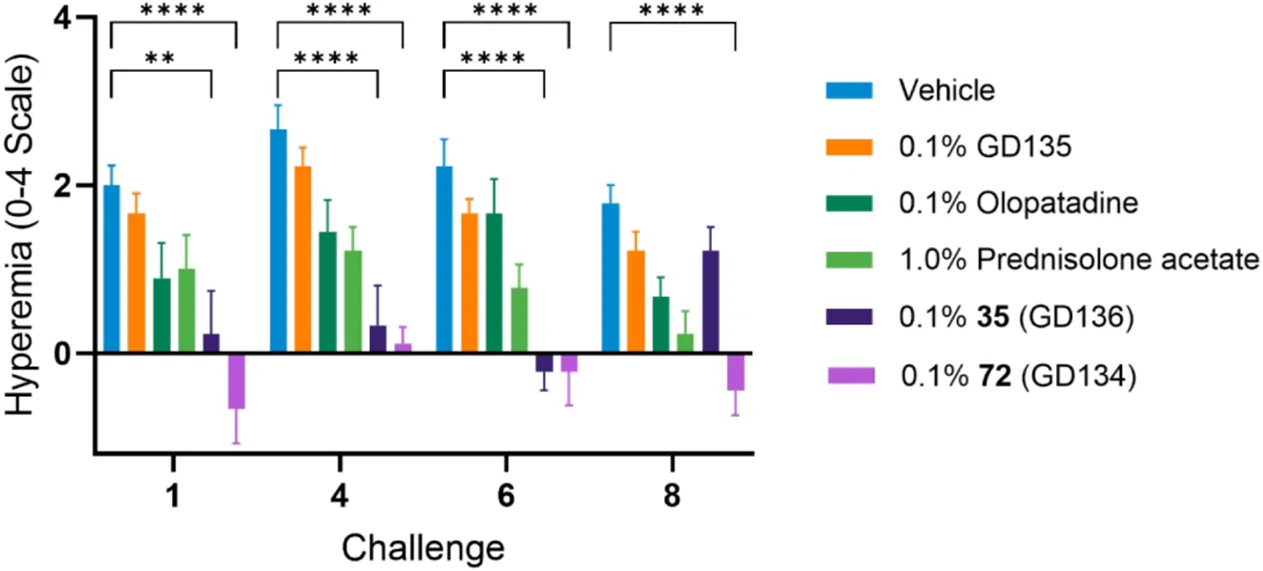
Effect of **35.HCl** and **72.HCl** on ocular hyperemia in the murine CAC model. All data are represented as the mean ± SEM for *n* = 9 animals per group. Significance: ** = *p* < 0.01, **** = *p* < 0.0001. The efficacy of **35.HCl** decreased to a level not significantly different from placebo after the 8^th^ challenge.

The differential efficacy of olopatadine and prednisolone over time likely reflects their distinct mechanisms of action. Olopatadine, an H_1_R antagonist and mast cell stabilizer, was more effective in early phase responses, characterized by histamine release. In contrast, prednisolone showed increased efficacy during later challenges, consistent with its anti-inflammatory activity in the chronic phase of the allergic response.^73,74^

Previous murine studies suggest that dual H_1_R/H_4_R antagonism may attenuate mast cell migration to inflammatory sites,^18,21^ consistent with the efficacy observed for **35.HCl** and **72.HCl** across both early and late phases of the CAC model. Notably, **35.HCl** and **72.HCl** did not increase squinting scores (Figure S10), indicating they do not cause ocular discomfort.

A decline in relative hyperemia efficacy was observed for **35.HCl** after the eighth challenge. Compound **35.HCl** was administered as a suspension (see Figure S9), which may have contributed to the observed loss of efficacy after the eighth challenge. More sophisticated formulation studies of **35.HCl** are necessary to resolve this issue. In contrast, **72.HCl** demonstrates continuous strong efficacy even with the simple formulation used in the presented study. Combining the potent dual-activity pharmacological profile, excellent pharmacokinetic and pharmacodynamic properties, ease of formulation and *in vivo* efficacy, **72.HCl** was selected as the preferred clinical candidate.

## Conclusion

Dual modulation of histamine receptors H_1_R and H_4_R represents a promising strategy for treating inflammatory disorders, such as AC due to their complementary roles in the underlying pathophysiology.^41^ Leveraging modular synthetic strategies and ligand optimization, we identified multiple series of dual-activity ligands with high affinity for both receptor subtypes. Among these, **35.HCl** (GD136) and **72.HCl** (GD134) emerge as dual H_1_R/H_4_R antagonist, displaying robust binding affinity to both human and murine H_1_R and H_4_R receptors, strong functional activity, and, for GD136, selectivity over the other two histamine receptor subtypes (H_2_R and H_3_R). GD136 and GD134 exhibit favorable pharmacokinetic properties for localized ocular administration, including limited *in vitro* metabolic stability and a short *in vivo* half-life, both of which minimize systemic exposure after application as eye drops. In a murine CAC model, GD136 and GD134 significantly reduced ocular hyperemia and demonstrated superior efficacy relative to established therapies such as olopatadine and prednisolone acetate. Importantly, GD136 and GD134 were the only treatments to substantially reduce ocular hyperemia, whereas selective H_1_R or H_4_R antagonism alone was insufficient. This unique efficacy in reducing eye redness, one of the most visible and bothersome symptoms of AC, represents a potentially important clinical differentiator for dual H_1_R/H_4_R antagonists. Moreover, neither compound increased squinting behavior, supporting their favorable ocular tolerability profile in mice. Safety studies, including mutagenicity, hERG, and off-target assessments, in combination with rapid systemic clearance that is expected to minimize the risk of systemic off-target effects following ocular administration support a favorable profile for the intended ophthalmic indication. Taken together, these findings identify **72.HCl** (GD134) as a well-balanced dual H_1_R/H_4_R antagonist with a compelling combination of efficacy, developability, and safety attributes, supporting its advancement as a clinical development candidate for AC. In accordance with standard ocular drug development practices, detailed ocular tissue distribution and pharmacokinetic studies will be conducted in subsequent stages to support later-stage clinical evaluation. The in-depth *in vitro* and *in vivo* characterization of GD134 underscores the therapeutic promise of dual-activity H_1_R/H_4_R ligands as a new first-in-class treatment modality for AC. Furthermore, this dual-target strategy may also apply to broader inflammatory indications, such as neurodegenerative diseases and asthma.^75–77^ To gain therapeutic access to these disease areas, we are developing new series of dual-activity H_1_R/H_4_R compounds with improved oral bioavailability.

## Experimentals

### Chemistry

#### General

All reagents were purchased from commercial suppliers and used without further purification. PhMe, DCM, Et_2_O, and THF were dried by passing through an activated alumina column before use. All other solvents used were used as received unless stated otherwise. TLC analyses were performed using Screening Devices or Merck F254 aluminum-backed silica plates, and visualized under 254 nm UV light or with KMnO_4_ staining. Analytical HPLC-MS analyses were conducted using a Shimadzu LC-40D liquid chromatography pump system with a Shimadzu SPD-M40 diode array detector. MS detection was performed with a Shimadzu LCMS-2020 EV mass spectrometer. The analyses were performed using the following conditions: XBridge (BEH C18) 2.5 μm column (50 mm × 2.1 mm) with solvent A (acetonitrile with 0.1% formic acid) and B (H_2_O with 0.1% formic acid) at a flow rate of 0.4 mL/min. Starting with 5% A, then a linear gradient to 90% A in 1.75 min, remaining 0.25 min at 90% A, then a linear gradient to 5% A in 0.25 min, remaining 0.75 min at 5% A, giving a total run time of 3.0 min. The purity of a compound was determined by calculating the peak area percentage of UV detection at 254 nm. HRMS spectra were determined with a Bruker micrOTOF mass spectrometer using ESI in positive ion mode. Normal phase flash chromatography was performed on a Biotage Isolera or Argonaut Flashmaster II device. Prepacked columns were purchased from Screening Devices (C18 and UltraPure irregular silica). Microwave reactions were carried out using a Biotage Initiator. IUPAC names were generated with ChemDraw Professional 22.0 (PerkinElmer). Nuclear magnetic resonance (NMR) spectra were determined with a Bruker AC200 200 MHz, a Bruker Avance II 500 MHz or a Bruker Avance III HD 600 MHz spectrometer. Chemical shifts are reported in parts per million (ppm) against the reference compound using the signal of the residual nondeuterated solvent (CDCl_3_ δ = 7.26 ppm (^1^H), δ = 77.16 ppm (^13^C); DMSO-d_6_ δ = 2.50 ppm (^1^H), δ = 39.52 ppm (^13^C); CD_3_OD δ = 3.31 ppm (^1^H), δ = 49.00 ppm (^13^C), D_2_O δ = 4.79 ppm (^1^H)). NMR spectra were processed using MestReNova 14.1 software. The peak multiplicities are defined as follows: s, singlet; d, doublet; t, triplet; q, quartet; dd, doublet of doublets; ddd, doublet of doublets of doublets; dt, doublet of triplets; dq, doublet of quartets; td, triplet of doublets; tt, triplet of triplets; qd, quartet of doublets; p, pentet; dp, doublet of pentets; br, broad signal; m, multiplet; app, apparent. For NMR listings, in addition to specific instructions that are given by the journal in the guidelines for authors the following additional procedures were used: 1) The notation “m” is used in case of obscured accurate interpretation as a result of (i) overlapping signals for different protons, or (ii) a result of overlapping signal lines within the same proton signal; 2) NMR signals that could only be detected with HSQC analysis are denoted with a # symbol; 3) NMR signals that could only be detected with HMBC analysis are denoted with a * symbol; 4) If one or more signals remain undetected after extensive 1D and 2D NMR analyses, this will be mentioned. 5) Signals for exchangeable proton atoms (such as NH and OH groups) are only listed if clearly visible (e.g., excluding the use of D_2_O or CD_3_OD) and if confirmed by a D_2_O shake and/or HSQC. The purity of all target compounds exceeds 95% as verified by HPLC.

#### General Procedure A: Synthesis of **2a–b** (Scheme 1)

The respective sulfonic acid (1.0 equiv) and KOAc (1.0 equiv) were mixed with the indicated volume of AcOH. The mixture was heated to 95 °C, and phthalic anhydride (1.0 equiv) was added. After stirring at 95 °C for 4 h, the reaction mixture was cooled to RT with continuous stirring. A white precipitate was formed which was filtered off by vacuum filtration. After washing with ice-cold EtOH, the precipitate was dried *in vacuo* to yield the corresponding product.

#### General Procedure B: Synthesis of **3a–b** (Scheme 1)

The respective potassium sulfonate **2a** or **2b** (1.0 equiv) was suspended in the indicated volume of PhMe. The mixture was heated at reflux. PCl_5_ (0.7–0.9 equiv) was added in portions, and the resulting suspension was stirred at reflux. After 1 h, a second portion of PCl_5_ (0.7–0.9 equiv) was added and heating at reflux was continued for another 90 min. The suspension was concentrated *in vacuo* and crushed ice was added to the residual solid. After full melting of the ice, the solids were filtered off, washed with ice-cold H_2_O and dried *in vacuo* to yield the corresponding product.

#### General Procedure C: Synthesis of **4c**, **4e–p** (Scheme 1)

The respective sulfonyl chloride **3a** or **3b** (1.0 equiv) was added to a solution of the corresponding amine or its salt (2.0–3.0 equiv) in the indicated volume of CHCl_3_ or DCM. The mixture was stirred at RT for 16 h. The reaction mixture was washed with H_2_O (50 mL) and 1.0 M aq. HCl (50 mL). After drying the organic layer over Na_2_SO_4_, it was concentrated, and the crude product was recrystallized from EtOH. The crystals were collected by vacuum filtration and dried *in vacuo* to yield the corresponding product.

#### General Procedure D: Synthesis of **5a–o** (Scheme 1)

The respective phthalimide **4a**–**p** (1.0 equiv) was dissolved in the indicated volume of EtOH. The mixture was heated to reflux. NH_2_NH_2_·H_2_O (64%) (1.1–1.2 equiv) was added, and heating was continued at reflux. After 3 h, the white precipitate was filtered off using vacuum filtration. The filtrate was concentrated, and the residue was resuspended in H_2_O (150 mL). The mixture was acidified to pH ∼2 using conc. HCl. The insoluble materials were filtered off using vacuum filtration and the filtrate was concentrated. The crude product was recrystallized from EtOH:H_2_O (9:1) and dried *in vacuo* to yield the corresponding product.

#### General Procedure E: Synthesis of **6a–b**, **6d–g** (Scheme 1)

The respective anthranilic acid (1.0 equiv) and urea (4.0–10 equiv) were mixed in a round-bottomed flask equipped with a reflux condenser. The mixture was heated to 140 °C. **CAUTION: urea will partially sublime and may clog the reflux condenser, leading to pressure buildup. This can be counteracted by periodically forcing the sublimed urea from the condenser back into the reaction flask with a spatula**. After stirring for 6 h at 140 °C, the reaction mixture was cooled to 100 °C and an equal volume of H_2_O was added. After stirring for 5–10 min at 100 °C, the suspension was allowed to cool to RT, and the precipitate was filtered off using vacuum filtration. The filter cake was washed with H_2_O and suspended in 0.2–0.5 M aq. NaOH. The mixture was brought to boiling for 5 min. After cooling to RT, the solution was stirred for up to 16 h. Upon acidification to pH ∼2 with conc. HCl, the white precipitate was filtered off using vacuum filtration and washed with MeOH:H_2_O (1:1). If indicated, the filter cake was further washed with H_2_O, triturated with hot EtOH (100 mL) and filtered hot. The collected solid was dried *in vacuo* to yield the corresponding product.

#### General Procedure F: Synthesis of **7a–g** (Scheme 1)

The respective quinazoline-2,4­(1H,3H)-dione **6a**–**g** (1.0 equiv) was dissolved in the indicated volume of POCl_3_ and either *N,N*-diethylaniline (1.8–2.0 equiv) or DIPEA (2.1 equiv) was added dropwise. The reaction mixture was heated at reflux for 3–16 h. The reaction mixture was carefully poured over crushed ice, and the resulting suspension was stirred until all ice had melted. **CAUTION: quenching of POCl**_**3**_ **might show unpredictable behavior and complete quenching may need extended times.** The mixture was extracted thrice with DCM (100 mL), and the combined organic layers were washed with brine. If indicated, the precipitate (i.e., material not dissolving in either layer) was filtered off and this crude product was purified by NP column chromatography (EtOAc in *c*Hex). After drying the organic layer over Na_2_SO_4_, it was filtered through a silica pad eluting with DCM. The filtrate was concentrated and dried *in vacuo* to yield the corresponding product.

#### General Procedure G: Synthesis of **8–35** (Scheme 1)

The respective primary amine (0.9–1.5 equiv) was added to a mixture of the corresponding dichloroquinazoline (1.0 equiv) and DIPEA (2.1–3.1 equiv) in the indicated volume of EtOAc. The reaction mixture was stirred at RT until TLC analysis indicated complete conversion to the corresponding 4-substituted quinazoline intermediate (typically 3–6 h). If indicated, the reaction was conducted in THF, and the intermediate was isolated. *N*-methylpiperazine (10–11 equiv) was added and the reaction mixture was heated at 120 °C for 10 min under microwave irradiation. The obtained solution was diluted with EtOAc (50 mL) and washed with H_2_O (50 mL) and brine (50 mL). After drying the organic layer over Na_2_SO_4_, the crude product was purified using NP column chromatography (EtOAc:MeOH:TEA 90:5:5 in *c*Hex) to yield the corresponding product. If indicated, the product was converted to its corresponding HCl salt using 4 N HCl in dioxane or to its corresponding fumarate using fumaric acid.

#### General Procedure H: Synthesis of **36a–b** (Scheme 2)

The respective aldehyde (1.0 equiv) was added to a solution of NH_4_OAc (0.3 equiv) in the indicated volume of CH_3_NO_2_. The reaction mixture was stirred at 90 °C under microwave irradiation until LCMS indicated complete conversion (typically 1 h). The reaction mixture was concentrated, and the crude product was purified using NP column chromatography (EtOAc in *c*Hex) to yield the corresponding product.

#### General Procedure I: Synthesis of **37a–b** (Scheme 2)

The respective nitroalkene **36a** or **36b** (1.0 equiv) was dissolved in a 5:1 mixture of Et_2_O and THF (50 mL). This solution was added dropwise to a solution of LiAlH_4_ (2.6 M in THF, 2.6 equiv) in Et_2_O (50 mL) at 0 °C. After stirring the reaction mixture overnight at RT, excess LiAlH_4_ was quenched by careful addition of H_2_O at 0 °C. **CAUTION: gas evolution**. After additional stirring for 30 min at 0 °C, the reaction mixture was filtered through Celite. The filtrate was dried over Na_2_SO_4_. The reaction mixture was concentrated, and the crude product was purified using NP column chromatography (DCM:MeOH:TEA 90:5:5 in DCM) to yield the corresponding product.

#### General Procedure J: Synthesis of **38a–b** (Scheme 2)

The respective aldehyde (1.0 equiv) was dissolved in EtOH (5 mL). H_2_NOH·HCl (1.8 equiv) and NaOAc (2.0 equiv) were added. After stirring the reaction mixture for 72 h at RT, the reaction mixture was concentrated, and the residue was suspended in H_2_O (5 mL). The aqueous phase was extracted thrice with EtOAc (5 mL). The combined organic phases were dried over Na_2_SO_4_ and concentrated to yield the corresponding product.

#### General Procedure K: Synthesis of **39a–b** (Scheme 2)

The respective oxime **38a** or **38b** (1.0 equiv) was dissolved in AcOH (3 mL), and Zn (1.0–6.0 equiv) was added. The reaction mixture was stirred at 90 °C under microwave irradiation until LCMS indicated complete conversion (typically 6 h). The reaction mixture was filtered through Celite and concentrated, to give a crude product that was purified by SPE using Si-propylsulfonic acid as stationary phase. MeOH (5 mL) was used to elute impurities and 7 M NH_3_ in MeOH (5 mL) was used to elute the desired product.

#### General Procedure L: Synthesis of **44–56** (Scheme 2)

The respective amine (1.0–2.6 equiv) was added to a mixture of dichloride **43** (1.0 equiv) and DIPEA (1.5–5.0 equiv) in the indicated volume of either dioxane or MeCN. The reaction mixture was stirred at 70 °C under microwave irradiation until LCMS indicated complete conversion (typically 30–60 min). The reaction mixture was concentrated and purified by NP column chromatography (EtOAc in *c*Hex) to yield the corresponding product. If indicated, an aqueous wash was performed before column chromatography (typically H_2_O or sat. aq. NaHCO_3_). If indicated, an alternative solvent system for NP column chromatography or precipitation was used.

#### General Procedure M: Synthesis of **60–75** (Scheme 2)

The respective monochloride **44–59** (1.0 equiv) was added to a mixture of *N*-methylpiperazine (1.8–10 equiv) and DIPEA (2.0–3.7 equiv) in the indicated volume of dioxane. The reaction mixture was stirred at 150 °C under microwave irradiation until LCMS indicated complete conversion (typically 1–2 h). The obtained mixture was diluted with EtOAc (50 mL) and washed with H_2_O (50 mL) and brine (50 mL). If indicated, a basic wash was performed (either sat. aq. NaHCO_3_ (50 mL) or 1 M aq. NaOH (50 mL)). After drying the organic phase over Na_2_SO_4_, the filtrate was concentrated to yield the corresponding product. If indicated, the crude product was further purified using NP column chromatography (EtOAc:MeOH:TEA 90:5:5 in *c*Hex or MeOH in DCM) to yield the corresponding product. If indicated, the product was converted to its corresponding HCl salt using 4 N HCl in dioxane or to its corresponding fumarate using fumaric acid in MeOH.

#### General Procedure N: Synthesis of **57–59** (Scheme 2)

The respective primary amine (1.0 equiv) was added to a mixture of CH_2_O (1.2 eq, either paraformaldehyde or 37% aq. solution as indicated) in DCE or DCM (10 mL). The reaction mixture was heated at reflux until LCMS indicated conversion (typically for 1–16 h). After cooling, Na_2_SO_4_ was added, and the reaction mixture was filtered. The filtrate was concentrated. The residue was dissolved in a 3 M HCl solution in DMF/H_2_O (4 mL, prepared by diluting conc. aq. HCl with 3 vol equiv of DMF). After stirring the reaction mixture at either RT for 3 h or at reflux for 16 h (as indicated), the reaction mixture was either (as indicated) diluted with DCM (50 mL) and washed with sat. aq. NaHCO_3_ (50 mL), or diluted with 1 M NaOH (50 mL) and extracted thrice with DCM (50 mL). The organic phase was dried over Na_2_SO_4_ and concentrated to yield a crude bicyclic intermediate, that was used in the next step without further purification. Specifically, the crude intermediate (1.2–1.4 equiv) was added to a mixture of dichloride **43** (1.0 equiv), DIPEA (2.1 equiv) and MeCN (5 mL). The reaction mixture was stirred at 70 °C under microwave irradiation until LCMS indicated complete conversion (typically 30 min). The reaction mixture was concentrated and purified by NP column chromatography (EtOAc in *c*Hex) to yield the corresponding product.
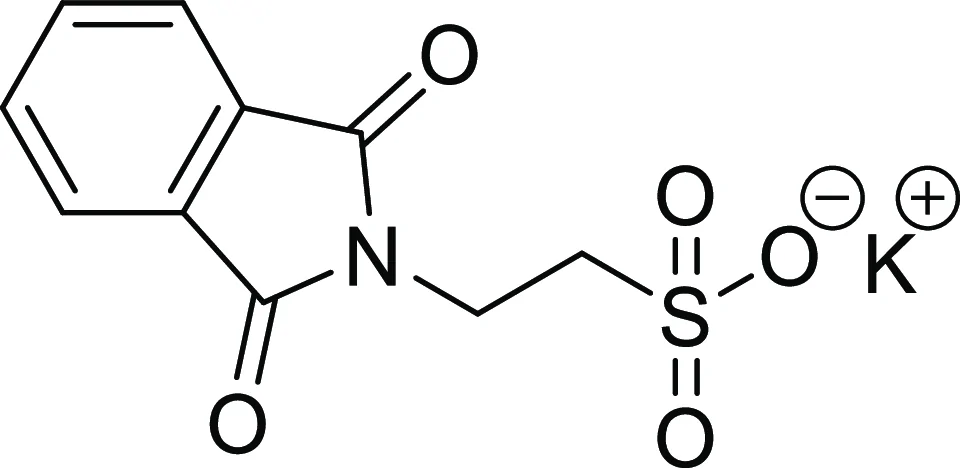


**Potassium 2-(1,3-dioxoisoindolin-2-yl)­ethane-1-sulfonate (2a).**Prepared according to general procedure A from 2-aminoethane-1-sulfonic acid (100.0 g, 0.799 mol, 1.0 equiv), KOAc (78.4 g, 0.799 mol, 1.0 equiv), and phthalic anhydride (118 g, 0.799 mol, 1.0 equiv) in AcOH (500 mL) to yield the title compound as a white solid (189 g, 81%). ^**1**^ **H NMR** (D_2_O, 500 MHz): δ 7.83–7.66 (m, 4H), 3.99 (t, J = 6.8 Hz, 2H), 3.19 (t, J = 6.8 Hz, 2H).
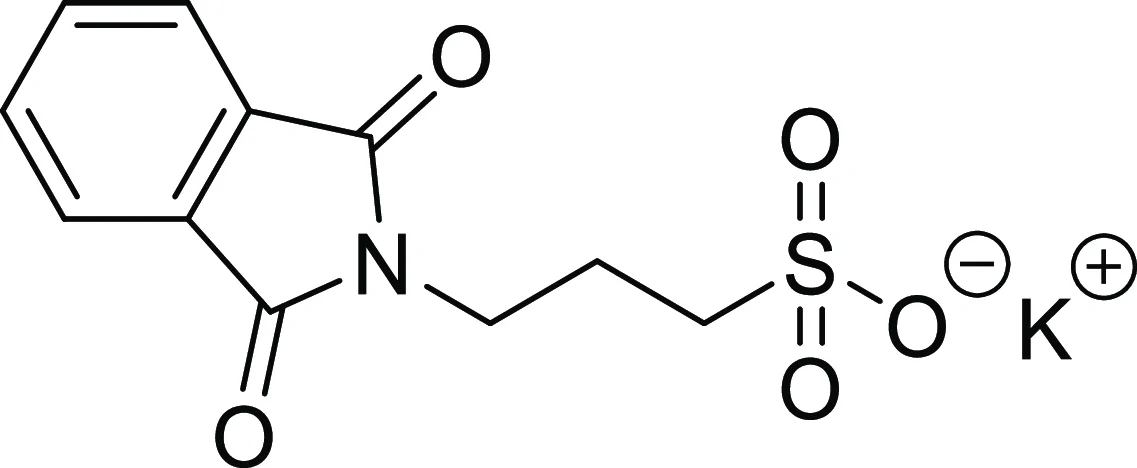


**Potassium 3-(1,3-dioxoisoindolin-2-yl)­propane-1-sulfonate (2b).**Prepared according to general procedure A from 3-amino-1-propanesulfonic acid (3.00 g, 21.6 mmol, 1.0 equiv), KOAc (2.12 g, 21.6 mmol, 1.0 equiv), and phthalic anhydride (3.19 g, 21.6 mmol, 1.0 equiv) in AcOH (13.5 mL) to yield the title compound as a white solid in quantitative yield (6.09 g). ^**1**^ **H NMR** (D_2_O, 200 MHz): δ 7.74 (s, 4H), 3.71 (t, J = 6.9 Hz, 2H), 2.97–2.89 (m, 2H), 2.12–1.98 (m, 2H).
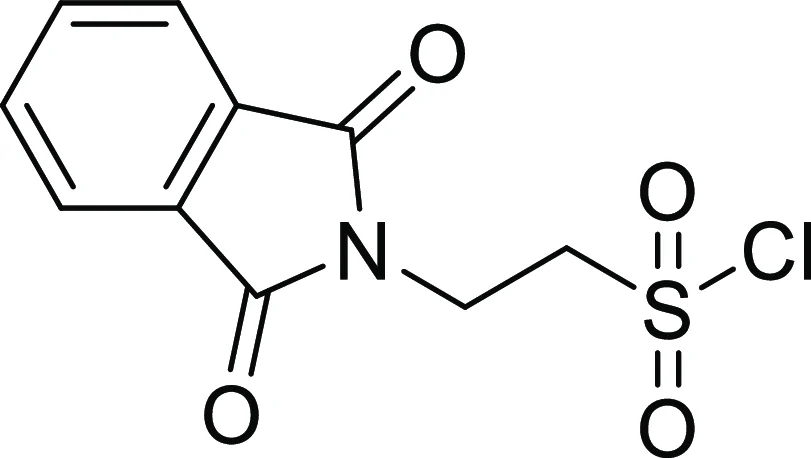


**2-(1,3-Dioxoisoindolin-2-yl)­ethane-1-sulfonyl chloride (3a).**Prepared according to general procedure B from sulfonate **2a** (50.0 g, 0.170 mol, 1.0 equiv) and PCl_5_ (49.7 g, 0.398 mol, 1.4 equiv) in PhMe (500 mL) to yield the title compound as a gray solid (37.0 g, 79%). ^**1**^ **H NMR** (CDCl_3_, 500 MHz): δ 7.89 (dd, J = 5.5, 3.1 Hz, 2H), 7.77 (dd, J = 5.5, 3.1 Hz, 2H), 4.36 (t, J = 6.5 Hz, 2H), 4.08 (t, J = 6.5 Hz, 2H).
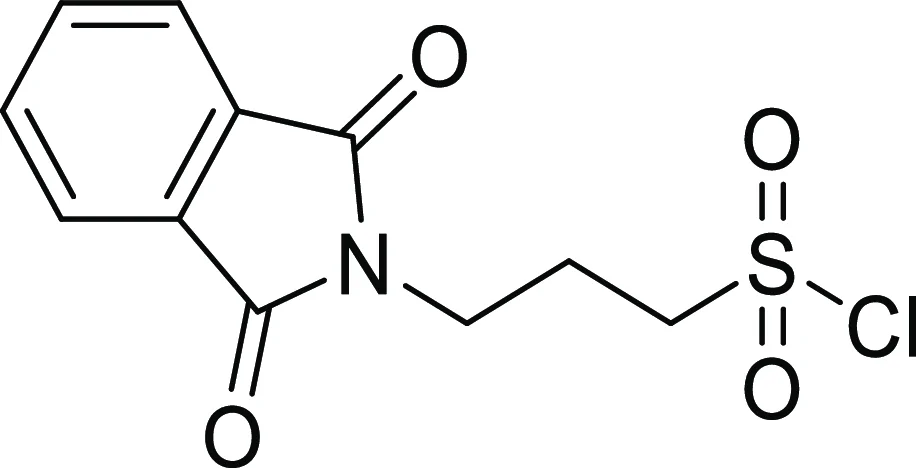


**3-(1,3-Dioxoisoindolin-2-yl)­propane-1-sulfonyl chloride (3b).**Prepared according to general procedure B from sulfonate **2b** (6.00 g, 20.9 mmol, 1.0 equiv) and PCl_5_ (8.22 g, 39.4 mmol, 1.4 equiv) in PhMe (25 mL) to yield the title compound as a white solid (5.64 g, 94%). ^**1**^ **H NMR** (CDCl_3_, 200 MHz): δ 7.88–7.81 (m, 2H), 7.78–7.71 (m, 2H), 3.87 (t, J = 6.5 Hz, 2H), 3.77–3.69 (m, 2H), 2.48–2.34 (m, 2H).
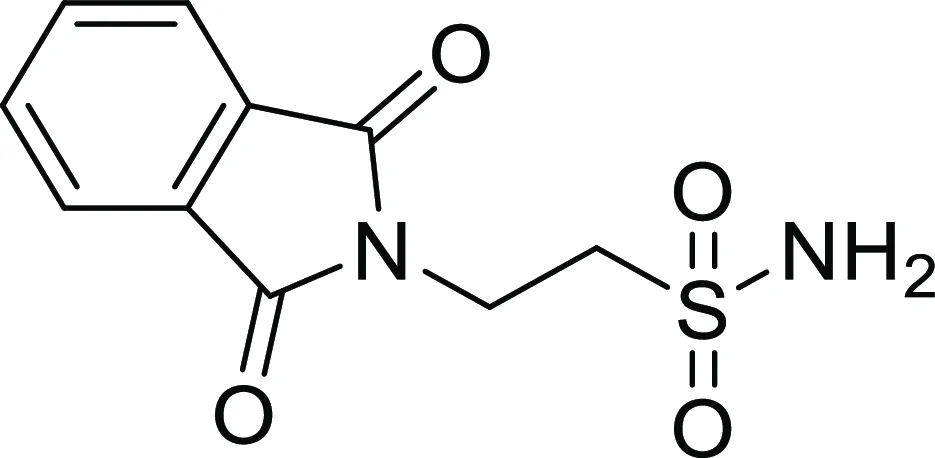


**2-(1,3-Dioxoisoindolin-2-yl)­ethane-1-sulfonamide (4a).**Sulfonyl chloride **3a** (2.00 g, 7.31 mmol, 1.0 equiv) was added portion wise to a solution of 0.5 M NH_3_ in dioxane (15 mL, 7.50 mmol, 1.0 equiv). After stirring the suspension for 48 h at RT, the reaction mixture was poured into H_2_O (50 mL) and the resulting precipitate was collected by vacuum filtration to yield the title compound as a white solid (1.52 g, 78%). ^**1**^ **H NMR** (DMSO-d_6_, 600 MHz): δ 7.91–7.86 (m, 2H), 7.86–7.82 (m, 2H), 7.03 (s, 2H), 4.00–3.93 (m, 2H), 3.36–3.31 (m, 2H).
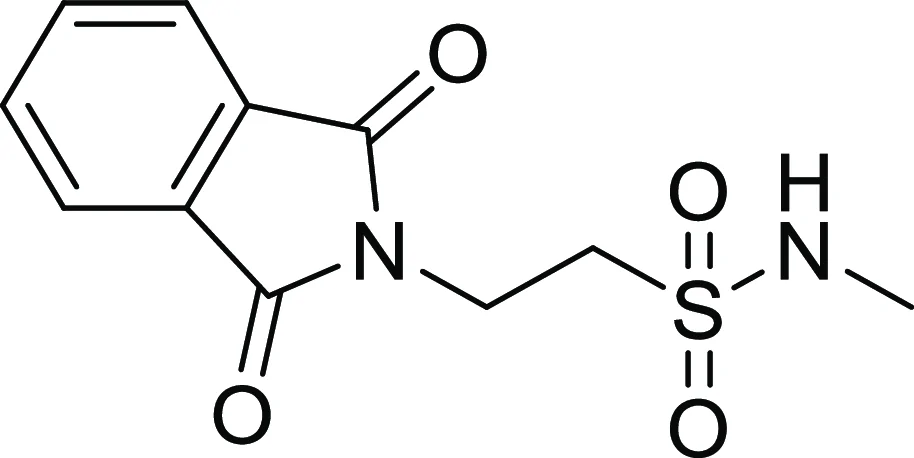


**2-(1,3-Dioxoisoindolin-2-yl)-** ***N*** **-methylethane-1-sulfonamide (4b).**Sulfonyl chloride **3a** (2.00 g, 7.31 mmol, 1.0 equiv) was added portion wise to a solution of 2.0 M MeNH_2_ in THF (15.0 mL, 30.0 mmol, 4.1 equiv). After stirring the suspension for 48 h at RT, the reaction mixture was poured in H_2_O (50 mL) causing the formation of a precipitate. The crude product was collected by vacuum filtration and recrystallized from EtOH:H_2_O 50:1 to yield the title compound as a white solid (1.04 g, 50%). ^1^H NMR (CDCl_3_, 500 MHz): δ 7.91–7.79 (m, 2H), 7.79–7.66 (m, 2H), 4.77 (q, J = 5.3 Hz, 1H), 4.16–4.06 (m, 2H), 3.44–3.32 (m, 2H), 2.84 (d, J = 5.1 Hz, 3H).
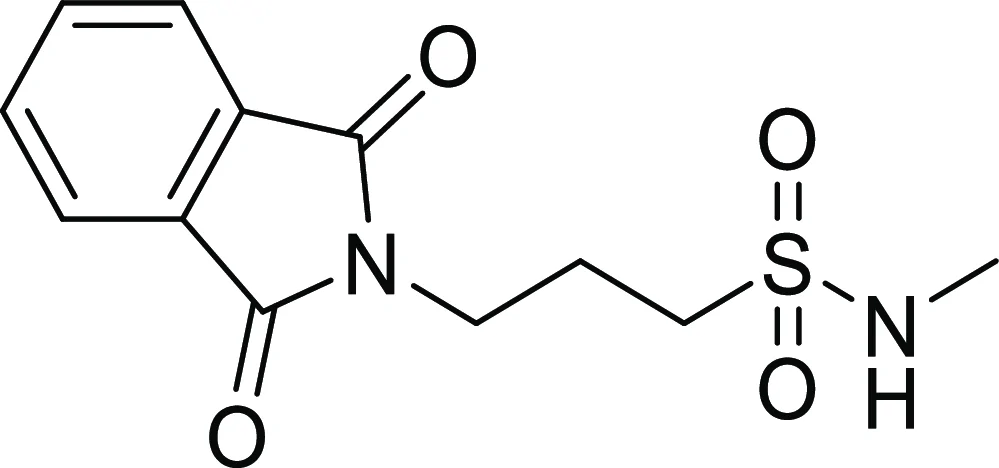


**2-(1,3-Dioxoisoindolin-2-yl)-** ***N*** **-methylpropane-1-sulfonamide (4c).**Prepared according to general procedure C from sulfonyl chloride **3b** (2.50 g, 8.69 mmol, 1.0 equiv) and MeNH_2_·HCl (1.76 g, 26.1 mmol, 3.0 equiv) in CHCl_3_ (17 mL) to yield the title compound as a white solid (854 mg, 35%). **^1^H NMR** (CDCl_3_, 200 MHz): δ 7.82–7.76 (m, 2H), 7.72–7.64 (m, 2H), 3.78 (t, J = 6.8 Hz, 2H), 3.07–2.99 (m, 2H), 2.74 (d, J = 5.2 Hz, 3H), 2.21–1.18 (m, 2H).
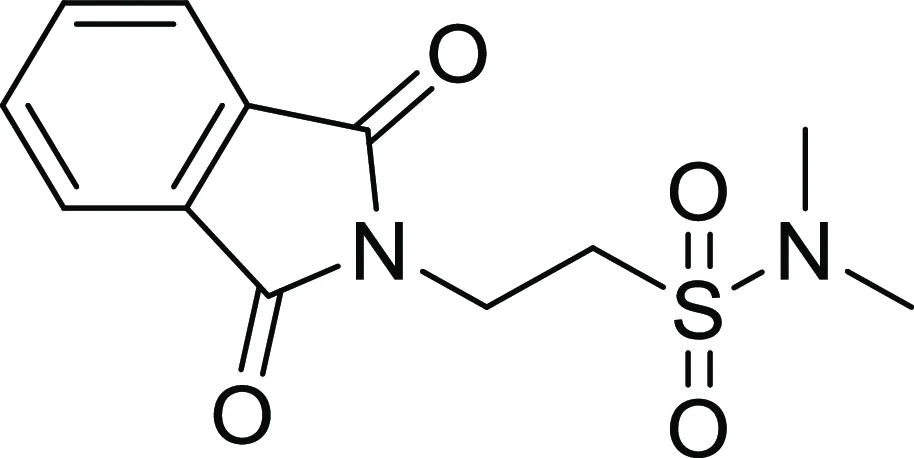


**2-(1,3-Dioxoisoindolin-2-yl)-*N*,*N*-dimethylethane-1-sulfonamide (4d).**Sulfonyl chloride **3a** (2.00 g, 7.31 mmol, 1.0 equiv) was added portion wise to a solution of 2 M (Me)_2_NH in THF (15.0 mL, 30.0 mmol, 4.1 equiv). After stirring the mixture for 48 h at RT, the mixture was poured in H_2_O (50 mL). The solid was collected by vacuum filtration and recrystallized from EtOH:H_2_O (50:1) to yield the title compound as a white solid (1.03 g, 50%). ^**1**^ **H NMR** (CDCl_3_, 200 MHz): δ 7.87–7.79 (m, 2H), 7.75–7.66 (m, 2H), 4.11 (t, J = 7.1 Hz, 2H), 3.30 (t, J = 7.1 Hz, 2H), 2.87 (s, 6H).
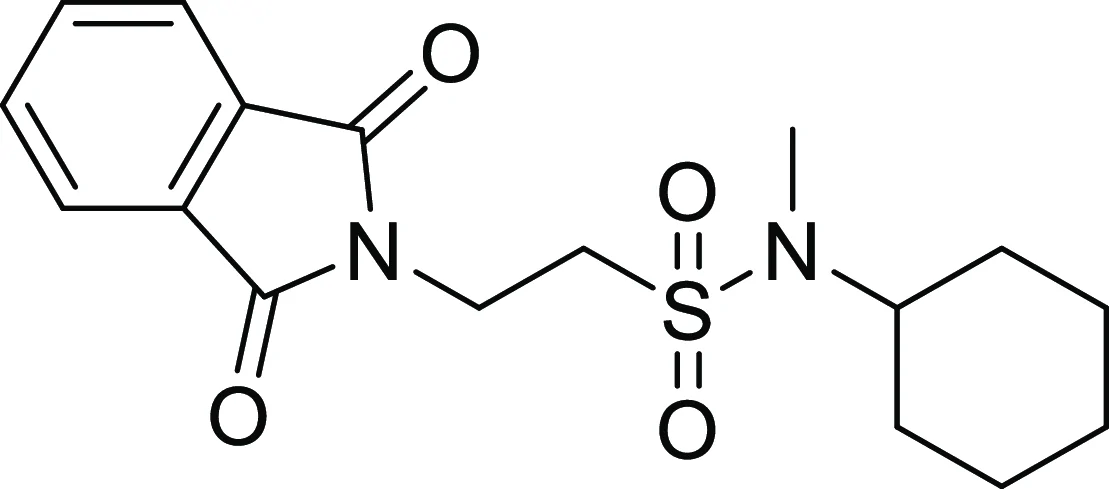


***N*-Cyclohexyl-2-(1,3-dioxoisoindolin-2-yl)-** ***N*** **-methylethane-1-sulfonamide (4e).**Prepared according to general procedure C from sulfonyl chloride **3a** (2.00 g, 7.31 mmol, 1.0 equiv) and *N*-methylcyclohexanamine (2.90 mL, 22.0 mmol, 3.0 equiv) in CHCl_3_ (15 mL) to yield the title compound as a white solid (1.19 g, 46%). ^**1**^ **H NMR** (DMSO-d_6_, 500 MHz): δ 7.92–7.88 (m, 2H), 7.87–7.83 (m, 2H), 3.94–3.88 (m, 2H), 3.51 (tt, J = 11.9, 3.8 Hz, 1H), 3.42–3.36 (m, 2H), 2.71 (s, 3H), 1.77–1.61 (m, 4H), 1.57–1.54 (m, 1H), 1.51–1.46 (m, 2H), 1.34–1.30 (m, 2H), 1.10–0.99 (m, 1H).
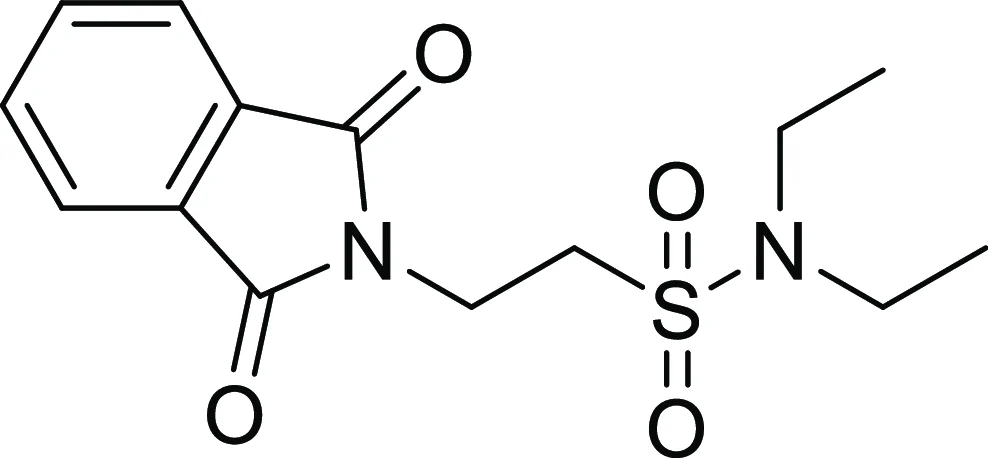


**2-(1,3-Dioxoisoindolin-2-yl)-*N*,*N*-diethylethane-1-sulfonamide (4f).**Prepared according to general procedure C from sulfonyl chloride **3a** (2.00 g, 7.31 mmol, 1.0 equiv) and Et_2_NH (2.50 mL, 24.1 mmol, 3.0 equiv) in DCM (15 mL) to yield the title compound as a white solid (1.40 g, 62%). ^**1**^ **H NMR** (DMSO-d_6_, 500 MHz): δ 7.90 (dd, J = 5.4, 3.0 Hz, 2H), 7.79–7.74 (m, 2H), 4.21–4.12 (m, 2H), 3.41–3.31 (m, 6H), 1.25 (t, J = 7.1 Hz, 6H).
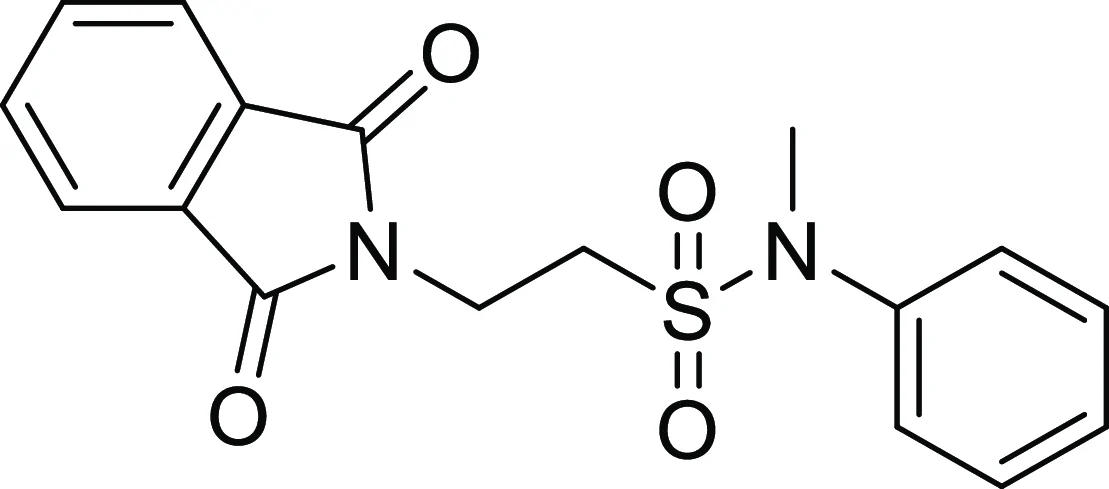


**2-(1,3-Dioxoisoindolin-2-yl)-** ***N*** **-methyl-*N*-phenylethane-1-sulfonamide (4g).**Prepared according to general procedure C from sulfonyl chloride **3a** (2.00 g, 7.31 mmol, 1.0 equiv) and *N*-methylaniline (2.38 mL, 22.0 mmol, 3.0 equiv) in CHCl_3_ (15 mL) to yield the title compound as a white solid (1.40 g, 56%). ^**1**^ **H NMR** (CDCl_3_, 200 MHz): δ 7.42–7.36 (m, 4H), 7.33–7.29 (m, 5H), 4.13 (t, J = 7.2 Hz, 2H), 3.44–3.38 (m, 5H).
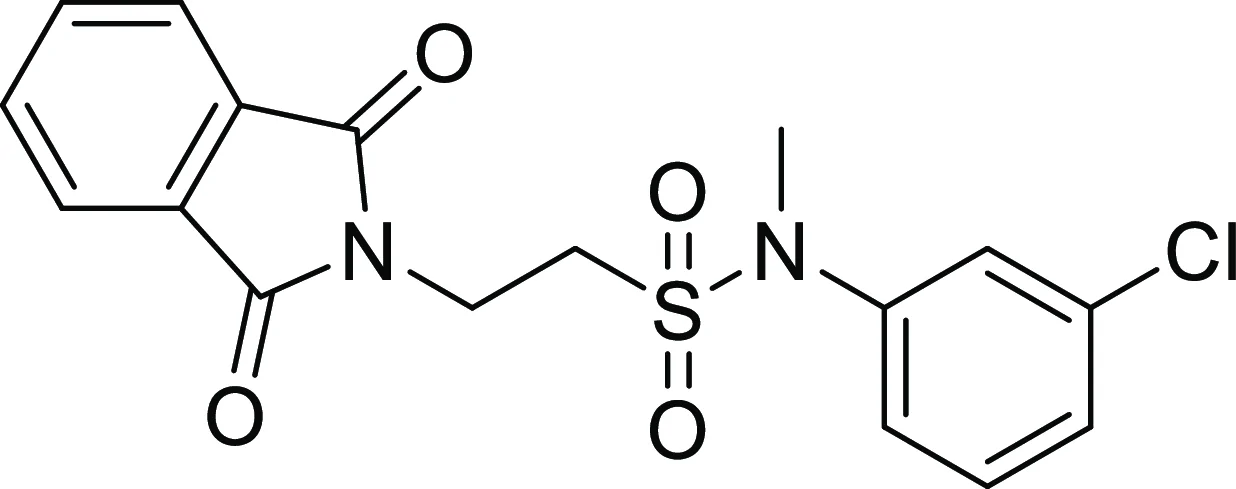


***N*-(3-Chlorophenyl)-2-(1,3-dioxoisoindolin-2-yl)-** ***N*** **-methylethane-1-sulfonamide (4h).**Prepared according to general procedure C from sulfonyl chloride **3a** (2.00 g, 7.31 mmol, 1.0 equiv) and 3-chloro-*N*-methyl-aniline (2.68 mL, 22.0 mmol, 3.0 equiv) in CHCl_3_ (15 mL) to yield the title compound as a white solid (1.89 g, 68%). ^**1**^ **H NMR** (CDCl_3_, 200 MHz): δ 7.88–7.85 (m, 2H), 7.75–7.72 (m, 2H), 7.38–7.27 (m, 4H), 4.12 (t, J = 7.2 Hz, 2H), 3.44–3.38 (m, 5H).
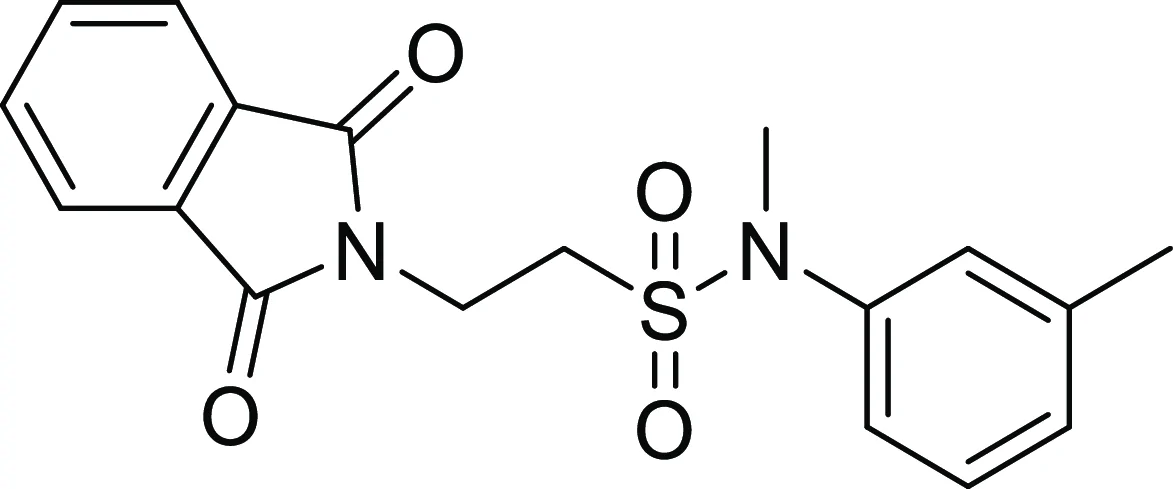


**2-(1,3-Dioxoisoindolin-2-yl)-** ***N*** **-methyl-*N*-(m-tolyl)­ethane-1-sulfonamide (4i).**Prepared according to general procedure C from sulfonyl chloride **3a** (2.00 g, 7.31 mmol, 1.0 equiv) and *N*,3-dimethylaniline (1.86 mL, 14.7 mmol, 2.0 equiv) in CHCl_3_ (15 mL) to yield the title compound as a white solid (1.59 g, 61%). ^**1**^ **H NMR** (CDCl_3_, 200 MHz): δ 7.89–7.84 (m, 2H), 7.78–7.73 (m, 2H), 7.28–7.26 (m, 3H), 7.13–7.10 (m, 1H), 4.14 (t, J = 7.2 Hz, 2H), 3.45–3.38 (m, 5H), 2.38 (s, 3H).
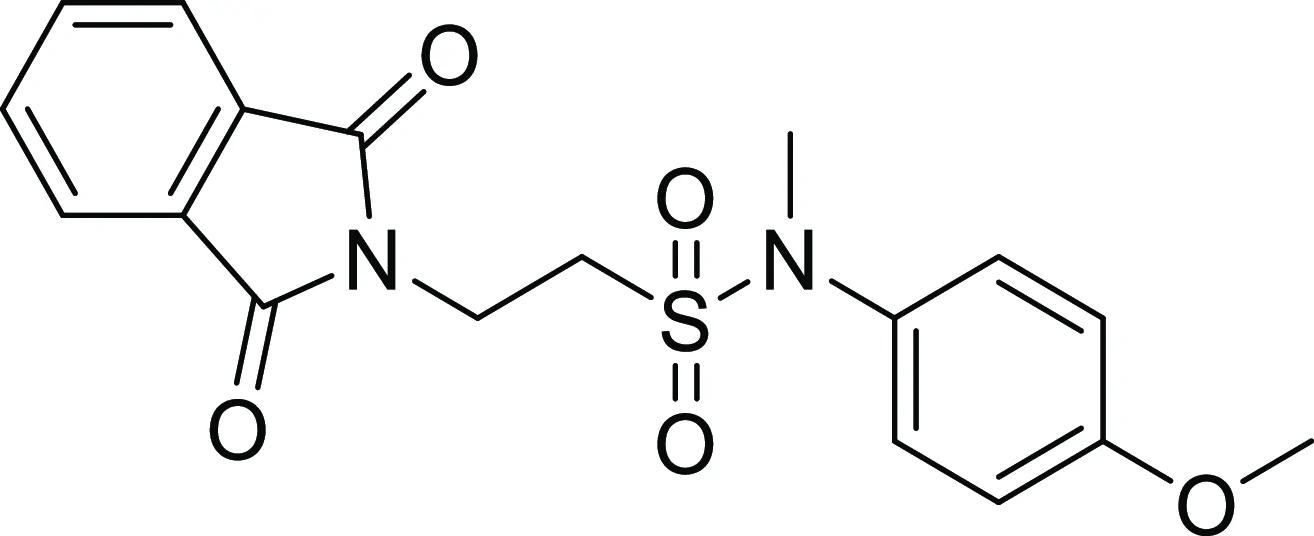


**2-(1,3-Dioxoisoindolin-2-yl)-*N*-(4-methoxyphenyl)-** ***N*** **-methylethane-1-sulfonamide (4j).**Prepared according to general procedure C from sulfonyl chloride **3a** (2.00 g, 7.31 mmol, 1.0 equiv) and 4-methoxy-*N*-methylaniline (2.82 mg, 20.5 mmol, 2.8 equiv) in CHCl_3_ (15 mL) to yield the title compound as a white solid in quantitative yield (2.74 g). ^**1**^ **H NMR** (CDCl_3_, 200 MHz): δ 7.86–7.82 (m, 2H), 7.76–7.71 (m, 2H), 7.37–7.32 (m, 2H), 6.91–6.87 (m, 2H), 4.13 (t, J = 7.4 Hz, 2H), 3.80 (s, 3H), 3.41–3.34 (m, 5H).
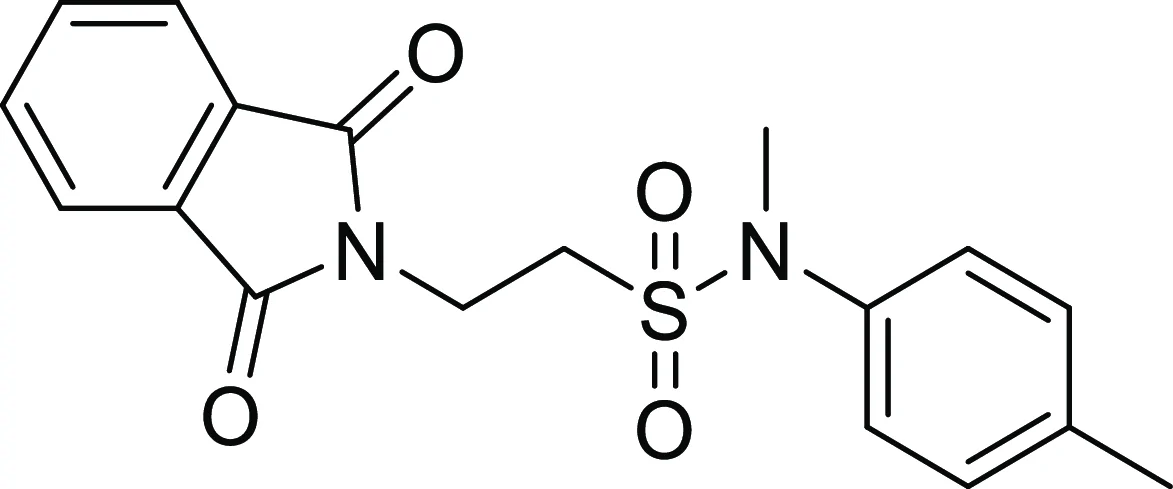


**2-(1,3-Dioxoisoindolin-2-yl)-** ***N*** **-methyl-*N*-(p-tolyl)­ethane-1-sulfonamide (4k).**Prepared according to general procedure C from sulfonyl chloride **3a** (2.00 g, 7.31 mmol, 1.0 equiv) and *N*,4-dimethylaniline (2.85 mL, 22.0 mmol, 3.0 equiv) in CHCl_3_ (15 mL) to yield the title compound as a white solid (1.55 g, 59%). ^**1**^ **H NMR** (CDCl_3_, 200 MHz): δ 7.87–7.81 (m, 2H), 7.76–7.71 (m, 2H), 7.33–7.29 (m, 2H), 7.20–7.16 (m, 2H), 4.12 (t, J = 7.4 Hz, 2H), 3.42–3.36 (m, 5H), 2.34 (s, 3H).
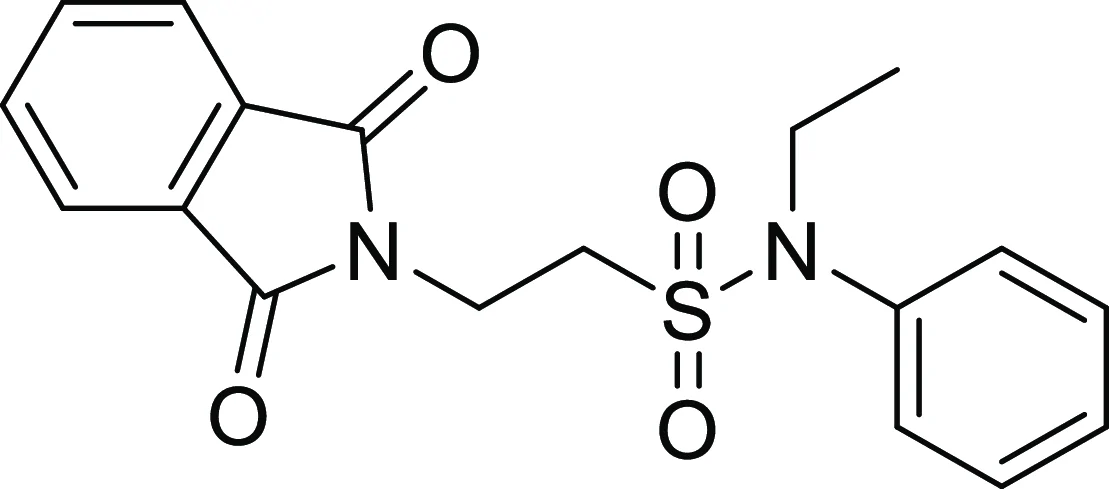


**2-(1,3-Dioxoisoindolin-2-yl)-*N*-ethyl-*N*-phenylethane-1-sulfonamide (4l).**Prepared according to general procedure C from sulfonyl chloride **3a** (3.00 g, 11.0 mmol, 1.0 equiv) and *N*-ethylaniline (4.16 mL, 33.0 mmol, 3.0 equiv) in CHCl_3_ (22 mL) to yield the title compound as a white solid (1.94 g, 49%). ^**1**^ **H NMR** (CD_3_OD, 200 MHz): δ 7.89–7.80 (m, 4H), 7.44–7.35 (m, 5H), 4.11 (t, J = 6.9 Hz, 2H), 3.79 (q, J = 7.0 Hz, 2H), 3.48 (t, J = 6.9 Hz, 2H), 1.10 (t, J = 7.0 Hz, 3H).
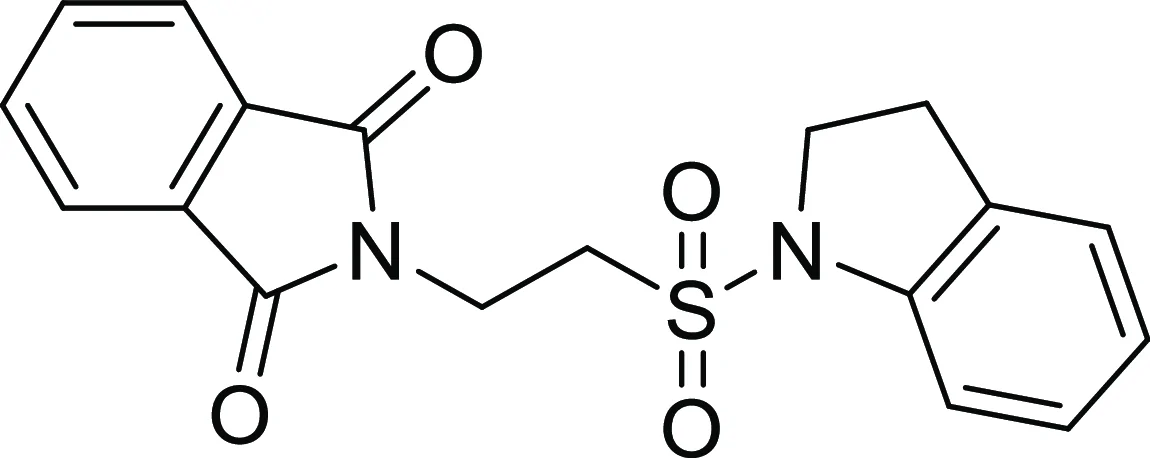


**2-(2-(Indolin-1-ylsulfonyl)­ethyl)­isoindoline-1,3-dione (4m).**Prepared according to general procedure C from sulfonyl chloride **3a** (2.00 g, 7.31 mmol, 1.0 equiv) and indoline (2.46 mL, 22.0 mmol, 3.0 equiv) in CHCl_3_ (15 mL) to yield the title compound as a white solid (2.31 g, 89%). ^**1**^ **H NMR** (CDCl_3_, 200 MHz): δ 7.84–7.69 (m, 4H), 7.35 (d, J = 8.3 Hz, 1H), 7.18–7.12 (m, 2H), 6.99–6.92 (m, 1H), 4.16 (t, J = 7.0 Hz, 2H), 4.07 (t, J = 8.5, 2H), 3.49 (t, J = 7.0 Hz, 2H), 3.15 (t, J = 8.5 Hz, 2H).
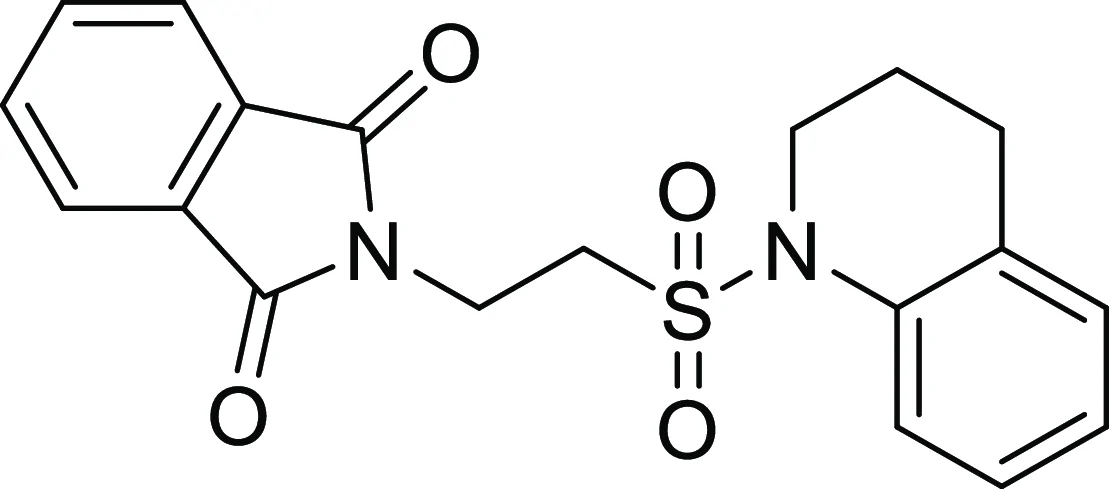


**2-(2-((3,4-Dihydroquinolin-1­(2H)-yl)­sulfonyl)­ethyl)­isoindoline-1,3-dione (4n).**Prepared according to general procedure C from sulfonyl chloride **3a** (3.00 g, 11.0 mmol, 1.0 equiv) and 1,2,3,4-tetrahydroquinoline (4.14 mL, 33.0 mmol, 3.0 equiv) in CHCl_3_ (22 mL) to yield the title compound as a white solid (1.51 g, 37%). ^**1**^ **H NMR** (DMSO-d_6_, 500 MHz): δ 7.91–7.83 (m, 4H), 7.50 (dd, J = 8.6, 1.2 Hz, 1H), 7.17 (app ddd, J = 7.3, 4.1, 2.4 Hz, 2H), 7.08 (app td, J = 7.3, 1.2 Hz, 1H), 3.94 (app dd, J = 8.0, 6.2 Hz, 2H), 3.76–3.67 (m, 2H), 3.57 (app dd, J = 8.0, 6.2 Hz, 2H), 2.81 (t, J = 6.6 Hz, 2H), 2.00–1.87 (m, 2H).
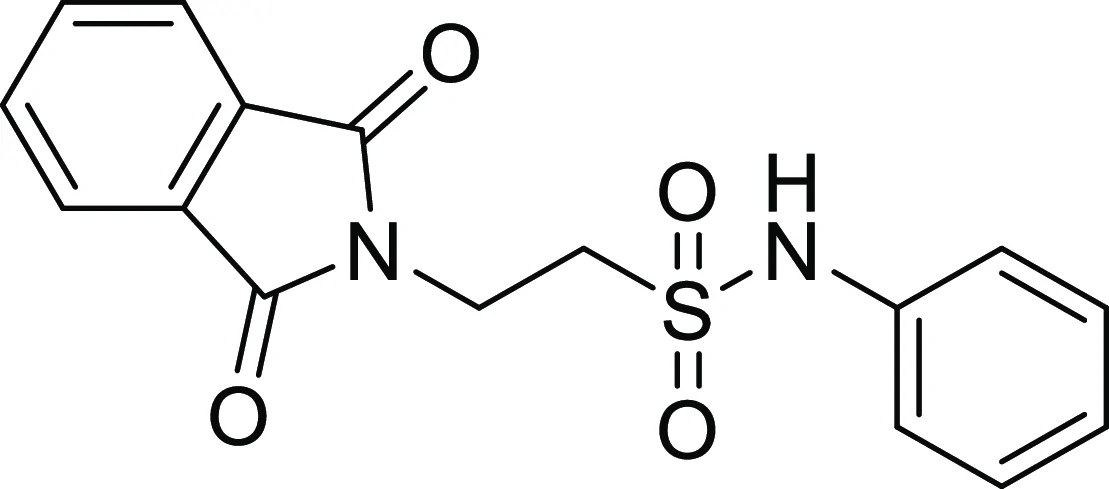


**2-(1,3-Dioxoisoindolin-2-yl)-*N*-phenylethane-1-sulfonamide (4o).**Prepared according to general procedure C from sulfonyl chloride **3a** (2.00 g, 7.31 mmol, 1.0 equiv) and PhNH_2_ (2.29 g, 24.6 mmol, 3.0 equiv) in CHCl_3_ (15 mL) to yield the title compound as a white solid (1.76 g, 73%). **^1^H NMR** (DMSO-d_6_, 500 MHz): δ 9.95 (s, 1H), 7.91–7.74 (m, 4H), 7.31 (dt, J = 7.4, 1.9 Hz, 2H), 7.20 (dt, J = 7.6, 1.2 Hz, 2H), 7.10 (tt, J = 7.3, 1.2 Hz, 1H), 4.01–3.88 (m, 2H), 3.49–3.40 (m, 2H).
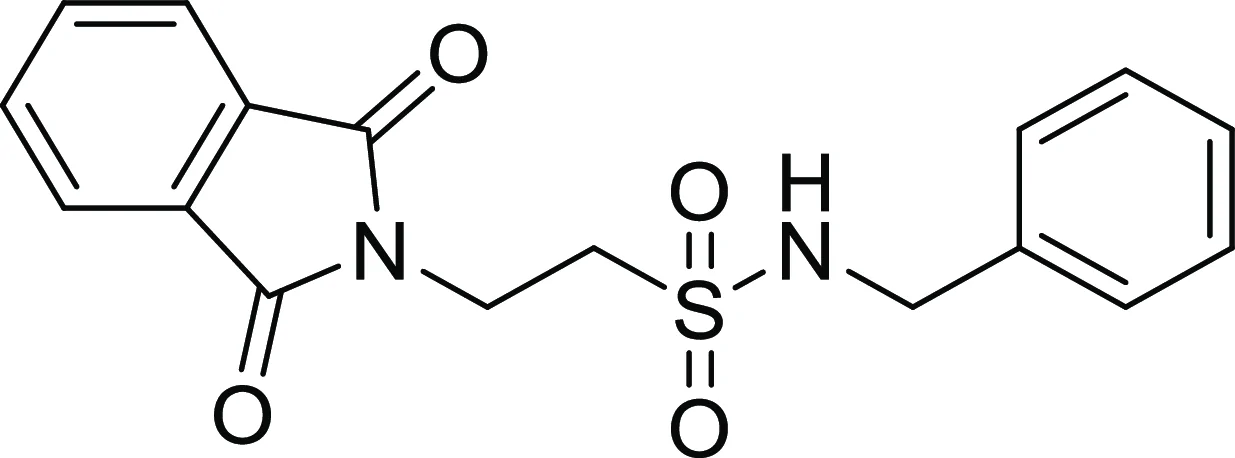


***N*-Benzyl-2-(1,3-dioxoisoindolin-2-yl)­ethane-1-sulfonamide (4p).**Prepared according to general procedure C from sulfonyl chloride **3a** (27.0 g, 98.7 mmol, 1.0 equiv) and PhCH_2_NH_2_ (32.2 mL, 296 mmol, 3.0 equiv) in CHCl_3_ (200 mL) to yield the title compound as a white solid (14.3 g, 74%). ^**1**^ **H NMR** (CDCl_3_, 500 MHz): δ 7.83 (dd, J = 5.3, 3.3 Hz, 2H), 7.72 (dd, J = 5.5, 3.0 Hz, 2H), 7.39–7.26 (m, 5H), 5.26 (t, J = 6.1 Hz, 1H), 4.33 (d, J = 6.1 Hz, 2H), 4.03–3.92 (m, 2H), 3.29–3.19 (m, 2H).
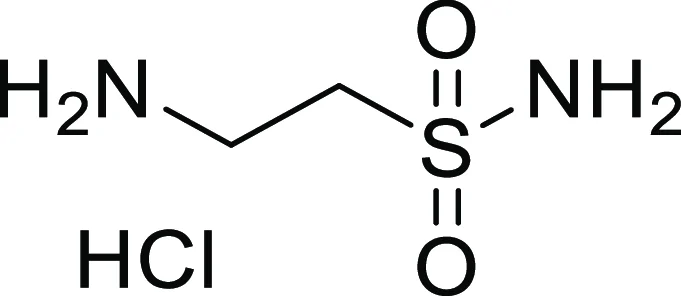


**2-Aminoethane-1-sulfonamide hydrochloride (5a).**Prepared according to general procedure D from phthalimide **4a** (1.52 g, 6.78 mmol, 1.0 equiv) and NH_2_NH_2_·H_2_O (0.360 mL, 7.41 mmol, 1.1 equiv) in EtOH (30 mL) to yield the title compound as white crystals (764 mg, 64%). ^**1**^ **H NMR** (D_2_O, 200 MHz): δ 3.62–3.55 (m, 2H), 3.51–3.44 (m, 2H).
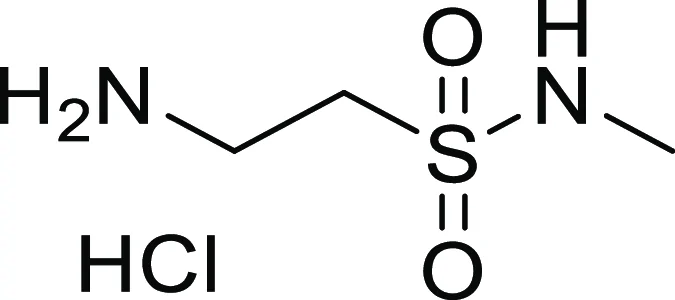


**2-Amino-** ***N*** **-methylethane-1-sulfonamide hydrochloride (5b).**Prepared according to general procedure D from phthalimide **4b** (1.03 g, 3.84 mmol, 1.0 equiv) and NH_2_NH_2_·H_2_O (0.205 mL, 4.22 mmol, 1.1 equiv) in EtOH (30 mL) to yield the title compound as white crystals (415 mg, 72%). ^**1**^ **H NMR** (D_2_O, 200 MHz): δ 3.53–3.46 (m, 2H), 3.43–3.40 (m, 2H), 2.73 (s, 3H).
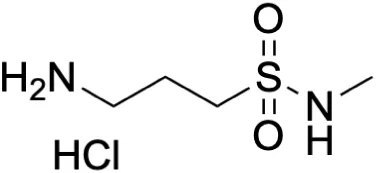


**2-Amino-** ***N*** **-methylpropane-1-sulfonamide hydrochloride (5c).**Prepared according to general procedure D from phthalimide **4c** (847 mg, 3.00 mmol, 1.0 equiv) and NH_2_NH_2_·H_2_O (0.160 mL, 3.30 mmol, 1.1 equiv) in EtOH (30 mL) to yield the title compound as white crystals (500 mg, 88%). ^**1**^ **H NMR** (D_2_O, 200 MHz): δ 3.31 (t, J = 7.5 Hz, 2H), 3.15 (t, J = 7.7 Hz, 2H), 2.72 (s, 3H), 2.13 (app p, J = 7.5 Hz, 2H).
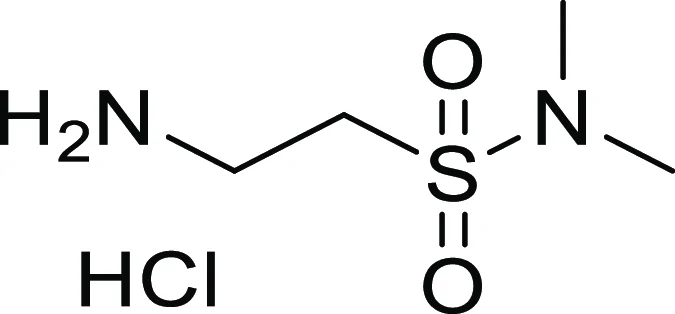


**2-Amino-*N*,*N*-dimethylethane-1-sulfonamide hydrochloride (5d).**Prepared according to general procedure D from phthalimide **4d** (1.03 g, 3.65 mmol, 1.0 equiv) and NH_2_NH_2_·H_2_O (0.195 mL, 4.02 mmol, 1.1 equiv) in EtOH (30 mL) to yield the title compound as white crystals (602 mg, 87%). ^**1**^ **H NMR** (D_2_O, 200 MHz): δ 3.49 (app s, 4H), 2.89 (s, 6H).
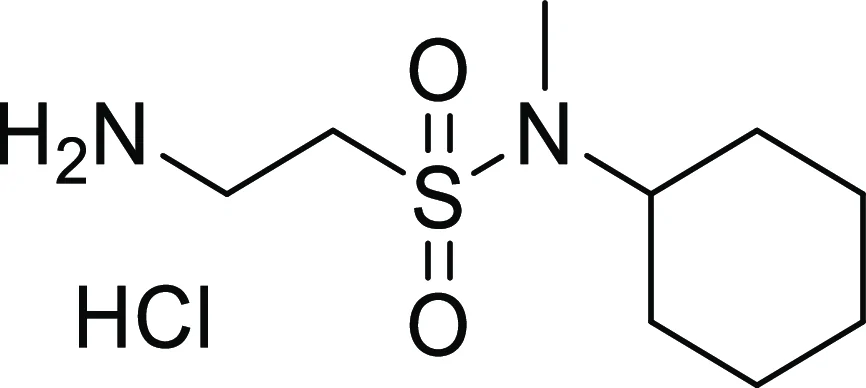


**2-Amino-*N*-cyclohexyl-** ***N*** **-methylethane-1-sulfonamide hydrochloride (5e).**Prepared according to general procedure D from phthalimide **4e** (1.19 g, 3.40 mmol, 1.0 equiv) and NH_2_NH_2_·H_2_O (0.190 mL, 4.08 mmol, 1.2 equiv) in EtOH (30 mL) to yield the title compound as white crystals in quantitative yield (910 mg). ^**1**^ **H NMR** (DMSO-d_6_, 500 MHz): δ 3.52 (app tt, J = 11.9, 3.8 Hz, 1H), 3.46–3.40 (m, 2H), 3.13–3.03 (m, 2H), 2.72 (s, 3H), 1.80–1.70 (m, 2H), 1.67–1.44 (m, 5H), 1.35–1.24 (m, 2H), 1.11–0.99 (m, 1H).
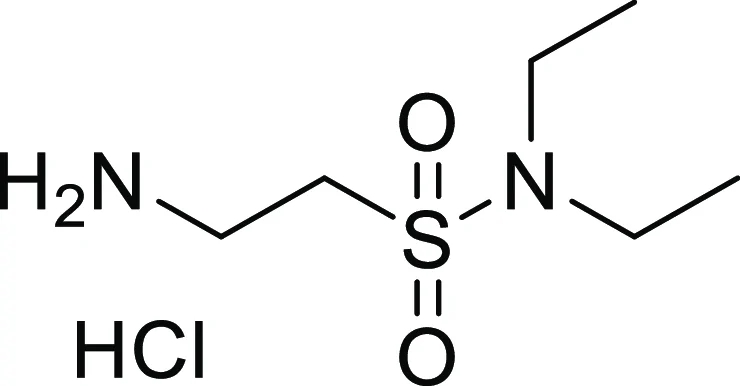


**2-Amino-*N*,*N*-diethylethane-1-sulfonamide hydrochloride (5f).**Prepared according to general procedure D from phthalimide **4f** (1.40 g, 4.51 mmol, 1.0 equiv) and NH_2_NH_2_·H_2_O (0.241 mL, 4.96 mmol, 1.1 equiv) in EtOH (30 mL) to yield the title compound as white crystals (500 mg, 62%). ^**1**^ **H NMR** (DMSO-d_6_, 500 MHz): δ 8.05 (s, 2H), 3.41–3.37 (m, 2H), 3.23 (q, J = 7.0 Hz, 4H), 3.14–3.09 (m, 2H), 1.12 (t, J = 7.1 Hz, 6H).
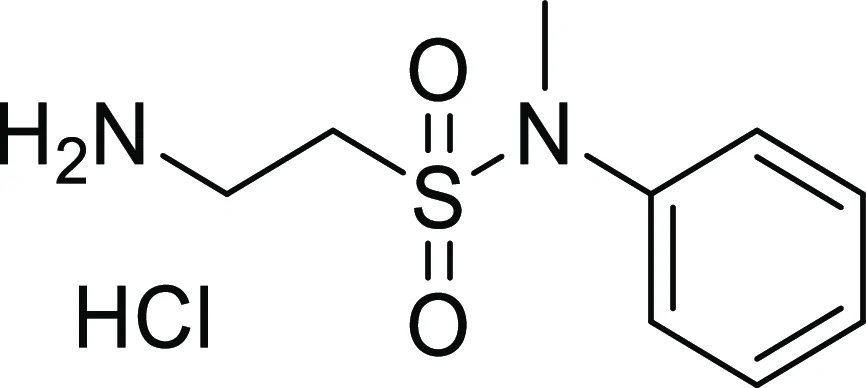


**2-Amino-** ***N*** **-methyl-*N*-phenylethane-1-sulfonamide (5g).**hPrepared according to general procedure D from phtalimide **4g** (4.30 g, 12.5 mmol, 1.0 equiv) and NH_2_NH_2_·H_2_O (0.640 mL, 15.0 mmol, 1.2 equiv) in EtOH (30 mL) to yield the title compound as a white solid (1.04 g, 33%). ^**1**^ **H NMR** (DMSO-d_6_, 500 MHz): δ 7.49–7.40 (m, 4H), 7.34 (tt, J = 7.3, 1.4 Hz, 1H), 3.59–3.51 (m, 2H), 3.30 (s, 3H), 3.14–3.06 (m, 2H).
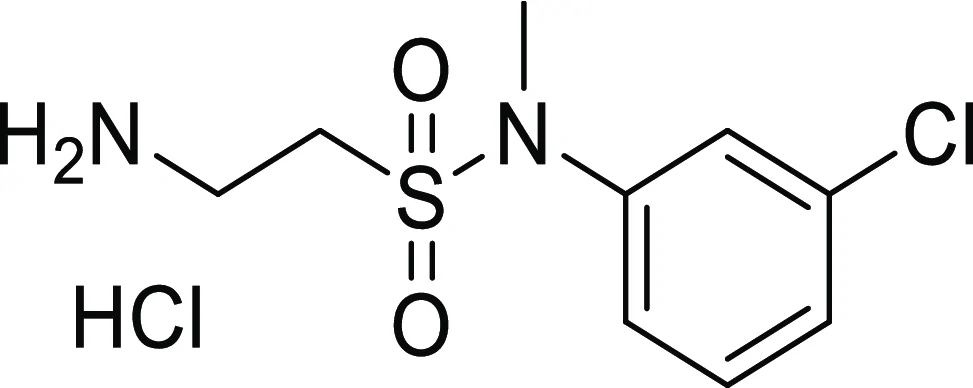


**2-Amino-*N*-(3-chlorophenyl)-** ***N*** **-methylethane-1-sulfonamide hydrochloride (5h).**Prepared according to general procedure D from phthalimide **4h** (253 mg, 0.668 mmol, 1.0 equiv) and NH_2_NH_2_·H_2_O (0.130 mL, 2.67 mmol, 4.0 equiv) in EtOH (30 mL) to yield the title compound as a white solid (140 mg, 84%). ^**1**^ **H NMR** (DMSO-d_6_, 600 MHz) δ 7.54–7.50 (m, 1H), 7.45–7.38 (m, 2H), 7.38–7.34 (m, 1H), 3.27 (s, 3H), 3.24–3.20 (m, 2H), 2.91–2.84 (m, 2H).
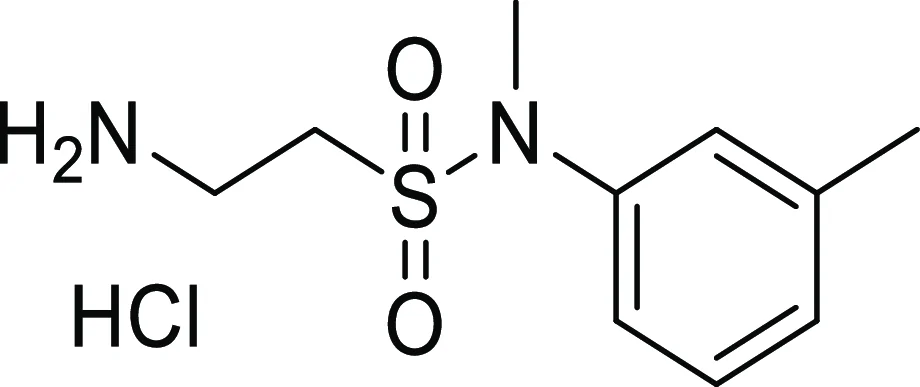


**2-Amino-** ***N*** **-methyl-*N*-(m-tolyl)­ethane-1-sulfonamide hydrochloride (5i).**Prepared according to general procedure D from phthalimide **4i** (688 mg, 1.92 mmol, 1.0 equiv) and NH_2_NH_2_·H_2_O (0.372 mL, 7.68 mmol, 4.0 equiv) in EtOH (30 mL) to yield the title compound as a yellow solid (322 mg, 63%). ^**1**^ **H NMR** (DMSO-d_6_, 500 MHz): δ 7.28 (t, *J* = 7.7 Hz, 1H), 7.24–7.18 (m, 2H), 7.11 (app d, *J* = 7.5 Hz, 1H), 3.22 (s, 3H), 3.20–3.14 (m, 2H), 2.91–2.81 (m, 2H), 2.31 (s, 3H).
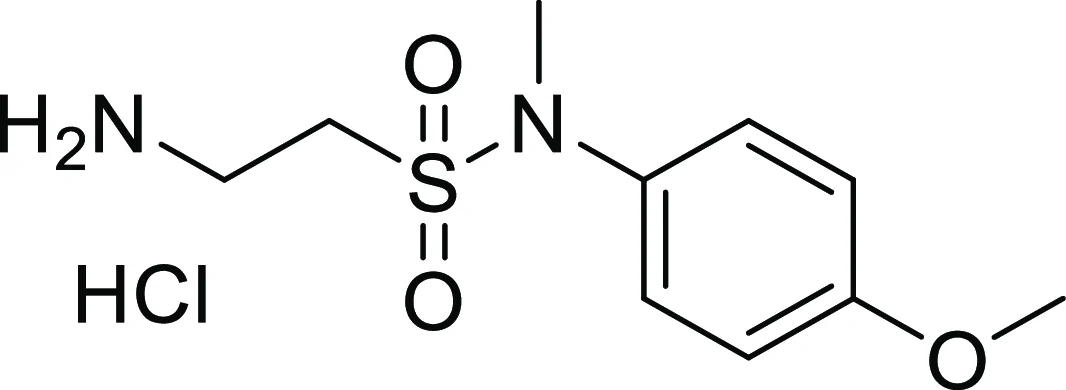


**2-Amino-*N*-(4-methoxyphenyl)-** ***N*** **-methylethane-1-sulfonamide hydrochloride (5j).**Prepared according to general procedure D from phthalimide **4j** (700 mg, 1.87 mmol, 1.0 equiv) and NH_2_NH_2_·H_2_O (0.363 mL, 7.49 mmol, 4.0 equiv) in EtOH (30 mL) to yield the title compound as a yellow solid (441 mg, 84%). ^**1**^ **H NMR** (DMSO-d_6_, 500 MHz): δ 7.37–7.30 (m, 2H), 6.98–6.91 (m, 2H), 3.76 (s, 3H), 3.20 (s, 3H), 3.20–3.15 (m, 2H), 2.94–2.86 (m, 2H).
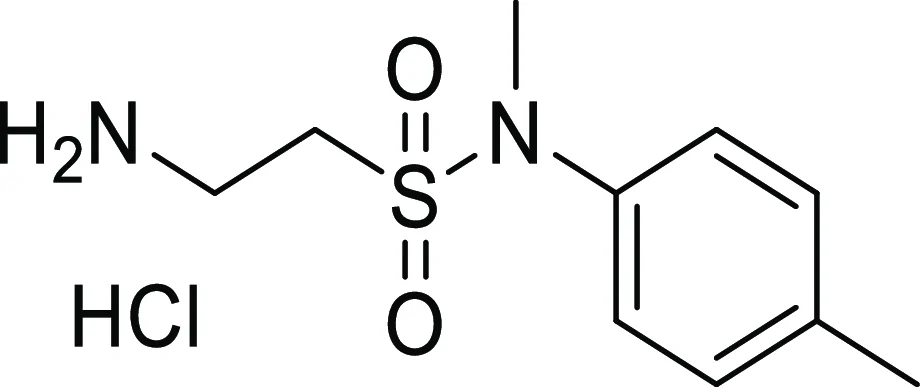


**2-Amino-** ***N*** **-methyl-*N*-(p-tolyl)­ethane-1-sulfonamide hydrochloride (5k).**Prepared according to general procedure D from phthalimide **4k** (773 mg, 2.16 mmol, 1 equiv) and NH_2_NH_2_·H_2_O (0.418 mL, 8.63 mmol, 4.0 equiv) in EtOH (30 mL) to yield the title compound as a gray solid (341 mg, 60%). ^**1**^ **H NMR** (DMSO-d_6_, 500 MHz): δ 7.31–7.27 (m, 2H), 7.22–7.18 (m, 2H), 3.98–3.91 (m, 2H), 3.51–3.39 (m, 2H), 3.23 (s, 3H), 2.29 (s, 3H).
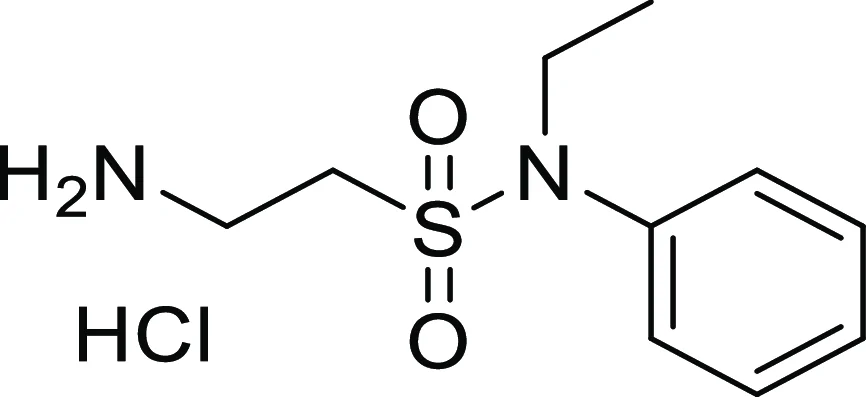


**2-Amino-*N*-ethyl-*N*-phenylethane-1-sulfonamide hydrochloride (5l).**Prepared according to general procedure D from phthalimide **4l** (1.94 g, 5.43 mmol, 1.0 equiv) and NH_2_NH_2_·H_2_O (0.315 mL, 6.51 mmol, 1.2 equiv) in EtOH (30 mL) to yield the title compound as white crystals (826 mg, 58%). ^**1**^ **H NMR** (DMSO-d_6_, 500 MHz): δ 7.49–7.34 (m, 5H), 3.71 (q, *J* = 7.1 Hz, 2H), 3.57–3.49 (m, 2H), 2.74 (t, *J* = 6.5 Hz, 2H), 1.00 (t, *J* = 7.1 Hz, 3H).
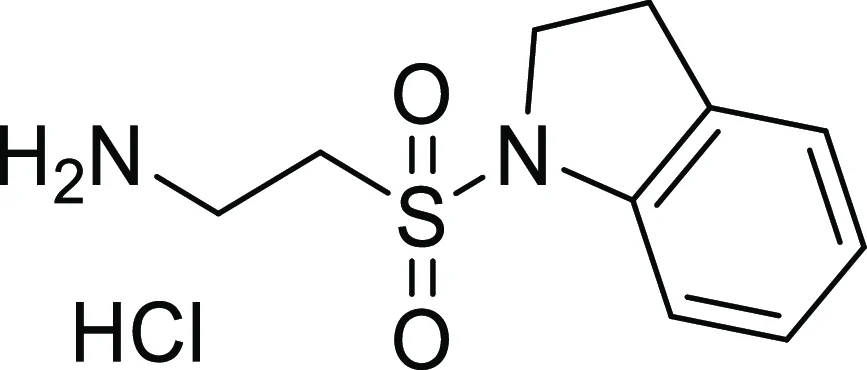


**2-(Indolin-1-ylsulfonyl)­ethan-1-amine hydrochloride (5m).**Prepared according to general procedure D from phthalimide **4m** (2.31 g, 6.49 mmol, 1.0 equiv) and NH_2_NH_2_·H_2_O (0.350 mL, 7.79 mmol, 1.2 equiv) in EtOH (30 mL) to yield the title compound as white crystals (1.40 g, 83%). ^**1**^ **H NMR** (CD_3_OD, 200 MHz): δ 7.34 (d, J = 7.6 Hz, 1H), 7.23 (d, J = 7.6 Hz, 1H), 7.16 (m, 1H), 7.02 (m, 1H), 4.03 (t, J = 8.5 Hz, 2H), 3.43–3.42 (m, 2H), 3.39–3.37 (m, 2H), 3.16 (t, J = 8.5 Hz, 2H).
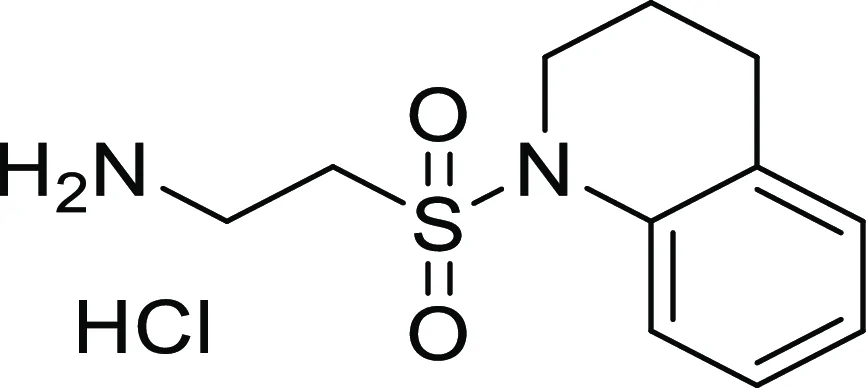


**2-((3,4-Dihydroquinolin-1­(2H)-yl)­sulfonyl)­ethan-1-amine hydrochloride (5n).**Prepared according to general procedure D from phthalimide **4n** (1.50 g, 4.05 mmol, 1.0 equiv) and NH_2_NH_2_·H_2_O (0.236 mL, 4.86 mmol, 1.2 equiv) in EtOH (30 mL) to yield the title compound as white crystals. The compound (1.22 g) contained 10% phthaloylhydrazide byproduct, amounting to a corrected yield of ∼90%. This solid was used without further purification. ^**1**^ **H NMR** (CD_3_OD, 200 MHz): δ 7.61 (app d, J = 6.8 Hz, 1H), 7.21–7.08 (m, 3H), 3.83 (t, J = 5.9 Hz, 2H), 3.49–3.45 (m, 2H), 3.38–3.31 (m, 2H), 2.89 (t, J = 6.6 Hz, 2H), 2.04 (t, J = 5.9, 2H).
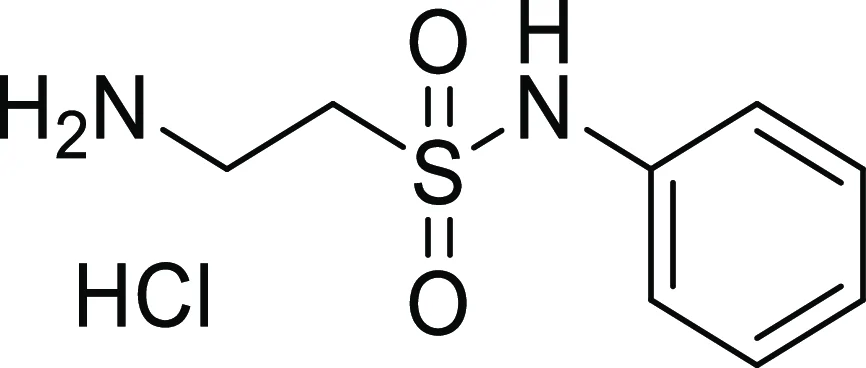


**2-Amino-*N*-phenylethane-1-sulfonamide hydrochloride (5o).**Prepared according to general procedure D from phthalimide **4o** (1.52 g, 6.78 mmol, 1.0 equiv) and NH_2_NH_2_·H_2_O (0.360 mL, 7.41 mmol, 1.1 equiv) in EtOH (30 mL) to yield the title compound as white crystals (764 mg, 64%). ^**1**^ **H NMR** (DMSO-d_6_, 600 MHz): δ 7.35–7.28 (m, 2H), 7.22–7.17 (m, 2H), 7.11–7.04 (m, 1H), 3.20–3.13 (m, 2H), 2.95–2.86 (m, 2H).
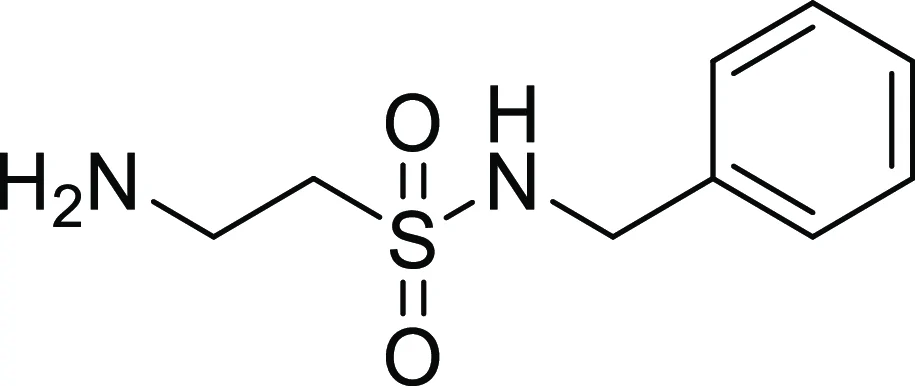


**2-Amino-*N*-benzylethane-1-sulfonamide (5p).**A solution of phthalimide**4p** (25.0 g, 72.6 mmol, 1.0 equiv) in EtOH (250 mL) was heated to reflux conditions after which NH_2_NH_2_·H_2_O (3.42 mL, 109 mmol, 1.5 equiv) was added. After stirring the reaction mixture for 4 h at reflux, the reaction mixture was cooled to RT, filtered using vacuum filtration and concentrated. H_2_O (150 mL) was added to the crude material and the pH was adjusted to ∼12 using 10% aq. NaOH. The aqueous phase was extracted thrice with CHCl_3_ (50 mL) and the combined organic phase was washed with brine (100 mL). After drying the organic layer over Na_2_SO_4_, it was concentrated and dried *in vacuo* to yield the title compound as a white solid in quantitative yield (15.6 g). ^**1**^ **H NMR** (DMSO-d_6_, 500 MHz): δ 7.35 (app d, J = 5.6 Hz, 4H), 7.31–7.24 (m, 1H), 4.14 (s, 2H), 3.03 (t, J = 6.7 Hz, 2H), 2.86 (t, J = 6.8 Hz, 2H).
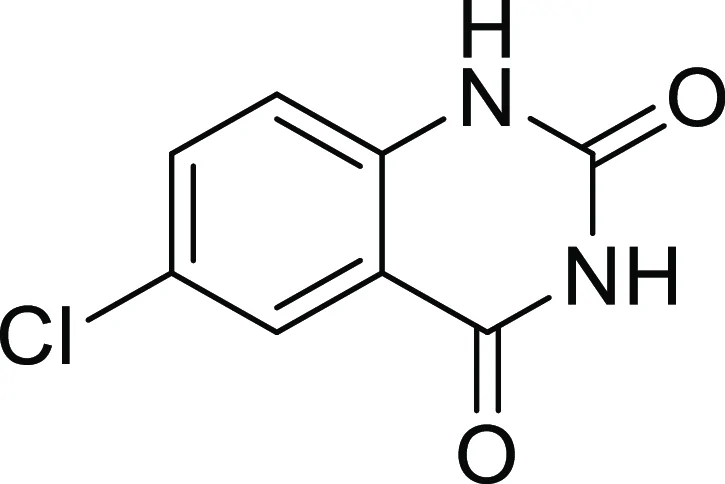


**6-Chloroquinazoline-2,4­(1H,3H)-dione (6a).**Prepared according to general procedure E from 2-amino-5-chloro-benzoic acid (4.25 g, 24.8 mmol, 1.0 equiv) and urea (15.0 g, 250 mmol, 10 equiv). The filter cake was washed with H_2_O and triturated with hot EtOH (100 mL) to yield the title compound as a white solid (2.81 g, 58%). ^**1**^ **H NMR** (DMSO-d_6_, 200 MHz): δ 11.37 (br, 2H), 7.80 (d, J = 2.5 Hz, 1H), 7.68 (dd, J = 8.7, 2.5 Hz, 1H), 7.18 (d, J = 8.7 Hz, 1H).
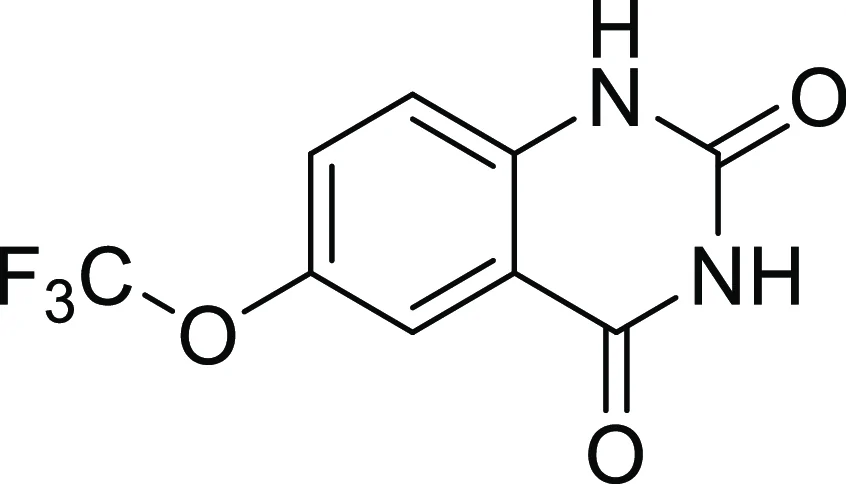


**6-(Trifluoromethoxy)­quinazoline-2,4­(1H,3H)-dione (6b).**Prepared according to general procedure E from 2-amino-5-(trifluoromethoxy)­benzoic acid (500 mg, 2.26 mmol, 1.0 equiv) and urea (543 mg, 9.04 mmol, 4.0 equiv) to yield the title compound as a white solid (321 mg, 58%). ^**1**^ **H NMR** (CD_3_OD, 500 MHz): δ 7.46 (d, J = 2.7 Hz, 1H), 7.14–7.06 (m, 1H), 6.77 (d, J = 8.9 Hz, 1H).
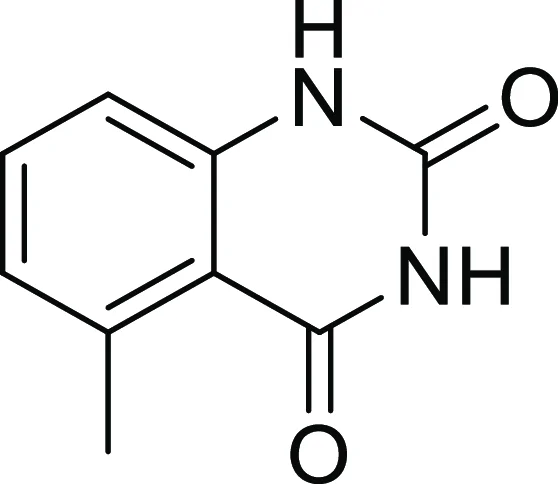


**5-Methylquinazoline-2,4-dione (6c).**A mixture of 2-amino-6-methylbenzoic acid (5.00 g, 33.1 mmol, 1.0 equiv) and urea (11.9 g, 198 mmol, 6.0 equiv) was heated to 150 °C. **CAUTION: urea will partially sublime and may clog the reflux condenser, leading to pressure buildup. This can be counteracted by periodically forcing the sublimed urea from the condenser back into the reaction flask with a spatula**. After stirring for 16 h at 150 °C, the reaction mixture was cooled to 100 °C and an equal volume of H_2_O was added. The suspension was cooled to RT and stirred for 30 min at RT. The solids were collected by vacuum filtration and triturated with glacial AcOH. The solid was washed with H_2_O and dried *in vacuo* to yield the title compound as a beige solid (3.41 g, 59%). ^**1**^ **H NMR** (CDCl_3_, 500 MHz): δ 11.04 (br, 1H), 10.99 (br, 1H), 7.44 (dd, J = 8.9, 7.8 Hz, 1H), 7.00 (d, J = 8.2 Hz, 1H), 6.93 (d, J = 7.4 Hz, 1H), 2.62 (s, 3H).
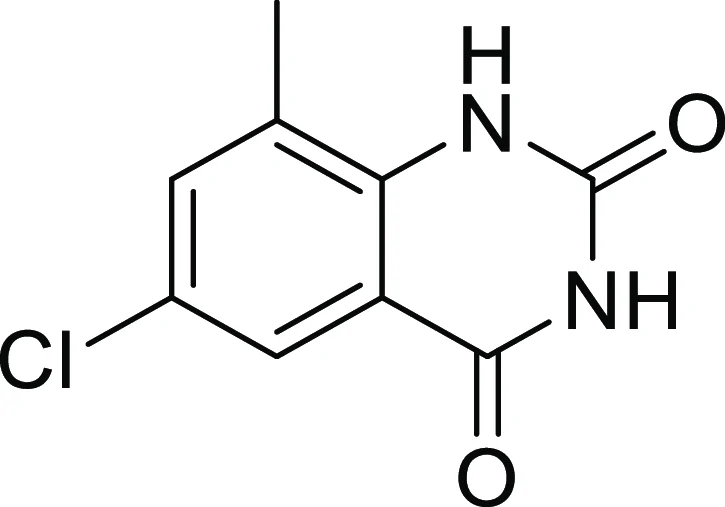


**6-Chloro-8-methylquinazoline-2,4­(1H,3H)-dione (6d).**Prepared according to general procedure E from 2-amino-3-methyl-5-chlorobenzoic acid (4.00 g, 21.6 mmol, 1.0 equiv) and urea (12.9 g, 216 mmol, 10 equiv). The filter cake was washed with H_2_O and triturated with hot EtOH (100 mL) to yield the title compound as a yellow solid (3.79 g, 83%). ^**1**^ **H NMR** (DMSO-d_6_, 200 MHz): δ 7.60 (s, 1H), 7.44 (s, 1H), 2.28 (s, 3H).
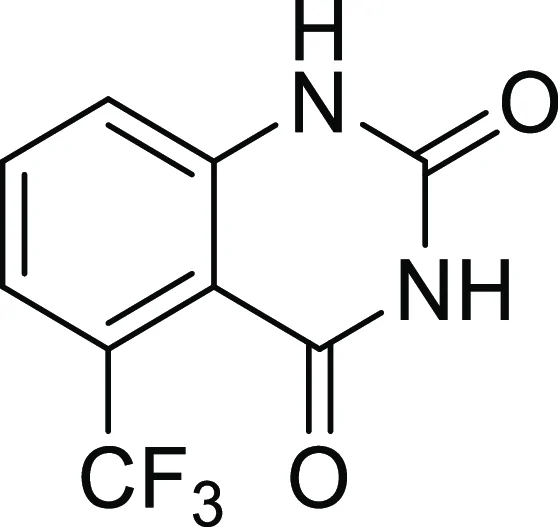


**5-(Trifluoromethyl)­quinazoline-2,4­(1H,3H)-dione (6e).**Prepared according to general procedure E from 2-amino-6-trifluoromethylbenzoic acid (1.00 g, 4.87 mmol, 1.0 equiv) and urea (2.92 g, 48.7 mmol, 10 equiv) to yield the title compound as a white solid (946 mg, 85%). ^**1**^ **H NMR** (DMSO-d_6_, 200 MHz): δ 11.43 (br, 2H), 7.78 (app t, J = 7.9 Hz, 1H), 7.58 (d, J = 7.4 Hz, 1H), 7.48 (d, J = 8.2 Hz, 1H).
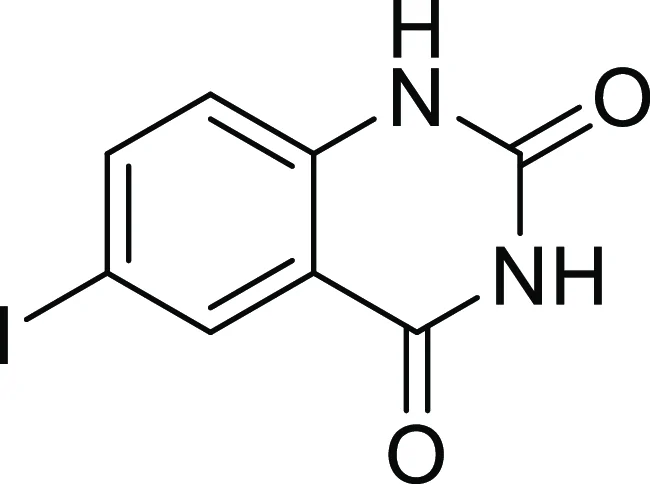


**6-Iodoquinazoline-2,4­(1H,3H)-dione (6f).**Prepared according to general procedure E from 2-amino-5-iodobenzoic acid (5.00 g, 19.0 mmol, 1.0 equiv) and urea (11.4 g, 190 mmol, 10 equiv) to yield the title compound as a yellow solid (5.34 g, 98%). ^**1**^ **H NMR** (DMSO-d_6_, 200 MHz): δ 8.04 (d, J = 2.0 Hz, 1H), 7.77 (dd, J = 8.6, 2.1 Hz, 1H), 6.90 (d, J = 8.6 Hz, 1H).
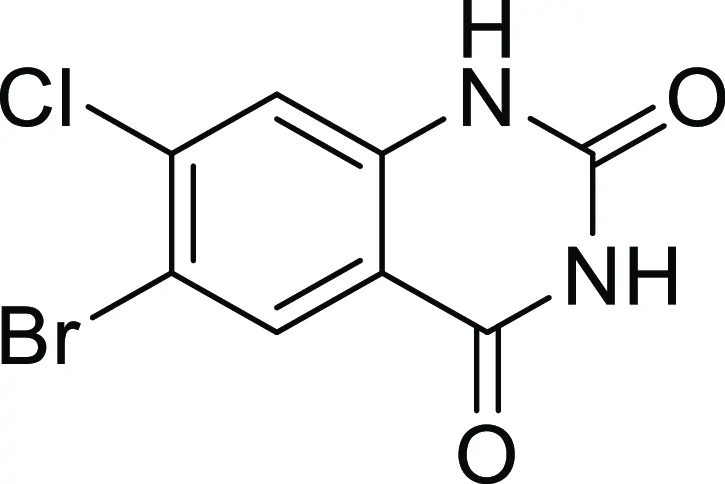


**6-Bromo-7-chloroquinazoline-2,4­(1H,3H)-dione (6g).**Prepared according to general procedure E from 2-amino-5-bromo-4-chlorobenzoic acid (987 mg, 3.94 mmol, 1.0 equiv) and urea (2.36 g, 39.4 mmol, 10 equiv) to yield the title compound as a white solid (1.00 g, 92%). ^**1**^ **H NMR** (DMSO- d_6_, 200 MHz): δ 8.01 (s, 1H), 7.36 (s, 1H).
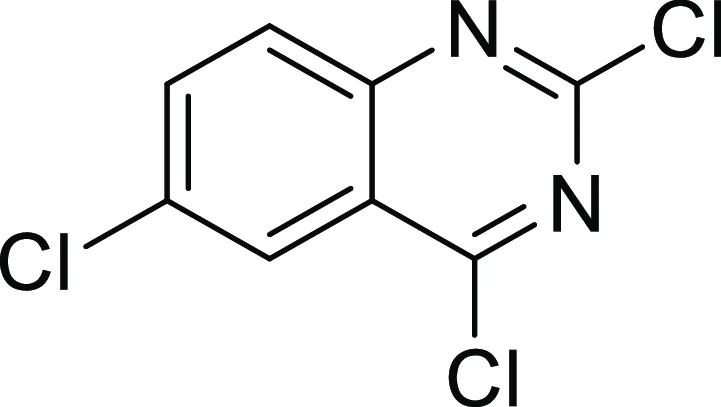


**2,4,6-Trichloroquinazoline (7a).**Prepared according to general procedure F from quinazoline-2,4­(1H,3H)-dione **6a** (1.41 g, 7.17 mmol, 1.0 equiv) and *N,N*-diethylaniline (2.30 mL, 14.0 mmol, 2.0 equiv) in POCl_3_ (5 mL) to yield the title compound as a white solid (1.52 g, 91%). ^**1**^ **H NMR** (CDCl_3_, 200 MHz) δ 8.23–8.22 (m, 1H), 7.97–7.87 (m, 2H).
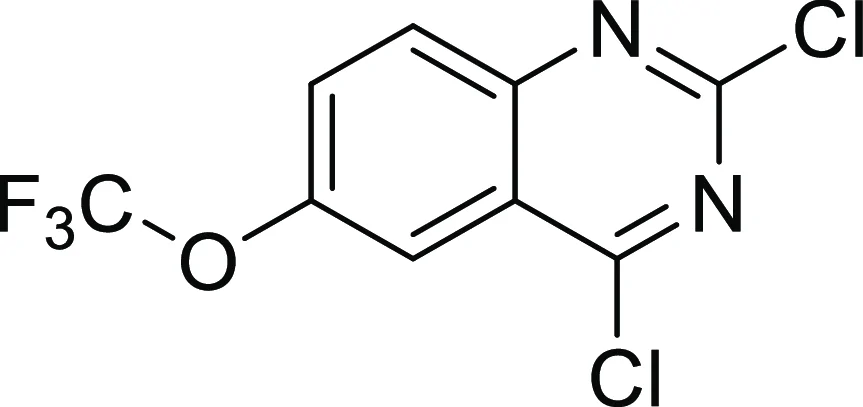


**2,4-Dichloro-6-(trifluoromethoxy)­quinazoline (7b).**Prepared according to general procedure F from quinazoline-2,4­(1H,3H)-dione **6b** (312 mg, 1.27 mmol, 1.0 equiv) and *N,N*-diethylaniline (0.363 mL, 2.28 mmol, 1.8 equiv) in POCl_3_ (4 mL) to yield the title compound as yellow crystals (50.0 mg, 13%). ^**1**^ **H NMR** (CD_3_OD, 500 MHz): δ 8.18 (dd, J = 2.6, 1.2 Hz, 1H), 8.12 (d, J = 9.1 Hz, 1H), 8.03 (dd, J = 9.2, 2.6 Hz, 1H).
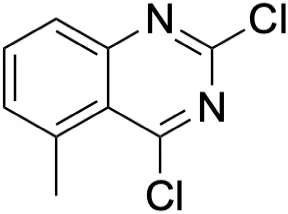


**2,4-Dichloro-5-methylquinazoline (7c).**Prepared according to general procedure F from quinazoline-2,4­(1H,3H)-dione **6c** (1.00 g, 5.68 mmol, 1.0 equiv) in POCl_3_ (20 mL). After stirring for 16 h at reflux, the reaction mixture was cooled to RT and carefully poured into ice H_2_O. After stirring for 30 min at RT, the solids were filtered off using vacuum filtration and the crude product was purified by NP column chromatography (0–15% EtOAc in *c*Hex) to yield the title compound as a white solid (831 mg, 69%). ^**1**^ **H NMR** (DMSO-d_6_, 500 MHz): δ 7.65 (dd, J = 8.6, 7.5 Hz, 1H), 7.40 (d, J = 8.0 Hz, 1H), 7.29 (d, J = 7.4 Hz, 1H), 2.74 (s, 3H).
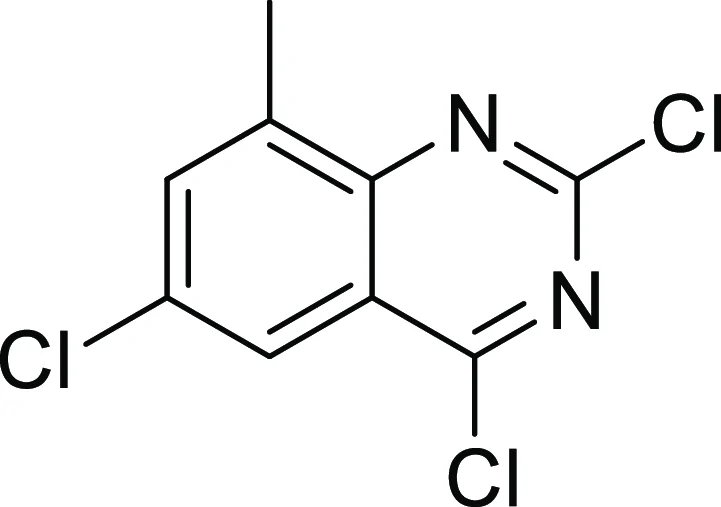


**2,4,6-Trichloro-8-methylquinazoline (7d).**Prepared according to general procedure F from quinazoline-2,4­(1H,3H)-dione **6d** (1.00 g, 4.76 mmol, 1.0 equiv) and *N,N-*diethylaniline (1.15 mL, 9.52 mmol, 2.0 equiv) in POCl_3_ (5 mL) to yield the title compound as a white solid (414 mg, 35%). ^**1**^ **H NMR** (CDCl_3_, 200 MHz): δ 8.00 (d, J = 1.8 Hz, 1H), 7.70 (dd, J = 2.2, 1.0 Hz, 1H), 2.66 (s, 3H).
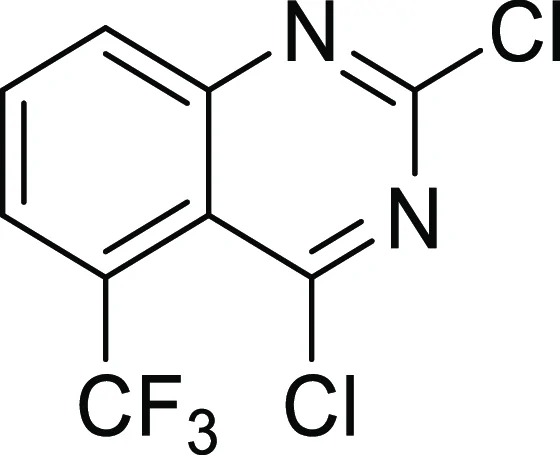


**2,4-Dichloro-5-trifluoromethylquinazoline (7e).**Prepared according to general procedure F from quinazoline-2,4­(1H,3H)-dione **6e** (814 mg, 3.45 mmol, 1.0 equiv) and DIPEA (1.26 mL, 7.25 mmol, 2.1 equiv) in POCl_3_ (4.0 mL) to yield the title compound as a white solid (948 mg, 85%). ^**1**^ **H NMR** (CDCl_3_, 200 MHz): δ 8.23–8.17 (m, 2H), 8.00 (app t, J = 7.9 Hz, 1H).
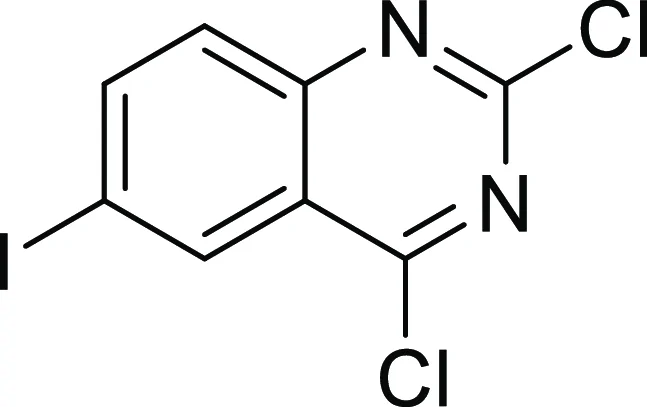


**2,4-Dichloro-6-iodoquinazoline (7f).**Prepared according to general procedure F from quinazoline-2,4­(1H,3H)-dione **6f** (2.00 g, 6.15 mmol, 1.0 equiv) and DIPEA (2.25 mL, 12.9 mmol, 2.1 equiv) in POCl_3_ (8 mL) to yield the title compound as a white solid (1.73 g, 87%). ^**1**^ **H NMR** (CDCl_3_, 200 MHz): δ 8.59 (d, J = 1.9 Hz, 1H), 8.19 (dd, J = 8.9, 1.9 Hz, 1H), 7.69 (d, J = 8.9 Hz, 1H).
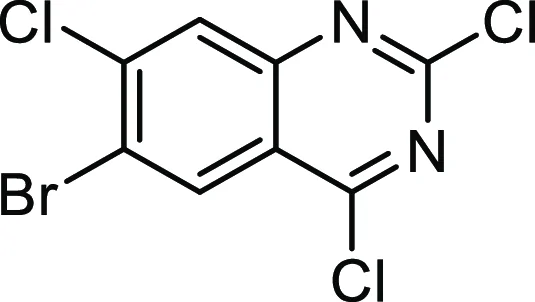


**6-Bromo-2,4,7-trichloroquinazoline (7g).**Prepared according to general procedure F from quinazoline-2,4­(1H,3H)-dione **6g** (950 mg, 3.45 mmol, 1.0 equiv) and DIPEA (1.26 mL, 7.25 mmol, 2.1 equiv) in POCl_3_ (4 mL) to yield the title compound as a white solid (431 mg, 40%). ^**1**^ **H NMR** (CDCl_3_, 200 MHz): δ 8.52 (s, 1H), 8.10 (s, 1H).
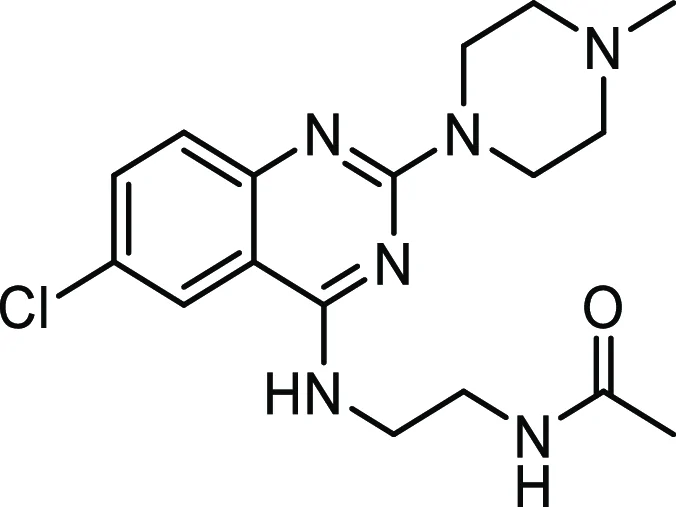


***N*-(2-((6-Chloro-2-(4-methylpiperazin-1-yl)­quinazolin-4-yl)­amino)­ethyl)­acetamide (8) (VUF10557).**Prepared according to general procedure G from dichloride **7a** (200 mg, 0.857 mmol, 1.0 equiv), *N*-(2-aminoethyl)­acetamide (87.5 mg, 0.857, 1.0 equiv), DIPEA (0.313 mL, 1.80 mmol, 2.1 equiv), *N*-methylpiperazine (0.952 mL, 8.57 mmol, 10 equiv) in EtOAc (3 mL) to yield the title compound as a white solid (115 mg, 37%). **ULCMS** (acidic mode) R_t_ 0.997 min, LC purity >95% (254 nm), *m*/*z* [M + H]^+^ calculated 363.16, found 363.15. ^**1**^ **H NMR** (DMSO-d_6_, 500 MHz): δ 8.14–8.05 (m, 2H), 7.97 (app t, J = 5.8 Hz, 1H), 7.49 (dd, J = 8.9, 2.4 Hz, 1H), 7.26 (d, J = 8.9 Hz, 1H), 3.83–3.70 (m, 4H), 3.49 (app q, J = 6.3 Hz, 2H), 3.31–3.27 (m, 2H), 2.42–2.30 (m, 4H), 2.22 (s, 3H), 1.80 (s, 3H). ^**13**^ **C NMR** (DMSO-d_6_, 126 MHz): δ 169.5, 159.2, 158.6, 150.5, 132.5, 127.0, 124.0, 122.1, 111.3, 54.6, 45.8, 43.3, 40.3, 37.6, 22.7. **HRMS** *m*/*z* [M + H]^+^ C_17_H_24_ClN_6_O^+^ calculated 363.1695, found 363.1701.
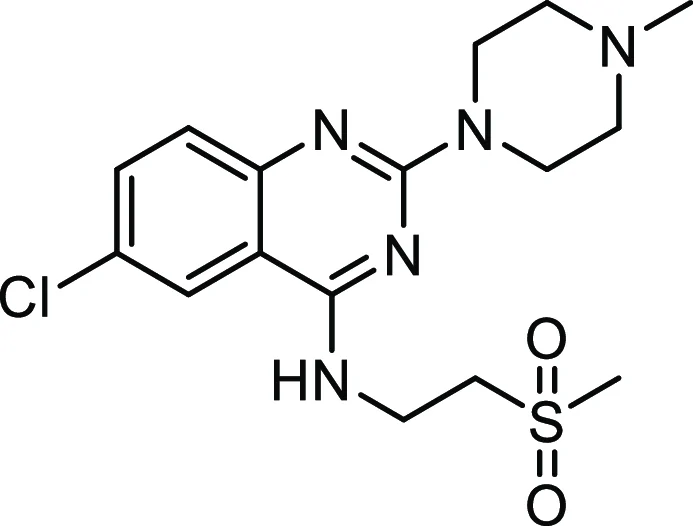


**6-Chloro-2-(4-methylpiperazin-1-yl)-*N*-(2-(methylsulfonyl)­ethyl)­quinazolin-4-amine (9) (VUF10646).**Prepared according to general procedure G from dichloride **7a** (200 mg, 0.857 mmol, 1.0 equiv), 2-(methylsulfonyl)­ethan-1-amine hydrochloride (151 mg, 0.946 mmol, 1.1 equiv), DIPEA (0.313 mL, 1.80 mmol, 2.1 equiv), and *N*-methylpiperazine (0.952 mL, 8.57 mmol, 10 equiv) in EtOAc (3 mL) to yield the title compound as a white solid (296 mg, 90%). **ULCMS** (acidic mode) R_t_ 1.878 min, LC purity >99% (254 nm), *m*/*z* [M + H]^+^ calculated 384.13, found 384.10. ^**1**^ **H NMR** (DMSO-d_6_, 600 MHz): δ 8.30 (app t, J = 5.5 Hz, 1H), 8.09–8.06 (m, 1H), 7.51 (dd, J = 8.9, 2.4 Hz, 1H), 7.28 (d, J = 8.9 Hz, 1H), 3.87 (app q, J = 6.4 Hz, 2H), 3.80–3.74 (m, 4H), 3.47 (app t, J = 6.6 Hz, 2H), 3.03 (s, 3H), 2.35 (app t, J = 5.0 Hz, 4H), 2.21 (s, 3H). ^**13**^ **C NMR** (DMSO-d_6_, 151 MHz): δ 158.9, 158.5, 150.5, 132.8, 127.1, 124.2, 122.0, 111.2, 54.6, 52.1, 45.9, 43.3, 40.9, 34.6. **HRMS** *m*/*z* [M + H]^+^ C_16_H_23_ClN_5_O_2_S^+^ calculated 384.1256, found 384.1255.
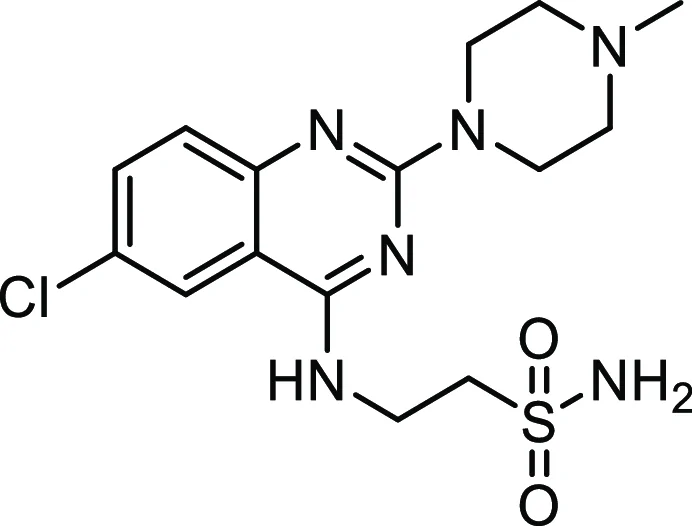


**2-((6-Chloro-2-(4-methylpiperazin-1-yl)­quinazolin-4-yl)­amino)­ethane-1-sulfonamide (10) (VUF10558).**Prepared according to general procedure G from dichloride **7a** (200 mg, 0.857 mmol, 1.0 equiv), amine **5a** (130 mg, 0.857 mmol, 1.0 equiv), DIPEA (0.313 mL, 1.80 mmol, 2.1 equiv), and *N*-methylpiperazine (0.952 mL, 8.57 mmol, 10 equiv) in EtOAc (3 mL) to yield the title compound as a white solid (125 mg, 38%). **ULCMS** (acidic mode) R_t_ 0.935 min, LC purity >99% (254 nm), *m*/*z* [M + H]^+^ calculated 385.88, found 385.00. ^**1**^ **H NMR** (DMSO-d_6_, 600 MHz): δ 8.19 (app t, J = 5.6 Hz, 1H), 8.04 (d, J = 2.4 Hz, 1H), 7.50 (dd, J = 8.9, 2.4 Hz, 1H), 7.27 (d, J = 8.9 Hz, 1H), 6.95 (s, 2H), 3.86–3.81 (m, 2H), 3.80–3.74 (m, 4H), 3.36–3.33 (m, 3H), 2.34 (app t, J = 5.0 Hz, 4H), 2.21 (s, 3H). ^**13**^ **C NMR** (DMSO-d_6_, 151 MHz): δ 158.8, 158.5, 150.5, 132.6, 127.0, 124.1, 122.0, 111.2, 54.6, 52.7, 45.9, 43.4, 35.9. **HRMS** *m*/*z* [M + H]^+^ C_15_H_22_ClN_6_O_2_S^+^ calculated 385.1208, found 385.1211.
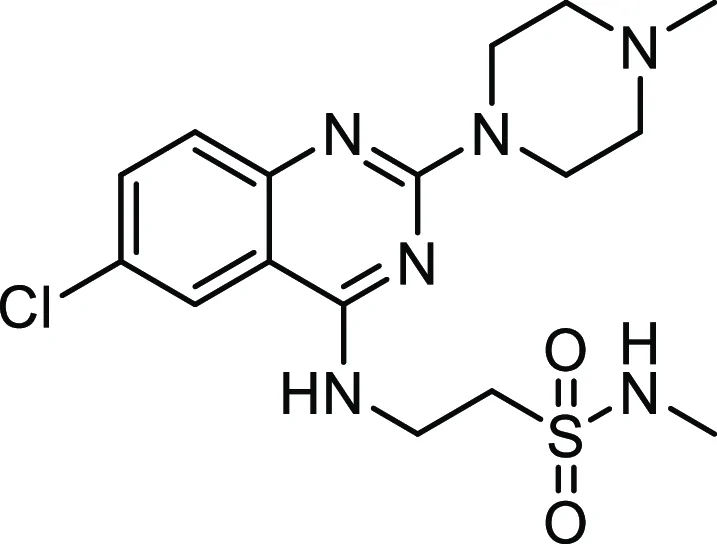


**2-((6-Chloro-2-(4-methylpiperazin-1-yl)­quinazolin-4-yl)­amino)-** ***N*** **-methylethane-1-sulfonamide (11) (VUF10570).**Prepared according to general procedure G from dichloride **7a** (200 mg, 0.857 mmol, 1.0 equiv), amine **5b** (131 mg, 0.857 mmol, 1.0 equiv), DIPEA (0.313 mL, 1.80 mmol, 2.1 equiv), and *N*-methylpiperazine (0.952 mL, 8.57 mmol, 10 equiv) in EtOAc (3 mL) to yield the title compound as a white solid (117 mg, 34%). **ULCMS** (acidic mode) R_t_ 1.043 min, LC purity >96% (254 nm), *m*/*z* [M + H]^+^ calculated 399.91, found 399.10. ^**1**^ **H NMR** (CD_3_OD, 500 MHz): δ 7.85 (d, J = 2.3 Hz, 1H), 7.49 (dd, J = 8.9, 2.4 Hz, 1H), 7.36 (d, J = 9.0 Hz, 1H), 4.00–3.87 (m, 6H), 3.47–3.39 (m, 2H), 2.72 (s, 3H), 2.56 (app t, J = 5.1 Hz, 4H), 2.37 (s, 3H). ^**13**^ **C NMR** (CD_3_OD, 126 MHz): δ 160.8, 160.2, 151.6, 134.2, 127.6, 127.4, 122.7, 112.7, 55.9, 49.6, 46.1, 44.6, 37.0, 29.2. **HRMS** *m*/*z* [M + H]^+^ C_16_H_24_ClN_6_O_2_S^+^ calculated 399.1364, found 399.1364.
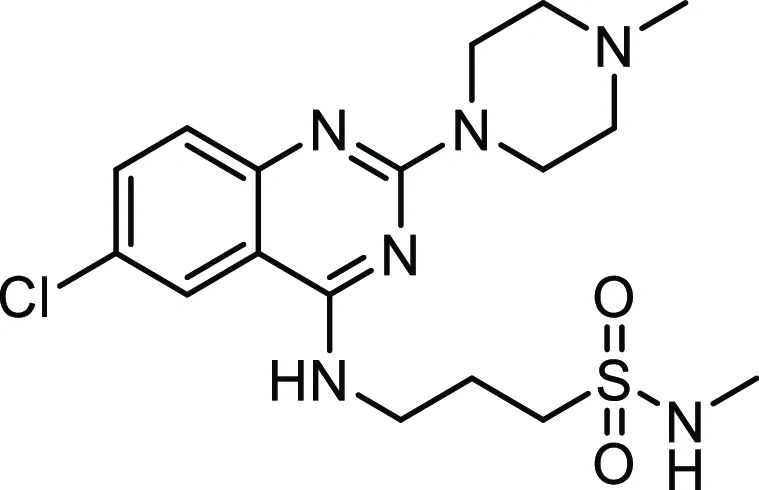


**3-((6-Chloro-2-(4-methylpiperazin-1-yl)­quinazolin-4-yl)­amino)-** ***N*** **-methylpropane-1-sulfonamide (12) (VUF10517).**Prepared according to general procedure G from dichloride **7a** (200 mg, 0.857 mmol, 1.0 equiv), amine **5c** (162 mg, 0.859 mmol, 1.0 equiv), and DIPEA (0.460 mL, 2.64 mmol, 3.1 equiv) in THF (3 mL). The intermediate was isolated by diluting the reaction mixture with EtOAc (50 mL), and washing the mixture with H2O (50 mL) and brine (50 mL). The organic phase was dried over Na_2_SO_4_ and concentrated, to yield a crude product that was purified using NP column chromatography (EtOAc in *c*Hex). To the pure intermediate in THF (3 mL) was added *N*-methylpiperazine (1.00 mL, 9.01 mmol, 11 equiv) and procedure G was followed, to yield the title compound as a white solid (104 mg, 30%). **ULCMS** (acidic mode) R_t_ 1.069 min, LC purity >99% (254 nm), *m*/*z* [M + H]^+^ calculated 413.14, found 413.10. ^**1**^ **H NMR** (DMSO-d_6_, 600 MHz): δ 8.14–8.06 (m, 2H), 7.49 (dd, J = 8.9, 2.4 Hz, 1H), 7.26 (d, J = 8.9 Hz, 1H), 6.89 (q, J = 5.0 Hz, 1H), 3.77 (m, 4H), 3.57 (app q, J = 6.7 Hz, 2H), 3.13–3.05 (m, 2H), 2.55 (d, J = 5.0 Hz, 3H), 2.35 (app t, J = 5.0 Hz, 4H), 2.21 (s, 3H), 2.05–1.95 (m, 2H). ^**13**^ **C NMR** (DMSO-d_6_, 151 MHz): δ 159.1, 158.6, 150.5, 132.5, 127.0, 124.0, 122.1, 111.2, 54.6, 47.4, 45.9, 43.3, 39.1, 28.6, 22.9. **HRMS** *m*/*z* [M + H]^+^ C_17_H_26_ClN_6_O_2_S^+^ calculated 413.1521, found 413.1526.
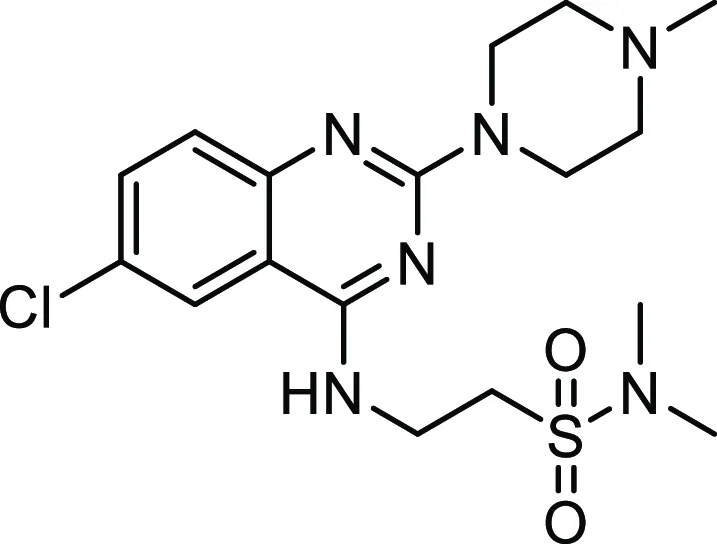


**2-((6-Chloro-2-(4-methylpiperazin-1-yl)­quinazolin-4-yl)­amino)-*N*,*N*-dimethylethane-1-sulfonamide (13) (VUF10571).**Prepared according to general procedure G from dichloride **7a** (200 mg, 0.857 mmol, 1.0 equiv), amine **5d** (162 mg, 0.859 mmol, 1.0 equiv), DIPEA (0.320 mL, 1.84 mmol, 2.1 equiv), and *N*-methylpiperazine (1.00 mL, 9.01 mmol, 11 equiv) in EtOAc (3 mL) to yield the title compound as a white solid (117 mg, 33%). **ULCMS** (acidic mode) R_t_ 1.104 min, LC purity >97% (254 nm), *m*/*z* [M + H]^+^ calculated 413.94, found 413.10. ^**1**^ **H NMR** (DMSO-d_6_, 500 MHz): δ 8.30 (app t, J = 5.6 Hz, 1H), 8.05 (d, J = 2.4 Hz, 1H), 7.51 (dd, J = 8.9, 2.3 Hz, 1H), 7.28 (d, J = 8.9 Hz, 1H), 3.87–3.73 (m, 6H), 3.41–3.35 (m, 2H), 2.77 (s, 6H), 2.34 (app t, J = 5.0 Hz, 4H), 2.20 (s, 3H). ^**13**^ **C NMR** (DMSO-d_6_, 126 MHz): δ 158.9, 158.4, 150.6, 132.7, 127.1, 124.2, 121.9, 111.2, 54.6, 45.9, 44.4, 43.3, 37.0, 35.1. **HRMS** *m*/*z* [M + H]^+^ C_17_H_26_ClN_6_O_2_S^+^ calculated 413.1521, found 413.1523.
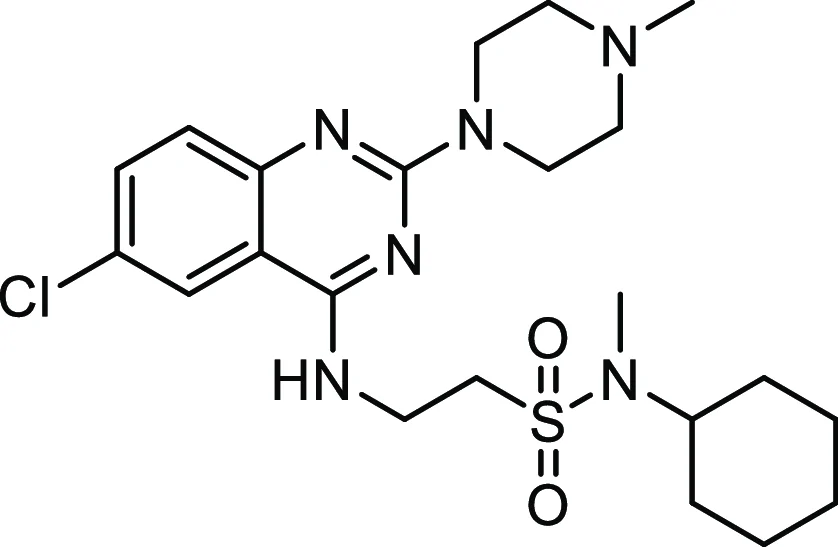


**2-((6-Chloro-2-(4-methylpiperazin-1-yl)­quinazolin-4-yl)­amino)-*N*-cyclohexyl-** ***N*** **-methylethane-1-sulfonamide (14) (VUF11302).**Prepared according to general procedure G from dichloride **7a** (200 mg, 0.857 mmol, 1.0 equiv), amine **5e** (217 mg, 0.857 mmol, 1.0 equiv), DIPEA (0.320 mL, 1.84 mmol, 2.1 equiv), and *N*-methylpiperazine (0.952 mL, 8.57 mmol, 10 equiv) in EtOAc (3 mL) to yield the title compound as a white solid (309 mg, 75%). **ULCMS** (acidic mode) R_t_ 1.347 min, LC purity >98% (254 nm), *m*/*z* [M + H]^+^ calculated 481.21, found 481.15. ^**1**^ **H NMR** (CDCl_3_, 600 MHz): δ 7.49 (d, J = 2.3 Hz, 1H), 7.43 (dd, J = 8.9, 2.3 Hz, 1H), 7.37 (d, J = 8.9 Hz, 1H), 6.36 (app t, J = 5.7 Hz, 1H), 4.07 (app q, J = 5.8 Hz, 2H), 3.97–3.85 (m, 4H), 3.68 (app tt, J = 12.0, 3.8 Hz, 1H), 3.26 (app t, J = 6.0 Hz, 2H), 2.80 (s, 3H), 2.48 (app t, J = 5.1 Hz, 4H), 2.34 (s, 3H), 1.85–1.78 (m, 2H), 1.77–1.71 (m, 2H), 1.67–1.59 (m, 1H), 1.47 (app qd, J = 12.4, 3.5 Hz, 2H), 1.34 (app qt, J = 13.2, 3.5 Hz, 2H), 1.05 (app qt, J = 13.1, 3.7 Hz, 1H). ^**13**^ **C NMR** (CDCl_3_, 151 MHz): δ 158.9, 158.8, 151.0, 133.4, 127.6, 126.2, 120.5, 111.1, 56.9, 55.3, 50.5, 46.4, 43.9, 35.9, 31.1, 28.7, 25.9, 25.3. **HRMS** *m*/*z* [M + H]^+^ C_22_H_29_N_6_O_2_S^+^ calculated 481.2147, found 481.2144.
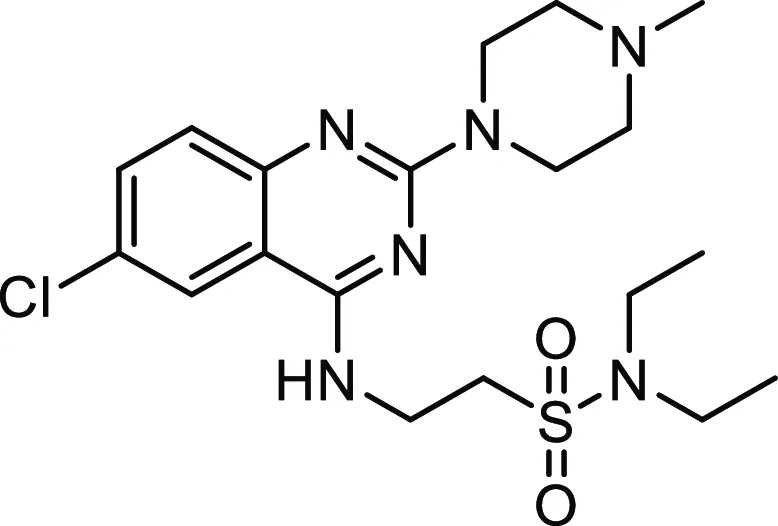


**2-((6-Chloro-2-(4-methylpiperazin-1-yl)­quinazolin-4-yl)­amino)-*N*,*N*-diethylethane-1-sulfonamide (15) (VUF10514).**Prepared according to general procedure G from dichloride **7a** (200 mg, 0.857 mmol, 1.0 equiv), amine **5f** (240 mg, 1.11 mmol, 1.3 equiv), DIPEA (0.320 mL, 1.84 mmol, 2.1 equiv), and *N*-methylpiperazine (0.952 mL, 8.57 mmol, 10 equiv) in EtOAc (3 mL) to yield the title compound as a white solid (154 mg, 44%). **ULCMS** (acidic mode) R_t_ 1.221 min, LC purity >99% (254 nm), *m*/*z* [M + H]^+^ calculated 441.18, found 441.15. ^**1**^ **H NMR** (DMSO-d_6_, 600 MHz): δ 8.26 (app t, J = 5.5 Hz, 1H), 8.04 (d, J = 2.4 Hz, 1H), 7.51 (dd, J = 8.9, 2.4 Hz, 1H), 7.28 (d, J = 8.9 Hz, 1H), 3.84–3.71 (m, 6H), 3.41–3.36 (m, 2H), 3.20 (q, J = 7.1 Hz, 4H), 2.34 (app t, J = 5.0 Hz, 4H), 2.20 (s, 3H), 1.10 (t, J = 7.1 Hz, 6H). ^**13**^ **C NMR** (DMSO-d_6_, 151 MHz): δ 158.9, 158.4, 150.5, 132.7, 127.1, 124.2, 122.0, 111.2, 54.6, 48.9, 45.9, 43.3, 41.2, 35.4, 14.4. **HRMS** *m*/*z* [M + H]^+^ C_19_H_30_ClN_6_O_2_S^+^ calculated 441.1834, found 441.1842.
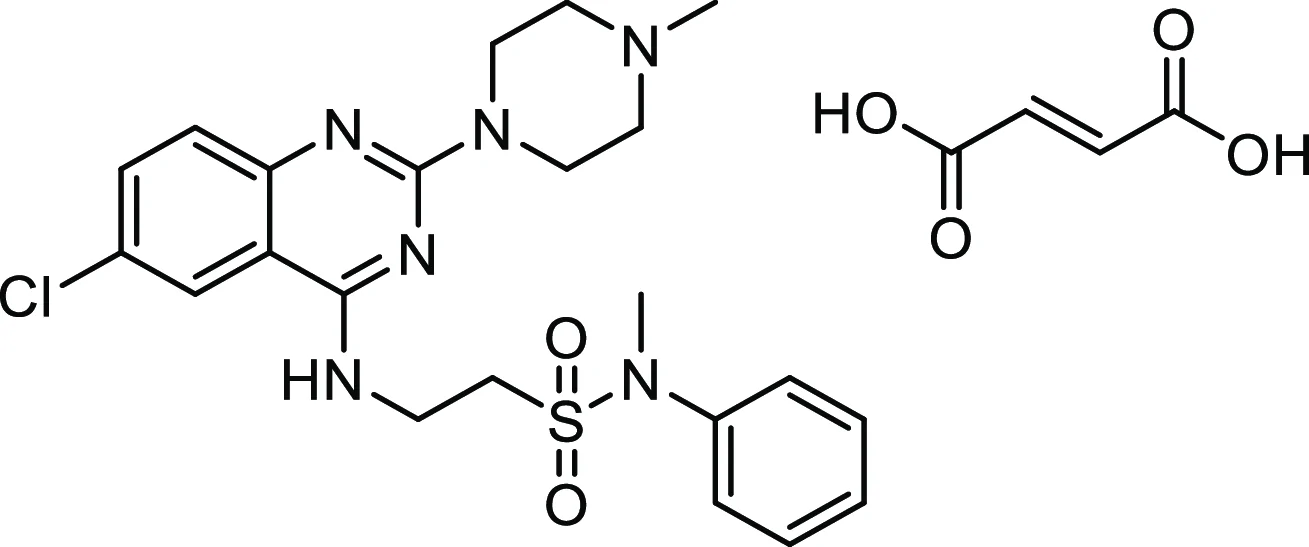


**2-((6-Chloro-2-(4-methylpiperazin-1-yl)­quinazolin-4-yl)­amino)-** ***N*** **-methyl-*N*-phenylethane-1-sulfonamide fumarate (16) (VUF10512).**Prepared according to general procedure G from dichloride **7a** (200 mg, 0.857 mmol, 1.0 equiv), amine **5g** (215 mg, 0.857 mmol, 1.0 equiv), DIPEA (0.320 mL, 1.84 mmol, 2.1 equiv), and *N*-methylpiperazine (0.952 mL, 8.57 mmol, 10 equiv) in EtOAc (3 mL). The product was converted to its corresponding fumaric acid salt using fumaric acid (99.4 mg, 0.857 mmol, 1.0 equiv) to yield the title compound as a white solid (231 mg, 51%). **ULCMS** (acidic mode) R_t_ 1.363 min, LC purity >98% (254 nm), *m*/*z* [M + H]^+^ calculated 475.16, found 475.15. ^**1**^ **H NMR** (DMSO-d_6_, 600 MHz): δ 8.31 (app t, J = 5.5 Hz, 1H), 8.02 (d, J = 2.4 Hz, 1H), 7.51 (dd, J = 8.9, 2.4 Hz, 1H), 7.43–7.35 (m, 4H), 7.33–7.25 (m, 2H), 6.59 (s, 2H), 3.82–3.77 (m, 2H), 3.74–3.66 (m, 4H), 3.52–3.46 (m, 2H), 3.27 (s, 3H), 2.34 (app t, J = 5.0 Hz, 4H), 2.24 (s, 3H). ^**13**^ **C NMR** (DMSO-d_6_, 151 MHz): δ 166.5, 158.8, 158.3, 150.5, 141.3, 134.3, 132.8, 129.1, 127.08, 127.05, 126.2, 124.2, 121.9, 111.1, 54.3, 46.1, 45.5, 43.0, 37.8, 35.1. **HRMS** *m*/*z* [M + H]^+^ C_22_H_28_ClN_6_O_2_S^+^ calculated 475.1677, found 475.1691.
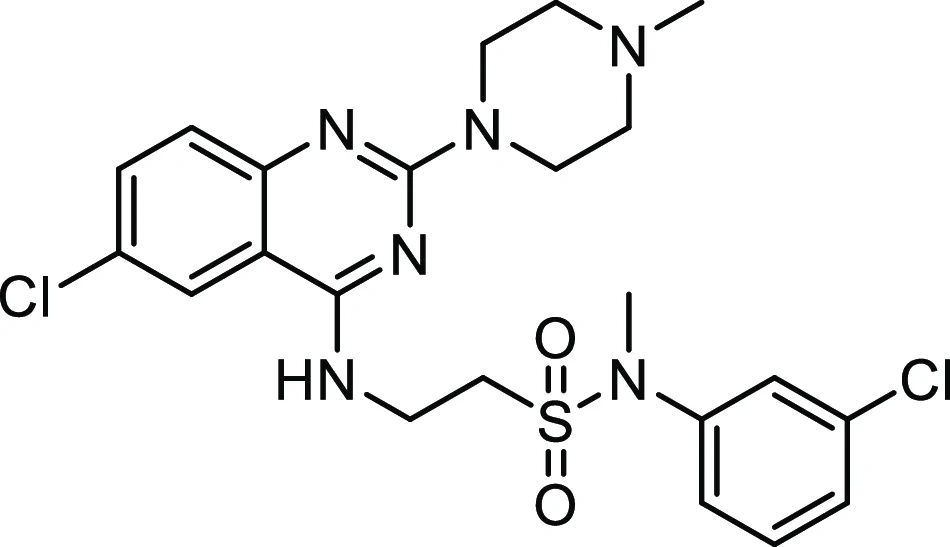


**2-((6-Chloro-2-(4-methylpiperazin-1-yl)­quinazolin-4-yl)­amino)-*N*-(3-chlorophenyl)-** ***N*** **-methylethane-1-sulfonamide (17) (VUF11293).**Prepared according to general procedure G from dichloride **7a** (200 mg, 0.857 mmol, 1.0 equiv), amine **5h** (255 mg, 0.894 mmol, 1.0 equiv), DIPEA (0.320 mL, 1.84 mmol, 2.1 equiv), and *N*-methylpiperazine (0.952 mL, 8.57 mmol, 10 equiv) in EtOAc (3 mL) to yield the title compound as a white solid (382 mg, 87%). **ULCMS** (acidic mode) R_t_ 1.351 min, LC purity >98% (254 nm), *m*/*z* [M + H]^+^ calculated 509.12, found 509.15. ^**1**^ **H NMR** (CDCl_3_, 600 MHz): δ 7.44 (dd, J = 8.9, 2.3 Hz, 1H), 7.40 (d, J = 2.2 Hz, 1H), 7.38 (d, J = 8.9 Hz, 1H), 7.36–7.34 (m, 1H), 7.30–7.23 (m, 4H), 4.07 (app q, J = 5.9 Hz, 2H), 3.92–3.81 (m, 4H), 3.40 (app t, J = 6.1 Hz, 2H), 3.33 (s, 3H), 2.46 (app t, J = 5.1 Hz, 4H), 2.35 (s, 3H). ^**13**^ **C NMR** (CDCl_3_, 151 MHz): δ 158.8, 158.7, 151.0, 142.2, 135.1, 133.5, 130.5, 127.8, 127.7, 126.3, 126.2, 124.4, 120.4, 110.9, 55.2, 48.3, 46.4, 43.9, 38.4, 35.7. **HRMS** *m*/*z* [M + H]^+^ C_22_H_27_Cl_2_N_6_O_2_S^+^ calculated 509.1288, found 509.1291.
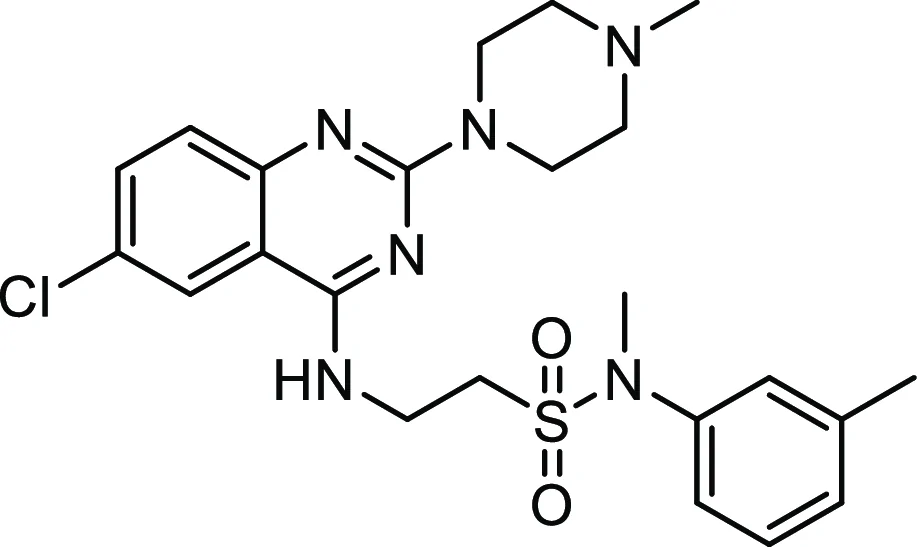


**2-((6-Chloro-2-(4-methylpiperazin-1-yl)­quinazolin-4-yl)­amino)-** ***N*** **-methyl-*N*-(m-tolyl)­ethane-1-sulfonamide (18) (VUF11300).**Prepared according to general procedure G from dichloride **7a** (200 mg, 0.857 mmol, 1.0 equiv), amine **5i** (280 mg, 1.06 mmol, 1.2 equiv), DIPEA (0.320 mL, 1.84 mmol, 2.1 equiv), and *N-*methylpiperazine (0.952 mL, 8.57 mmol, 10 equiv) in EtOAc (3 mL) to yield the title compound as a white solid (413 mg, 99%). **ULCMS** (acidic mode) R_t_ 1.333 min, LC purity >96% (254 nm), *m*/*z* [M + H]^+^ calculated 489.18, found 489.15. ^**1**^ **H NMR** (CDCl_3_, 600 MHz): δ 7.43 (dd, J = 8.9, 2.3 Hz, 1H), 7.40 (d, J = 2.3 Hz, 1H), 7.37 (d, J = 8.9 Hz, 1H), 7.23 (app t, J = 7.7 Hz, 1H), 7.17–7.12 (m, 2H), 7.09–7.05 (m, 1H), 6.18 (app t, J = 5.8 Hz, 1H), 4.06 (app q, J = 5.9 Hz, 2H), 3.94–3.82 (m, 4H), 3.40 (app t, J = 6.1 Hz, 2H), 3.33 (s, 3H), 2.46 (app t, J = 5.1 Hz, 4H), 2.35 (s, 3H), 2.32 (s, 3H). ^**13**^ **C NMR** (CDCl_3_, 151 MHz): δ 158.8, 158.7, 151.0, 140.9, 139.7, 133.5, 129.3, 128.6, 127.6, 127.3, 126.2, 123.4, 120.5, 111.0, 55.2, 48.1, 46.3, 43.9, 38.6, 35.7, 21.5. **HRMS** *m*/*z* [M + H]^+^ C_23_H_30_ClN_6_O_2_S^+^ calculated 489.1834, found 489.1828.
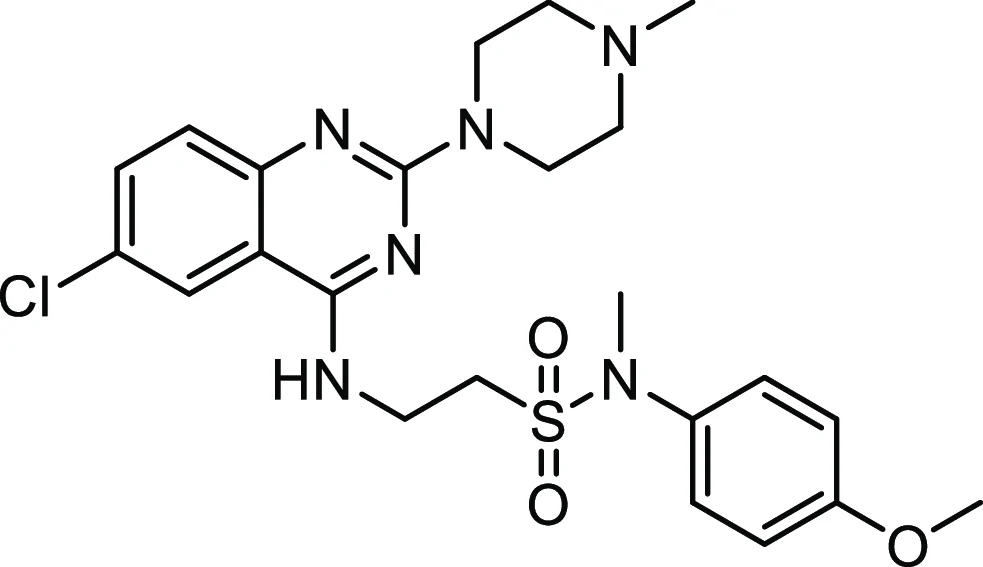


**2-((6-Chloro-2-(4-methylpiperazin-1-yl)­quinazolin-4-yl)­amino)-*N*-(4-methoxyphenyl)-** ***N*** **-methylethane-1-sulfonamide (19) (VUF11298).**Prepared according to general procedure G from dichloride **7a** (200 mg, 0.857 mmol, 1.0 equiv), amine **5j** (353 mg, 1.26 mmol, 1.5 equiv), DIPEA (0.320 mL, 1.84 mmol, 2.1 equiv), and *N*-methylpiperazine (0.952 mL, 8.57 mmol, 10 equiv) in EtOAc (3 mL) to yield the title compound as a white solid (189 mg, 30%). **ULCMS** (acidic mode) R_t_ 1.395 min, LC purity >98% (254 nm), *m*/*z* [M + H]^+^ calculated 505.17, found 505.20. ^**1**^ **H NMR** (CDCl_3_, 600 MHz): δ 7.43 (dd, J = 8.9, 2.3 Hz, 1H), 7.40 (d, J = 2.2 Hz, 1H), 7.37 (d, J = 8.9 Hz, 1H), 7.28–7.24 (m, 2H), 6.88–6.83 (m, 2H), 4.06 (app q, J = 5.9 Hz, 2H), 3.93–3.86 (m, 4H), 3.78 (s, 3H), 3.37 (app t, J = 6.0 Hz, 2H), 3.31 (s, 3H), 2.48 (app t, J = 5.1 Hz, 4H), 2.35 (s, 3H). ^**13**^ **C NMR** (CDCl_3_, 151 MHz): δ 159.1, 158.8, 158.7, 150.9, 133.50, 133.48, 128.3, 127.6, 126.2, 120.5, 114.8, 111.0, 55.6, 55.2, 48.0, 46.3, 43.9, 39.0, 35.7. **HRMS** *m*/*z* [M + H]^+^ C_23_H_30_ClN_6_O_3_S^+^ calculated 505.1783, found 505.1785.
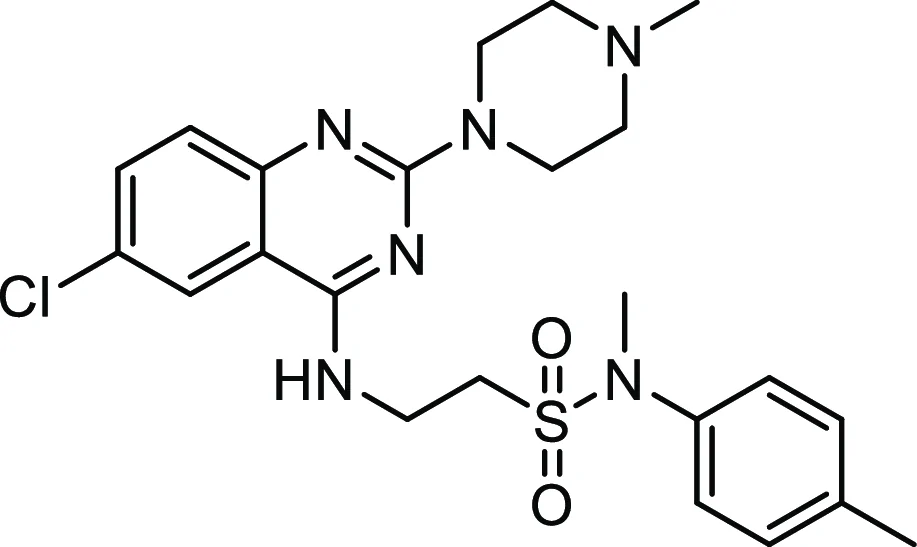


**2-((6-Chloro-2-(4-methylpiperazin-1-yl)­quinazolin-4-yl)­amino)-** ***N*** **-methyl-*N*-(p-tolyl)­ethane-1-sulfonamide (20) (VUF11299).**Prepared according to general procedure G from dichloride **7a** (200 mg, 0.857 mmol, 1.0 equiv), amine **5k** (281 mg, 1.06 mmol, 1.2 equiv), DIPEA (0.320 mL, 1.84 mmol, 2.1 equiv), and *N*-methylpiperazine (0.952 mL, 8.57 mmol, 10 equiv) in EtOAc (3 mL) to yield the title compound as a white solid (137 mg, 33%). **ULCMS** (acidic mode) R_t_ 1.343 min, LC purity >96% (254 nm), *m*/*z* [M + H]^+^ calculated 489.18, found 489.15. ^**1**^ **H NMR** (CDCl_3_, 600 MHz): δ 7.43 (dd, J = 8.9, 2.3 Hz, 1H), 7.39–7.35 (m, 2H), 7.25–7.21 (m, 2H), 7.17–7.12 (m, 2H), 7.16–7.13 (m, 2H), 4.06 (app q, J = 5.9 Hz, 2H), 3.93–3.81 (m, 4H), 3.38 (app t, J = 5.9 Hz, 2H), 3.32 (s, 3H), 2.46 (app t, J = 5.1 Hz, 4H), 2.34 (s, 3H), 2.32 (s, 3H). ^**13**^ **C NMR** (CDCl_3_, 151 MHz): δ 158.79, 158.76, 151.0, 138.3, 137.9, 133.5, 130.2, 127.6, 126.5, 126.1, 120.5, 111.0, 55.2, 48.0, 46.4, 43.9, 38.7, 35.7, 21.1. **HRMS** *m*/*z* [M + H]^+^ C_23_H_30_ClN_6_O_2_S^+^ calculated 489.1834, found 489.1828.
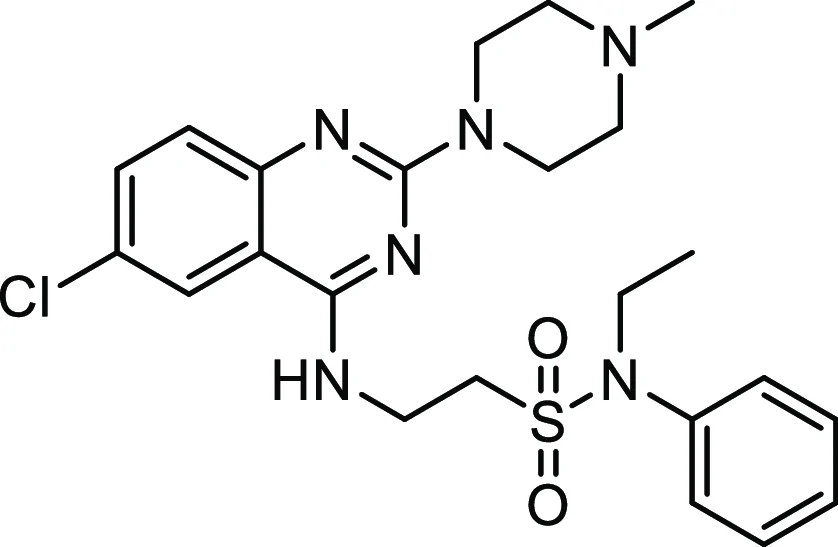


**2-((6-Chloro-2-(4-methylpiperazin-1-yl)­quinazolin-4-yl)­amino)-*N*-ethyl-*N*-phenylethane-1-sulfonamide (21) (VUF11291).**Prepared according to general procedure G from dichloride **7a** (720 mg, 3.08 mmol, 1.0 equiv), amine **5l** (820 mg, 3.10 mmol, 1.0 equiv), DIPEA (1.13 mL, 6.47 mmol, 2.1 equiv), and *N*-methylpiperazine (3.42 mL, 30.8 mmol, 10 equiv) in EtOAc (10 mL) to yield the title compound as a white solid (1.07 g, 70%). **ULCMS** (acidic mode) R_t_ 1.325 min, LC purity >98% (254 nm), *m*/*z* [M + H]^+^ calculated 489.18, found 489.15. ^**1**^ **H NMR** (CDCl_3_, 600 MHz): δ 7.43 (dd, J = 8.9, 2.3 Hz, 1H), 7.40–7.35 (m, 4H), 7.34–7.30 (m, 3H), 6.19 (app t, J = 5.7 Hz, 1H), 4.07 (app q, J = 5.9 Hz, 2H), 3.93–3.83 (m, 4H), 3.76 (q, J = 7.1 Hz, 2H), 3.39 (app t, J = 5.9 Hz, 2H), 2.45 (app t, J = 5.1 Hz, 4H), 2.34 (s, 3H), 1.14 (t, J = 7.1 Hz, 3H). ^**13**^ **C NMR** (CDCl_3_, 151 MHz): δ 158.81, 158.78, 151.0, 138.5, 133.4, 129.7, 129.0, 128.4, 127.6, 126.1, 120.5, 111.0, 55.3, 49.7, 46.44, 46.41, 43.9, 35.8, 14.8. **HRMS** *m*/*z* [M + H]^+^ C_23_H_30_ClN_6_O_2_S^+^ calculated 489.1834, found 489.1832.
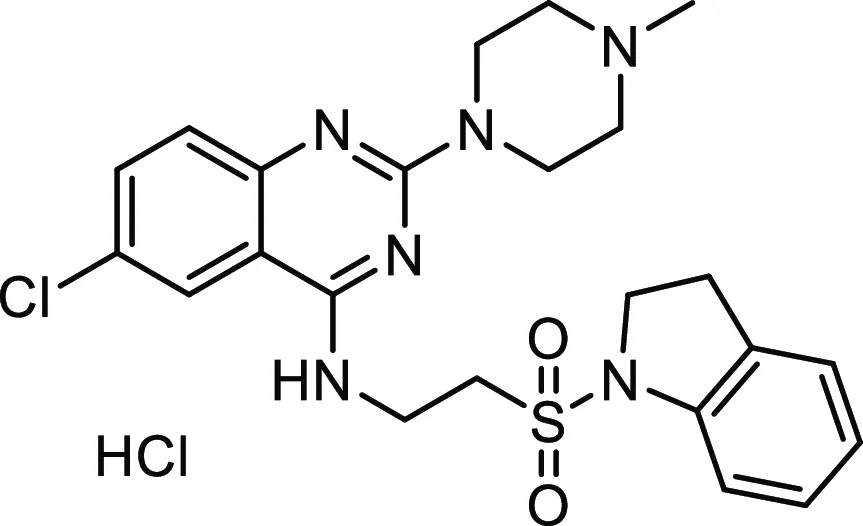


**6-Chloro-*N*-(2-(indolin-1-ylsulfonyl)­ethyl)-2-(4-methylpiperazin-1-yl)­quinazolin-4-amine hydrochloride (22) (VUF11089).**Prepared according to general procedure G from dichloride **7a** (200 mg, 0.857 mmol, 1.0 equiv), amine **5m** (225 mg, 0.857 mmol, 1.0 equiv), DIPEA (0.320 mL, 1.84 mmol, 2.1 equiv), and *N*-methylpiperazine (0.952 mL, 8.57 mmol, 10 equiv) in EtOAc (3 mL) to yield the title compound as a white solid (273 mg, 61%). **ULCMS** (acidic mode) R_t_ 1.309 min, LC purity >98% (254 nm), *m*/*z* [M + H]^+^ calculated 487.16, found 487.15. ^**1**^ **H NMR** (DMSO-d_6_, 500 MHz): δ 8.38 (br, 1H), 8.03 (d, J = 2.4 Hz, 1H), 7.54 (dd, J = 8.9, 2.4 Hz, 1H), 7.30 (d, J = 9.0 Hz, 1H), 7.26–7.13 (m, 3H), 6.97 (app td, J = 7.4, 1.1 Hz, 1H), 3.99 (t, J = 8.5 Hz, 2H), 3.89–3.78 (m, 2H), 3.56 (app t, J = 6.8 Hz, 2H), 3.07 (t, J = 8.5 Hz, 2H), 2.61 (s, 1H). ^**13**^ **C NMR** (DMSO-d_6_, 126 MHz): δ 159.5, 142.1, 133.4, 132.1, 128.0, 127.6, 126.0, 123.8, 122.5, 113.5, 111.8, 50.2, 46.7, 35.5, 27.9. The ^1^H and ^13^C spectra miss the piperazine proton signals and six carbon signals, respectively, which could not be identified despite extensive 2D NMR efforts. **HRMS** *m*/*z* [M + H]^+^ C_23_H_28_ClN_6_O_2_S^+^ calculated 487.1677, found 487.1680.
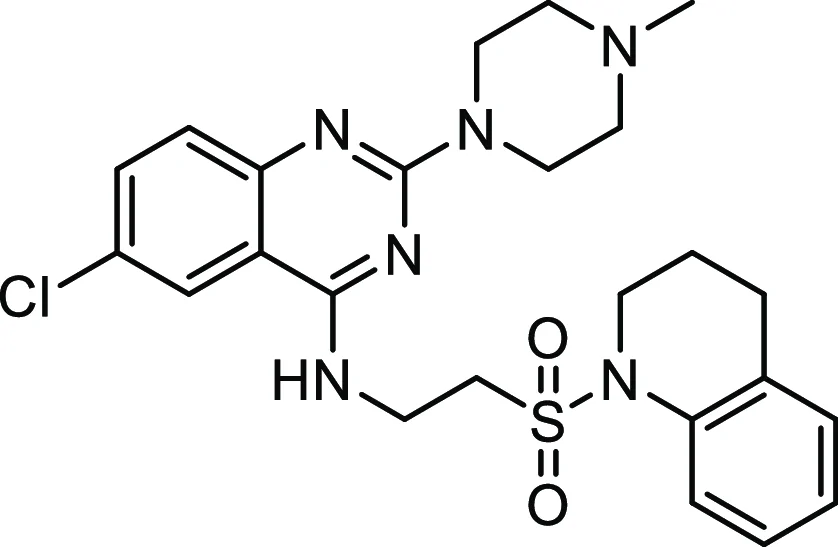


**6-Chloro-*N*-(2-((3,4-dihydroquinolin-1­(2H)-yl)­sulfonyl)­ethyl)-2-(4-methylpiperazin-1-yl)­quinazolin-4-amine (23) (VUF11292).**Prepared according to general procedure G from dichloride **7a** (930 mg, 3.98 mmol, 1.0 equiv), amine **5n** (1.10 g, 3.97 mmol, 1.0 equiv), DIPEA (1.46 mL, 8.36 mmol, 2.1 equiv), and *N*-methylpiperazine (4.42 mL, 39.8 mmol, 10 equiv) in EtOAc (10 mL) to yield the title compound as a white solid (1.44 g, 7.0%). **ULCMS** (acidic mode) R_t_ 1.329 min, LC purity >99% (254 nm), *m*/*z* [M + H]^+^ calculated 501.18, found 501.30. ^**1**^ **H NMR** (CDCl_3_, 600 MHz): δ 7.67 (d, J = 8.0 Hz, 1H), 7.44 (dd, J = 8.9, 2.2 Hz, 1H), 7.36 (d, J = 8.9 Hz, 1H), 7.34 (d, J = 2.3 Hz, 1H), 7.16–7.11 (m, 1H), 7.10–7.07 (m, 1H), 7.06–7.03 (m, 1H), 6.02 (app t, J = 5.8 Hz, 1H), 4.05 (app q, J = 5.9 Hz, 2H), 3.90–3.78 (m, 6H), 3.44 (app t, J = 6.1 Hz, 2H), 2.78 (t, J = 6.7 Hz, 2H), 2.45 (app t, J = 5.1 Hz, 4H), 2.34 (s, 3H), 2.01–1.92 (m, 2H). ^**13**^ **C NMR** (CDCl_3_, 151 MHz): δ 158.7, 158.7, 151.0, 136.6, 133.5, 130.1, 129.3, 127.6, 127.1, 126.1, 124.9, 122.4, 120.5, 110.9, 55.2, 50.8, 46.8, 46.4, 43.9, 35.9, 27.1, 22.7. **HRMS** *m*/*z* [M + H]^+^ C_24_H_30_ClN_6_O_2_S^+^ calculated 501.1834, found 501.1831.
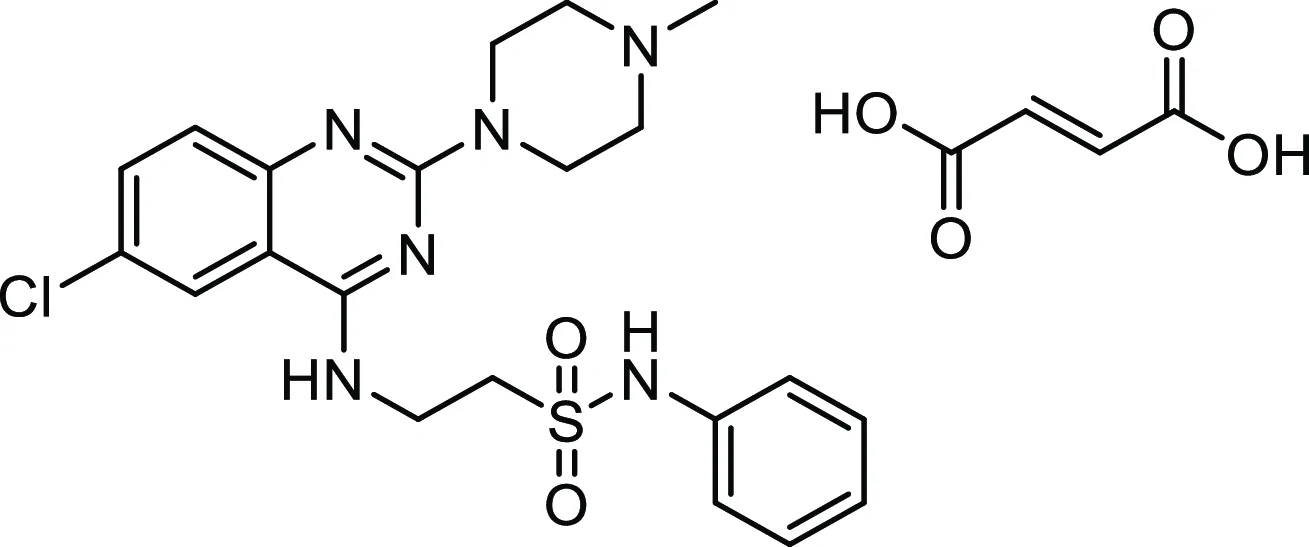


**2-((6-Chloro-2-(4-methylpiperazin-1-yl)­quinazolin-4-yl)­amino)-*N*-phenylethane-1-sulfonamide fumarate (24) (VUF10519).**Prepared according to general procedure G from dichloride **7a** (200 mg, 0.857 mmol, 1.0 equiv), amine **5o** (172 mg, 0.727 mmol, 0.8 equiv), DIPEA (0.320 mL, 1.84 mmol, 2.1 equiv), and *N*-methylpiperazine (0.952 mL, 8.57 mmol, 10 equiv) in EtOAc (3 mL). The product was converted to its corresponding fumaric acid salt using fumaric acid (99.4 mg, 0.857 mmol, 1.0 equiv) to yield the title compound as a white solid (312 mg, 57%). **ULCMS** (acidic mode) R_t_ 1.228 min, LC purity >97% (254 nm), *m*/*z* [M + H]^+^ calculated 461.14, found 461.10. ^**1**^ **H NMR** (DMSO-d_6_, 600 MHz): δ 8.24 (app t, J = 5.6 Hz, 1H), 7.98 (d, J = 2.4 Hz, 1H), 7.50 (dd, J = 8.9, 2.4 Hz, 1H), 7.32–7.24 (m, 3H), 7.32–7.24 (m, 3H), 7.22–7.17 (m, 2H), 7.08 (tt, J = 7.3, 1.2 Hz, 1H), 6.60 (s, 2H), 3.83–3.77 (m, 2H), 3.74–3.67 (m, 4H), 3.50–3.42 (m, 2H), 2.43 (app t, J = 5.0 Hz, 4H), 2.30 (s, 3H). ^**13**^ **C NMR** (DMSO-d_6_, 151 MHz): δ 166.4, 158.9, 158.3, 150.4, 138.1, 134.3, 132.8, 129.3, 127.0, 124.3, 123.9, 122.0, 119.4, 111.2, 54.0, 49.0, 45.2, 42.8, 35.4. **HRMS** *m*/*z* [M + H]^+^ C_21_H_26_ClN_6_O_2_S^+^ calculated 461.1521, found 461.1522.
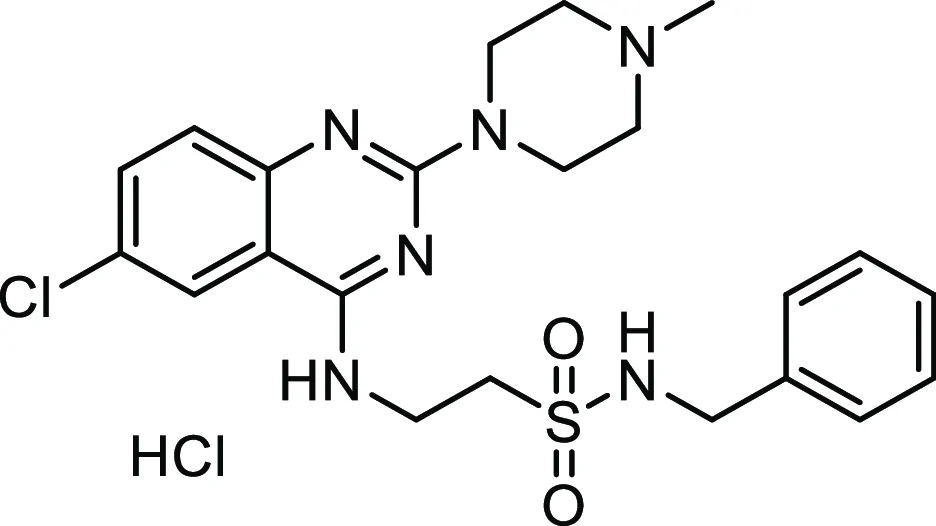


***N*-Benzyl-2-((6-chloro-2-(4-methylpiperazin-1-yl)­quinazolin-4-yl)­amino)­ethane-1-sulfonamide hydrochloride (25) (VUF26229).**Prepared according to general procedure G from dichloride **7a** (200 mg, 0.857 mmol, 1.0 equiv), amine **5p** (229 mg, 0.857 mmol, 1.0 equiv), DIPEA (0.373 mL, 2.14 mmol, 2.5 equiv), and *N*-methylpiperazine (0.952 mL, 8.57 mmol, 10 equiv) in EtOAc (3 mL). The product was converted to its corresponding HCl salt using 4 N HCl in dioxane (0.214 mL, 0.857 mmol, 1.0 equiv) to yield the title compound as a yellow oil (367 mg, 84%). **LCMS** R_t_ 1.260 min, purity >99%, calculated [M + H]^+^ 475.16, found 475.15. ^**1**^ **H NMR** (CD_3_OD, 600 MHz): δ 8.21 (d, J = 2.2 Hz, 1H), 7.84 (dd, J = 8.9, 2.2 Hz, 1H), 7.76 (d, J = 8.9 Hz, 1H), 7.39–7.33 (m, 2H), 7.33–7.29 (m, 2H), 7.26 (tt, J = 7.2, 1.4 Hz, 1H), 4.27 (s, 2H), 4.08 (app t, J = 6.7 Hz, 2H), 3.39 (app t, J = 6.7 Hz, 3H), 2.98 (s, 3H). ^**13**^ **C NMR** (CD_3_OD, 151 MHz): δ 159.4, 152.0, 137.9, 135.4, 130.7, 128.3, 127.6, 127.3, 123.1, 111.2, 52.3, 50.2, 46.2, 42.3, 36.4. The ^1^H and ^13^C spectra miss two proton signals and three carbon signals, respectively, which could not be identified despite extensive 2D NMR efforts. **HRMS** *m*/*z* [M + H]^+^ C_22_H_28_ClN_6_O_2_S^+^ calculated 475.1677, found 475.1680.
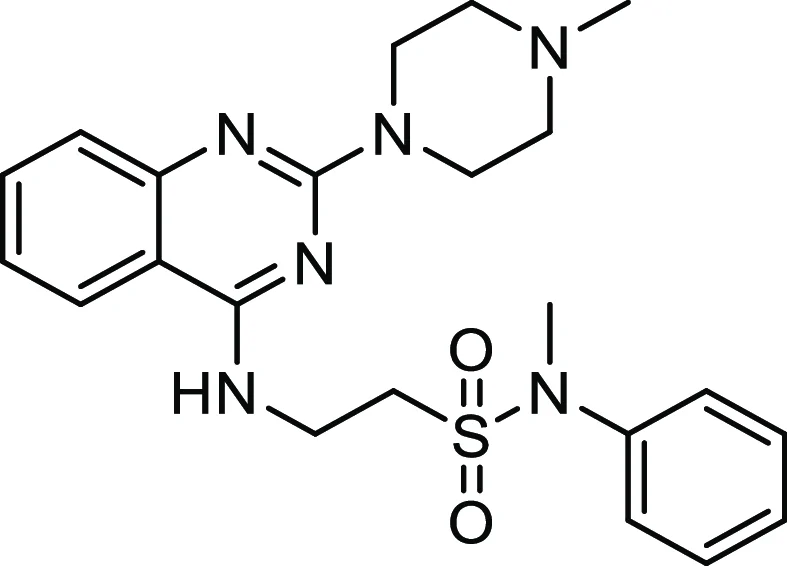


***N*** **-methyl-2-((2-(4-methylpiperazin-1-yl)­quinazolin-4-yl)­amino)-*N*-phenylethane-1-sulfonamide (26) (VUF11347).**Prepared according to general procedure G from 2,4-dichloroquinazoline (180 mg, 0.904 mmol, 1.0 equiv), amine **5g** (227 mg, 0.905 mmol, 1.0 equiv), DIPEA (0.330 mL, 1.90 mmol, 2.1 equiv), and *N*-methylpiperazine (1.00 mL, 9.04 mmol, 10 equiv) in EtOAc (3 mL) to yield the title compound as a white solid (185 mg, 47%). **ULCMS** (acidic mode) R_t_ 1.173 min, LC purity >99% (254 nm), *m*/*z* [M + H]^+^ calculated 441.20, found 441.55. ^**1**^ **H NMR** (CDCl_3_, 600 MHz): δ 7.51 (ddd, J = 8.2, 6.9, 1.3 Hz, 1H), 7.46–7.43 (m, 2H), 7.38–7.32 (m, 4H), 7.29–7.26 (m, 1H), 7.06 (ddd, J = 8.2, 6.8, 1.3 Hz, 1H), 6.24 (app t, J = 5.9 Hz, 1H), 4.08 (app q, J = 5.9 Hz, 2H), 3.92–3.83 (m, 4H), 3.39 (app t, J = 6.2 Hz, 2H), 3.34 (s, 3H), 2.46 (app t, J = 5.1 Hz, 4H), 2.34 (s, 3H). ^**13**^ **C NMR** (CDCl_3_, 151 MHz): δ 159.6, 158.7, 152.3, 141.0, 133.0, 129.6, 127.7, 126.5, 126.0, 121.4, 121.0, 110.5, 55.3, 48.2, 46.4, 44.0, 38.5, 35.6. **HRMS** *m*/*z* [M + H]^+^ C_22_H_29_N_6_O_2_S^+^ calculated 441.2067, found 441.2062.
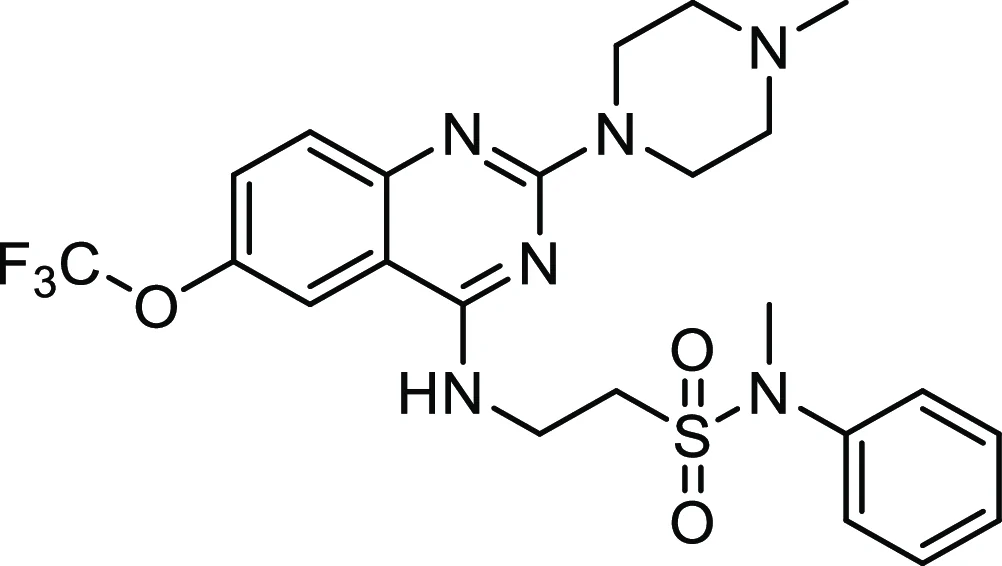


***N*** **-Methyl-2-((2-(4-methylpiperazin-1-yl)-6-(trifluoromethoxy)­quinazolin-4-yl)­amino)-*N*-phenylethane-1-sulfonamide (27) (VUF11602).**Prepared according to general procedure G from dichloride **7b** (200 mg, 0.707 mmol, 1.0 equiv), amine **5g** (177 mg, 0.707 mmol, 1.0 equiv), DIPEA (0.259 mL, 1.48 mmol, 2.1 equiv), and *N*-methylpiperazine (0.785 mL, 7.07 mmol, 10 equiv) in EtOAc (3 mL) to yield the title compound as a white solid (110 mg, 23%). **ULCMS** (acidic mode) R_t_ 1.368 min, LC purity >96% (254 nm), *m*/*z* [M + H]^+^ calculated 525.18, found 525.20. ^**1**^ **H NMR** (CDCl_3_, 600 MHz): δ 7.44 (d, J = 9.1 Hz, 1H), 7.40–7.32 (m, 5H), 7.28–7.26 (m, 2H), 6.18 (app t, J = 5.7 Hz, 1H), 4.06 (app q, J = 5.9 Hz, 2H), 3.93–3.84 (m, 4H), 3.39 (app t, J = 6.1 Hz, 2H), 3.35 (s, 3H), 2.47 (app t, J = 5.1 Hz, 4H), 2.35 (s, 3H). ^**13**^ **C NMR** (CDCl_3_, 151 MHz): δ 159.3, 158.9, 151.2, 142.8, 141.0, 129.6, 127.8, 127.7, 127.0, 126.5, 120.7 (q, J = 256.9 Hz), 113.3, 110.0, 55.2, 48.0, 46.3, 43.9, 38.5, 35.7. ^**19**^ **F NMR** (CDCl_3_, 471 MHz): δ −58.1 (s, 3F). **HRMS** *m*/*z* [M + H]^+^ C_23_H_28_F_3_N_6_O_3_S^+^ calculated 525.1890, found 525.1882.
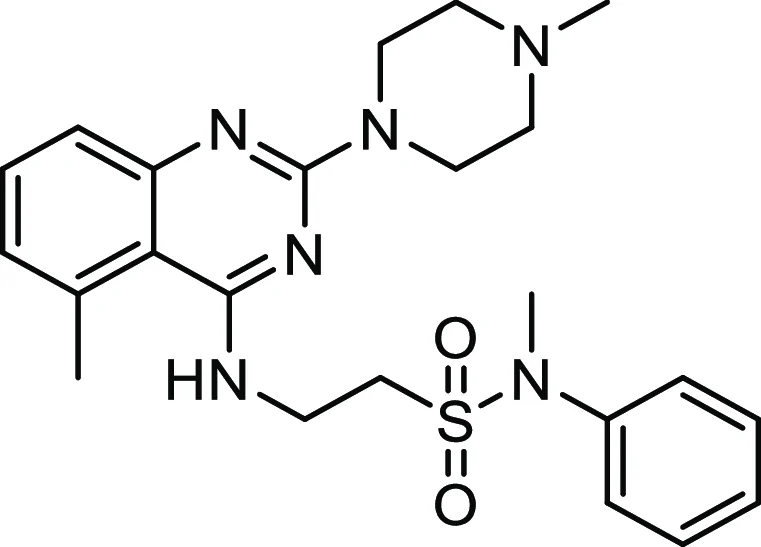


***N*** **-Methyl-2-((5-methyl-2-(4-methylpiperazin-1-yl)­quinazolin-4-yl)­amino)-*N*-phenylethane-1-sulfonamide (28) (VUF11348).**Prepared according to general procedure G from dichloride **7c** (200 mg, 0.939 mmol, 1.0 equiv), amine **5g** (236 mg, 0.941 mmol, 1.0 equiv), DIPEA (0.343 mL, 1.97 mmol, 2.1 equiv), and *N*-methylpiperazine (1.04 mL, 9.39 mmol, 10 equiv) in EtOAc (3 mL) to yield the title compound as a white solid (200 mg, 85%). **ULCMS** (acidic mode) R_t_ 1.251 min, LC purity >97% (254 nm), *m*/*z* [M + H]^+^ calculated 455.22, found 455.15. ^**1**^ **H NMR** (DMSO-d_6_, 600 MHz): δ 7.46–7.37 (m, 4H), 7.33 (dd, J = 8.4, 7.1 Hz, 1H), 7.30 (tt, J = 7.1, 1.3 Hz, 1H), 7.12 (d, J = 7.9 Hz, 1H), 6.98 (app t, J = 5.6 Hz, 1H), 6.82 (d, J = 7.1 Hz, 1H), 3.92–3.82 (m, 2H), 3.73–3.61 (m, 4H), 3.53–3.46 (m, 2H), 3.28 (s, 3H), 2.69 (s, 3H), 2.26 (app t, J = 5.0 Hz, 4H), 2.19 (s, 3H). ^**13**^ **C NMR** (DMSO-d_6_, 151 MHz): δ 160.4, 157.3, 153.8, 141.3, 134.1, 131.8, 129.1, 127.1, 126.4, 124.0, 123.7, 110.6, 54.5, 45.9, 45.8, 43.3, 37.8, 35.8, 23.2. **HRMS** *m*/*z* [M + H]^+^ C_23_H_31_N_6_O_2_S^+^ calculated 455.2225, found 455.2221.
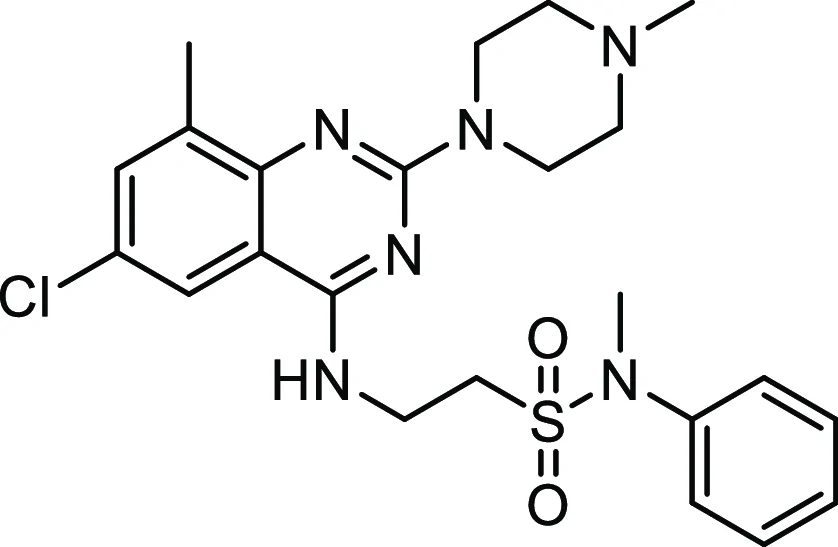


**2-((6-Chloro-8-methyl-2-(4-methylpiperazin-1-yl)­quinazolin-4-yl)­amino)-** ***N*** **-methyl-*N*-phenylethane-1-sulfonamide (29) (VUF11601).**Prepared according to general procedure G from dichloride **7d** (230 mg, 0.952 mmol, 1.0 equiv), amine **5g** (230 mg, 0.917 mmol, 1.0 equiv), DIPEA (0.348 mL, 2.00 mmol, 2.1 equiv), and *N*-methylpiperazine (1.06 mL, 9.52 mmol, 10 equiv) in EtOAc (3 mL) to yield the title compound as a white solid (250 mg, 56%). **ULCMS** (acidic mode) R_t_ 1.512 min, LC purity >95% (254 nm), *m*/*z* [M + H]^+^ calculated 489.18, found 489.05. ^**1**^ **H NMR** (DMSO-d_6_, 600 MHz): δ 8.23 (app t, J = 5.5 Hz, 1H), 7.87–7.84 (m, 1H), 7.45–7.36 (m, 5H), 7.29 (tt, J = 6.9, 1.9 Hz, 1H), 3.84–3.69 (m, 6H), 3.47 (app dd, J = 8.6, 5.8 Hz, 2H), 3.27 (s, 3H), 2.46–2.40 (m, 4H), 2.38 (s, 3H), 2.30 (s, 3H). ^**13**^ **C NMR** (DMSO-d_6_, 151 MHz): δ 159.3, 157.6, 149.3, 141.3, 135.5, 132.2, 129.1, 127.1, 126.3, 123.8, 119.3, 110.5, 54.1, 46.1, 45.3, 42.8, 37.9, 35.2, 17.0. **HRMS** *m*/*z* [M + H]^+^ C_23_H_30_ClN_6_O_2_S^+^ calculated 489.1834, found 489.1829.
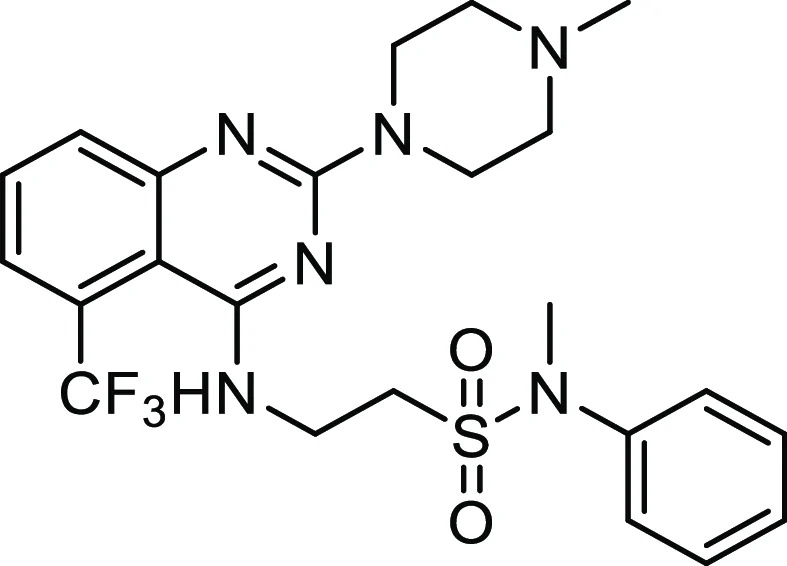


***N*** **-Methyl-2-((2-(4-methylpiperazin-1-yl)-5-(trifluoromethyl)­quinazolin-4-yl)­amino)-*N*-phenylethane-1-sulfonamide (30) (VUF15127).**Prepared according to general procedure G from dichloride **7e** (240 mg, 0.899 mmol, 1.0 equiv), amine **5g** (230 mg, 0.917 mmol, 1.0 equiv), DIPEA (0.329 mL, 1.88 mmol, 2.1 equiv), and *N*-methylpiperazine (0.998 mL, 8.99 mmol, 10 equiv) in EtOAc (3 mL) to yield the title compound as a white solid (300 mg, 66%). **ULCMS** (acidic mode) R_t_ 1.450 min, LC purity >99% (254 nm), *m*/*z* [M + H]^+^ calculated 509.19, found 509.30. ^**1**^ **H NMR** (CDCl_3_, 600 MHz): δ 7.64 (dd, J = 8.5, 1.3 Hz, 1H), 7.49 (app t, J = 8.1 Hz, 1H), 7.43 (dd, J = 7.8, 1.3 Hz, 1H), 7.38–7.33 (m, 4H), 7.29–7.27 (m, 1H), 6.44–6.36 (m, 1H), 4.11–3.99 (m, 2H), 3.91–3.76 (m, 4H), 3.41 (app t, J = 6.9 Hz, 2H), 3.33 (s, 3H), 2.42 (app t, J = 5.1 Hz, 4H), 2.33 (s, 3H). ^**13**^ **C NMR** (CDCl_3_, 151 MHz): δ 158.0, 157.9, 155.5, 141.2, 131.9, 130.8, 129.5, 127.6, 126.4, 125.6 (q, J = 273.0 Hz), 124.1 (q, J = 30.4 Hz), 121.0 (q, J = 7.5 Hz), 106.9, 55.2, 47.4, 46.4, 43.8, 38.4, 36.8. ^**19**^ **F NMR** (CDCl_3_, 471 MHz): δ −55.3 (s, 3F). **HRMS** *m*/*z* [M + H]^+^ C_23_H_28_F_3_N_6_O_2_S^+^ calculated 509.1941, found 509.1939.
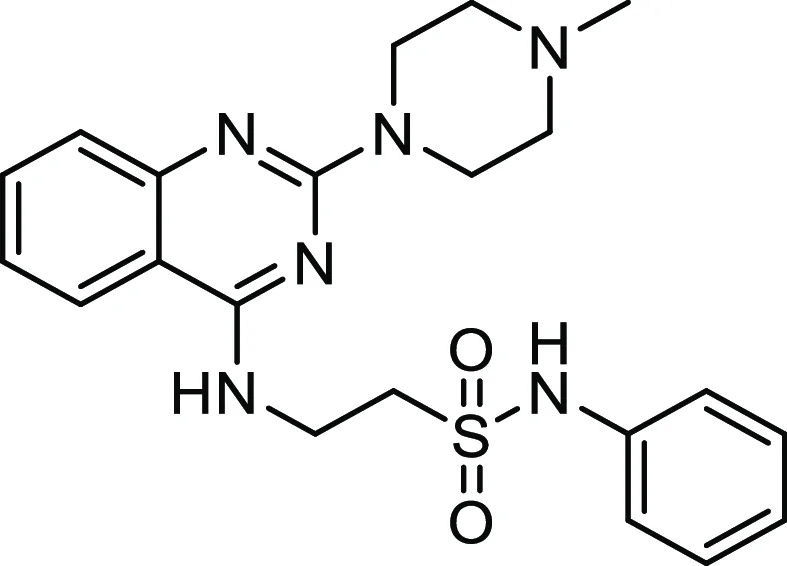


**2-((2-(4-Methylpiperazin-1-yl)­quinazolin-4-yl)­amino)-*N*-phenylethane-1-sulfonamide (31) (VUF15133).**Prepared according to general procedure G from 2,4-dichloroquinazoline (179 mg, 0.899 mmol, 1.0 equiv), amine **5o** (210 mg, 0.887 mmol, 1.0 equiv), DIPEA (0.329 mL, 1.89 mmol, 2.1 equiv), and *N*-methylpiperazine (0.988 mL, 8.99 mmol, 10 equiv) in EtOAc (3 mL) to yield the title compound as a white solid (160 mg, 91%). **ULCMS** (acidic mode) R_t_ 1.117 min, LC purity >95% (254 nm), *m*/*z* [M + H]^+^ calculated 427.18, found 427.15. ^**1**^ **H NMR** (DMSO-d_6_, 600 MHz): δ 8.11 (app t, J = 5.6 Hz, 1H), 7.83 (d, J = 8.1 Hz, 1H), 7.52–7.46 (m, 1H), 7.31–7.20 (m, 5H), 7.08 (tt, J = 7.3, 1.1 Hz, 1H), 7.05 (app dd, J = 7.4, 1.2 Hz, 1H), 3.84–3.78 (m, 2H), 3.70–3.60 (m, 4H), 3.50–3.42 (m, 3H), 2.26 (app t, J = 5.0 Hz, 4H), 2.19 (s, 3H). ^**13**^ **C NMR** (DMSO-d_6_, 151 MHz): δ 159.6, 158.2, 151.7, 138.1, 132.5, 129.3, 125.1, 123.9, 122.5, 120.6, 119.4, 110.4, 54.6, 49.2, 45.9, 43.3, 35.3. **HRMS** *m*/*z* [M + H]^+^ C_21_H_27_N_6_O_2_S^+^ calculated 427.1911, found 427.1905.
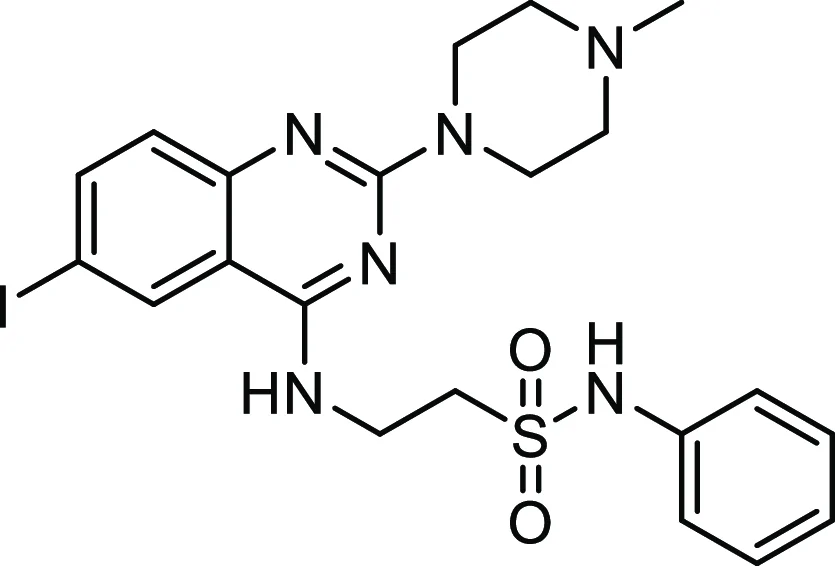


**2-((6-Iodo-2-(4-methylpiperazin-1-yl)­quinazolin-4-yl)­amino)-*N*-phenylethane-1-sulfonamide (32) (VUF10659).**Prepared according to general procedure G from dichloride **7f** (200 mg, 0.616 mmol, 1.0 equiv), amine **5o** (134 mg, 0.566 mmol, 1.0 equiv), DIPEA (0.225 mL, 1.29 mmol, 2.1 equiv), and *N*-methylpiperazine (0.684 mL, 6.16 mmol, 10 equiv) in EtOAc (3 mL) to yield the title compound as a white solid (261 mg, 76%). **ULCMS** (acidic mode) R_t_ 1.269 min, LC purity >96% (254 nm), *m*/*z* [M + H]^+^ calculated 553.08, found 553.10. ^**1**^ **H NMR** (DMSO-d_6_, 600 MHz): δ 8.24 (d, J = 2.0 Hz, 1H), 8.19 (app t, J = 5.6 Hz, 1H), 7.71 (dd, J = 8.8, 2.0 Hz, 1H), 7.29 (app dd, J = 8.5, 7.3 Hz, 2H), 7.23–7.16 (m, 2H), 7.08 (tt, J = 7.4, 1.1 Hz, 1H), 7.04 (d, J = 8.8 Hz, 1H), 3.83–3.74 (m, 2H), 3.71–3.61 (m, 4H), 3.48–3.43 (m, 2H), 2.27 (app t, J = 5.0 Hz, 4H), 2.20 (s, 3H). ^**13**^ **C NMR** (DMSO-d_6_, 151 MHz): δ 158.4, 158.2, 151.0, 140.6, 138.1, 131.2, 129.3, 127.3, 123.9, 119.4, 112.6, 83.2, 54.5, 49.0, 45.8, 43.2, 35.4. **HRMS** *m*/*z* [M + H]^+^ C_21_H_26_IN_6_O_2_S^+^ calculated 553.0877, found 553.0875.
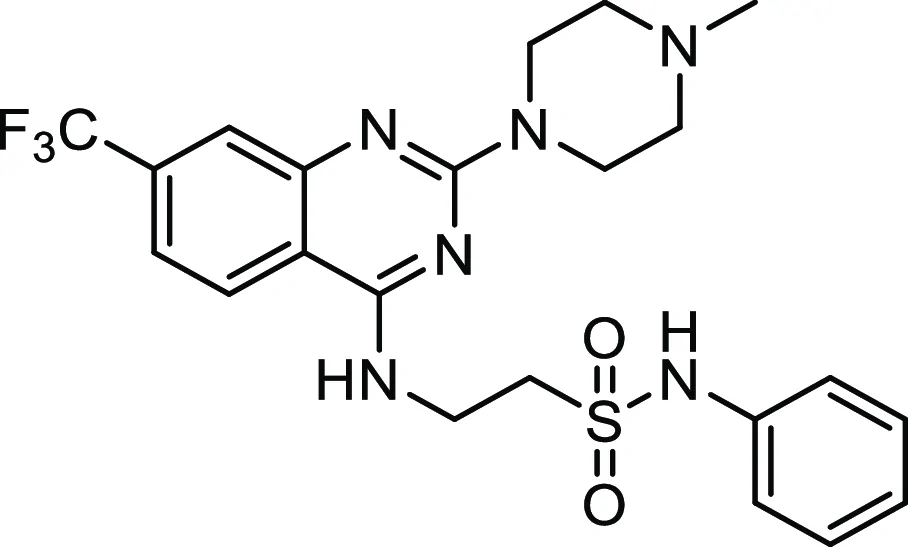


**2-((2-(4-Methylpiperazin-1-yl)-7-(trifluoromethyl)­quinazolin-4-yl)­amino)-*N*-phenylethane-1-sulfonamide (33) (VUF26160).**Prepared according to general procedure G from 2,4-dichloro-7-(trifluoromethyl)­quinazoline (174 mg, 0.652 mmol, 1.0 equiv), amine **5o** (149 mg, 0.629 mmol, 1.0 equiv), DIPEA (0.400 mL, 2.30 mmol, 3.5 equiv), and *N*-methylpiperazine (0.724 mL, 6.52 mmol, 10 equiv) in EtOAc (3 mL) to yield the title compound as a white solid (15 mg, 4.0%). **ULCMS** (acidic mode) R_t_ 1.383 min, LC purity >99% (254 nm), *m*/*z* [M + H]^+^ calculated 495.17, found 495.05. ^**1**^ **H NMR** (CDCl_3_, 500 MHz): δ 7.71 (s, 1H), 7.54 (d, J = 8.4 Hz, 1H), 7.29–7.26 (m, 2H), 7.24–7.09 (m, 4H), 6.31 (app t, J = 5.8 Hz, 1H), 4.13–4.05 (m, 2H), 3.92–3.79 (m, 4H), 3.56–3.49 (m, 2H), 2.45 (app t, J = 5.1 Hz, 4H), 2.34 (s, 3H). ^**13**^ **C NMR** (DMSO-d_6_, 151 MHz): δ 159.7, 159.2, 152.0, 138.5, 133.1 (q, J = 31.6 Hz), 129.8, 126.2 (q, J = 272.9 Hz), 125.0, 124.3, 122.4 (q, J = 4.1 Hz), 119.8, 116.1 (q, J = 3.3 Hz), 55.0, 49.4, 46.3, 43.7, 35.9. ^**19**^ **F NMR** (CDCl_3_, 471 MHz): δ −63.4 (s, 3F). **HRMS** *m*/*z* [M + H]^+^ C_22_H_26_F_3_N_6_O_2_S^+^ calculated 495.1785, found 495.1782.
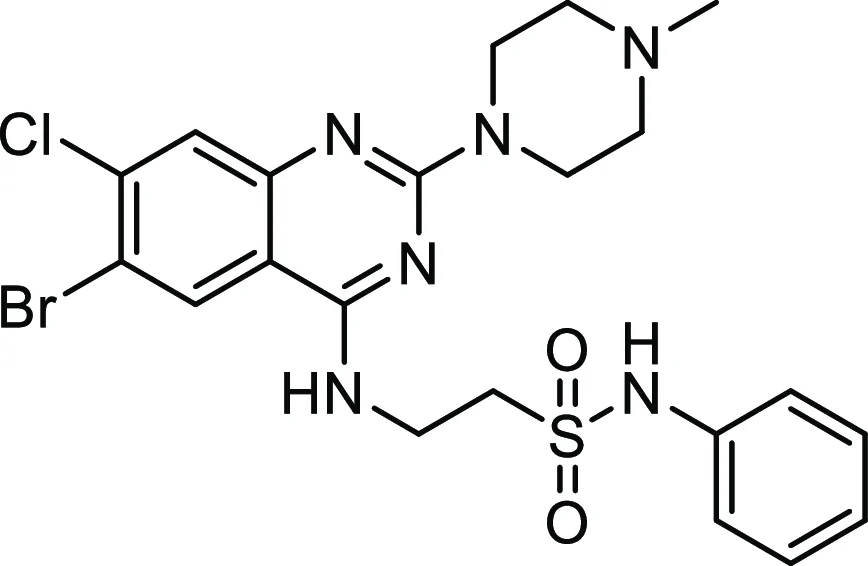


**2-((6-Bromo-7-chloro-2-(4-methylpiperazin-1-yl)­quinazolin-4-yl)­amino)-*N*-phenylethane-1-sulfonamide (34) (VUF10776).**Prepared according to general procedure G from dichloride **7g** (200 mg, 0.640 mmol, 1.0 equiv), amine **5o** (134 mg, 0.566 mmol, 0.9 equiv), DIPEA (0.234 mL, 1.34 mmol, 2.1 equiv), and *N*-methylpiperazine (0.711 mL, 6.40 mmol, 10 equiv) in EtOAc (3 mL) to yield the title compound as a white solid (208 mg, 60%). **ULCMS** (acidic mode) R_t_ 1.405 min, LC purity >98% (254 nm), *m*/*z* [M + H]^+^ calculated 539.06, found 539.00. ^**1**^ **H NMR** (DMSO-d_6_, 600 MHz): δ 8.34–8.26 (m, 2H), 7.43 (s, 1H), 7.31–7.24 (m, 2H), 7.21–7.16 (m, 2H), 7.07 (app t, J = 7.4 Hz, 1H), 3.82–3.74 (m, 2H), 3.82–3.74 (m, 4H), 3.51–3.44 (m, 2H), 2.30–2.23 (m, 4H), 2.19 (s, 3H). ^**13**^ **C NMR** (DMSO-d_6_, 151 MHz): δ 158.7, 158.5, 151.8, 138.1, 136.9, 129.3, 127.9, 125.6, 123.9, 119.4, 111.3, 110.6, 54.5, 48.9, 45.8, 43.2, 35.5. **HRMS** *m*/*z* [M + H]^+^ C_21_H_25_BrClN_6_O_2_S^+^ calculated 539.0626, found 539.0627.
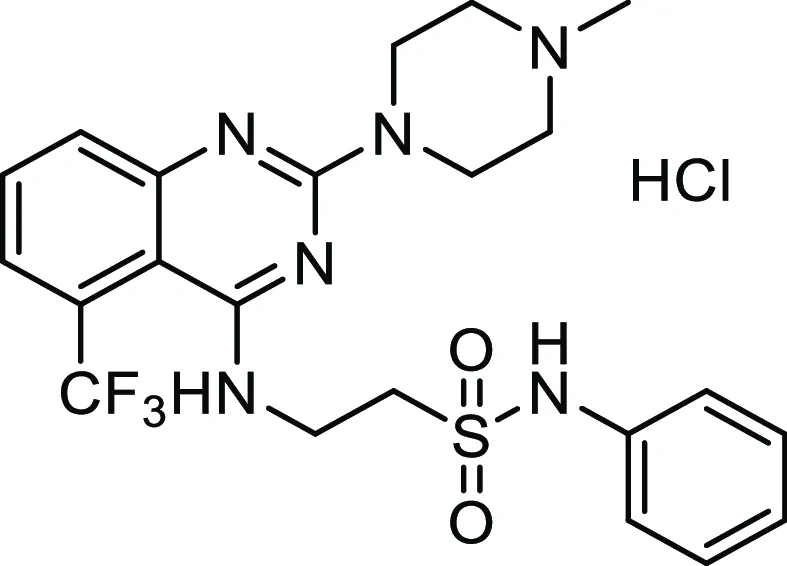


**2-((2-(4-Methylpiperazin-1-yl)-5-(trifluoromethyl)­quinazolin-4-yl)­amino)-*N*-phenylethane-1-sulfonamide hydrochloride (35.HCl) (VUF10775/GD136).** Prepared according to general procedure G from dichloride **7e** (200 mg, 0.749 mmol, 1.0 equiv), amine **5o** (164 mg, 0.693 mmol, 0.9 equiv), DIPEA (0.274 mL, 1.57 mmol, 2.1 equiv), and *N*-methylpiperazine (0.831 mL, 7.49 mmol, 10 equiv) in EtOAc (3 mL). The corresponding free base product was converted to its corresponding HCl salt using 4 N HCl in dioxane (0.187 mL, 0.749 mmol, 1.0 equiv) to yield the title compound as a white solid (308 mg, 77%).

Free base **: ULCMS** (acidic mode) R_t_ 1.431 min, LC purity >99%, *m*/*z* [M + H]^+^ calculated 495.17, found 495.55. ^**1**^ **H NMR** (DMSO-d_6_, 500 MHz): δ 7.65–7.59 (m, 1H), 7.57 (dd, J = 8.6, 1.5 Hz, 1H), 7.51 (d, J = 7.1 Hz, 1H), 7.33–7.26 (m, 2H), 7.23–7.18 (m, 2H), 7.08 (tt, J = 7.5, 1.1 Hz, 1H), 6.69–6.58 (m, 1H), 3.98–3.90 (m, 2H), 3.76–3.64 (m, 4H), 3.48–3.41 (m, 2H), 2.34–2.25 (m, 4H), 2.19 (s, 3H). ^**13**^ **C NMR** (DMSO-d_6_, 126 MHz): δ 158.2, 157.7, 155.3, 138.6, 131.8, 131.7, 129.8, 124.3, 123.7 (q, J = 283.5 Hz), 123.5 (q, J = 20.0 Hz), 121.1 (q, J = 7.7 Hz), 120.0, 106.6, 55.0, 49.6, 46.3, 43.7, 37.1. ^**19**^ **F NMR** (DMSO-d_6_, 471 MHz): δ −54.5 (s, 3F). Regiochemistry was proven in-depth by extensive 2D NMR analysis (see SI page S17).

HCl salt **: ULCMS** (acidic mode) R_t_ 1.382 min, LC purity >99% (254 nm), *m*/*z* [M + H]^+^ calculated 495.17, found 495.15. ^**1**^ **H NMR** (CD_3_OD, 600 MHz): δ 8.11 (dd, J = 8.5, 1.3 Hz, 1H), 8.00 (t, J = 8.0 Hz, 1H), 7.94 (dd, J = 7.7, 1.2 Hz, 1H), 7.35–7.29 (m, 2H), 7.28–7.23 (m, 2H), 7.14 (tt, J = 7.3, 1.2 Hz, 1H), 4.90 (br, 2H), 4.21 (app t, J = 6.7 Hz, 2H), 3.69 (br, 4H), 3.55 (app t, J = 6.7 Hz, 2H), 3.34 (br, 2H), 3.00 (s, 3H). ^**13**^ **C NMR** (CD_3_OD, 151 MHz): δ 160.1, 151.9, 144.1, 138.9, 136.0, 130.6, 126.5 (q, J = 30.9 Hz), 126.3 (q, J = 6.9 Hz), 125.9, 125.7 (q, J = 272.4 Hz), 124.3, 121.6, 108.4, 53.7, 50.0, 43.68, 43.67, 39.1. ^**19**^ **F NMR** (CD_3_OD, 471 MHz): δ −57.5 (s, 3F). **HRMS** *m*/*z* [M + H]^+^ C_22_H_26_F_3_N_6_O_2_^+^ calculated 495.1785, found 495.1789. Regiochemistry was proven in-depth by extensive 2D NMR analysis (see SI page S24).
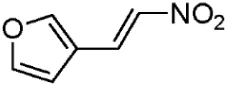


**((E)-3-(2-Nitrovinyl)­furan (36a).**Prepared according to general procedure H from furan-3-carbaldehyde (2.70 mL, 31.2 mmol, 1.0 equiv), NH_4_OAc (0.726 mg, 9.42 mmol, 0.3 equiv) and CH_3_NO_2_ (7 mL) to yield the title compound as a bright yellow solid (3.55 g, 82%). ^**1**^ **H NMR** (CDCl_3_, 250 MHz): δ 7.94 (d, *J* = 13.5 Hz, 1H), 7.84 (app s, 1H), 7.52 (app s, 1H), 7.39 (d, *J* = 13.5 Hz, 1H), 6.57 (d, *J* = 1.8 Hz, 1H).
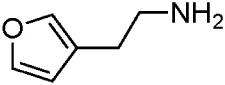


**2-(Furan-3-yl)­ethan-1-amine (37a).**Prepared according to general procedure I from nitroalkene **36a** ((*E*)-3-(2-nitrovinyl)­furan) (3.55 g, 25.5 mmol, 1.0 equiv) and LiAlH_4_ (2.6 M in THF, 27.6 mL, 66.3 mmol, 2.6 equiv) to yield the title compound as a yellow oil (624 mg, 22%). ^**1**^ **H NMR** (CDCl_3_, 250 MHz): δ 7.35 (app s, 1H), 7.24 (app s, 1H), 6.26 (app s, 1H), 2.86 (t, *J* = 6.7 Hz, 2H), 2.53 (t, *J* = 6.7 Hz, 2H), 1.49–1.32 (m, 1H).
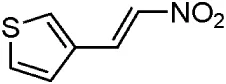


**(** ***E*** **)-3-(2-Nitrovinyl)­thiophene (36b).**Prepared according to general procedure H from thiophene-3-carbaldehyde (2.59 mL, 31.2 mmol, 1.0 equiv), NH_4_OAc (0.726 mg, 9.42 mmol, 0.3 equiv) and CH_3_NO_2_ (8 mL) to yield the title compound as a bright yellow solid (2.87 g, 66%). ^**1**^ **H NMR** (CDCl_3_, 250 MHz): δ 8.01 (d, *J* = 13.6 Hz, 1H), 7.73 (d, *J* = 2.2 Hz, 1H), 7.54–7.39 (m, 2H), 7.31–7.23 (m, 1H).
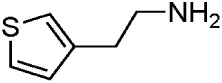


**2-(Thiophen-3-yl)­ethan-1-amine (37b).**Prepared according to general procedure I from nitroalkene **36b** ((*E*)-3-(2-nitrovinyl)­thiophene) (1.78 g, 11.4 mmol, 1.0 equiv) and LiAlH_4_ (2.6 M in THF, 12.4 mL, 29.7 mmol, 2.6 equiv) to yield the title compound as a yellow oil (550 mg, 38%). ^**1**^ **H NMR** (CDCl_3_, 250 MHz): δ 7.30–7.27 (m, 1H), 7.00 (d, *J* = 2.4 Hz, 1H), 6.95 (d, *J* = 4.9 Hz, 1H), 2.96 (t, *J* = 6.6 Hz, 2H), 2.78 (t, *J* = 6.7 Hz, 2H), 1.60–1.50 (m, 2H).
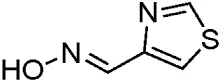


**(** ***E*** **)-Thiazole-4-carbaldehyde oxime (38a).**Prepared according to general procedure J from thiazole-4-carbaldehyde (200 mg, 1.77 mmol, 1.0 equiv), H_2_NOH·HCl (221 mg, 3.18 mmol, 1.8 equiv), NaOAc (290 mg, 3.54 mmol, 2.0 equiv) and EtOH to yield the title compound as a yellow solid (173 mg, 76%). ^**1**^ **H NMR** (CD_3_OD, 400 MHz): δ 9.03 (s, 1H), 8.23 (s, 1H), 7.68 (s, 1H).
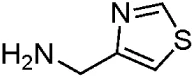


**Thiazol-4-ylmethanamine (39a).**Prepared according to general procedure K from oxime **38a** ((*E*)-thiazole-4-carbaldehyde oxime) (173 mg, 1.35 mmol, 1.0 equiv), Zn (88 mg, 1.35 mmol, 1.0 equiv) and AcOH to yield the title compound as a yellow oil (93 mg, 60%). ^**1**^ **H NMR** (CDCl_3_, 400 MHz): δ 8.80 (s, 1H), 7.19 (s, 1H), 4.15–4.05 (m, 2H).
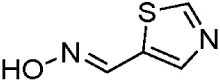


**(** ***E*** **)-Thiazole-5-carbaldehyde oxime (38b).**Prepared according to general procedure J from thiazole-5-carbaldehyde (200 mg, 1.77 mmol, 1.0 equiv), H_2_NOH·HCl (221 mg, 3.18 mmol, 1.8 equiv) and NaOAc (290 mg, 3.54 mmol, 2.0 equiv) and EtOH to yield the title compound as a yellow solid (203 mg, 90%). ^**1**^ **H NMR** (CD_3_OD, 400 MHz): δ 9.09 (s, 1H), 8.24 (s, 1H), 7.89 (s, 1H).
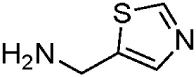


**Thiazol-5-ylmethanamine (39b).**Prepared according to general procedure K from oxime **38b** ((*E*)-thiazole-5-carbaldehyde oxime) (203 mg, 1.58 mmol, 1.0 equiv), Zn (621 mg, 9.50 mmol, 6.0 equiv) and AcOH to yield the title compound as a yellow oil (141 mg, 78%). ^**1**^ **H NMR** (CDCl_3_, 400 MHz): δ 8.72 (s, 1H), 7.72 (s, 1H), 4.20–4.10 (m, 2H), 2.85–2.70 (m, 2H).
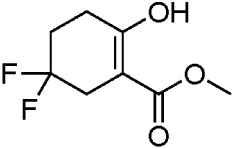


**Methyl 5,5-difluoro-2-oxocyclohexane-1-carboxylate (40).**Me_2_CO_3_ (28.3 mL, 336 mmol, 3.0 equiv) was added to a suspension of NaH (60% in mineral oil, 13.4 g, 336 mmol, 3.0 equiv) in THF (65 mL). The reaction mixture was heated to reflux and 4,4-difluorocyclohexanone (15.0 g, 112 mmol, 1.0 equiv) in THF (30 mL) was added dropwise over 15 min. Heating was continued at reflux for 2 h. The mixture was cooled to RT and diluted with Et_2_O (200 mL). The resulting mixture was carefully quenched with 1 M aq. HCl at 0 °C until pH < 1. The organic phase was separated, and the aqueous phase was extracted twice with Et_2_O (150 mL). The combined organic phases were dried over Na_2_SO_4_ and concentrated to yield the title compound as a brown oil in quantitative yield (21.5 g). ^**1**^ **H NMR** (CDCl_3_, 500 MHz): δ 12.17 (s, 1H), 3.78 (s, 3H), 2.74 (t, *J* = 14.7 Hz, 2H), 2.55 (tt, *J* = 6.9, 1.4 Hz, 2H), 2.18–2.06 (m, 2H).
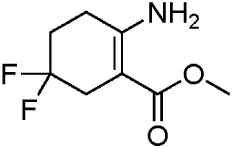


**Methyl 2-amino-5,5-difluorocyclohex-1-ene-1-carboxylate (41).**NH_4_OAc (43.1 g, 559 mmol, 5.0 equiv) was added to a solution of keto-ester **40** (21.5 g, 112 mmol, 1.0 equiv) in MeOH (600 mL). The reaction mixture was stirred at RT for 14 h. The reaction mixture was concentrated. The crude product was dissolved in EtOAc (700 mL). The solution was washed thrice with H_2_O (400 mL) The organic phase was dried over Na_2_SO_4_ and concentrated to yield the title compound as a brown oil in quantitative yield (21.4 g). ^**1**^ **H NMR** (CDCl_3_, 500 MHz): δ 3.69 (s, 3H), 2.76 (t, *J* = 15.3 Hz, 2H), 2.47 (t, *J* = 6.9 Hz, 2H), 2.07 (tt, *J* = 13.3, 6.9 Hz, 2H).
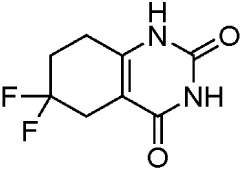


**6,6-Difluoro-5,6,7,8-tetrahydroquinazoline-2,4­(1** ***H***,**3** ***H*** **)-dione (42).**A solution of enamine **41** (22.3 g, 117 mmol, 1.0 equiv) in DCE (500 mL) was cooled to 0 °C. After the addition of pyridine (23.6 mL, 292 mmol, 2.5 equiv), COCl_2_ (20% in PhMe) (99.0 mL, 175 mmol, 1.5 equiv) was added dropwise. **CAUTION: working with COCl**_**2**_ **requires a well-ventilated fume hood and a scrubber system with aq. NaOH. Concentration by rotavap evaporation was also performed in the fume hood.** The reaction mixture was stirred at 0 °C for 2 h. Aq. NH_4_OH (25%, 291 mL, 1.87 mol, 16 equiv) was added carefully at 0 °C. The reaction mixture was heated to 50 °C for 16 h. The mixture was concentrated, and H_2_O (300 mL) was added. The resulting suspension was filtered. The solids were washed with H_2_O (700 mL) and dried *in vacuo* to yield the title compound as a white solid (19.5 g, 83%). ^**1**^ **H NMR** (DMSO-d_6_, 500 MHz): δ 2.69 (t, *J* = 14.5 Hz, 2H), 2.61–2.54 (m, 2H), 2.19 (tt, *J* = 13.8, 6.8 Hz, 2H).
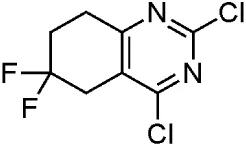


**2,4-Dichloro-6,6-difluoro-5,6,7,8-tetrahydroquinazoline (43).**DIPEA (34.2 mL, 196 mmol, 2.2 equiv) was added to a suspension of 6,6-difluoro-5,6,7,8-tetrahydroquinazoline-2,4­(1*H*,3*H*)-dione **42** (18.0 g, 89.0 mmol, 1.0 equiv) in POCl_3_ (124 mL, 1.34 mol, 15 equiv). The resulting mixture was stirred at reflux for 1 h. The reaction mixture was carefully poured over crushed ice, and the resulting suspension was stirred until all ice had melted. **CAUTION: quenching of POCl**_**3**_ **might show unpredictable behavior and complete quenching may need extended times.** The mixture was extracted thrice with DCM (100 mL) and the combined organic phases were washed with brine (100 mL). The organic phase was dried over Na_2_SO_4_ and concentrated. The crude product was purified using NP column chromatography (EtOAc in *c*Hex) to yield the title compound as a yellowish solid (15.5 g, 73%). ^**1**^ **H NMR** (CDCl_3_, 500 MHz): δ 3.25 (t, *J* = 13.9 Hz, 2H), 3.17 (t, *J* = 6.9 Hz, 2H), 2.40–2.26 (m, 2H).
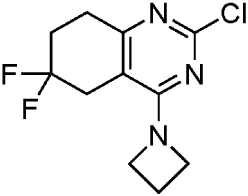


**4-(Azetidin-1-yl)-2-chloro-6,6-difluoro-5,6,7,8-tetrahydroquinazoline (44).**Prepared according to general procedure L from dichloride **43** (100 mg, 0.417 mmol, 1.0 equiv), azetidine (0.028 mL, 0.417 mmol, 1.0 equiv), DIPEA (0.153 mL, 0.877 mmol, 2.1 equiv) and MeCN (5 mL) to yield the title compound as a white solid (79 mg, 73%). ^**1**^ **H NMR** (CDCl_3_, 250 MHz): δ 4.33 (t, *J* = 7.7 Hz, 4H), 3.06 (t, *J* = 14.1 Hz, 2H), 2.92 (t, *J* = 6.9 Hz, 2H), 2.50–2.30 (m, 2H), 2.30–2.04 (m, 2H).
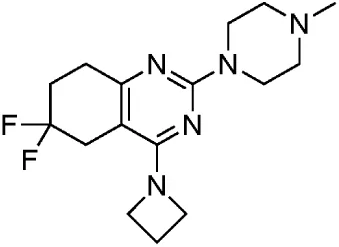


**4-(Azetidin-1-yl)-6,6-difluoro-2-(4-methylpiperazin-1-yl)-5,6,7,8-tetrahydroquinazoline (60).**Prepared according to general procedure M from monochloride **44** (79 mg, 0.304 mmol, 1.0 equiv), *N*-methylpiperazine (0.169 mL, 1.52 mmol, 5.0 equiv), DIPEA (0.112 mL, 0.639 mmol, 2.1 equiv) and dioxane (5 mL) to yield the title compound as a white solid (84 mg, 85%). **ULCMS** (acidic mode) R_t_ 1.097 min, LC purity >98% (254 nm), *m*/*z* [M + H]^+^ calculated 324.39, measured 324.20. ^**1**^ **H NMR** (CDCl_3_, 600 MHz): δ 4.18 (t, *J* = 7.6 Hz, 4H), 3.95–3.80 (m, 4H), 2.96 (t, *J* = 14.5 Hz, 2H), 2.80 (t, *J* = 6.9 Hz, 2H), 2.67–2.53 (m, 4H), 2.44 (s, 3H), 2.31 (p, *J* = 7.7 Hz, 2H), 2.21–2.14 (m, 2H). ^**13**^ **C NMR** (CDCl_3_, 151 MHz): δ 163.3, 160.8#, 159.6#, 122.4 (t, *J* = 239.9 Hz), 97.9 (t, *J* = 6.3 Hz), 54.6, 52.0, 45.6, 43.0, 33.1 (t, *J* = 27.9 Hz), 30.1 (t, *J* = 24.3 Hz), 29.5, 16.7. ^**19**^ **F NMR** (CDCl_3_, 471 MHz): δ −95.8 (s, 2F). **HRMS** *m*/*z* [M + H]^+^ C_16_H_24_F_2_N_5_^+^ calculated 324.1994, found 324.1993.
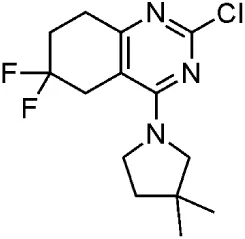


**2-Chloro-4-(3,3-dimethylpyrrolidin-1-yl)-6,6-difluoro-5,6,7,8-tetrahydroquinazoline (45).**Prepared according to general procedure L from dichloride **43** (100 mg, 0.419 mmol, 1.0 equiv), 3.3-dimethylpyrrolidine (0.052 mL, 0.419 mmol, 1.0 equiv), DIPEA (0.154 mL, 0.879 mmol, 2.1 equiv) and MeCN (5 mL) to yield the title compound as a white solid (75 mg, 59%). **^1^H NMR** (CDCl_3_, 250 MHz): δ 3.70 (t, *J* = 6.7 Hz, 2H), 3.16 (t, *J* = 14.0 Hz, 2H), 2.94 (t, *J* = 7.1 Hz, 2H), 2.31–2.10 (m, 2H), 2.01–1.86 (m, 2H), 1.81 (t, *J* = 6.2 Hz, 2H), 1.57 (s, 6H).
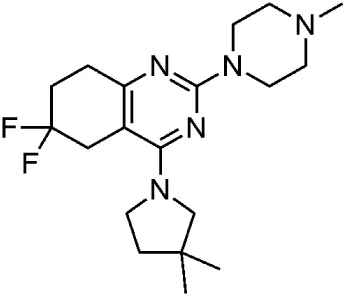


**4-(3,3-Dimethylpyrrolidin-1-yl)-6,6-difluoro-2-(4-methylpiperazin-1-yl)-5,6,7,8-tetrahydroquinazoline (61).**Prepared according to general procedure M from monochloride **45** (75 mg, 0.249 mmol, 1.0 equiv), *N*-methylpiperazine (0.138 mL, 1.24 mmol, 5.0 equiv), DIPEA (0.091 mL, 0.522 mmol, 2.1 equiv) and dioxane (5 mL) to yield the title compound as a white solid (43 mg, 47%). **ULCMS** (acidic mode) R_t_ 1.257 min, LC purity >96% (254 nm), *m*/*z* [M + H]^+^ calculated 366.47, measured 366.55. ^**1**^ **H NMR** (CDCl_3_, 600 MHz): δ 3.97–3.83 (m, 4H), 3.61 (t, *J* = 7.1 Hz, 2H), 3.05 (t, *J* = 14.3 Hz, 2H), 2.88–2.79 (m, 2H), 2.73–2.58 (m, 4H), 2.51–2.41 (m, 3H), 2.22–2.11 (m, 2H), 1.95–1.86 (m, 2H), 1.78 (t, *J* = 6.8 Hz, 2H), 1.54 (s, 6H). ^**13**^ **C NMR** (CDCl_3_, 151 MHz): δ 162.5, 156.6, 122.7 (t, *J* = 239.8 Hz), 99.4, 63.6, 54.5, 51.1, 45.4, 43.3, 42.5, 35.9 (t, *J* = 27.7 Hz), 30.0, 29.7 (t, *J* = 24.1 Hz), 25.5, 23.2. One signal missing. ^**19**^ **F NMR** (CDCl_3_, 471 MHz): δ −96.6 (s, 2F). **HRMS** *m*/*z* [M + H]^+^ C_19_H_30_F_2_N_5_^+^ calculated 366.2464, found 366.2463.
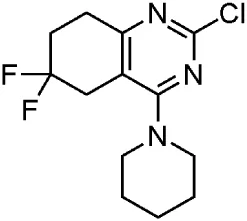


**2-Chloro-6,6-difluoro-4-(piperidin-1-yl)-5,6,7,8-tetrahydroquinazoline (46).**Prepared according to general procedure L from dichloride **43** (101 mg, 0.423 mmol, 1.0 equiv), piperidine (0.042 mL, 0.423 mmol, 1.0 equiv), DIPEA (0.155 mL, 0.889 mmol, 2.1 equiv) and MeCN (5 mL) to yield the title compound as a white solid (118 mg, 97%). ^**1**^ **H NMR** (CDCl_3_, 250 MHz): δ 3.40–3.32 (m, 4H), 3.12–2.85 (m, 4H), 2.42–2.12 (m, 2H), 1.70–1.60 (m, 6H).
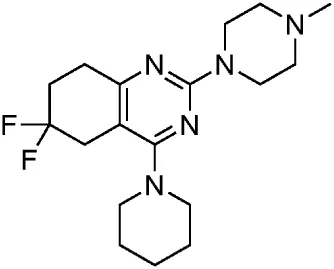


**6,6-Difluoro-2-(4-methylpiperazin-1-yl)-4-(piperidin-1-yl)-5,6,7,8-tetrahydroquinazoline (62).**Prepared according to general procedure M from monochloride **46** (118 mg, 0.410 mmol, 1.0 equiv), *N*-methylpiperazine (0.227 mL, 2.05 mmol, 5.0 equiv), DIPEA (0.150 mL, 0.861 mmol, 2.1 equiv) and dioxane (5 mL) to yield the title compound as a white solid (120 mg, 83%). **ULCMS** (acidic mode) R_t_ 1.306 min, LC purity >96% (254 nm), *m*/*z* [M + H]^+^ calculated 352.45, measured 350.50. ^**1**^ **H NMR** (CDCl_3_, 600 MHz): δ 4.01–3.78 (m, 4H), 3.27–3.15 (m, 4H), 2.94 (t, *J* = 13.7 Hz, 2H), 2.87 (t, *J* = 7.1 Hz, 2H), 2.71–2.57 (m, 4H), 2.46 (s, 3H), 2.23 (tt, *J* = 13.8, 7.1 Hz, 2H), 1.68–1.61 (m, 6H). ^**13**^ **C NMR** (CDCl_3_, 151 MHz): δ 167.1, 162.5, 159.7, 122.6 (t, *J* = 240.6 Hz), 102.3, 54.6, 49.3, 45.5, 42.9, 34.5 (t, *J* = 27.5 Hz), 30.1 (t, *J* = 24.2 Hz), 29.9 (t, *J* = 5.5 Hz), 25.9, 24.6. ^**19**^ **F NMR** (CDCl_3_, 471 MHz): δ −97.7 (s, 2F). **HRMS** *m*/*z* [M + H]^+^ C_18_H_28_F_2_N_5_^+^ calculated 352.2307, found 352.2305.
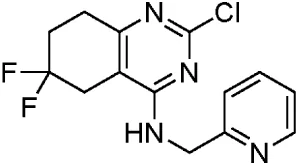


**2-Chloro-6,6-difluoro-** ***N*** **-(pyridin-2-ylmethyl)-5,6,7,8-tetrahydroquinazolin-4-amine (47).**Prepared according to general procedure L from dichloride **43** (150 mg, 0.627 mmol, 1.0 equiv), pyridin-2-ylmethanamine (0.168 mL, 1.63 mmol, 2.6 equiv), DIPEA (0.230 mL, 1.31 mmol, 2.1 equiv) and MeCN (5 mL) to yield the title compound as a white solid (35 mg, 18%). ^**1**^ **H NMR** (CDCl_3_, 250 MHz): δ 8.56 (s, 1H), 7.72 (d, *J* = 6.2 Hz, 1H). 7.40–7.16 (m, 2H), 6.72 (s, 1H), 4.76 (s, 2H), 3.01–2.97 (m, 4H), 2.26 (d, *J* = 6.4, 2H).
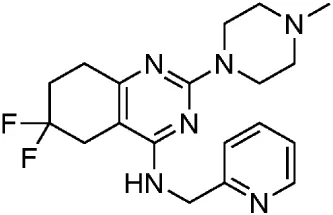


**6,6-Difluoro-2-(4-methylpiperazin-1-yl)-** ***N*** **-(pyridin-2-ylmethyl)-5,6,7,8-tetrahydroquinazolin-4-amine (63).**Prepared according to general procedure M from monochloride **47** (35 mg, 0.113 mmol, 1.0 equiv), *N*-methylpiperazine (0.062 mL, 0.563 mmol, 5.0 equiv), DIPEA (0.041 mL, 0.237 mmol, 2.1 equiv) and dioxane (5 mL) to yield the title compound as a white solid (20 mg, 47%). **ULCMS** (acidic mode) R_t_ 0.974 min, LC purity >96% (254 nm), *m*/*z* [M + H]^+^ calculated 375.44, measured 375.15. ^**1**^ **H NMR** (CDCl_3_, 600 MHz): δ 8.56 (ddd, *J* = 5.0, 1.7, 0.9 Hz, 1H), 7.69–7.61 (m, 1H), 7.30–7.27 (m, 1H), 7.20 (ddd, *J* = 7.6, 4.9, 1.1 Hz, 1H), 5.89–5.75 (m, 1H), 4.71 (d, *J* = 4.7 Hz, 2H), 4.02–3.76 (m, 4H), 2.88 (t, *J* = 14.3 Hz, 2H), 2.81 (t, *J* = 6.8 Hz, 2H), 2.62–2.52 (m, 4H), 2.41 (s, 3H), 2.21 (tt, *J* = 13.5, 6.8 Hz, 2H). ^**13**^ **C NMR** (CDCl_3_, 151 MHz): δ 160.5, 160.1, 159.7, 157.1, 149.0, 136.6, 122.7 (t, *J* = 240.7 Hz), 122.2, 121.9, 97.1 (t, *J* = 5.7 Hz), 54.6, 45.7, 45.7, 43.2, 31.7 (t, *J* = 28.0 Hz), 30.5 (t, *J* = 24.3 Hz), 29.5 (t, *J* = 5.4 Hz). ^**19**^ **F NMR** (CDCl_3_, 471 MHz): δ −95.0 (s, 2F). **HRMS** *m*/*z* [M + H]^+^ C_19_H_25_F_2_N_6_^+^ calculated 375.2103, found 375.2102.
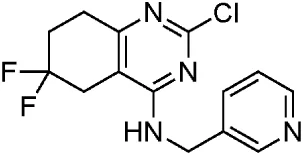


**2-Chloro-6,6-difluoro-** ***N*** **-(pyridin-3-ylmethyl)-5,6,7,8-tetrahydroquinazolin-4-amine (48).**Prepared according to general procedure L from dichloride **43** (150 mg, 0.627 mmol, 1.0 equiv), pyridin-3-ylmethanamine (0.168 mL, 1.63 mmol, 2.6 equiv), DIPEA (0.230 mL, 1.31 mmol, 2.1 equiv) and MeCN (5 mL) to yield the title compound as a white solid (120 mg, 62%). ^**1**^ **H NMR** (CDCl_3_, 250 MHz): δ 8.67–8.36 (m, 2H), 7.74 (d, *J* = 7.8 Hz, 1H), 7.38–7.19 (m, 1H), 5.40–5.30 (m, 1H), 4.72 (d, *J* = 5.6 Hz, 2H), 2.98–2.82 (m, 4H), 2.25 (tt, *J* = 13.5, 6.9 Hz, 2H).
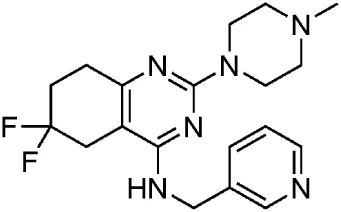


**6,6-Difluoro-2-(4-methylpiperazin-1-yl)-** ***N*** **-(pyridin-3-ylmethyl)-5,6,7,8-tetrahydroquinazolin-4-amine (64).**Prepared according to general procedure M from monochloride (120 mg, 0.386 mmol, 1.0 equiv), *N*-methylpiperazine (0.214 mL, 1.93 mmol, 5.0 equiv), DIPEA (0.142 mL, 0.811 mmol, 2.1 equiv) and dioxane (5 mL) to yield the title compound as a white solid (123 mg, 85%). **ULCMS** (acidic mode) R_t_ 0.730 min, LC purity >95% (254 nm), *m*/*z* [M + H]^+^ calculated 375.44, measured 375.20. ^**1**^ **H NMR** (CDCl_3_, 600 MHz): δ 8.58 (dd, *J* = 2.3, 0.8 Hz, 1H), 8.51 (dd, *J* = 4.8, 1.7 Hz, 1H), 7.68–7.61 (m, 1H), 7.26–7.23 (m, 1H), 4.73 (t, *J* = 5.8 Hz, 1H), 4.67 (d, *J* = 5.6 Hz, 2H), 4.00–3.72 (m, 4H), 2.89–2.72 (m, 4H), 2.63–2.50 (m, 4H), 2.42 (s, 3H), 2.20 (tt, *J* = 13.5, 6.8 Hz, 2H). ^**13**^ **C NMR** (CDCl_3_,151 MHz): δ 160.5, 160.4, 159.9, 149.2, 148.7, 135.2, 134.9, 123.5, 122.4 (t, *J* = 241.0 Hz), 96.6, 54.5, 43.0, 42.5, 31.8 (t, *J* = 28.2 Hz), 30.4 (t, *J* = 24.3 Hz), 29.5 (t, *J* = 5.4 Hz). ^**19**^ **F NMR** (CDCl_3_, 471 MHz): δ −95.0 (s, 2F). **HRMS** *m*/*z* [M + H]^+^ C_19_H_25_F_2_N_6_^+^ calculated 375.2103, found 375.2102.
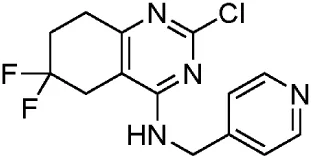


**2-Chloro-6,6-difluoro-** ***N*** **-(pyridin-4-ylmethyl)-5,6,7,8-tetrahydroquinazolin-4-amine (49).**Prepared according to general procedure L from dichloride (150 mg, 0.627 mmol, 1.0 equiv), pyridin-4-ylmethanamine (0.168 mL, 1.63 mmol, 2.6 equiv), DIPEA (0.230 mL, 1.31 mmol, 2.1 equiv) and MeCN (5 mL) to yield the title compound as a white solid solid (55 mg, 28%). ^**1**^ **H NMR** (CDCl_3_, 250 MHz): δ 8.55 (d, *J* = 5.9 Hz, 2H), 7.38–7.12 (m, 2H), 5.33–5.27 (m, 1H), 4.75 (d, *J* = 5.8 Hz, 2H), 3.02–2.80 (m, 4H), 2.39–2.08 (m, 2H).
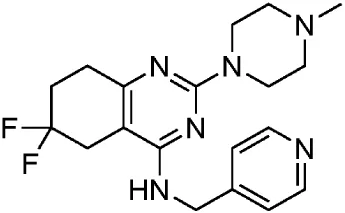


**6,6-Difluoro-2-(4-methylpiperazin-1-yl)-** ***N*** **-(pyridin-4-ylmethyl)-5,6,7,8-tetrahydroquinazolin-4-amine (65).**Prepared according to general procedure M from monochloride **49** (55 mg, 0.177 mmol, 1.0 equiv), *N*-methylpiperazine (0.098 mL, 0.885 mmol, 5.0 equiv), DIPEA (0.065 mL, 0.372 mmol, 2.1 equiv) and dioxane (5 mL). The crude product was purified using NP column chromatography (EtOAc:MeOH:TEA 90:5:5 in *c*Hex) to yield the title compound as a white solid (51 mg, 77%). **ULCMS** (acidic mode) R_t_ 0.429 min, LC purity >99% (254 nm), *m*/*z* [M + H]^+^ calculated 375.44, measured 375.20. ^**1**^ **H NMR** (CDCl_3_, 600 MHz): δ 8.57–8.49 (m, 2H), 7.24–7.19 (m, 2H), 4.80 (t, *J* = 5.8 Hz, 1H), 4.66 (d, *J* = 5.7 Hz, 2H), 3.98–3.63 (m, 4H), 2.87–2.77 (m, 4H), 2.59–2.46 (m, 4H), 2.41 (s, 3H), 2.22 (tt, *J* = 13.5, 6.8 Hz, 2H). ^**13**^ **C NMR** (CDCl_3_, 151 MHz): δ 160.6, 160.5, 159.8, 149.9, 148.6, 122.4 (t, *J* = 241.8 Hz), 122.1, 96.6, 54.4, 45.5, 43.9, 42.9, 31.8 (t, *J* = 28.3 Hz), 30.4 (t, *J* = 24.3 Hz), 29.5 (t, *J* = 5.5 Hz). ^**19**^ **F NMR** (CDCl_3_, 471 MHz): δ −95.0 (s, 2F). **HRMS** *m*/*z* [M + H]^+^ C_19_H_25_F_2_N_6_^+^ calculated 375.2103, found 375.2105.
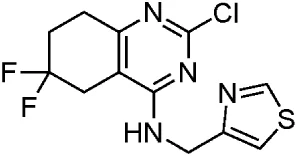


**2-Chloro-6,6-difluoro-** ***N*** **-(thiazol-4-ylmethyl)-5,6,7,8-tetrahydroquinazolin-4-amine (50).**Prepared according to general procedure L from dichloride **43** (2,4-dichloro-6,6-difluoro-5,6,7,8-tetrahydroquinazoline) (93 mg, 0.390 mmol, 1.0 equiv), **39a** (68 mg, 0.390 mmol, 1.0 equiv), DIPEA (0.341 mL, 1.95 mmol, 5.0 equiv) and MeCN (5 mL) to yield the title compound as a white solid (34 mg, 28%). ^**1**^ **H NMR** (CDCl_3_, 500 MHz): δ 8.84 (d, *J* = 2.0 Hz, 1H), 7.35 (d, *J* = 2.0 Hz, 1H), 5.83–5.56 (m, 1H), 4.83 (d, *J* = 5.1 Hz, 2H), 2.96 (t, *J* = 6.8 Hz, 2H), 2.86 (t, *J* = 13.8 Hz, 2H), 2.24 (tt, *J* = 13.4, 6.9 Hz, 2H).
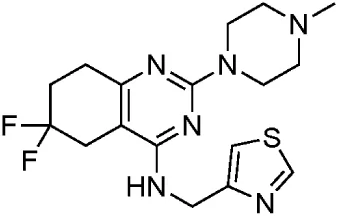


**6,6-Difluoro-2-(4-methylpiperazin-1-yl)-** ***N*** **-(thiazol-4-ylmethyl)-5,6,7,8-tetrahydroquinazolin-4-amine (66).**Prepared according to general procedure M from monochloride **50** (34 mg, 0.107 mmol, 1.0 equiv), *N*-methylpiperazine (0.059 mL, 0.535 mmol, 5.0 equiv), DIPEA (0.039 mL, 0.225 mmol, 2.1 equiv) and dioxane (2 mL). The crude product was purified using NP column chromatography (EtOAc:MeOH:TEA 90:5:5 in *c*Hex) to yield the title compound as a white solid (19 mg, 47%). **ULCMS** (acidic mode) R_t_ 1.066 min, LC purity >95% (254 nm), *m*/*z* [M + H]^+^ calculated 381.46, measured 381.15. ^**1**^ **H NMR** (CD_3_OD, 600 MHz): δ 8.94 (d, *J* = 2.0 Hz, 1H), 7.37–7.20 (m, 1H), 4.77 (d, *J* = 1.1 Hz, 2H), 3.78–3.63 (m, 4H), 2.88 (t, *J* = 14.6 Hz, 2H), 2.76 (t, *J* = 6.9 Hz, 2H), 2.54–2.48 (m, 4H), 2.37 (s, 3H), 2.22 (tt, *J* = 13.5, 6.8 Hz, 2H). ^**13**^ **C NMR** (CD_3_OD, 151 MHz): δ 160.9, 159.9, 159.1, 155.8, 153.7, 122.6 (t, *J* = 238.6 Hz), 114.3, 97.6 (t, *J* = 5.8 Hz), 54.1, 44.4, 42.9, 40.3, 31.3 (t, *J* = 28.2 Hz), 29.8 (t, *J* = 24.7 Hz), 28.7 (t, *J* = 5.6 Hz). ^**19**^ **F NMR** (CD_3_OD, 471 MHz): δ −97.1 (s, 2F). **HRMS** *m*/*z* [M + H]^+^ C_17_H_23_F_2_N_6_S^+^ calculated 381.1667, found 381.1670.
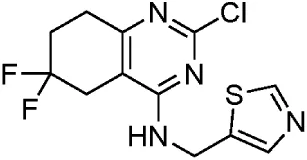


**2-Chloro-6,6-difluoro-** ***N*** **-(thiazol-5-ylmethyl)-5,6,7,8-tetrahydroquinazolin-4-amine (51).**Prepared according to general procedure L from dichloride **43** (103 mg, 0.431 mmol, 1.0 equiv), **39b** (49 mg, 0.431 mmol, 1.0 equiv), DIPEA (0.376 mL, 2.15 mmol, 5.0 equiv) and MeCN (5 mL) to yield the title compound as a white solid (22 mg, 16%). ^**1**^ **H NMR** (DMSO-d_6_, 500 MHz): δ 8.95 (s, 1H), 8.05 (t, *J* = 5.9 Hz, 1H), 7.84 (s, 1H), 4.74 (d, *J* = 5.7 Hz, 2H), 2.93 (t, *J* = 14.6 Hz, 2H), 2.79 (t, *J* = 6.9 Hz, 2H), 2.27 (tt, *J* = 13.9, 6.8 Hz, 2H).
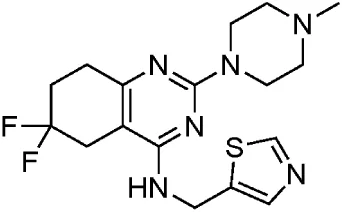


**6,6-Difluoro-2-(4-methylpiperazin-1-yl)-** ***N*** **-(thiazol-5-ylmethyl)-5,6,7,8-tetrahydroquinazolin-4-amine (67).**Prepared according to general procedure M from monochloride **51** (22 mg, 0.069 mmol, 1.0 equiv), *N*-methylpiperazine (0.038 mL, 1.11 mmol, 5.0 equiv), DIPEA (0.025 mL, 0.145 mmol, 2.1 equiv) and dioxane (2 mL). The crude product was purified using NP column chromatography (EtOAc:MeOH:TEA 90:5:5 in *c*Hex) to yield the title compound as a white solid (23 mg, 87%). **ULCMS** (acidic mode) R_t_ 1.044 min, LC purity >99% (254 nm), *m*/*z* [M + H]^+^ calculated 381.46, measured 381.55. ^**1**^ **H NMR** (CD_3_OD, 600 MHz): δ 8.83 (d, *J* = 0.8 Hz, 1H), 7.83–7.73 (m, 1H), 4.82 (d, *J* = 0.9 Hz, 2H), 3.81 (br, 4H), 2.83–2.72 (m, 4H), 2.53 (app t, *J* = 5.1 Hz, 4H), 2.37 (s, 3H), 2.20 (tt, *J* = 13.8, 7.1 Hz, 2H). ^**13**^ **C NMR** (CD_3_OD, 151 MHz): δ 160.6, 159.8, 159.6, 153.9, 140.3, 138.4, 122.5 (t, *J* = 239.4 Hz), 97.4, 54.4, 44.6, 43.1, 36.1, 31.3 (t, *J* = 239.0 Hz), 29.7 (t, *J* = 24.5 Hz), 28.8 (t, *J* = 5.5 Hz). ^**19**^ **F NMR** (CD_3_OD, 471 MHz): δ −97.2 (s, 2F). **HRMS** *m*/*z* [M + H]^+^ C_17_H_23_F_2_N_6_S^+^ calculated 381.1667, found 381.1670.
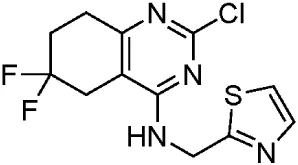


**2-Chloro-6,6-difluoro-** ***N*** **-(thiazol-2-ylmethyl)-5,6,7,8-tetrahydroquinazolin-4-amine (52).**Prepared according to general procedure L from dichloride **43** (103 mg, 0.431 mmol, 1.0 equiv), 2-aminomethylthiazole hydrochloride (65 mg, 0.431 mmol, 1.0 equiv), and DIPEA (0.367 mL, 2.15 mmol, 5.0 equiv) and MeCN (5 mL) to yield the title compound as a white solid (70 mg, 51%). ^**1**^ **H NMR** (CDCl_3_, 250 MHz): δ 7.75 (d, *J* = 3.3 Hz, 1H), 7.35 (d, *J* = 3.3 Hz, 1H), 6.00–5.90 (m, 1H), 5.01 (d, *J* = 5.1 Hz, 2H), 3.03–2.79 (m, 4H), 2.34–2.17 (m, 2H).
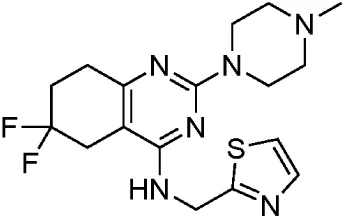


**6,6-Difluoro-2-(4-methylpiperazin-1-yl)-** ***N*** **-(thiazol-2-ylmethyl)-5,6,7,8-tetrahydroquinazolin-4-amine (68).**Prepared according to general procedure M from monochloride **52** (70 mg, 0.221 mmol, 1.0 equiv), *N*-methylpiperazine (0.123 mL, 1.11 mmol, 5.0 equiv), and DIPEA (0.081 mL, 0.464 mmol, 2.1 equiv) and dioxane (5 mL). The crude product was purified using NP column chromatography (EtOAc:MeOH:TEA 90:5:5 in *c*Hex) to yield the title compound as a white solid (11 mg, 13%). **ULCMS** (acidic mode) R_t_ 1.088 min, LC purity >99% (254 nm), *m*/*z* [M + H]^+^ calculated 381.46, measured 381.15. ^**1**^ **H NMR** (CDCl_3_, 600 MHz): δ 7.71 (d, *J* = 3.3 Hz, 1H), 7.27 (d, *J* = 3.3 Hz, 1H), 5.40–5.24 (m, 1H), 4.94 (d, *J* = 5.7 Hz, 2H), 4.15–3.78 (m, 4H), 2.83–2.64 (m, 8H), 2.54 (s, 3H), 2.20 (tt, *J* = 13.4, 6.7 Hz, 2H). ^**13**^ **C NMR** (CDCl_3_, 151 MHz): δ 168.4, 160.2, 142.1, 122.4 (t, *J* = 241.1 Hz), 119.4, 97.3, 54.2, 45.0, 42.5, 42.4, 31.7 (t, *J* = 28.2 Hz), 30.3 (t, *J* = 24.4 Hz), 29.5 (t, *J* = 4.9 Hz). Two signals missing. ^**19**^ **F NMR** (CDCl_3_, 471 MHz): δ −95.1 (s, 2F). **HRMS** *m*/*z* [M + H]^+^ C_17_H_23_F_2_N_6_S^+^ calculated 381.1667, found 381.1667.
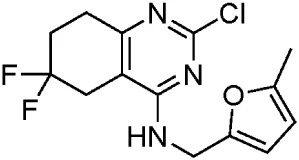


**2-Chloro-6,6-difluoro-** ***N*** **-((5-methylfuran-2-yl)­methyl)-5,6,7,8-tetrahydroquinazolin-4-amine (53).**Prepared according to general procedure L from dichloride **43** (150 mg, 0.627 mmol, 1.0 equiv), (5-methylfuran-2-yl)­methanamine (0.070 mL, 0.627 mmol, 1.0 equiv), DIPEA (0.231 mL, 1.32 mmol, 2.1 equiv) and MeCN (5 mL) to yield the title compound as a white solid (104 mg, 53%). **^1^H NMR** (CDCl_3_, 250 MHz): δ 6.21 (d, *J* = 3.0 Hz, 1H), 5.96–5.90 (m, 1H), 4.92–4.79 (m, 1H), 4.63 (d, *J* = 4.9 Hz, 2H), 2.94–2.86 (m, 2H), 2.85–2.75 (m, 2H), 2.40–2.20 (m, 5H).
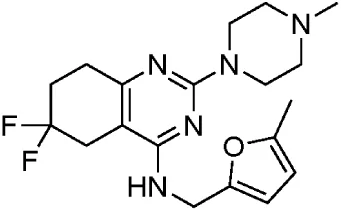


**6,6-Difluoro-** ***N*** **-((5-methylfuran-2-yl)­methyl)-2-(4-methylpiperazin-1-yl)-5,6,7,8-tetrahydroquinazolin-4-amine (69).**Prepared according to general procedure M from monochloride **53** (104 mg, 0.331 mmol, 1.0 equiv), *N*-methylpiperazine (0.184 mL, 1.66 mmol, 5.0 equiv), DIPEA (0.122 mL, 0.696 mmol, 2.1 equiv) and dioxane (5 mL) to yield the title compound as a white solid (110 mg, 88%). **ULCMS** (acidic mode) R_t_ 1.245 min, LC purity >97% (254 nm), *m*/*z* [M + H]^+^ calculated 378.44, measured 378.20. ^**1**^ **H NMR** (CDCl_3_, 600 MHz): δ 6.10 (d, *J* = 3.0 Hz, 1H), 5.90 (dq, *J* = 3.0, 1.0 Hz, 1H), 4.56 (d, *J* = 5.0 Hz, 2H), 4.54–4.50 (m, 1H), 4.09–3.81 (m, 4H), 2.80 (t, *J* = 6.9 Hz, 2H), 2.75 (t, *J* = 14.3 Hz, 2H), 2.71–2.61 (m, 4H), 2.48 (s, 3H), 2.28 (d, *J* = 0.6 Hz, 3H), 2.19 (tt, *J* = 13.5, 6.9 Hz, 2H). ^**13**^ **C NMR** (CDCl_3_, 151 MHz): δ 160.3, 160.1#, 151.9, 150.0, 122.5 (t, *J* = 240.5 Hz), 108.3, 106.3, 96.9, 54.4, 45.4, 42.8, 38.1, 31.7 (t, *J* = 28.2 Hz), 30.4 (t, *J* = 24.4 Hz), 29.4 (t, *J* = 5.6 Hz), 13.6. One signal missing. ^**19**^ **F NMR** (CDCl_3_, 471 MHz): δ −95.0 (s, 2F). **HRMS** *m*/*z* [M + H]^+^ C_19_H_26_F_2_N_5_O^+^ calculated 378.2100, found 378.2101.
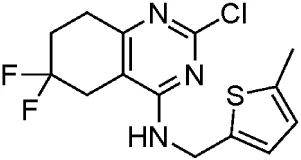


**2-Chloro-6,6-difluoro-** ***N*** **-((5-methylthiophen-2-yl)­methyl)-5,6,7,8-tetrahydroquinazolin-4-amine (54).**Prepared according to general procedure L from dichloride **43** (118 mg, 0.494 mmol, 1.0 equiv), (5-methylthiophen-2-yl)­methanamine (0.081 mg, 0.494 mmol, 1.0 equiv), DIPEA (0.431 mL, 2.47 mmol, 5 equiv) and MeCN (5 mL) to yield the title compound as a white solid (69 mg, 42%). ^**1**^ **H NMR** (CDCl_3_, 250 MHz): δ 8.83 (d, *J* = 3.3 Hz, 1H), 6.61 (d, *J* = 2.4 Hz, 1H), 4.91–4.80 (m, 1H), 4.77 (d, *J* = 4.9 Hz, 2H), 2.97 (t, *J* = 6.8 Hz, 2H), 2.77 (t, *J* = 13.8 Hz, 2H), 2.46 (s, 3H), 2.36–2.13 (m, 2H).
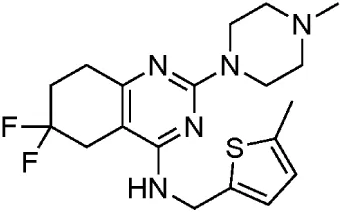


**6,6-Difluoro-2-(4-methylpiperazin-1-yl)-** ***N*** **-((5-methylthiophen-2-yl)­methyl)-5,6,7,8-tetrahydroquinazolin-4-amine (70).**Prepared according to general procedure M from monochloride **54** (69 mg, 0.209 mmol, 1.0 equiv), *N*-methylpiperazine (0.116 mL, 1.05 mmol, 5.0 equiv), DIPEA (0.077 mL, 0.439 mmol, 2.1 equiv) and dioxane (5 mL) to yield the title compound as a white solid (70 mg, 85%). **ULCMS** (acidic mode) R_t_ 1.293 min, LC purity >97% (254 nm), *m*/*z* [M + H]^+^ calculated 394.50, measured 394.15. ^**1**^ **H NMR** (CDCl_3_, 600 MHz): δ 6.75 (d, *J* = 3.4 Hz, 1H), 6.58 (dq, *J* = 3.4, 1.1 Hz, 1H), 4.76–4.62 (m, 3H), 4.28–3.95 (m, 4H), 2.94–2.77 (m, 6H), 2.73 (t, *J* = 14.2 Hz, 2H), 2.60 (s, 3H), 2.43 (d, *J* = 1.1 Hz, 3H), 2.19 (tt, *J* = 13.5, 6.8 Hz, 2H). ^**13**^ **C NMR** (CDCl_3_, 151 MHz): δ 160.3, 160.2#, 139.9, 139.3, 125.8, 124.6, 122.4 (t, *J* = 241.1 Hz), 97.3, 54.0, 44.7, 42.2, 40.1, 31.7 (t, *J* = 28.3 Hz), 30.3 (t, *J* = 24.5 Hz), 29.4, 15.3. One signal missing. ^**19**^ **F NMR** (CDCl_3_, 471 MHz): δ −95.1 (s, 2F). **HRMS** *m*/*z* [M + H]^+^ C_19_H_26_F_2_N_5_S^+^ calculated 394.1871, found 394.1868.
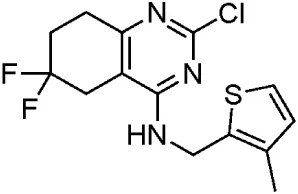


**2-Chloro-6,6-difluoro-** ***N*** **-((3-methylthiophen-2-yl)­methyl)-5,6,7,8-tetrahydroquinazolin-4-amine (55).**Prepared according to general procedure L from dichloride **43** (150 mg, 0.627 mmol, 1.0 equiv),), (3-methylthiophen-2-yl)­methanamine (0.080 mg, 0.627 mmol, 1.0 equiv), DIPEA (0.230 mL, 1.32 mmol, 2.1 equiv) and MeCN (5 mL) to yield the title compound as a white solid (119 mg, 58%). ^**1**^ **H NMR** (CDCl_3_, 250 MHz): δ 7.18 (d, *J* = 5.1 Hz, 1H), 6.85 (d, *J* = 5.1 Hz, 1H), 4.80–4.70 (m, 3H), 3.00–2.70 (m, 4H), 2.27–2.15 (m, 5H).
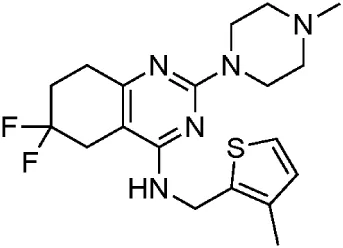


**6,6-Difluoro-2-(4-methylpiperazin-1-yl)-** ***N*** **-((3-methylthiophen-2-yl)­methyl)-5,6,7,8-tetrahydroquinazolin-4-amine (71).**Prepared according to general procedure M from monochloride **55** (119 mg, 0.361 mmol, 1.0 equiv), *N*-methylpiperazine (0.200 mL, 1.80 mmol, 5.0 equiv), DIPEA (0.132 mL, 0.758 mmol, 2.1 equiv) and dioxane (5 mL). The crude product was purified using NP column chromatography (EtOAc:MeOH:TEA 90:5:5 in *c*Hex) to yield the title compound as a white solid (86 mg, 61%). **ULCMS** (acidic mode) R_t_ 1.330 min, LC purity >99% (254 nm), *m*/*z* [M + H]^+^ calculated 394.50, measured 394.15. ^**1**^ **H NMR** (CDCl_3_, 600 MHz): δ 7.12 (d, *J* = 5.1 Hz, 1H), 6.82 (d, *J* = 5.1 Hz, 1H), 4.72 (d, *J* = 5.2 Hz, 2H), 4.54–4.42 (m, 1H), 4.10–3.84 (m, 4H), 2.80 (t, *J* = 6.8 Hz, 2H), 2.76–2.64 (m, 6H), 2.50 (s, 3H), 2.25 (s, 3H), 2.23–2.15 (m, 2H). ^**13**^ **C NMR** (CDCl_3_, 151 MHz): δ 160.3, 160.2#, 134.8, 134.5, 130.1, 123.4, 122.5 (t, *J* = 241.2 Hz), 96.9, 54.4, 45.3, 42.8, 37.8, 31.7 (t, *J* = 28.1 Hz), 30.4 (t, *J* = 24.4 Hz), 29.5 (t, *J* = 5.1 Hz), 13.7. One signal missing. ^**19**^ **F NMR** (CDCl_3_, 471 MHz): δ −95.0 (s, 2F). **HRMS** *m*/*z* [M + H]^+^ C_19_H_26_F_2_N_5_S^+^ calculated 394.1871, found 394.1875.
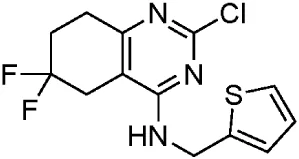


**2-Chloro-6,6-difluoro-** ***N*** **-(thiophen-2-ylmethyl)-5,6,7,8-tetrahydroquinazolin-4-amine (56).**Prepared according to general procedure L in three batches, with each batch using dichloride **43** (4.00 g, 16.7 mmol, 1.0 equiv), thiophen-2-ylmethanamine (1.72 mL, 16.7 mmol, 1.0 equiv), DIPEA (6.43 mL, 36.7 mmol, 2.2 equiv) and MeCN (15 mL). The three batches were combined and diluted with DCM (150 mL). The resulting suspension was washed with H_2_O (100 mL). The organic phase was dried over Na_2_SO_4_ and concentrated to yield a yellow oil, that was redissolved in EtOAc (2 mL). Dropwise addition of heptane to this caused the formation of a white precipitate that was filtered to yield the title compound as a white solid (10.6 g, extrapolated yield 67%). ^**1**^ **H NMR** (CDCl_3_, 500 MHz): δ 7.26 (m, 1H), 7.06 (dd, *J* = 3.5, 1.1 Hz, 1H), 6.98 (dd, *J* = 5.1, 3.5 Hz, 1H), 5.05–4.94 (m, 1H), 4.87 (d, *J* = 5.4 Hz, 2H), 2.97 (t, *J* = 6.9 Hz, 2H), 2.79 (t, *J* = 13.8 Hz, 2H), 2.24 (tt, *J* = 13.5, 6.9 Hz, 2H).
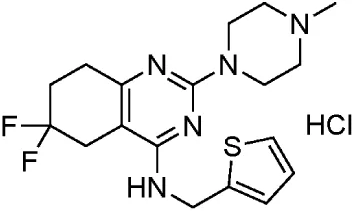


**6,6-Difluoro-2-(4-methylpiperazin-1-yl)-** ***N*** **-(thiophen-2-ylmethyl)-5,6,7,8-tetrahydroquinazolin-4-amine hydrochloride (72.HCl, GD134).** Prepared according to general procedure M from monochloride **56** (8.0 g, 25.3 mmol, 1.0 equiv), *N*-methylpiperazine (7.61 mL, 68.4 mmol, 2.7 equiv), DIPEA (12.0 mL, 68.4 mmol, 2.7 equiv) and dioxane (60 mL). The reaction mixture was diluted with EtOAc (250 mL) and washed with 1 M aq. NaOH (250 mL). After drying the organic phase over Na_2_SO_4_, the filtrate was concentrated and purified using NP column chromatography (EtOAc:MeOH:TEA 90:5:5 in *c*Hex). The product was converted to its corresponding HCl salt using 4 N HCl in dioxane (4.48 mL, 17.9 mmol, 1.0 equiv) to yield the title compound as a white solid (5.0 g, 48%).

Free base: **ULCMS** (acidic mode) R_t_ 1.201 min, LC purity >99% (254 nm), *m*/*z* [M + H]^+^ calculated 380.47, measured 380.10. ^**1**^ **H NMR** (CDCl_3_, 600 MHz): δ 7.21 (dd, *J* = 5.1, 1.3 Hz, 1H), 7.01–6.98 (m, 1H), 6.95 (dd, *J* = 5.1, 3.4 Hz, 1H), 4.82 (d, *J* = 5.7 Hz, 2H), 4.59 (t, *J* = 5.7 Hz, 1H), 3.96–3.77 (m, 4H), 2.80 (t, *J* = 6.8 Hz, 2H), 2.72 (t, *J* = 14.3 Hz, 2H), 2.54–2.43 (m, 4H), 2.35 (s, 3H), 2.25–2.16 (m, 2H). ^**13**^ **C NMR** (CDCl_3_, 151 MHz): δ 160.24, 160.17, 160.1, 142.1, 126.6, 125.8, 125.2, 122.6 (t, *J* = 240.8 Hz), 96.3 (t, *J* = 5.6 Hz), 55.0, 46.2, 43.6, 39.7, 31.7 (t, *J* = 28.2 Hz), 30.4 (t, *J* = 24.4 Hz), 29.5 (t, *J* = 5.5 Hz). ^**19**^ **F NMR** (CDCl_3_, 471 MHz): δ −95.0 (s, 2F). **HRMS** *m*/*z* [M + H]^+^ C_18_H_24_F_2_N_5_S^+^ calculated 380.1715, found 380.1716. Regiochemistry was proven in-depth by extensive 2D NMR analysis (see Supporting Information page S31)

HCl salt: **ULCMS** (acidic mode) R_t_ 1.221 min, LC purity >99% (254 nm), *m*/*z* [M + H]^+^ calculated 380.47, measured 380.20. **HRMS** *m*/*z* [M + H]^+^ C_18_H_24_F_2_N_5_S^+^ calculated 380.1715, found 380.1718.

^**1**^ **H NMR** (CD_3_OD, 600 MHz): δ 7.30 (dd, *J* = 5.1, 1.2 Hz, 1H), 7.08 (dd, *J* = 3.5, 1.2 Hz, 1H), 6.96 (dd, *J* = 5.1, 3.5 Hz, 1H), 4.90 (s, 2H), 3.01–2.96 (m, 5H), 2.92 (t, *J* = 13.9 Hz, 2H), 2.33 (tt, *J* = 13.4, 6.8 Hz, 2H). Two signals are not unambiguously evident (piperazine CH_2_). ^**13**^ **C NMR** (CD_3_OD, 151 MHz): δ 160.8, 140.2, 126.3, 126.2, 124.9, 121.2 (t, *J* = 240.2 Hz), 101.5, 42.2, 39.6, 30.5 (t, *J* = 29.4 Hz), 28.2 (t, *J* = 25.3 Hz), 24.5. Four signals missing. ^**19**^ **F NMR** (CD_3_OD, 471 MHz): δ −97.7 (s, 2F).

^**1**^ **H NMR** (DMSO-d_6_, 600 MHz): δ 11.73–11.48 (m, 1H) 9.18–9.03 (m, 1H), 7.40 (dd, *J* = 5.1, 1.3 Hz, 1H), 7.14–7.07 (m, 1H), 6.97 (dd, *J* = 5.1, 3.4 Hz, 1H), 4.90–4.73 (m, 4H), 3.62–3.54 (m, 4H), 3.17–3.06 (m, 2H), 3.03–2.89 (m, 4H), 2.81–2.72 (m, 3H), 2.29 (tt, *J* = 13.8, 6.7 Hz, 2H). ^**13**^ **C NMR** (DMSO-d_6_, 151 MHz): δ 160.3, 149.8*, 141.1, 127.2, 127.1, 126.1, 122.9 (t, *J* = 239.4 Hz), 100.9, 51.9, 42.5, 42.3, 39.7#, 31.4 (t, *J* = 28.5 Hz), 28.5 (t, *J* = 24.4 Hz), 25.3. One signal missing. ^**19**^ **F NMR** (DMSO-d_6_, 471 MHz): δ −94.5 (s, 2F).
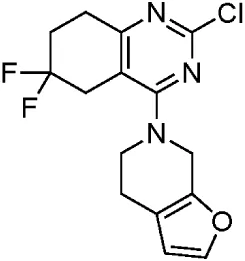


**6-(2-Chloro-6,6-difluoro-5,6,7,8-tetrahydroquinazolin-4-yl)-4,5,6,7-tetrahydrofuro­[2,3-** ***c*** **]­pyridine (57).**Prepared according to general procedure N from amine **37a** (556 mg, 5.00 mmol, 1.0 equiv), CH_2_O (37% aq. solution, 0.451 mL, 6.00 mmol, 1.2 equiv) and DCM (10 mL). The reaction mixture was diluted with DCM and washed with sat. aq. NaHCO_3_. The crude bicyclic intermediate (105 mg, 0.853 mmol, 1.2 equiv), dichloride **43** (169 mg, 0.707 mmol, 1.0 equiv), and DIPEA (0.259 mL, 1.49 mmol, 1.5 equiv) were stirred in MeCN (5 mL) to yield the title compound as a white solid (57 mg, 3.5%). ^**1**^ **H NMR** (CDCl_3_, 250 MHz): δ 7.32 (d, *J* = 0.9 Hz, 1H), 6.29 (d, *J* = 1.7 Hz, 1H), 4.49 (s, 2H), 3.61 (t, *J* = 5.5 Hz, 2H), 3.09–3.02 (m, 4H), 2.74 (t, *J* = 5.4 Hz, 2H), 2.46–2.14 (m, 2H).
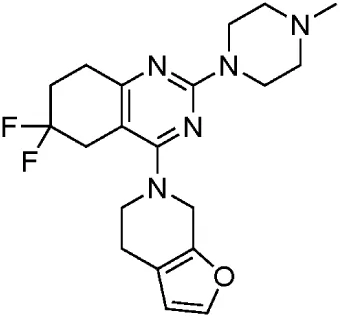


**6-(6,6-Difluoro-2-(4-methylpiperazin-1-yl)-5,6,7,8-tetrahydroquinazolin-4-yl)-4,5,6,7-tetrahydrofuro­[2,3-** ***c*** **]­pyridine (73).**Prepared according to general procedure M from monochloride **57** (57 mg, 0.175 mmol, 1.0 equiv), *N*-methylpiperazine (0.097 mL, 0.875 mmol, 5.0 equiv), DIPEA (0.064 mL, 0.367 mmol, 2.1 equiv) and dioxane (5 mL). The crude product was purified using NP column chromatography (EtOAc:MeOH:TEA 90:5:5 in *c*Hex) to yield the title compound as a white solid (21 mg, 31%). **ULCMS** (acidic mode) R_t_ 1.452 min, LC purity >95% (254 nm), *m*/*z* [M + H]^+^ calculated 390.45, measured 390.55. ^**1**^ **H NMR** (CDCl_3_, 600 MHz): δ 7.30 (d, *J* = 1.8 Hz, 1H), 6.28 (d, *J* = 1.9 Hz, 1H), 4.36 (s, 2H), 4.09 – 3.84 (m, 4H), 3.49 – 3.44 (m, 2H), 3.01 (t, *J* = 13.6 Hz, 2H), 2.88 (t, *J* = 7.1 Hz, 2H), 2.80 – 2.71 (m, 4H), 2.69 – 2.66 (m, 2H), 2.53 (s, 3H), 2.25 (tt, *J* = 13.6, 7.1 Hz, 2H). **^13^C NMR** (151 MHz, CDCl_3_) δ 166.2, 163.2, 159.4, 147.7, 141.3, 122.4 (t, *J* = 240.9 Hz), 115.5, 110.1, 101.6^#^, 54.3, 46.8, 45.9, 45.1, 42.5, 34.4 (t, *J* = 27.7 Hz), 30.0 (t, *J* = 24.3 Hz), 29.9 (t, *J* = 5.4 Hz), 22.8. ^**19**^ **F NMR** (471 MHz, CDCl_3_): δ −97.6 (s, 2F). **HRMS** *m*/*z* [M + H]^+^ C_20_H_26_F_2_N_5_O^+^ calculated 390.2100, found 390.2101.
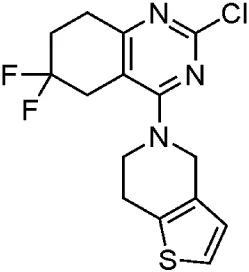


**5-(2-Chloro-6,6-difluoro-5,6,7,8-tetrahydroquinazolin-4-yl)-4,5,6,7-tetrahydrothieno­[3,2-** ***c*** **]­pyridine (58).**Prepared according to general procedure N from commercially available 2-(thiophen-2-yl)­ethan-1-amine (0.390 mL, 3.33 mmol, 1.0 equiv), CH_2_O (paraformaldehyde, 120 mg, 4.00 mmol, 1.2 equiv) and DCE (10 mL). The reaction mixture was diluted with 1 M aq. NaOH and extracted with DCM. The crude bicyclic intermediate (0.126 mg, 0.905 mmol, 1.4 equiv), dichloride **43** (151 mg, 0.632 mmol, 1.0 equiv), and DIPEA (0.232 mL, 1.33 mmol, 2.1 equiv) were stirred in MeCN (5 mL) to yield the title compound as a white solid (87 mg, 40%). ^**1**^ **H NMR** (CDCl_3_, 250 MHz): δ 7.16 (d, *J* = 5.2 Hz, 1H), 6.81 (d, *J* = 5.2 Hz, 1H), 4.56 (s, 2H), 3.73 (t, *J* = 5.6 Hz, 2H), 3.19–2.98 (m, 6H), 2.43–2.18 (m, 2H).
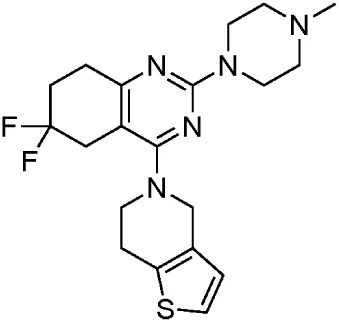


**5-(6,6-Difluoro-2-(4-methylpiperazin-1-yl)-5,6,7,8-tetrahydroquinazolin-4-yl)-4,5,6,7-tetrahydrothieno­[3,2-** ***c*** **]­pyridine (74).**Prepared according to general procedure M from monochloride **58** (87 mg, 0.255 mmol, 1.0 equiv), *N*-methylpiperazine (0.141 mL, 1.27 mmol, 5.0 equiv), DIPEA (0.093 mL, 0.535 mmol, 2.1 equiv) and dioxane (5 mL). The crude product was purified using NP column chromatography (EtOAc:MeOH:TEA 90:5:5 in *c*Hex) to yield the title compound as a white solid (37 mg, 36%). **ULCMS** (acidic mode) R_t_ 1.491 min, LC purity >97% (254 nm), *m*/*z* [M + H]^+^ calculated 406.51, measured 406.10. ^**1**^ **H NMR** (CDCl_3_, 600 MHz): δ 7.13 (d, *J* = 5.1 Hz, 1H), 6.81 (d, *J* = 5.1 Hz, 1H), 4.41 (s, 2H), 4.14–3.86 (m, 4H), 3.59 (t, *J* = 5.6 Hz, 2H), 3.06–2.99 (m, 4H), 2.88 (t, *J* = 7.1 Hz, 2H), 2.84–2.71 (m, 4H), 2.56 (s, 3H), 2.25 (tt, *J* = 13.7, 7.1 Hz, 2H). ^**13**^ **C NMR** (CDCl_3_, 151 MHz): δ 166.3, 163.2, 133.4, 133.0, 125.0, 123.2, 122.4 (t, *J* = 240.9 Hz), 102.0, 54.2, 48.2, 46.5, 42.4, 34.5 (t, *J* = 27.7 Hz), 30.0 (t, *J* = 24.1 Hz), 29.9 (t, *J* = 5.2 Hz), 25.4. One signal missing. ^**19**^ **F NMR** (CDCl_3_, 471 MHz): δ −97.6 (s, 2F). **HRMS** *m*/*z* [M + H]^+^ C_20_H_26_F_2_N_5_S^+^ calculated 406.1871, found 406.1869.
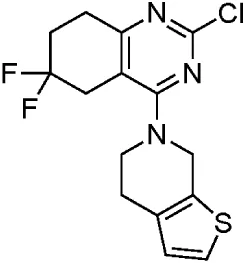


**6-(2-Chloro-6,6-difluoro-5,6,7,8-tetrahydroquinazolin-4-yl)-4,5,6,7-tetrahydrothieno­[2,3-** ***c*** **]­pyridine (59).**Prepared according to general procedure N from amine **37b** (455 mg, 3.58 mmol, 1.0 equiv), CH_2_O (37% aq. solution, 0.269 mL, 3.58 mmol, 1.2 equiv) and DCM (10 mL). The reaction mixture was diluted with 1 M aq. NaOH and extracted with DCM. The crude bicyclic intermediate (156 mg, 1.12 mmol, 1.2 equiv), **43** (217 mg, 0.908 mmol, 1.0 equiv), and DIPEA (0.333 mL, 1.91 mmol, 2.1 equiv) were stirred in MeCN (5 mL) to yield the title compound as a white solid (194 mg, 16%). ^**1**^ **H NMR** (CDCl_3_, 250 MHz): δ 7.18 (d, *J* = 5.0 Hz, 1H), 6.84 (d, *J* = 5.1 Hz, 1H), 4.69 (s, 2H), 3.67 (t, *J* = 5.7 Hz, 2H), 3.16–3.03 (m, 4H), 2.95 (t, *J* = 5.6 Hz, 2H), 2.41–2.20 (m, 2H).
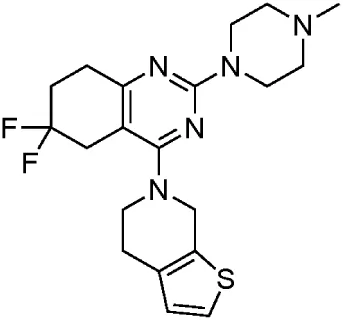


**6-(6,6-Difluoro-2-(4-methylpiperazin-1-yl)-5,6,7,8-tetrahydroquinazolin-4-yl)-4,5,6,7-tetrahydrothieno­[2,3-** ***c*** **]­pyridine (75).** Prepared according to general procedure M from monochloride **59** (184 mg, 0.538 mmol, 1.0 equiv), *N*-methylpiperazine (0.299 mL, 2.69 mmol, 5.0 equiv), DIPEA (0.197 mL, 1.13 mmol, 2.1 equiv) and dioxane (5 mL). The crude product was purified using NP column chromatography (EtOAc:MeOH:TEA 90:5:5 in *c*Hex) to yield the title compound as a white solid (183 mg, 84%). **ULCMS** (acidic mode) R_t_ 1.510 min, LC purity >97% (254 nm), *m*/*z* [M + H]^+^ calculated 406.51, measured 406.20. ^**1**^ **H NMR** (CDCl_3_, 600 MHz): δ 7.14 (d, *J* = 5.1 Hz, 1H), 6.82 (d, *J* = 5.0 Hz, 1H), 4.54 (s, 2H), 4.14–3.83 (m, 4H), 3.54 (t, *J* = 5.7 Hz, 2H), 3.03 (t, *J* = 13.6 Hz, 2H), 2.92–2.84 (m, 4H), 2.82–2.64 (m, 4H), 2.54 (s, 3H), 2.25 (tt, *J* = 13.8, 7.1 Hz, 2H). ^**13**^ **C NMR** (CDCl_3_, 151 MHz): δ 166.1, 163.2, 159.3, 133.7, 132.7, 127.0, 122.7, 122.4 (t, *J* = 240.5 Hz), 102.0, 54.3, 47.3, 46.4, 45.1, 42.5, 34.4 (t, *J* = 27.7 Hz), 30.0 (t, *J* = 24.5 Hz), 29.9 (t, *J* = 5.4 Hz), 26.0. ^**19**^ **F NMR** (CDCl_3_, 471 MHz): δ −97.6 (s, 2F). **HRMS** *m*/*z* [M + H]^+^ C_20_H_26_F_2_N_5_S^+^ calculated 406.1871, found 406.1875.

### Molecular Docking

The X-ray structure of doxepin-bound H_1_R (PDB: 3RZE)^53^ and the cryo-EM structure of adriforant-bound H_4_R (PDB: 9JG1)^54^ were prepared using the Structure Preparation module in MOE v2024.06. Hydrogen atoms were added, and partial charges were calculated using the Amber:EHT force field. Compounds **35** and **72** were converted from their SMILES representation into 3D structures using MOE. Each compound was visually inspected and modified to ensure the correct isomeric form. During this process, the protonation state was defined such that the *N*-methylpiperazine nitrogen was protonated, yielding a positively charged species. Compounds **35** and **72** were docked into the prepared receptors using MOE’s docking module. To facilitate proper ligand placement and guide the docking process, a pharmacophoric constraint consisting of a cation and hydrogen bond donor (Cat&Don) was placed near D^3.32^. Ligand placement was performed using the Triangle Matcher method, in which poses were scored using London dG, and the top 50 were retained. The refinement process was performed using the Induced Fit method, where all poses were scored using the GBVI/WSA dG score. The best-scoring pose of each compound showing an ionic interaction to D^3.32^ was selected for visualization. Docking scores of **35** in H_1_R ranged from −10.3 to −8.3 kcal/mol, and in H_4_R ranged from −9.9 to −8.5 kcal/mol. Docking scores of **72** in H_1_R ranged from −9.3 to −7.8 kcal/mol, and in H_4_R ranged from −8.7 to −7.2 kcal/mol.

### Molecular Dynamics Simulations

Missing regions in the receptor structures were modeled prior to molecular dynamics simulations. For H_1_R, the unresolved portion of ECL2 was constructed in MOE using the Loop Modeler (de novo protocol, 1000 backbone conformations), and the conformation with the lowest S-score was selected. For both H_1_R and H_4_R, the unresolved ICL3 loop was replaced with a six-glycine linker connecting TM5 and TM6, generated using the same Loop Modeler de novo protocol. A sodium ion was positioned into the sodium binding pocket based on the sodium-bound β_1_-adrenoreceptor crystal structure (PDB: 4BVN).^78^

Protein–ligand complexes were prepared using the CHARMM-GUI web server.^79^ The N-terminus was acetylated and the C-terminus amidated. Each complex was embedded in a box of approximately 90 × 90 × 120 Å containing a POPC bilayer, water molecules, and 150 mM concentration of NaCl. Systems were parametrized using the ff19SB protein force field, Lipid21 lipid force field, GAFF2 ligand force field, and the TIP3P water model. All simulations were performed using GROMACS 2025.3 ^80^ Energy minimization was carried out using 5000 steps of steepest descent, followed by equilibration using the default CHARMM-GUI multistage protocol with progressively released positional restraints. Production simulations were conducted in the NPT ensemble for 100 ns with a 2 fs time step. Covalent bonds involving hydrogens were constrained using the LINCS algorithm. Temperature was maintained at 310.15 K using the V-rescale thermostat (τt = 1.0 ps). Pressure was controlled at 1 bar using the C-rescale barostat with semi-isotropic coupling (τp = 5.0 ps, compressibility = 4.5 × 10^–5^ bar). Long-range electrostatics were treated using the particle-mesh Ewald (PME) method with a real-space cutoff of 0.9 nm, Short-range van der Waals interactions employed a 0.9 nm cutoff with analytical dispersion corrections applied to energy and pressure. Atomic positions were saved every 100 ps.

Ligand stability during MD simulation was monitored by root-mean-square deviation (RMSD) of ligand heavy atoms relative to the first frame after least-squares alignment of each trajectory frame to the protein backbone. Protein–ligand and water-mediated interactions were analyzed for each frame of the MD trajectories using ProLIF (version 2.1.0),^81^ generating binary interaction fingerprints that encode the presence or absence of specific contacts between the ligand and protein residues. Pairwise similarity between frames was computed using the Tanimoto coefficient applied to the interaction fingerprints. A full similarity matrix was constructed and converted to a distance matrix (1 – Tanimoto similarity), which was subjected to hierarchical clustering using average linkage and a distance cutoff of 0.4. The highest populated cluster was used to find the medoid frame to yield a representative binding pose for structural visualization and for figure making. The figures of the ligand–receptor complexes were generated in PyMOL (The PyMOL Molecular Graphics System, Version v3.1.6.1 Schrödinger, LLC).

### Pharmacology

Cell culture and transient transfection of HEK 293T cells were performed as described previously.^82^ Homogenates of transfected HEK 293T cells transiently transfected with various histamine receptor subtypes were used for radioligand binding assays. Tritium-labeled mepyramine, tiotidine *N* ^α^-methylhistamine, and histamine (all obtained from Revvity, The Netherlands) were used for radioligand binding assay of histamine receptor H_1_R, H_2_R, H_3_R, and H_4_R, respectively. Buffer used for H_1_R or H_2_R radioligand binding assay contains 50 mM KH_2_PO_4_/Na_2_HPO_4_ (pH 7.4), while buffer H_3_R or H_4_R radioligand binding assay contains 50 mM Tris-HCl (pH 7.4). The buffer for H_2_R binding assay is supplemented with 0.1% bovine serum albumin to reduce nonspecific radioligand binding. The reactions of all radioligand binding assays took place in 96-well plates in a total volume of 200 μL, incubated for 1 h at room temperature (22 °C), and harvested on UniFilter-96 GF/C microplates (Revvity) that were pretreated with 0.5% 750 kDa PEI. The radioactivity of bound radioligands was quantified in a MicroBeta^2^ microplate counter (Revvity) after addition of MicroScint-0 (Revvity). The data were analyzed with Prism (GraphPad Software Inc., USA) using one site—Fit *K*_i_ competitive binding model.

CHO-K1 stably transfected with a CRE-luciferase reporter gene were grown in DMEM/F-12 medium supplemented with 5% bovine fetal serum and 100 U/mL penicillin/streptomycin (Life Technologies, CA) and used to transiently express the human and mouse H_1_Rs or stably express the human and mouse H_4_Rs under hygromycin B selection. The activities of H_1_Rs were measured as the increases of luciferase activity, while those of H_4_Rs were assessed as the inhibition of luciferase activity induced by 1 μM forskolin. For antagonism study, a single concentration of histamine was challenged with different concentrations of antagonists, incubated for 5 h before the cells were lysed for the measurement of luminescence emitted by the firefly luciferase. The experimental results were analyzed using the variable slope model log­(agonist) or log­(inhibitor) vs response equations using GraphPad Prism version 10.0.0 for Windows (GraphPad Software, Boston, Massachusetts, USA). Inhibition constant (K_b_) values of antagonists for H_4_Rs were determined using the Cheng-Prusoff equation, while K_b_ values for H_1_Rs were determined using a modified Cheng-Prusoff equation (K_b_= IC_50_/(1+([agonist]/EC_50_)^K^)),^83^ with K is the Hill-slope of histamine (0.53 and 0.49 for human and mouse H_1_R, respectively).

### LogD_7.4_ Study

The LogD_7.4_ was measured by Cerep (now Eurofins, Poitiers, France; Study number 100009395) using a shake-flask method. The compound was incubated in a biphasic system (1-octanol and PBS pH 7.4) under constant shaking. After equilibration, samples from both phases are collected and analyzed using HPLC. The distribution of **35.HCl** and **72.HCl** between the PBS at pH_7.4_ and 1-octanol (LogD_7.4_) was determined as a measure of its lipophilicity.

### Stability Study

A preliminary assessment of the stability of **35.HCl** and **72.HCl** in an aqueous solution was performed by dissolving 1.0 mg in 1.0 mL H_2_O, which equates to a solution of 1.8 mM. The stability over time was determined by HPLC analysis.

### Thermodynamic Solubility

The thermodynamic solubility was measured by Cerep (now Eurofins, Poitiers, France; Study number 100009395). Compounds **35.HCl** and **72.HCl** were each added to a buffered solution (PBS, pH = 7.4) and subsequently mixed for 24 h at RT to ensure formation of an equilibrium. After the incubation period, the saturated solution was filtered and the aqueous solubility (μM) was determined by comparing the peak area of the peak in the calibration standard (200 μM in organic solvent, MeOH:H_2_O 60/40 v/v) with the peak area of the corresponding peak in the buffered sample.

### Forced Degradation Study

The forced degradation study was conducted by SEPS Pharma (now Eurofins; project number P3001). The stability of **35.HCl** was evaluated under neutral (Milli-Q water), acidic (0.1 M aq. HCl), and basic (0.1 M aq. NaOH) conditions for up to 7 days at ambient temperature and 60 °C. Photostability was assessed by exposing **35.HCl** to light both as a solution and as a solid powder for 7 h at 300 and 700 W, as well as under covered (dark) conditions. Thermal and humidity stress studies were performed by incubating a solution of **35.HCl** at 80 °C for 7 days and at 100 °C for 15 min. The solid form of **35.HCl** was incubated at 80 °C and at 80 °C/75% relative humidity for 7 days. Oxidative stability was evaluated by incubating **35.HCl** for 7 days in 0.3% H_2_O_2_ with 10% aq. MeOH under both light and dark conditions. Additional oxidative conditions included incubation with excess Fe­(III)­Cl_3_·6H_2_O in 10% aq. MeOH at 40 °C under dark conditions for 7 days, and incubation in 5 mM 2,2′-azobis­(2-amidinopropane) dihydrochloride with 0.05% aq. MeOH. For experiments conducted over 7 days, samples were analyzed on days 1, 3, 4, and 7. Area percentages were determined by HPLC analysis with UV detection at 230 nm.

### *In Vitro* ADMET Study

Microsomal stability, Caco-2, protein binding, and the Ames fluctuation assays were conducted by Cerep (now Eurofins, Poitiers, France; study number 100009395).

### Microsomal Stability

Microsomal stability was assessed by incubating compound **35.HCl** and **72.HCl** (both 100 nM) with liver microsomes from in human, rat, and mouse and are presented as half-life (minutes). The intrinsic clearance (CL_int_; μL/min/mg). CL_int_ was calculated with the following formula.

### Caco-2 Permeability

Caco-2 cell monolayers were used to evaluate intestinal permeability. Apparent permeability (P_app) and recovery were calculated. Low recovery (∼28% and ∼55% for **35.HCl** and **72.HCl**, respectively) was attributed to compound binding, metabolism, or intracellular accumulation.

### Safety Panel Screening of **72** (GD134)

The off-target panel for **72.HCl** (GD134) was conducted by ICE Bioscience (Beijing, China, study number VRI-01–20260414) according to standard protocols.

### hERG Binding Assay

hERG channel binding affinity was determined using [^3^H]­astemizole in a radioligand displacement assay using HEK293 cells expressing hERG channels, following the procedure described by Chiu et al.^71^

### Pharmacokinetic Study

The pharmacokinetic profile of **35.HCl** and **72.HCl** following intravenous (IV) and oral (PO) administration in male CD1 mice was conducted by ChemPartner. After IV/PO dosing, blood was collected at 0.083, 0.25, 0.5, 1, 2, 4, 8, 12, and 24 h, blood samples were collected for LC-MS/MS analysis to quantify the different **35.HCl** and **72.HCl** concentrations, enabling the calculation of the relevant pharmacokinetic parameters. All procedures were performed in accordance with the Ethical Guidelines on the Protection of Animals Used for Scientific Purposes at the DMPK Group at Shanghai ChemPartner Co., Ltd. study number: CPB-P13-7926M02.

### Ames Fluctuation Assay

The Ames test was conducted using 384-well plates and four Salmonella strains (TA98, TA100, TA1535, and TA1537). No significant increase for either compound in revertant colonies was observed.

### *In Vivo* CAC Model

Female Balb/C mice were sensitized on Day 0 by subcutaneous injection of short ragweed (SRW) mixed in aluminum hydroxide (0.1 mg SRW/0.65 mg Al­(OH)_3_) in both hind hocks and were left alone for the next 16 days. On Day 17, they were topically challenged with SRW and randomized into groups based on their hyperemia response. On Days 18–19, animals received topical doses of the respective test compounds once daily and four times daily on day 20. Mice were dosed topically to the cornea with a 3 μL drop of treatment in each eye, using a calibrated micropipette. Days 21–24 were repeat challenges in which mice received twice-daily (total of eight challenges over 4 days) topical SRW challenges and three-times-daily treatment with their respective test compound. The repeat challenges were performed to enhance the challenge reaction to a more robust inflammatory reaction, as has been shown clinically with modified Ora-CAC protocols,^84^ which have been used to develop and support the approval of anti-inflammatories for the eye.^85,86^ The challenges described in Figure 4 and Figure S10 correspond to time points after these eight challenges on days 21–24. Compounds **35.HCl** and **72.HCl** were tested as a phosphate-buffered solution at pH 6.6 (**35.HCl**: 0.1% = 1 mg/mL = 1.8 mM; **72.HCl**: 0.1% = 1 mg/mL = 2.2 mM). Hyperemia and squinting scores (Figure 4 and Figure S10, respectively) were assessed 30 min after the post-topical dose and 18 min after the post-topical challenge, using a 4-point scale with 0.5-unit increments (0 = none; 4 = extremely severe). Data were analyzed using a two-way ANOVA with Bonferroni posttests comparing each group to vehicle-treated controls.

## Supplementary Material













## Notes

2D NMR analysis for the regiochemistry of **35** and **72**, ^1^H/^13^C/^19^F NMR spectra, LCMS purity data, HRMS identity data of **8-34**/**60-71**/**73-75**, *in vitro* and *in vivo* pharmacological data, and qualitative solubility data (PDF)
PDB of H_1_R of **35** (PDB)
PDB of H_1_R of **72** (PDB)
PDB of H_4_R of **35** (PDB)
PDB of H_4_R of **72** (PDB)
Biochemical and biological data (CSV)

## Abbreviations

AC: allergic conjunctivitis
AD: atopic dermatitis
ADMET: absorption, distribution, metabolism, excretion, and toxicity
CAC: conjunctival allergen challenge
CRE: cAMP response element
GPCR: G protein-coupled receptor
hERG: human ether-a-go-go-related gene
HxR: human histamine Hx receptor (x may be 1, 2, 3, or 4)
LLE: ligand lipophilicity efficiency
PK/PD: pharmacokinetics/pharmacodynamics
SAR: structure–activity relationship
TM: transmembrane
TPSA: total polar surface area.
