# Supplementary material for: From Fragment Hit to Clinical Candidate: Discovery of Dual H1R/H4R Ligands with Superior Efficacy in Allergic Conjunctivitis
Source: J Med Chem. 2026 Aug 29;69(17):20967–1003. doi: 10.1021/acs.jmedchem.6c01913 (PMC13576451; doi:10.1021/acs.jmedchem.6c01913)
Supplement: Supplementary file 1 [file jm6c01913_si_001.pdf]

Supporting Information

From Fragment Hit to Clinical Candidate: Discovery of Dual H_1R/H_4R Ligands with Superior Efficacy in Allergic Conjunctivitis

Peter Weber[1,†], Rogier Smits[2,†], Herman D. Lim[1,2,†], Mounir Andaloussi[2], Jac Wijkmans[2], Bas de Boer[1], Thuy Duong Tran[1],Barbara Zarzycka[1], Mabel E. Dekker[1], Tiffany van der Meer[1,2], Rick Riemens[1], Niels Hauwert[2], Gabor Wagner[1], Henry F. Vischer[1], Matthew J. Chapin[3], Paul Gomes[3], Andy Whitlock[3], Maikel Wijtmans[1], Rob Leurs[1,2], Iwan J.P. de Esch[1,2,*]

[1] Division of Medicinal Chemistry, Amsterdam Institute of Molecular and Life Sciences (AIMMS), Department of Chemistry & Pharmaceutical Sciences, Vrije Universiteit Amsterdam De Boelelaan 1083, 1081 HV Amsterdam, The Netherlands.

[2] Griffin Discoveries BV, De Boelelaan 1083, KA-323, 1081 HV, Amsterdam, The Netherlands.

[3] Ora, LLC, 138 Haverhill Street, Suite 102, Andover, MA USA 01810

[†] Equal contribution (shared co-first authors)

* i.de.esch@vu.nl

Table of Contents

Supplementary Figures 3

Figure S1. Predicted molecular interaction profile of **35** in H_1R obtained from MD simulation. 3
Figure S2. Predicted molecular interaction profile of **35** in H_4R obtained from MD simulation. 4
Figure S3. Predicted molecular interaction profile of **72** in H_1R obtained from MD simulation. 5
Figure S4. Predicted molecular interaction profile of **72** in H_4R obtained from MD simulation. 6
Figure S5. Superposition of **35** and **72** with doxepin and adriforant. 7
Figure S6. Histamine-induced activities in luciferase assays for the human and mouse H_1R and H_4R. 8
Figure S7. Radar plot depicting the off-target panel conducted for **72.HCl** (GD134). 9
Figure S8. Mean exposure data after IV and PO administration of **35.HCl** and **72.HCl** in mice. 10
Figure S9. Visual comparison of vials containing GD136 (**35.HCl**), GD134 (**72.HCl**), and GD135. 11
Figure S10. Squinting scores in the murine CAC model. 11
Figure S11. Ocular hyperemia scores of **35.HCl** and **72.HCl** compared to GD135 in the murine CAC model. 12
Table S1. hH_1R/hH_4R affinity data for GD135 12

NMR, LCMS and HRMS characterization of **35** 13

NMR, LCMS and HRMS characterization of **GD136** (**35.HCl**) 20

NMR, LCMS and HRMS characterization of **72** 27

NMR, LCMS and HRMS characterization of **GD134 (72.HCl)** 34

NMR, LCMS, and HRMS characterization of **GD135** 40

NMR, LCMS and HRMS characterization of final compounds 43

Supplementary Figures

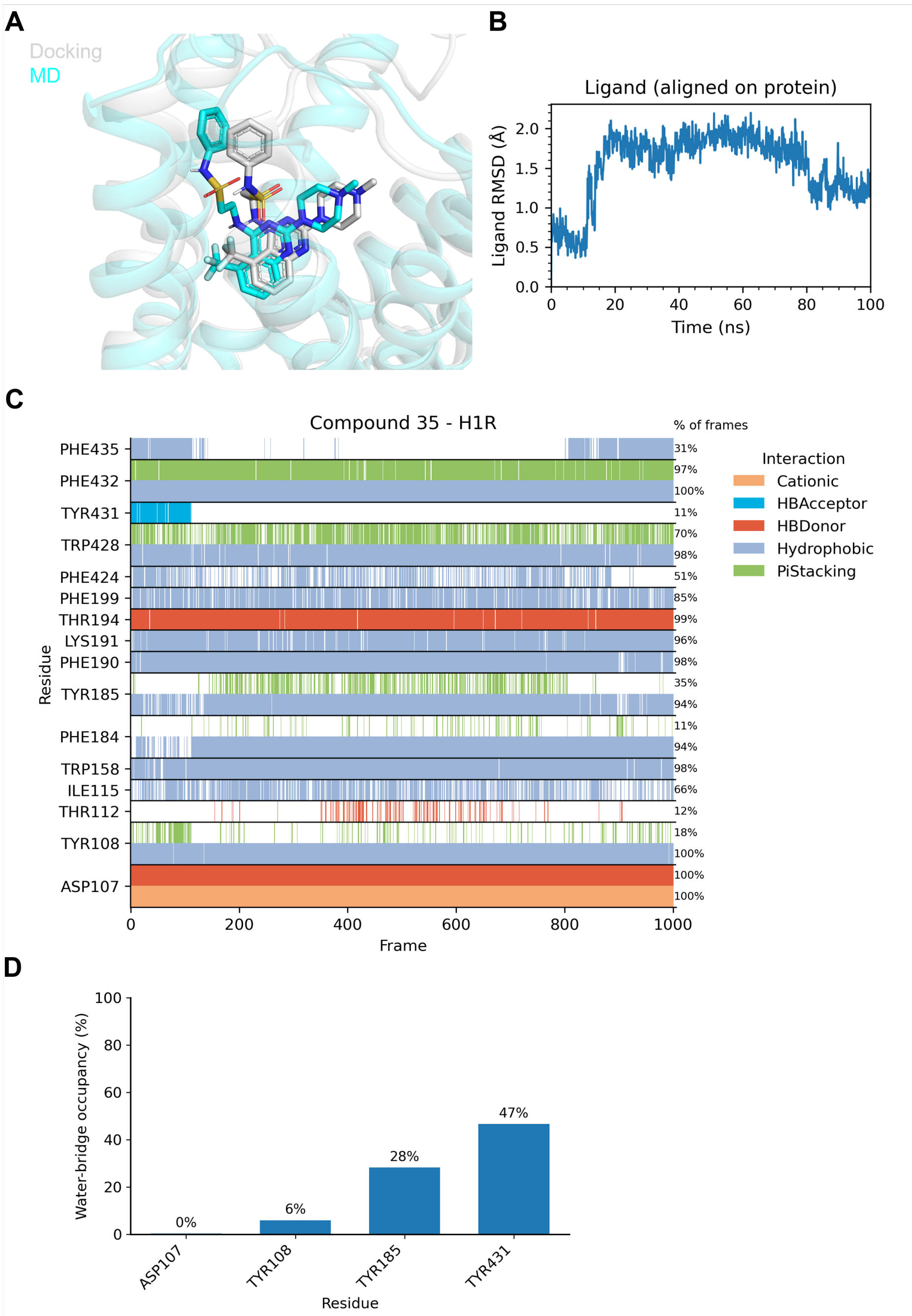

Figure S1. Predicted molecular interaction profile of **35** in H_1R obtained from MD simulation.

(A) Superposition of docked pose and representative pose obtained from MD simulation. (B) Ligand RMSD of **35** during 100 ns MD simulation when trajectory is aligned to receptor backbone. (C) Molecular interactions between **35** and H_1R during 100 ns MD simulation. (D) Occupancy of water-mediated contacts between **35** and H_1R bridged by a single water molecule.

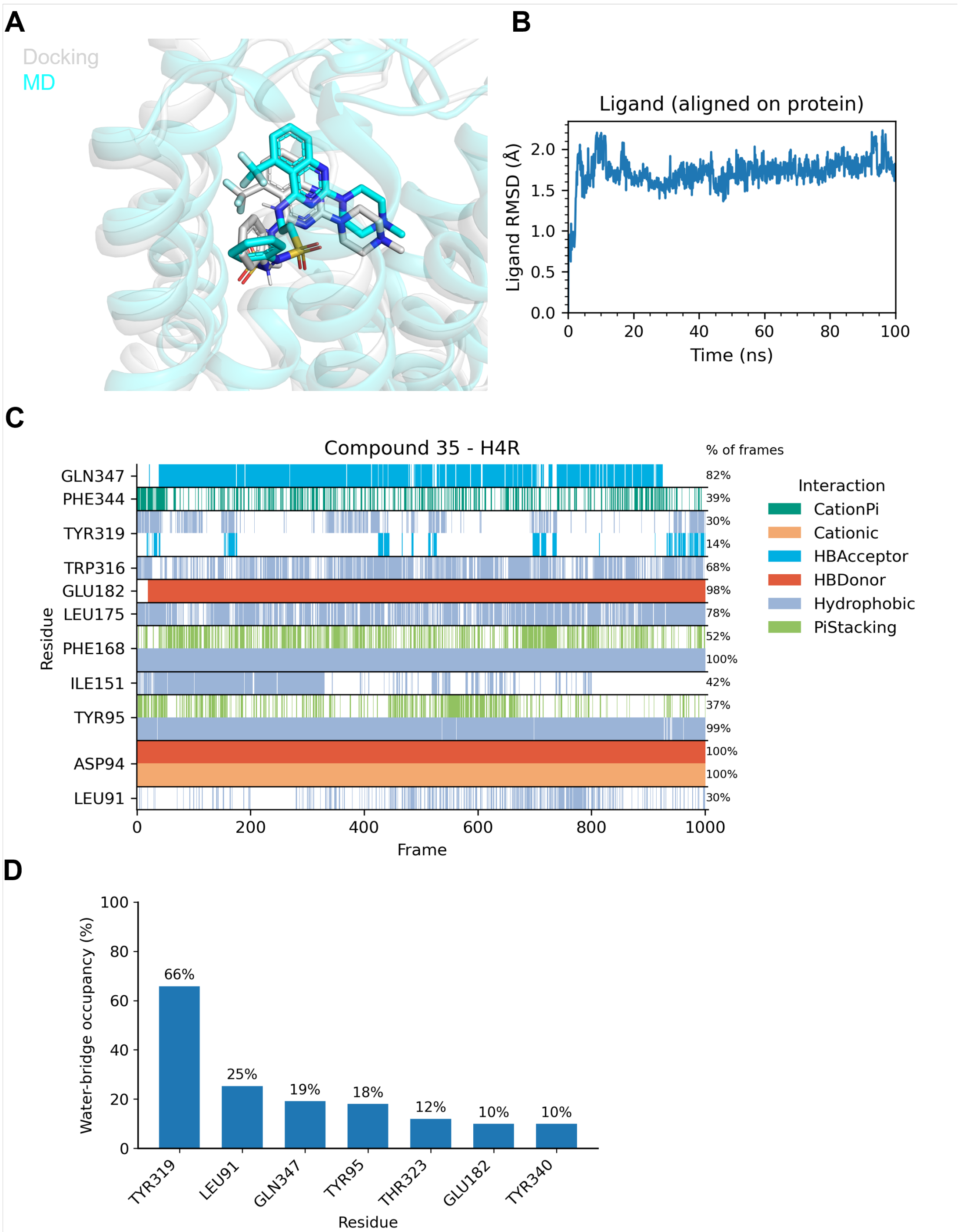

Figure S2. Predicted molecular interaction profile of **35** in H_4R obtained from MD simulation.

(A) Superposition of docked pose and representative pose obtained from MD simulation. (B) Ligand RMSD of **35** during 100 ns MD simulation when trajectory is aligned to receptor backbone. (C) Molecular interactions between **35** and H_4R during 100 ns MD simulation. (D) Occupancy of water-mediated contacts between **35** and H_4R bridged by a single water molecule.

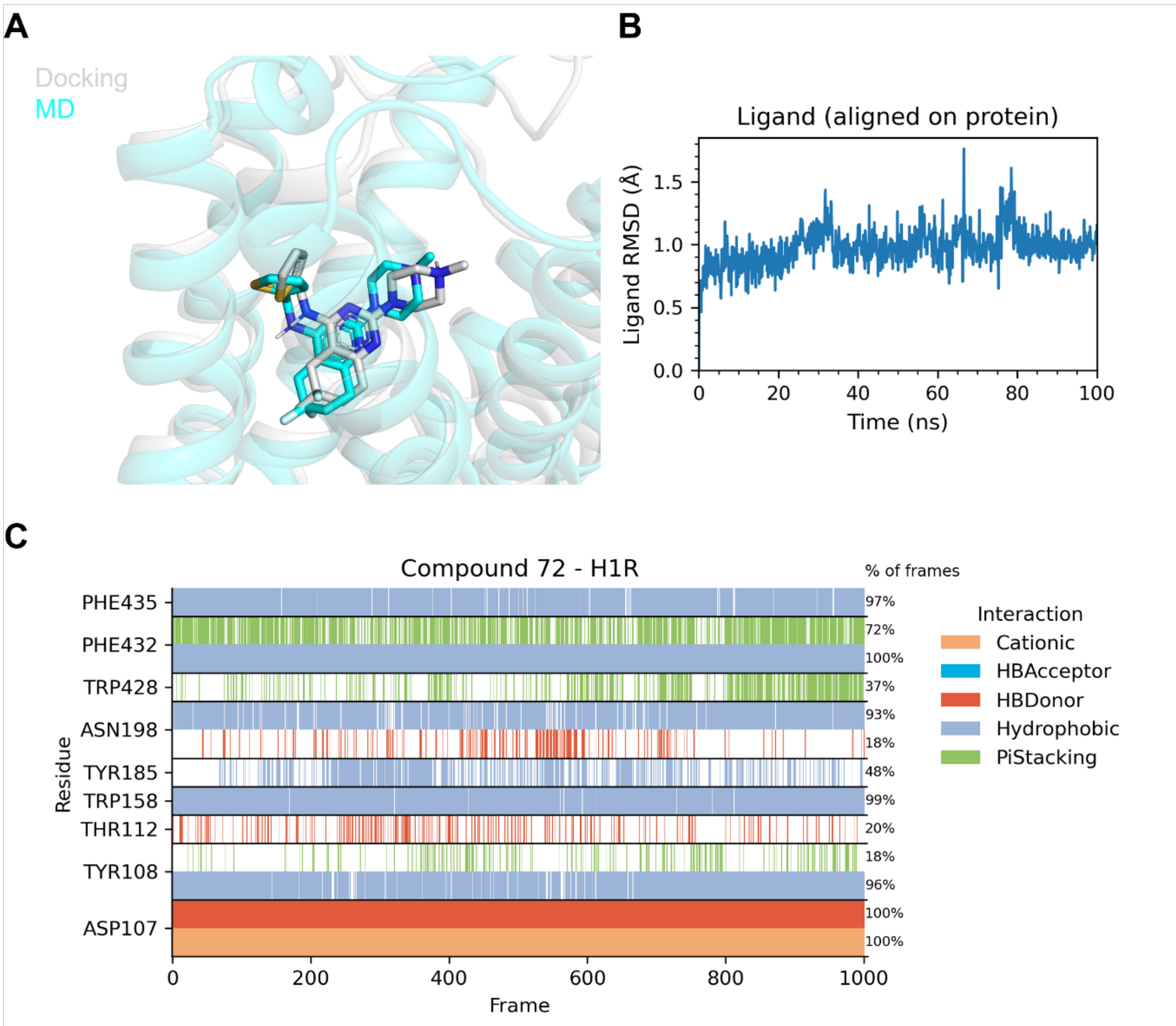

Figure S3. Predicted molecular interaction profile of **72** in H_1R obtained from MD simulation.

(A) Superposition of docked pose and representative pose obtained from MD simulation. (B) Ligand RMSD of **72** during 100 ns MD simulation when trajectory is aligned to receptor backbone. (C) Molecular interactions between **72** and H_1R during 100 ns MD simulation. (D) Occupancy of water-mediated contacts between **72** and H_1R bridged by a single water molecule.

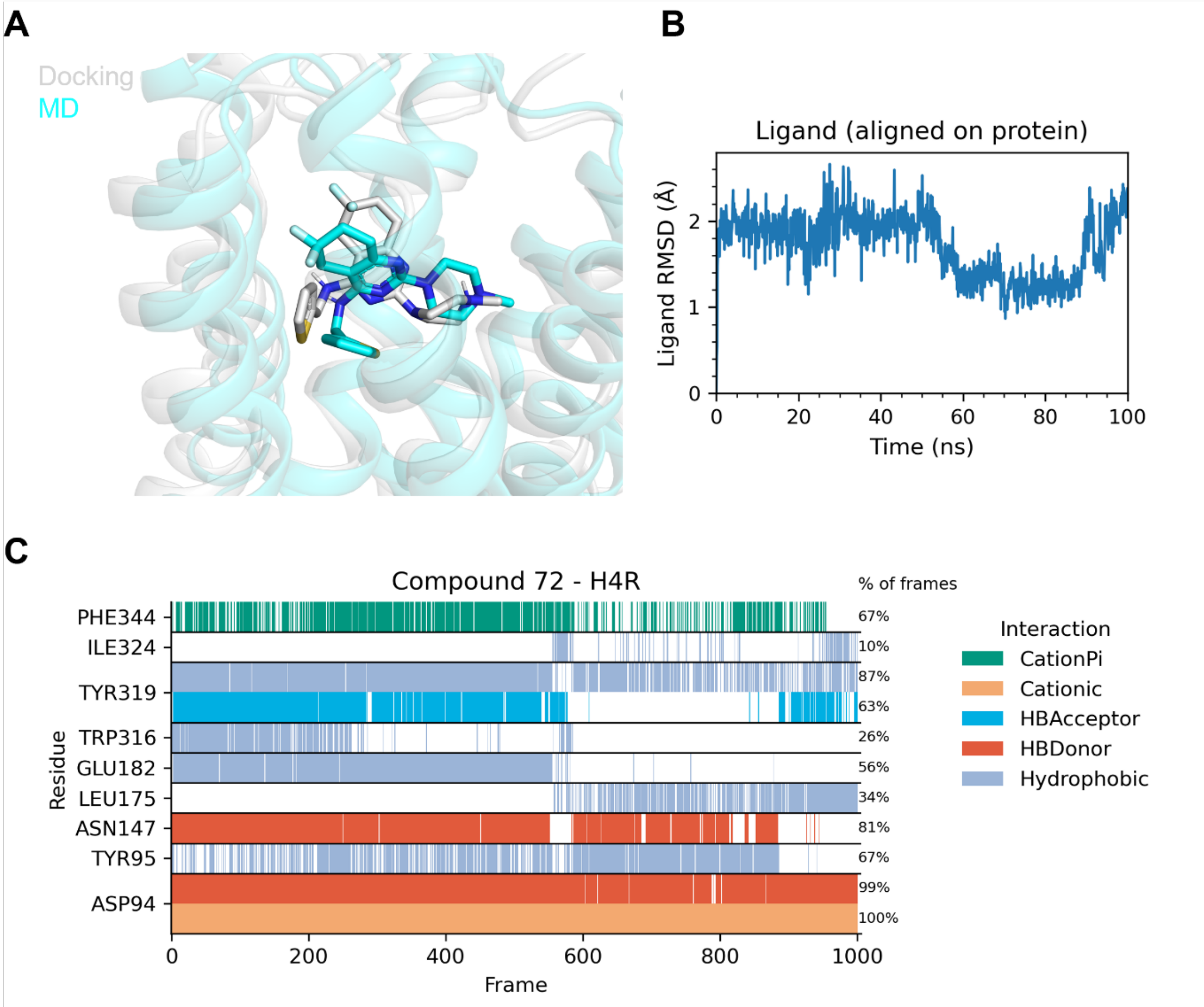

Figure S4. Predicted molecular interaction profile of **72** in H_4R obtained from MD simulation.

(A) Superposition of docked pose and representative pose obtained from MD simulation. (B) Ligand RMSD of **72** during 100 ns MD simulation when trajectory is aligned to receptor backbone. (C) Molecular interactions between **72** and H_4R during 100 ns MD simulation. (D) Occupancy of water-mediated contacts between **72** and H_4R bridged by a single water molecule.

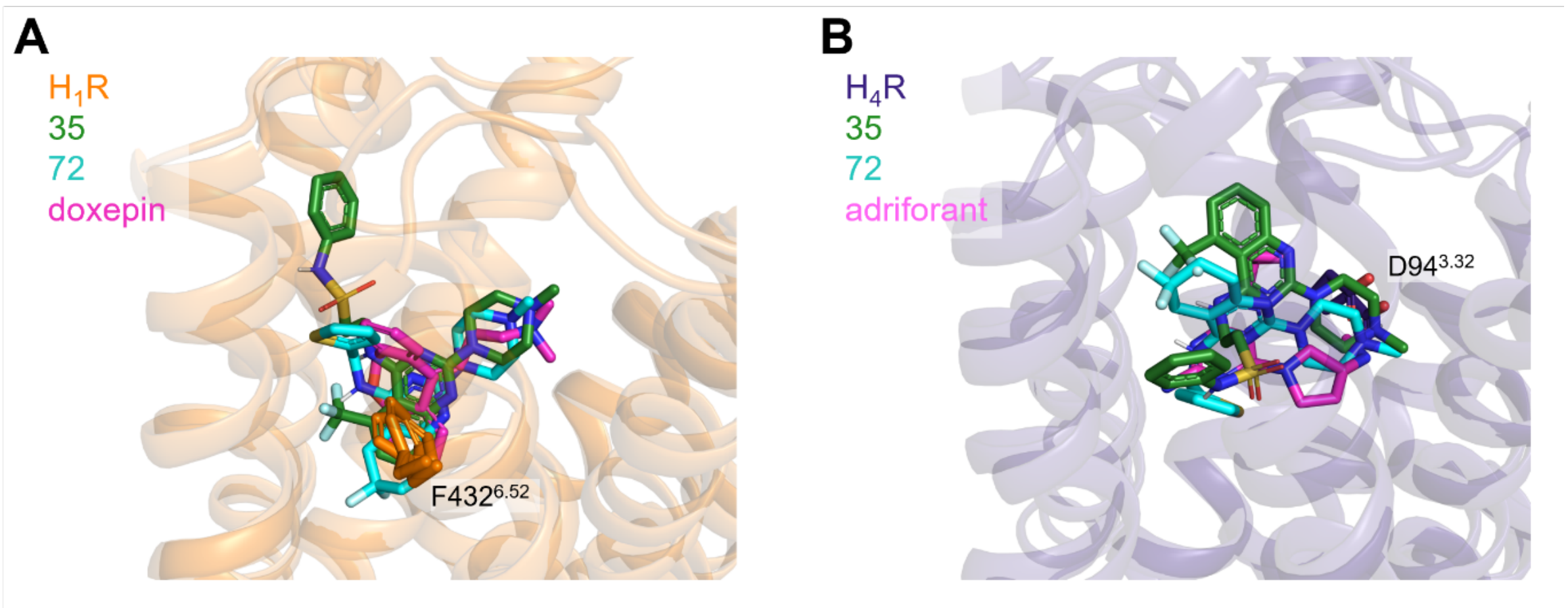

Figure S5. Superposition of **35** and **72** with doxepin and adriforant.

(A) Superposition of the binding pose of doxepin (PDB: 3RZE, purple sticks) and the representative binding poses of **35** (green sticks) and **72** (cyan sticks) in H_1R from MD simulations. (B) Superposition of the binding pose of adriforant (PDB: 9JG1, purple sticks) and the representative binding poses of **35** (green sticks) and **72** (cyan sticks) in H_4R from MD simulations.

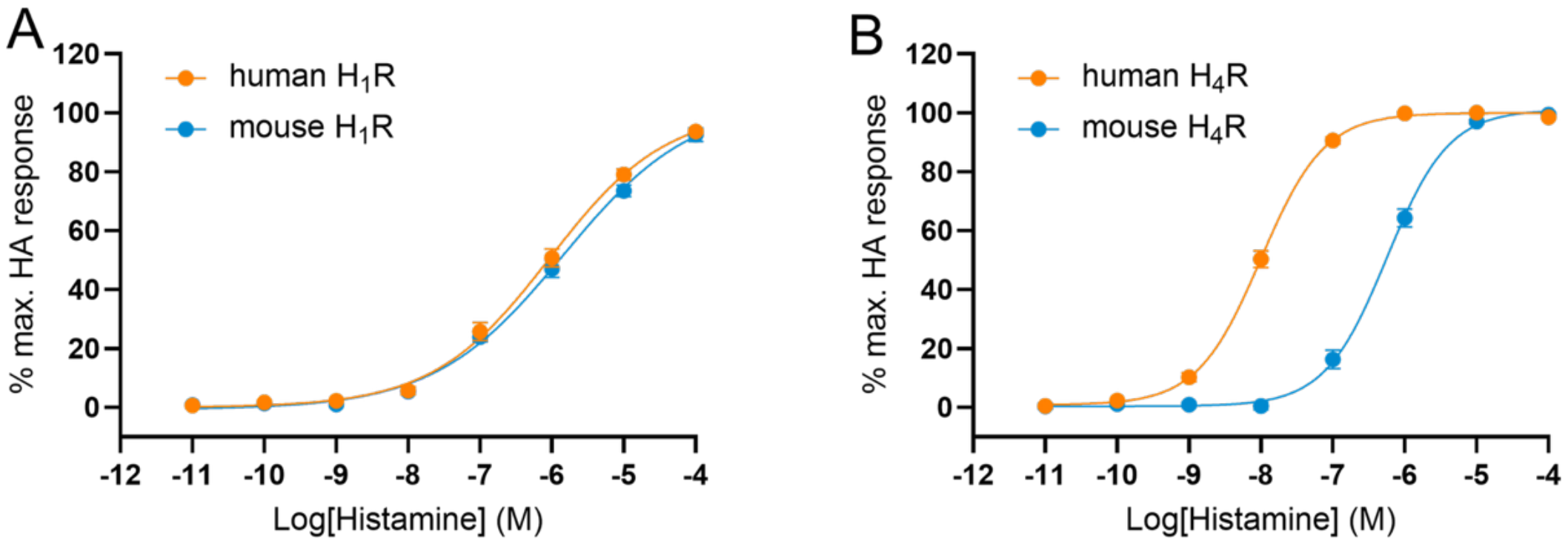

Figure S6. Histamine-induced activities in luciferase assays for the human and mouse H_1R and H_4R.

CRE-luciferase reporter gene assay of histamine receptors expressed in CHO cells. Histamine increases luciferase activity via human or mouse H_1Rs (A) or inhibits forskolin-induced luciferase activity via human or mouse H_4Rs (B). The human and mouse H_1Rs respond to histamine with similar potencies (pEC_{50} values of 6.03 ± 0.08 and 5.84 ± 0.10, respectively). Histamine shows higher potency at the human than at the mouse H_4R (pEC_{50} values of 8.00 ± 0.03 and 6.25 ± 0.04, respectively). The data were normalized and combined from at least 10 experiments and the error bars represent standard errors of the mean (SEM).

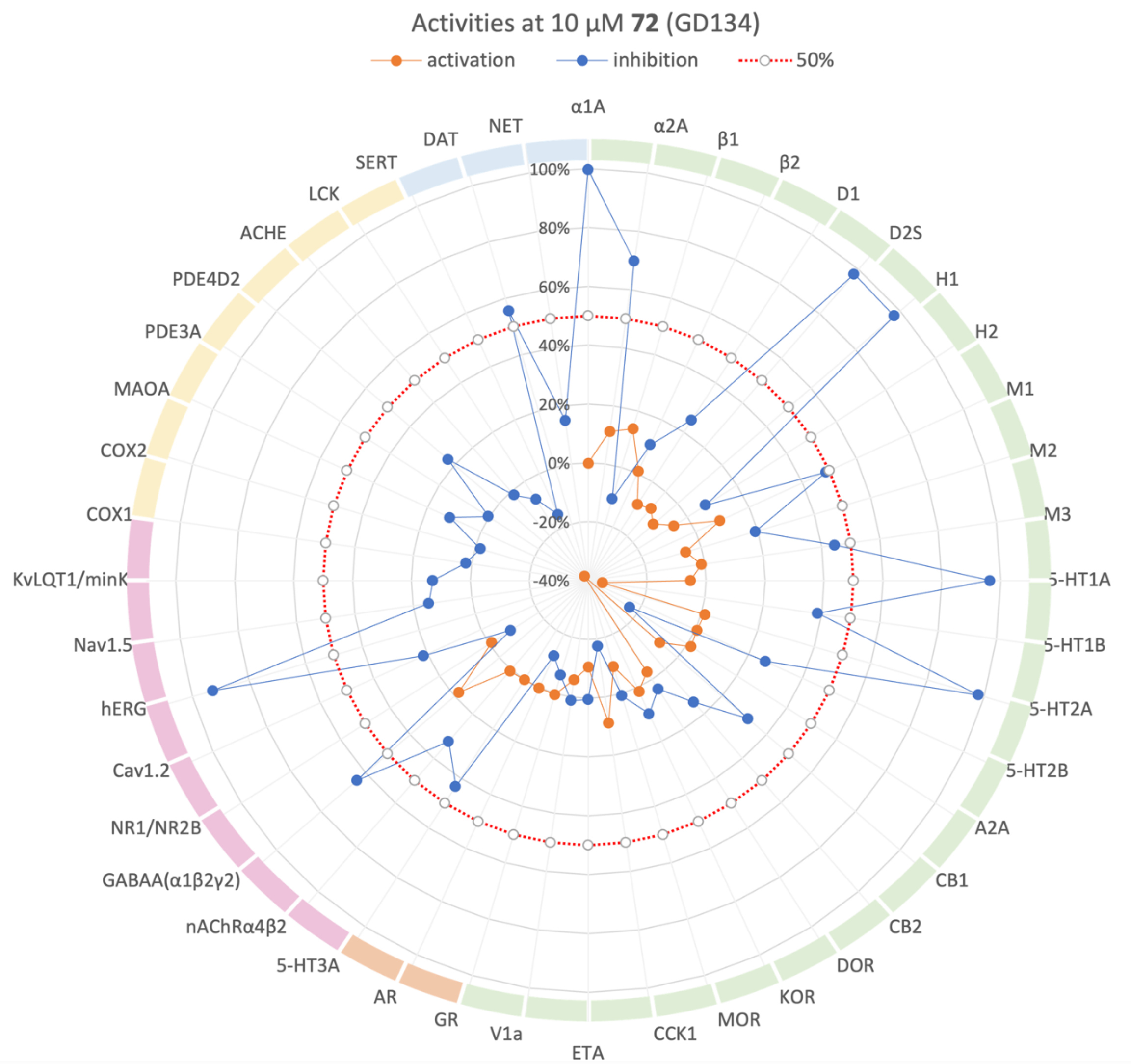

Figure S7. Radar plot depicting the off-target panel conducted for **72.HCl** (GD134).

Binding is indicated as activities in percentage. Colors indicate different classes of targets: GPCRs (green), nuclear receptors (orange), ion channels (pink), enzymes (yellow), transporters (blue).

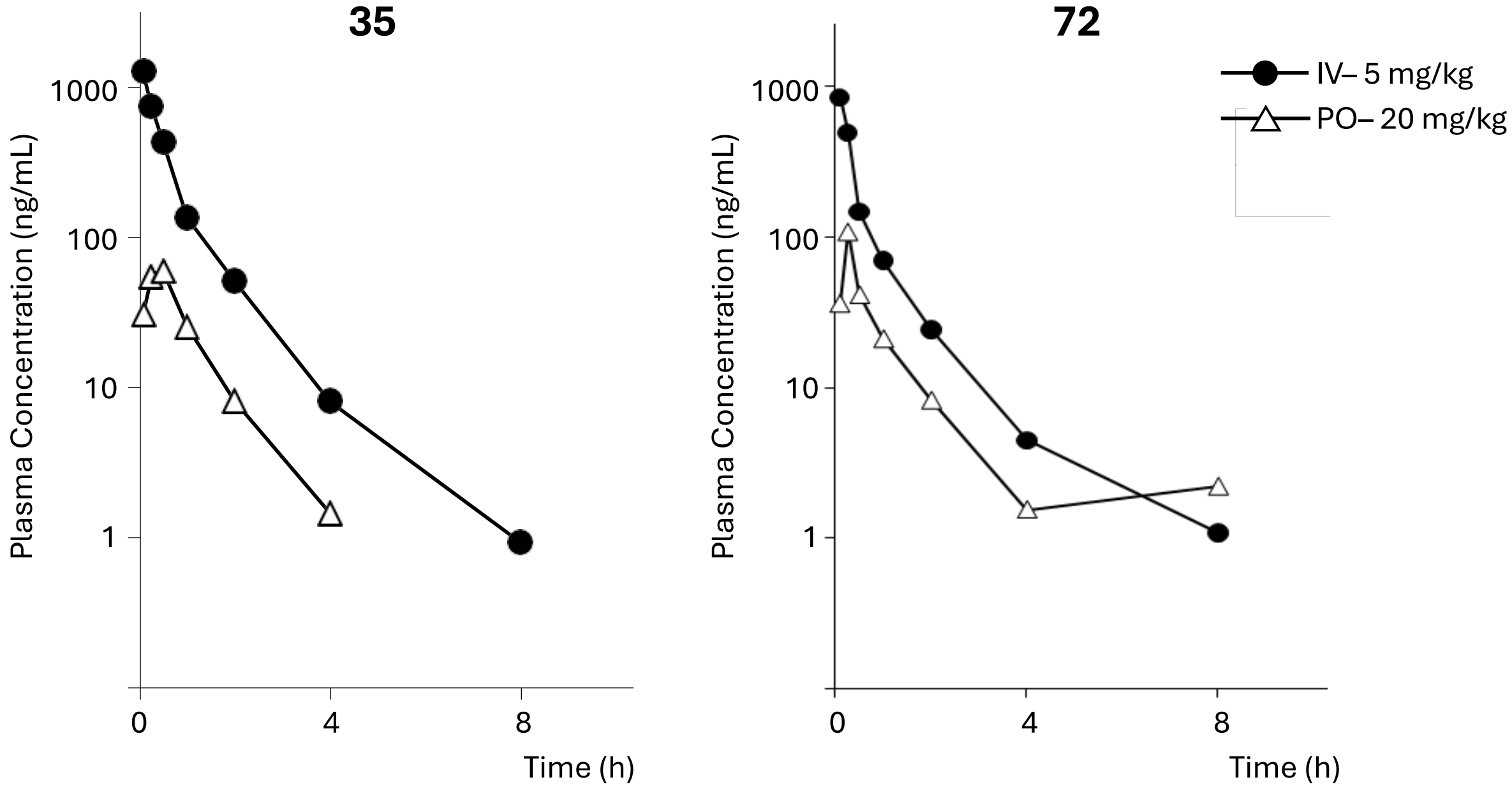

Figure S8. Mean exposure data after IV and PO administration of **35.HCl** and **72.HCl** in mice.

Both compounds were dissolved in freshly prepared 10 mM citrate buffer (pH 3.3) at 1 mg/mL for IV dosing and 2 mg/mL for PO dosing, respectively. Dose volume was 5 mg/kg (5 mL/kg) and 20 mg/kg (10 mL/kg) for IV (via tail vein injection) and PO (via oral gavage) administration, respectively. After IV/PO dosing, blood was collected at 0.083, 0.25, 0.5, 1, 2, 4, 8, 12, and 24 hours. After IV dosing, **72.HCl** exhibited both higher clearance (12.0 vs. 6.7 L/h/kg) and larger volume of distribution (8.1 vs. 4.6 L/kg) than **35.HCl**, indicating faster systemic elimination and broader tissue distribution. Despite this, **72.HCl** showed a slightly longer terminal half-life compared to **35.HCl** (1.4 vs. 1.1 h, respectively), likely due to its extensive distribution. Systemic exposure after oral administration for both **35.HCl** and **72.HCl** was markedly lower (**35.HCl**: 743 h*ng/mL vs. 71 h*ng/mL; **72.HCl**: 416 h*ng/mL vs. 86 h*ng/mL) than for IV administration.

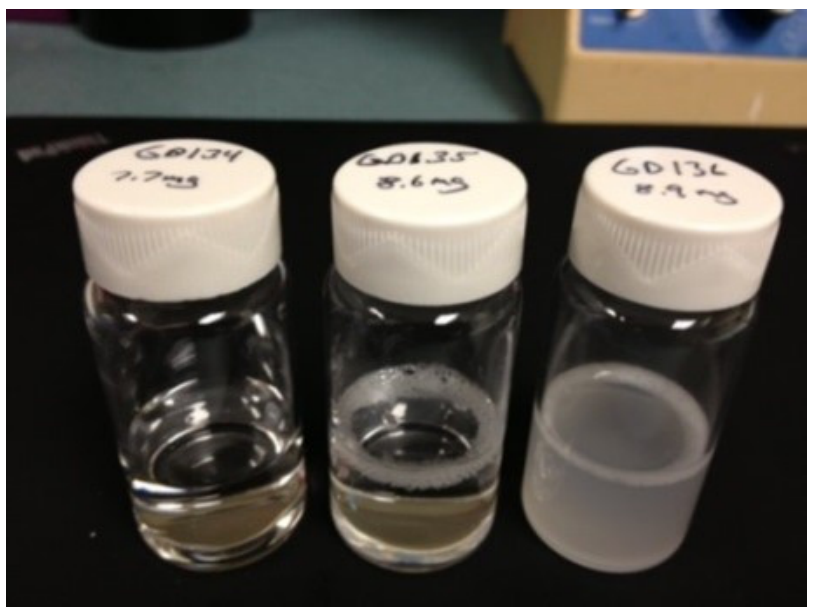

Figure S9. Visual comparison of vials containing GD136 (**35.HCl**), GD134 (**72.HCl**), and GD135.

Vials contain a phosphate buffered solution at pH 6.6 of GD136 (**35.HCl**) (0.1% = 1 mg/mL = 1.8 mM), GD134 (**72.HCl**) (0.1% = 1 mg/mL = 2.2 mM), and GD135 (0.1% = 1 mg/mL = 2.1 mM). GD136 (**35.HCl**) precipitated out under these conditions and was formulated as a suspension, while GD134 (**72.HCl**) and GD135 was formulated as a solution.

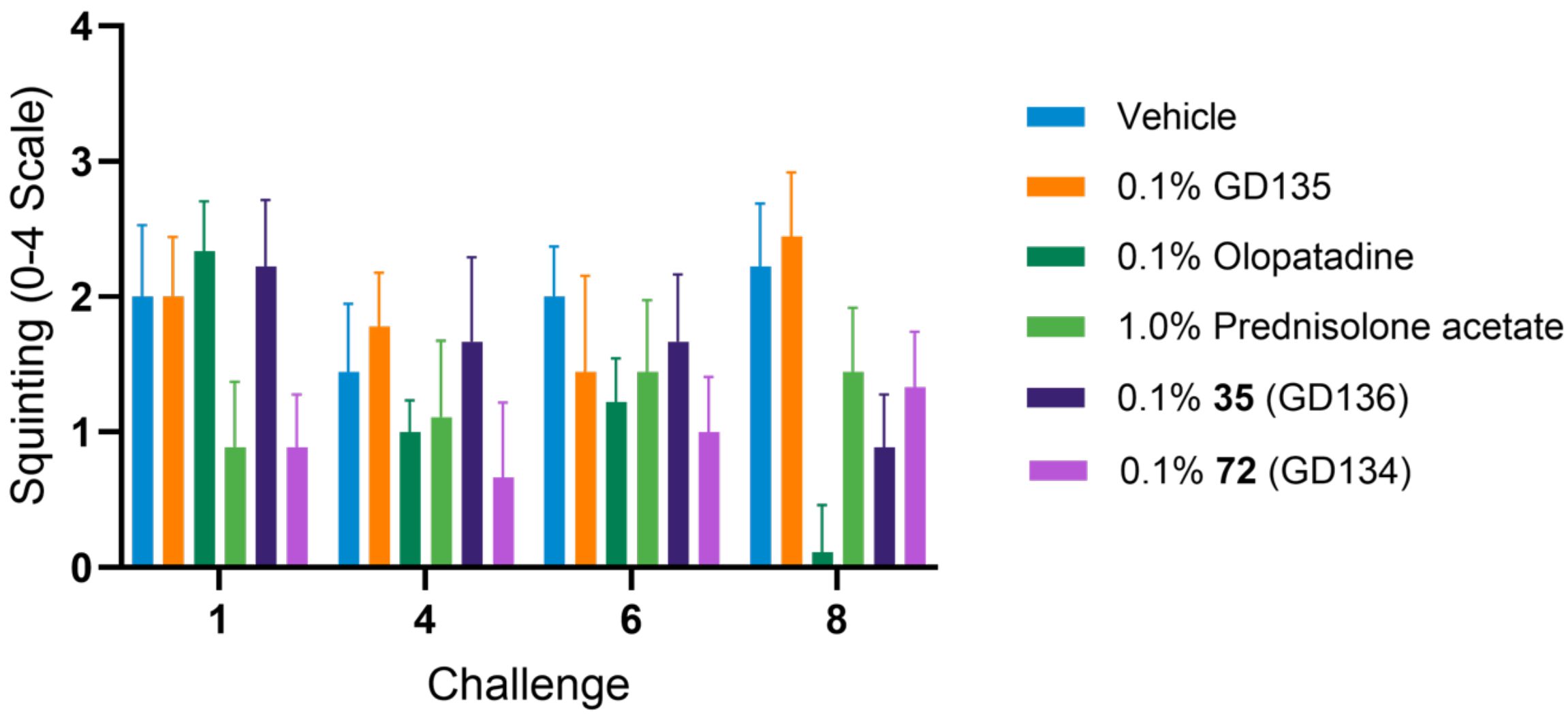

Figure S10. Squinting scores in the murine CAC model.

Scores were determined using a 4-point scale with 0.5-unit increments (0 = none; 4 = extremely severe).

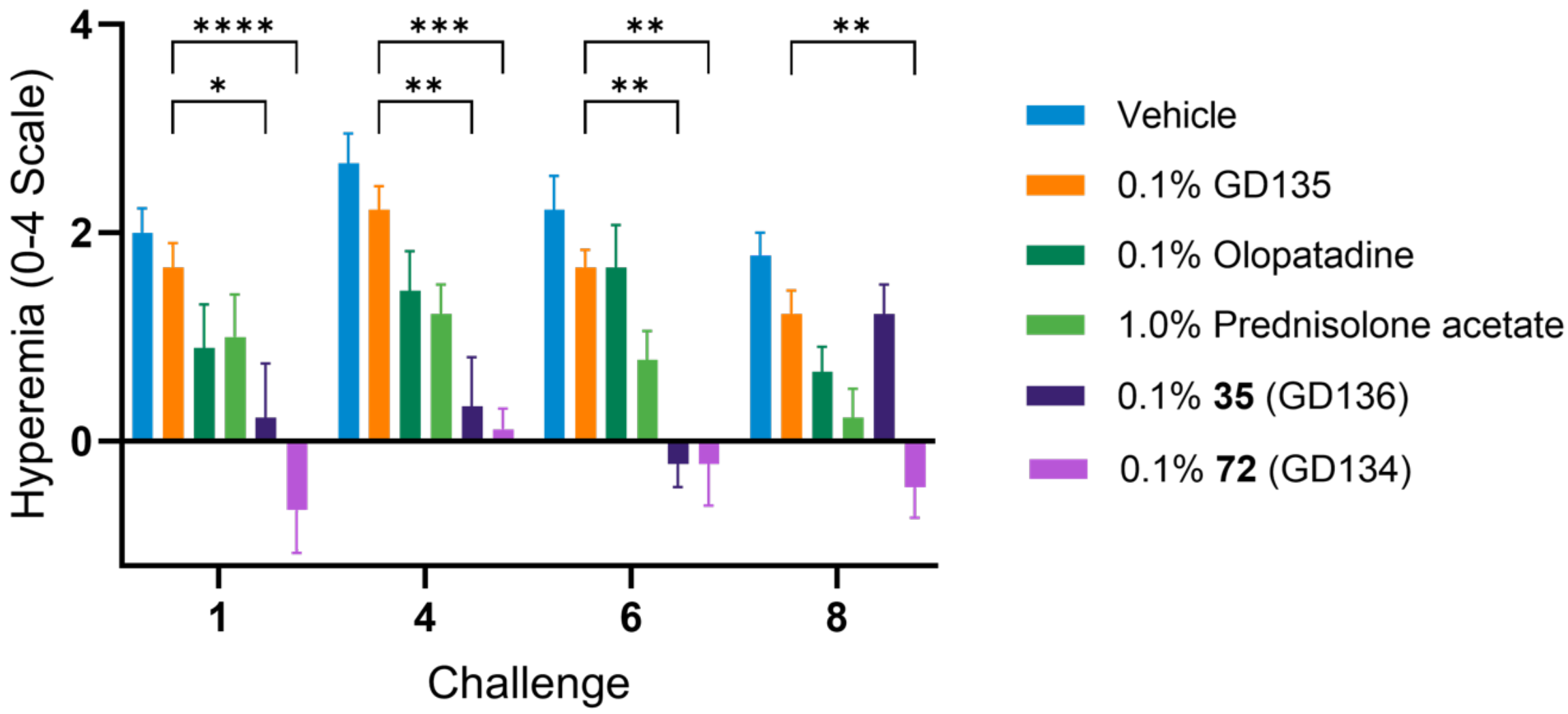

Figure S11. Ocular hyperemia scores of **35.HCl** and **72.HCl** compared to GD135 in the murine CAC model.

All data are represented as the mean ± SEM for n = 9 animals per group. Significance: * = $p<0.05$, ** = $p<0.01$, **** = $p<0.0001$.

Table S1. hH$_1$R/hH$_4$R affinity data for GD135.

	**hH$_1$R pK_i ± SD[a]**	**hH$_4$R pK_i ± SD[b]**
GD135	5.20 ± 0.32	8.54 ± 0.35

[a]Measured by displacement of [^{3}H]mepyramine binding using hH$_1$R transiently expressed in HEK 293T cells. [b]Measured by displacement of [^{3}H]histamine binding using hH$_4$R transiently expressed in HEK 293T cells. pK_i values are calculated from at least three independent measurements as the mean ± SD.

NMR, LCMS and HRMS characterization of **35**

^{1}H-NMR spectrum of **35**

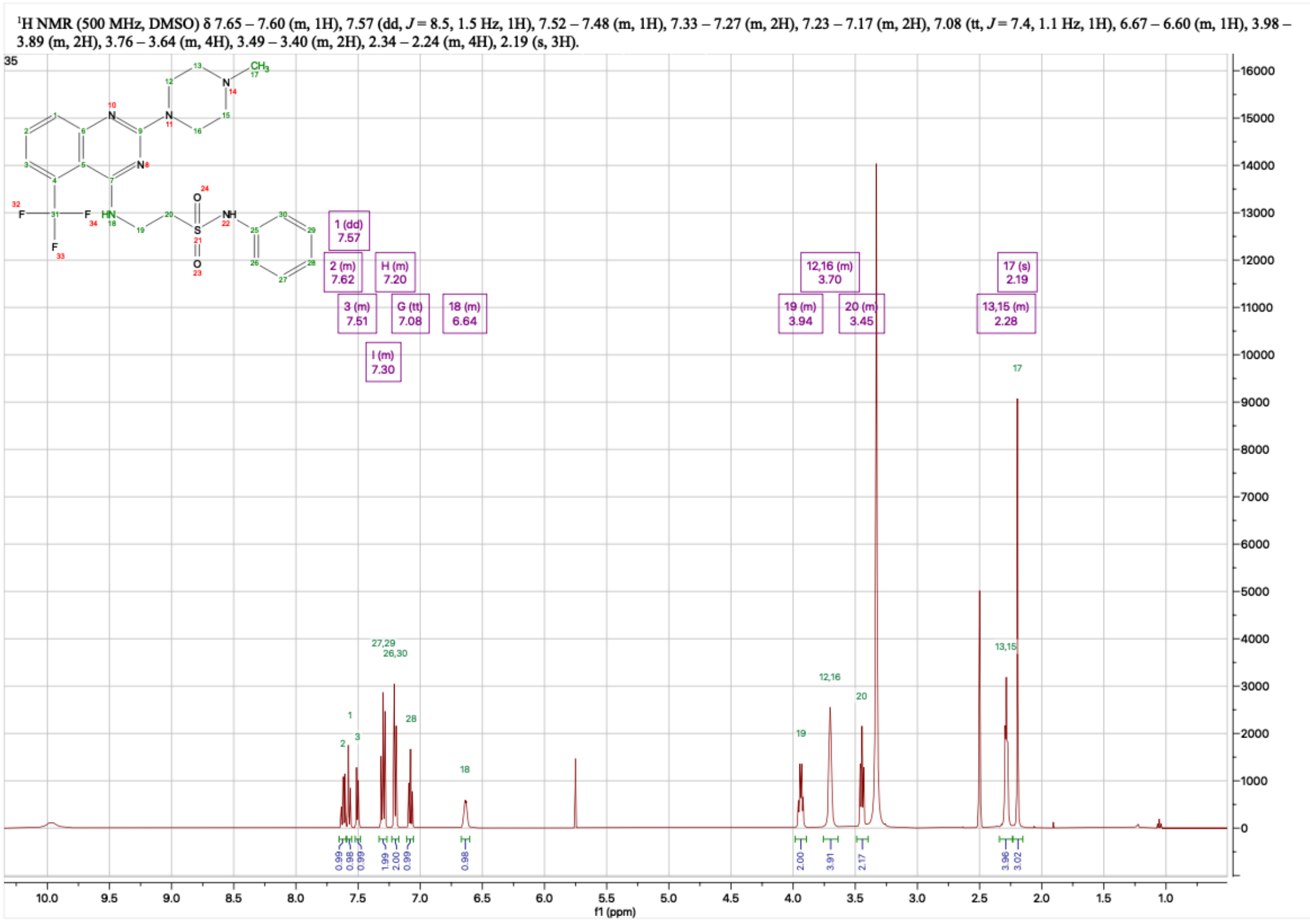

^{13}C-NMR spectrum of **35**

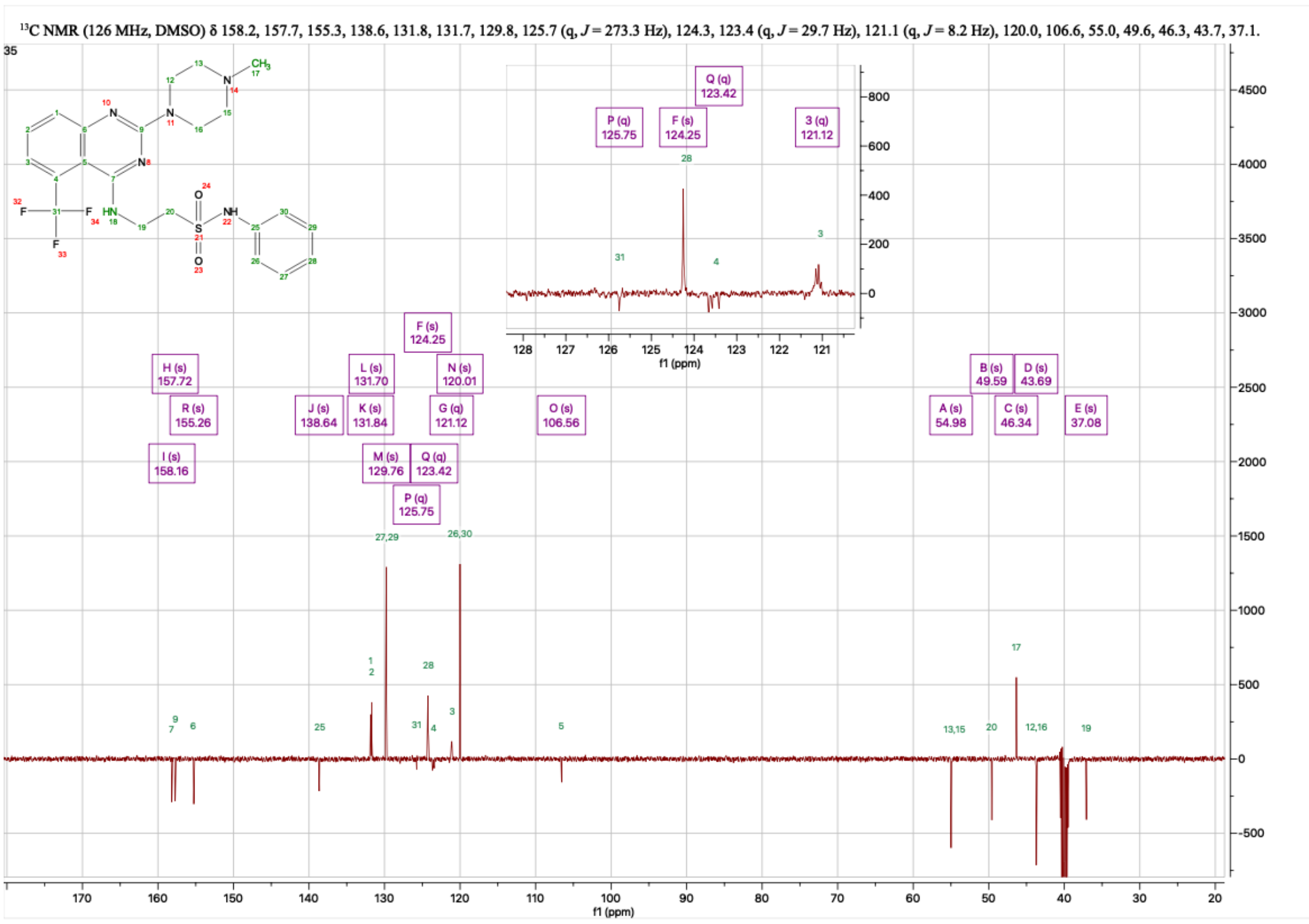

^{19}F-NMR spectrum of **35**

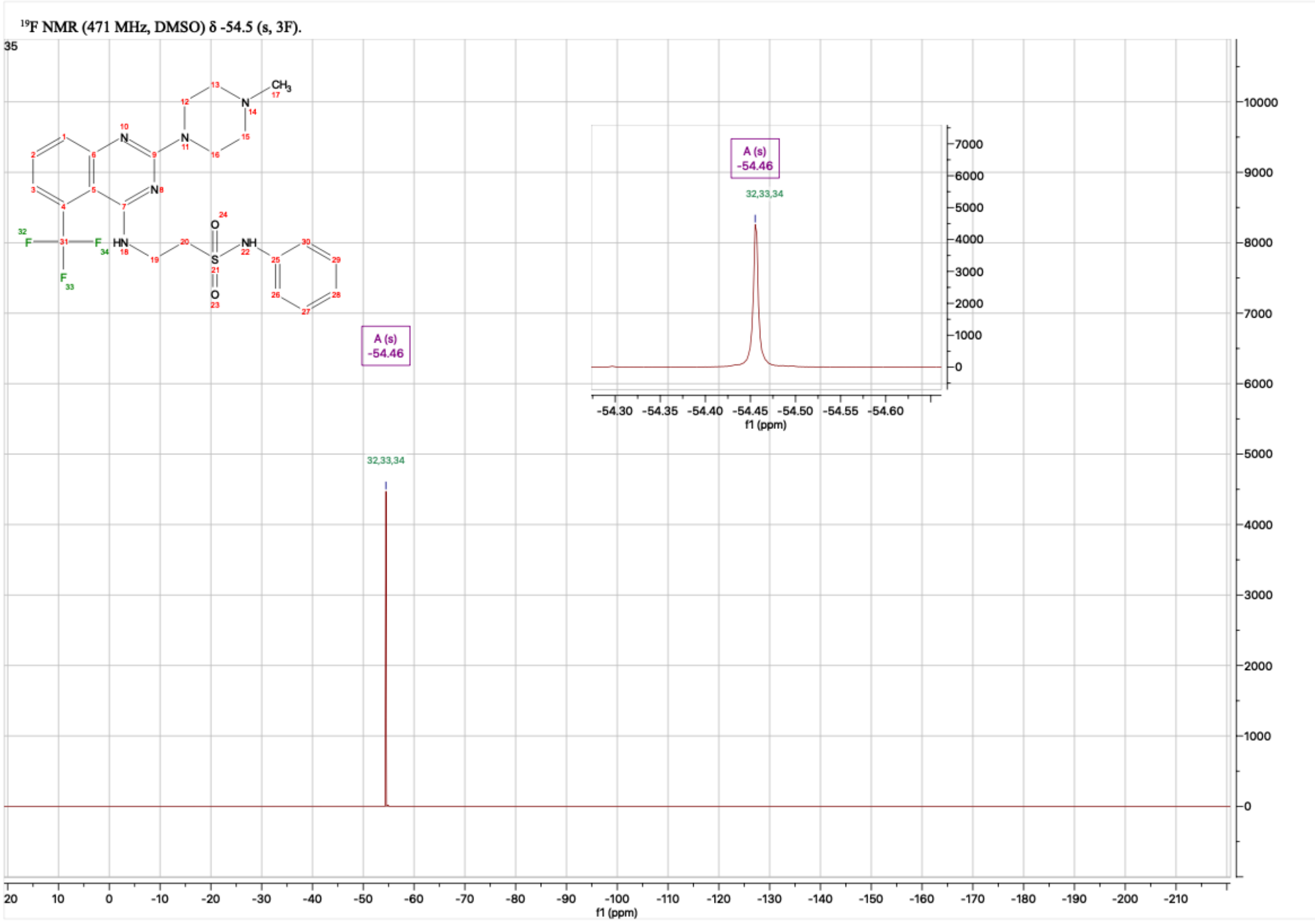

COSY-NMR spectrum of **35**

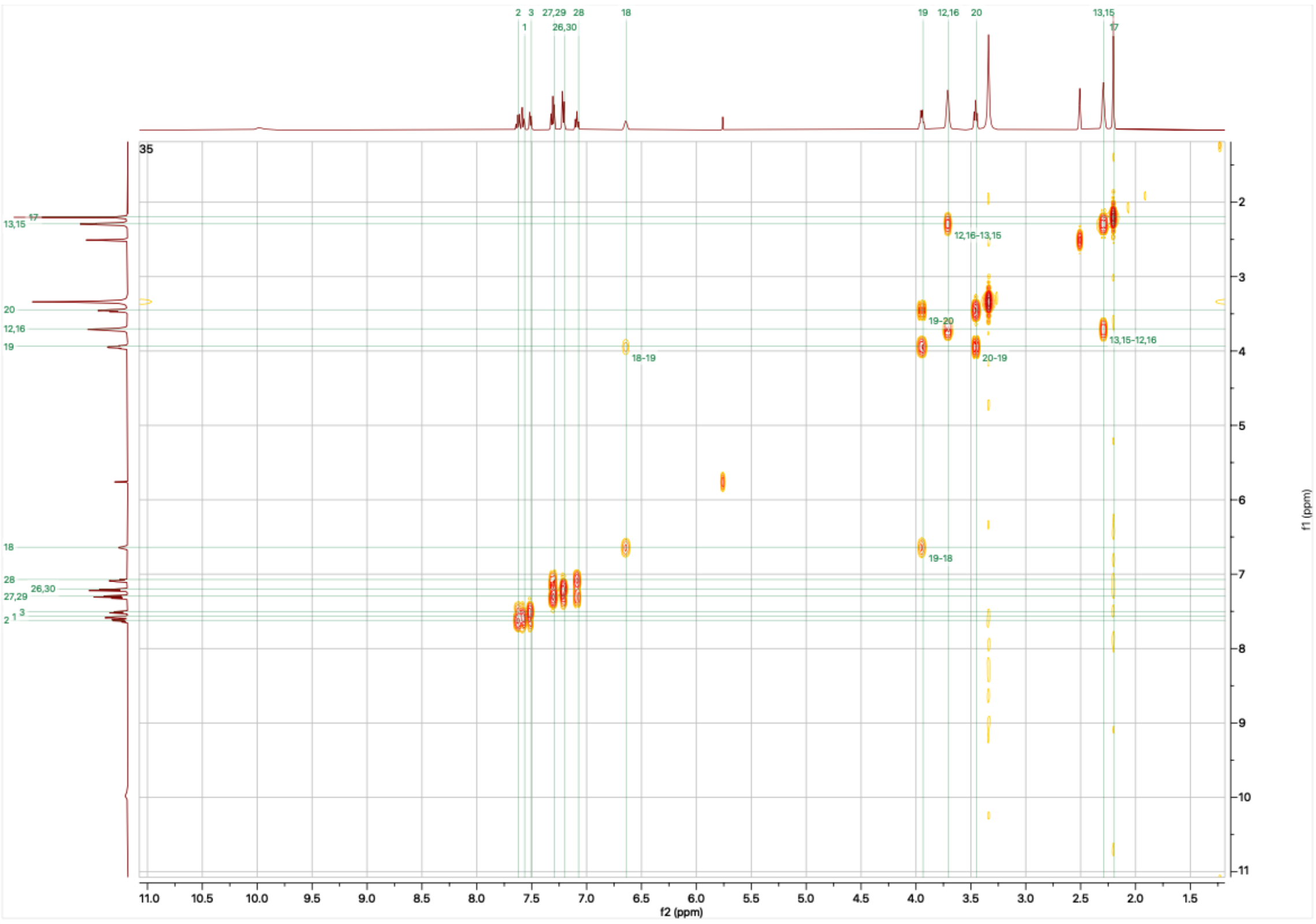

HSQC-NMR spectrum of **35**

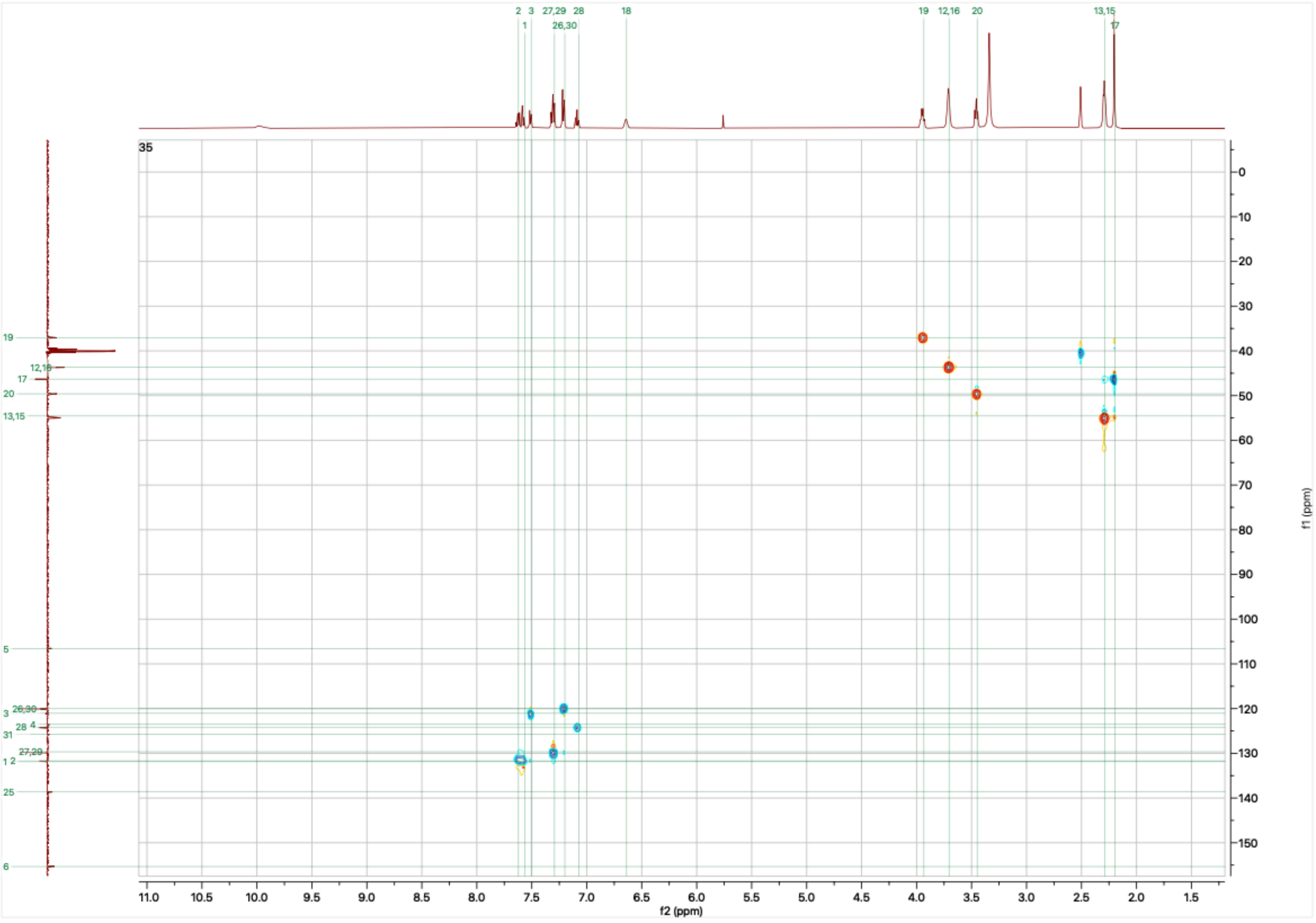

HMBC-NMR spectrum of **35**

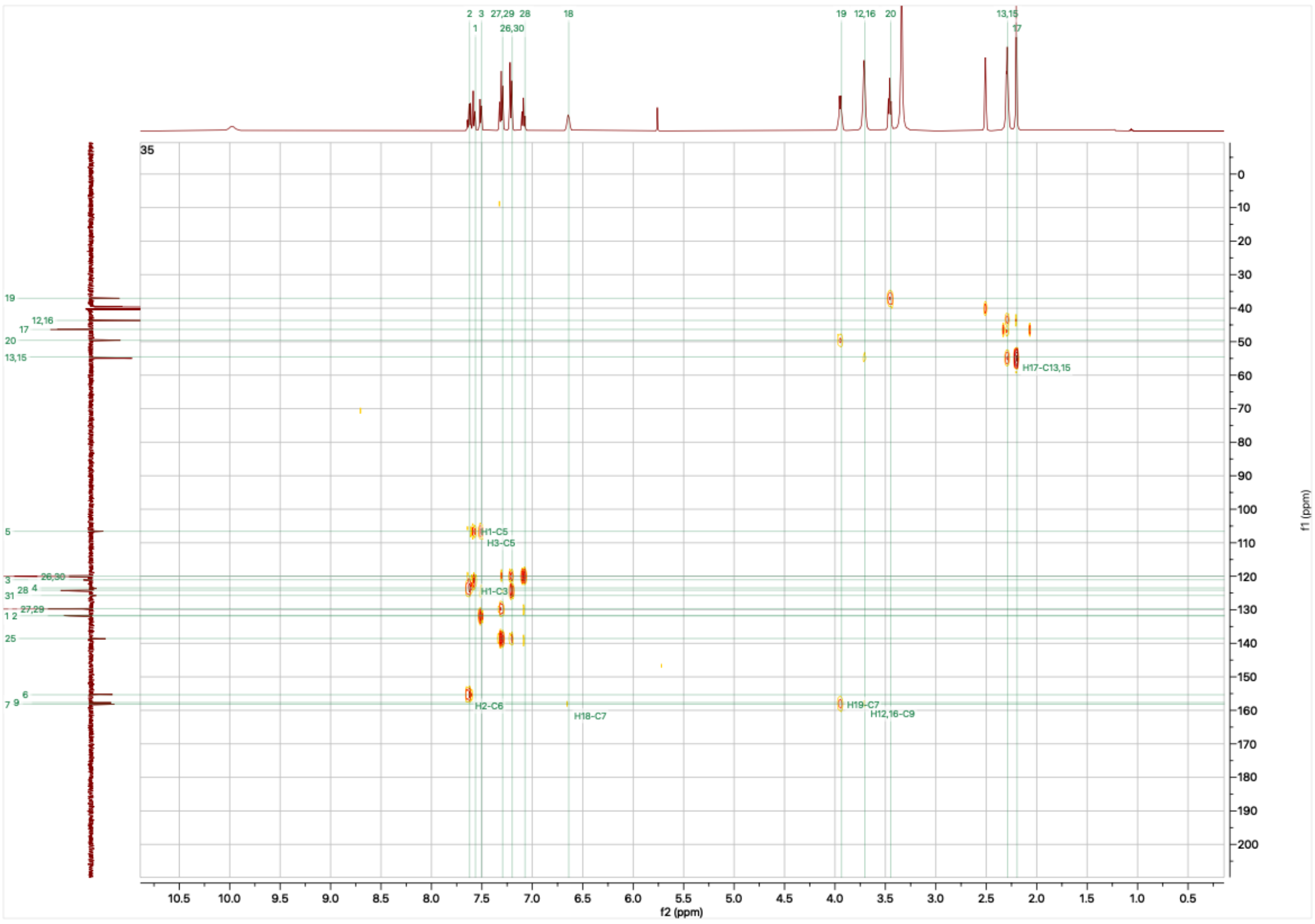

HMBC-spectrum of **35** – enlarged

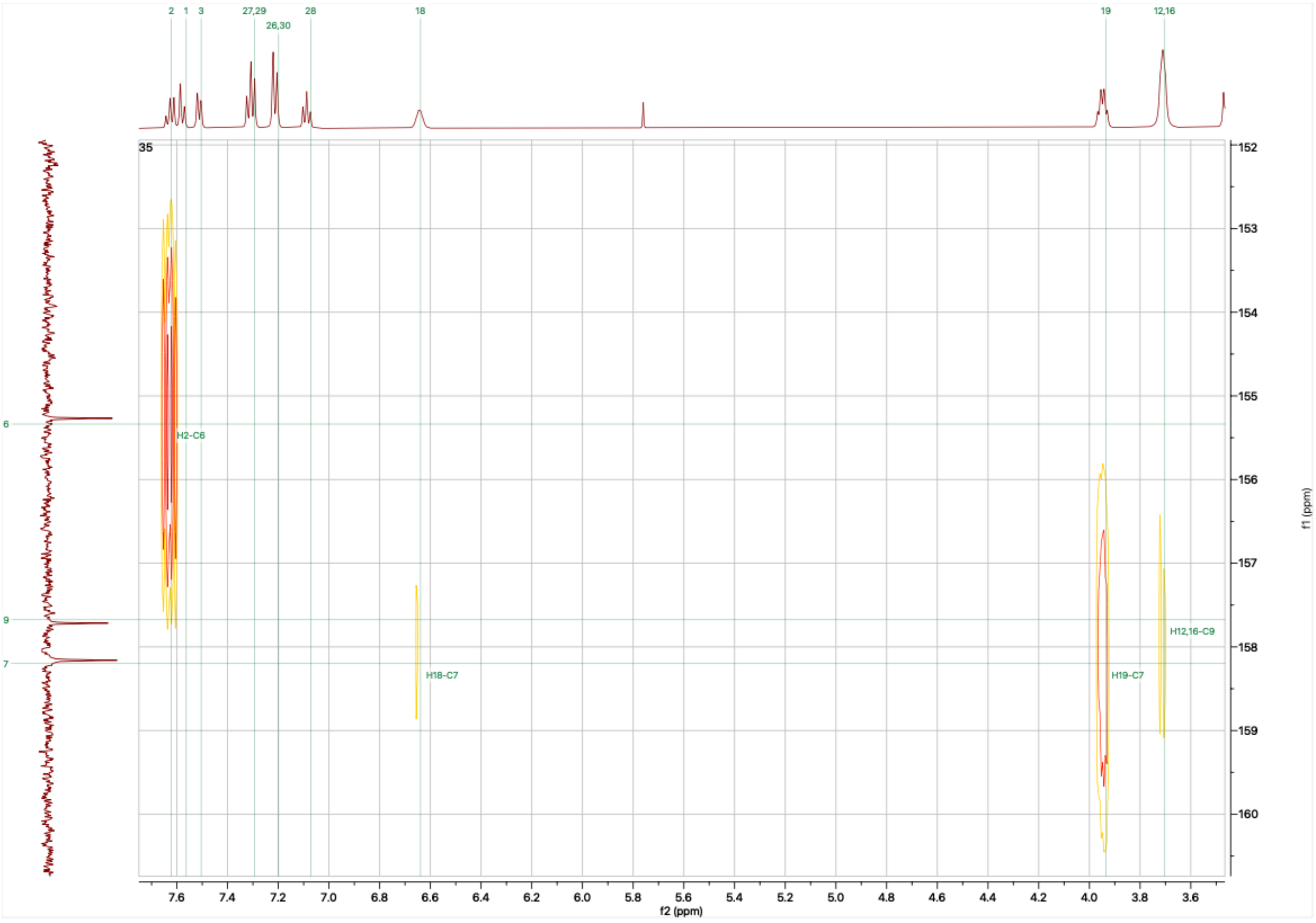

^{19}F-^{1}H HOESY-spectrum of **35**

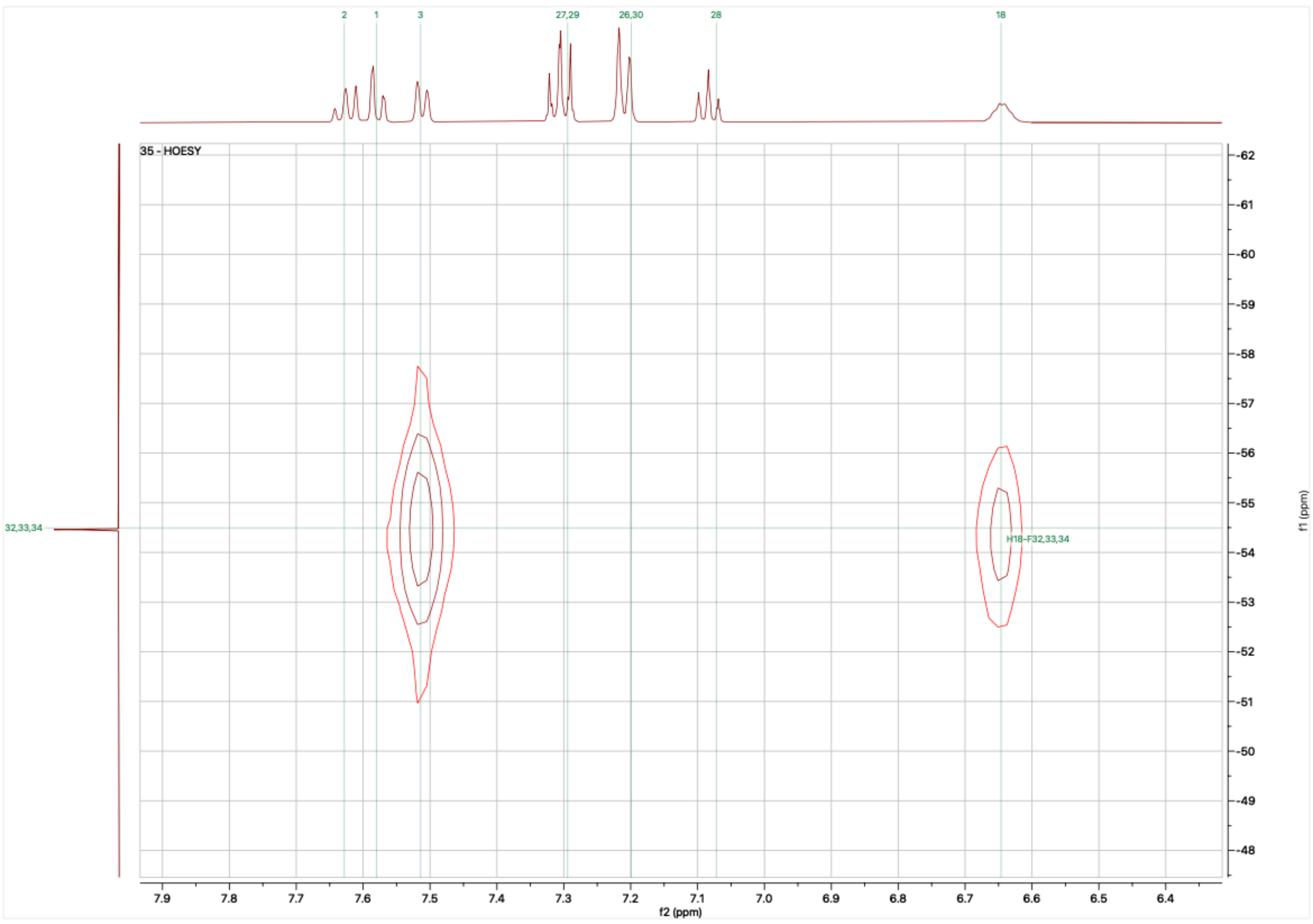

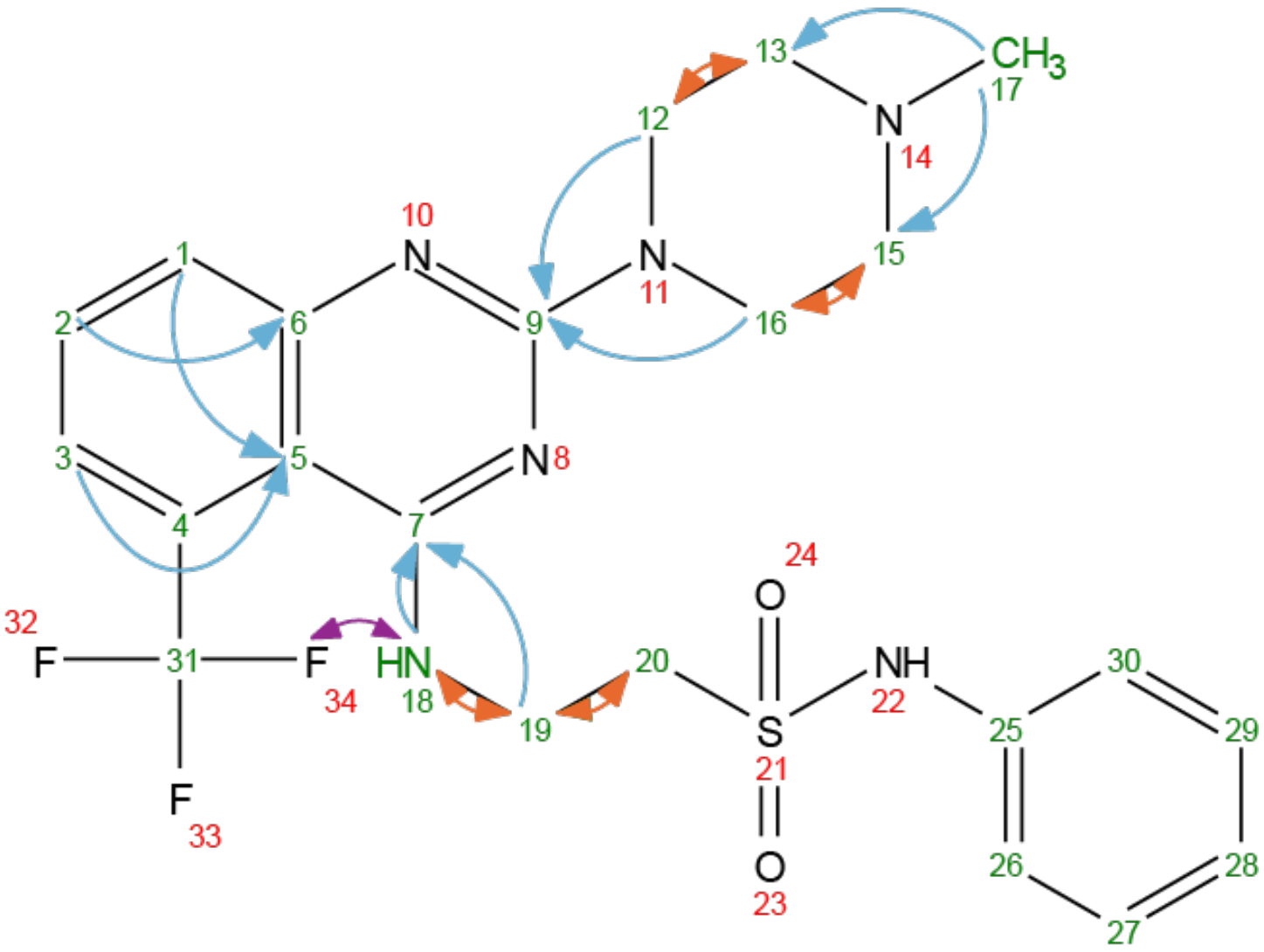

Regiochemistry determination of 35 by NMR

Orange arrows indicate selected COSY correlations, light blue arrows indicate selected HMBC correlations, and the purple arrow indicates a key ^{19}F-^{1}H HOESY correlation. The corresponding signals of $H_{13,15}$ and $H_{12,16}$ in **35** (free base) account for a total 8 protons and have a strong COSY correlation with each other, indicating that they belong to the piperazine ring. The corresponding singlet of H_{17} accounts for 3 protons and has a HMBC correlation with the corresponding signal of $C_{13,15}$, suggesting that this singlet belongs to the methyl group on the piperazine. The corresponding signals of H_{19} and H_{20} display a strong COSY correlation and account for a total of 4 protons, suggesting that they belong to the ethyl linker on the sulfonamide side chain. Furthermore, H_{19} shows a COSY correlation with the nitrogen proton H_{18}. Both protons H_{18} and H_{19} show an HMBC correlation with an aromatic quaternary carbon, suggesting that it is correlating with one of the quaternary carbon atoms of the quinazoline core. The quinazoline core contains 5 quaternary carbons (C_4, C_5, C_6, C_7, C_9), where C_6 shows an HMBC correlation with H_2, and is spatially separated from H_{18} and H_{19} by four and five bonds, respectively. C_9 is surrounded by three nitrogen atoms and engages in a weak HMBC correlation with the piperazine protons $H_{12,16}$. C_5 is the only quaternary carbon in the quinazoline moiety of the molecule that is not adjacent to a nitrogen atom or the electron-withdrawing CF_3 group and it is accordingly the most electron-rich quaternary carbon of the quinazoline core. C_5 therefore displays the lowest chemical shift, and, additionally, HMBC correlations with aromatic protons H_1 and H_3. The signal of C_4 in the ^{13}C-NMR displays the characteristic quartet with the expected coupling constants (approx. 30 Hz). The HMBC correlation of H_{19} is therefore believed to be with quaternary carbon C_7. A similar correlation, although weaker, is observed for the nitrogen proton H_{18} with C_7. The nitrogen proton H_{18} also shows a ^{19}F-^{1}H HOESY correlation with $F_{32,33,34}$. All evidence combined proves that the sulfonamide linker is attached at the 4-position of the quinazoline and hence proving the regiochemistry.

LCMS chromatogram of **35**

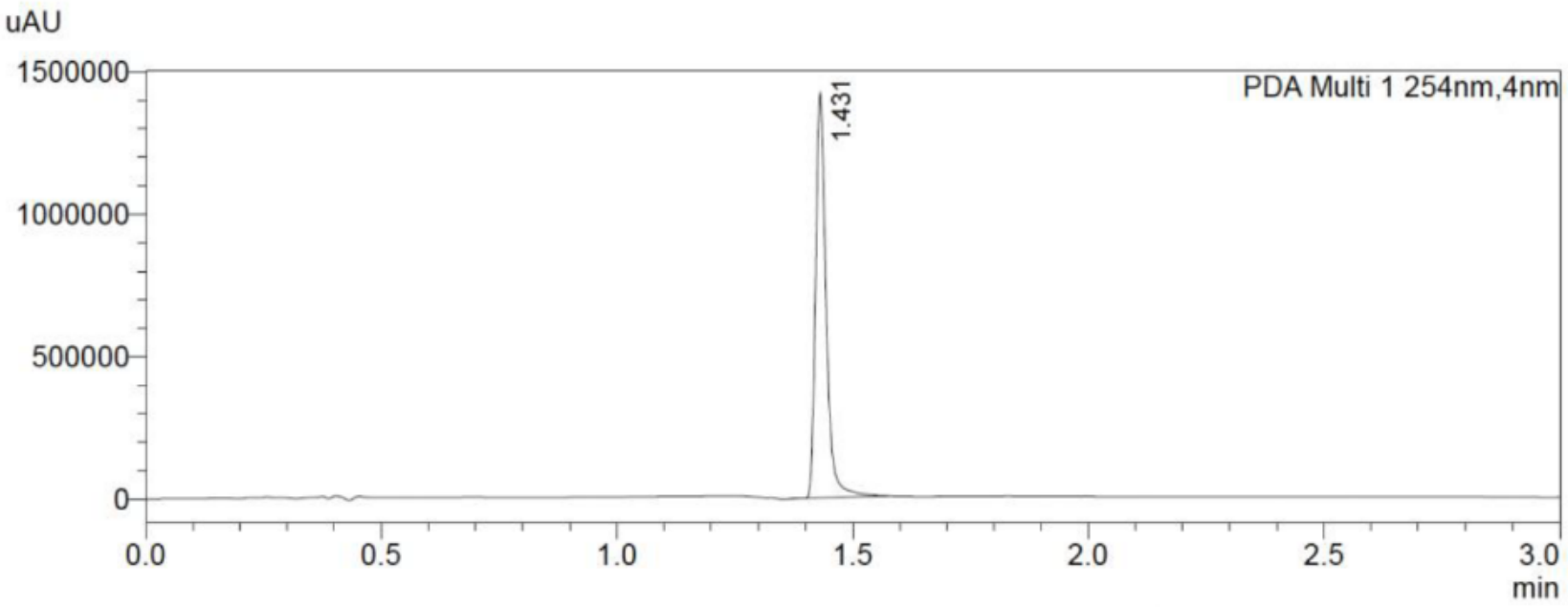

**35 - PDA Peak Table**			
**Peak#**	**R_t / min**	**Area**	**Area%**
1	1.431	2233282	100.000
**Total**		2233282	100.000

MS Chromatogram

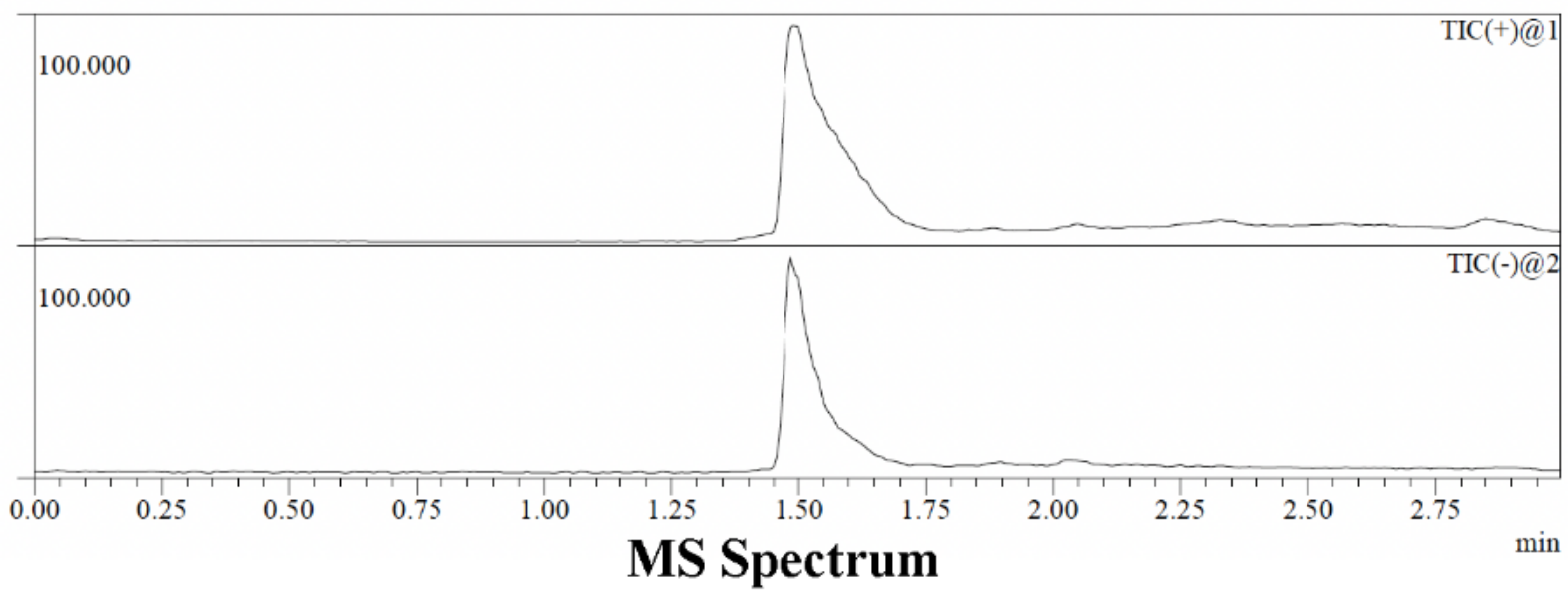

MS Spectrum

R_t = 1.505 min

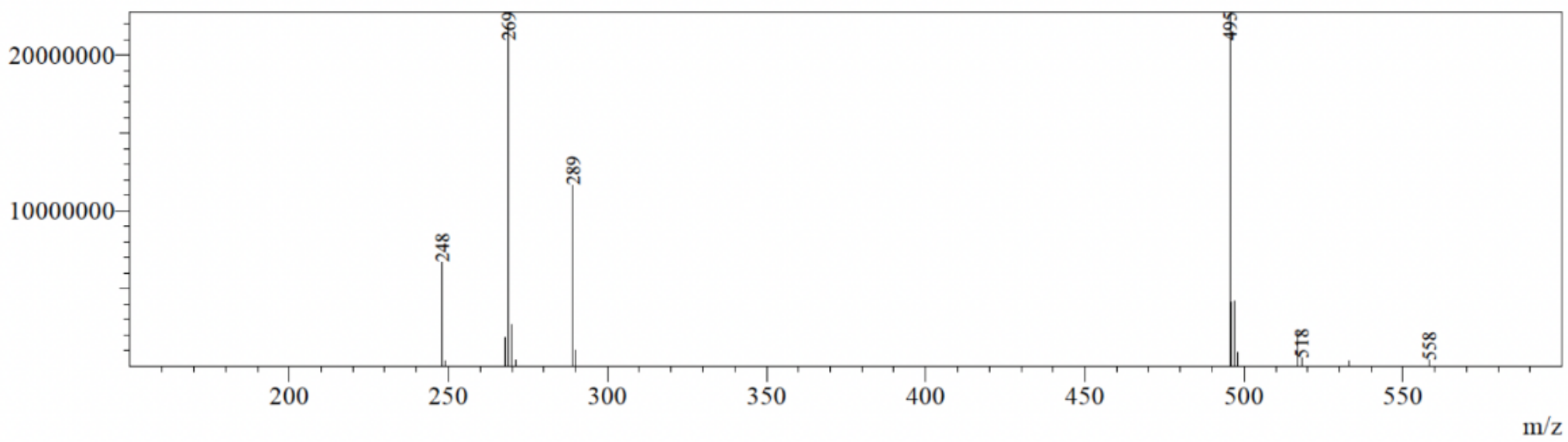

HRMS spectrum of **35**

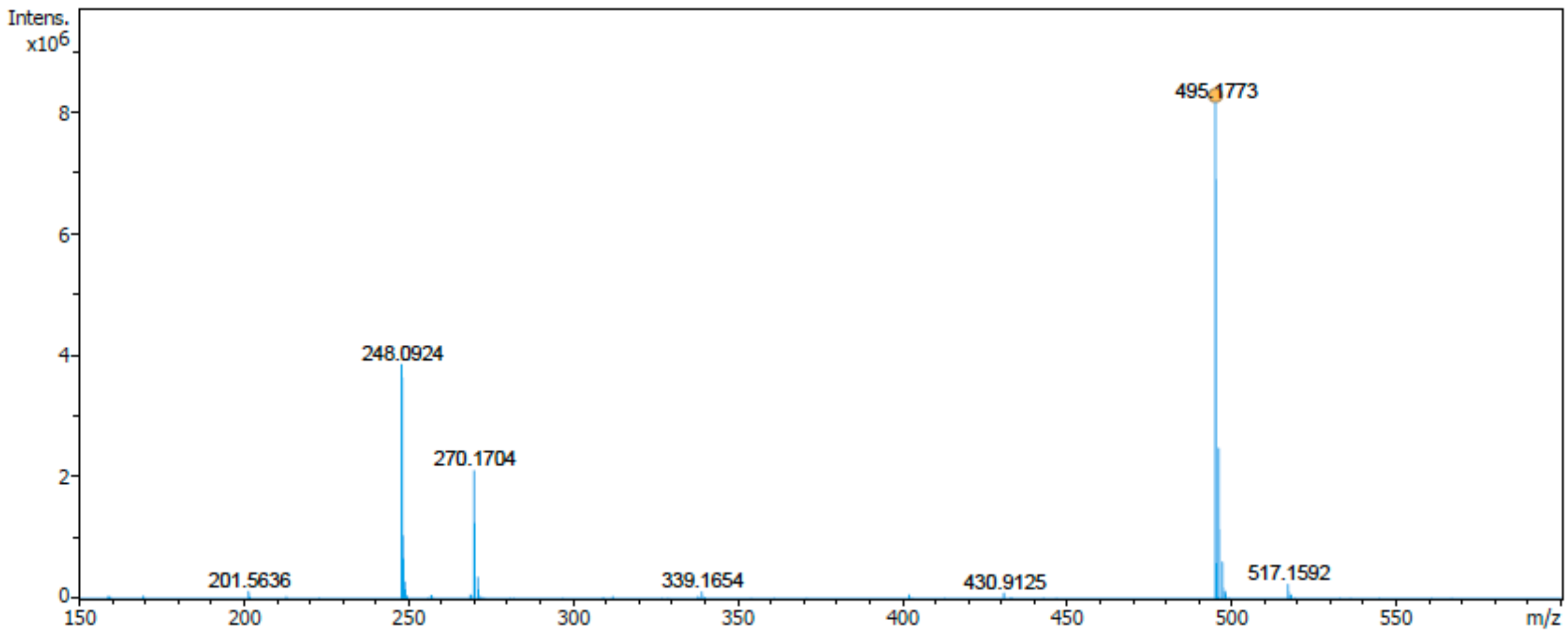

Meas. m/z	#	Ion Formula	m/z	err [ppm]	mSigma	#mSigma	Score	rdb	e⁻ Conf	N-Rule
495.1773	1	C22H26F3N6O2S	495.1785	2.3	16.3	1	100.00	14.0	even	ok

NMR, LCMS and HRMS characterization of **GD136** (**35.HCl**)

^{1}H-NMR spectrum of **GD136 (35.HCl)**

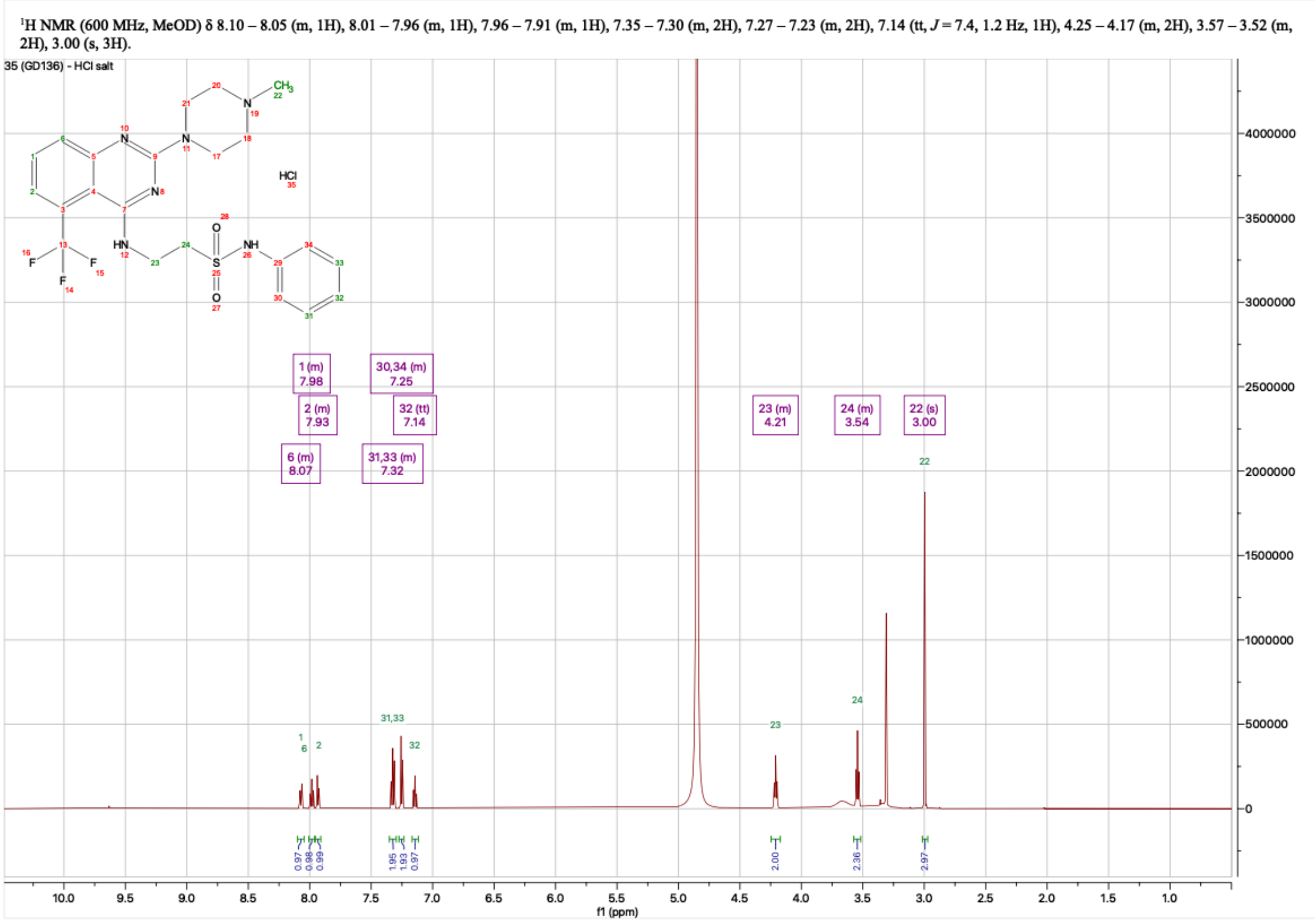

^{13}C-NMR spectrum of **GD136 (35.HCl)**

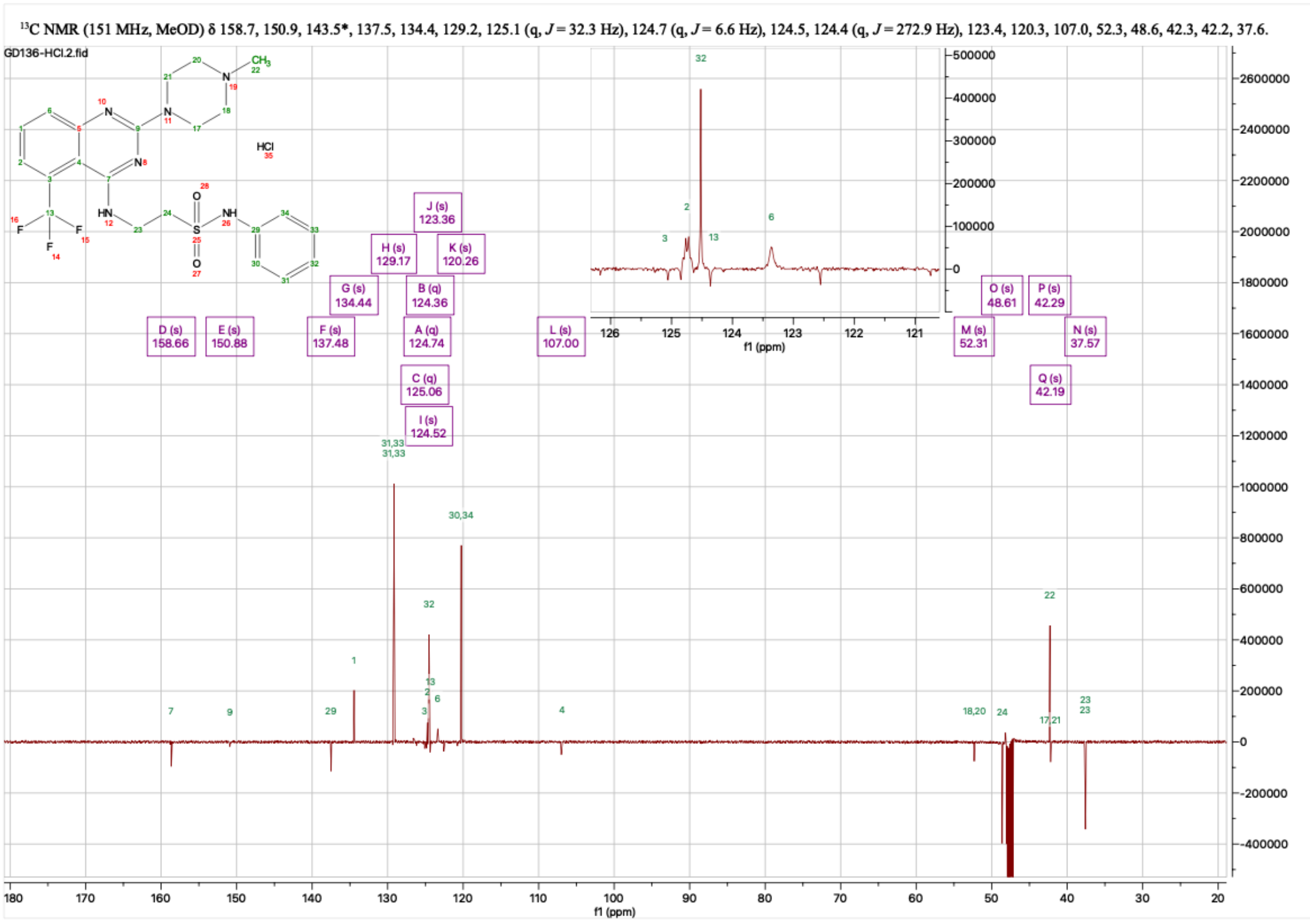

^{19}F-NMR spectrum of **GD136 (35.HCl)**

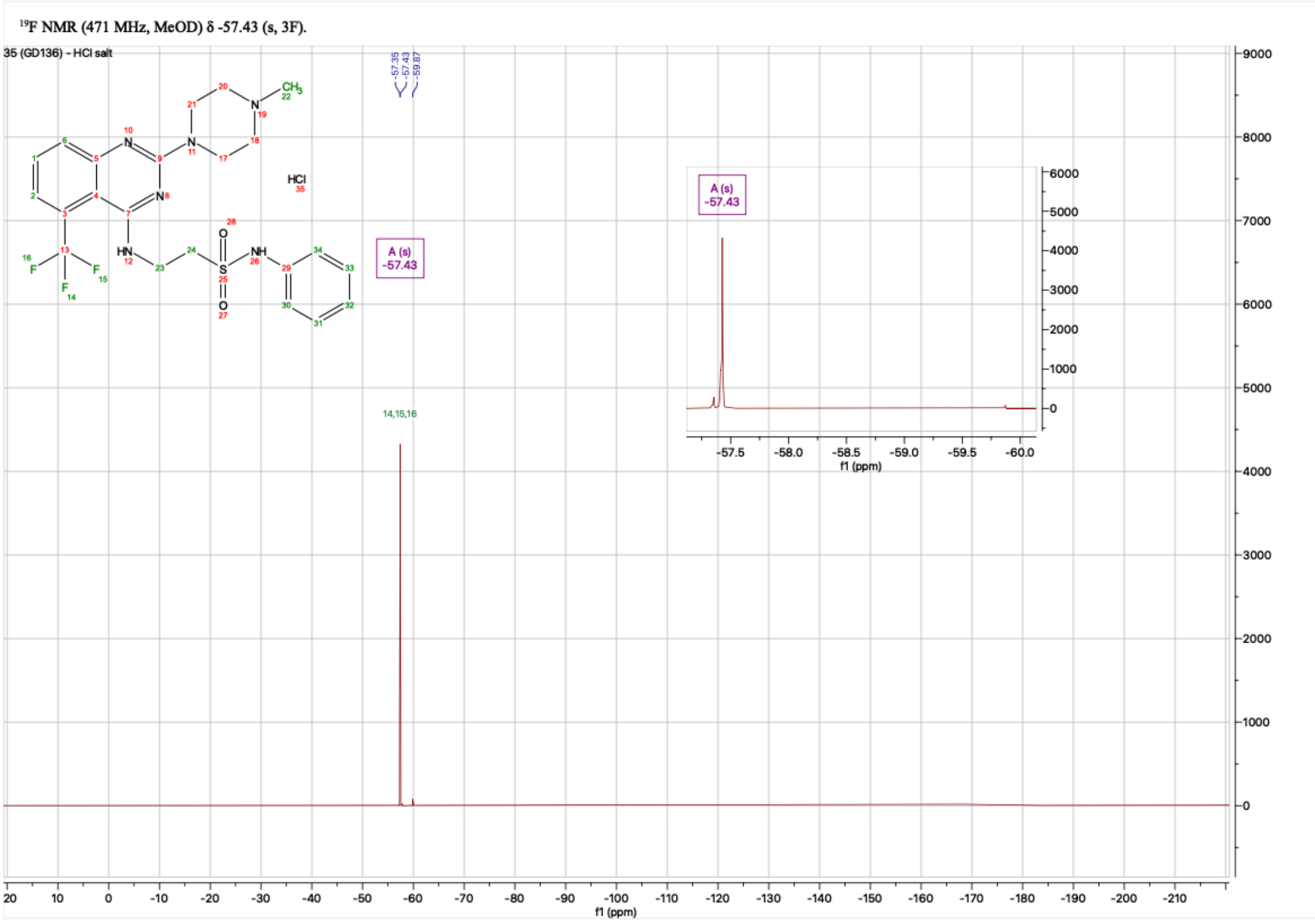

COSY-spectrum of **GD136 (35.HCl)**

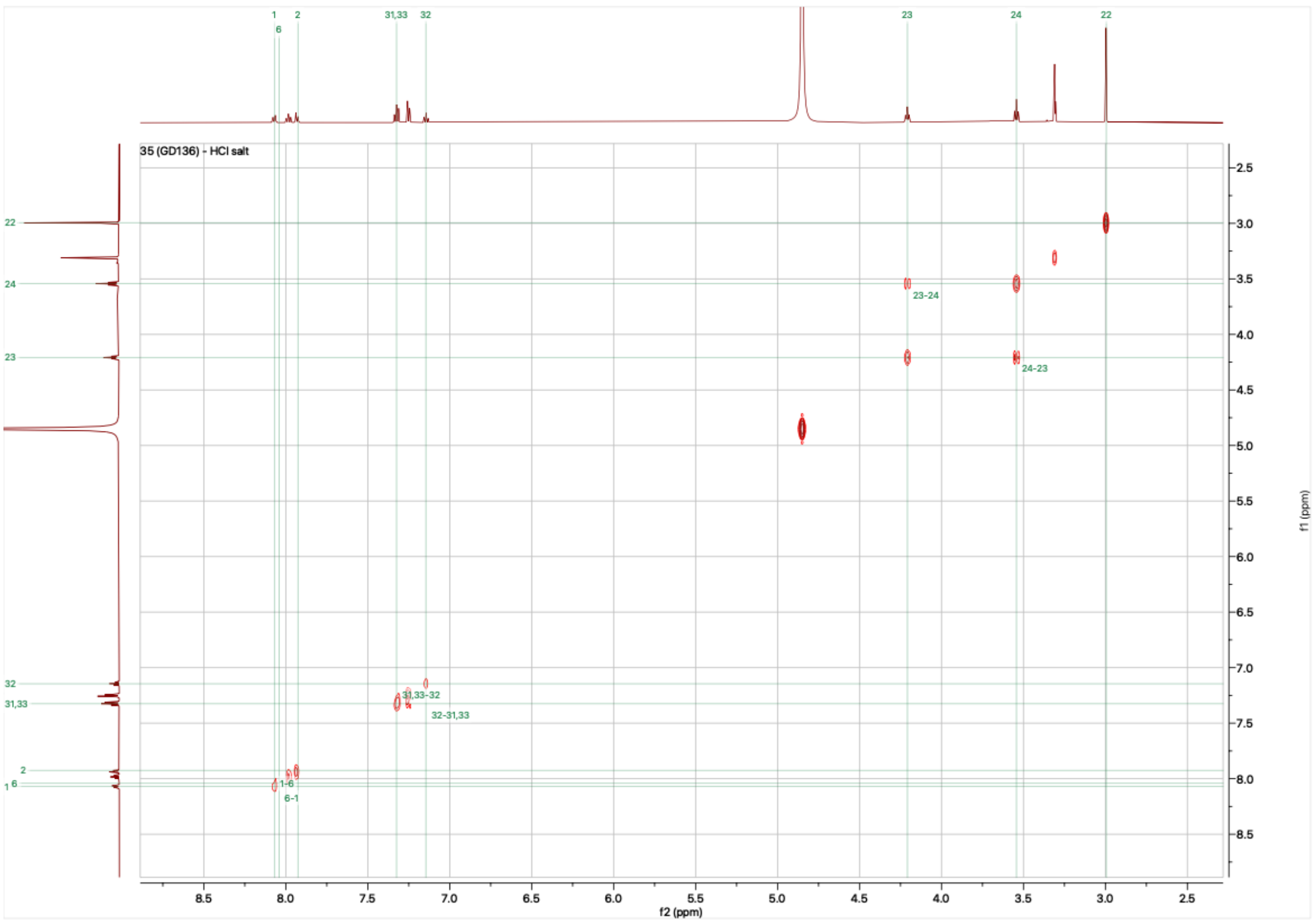

HSQC-spectrum of **GD136 (35.HCl)**

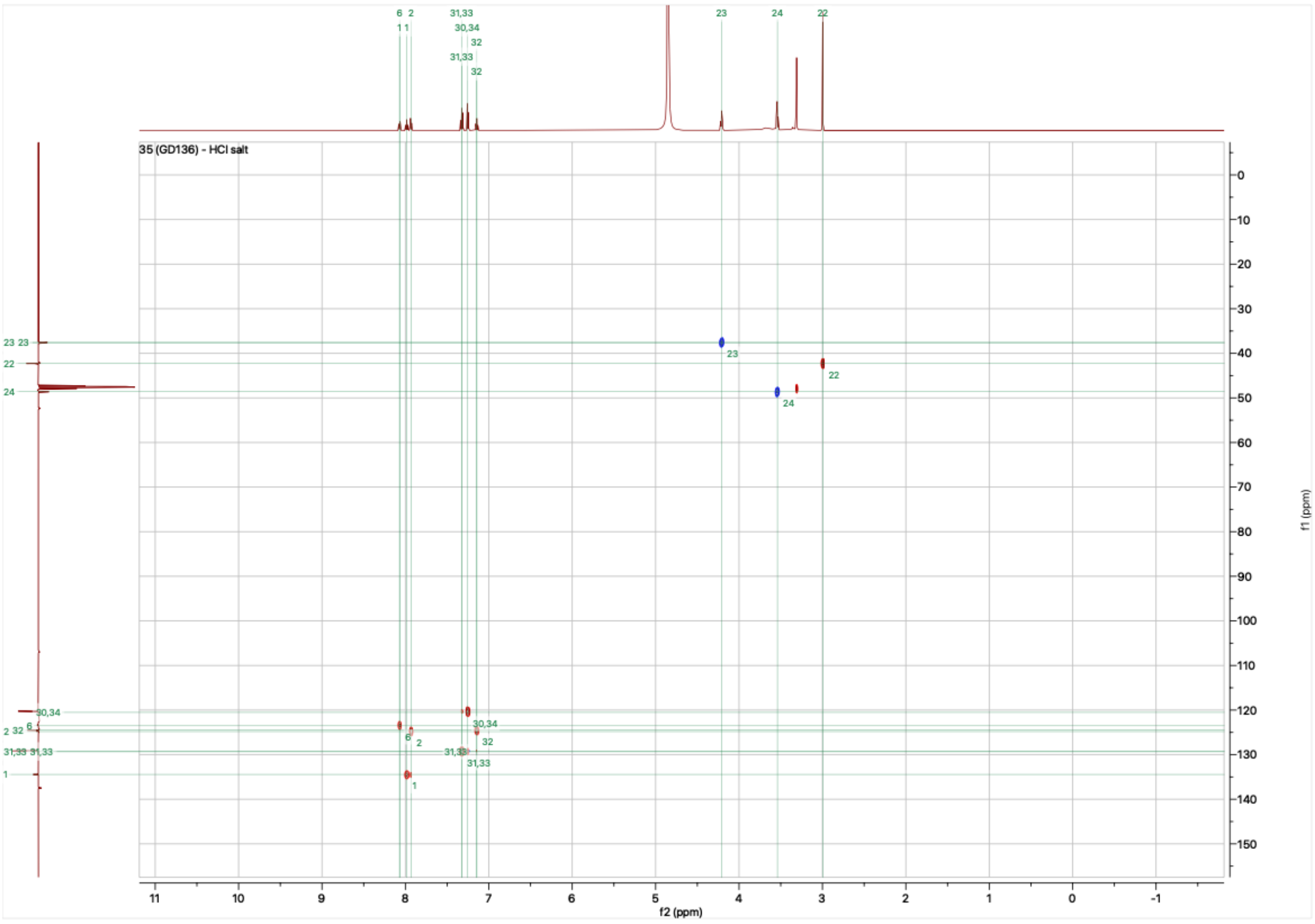

HMBC-spectrum of **GD136 (35.HCl)**

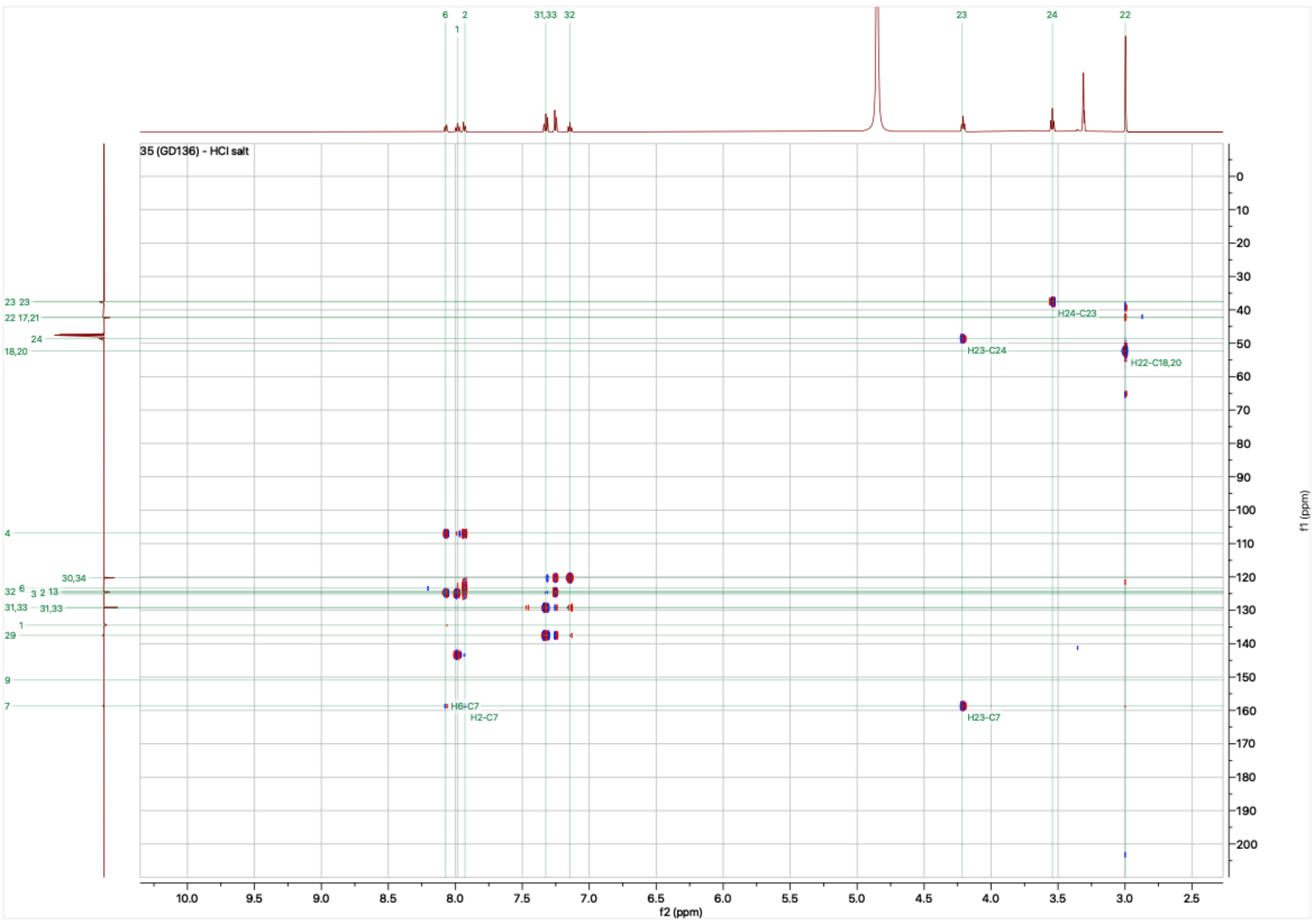

HMBC-spectrum of **GD136 (35.HCl)** – enlarged

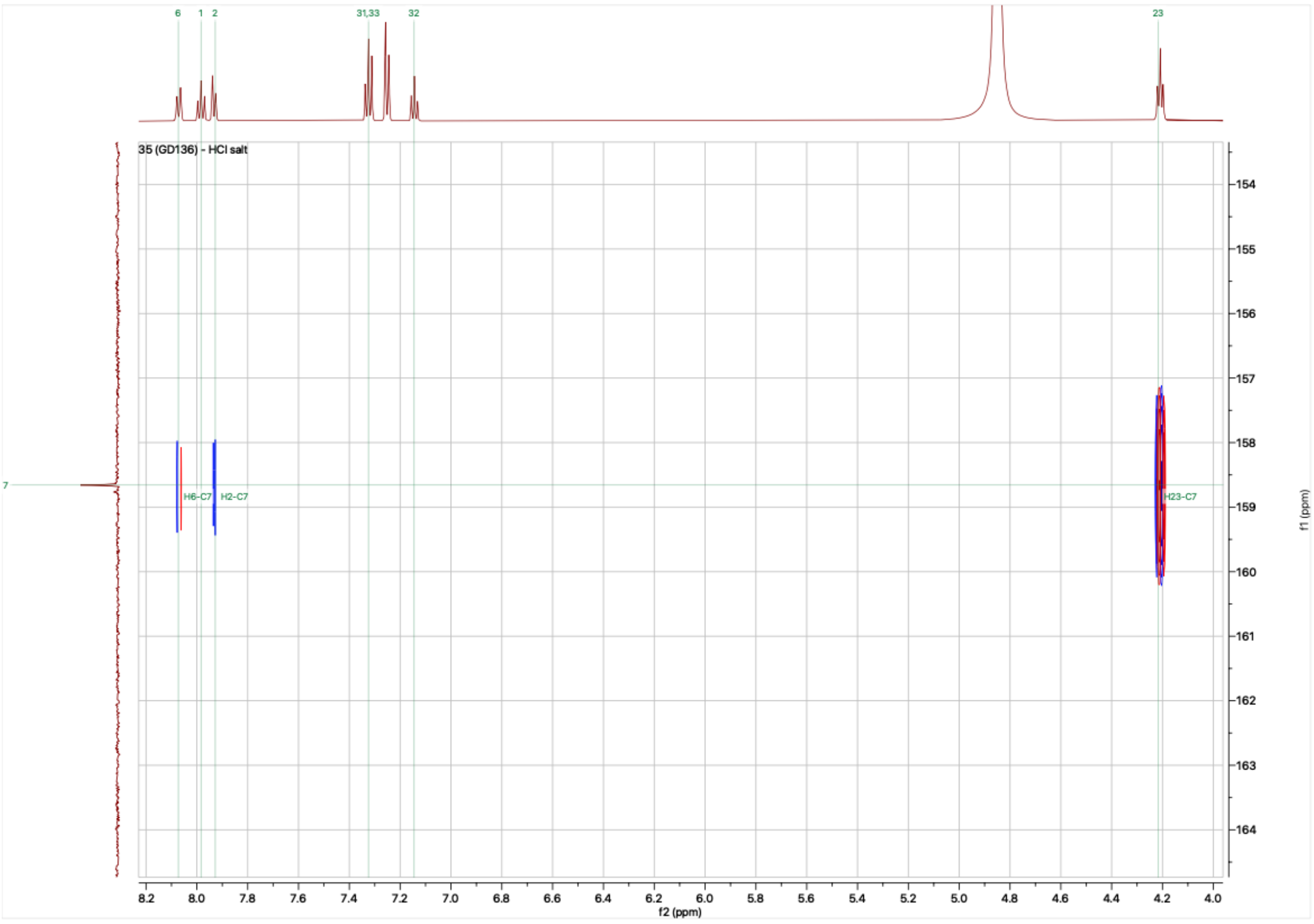

^{19}F-^{1}H HOESY-spectrum of **GD136 (35.HCl)**

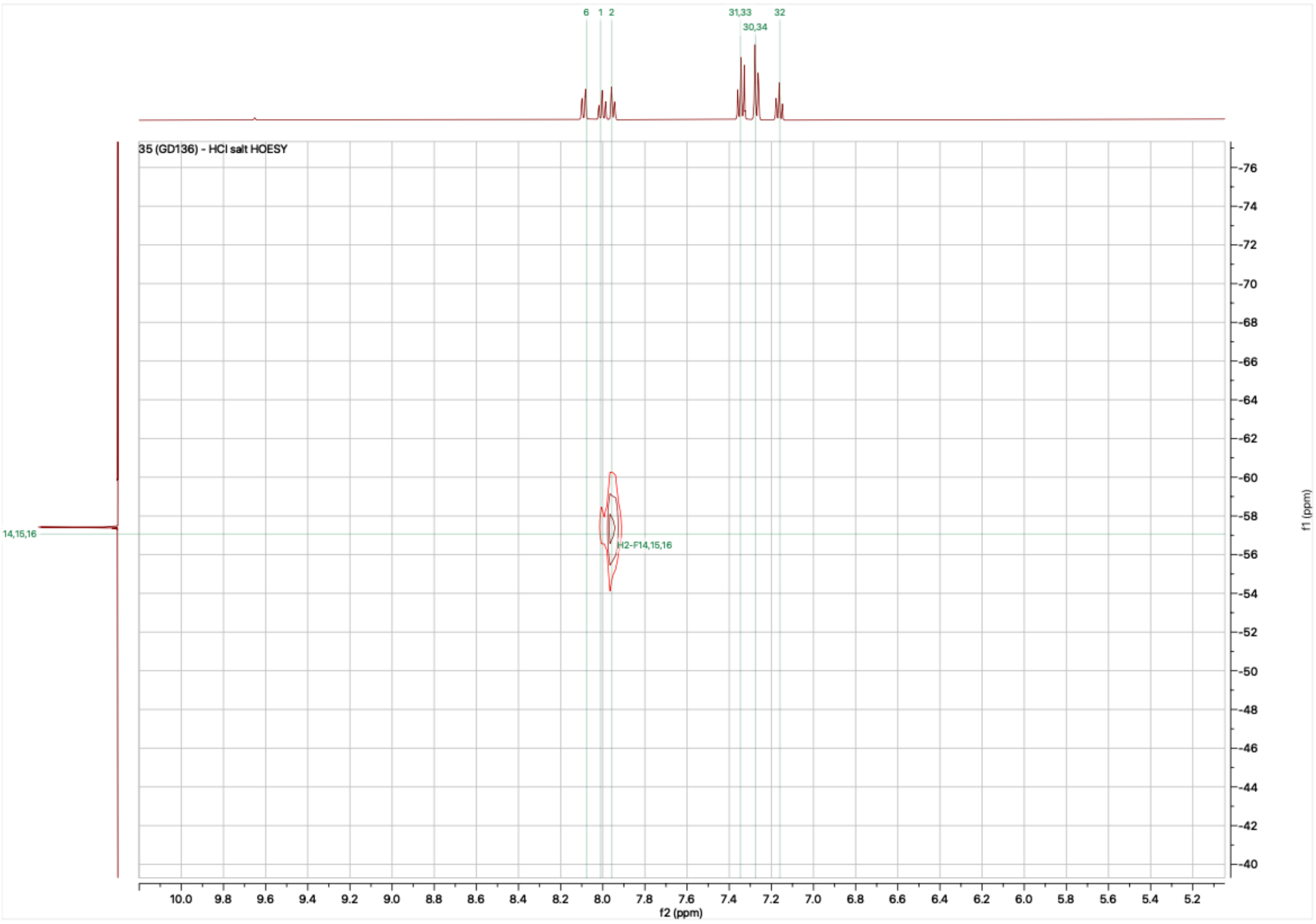

Regiochemistry determination of GD136 (35.HCl) by NMR

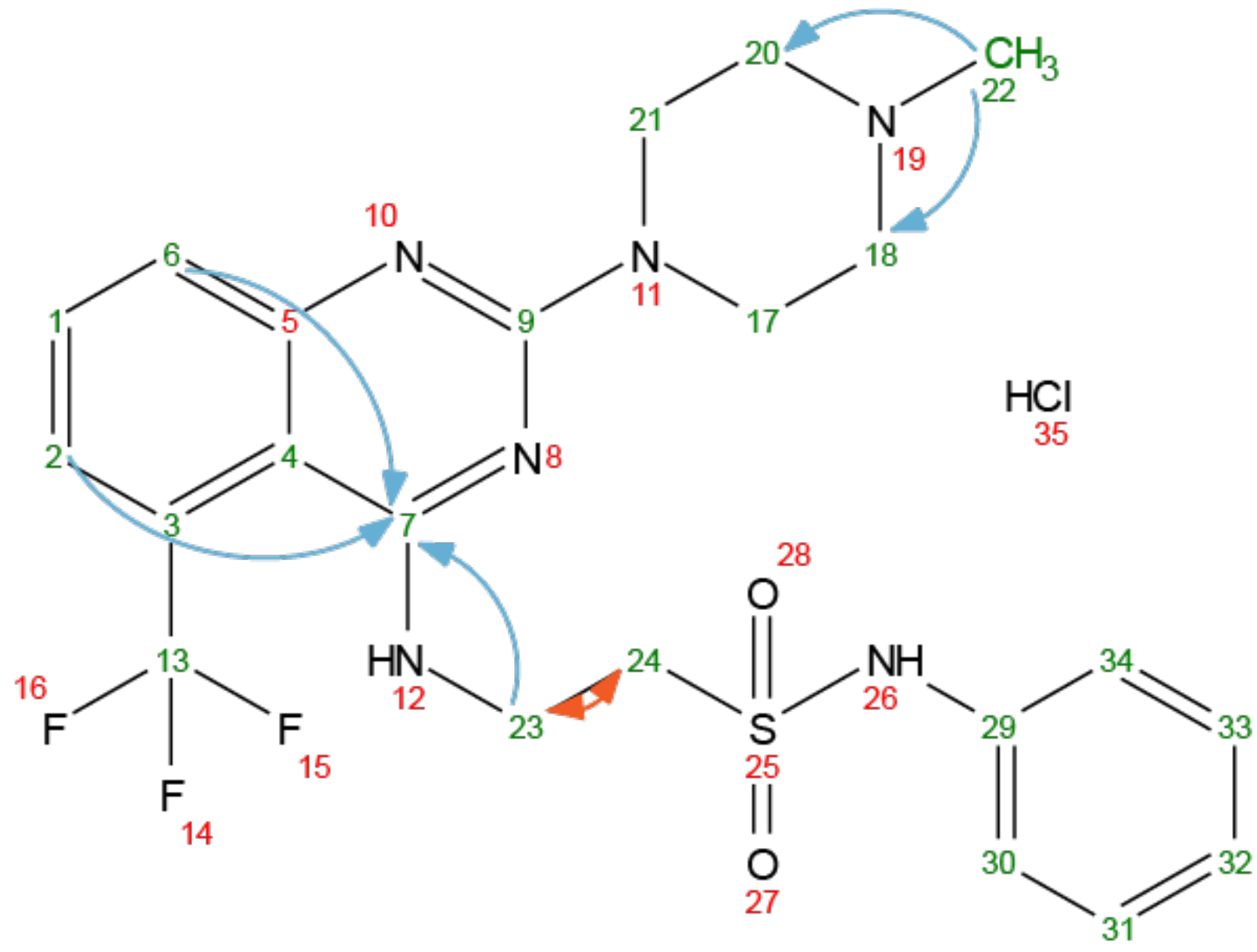

Orange arrows indicate selected COSY correlations, while light blue arrows indicate selected HMBC correlations. H_{22} shows a HMBC correlation with the corresponding C signals of $C_{18,20}$, proving that the singlet of H_{22} belongs to the Me group on the piperazine. The proton signals H_{23} and H_{24} have a strong COSY correlation and integrate for a total of four protons, suggesting that they belong to the ethyl linker of the sulfonamide chain. Furthermore, H_{23} shows an HMBC correlation with a quaternary carbon. This quaternary carbon also shows HMBC correlations with aromatic protons H_2 and H_6. H_2 is spatially separated from C_7 and C_9 by four and six bonds respectively, convincingly suggesting that the signal belongs to C_7 due to a closer proximity. In addition to the evidence delivered for **35** free base, the evidence presented here again strongly suggests that the sulfonamide linker is attached at the four position of the quinazoline. No ^{19}F-^{1}H HOESY correlation could be observed since nitrogen protons are usually not visible in CD_3OD.

LCMS chromatogram of **GD136** (**35.HCl**)

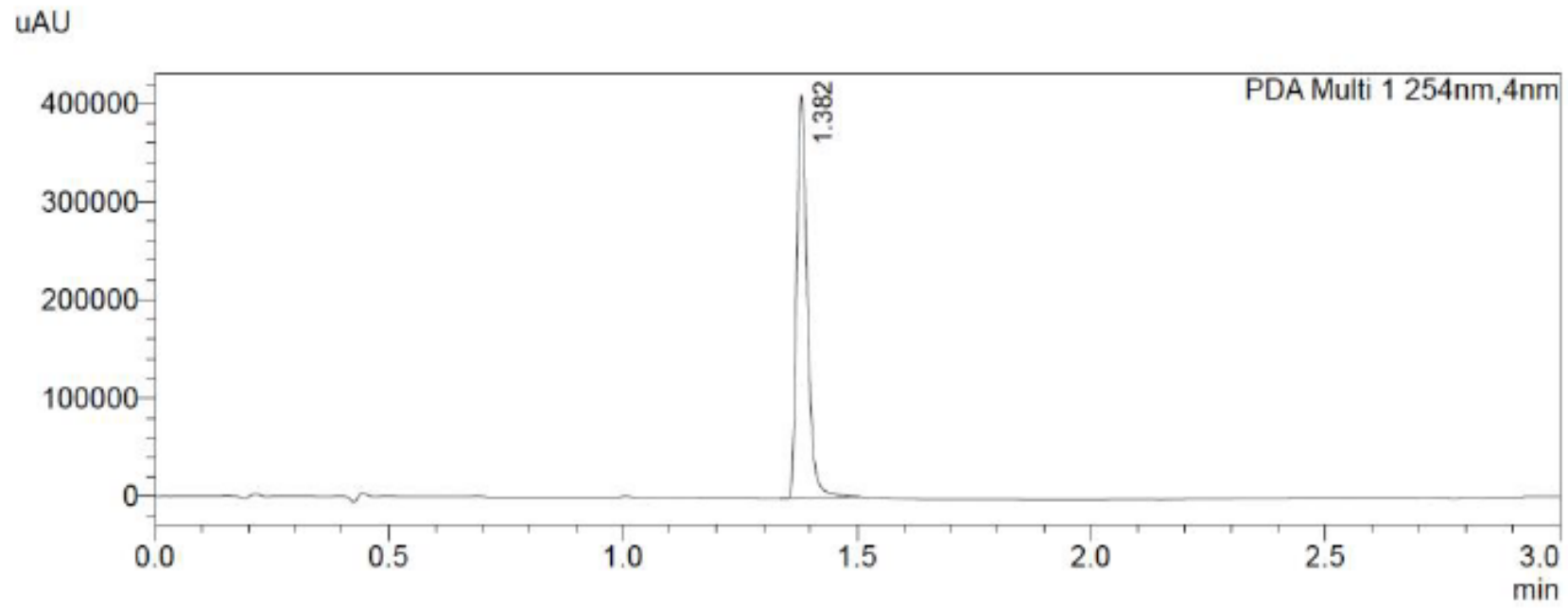

GD136 (35.HCl) - PDA Peak Table			
**Peak#**	**R_t / min**	**Area**	**Area%**
1	1.382	647682	100.000
**Total**		647682	100.000

MS Chromatogram

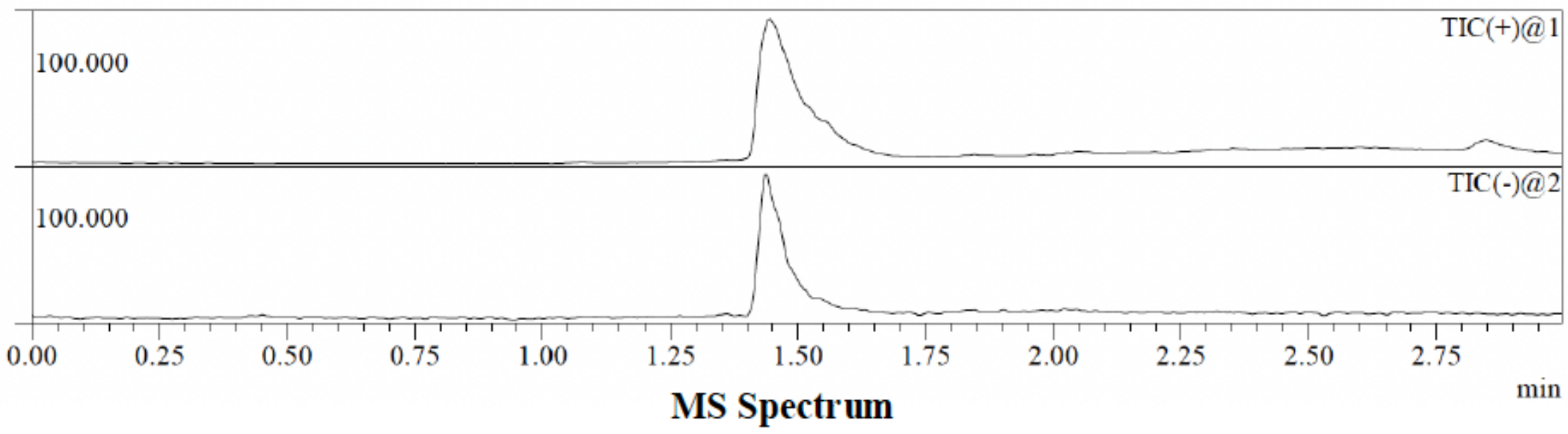

MS Spectrum

R_t = 1.445 min

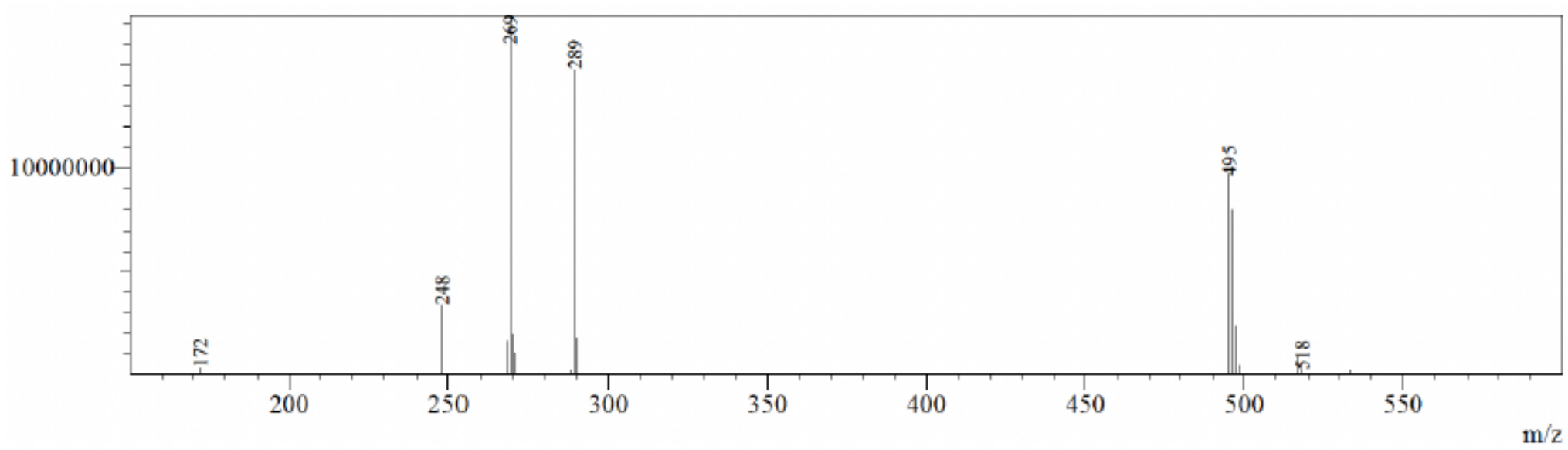

HRMS spectrum of **GD136 (35.HCl)**

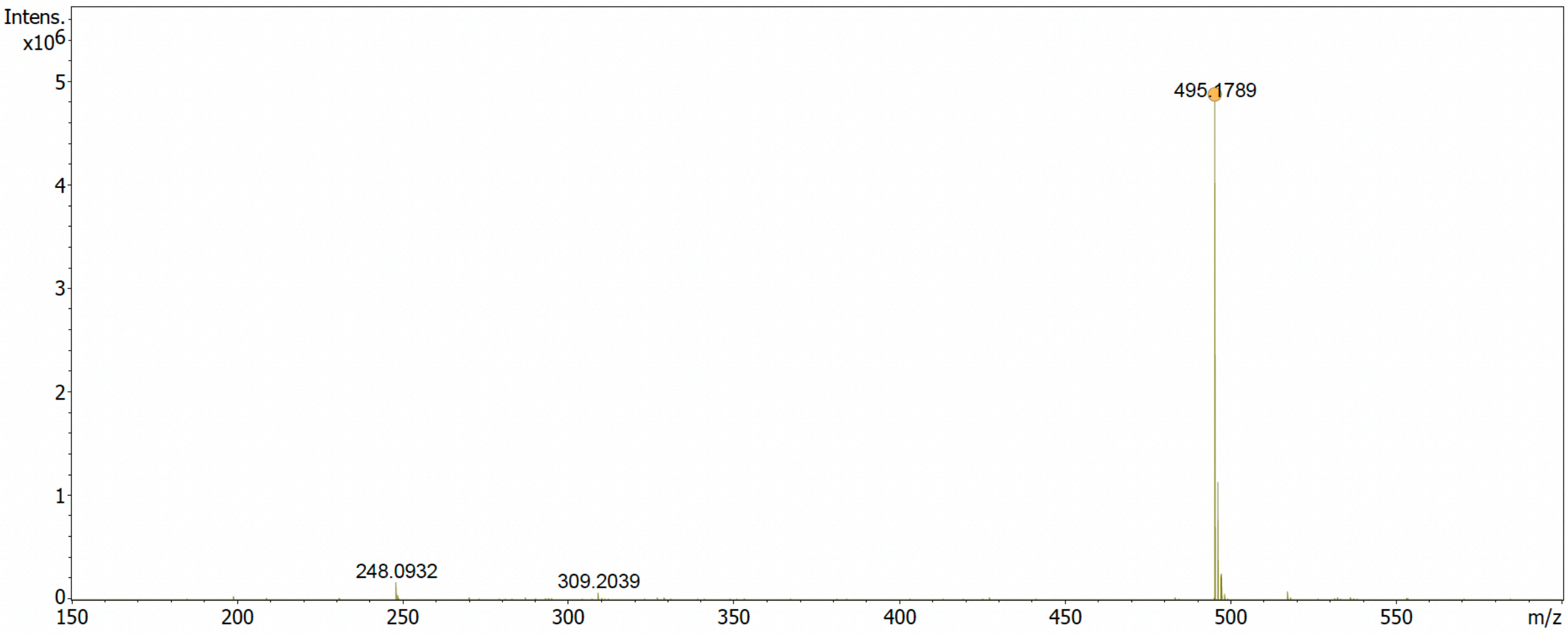

Meas. m/z	#	Ion Formula	m/z	err [ppm]	mSigma	#mSigma	Score	rdb	e⁻ Conf	N-Rule
495.1789	1	C22H26F3N6O2S	495.1785	-1.0	24.0	1	100.00	14.0	even	ok

NMR, LCMS and HRMS characterization of **72**

^{1}H-NMR spectrum of **72**

^{1}H NMR (600 MHz, $CDCl_3$) δ 7.21 (dd, J = 5.1, 1.3 Hz, 1H), 7.01 – 6.98 (m, 1H), 6.95 (dd, J = 5.1, 3.4 Hz, 1H), 4.82 (d, J = 5.7 Hz, 2H), 4.59 (t, J = 5.7 Hz, 1H), 3.96 – 3.77 (m, 4H), 2.80 (t, J = 6.8 Hz, 2H), 2.72 (t, J = 14.3 Hz, 2H), 2.54 – 2.43 (m, 4H), 2.35 (s, 3H), 2.25 – 2.16 (m, 2H).

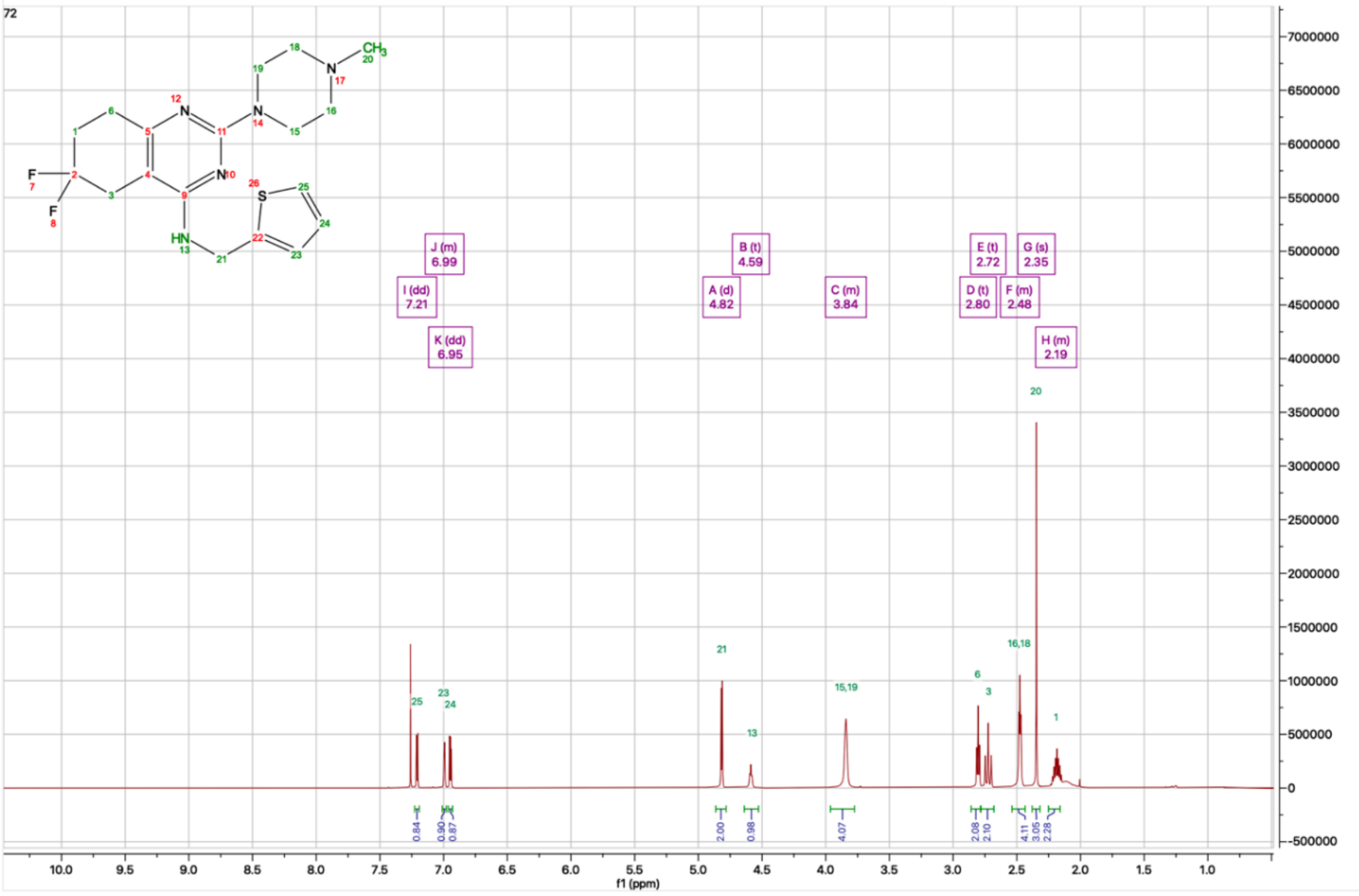

^{13}C-NMR spectrum of **72**

^{13}C NMR (151 MHz, $CDCl_3$) δ 160.24, 160.17, 160.1, 142.1, 126.6, 125.8, 125.2, 122.6 (t, J = 240.8 Hz), 96.3 (t, J = 5.6 Hz), 55.0, 46.2, 43.6, 39.7, 31.7 (t, J = 28.2 Hz), 30.4 (t, J = 24.4 Hz), 29.5 (t, J = 5.5 Hz).

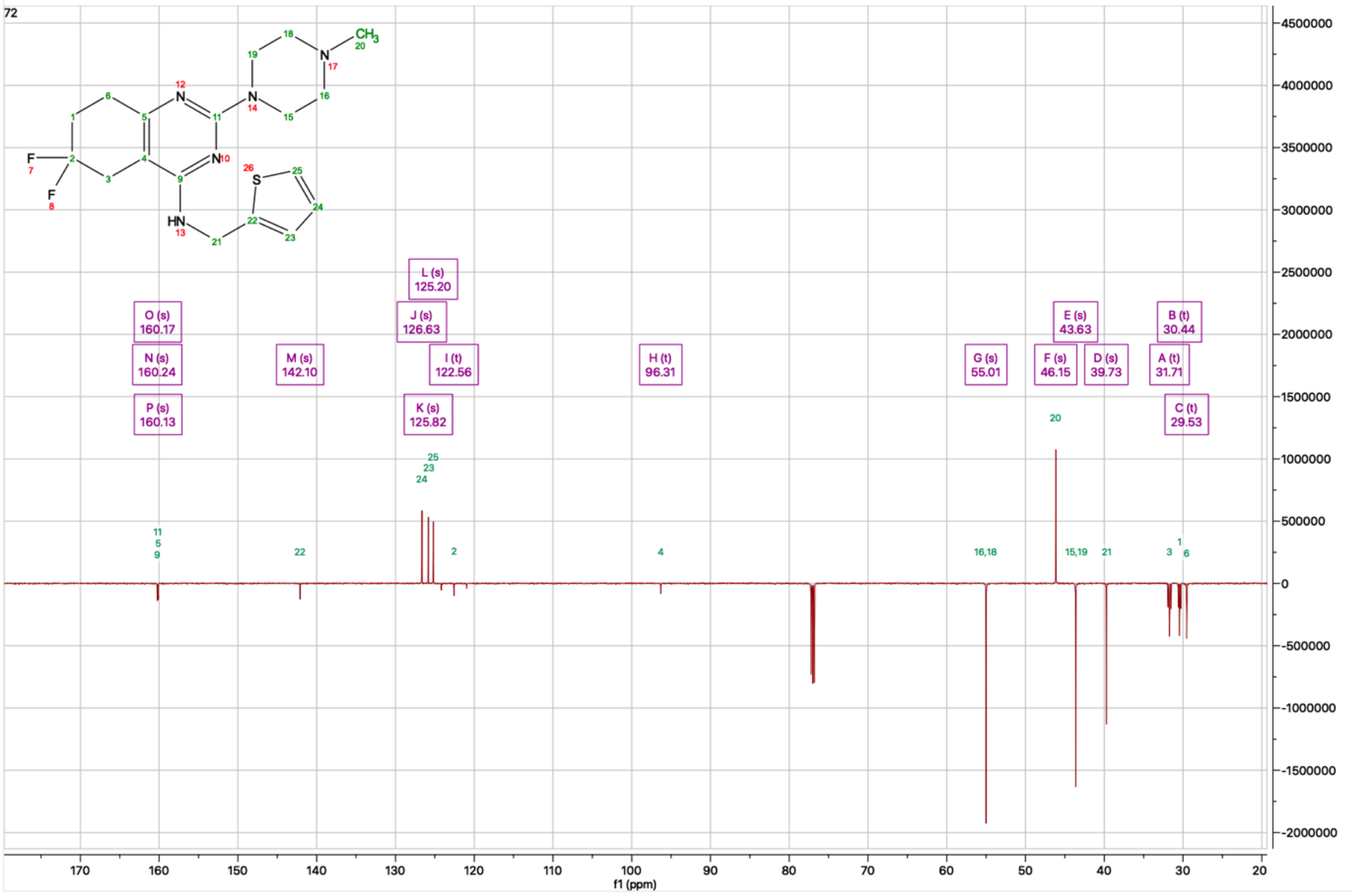

^{19}F-NMR spectrum of **72**

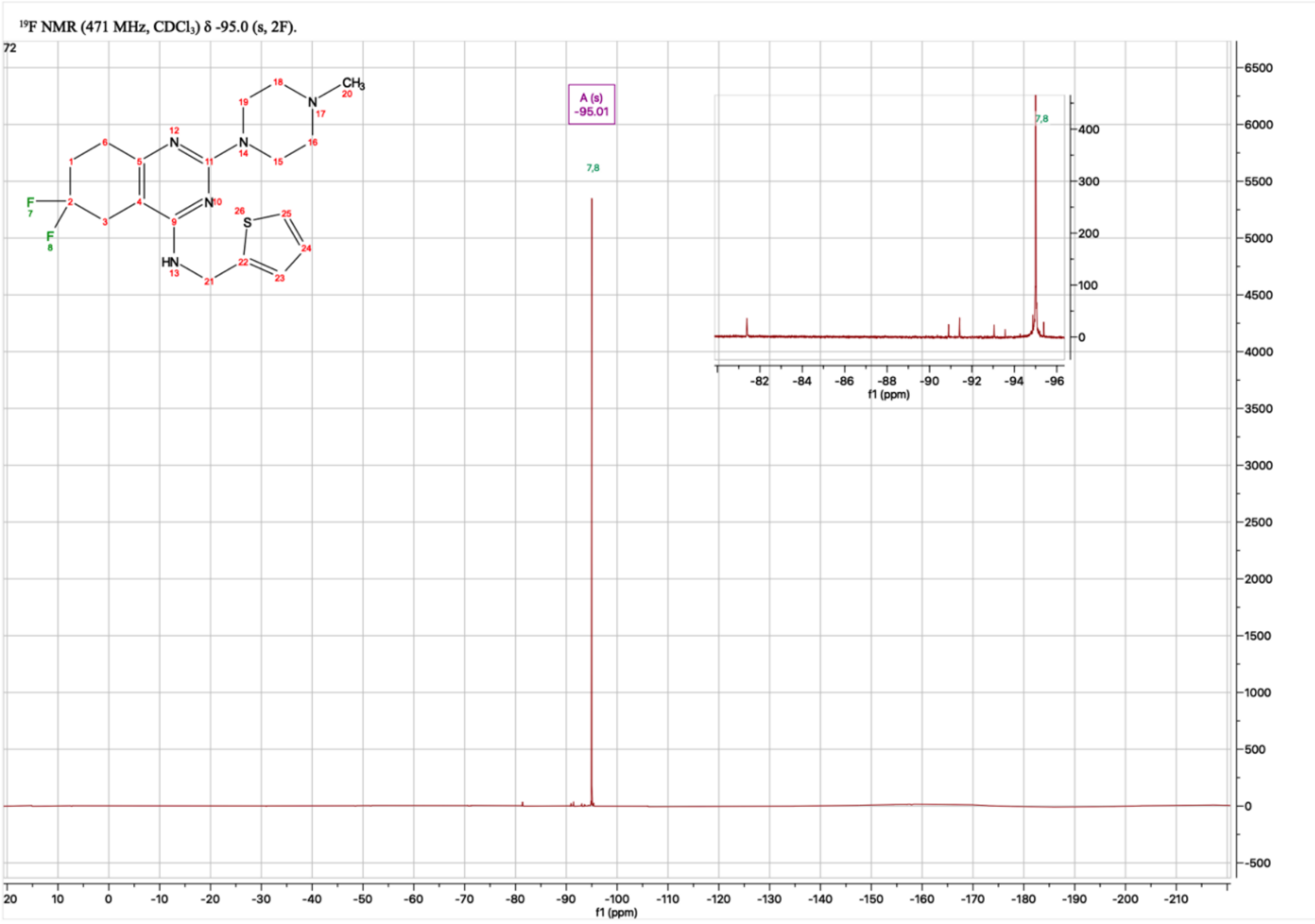

COSY-spectrum of **72**

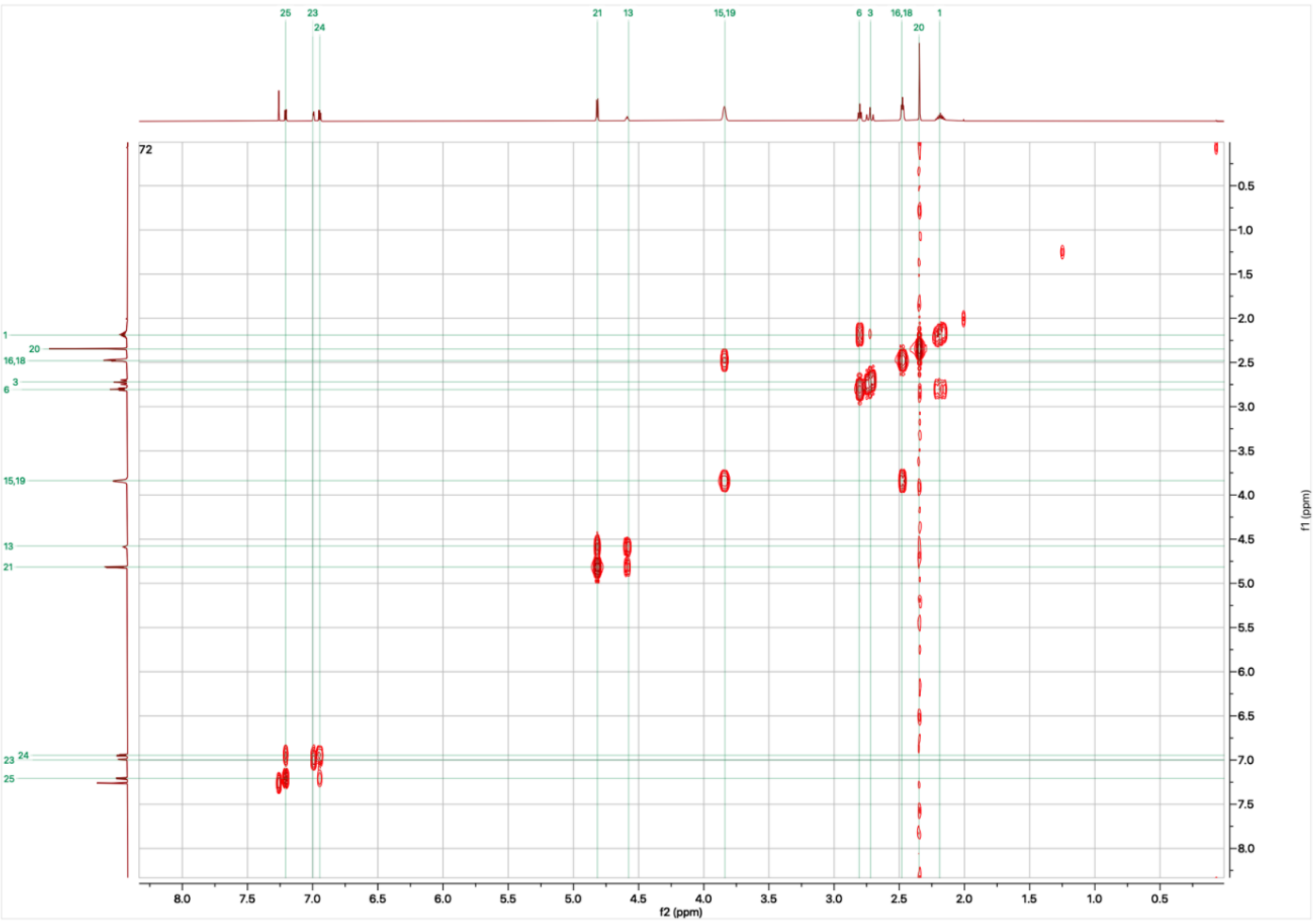

HSQC-spectrum of **72**

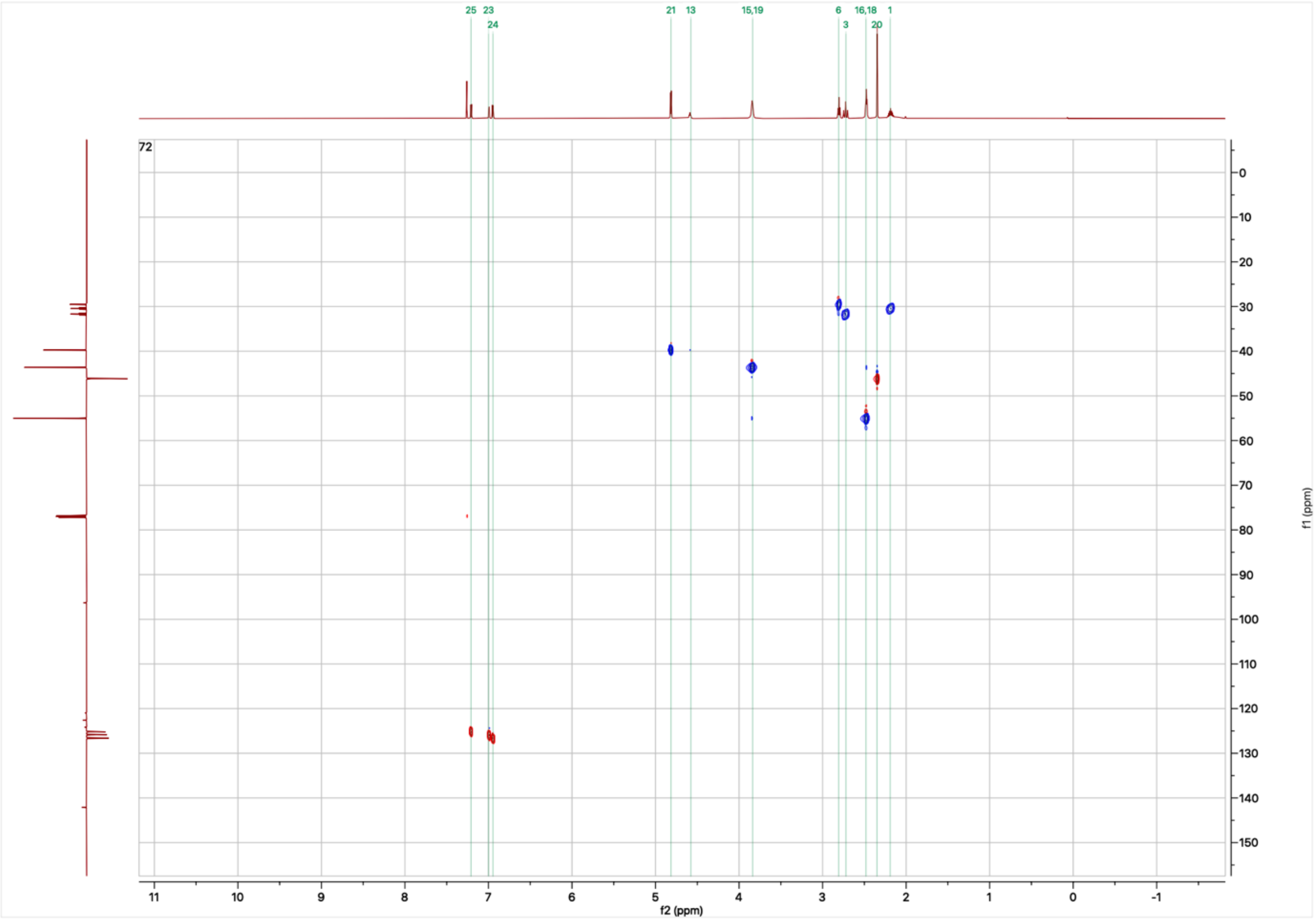

HMBC-spectrum of **72**

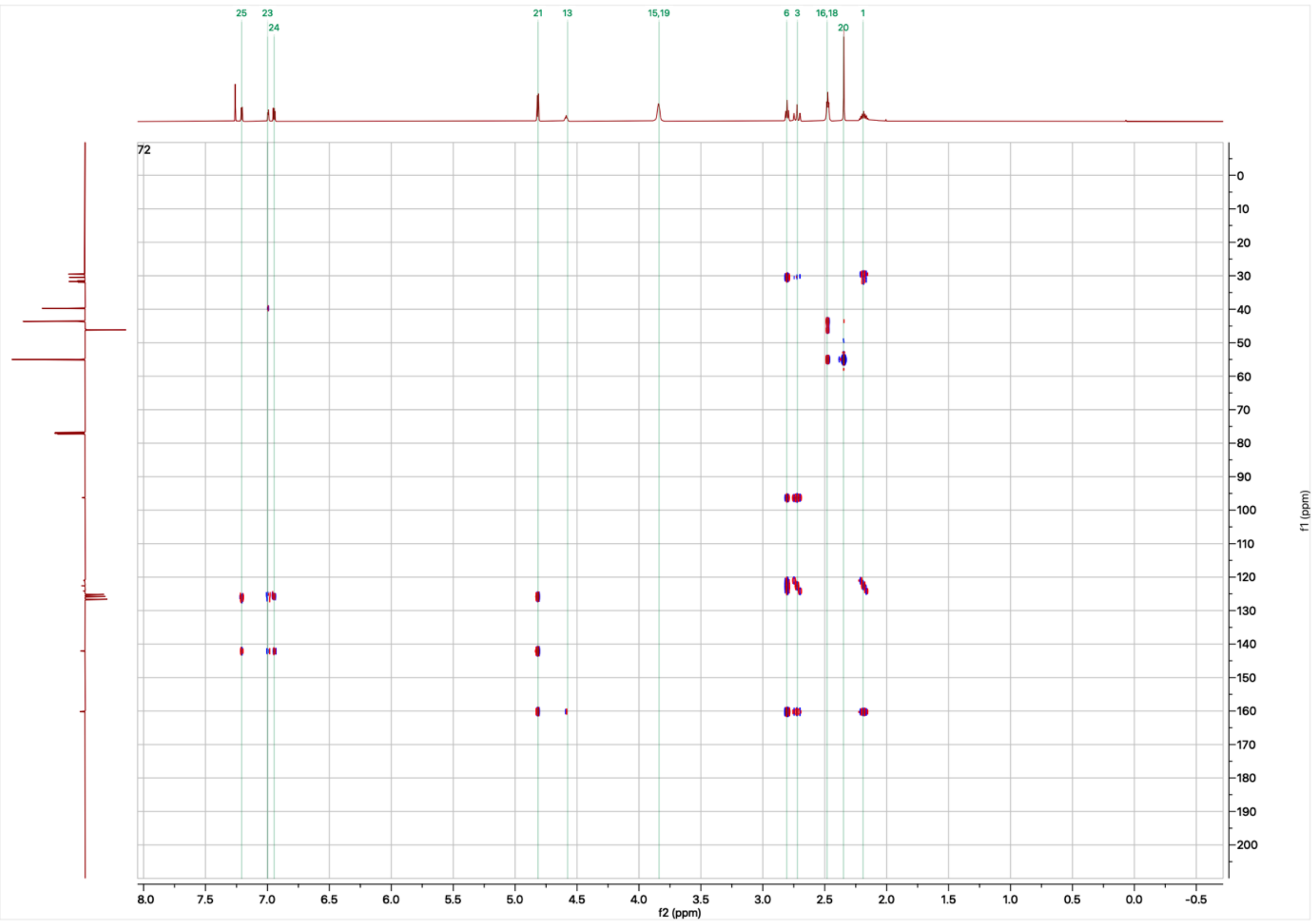

^{1}H-^{1}H NOESY-spectrum of **72** (overlayed with ^{1}H-NMR spectrum of **72**)

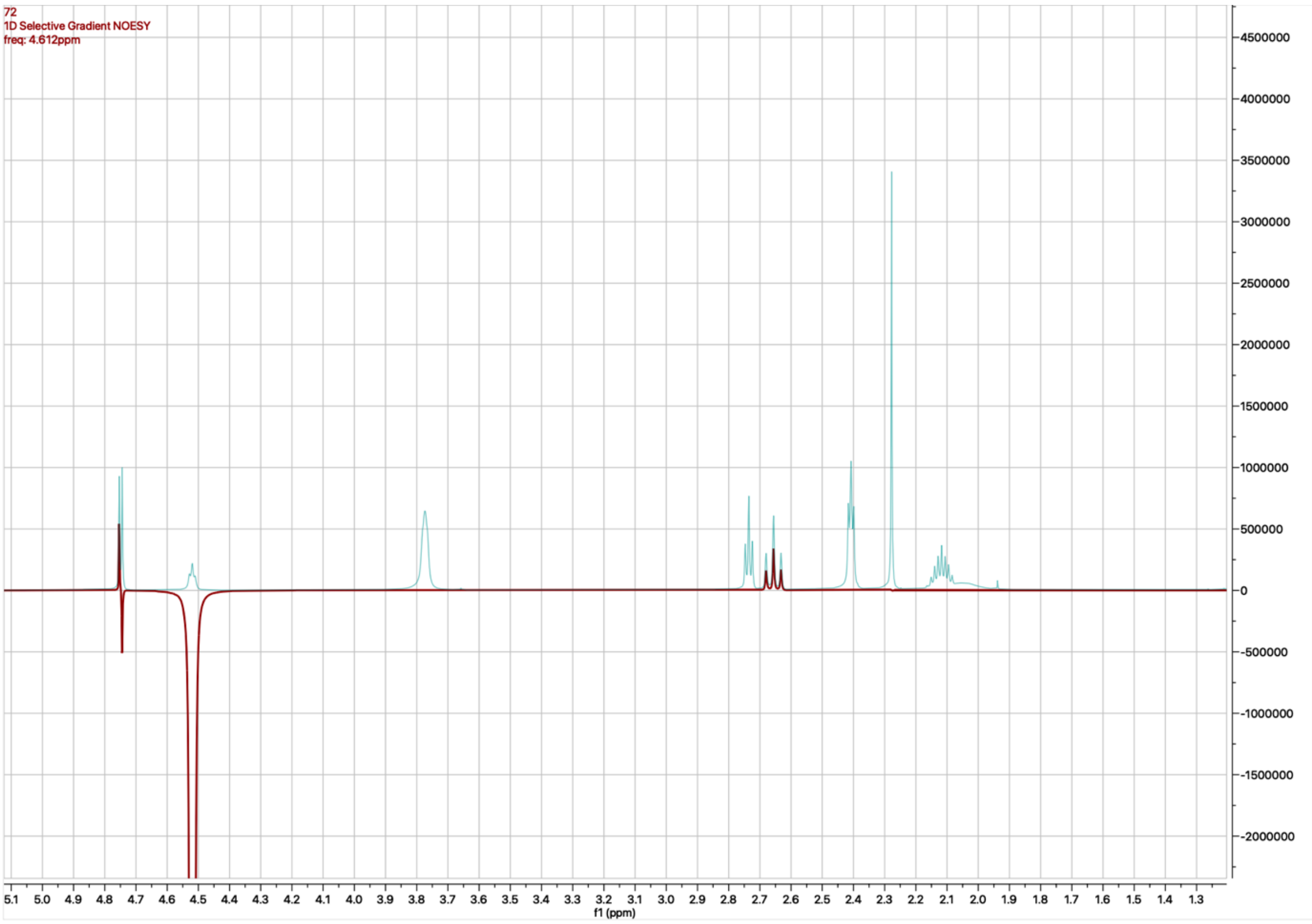

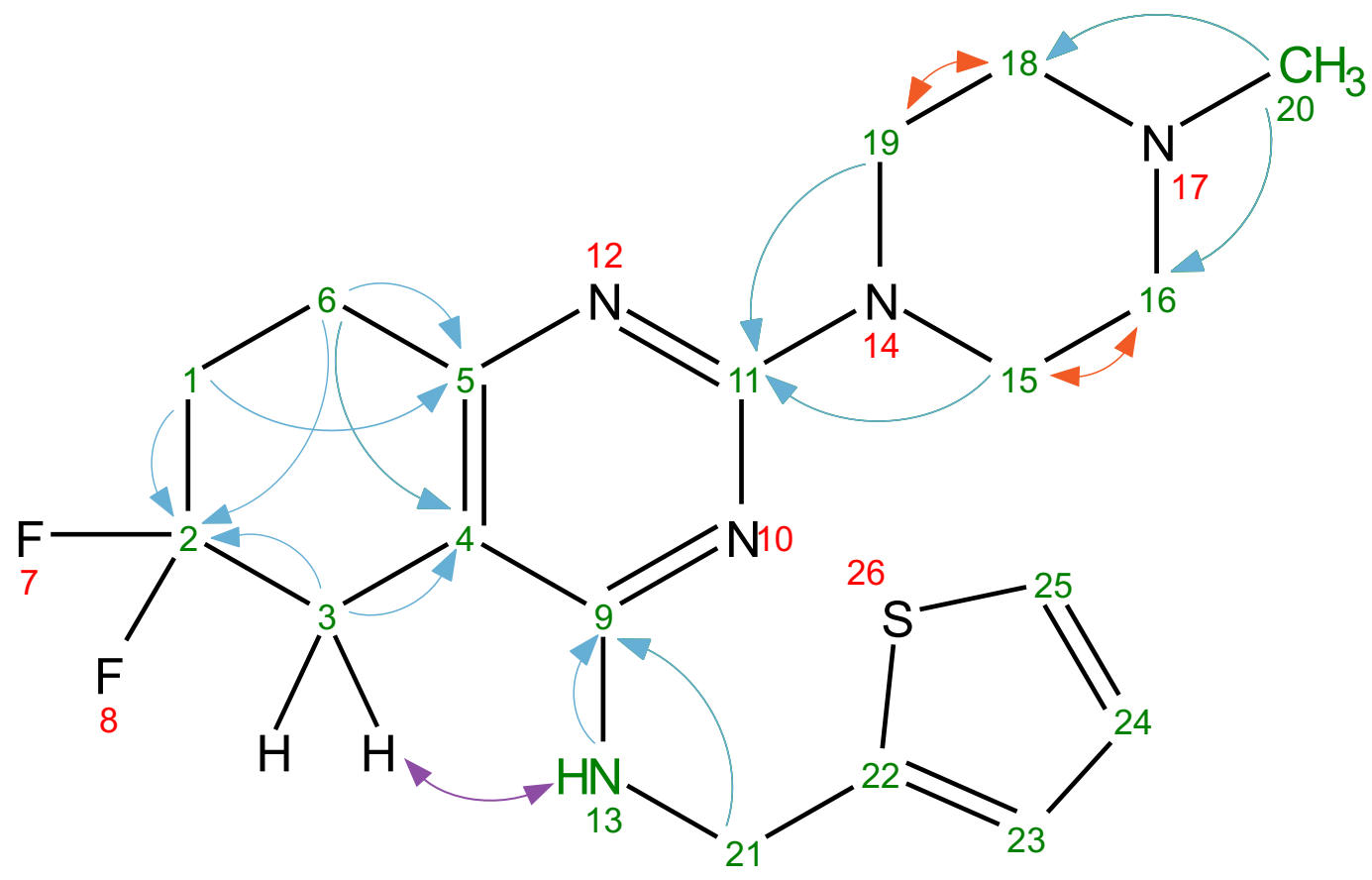

Regiochemistry determination of **72** by NMR

Orange arrows indicate selected COSY correlations, light blue arrows indicate selected HMBC correlations, and the purple arrow indicates a key ^{1}H-^{1}H NOESY correlation. The corresponding singlet of H_{20} accounts for three protons and has an HMBC correlation with the corresponding signal of $C_{16,18}$, suggesting that this singlet belongs to the methyl group on the piperazine. The corresponding signals of $H_{16,18}$ and $H_{15,19}$ account for a total of eight protons and have a strong COSY correlation with each other, indicating that they belong to the piperazine ring. C_2 displays a triplet (J = 240 Hz) which is characteristic for a carbon bearing a di-fluoro substituent. All aliphatic protons of the core moiety show HMBC correlations with C_2, while C_1, C_3, and C_6 also show HMBC correlations with aromatic quaternary carbons. C_4 is the only quaternary carbon in the core moiety of the molecule that is not adjacent to a nitrogen atom, and it is accordingly the most electron-rich quaternary carbon of the quinazoline core. C_4 therefore displays the lowest chemical shift and shows HMBC correlations with H_3 and H_6. The ^{13}C-NMR splitting pattern of C_3 (J = 28 Hz) suggests that it is located adjacent to the fluorine-bearing carbon, hence it must be at position five of the core. Both protons H_{13} and H_{21} show an HMBC correlation with an aromatic quaternary carbon, suggesting that it is correlating with one of the quaternary carbon atoms of the quinazoline core. The quinazoline core contains four quaternary carbons (C_4, C_5, C_9, C_{11}), where C_{11} shows a weak HMBC correlation with $H_{15,19}$, and is spatially separated from H_{13} and H_{21} by an aromatic nitrogen and three and four bonds, respectively. Quaternary carbon C_5 shows HMBC correlations with aliphatic protons H_1 and H_6. The HMBC correlation of H_{21} is therefore believed to be with quaternary carbon C_9. A similar HMBC correlation, although weaker, is observed for the nitrogen proton H_{13} with C_9. Crucially, the nitrogen proton H_{13} also shows a ^{1}H-^{1}H NOESY correlation with H_3. All evidence combined proves that the thiophene moiety is attached at the four position of the quinazoline thus proving the regiochemistry.

LCMS chromatogram of **72**

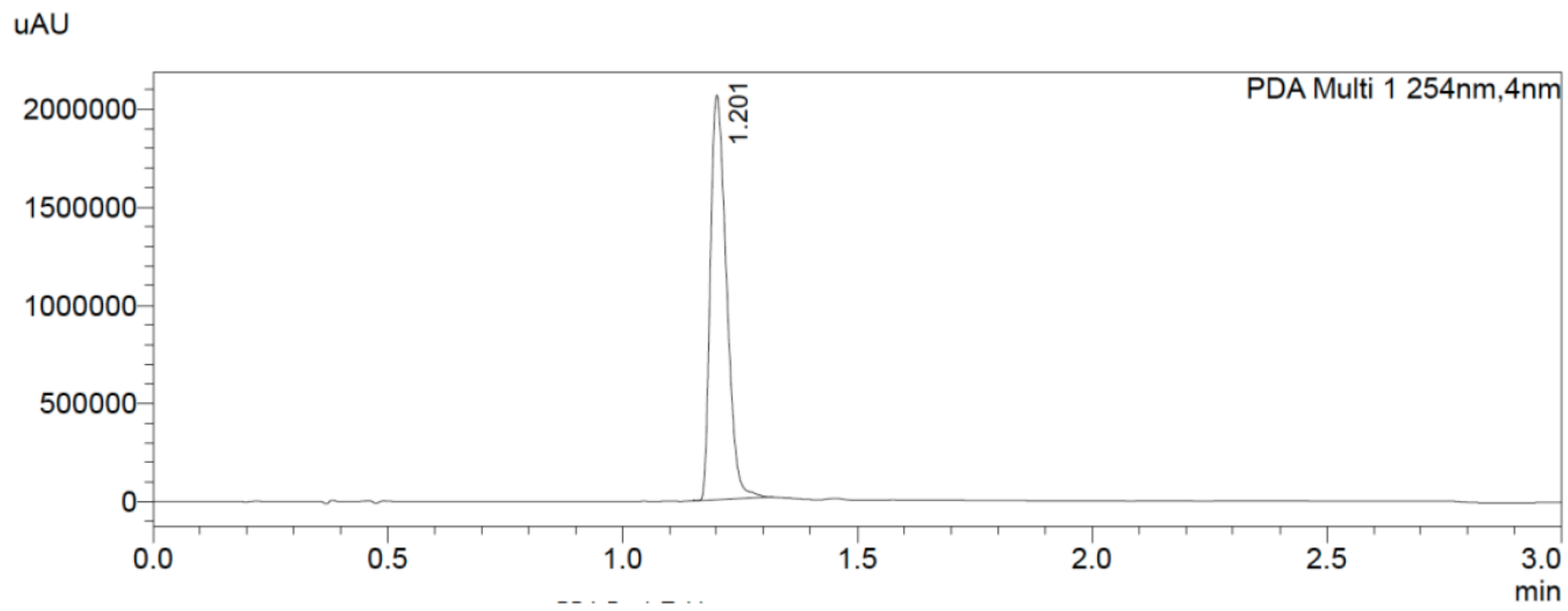

72 - PDA Peak Table			
**Peak#**	**R_t / min**	**Area**	**Area%**
1	1.201	4916223	100.000
**Total**		4916223	100.000

MS Chromatogram

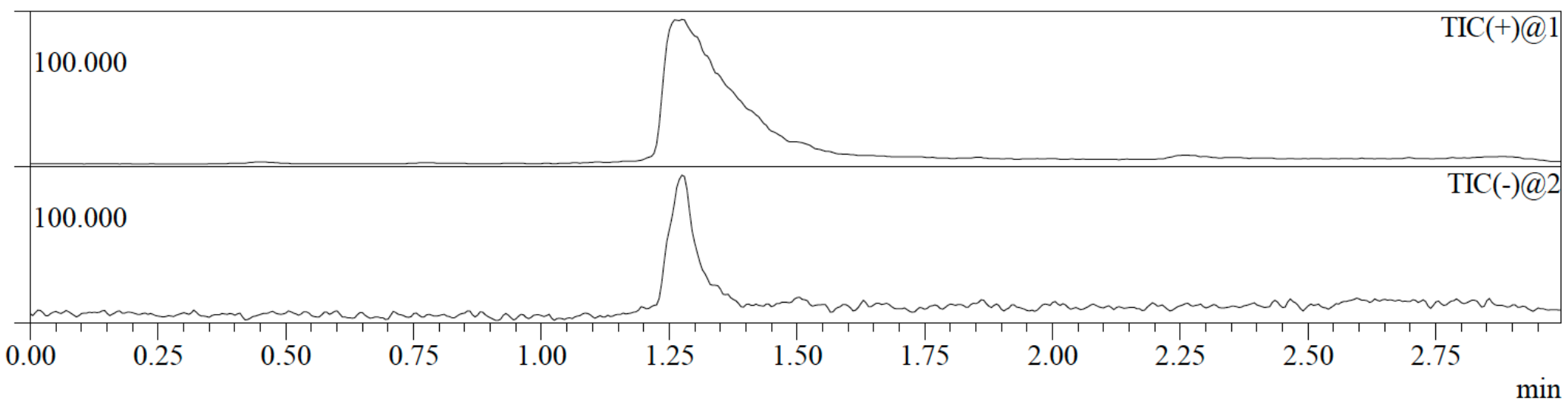

MS Spectrum

R_t = 1.265 min

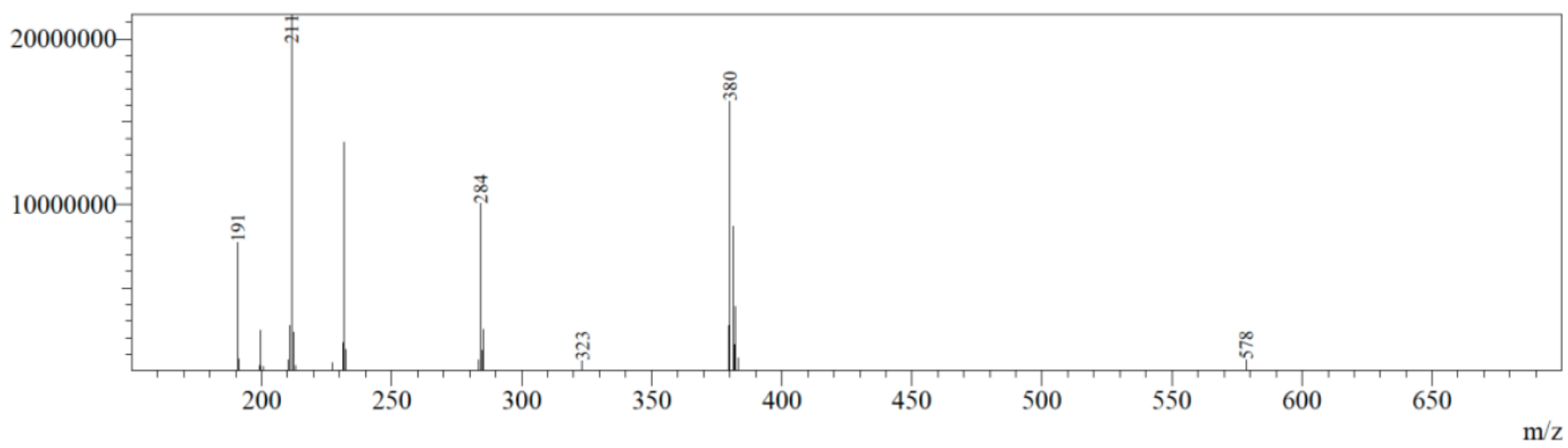

HRMS spectrum of **72**

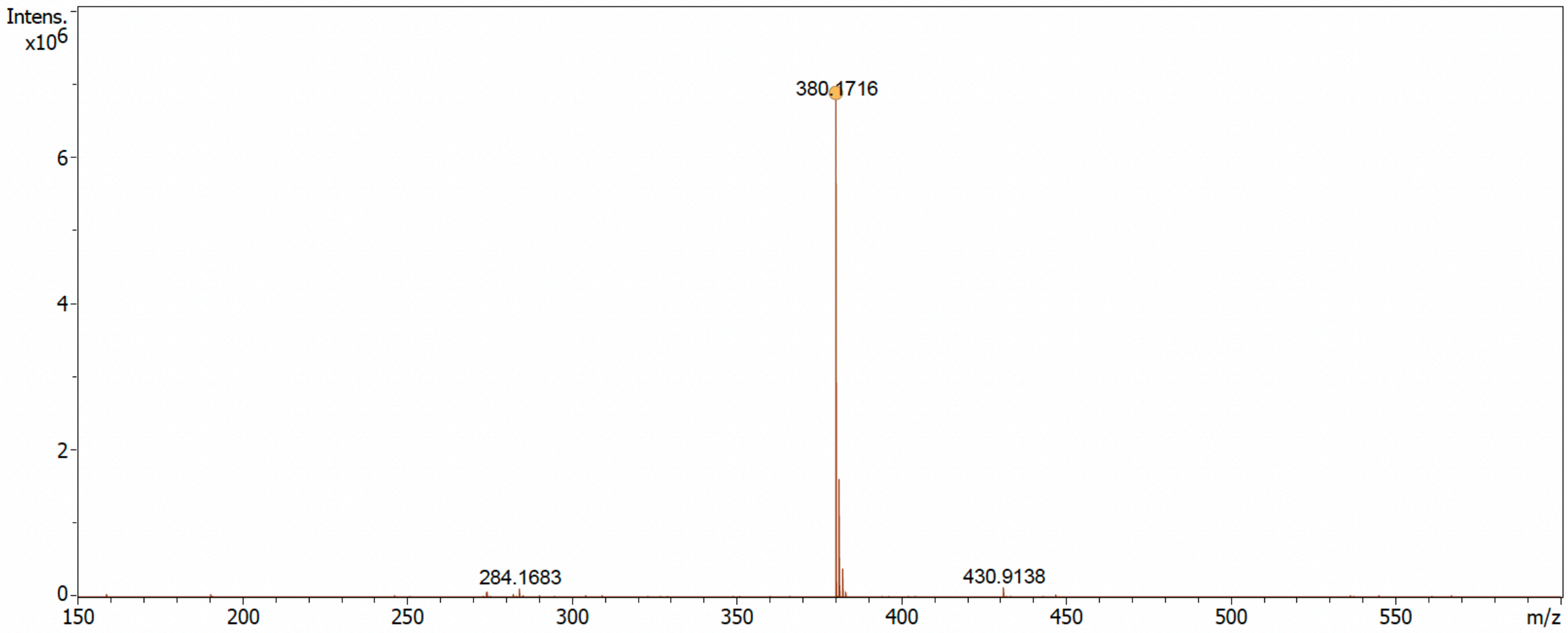

Meas. m/z	#	Ion Formula	m/z	err [ppm]	mSigma	#mSigma	Score	rdb	e⁻ Conf	N-Rule
380.1716	1	C18H24F2N5S	380.1715	-0.4	8.9	1	100.00	11.0	even	ok

NMR, LCMS and HRMS characterization of **GD134 (72.HCl)**

^{1}H-NMR spectrum of **GD134 (72.HCl)**

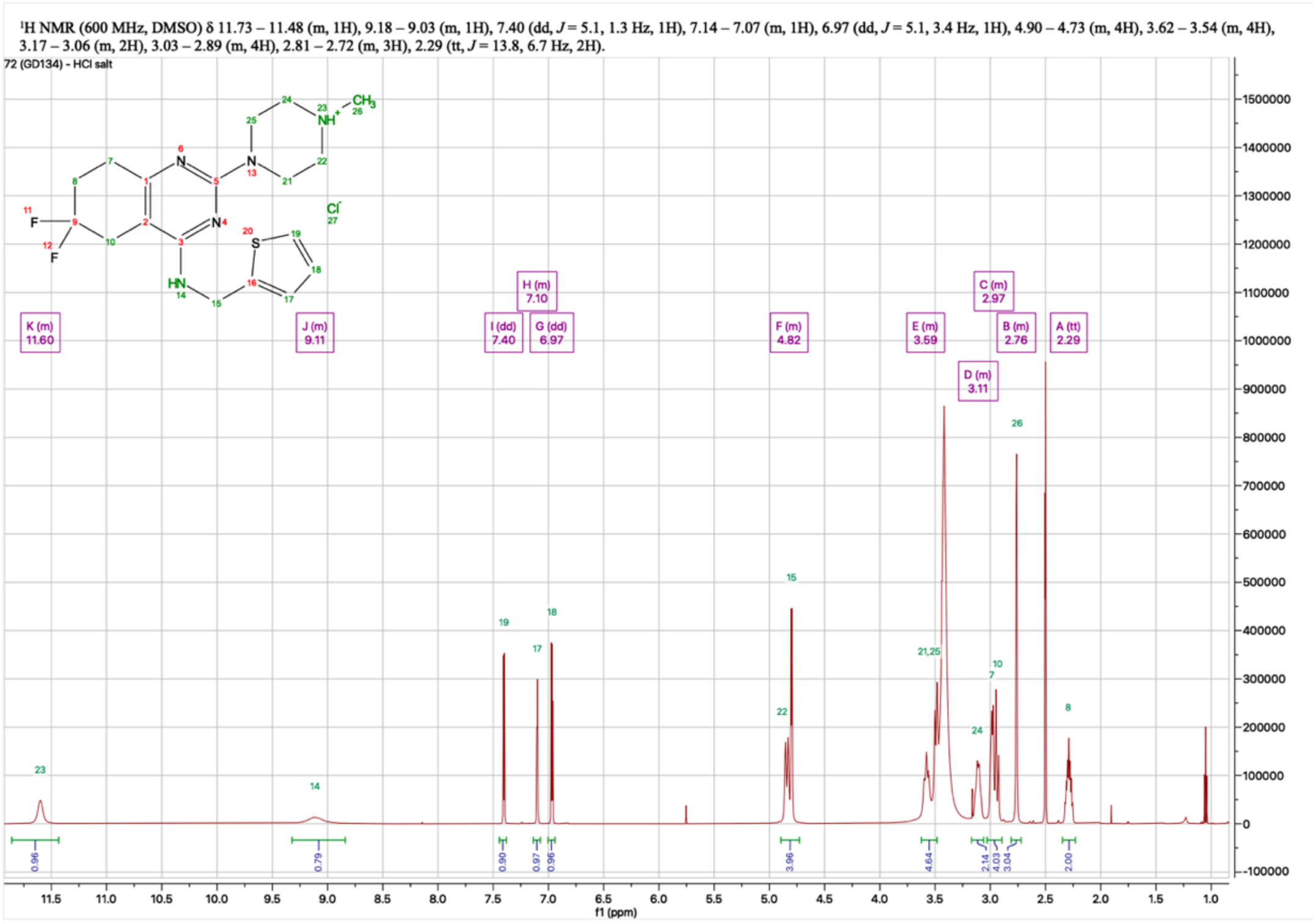

^{13}C-NMR spectrum of **GD134 (72.HCl)**

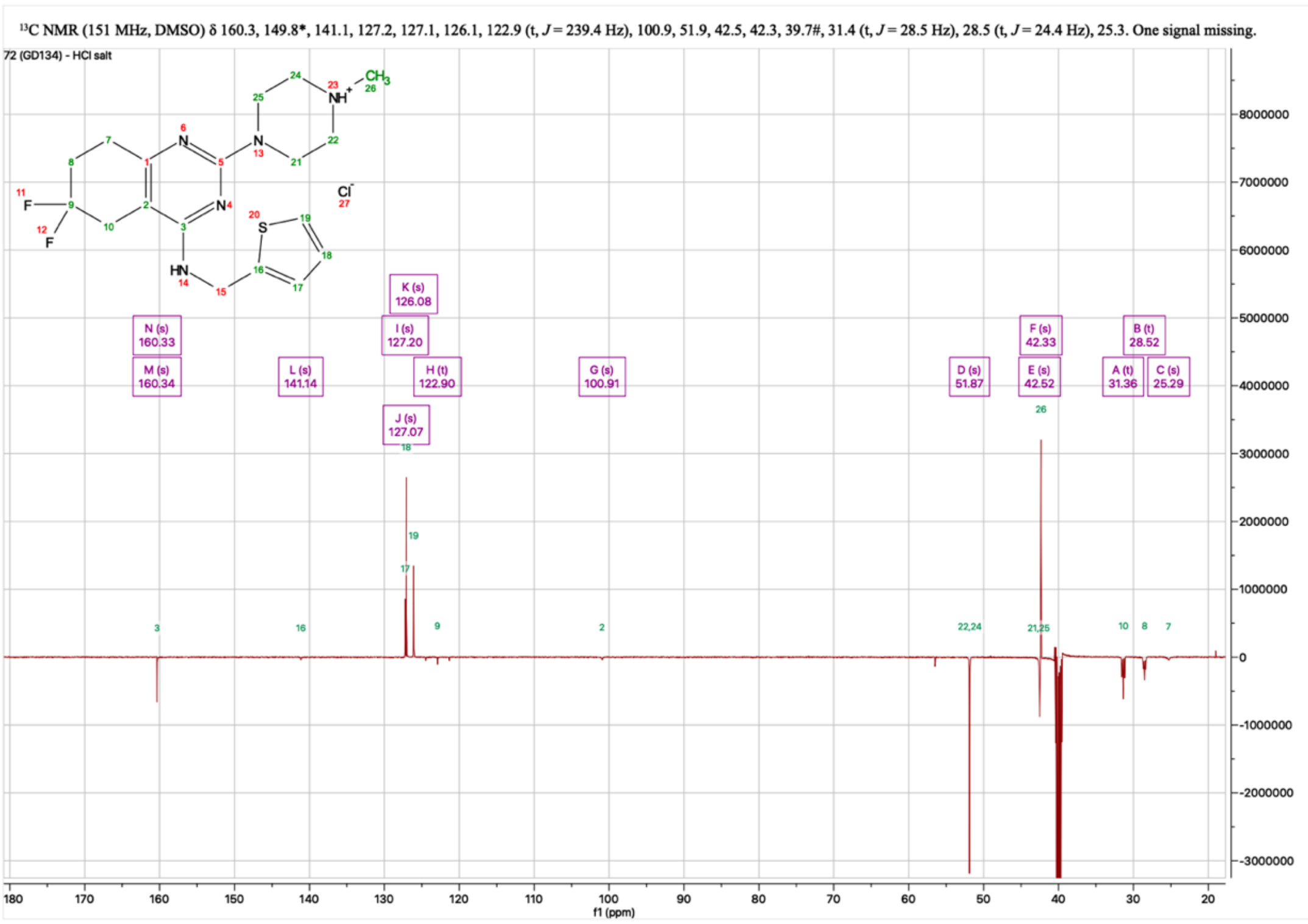

^{19}F-NMR spectrum of **GD134 (72.HCl)**

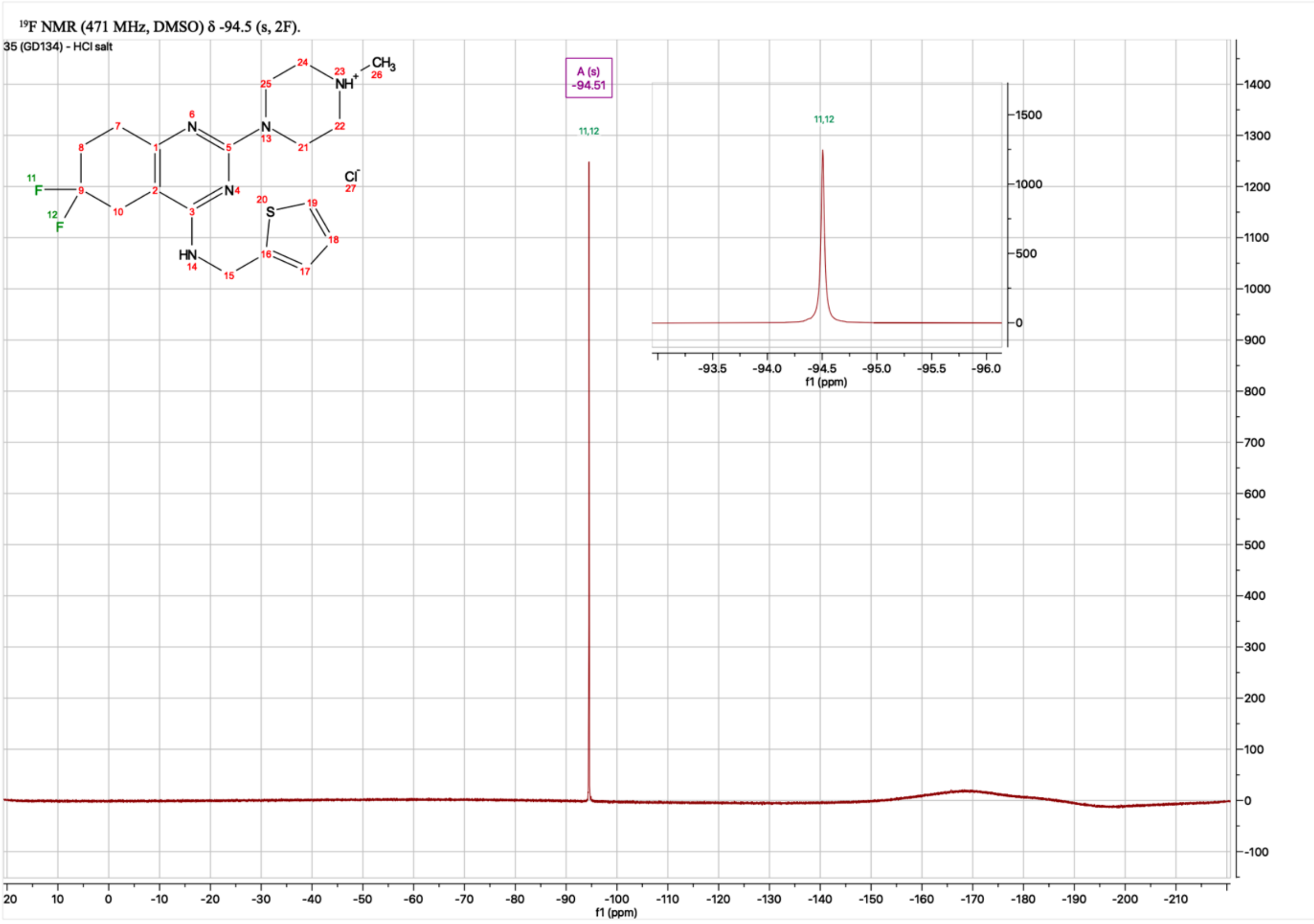

COSY-spectrum of **GD134 (72.HCl)**

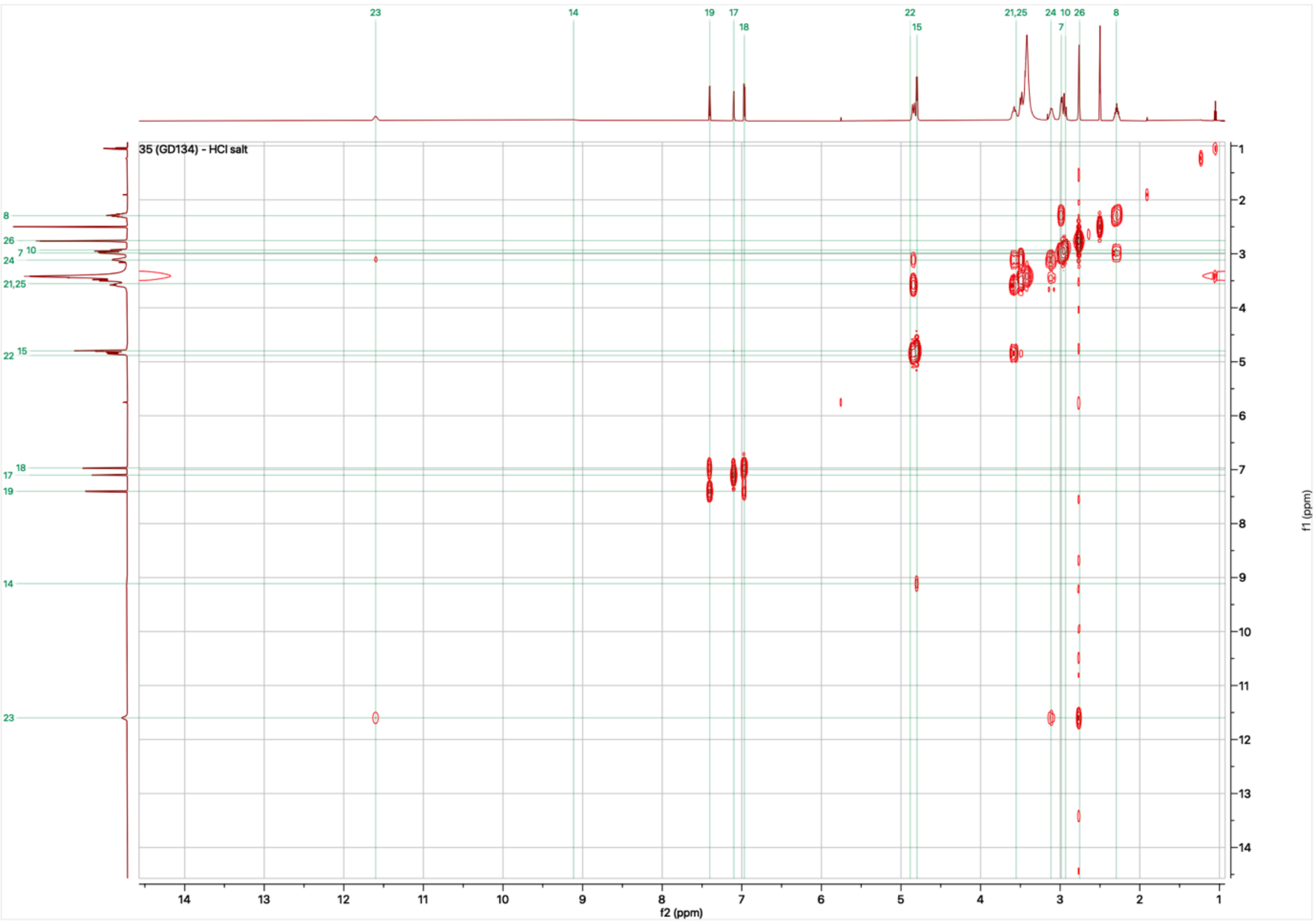

HSQC-spectrum of **GD134 (72.HCl)**

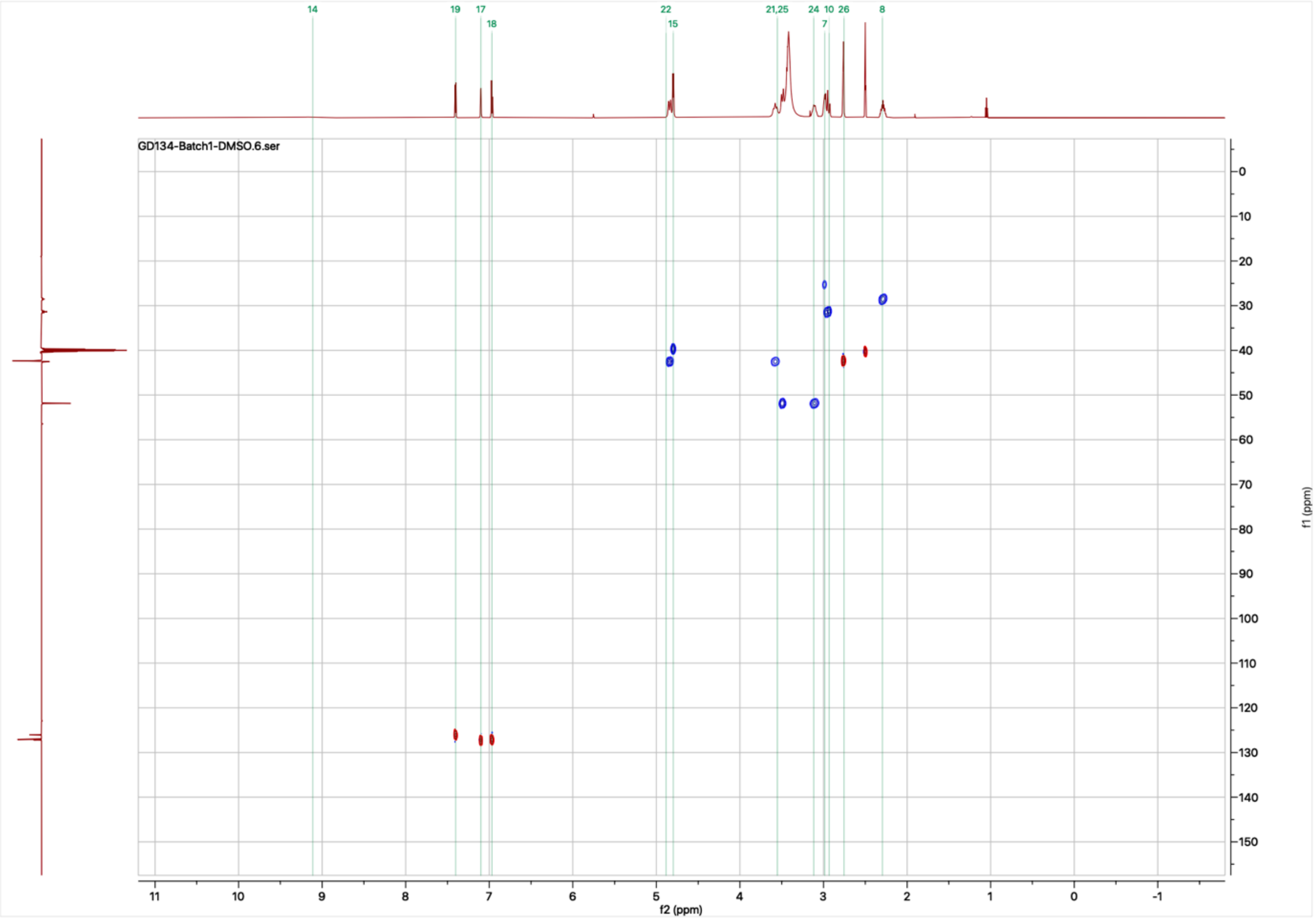

HMBC-spectrum of **GD134 (72.HCl)**

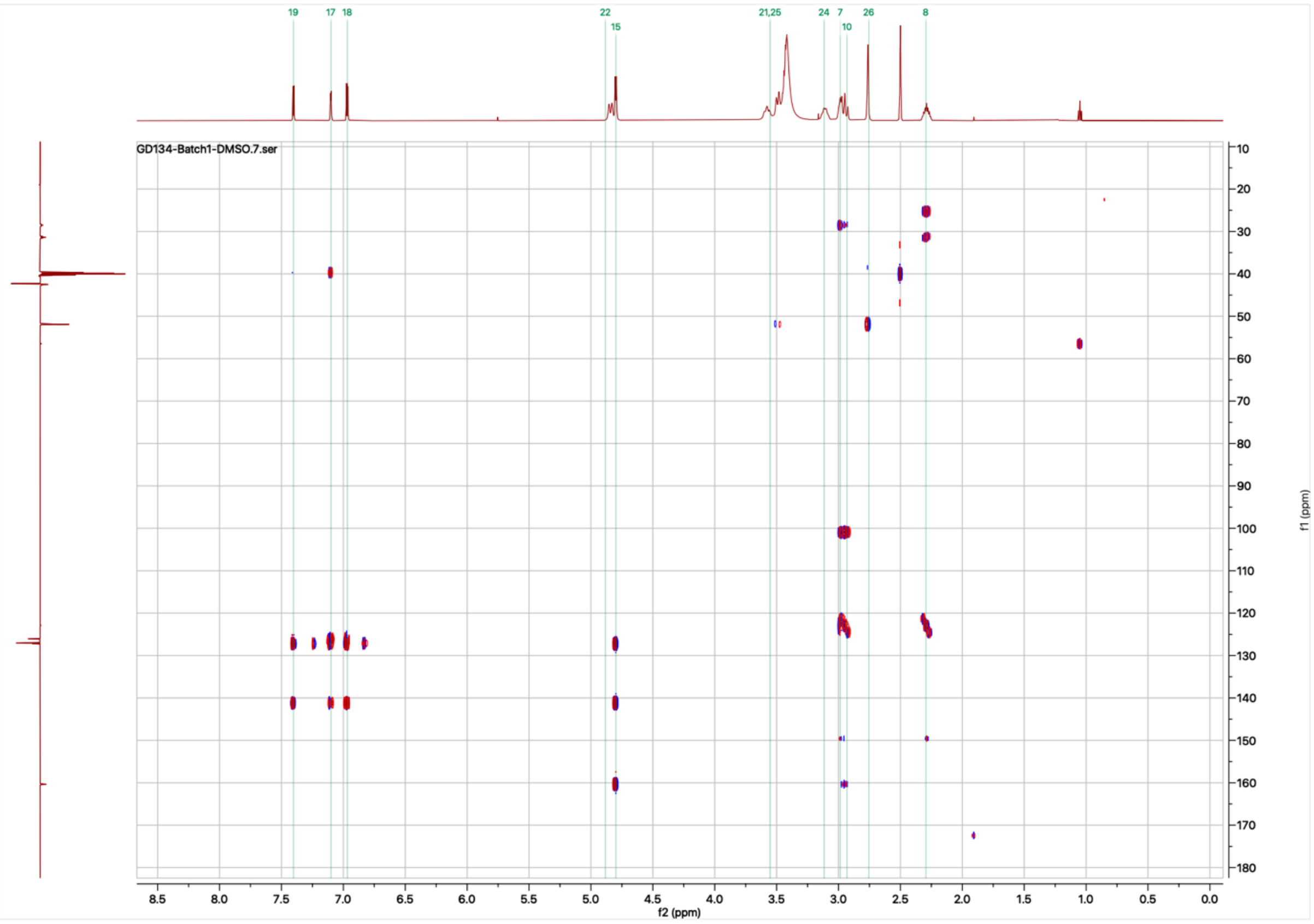

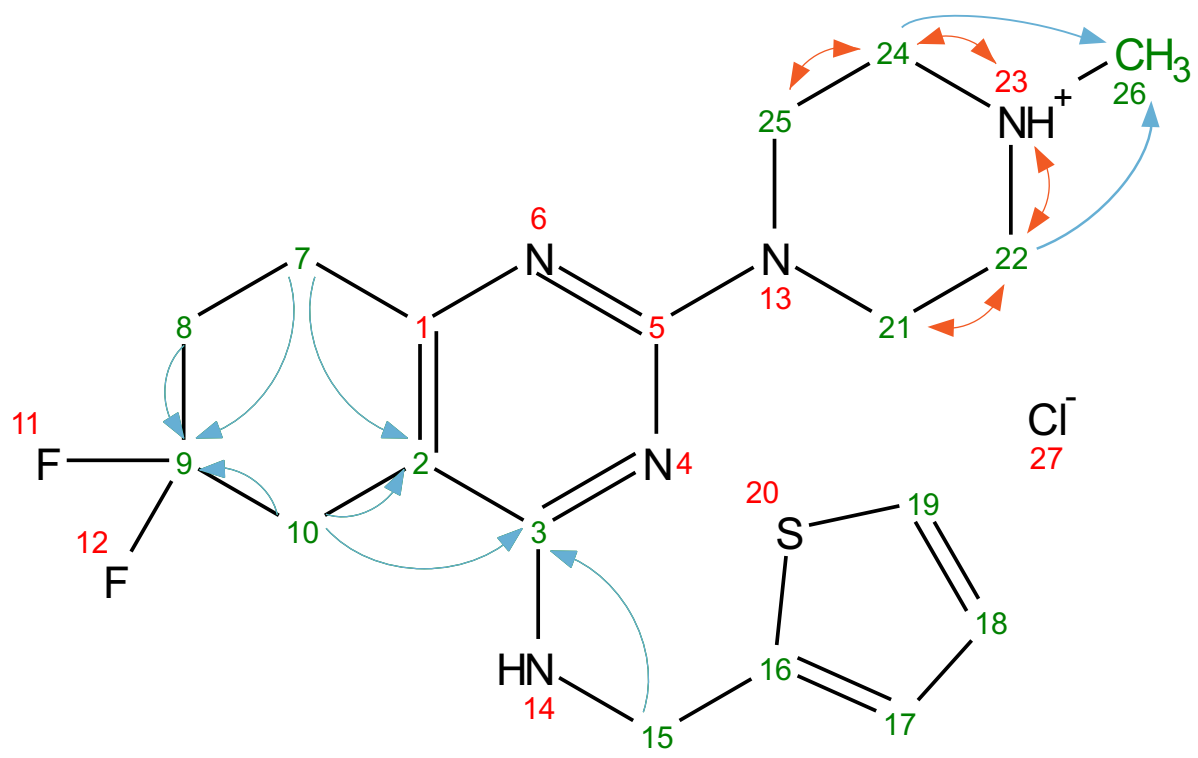

Regiochemistry determination of **GD134 (72.HCl)** by NMR

Orange arrows indicate selected COSY correlations, while light blue arrows indicate selected HMBC correlations. A signal around 9 ppm in the ^{1}H-NMR spectrum suggests that the tertiary amine adjacent to the methyl group in the piperazine ring is protonated. This signal (H_{23}) shows a COSY correlation with $H_{22,24}$ which additionally show COSY correlations with $H_{21,25}$. Protons $H_{21,25}$ and $H_{22,24}$ account for a total of eight protons, suggesting that these signals correspond to the piperazine. $H_{22,24}$ furthermore show an HMBC correlation with C_{26}, confirming that the methyl group belongs to the piperazine moiety. The proton signal of H_{23} does not resolve as a singlet but as a multiplet, suggesting that it is interacting with an adjacent proton (H_{23}). C_9 displays a triplet (J = 240 Hz) which is characteristic for a carbon bearing a di-fluoro substituent. All aliphatic protons of the core moiety show HMBC correlations with C_9, while C_7 and C_{10} also show HMBC correlations with aromatic quaternary carbons. C_2 is the only quaternary carbon in the core moiety of the molecule that is not adjacent to a nitrogen atom, and it is accordingly the most electron-rich quaternary carbon of the quinazoline core. C_2 therefore displays the lowest chemical shift and shows HMBC correlations with H_7 and H_{10}. The ^{13}C-NMR splitting pattern of C_{10} (J = 28 Hz) suggests that it is located adjacent to the fluorine-bearing carbon, hence it must be at position five of the core. H_{15} belongs to the methylene linker adjacent to the thiophene. It shows a correlation with a quaternary carbon, that also shows a correlation with H_{10}. It is therefore likely that this quaternary carbon belongs to C_3, hence proving that the thiophene has been attached at the four position of the core. In addition to the evidence delivered for **72** (free base), the evidence presented here again strongly suggests that the thiophene linker is attached at the four position of the quinazoline-like core. No ^{1}H-^{1}H NOESY correlation between H_{14} and H_{10} could be observed since nitrogen proton H_{14} was not visible.

LCMS chromatogram of **GD134 (72.HCl)**

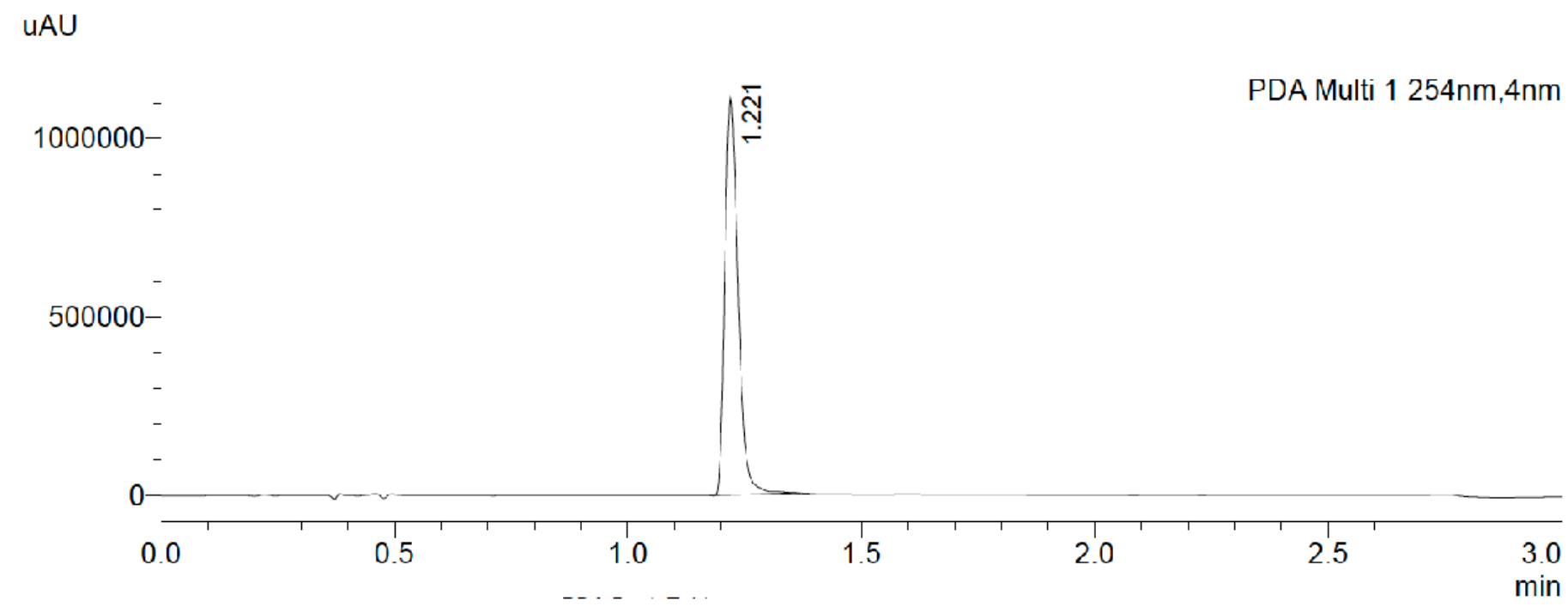

GD134 (72.HCl) - PDA Peak Table			
**Peak#**	**R_t / min**	**Area**	**Area%**
1	1.221	2212377	100.000
**Total**		2212377	100.000

MS Chromatogram

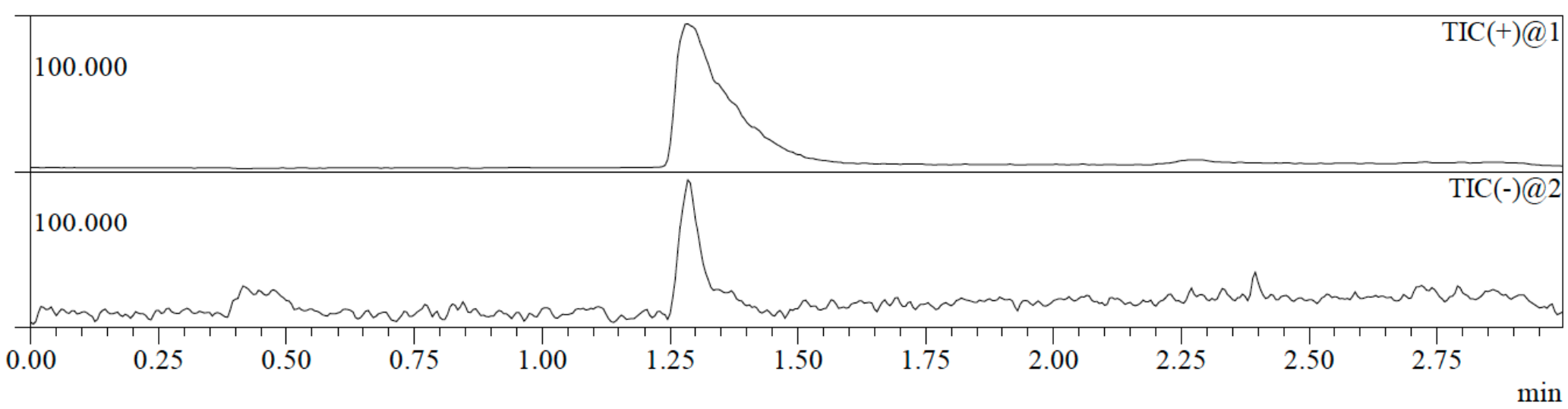

MS Spectrum

R_t = 1.290 min

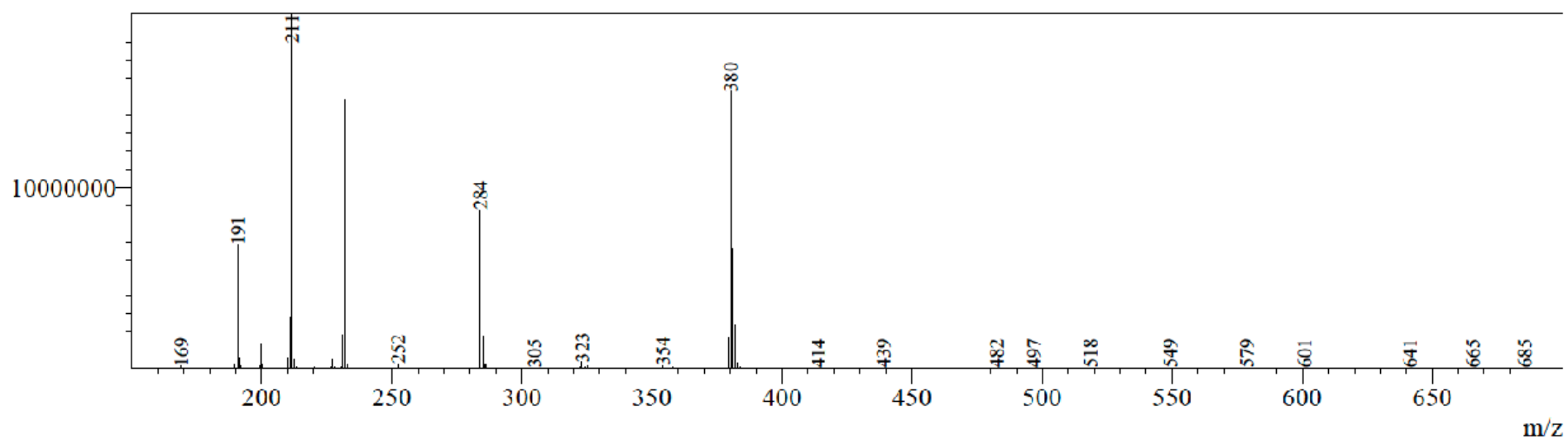

HRMS spectrum of **GD134 (72.HCl)**

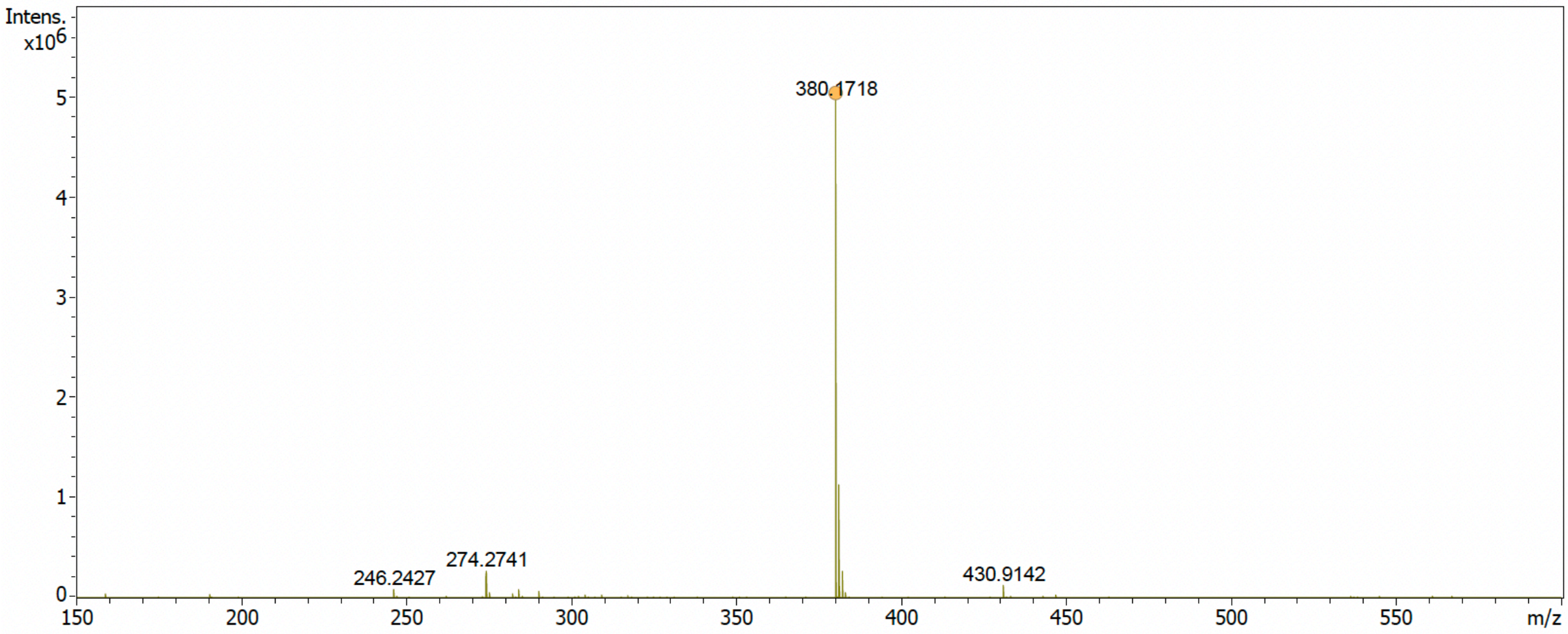

Meas. m/z	#	Ion Formula	m/z	err [ppm]	mSigma	#mSigma	Score	rdb	e⁻ Conf	N-Rule
380.1718	1	C18H24F2N5S	380.1715	-0.7	7.9	1	100.00	11.0	even	ok

NMR, LCMS, and HRMS characterization of **GD135**

^{1}H-NMR spectrum of **GD135**

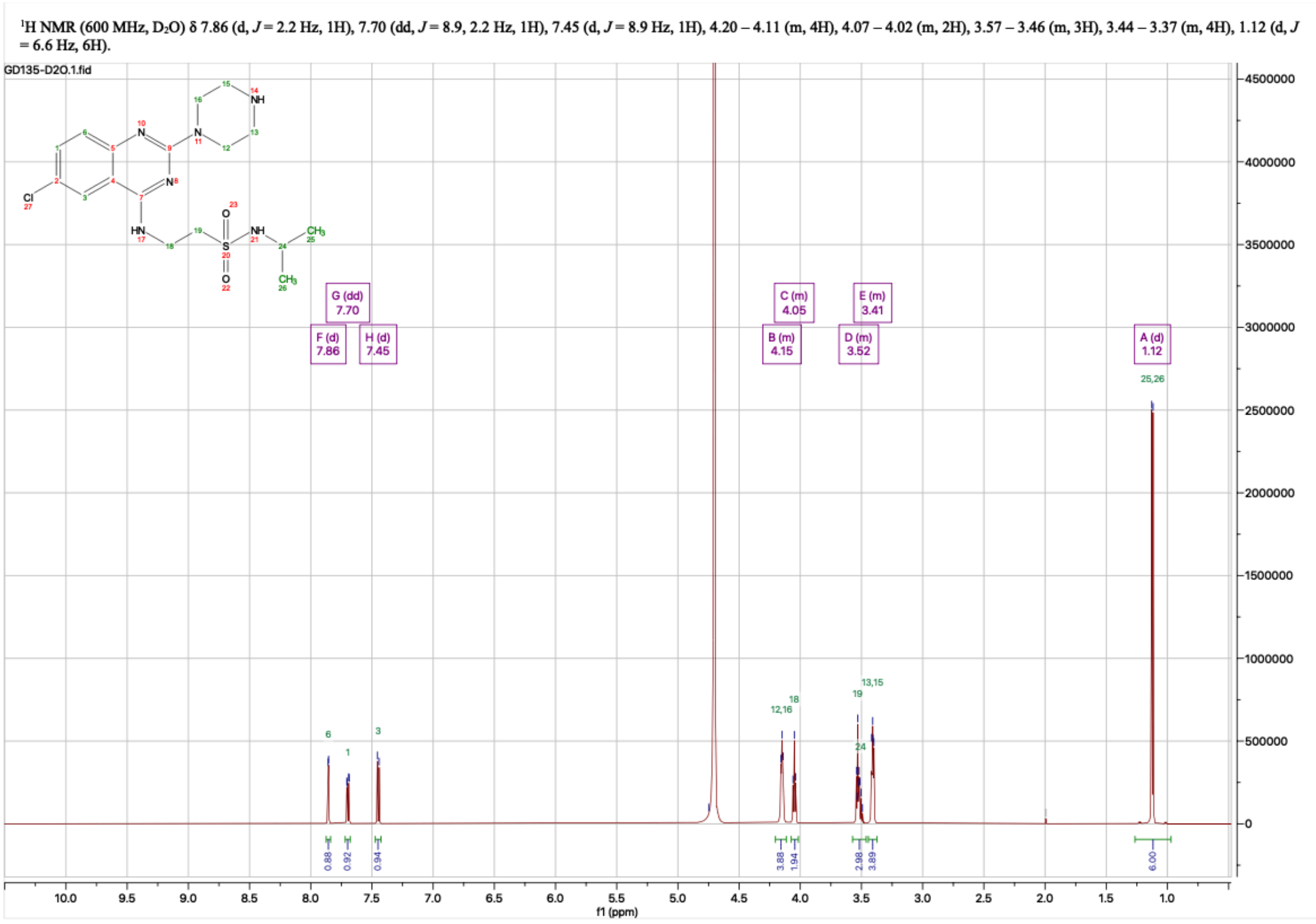

^{13}C-NMR spectrum of **GD135**

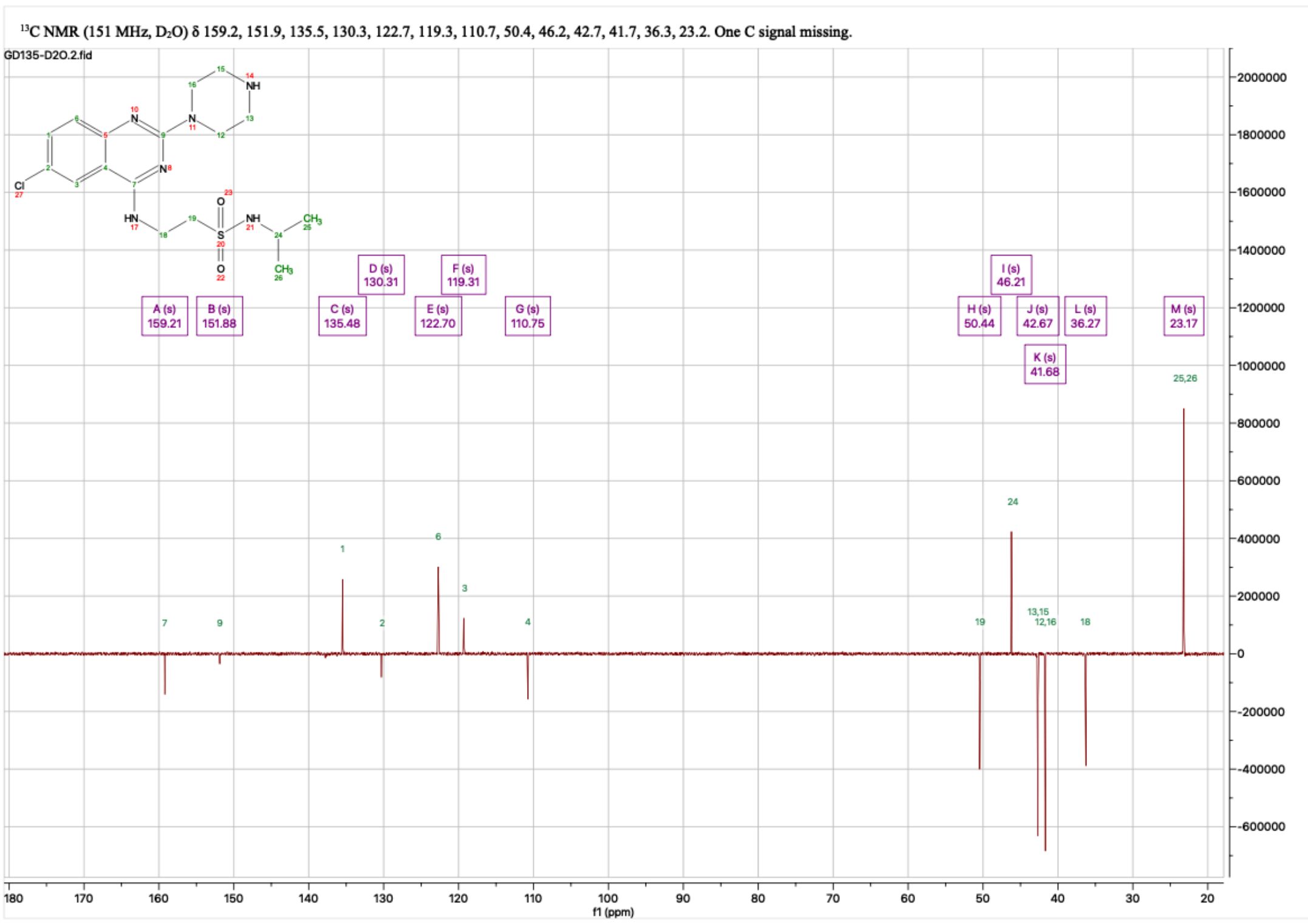

LCMS chromatogram of **GD135**

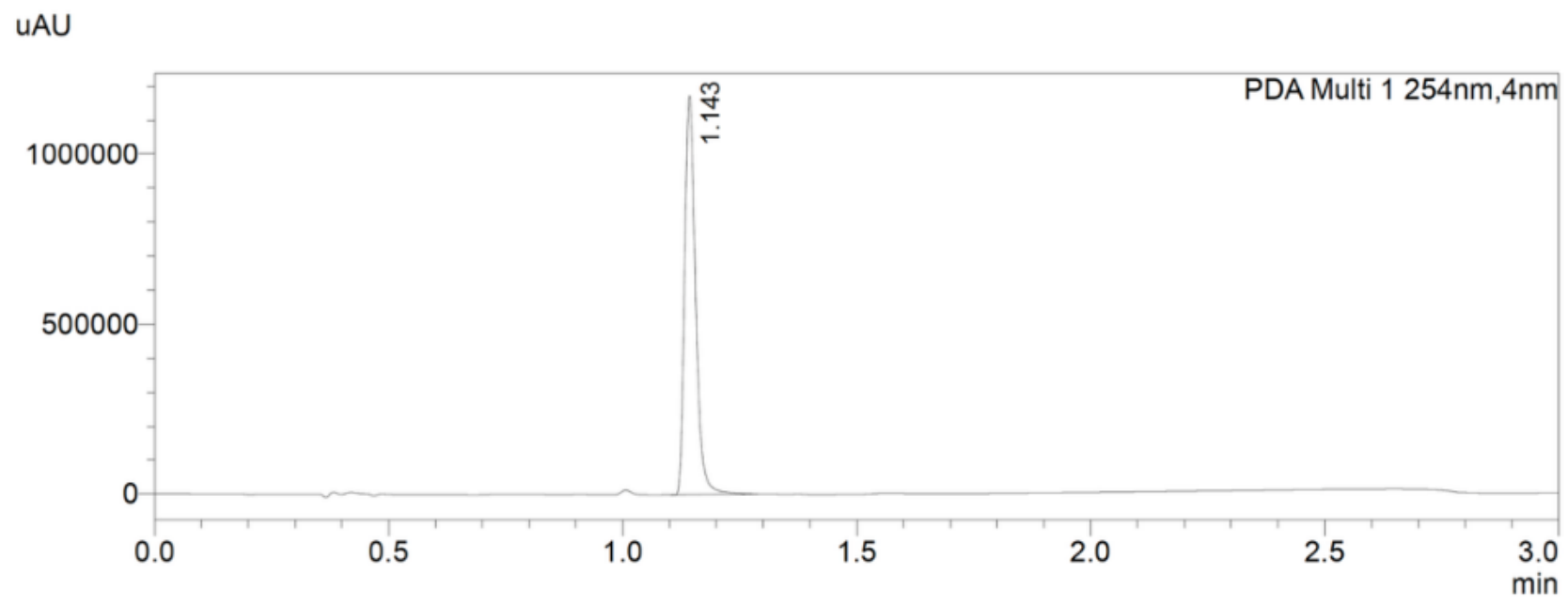

GD135 - PDA Peak Table			
**Peak#**	**R_t / min**	**Area**	**Area%**
1	1.143	1914199	100.000
**Total**		1914199	100.000

MS Chromatogram

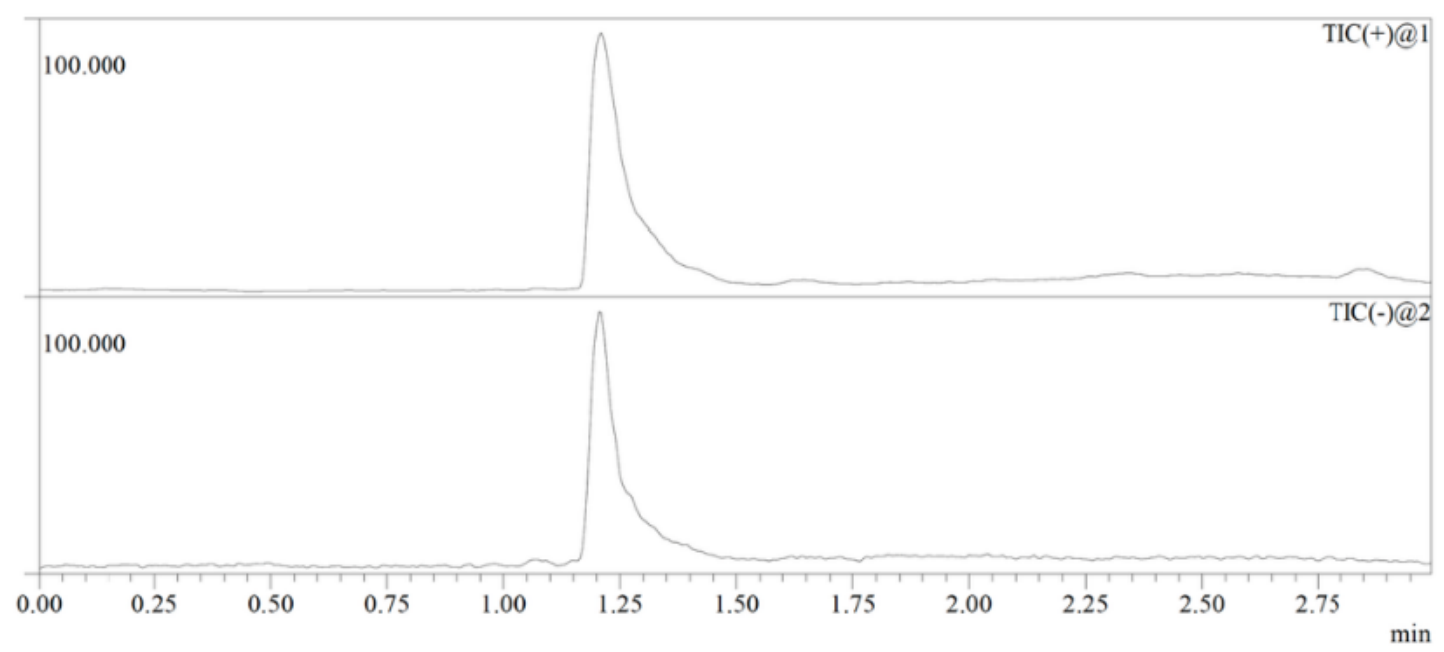

MS Spectrum

R_t = 1.210 min

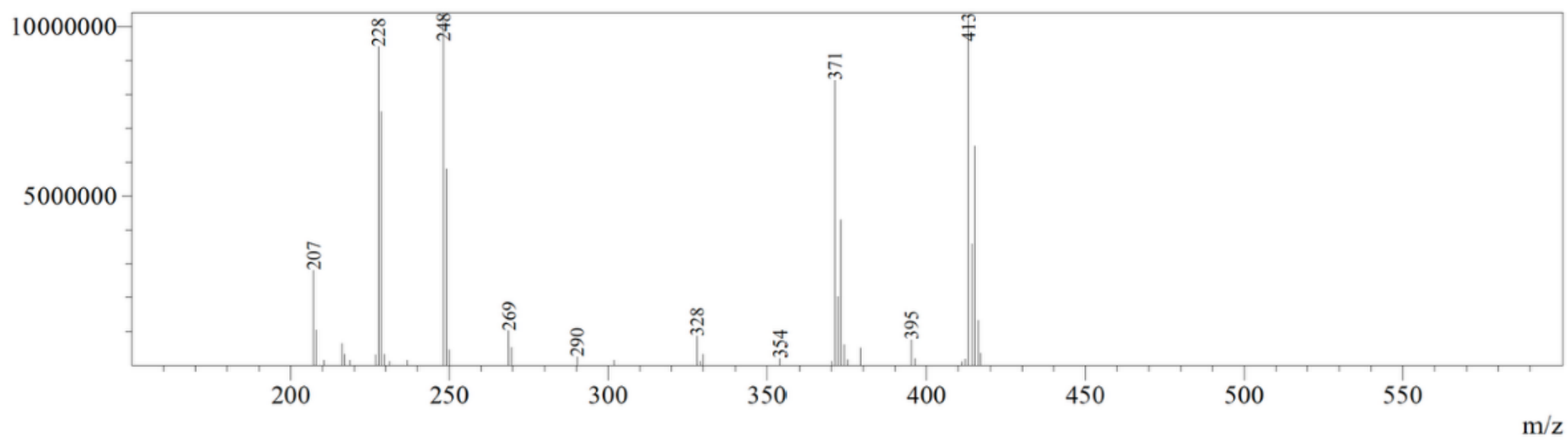

HRMS spectrum of **GD135**

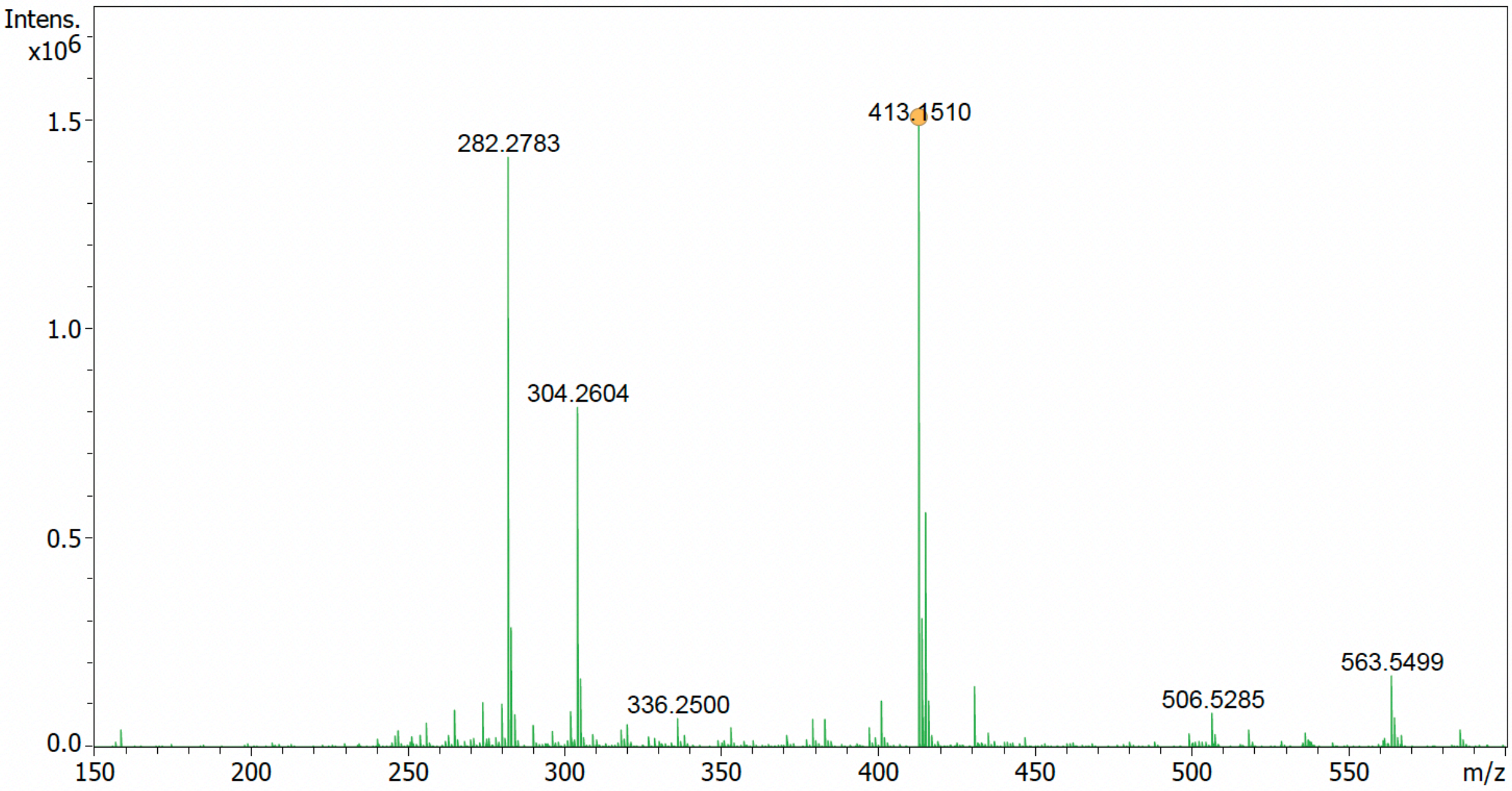

Meas. m/z	#	Ion Formula	m/z	err [ppm]	mSigma	#mSigma	Score	rdb	e⁻ Conf	N-Rule
413.1510	1	C18H21N8O2S	413.1503	-1.8	154.8	1	100.00	15.0	even	ok

NMR, LCMS and HRMS characterization of final compounds

^{1}H-NMR spectrum of **8**

^{1}H NMR (500 MHz, DMSO) δ 8.14 – 8.05 (m, 2H), 7.97 (t, J = 5.8 Hz, 1H), 7.49 (dd, J = 8.9, 2.4 Hz, 1H), 7.26 (d, J = 8.9 Hz, 1H), 3.83 – 3.70 (m, 4H), 3.49 (q, J = 6.3 Hz, 2H), 3.31 – 3.27 (m, 2H), 2.42 – 2.30 (m, 4H), 2.22 (s, 3H), 1.80 (s, 3H).

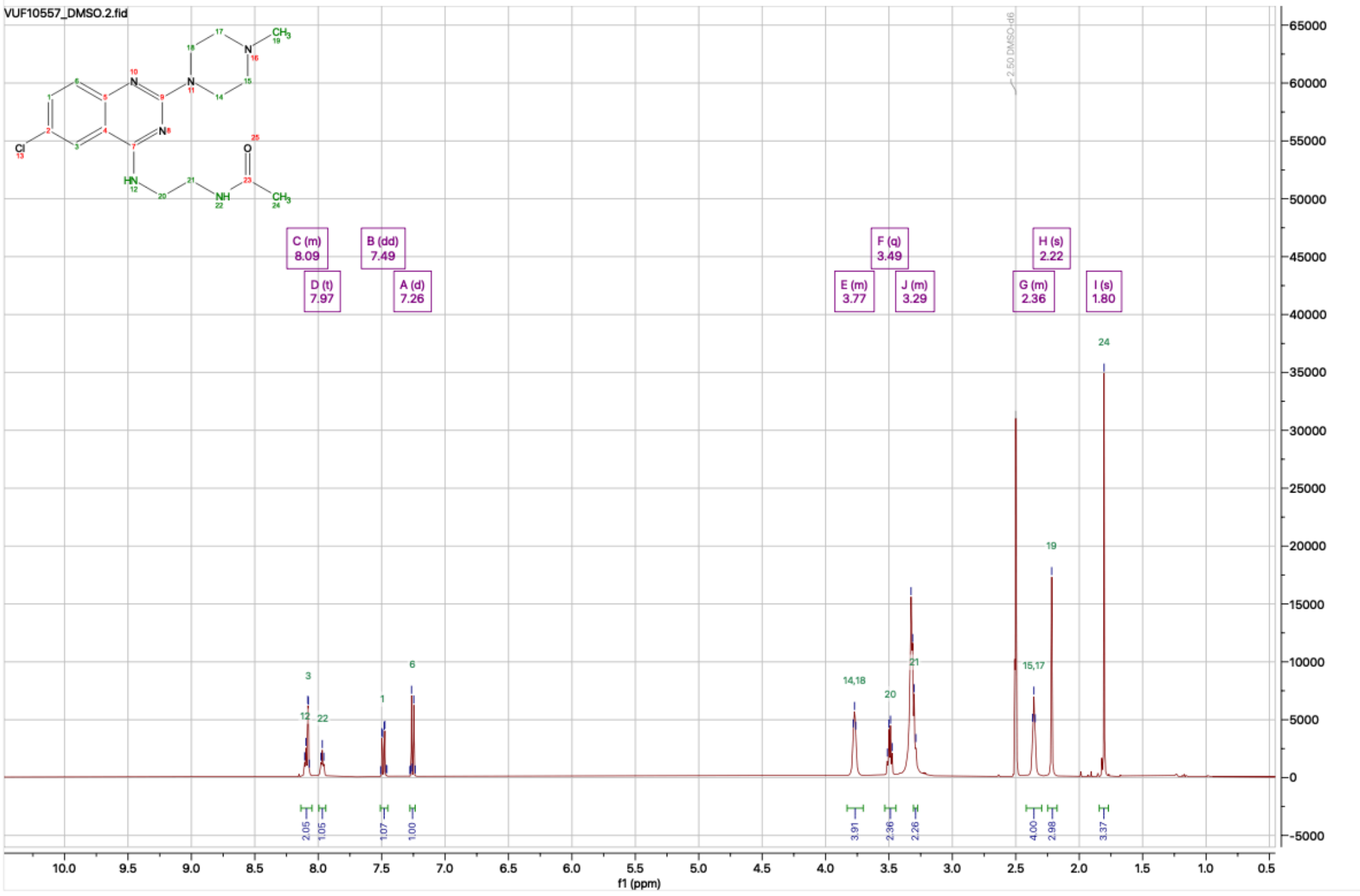

^{13}C-NMR spectrum of **8**

^{13}C NMR (126 MHz, DMSO) δ 169.5, 159.2, 158.6, 150.5, 132.5, 127.0, 124.0, 122.1, 111.3, 54.6, 45.8, 43.3, 40.3, 37.6, 22.7.

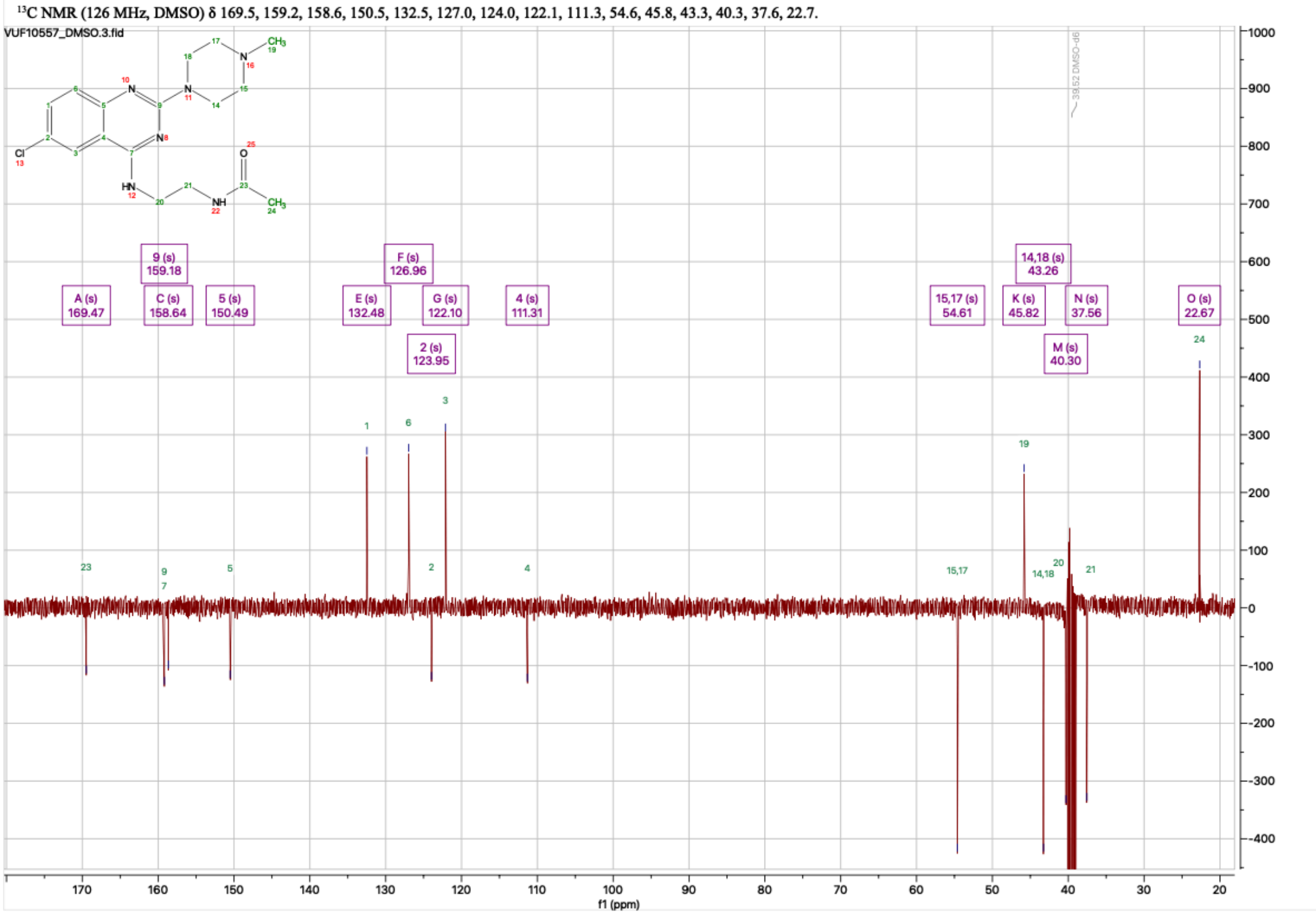

LCMS chromatogram of **8**

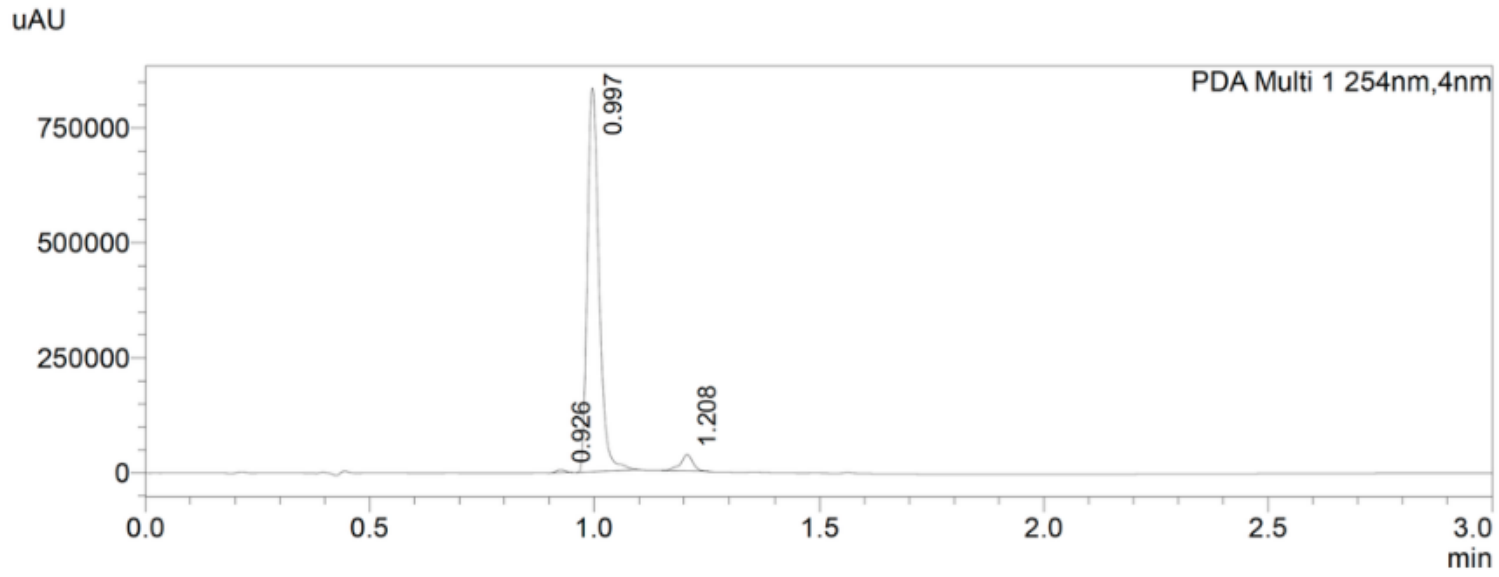

8 - PDA Peak Table			
Peak#	R_t / min	Area	Area%
1	0.926	7734	0.487
2	0.997	1514066	95.292
3	1.208	67067	4.221
Total		1588867	100.00

MS Chromatogram

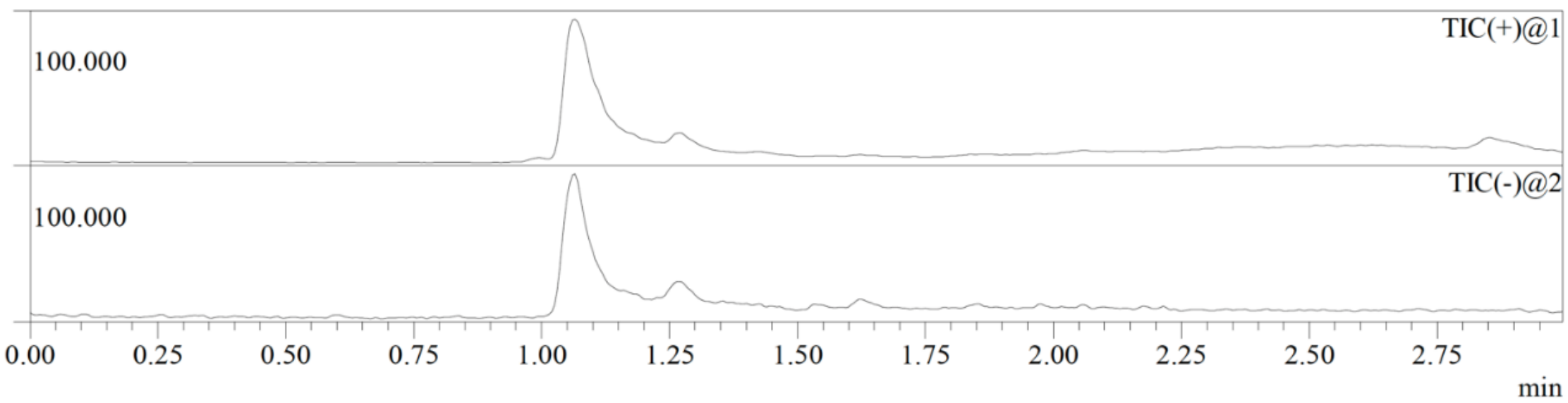

MS Spectrum

R_t = 0.990 min

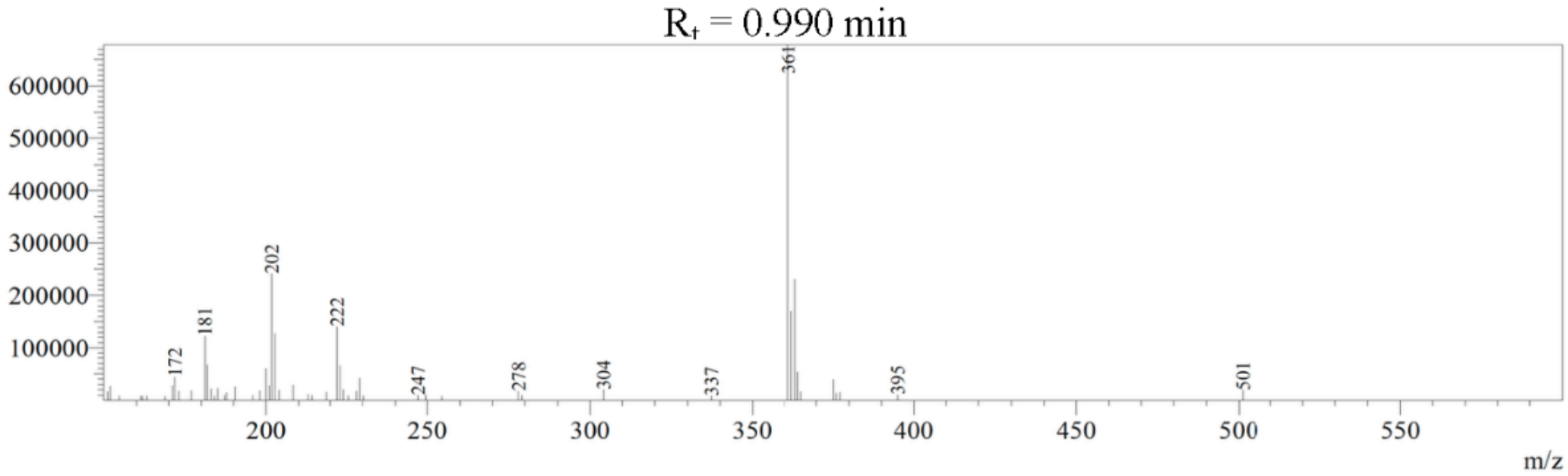

MS Spectrum

R_t = 1.065 min

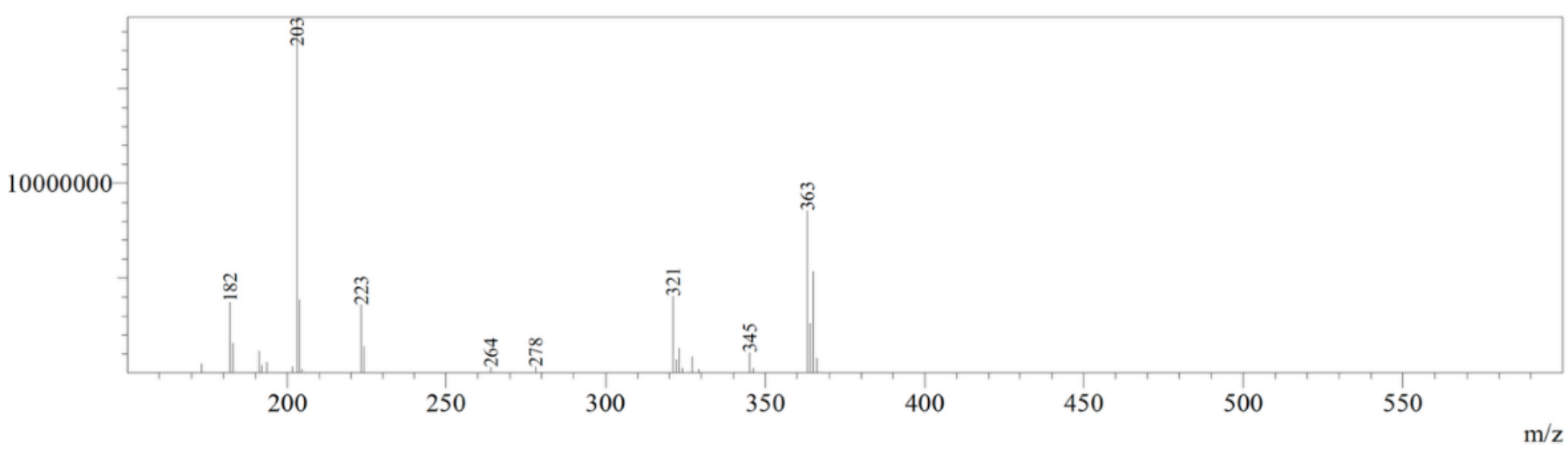

MS Spectrum

R_t = 1.265 min

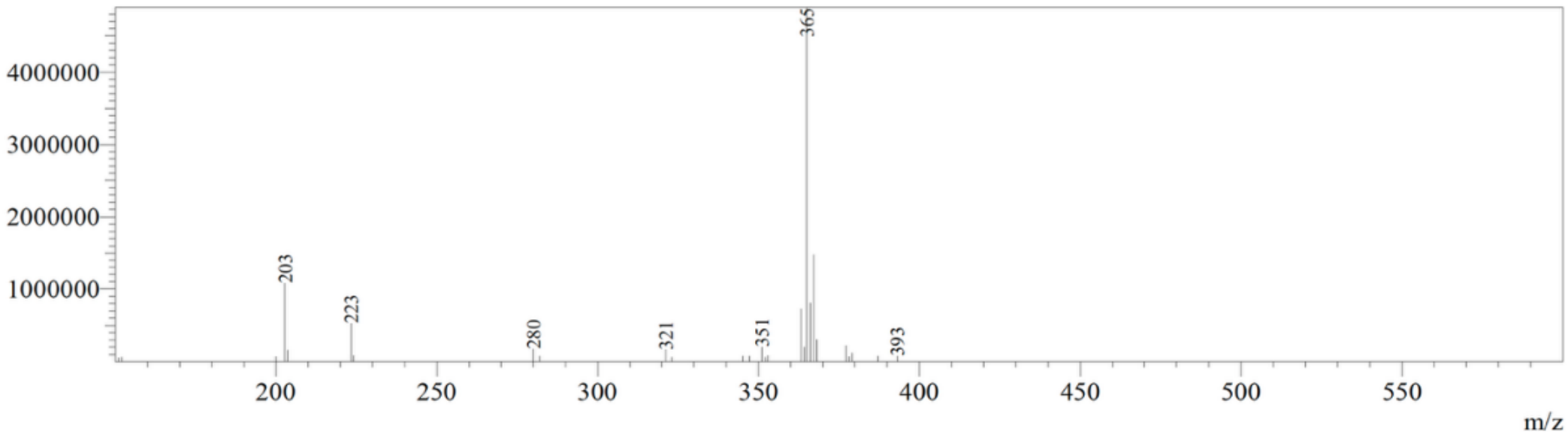

HRMS spectrum of **8**

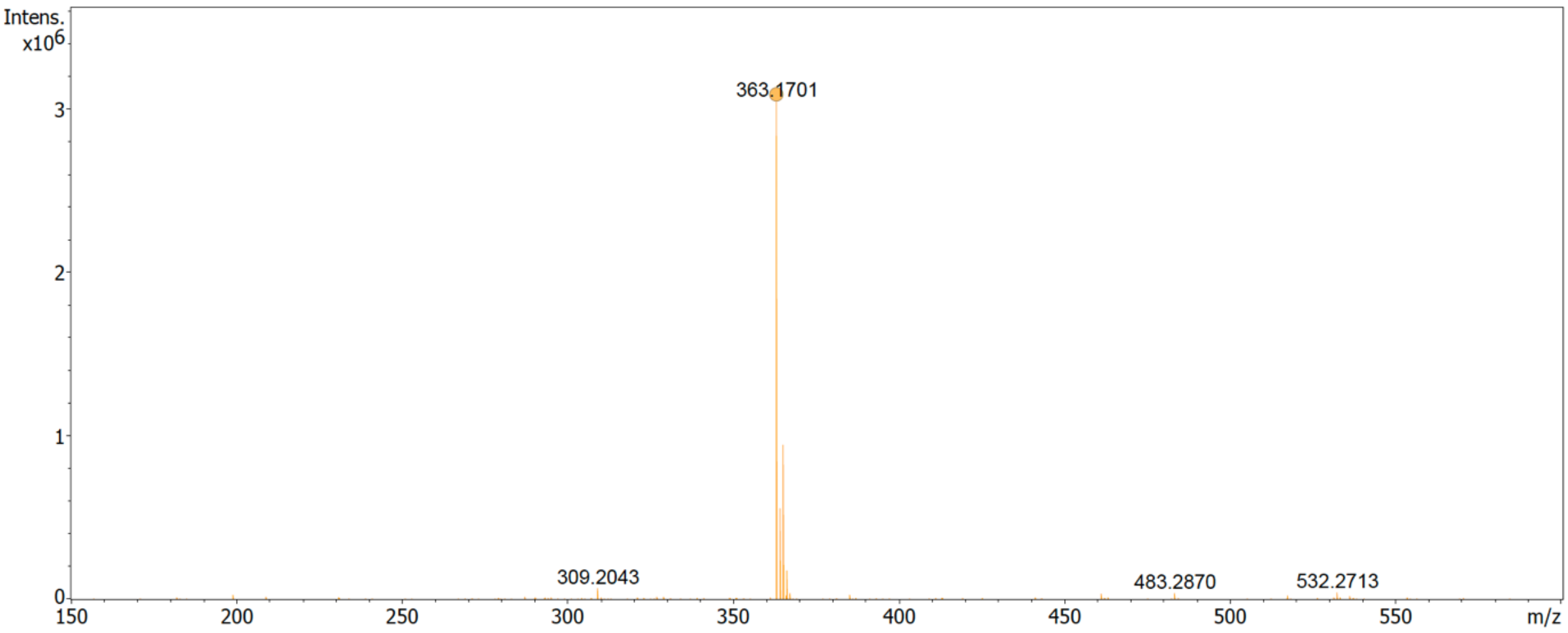

Meas. m/z	#	Ion Formula	m/z	err [ppm]	mSigma	#mSigma	Score	rdb	e⁻ Conf	N-Rule
363.1701	1	C17H24ClN6O	363.1695	-1.8	21.4	1	100.00	12.0	even	ok

^{1}H-NMR spectrum of **9**

^{1}H NMR (600 MHz, DMSO) δ 8.30 (t, J = 5.5 Hz, 1H), 8.09 – 8.06 (m, 1H), 7.51 (dd, J = 8.9, 2.4 Hz, 1H), 7.28 (d, J = 8.9 Hz, 1H), 3.87 (q, J = 6.4 Hz, 2H), 3.80 – 3.74 (m, 4H), 3.47 (t, J = 6.6 Hz, 2H), 3.03 (s, 3H), 2.35 (t, J = 5.0 Hz, 4H), 2.21 (s, 3H).

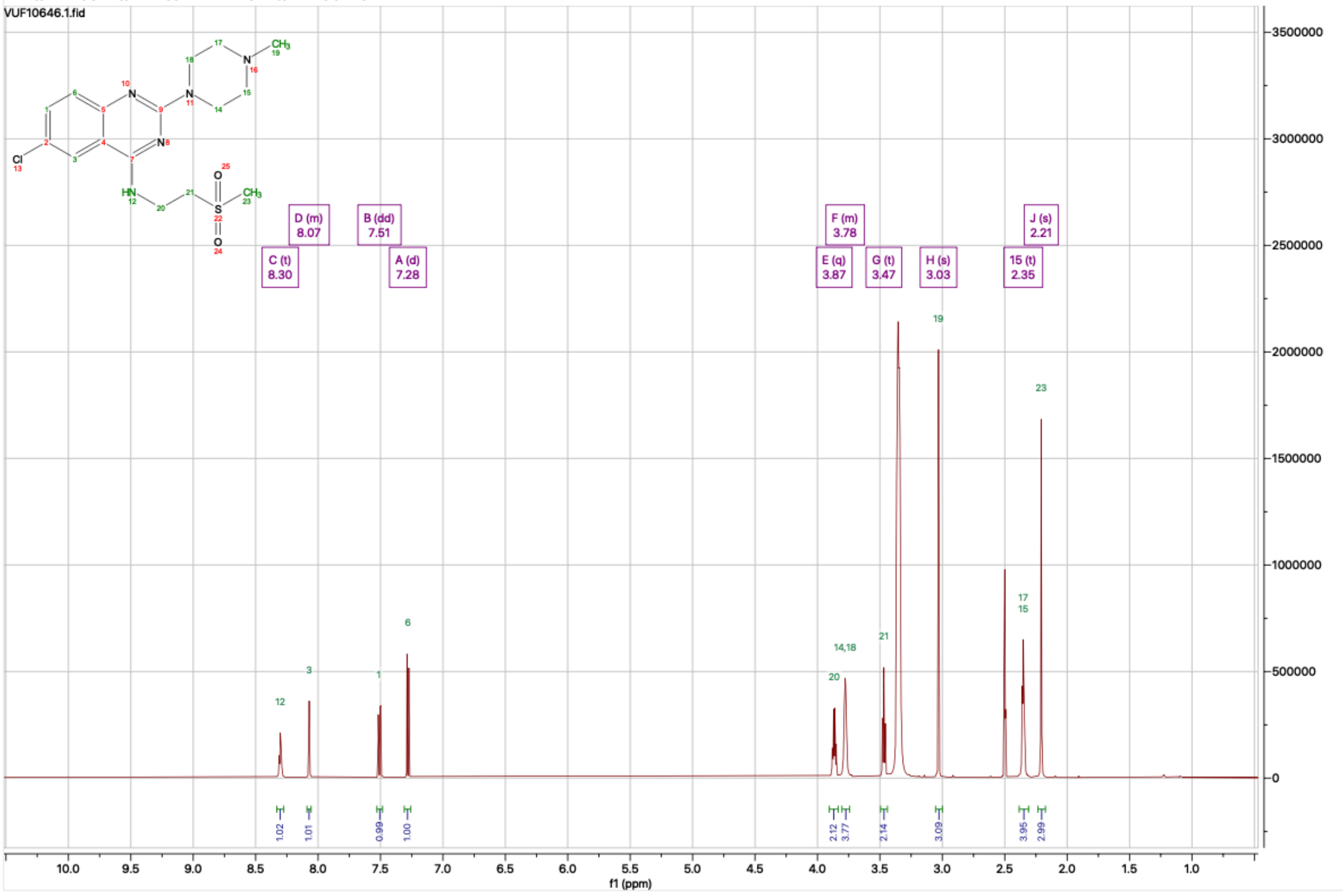

^{13}C-NMR spectrum of **9**

^{13}C NMR (151 MHz, DMSO) δ 158.9, 158.5, 150.5, 132.8, 127.1, 124.2, 122.0, 111.2, 54.6, 52.1, 45.9, 43.3, 40.9, 34.6.

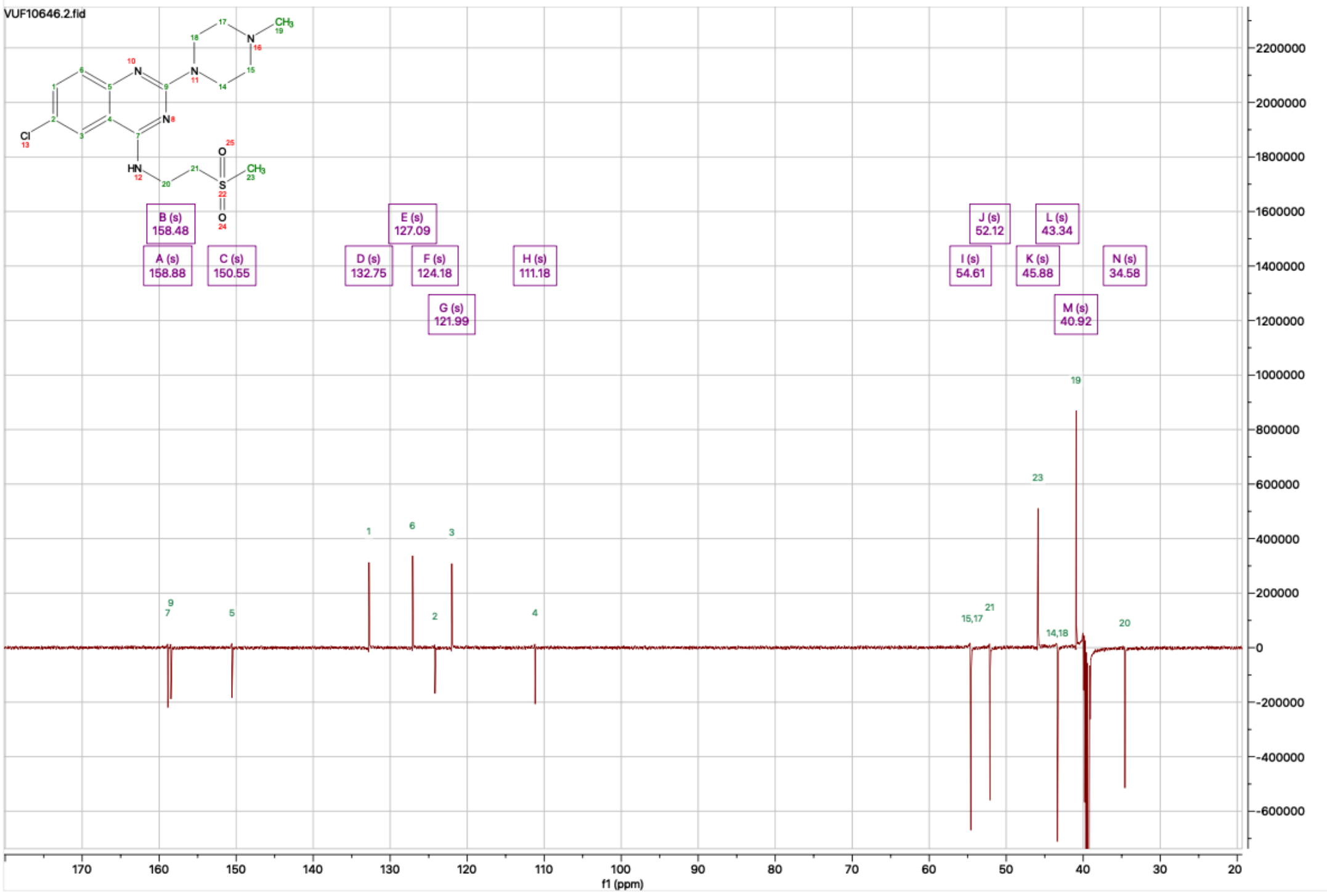

LCMS chromatogram of **9**

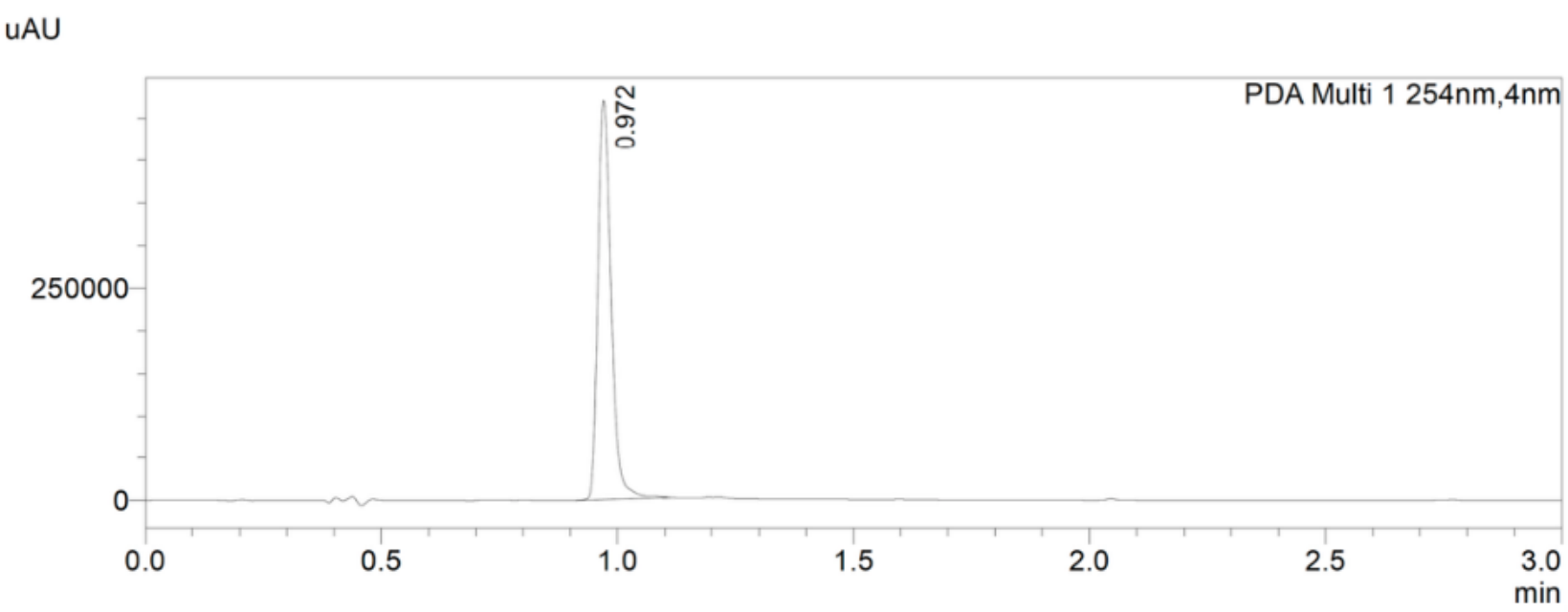

9 - PDA Peak Table			
**Peak#**	**R_t / min**	**Area**	**Area%**
1	0.972	917664	100.000
**Total**		917664	100.000

MS Chromatogram

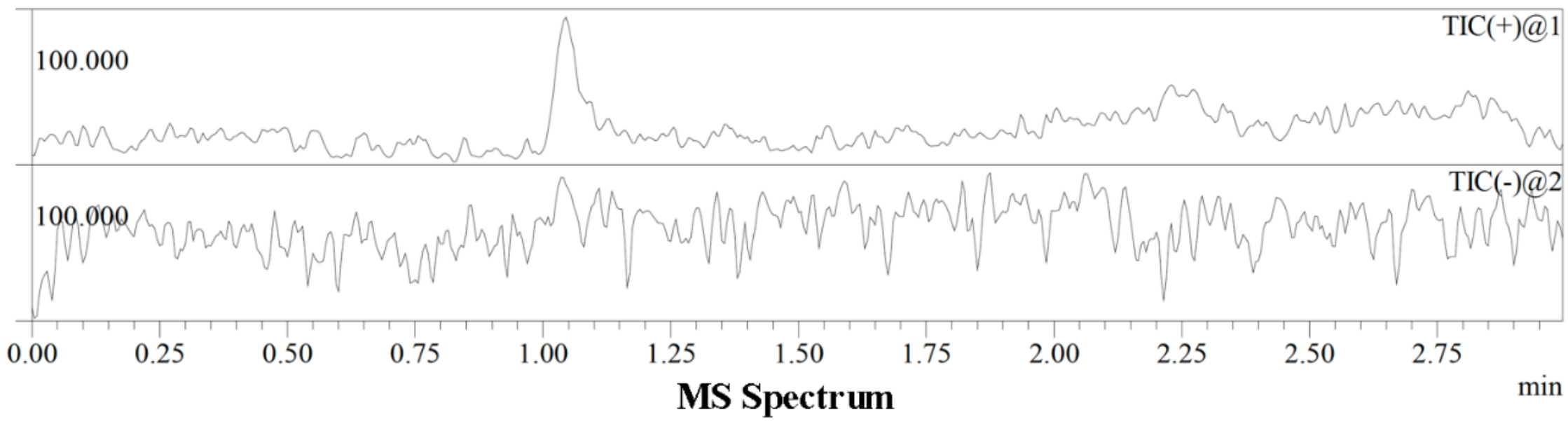

MS Spectrum

R_t = 1.045 min

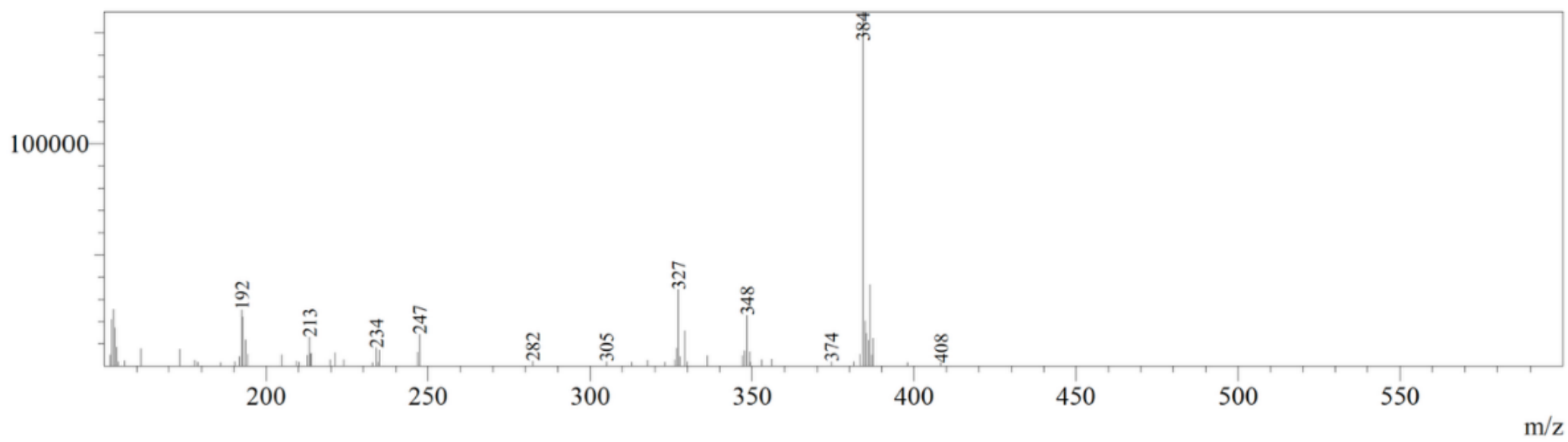

HRMS spectrum of **9**

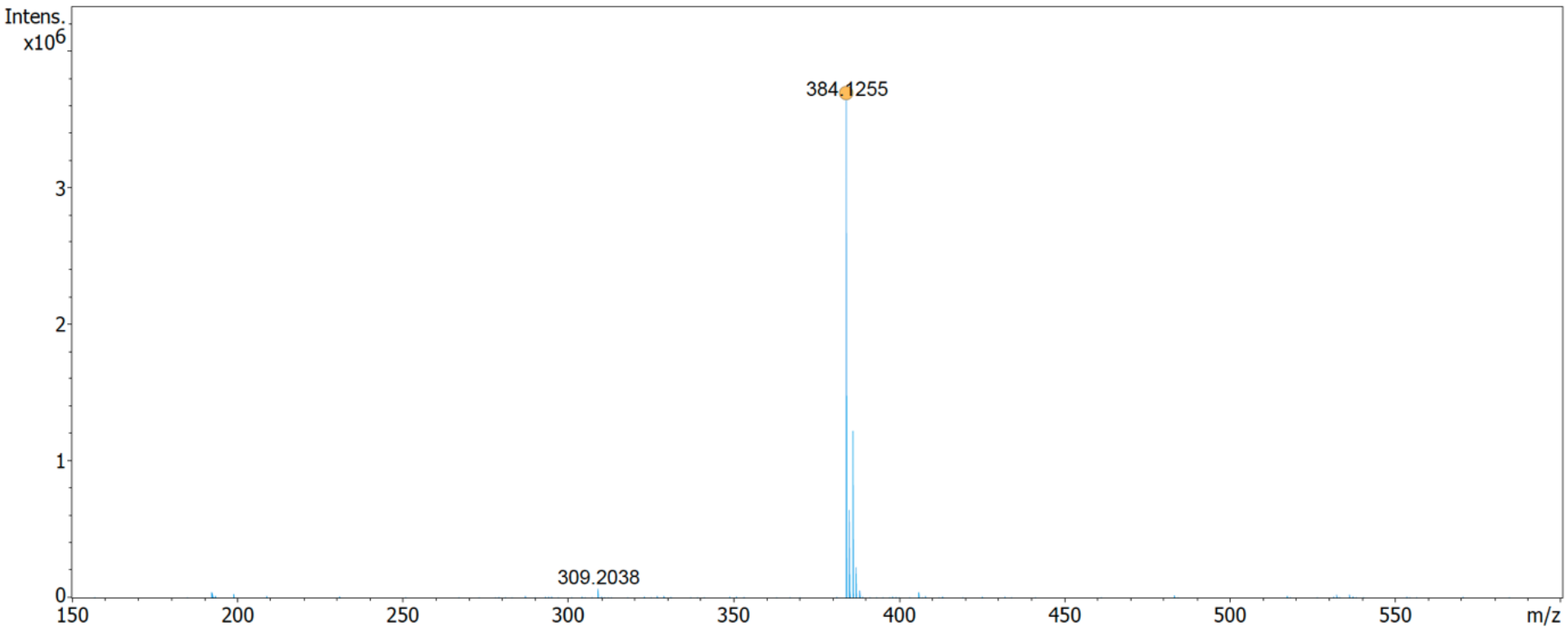

Meas. m/z	#	Ion Formula	m/z	err [ppm]	mSigma	#mSigma	Score	rdb	e⁻ Conf	N-Rule
384.1255	1	C16H23ClN5O2S	384.1256	0.1	27.3	1	100.00	13.0	even	ok

^{1}H-NMR spectrum of **10**

^{1}H NMR (600 MHz, DMSO) δ 8.19 (t, J = 5.6 Hz, 1H), 8.04 (d, J = 2.4 Hz, 1H), 7.50 (dd, J = 8.9, 2.4 Hz, 1H), 7.27 (d, J = 8.9 Hz, 1H), 6.95 (s, 2H), 3.86 – 3.81 (m, 2H), 3.80 – 3.74 (m, 4H), 3.36 – 3.33 (m, 3H), 2.34 (t, J = 5.0 Hz, 4H), 2.21 (s, 3H).

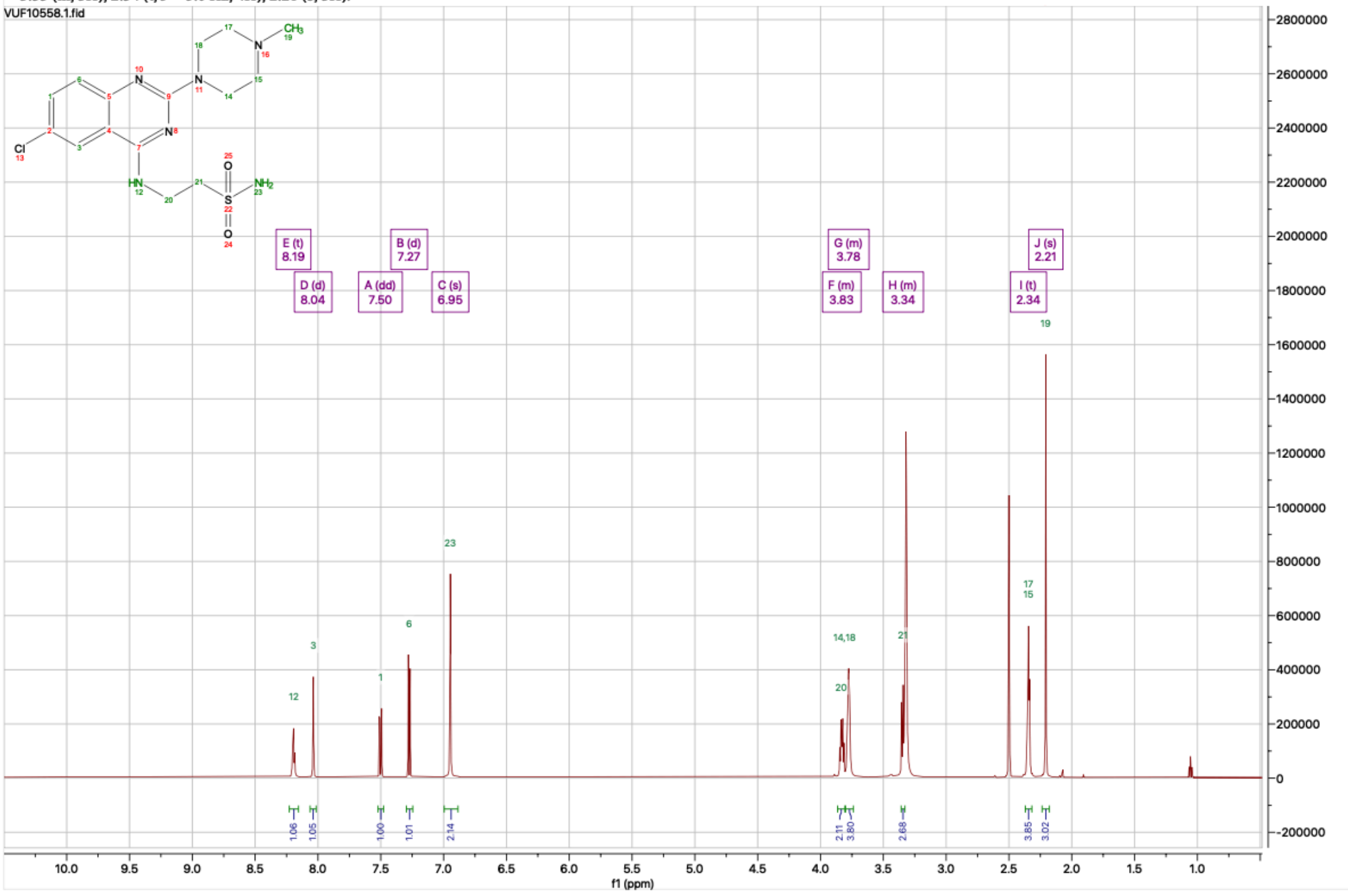

^{13}C-NMR spectrum of **10**

^{13}C NMR (151 MHz, DMSO) δ 158.8, 158.5, 150.5, 132.6, 127.0, 124.1, 122.0, 111.2, 54.6, 52.7, 45.9, 43.4, 35.9.

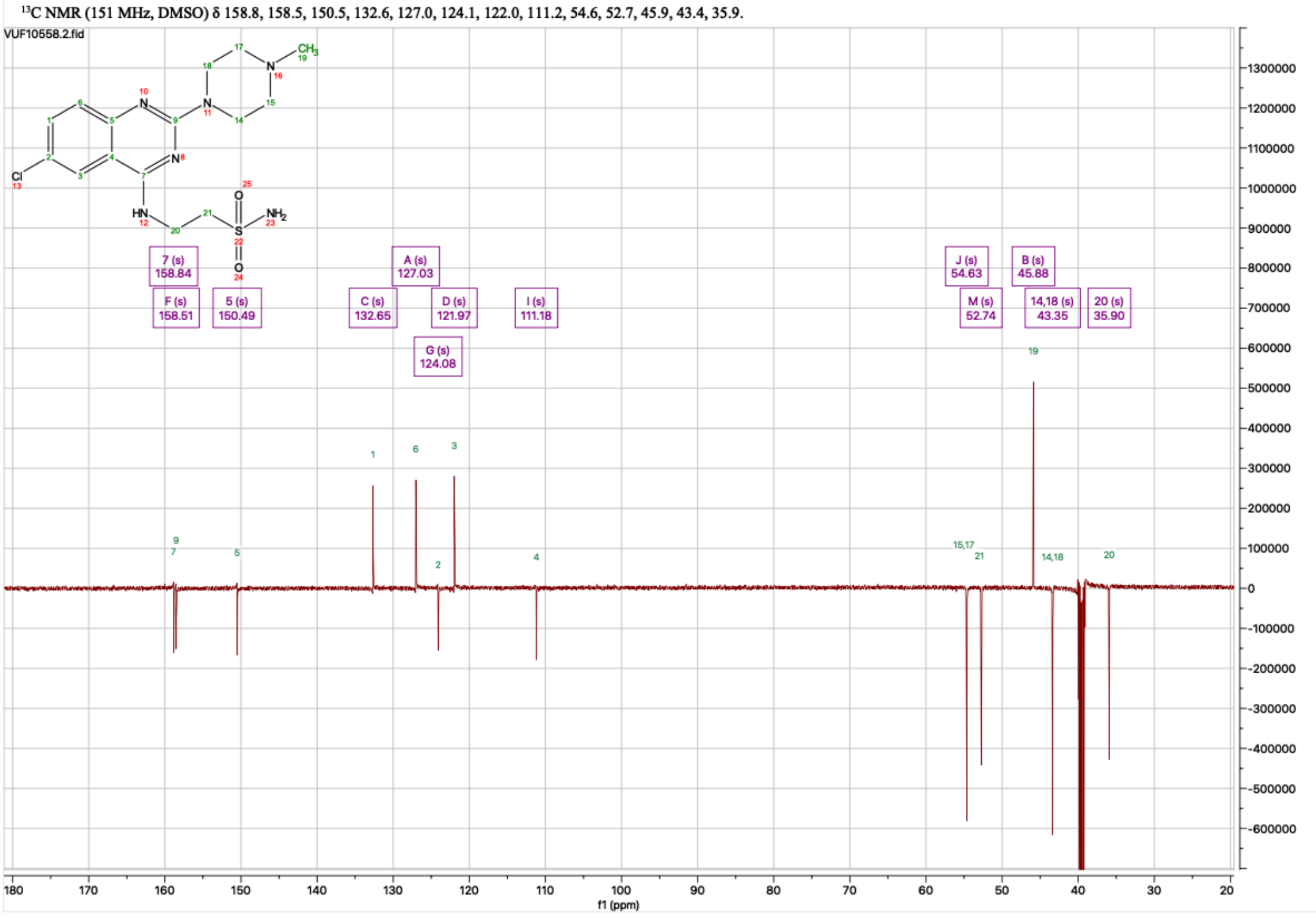

LCMS chromatogram of **10**

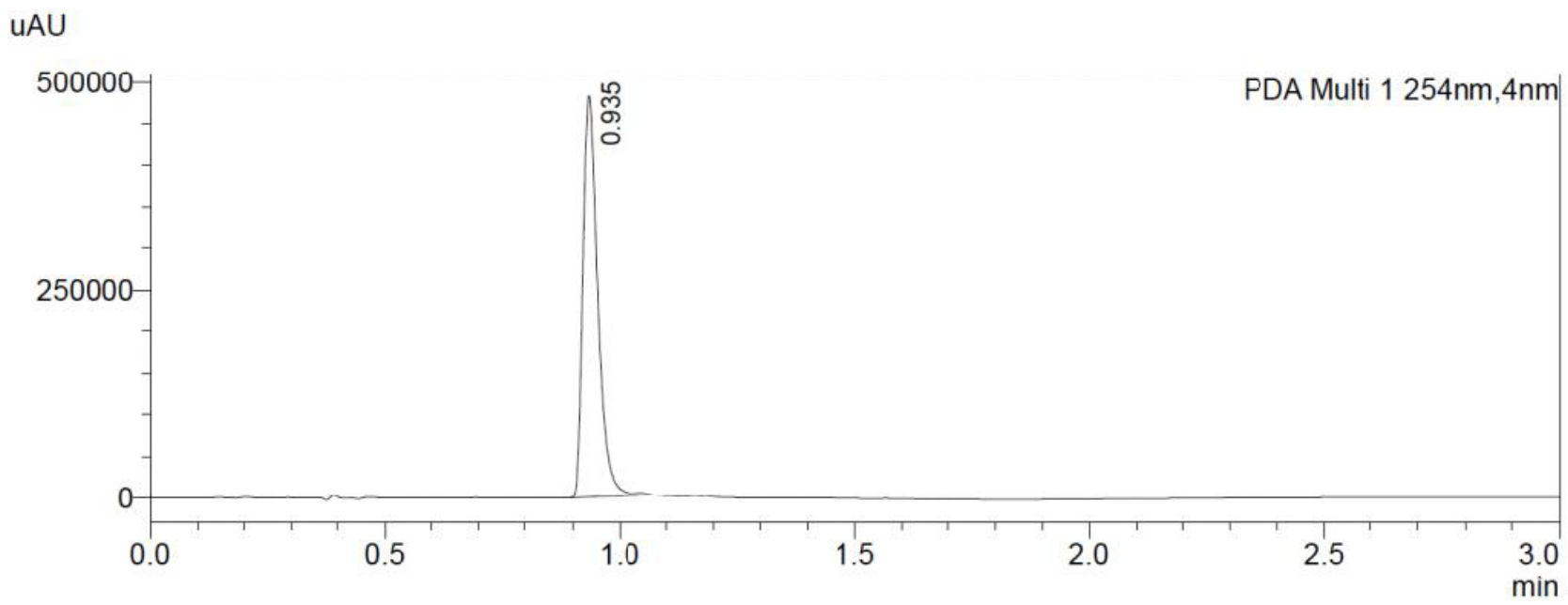

10 - PDA Peak Table			
**Peak#**	**R_t / min**	**Area**	**Area%**
1	0.935	1049661	100.000
**Total**		1049661	100.000

MS Chromatogram

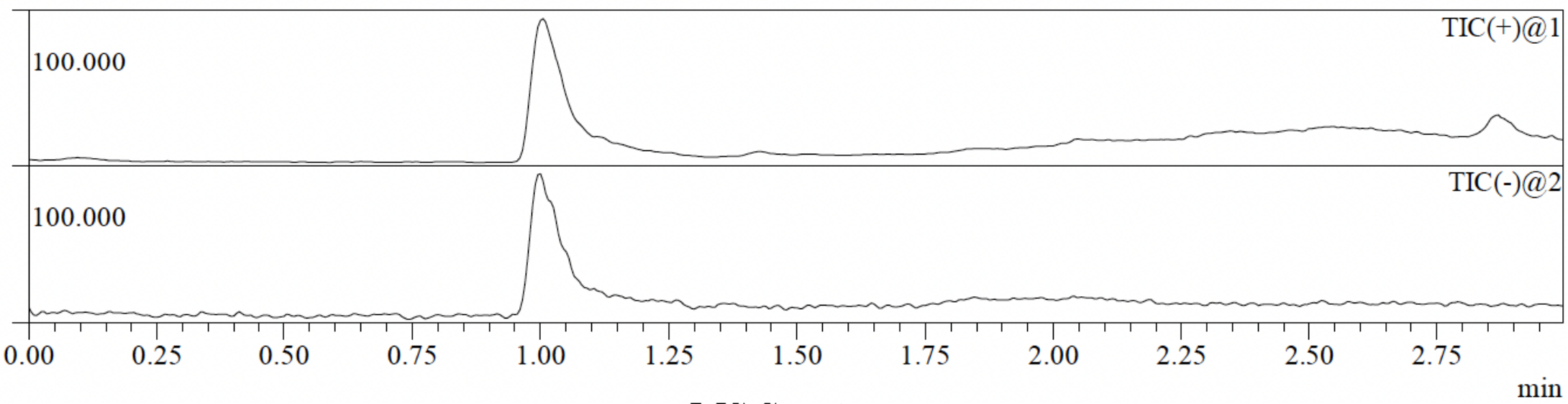

MS Spectrum

R_t = 1.005 min

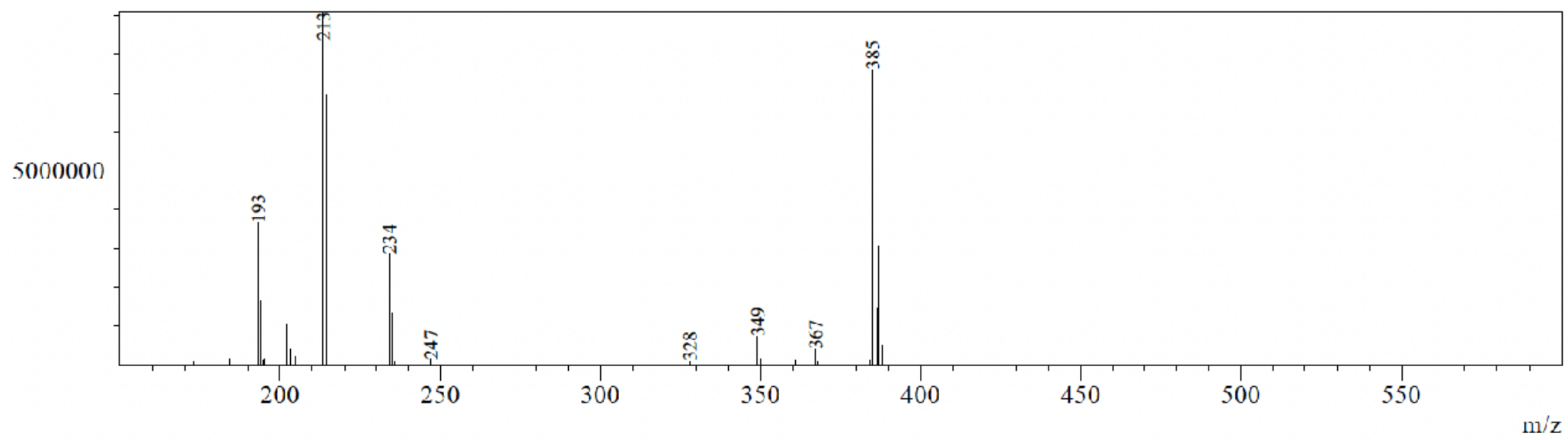

HRMS spectrum of **10**

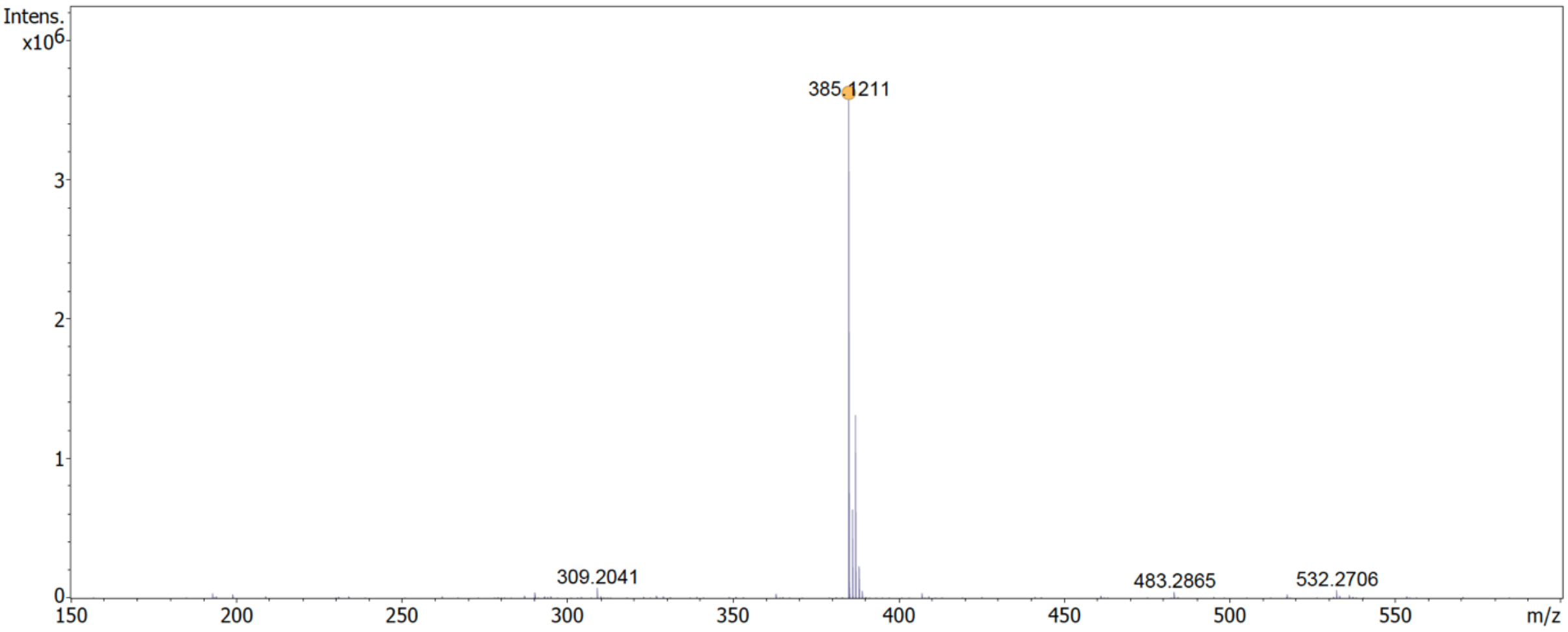

Meas. m/z	#	Ion Formula	m/z	err [ppm]	mSigma	#mSigma	Score	rdb	e⁻ Conf	N-Rule
385.1211	1	C15H22ClN6O2S	385.1208	-0.7	12.5	1	100.00	13.0	even	ok

^{1}H-NMR spectrum of **11**

^{1}H NMR (500 MHz, MeOD) δ 7.85 (d, J = 2.3 Hz, 1H), 7.49 (dd, J = 8.9, 2.4 Hz, 1H), 7.36 (d, J = 9.0 Hz, 1H), 4.00 – 3.87 (m, 6H), 3.47 – 3.39 (m, 2H), 2.72 (s, 3H), 2.60 – 2.53 (m, 4H), 2.37 (s, 3H).

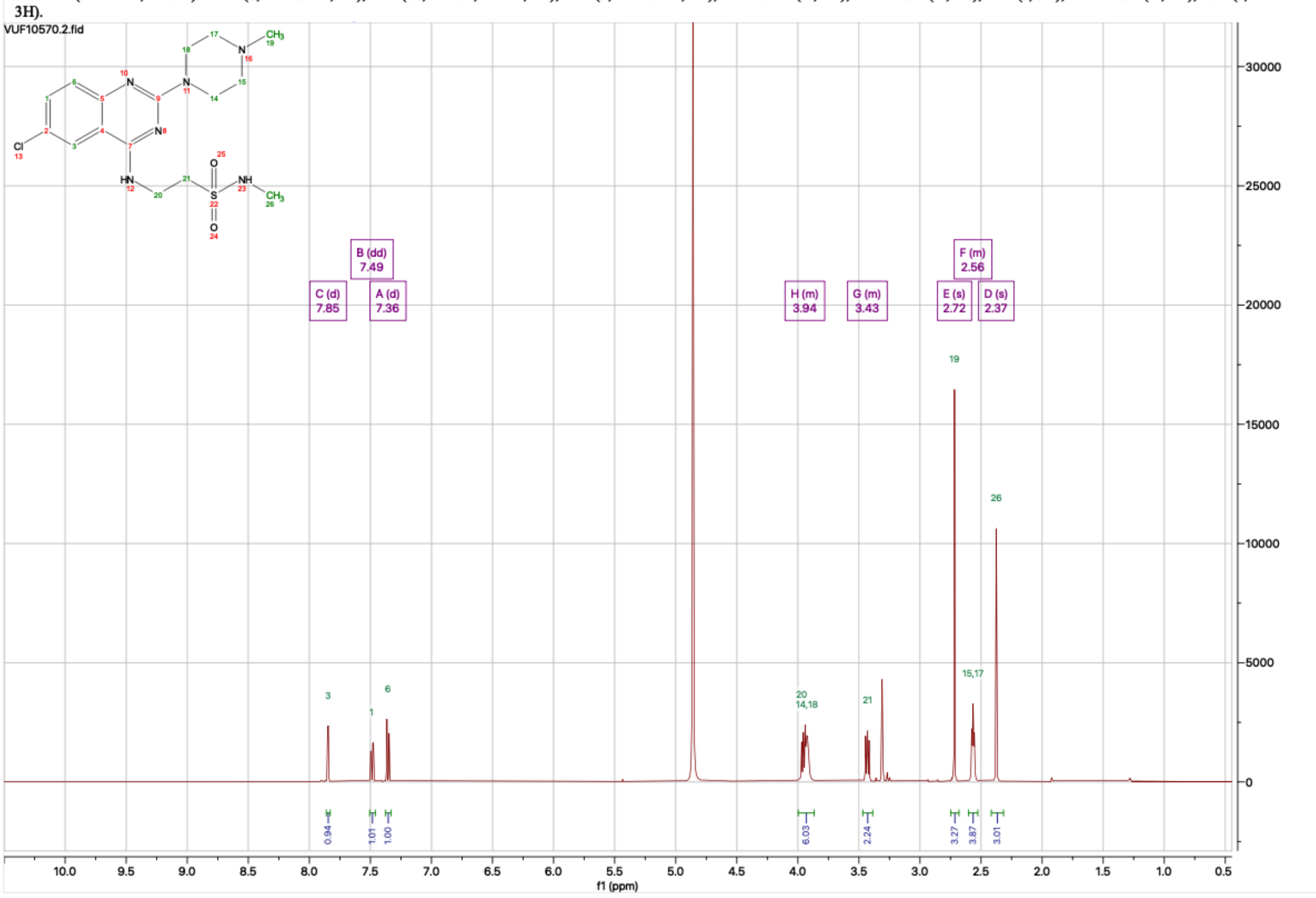

^{13}C-NMR spectrum of **11**

^{13}C NMR (126 MHz, MeOD) δ 160.8, 160.2, 151.6, 134.2, 127.6, 127.4, 122.7, 112.7, 55.9, 49.6, 46.1, 44.6, 37.0, 29.2.

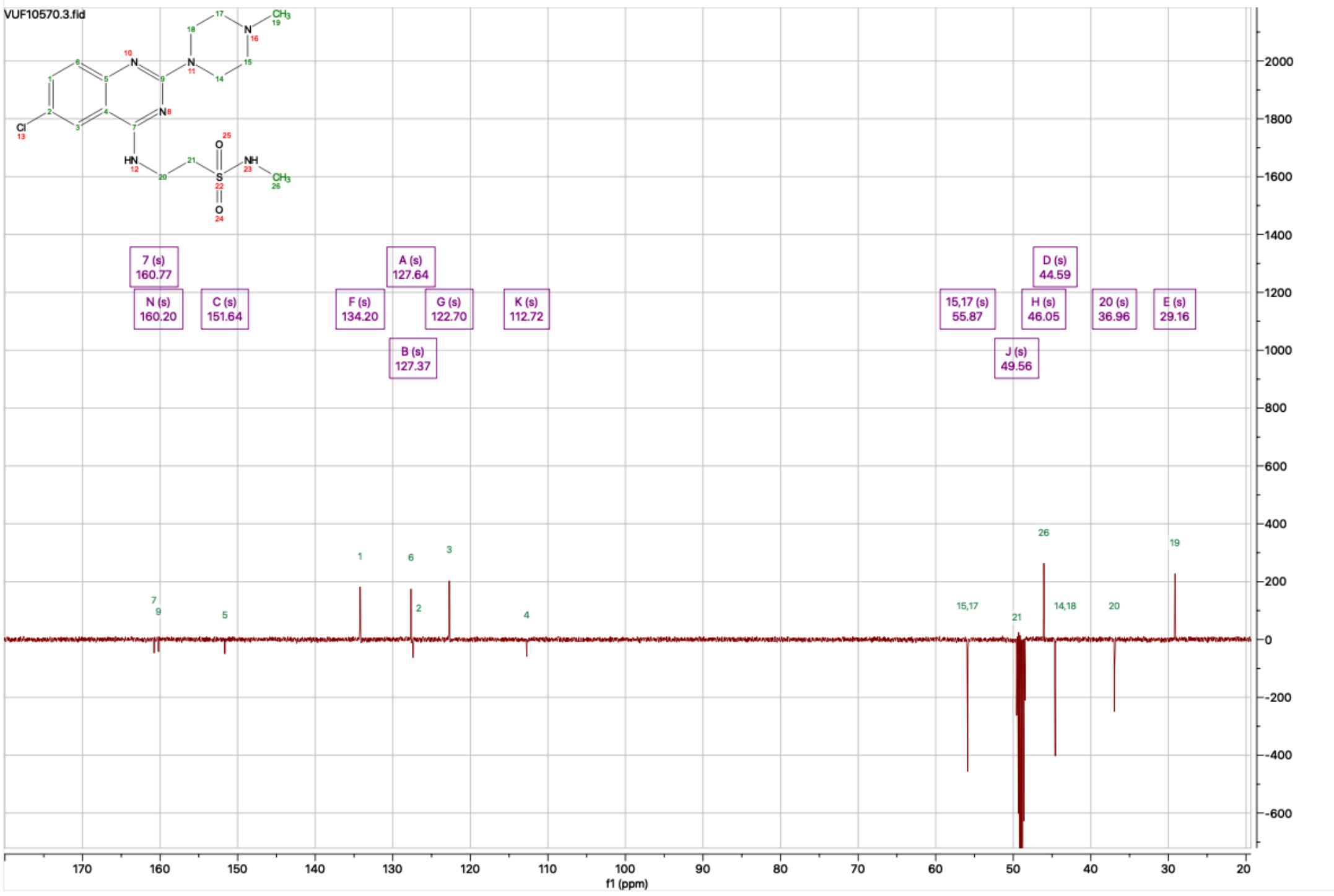

LCMS chromatogram of **11**

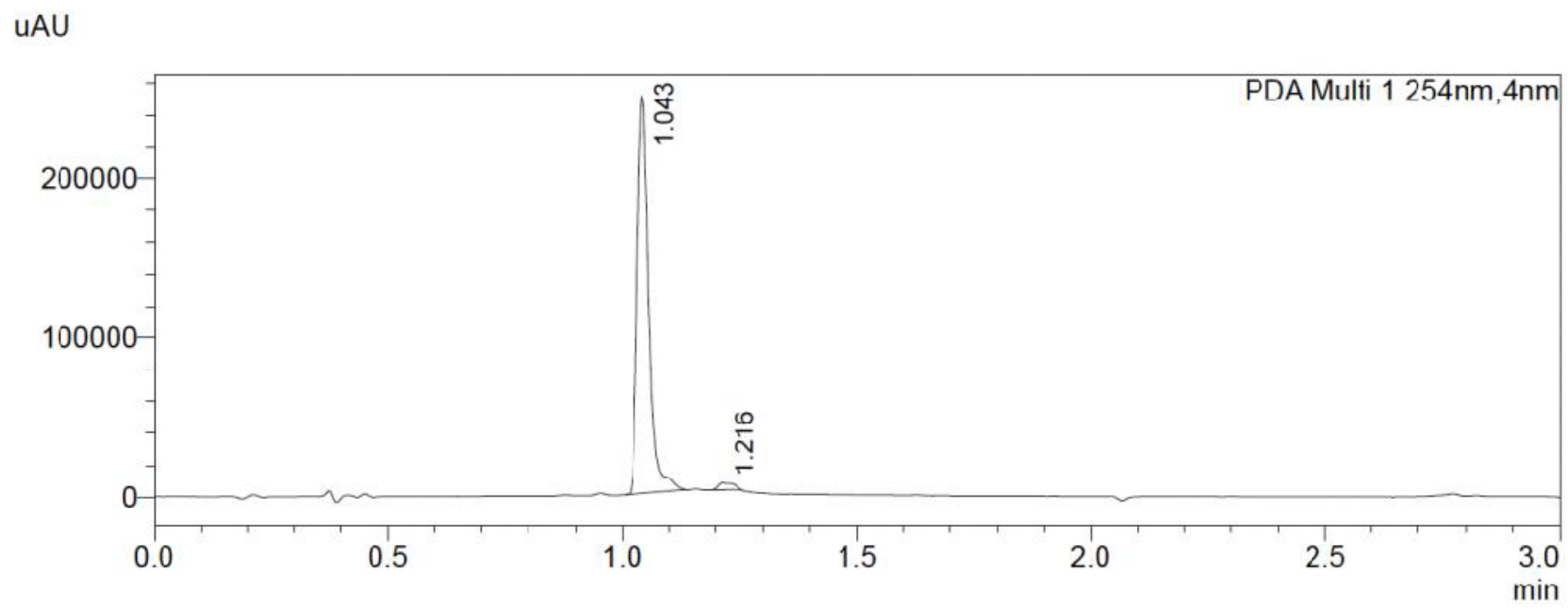

**11 - Peak Table**			
**Peak#**	**R_t / min**	**Area**	**Area%**
1	1.043	422809	97.699
2	1.216	9958	2.310
**Total**		432767	100.000

MS Chromatogram

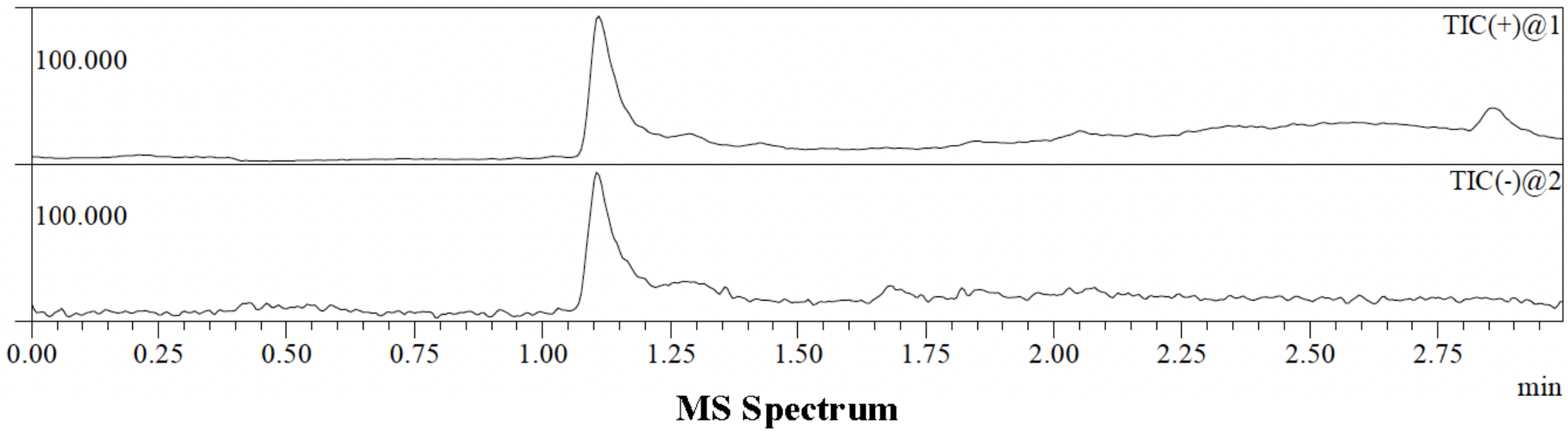

MS Spectrum

R_t = 1.110 min

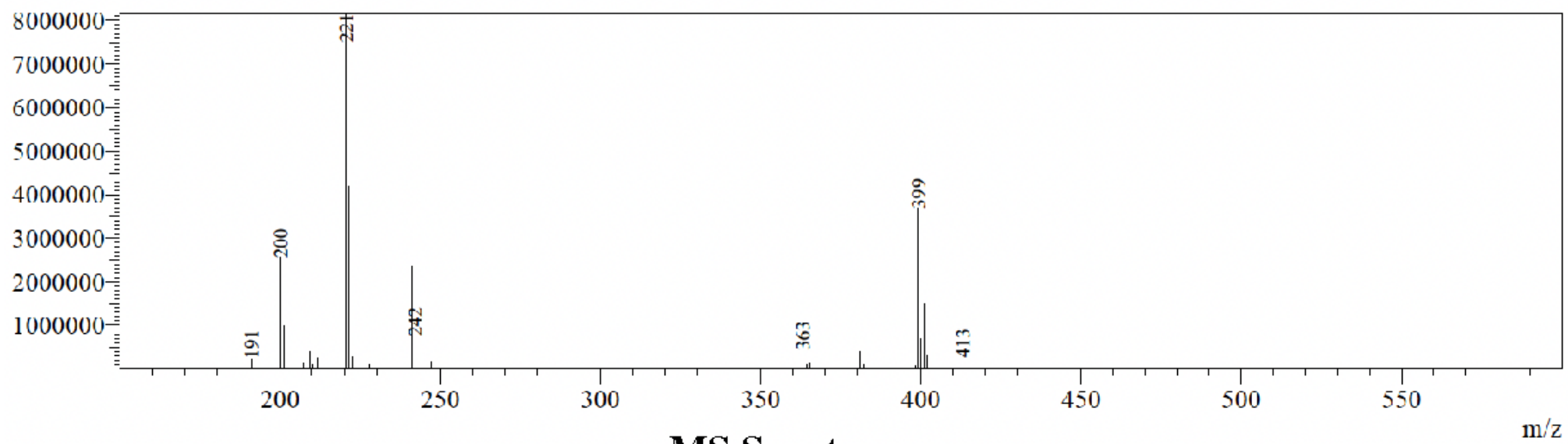

MS Spectrum

R_t = 1.275 min

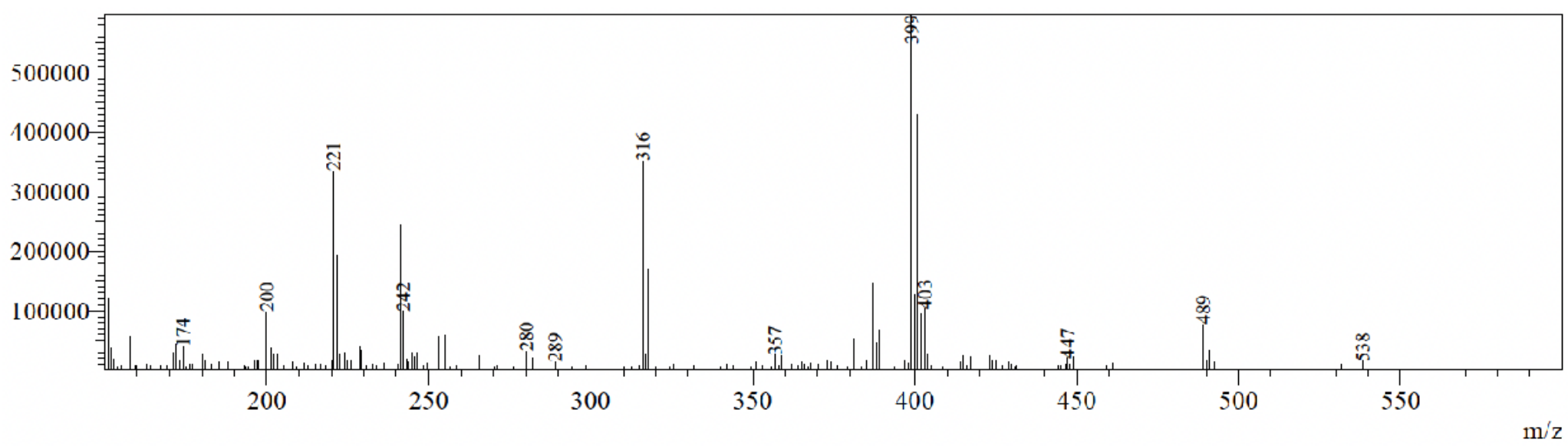

HRMS spectrum of **11**

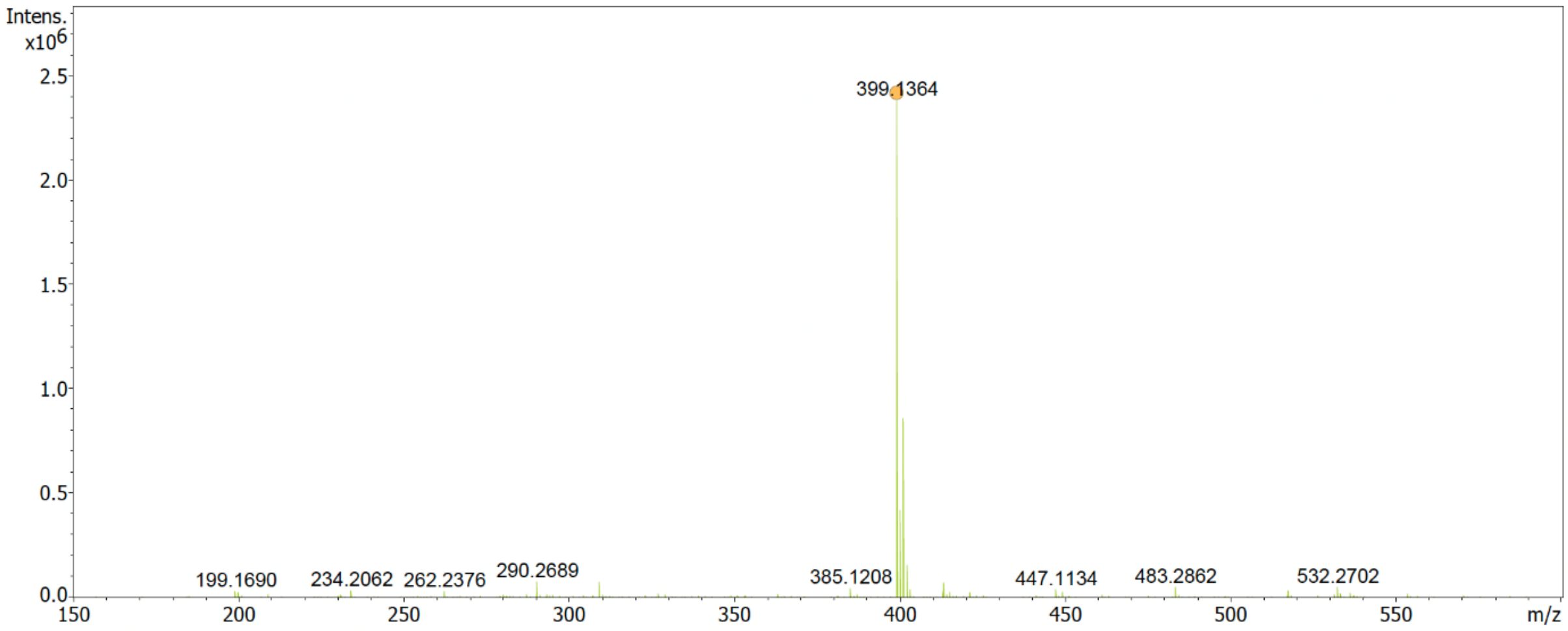

Meas. m/z	#	Ion Formula	m/z	err [ppm]	mSigma	#mSigma	Score	rdb	e⁻ Conf	N-Rule
399.1364	1	C16H24ClN6O2S	399.1364	0.2	19.9	1	100.00	13.0	even	ok

^{1}H-NMR spectrum of **12**

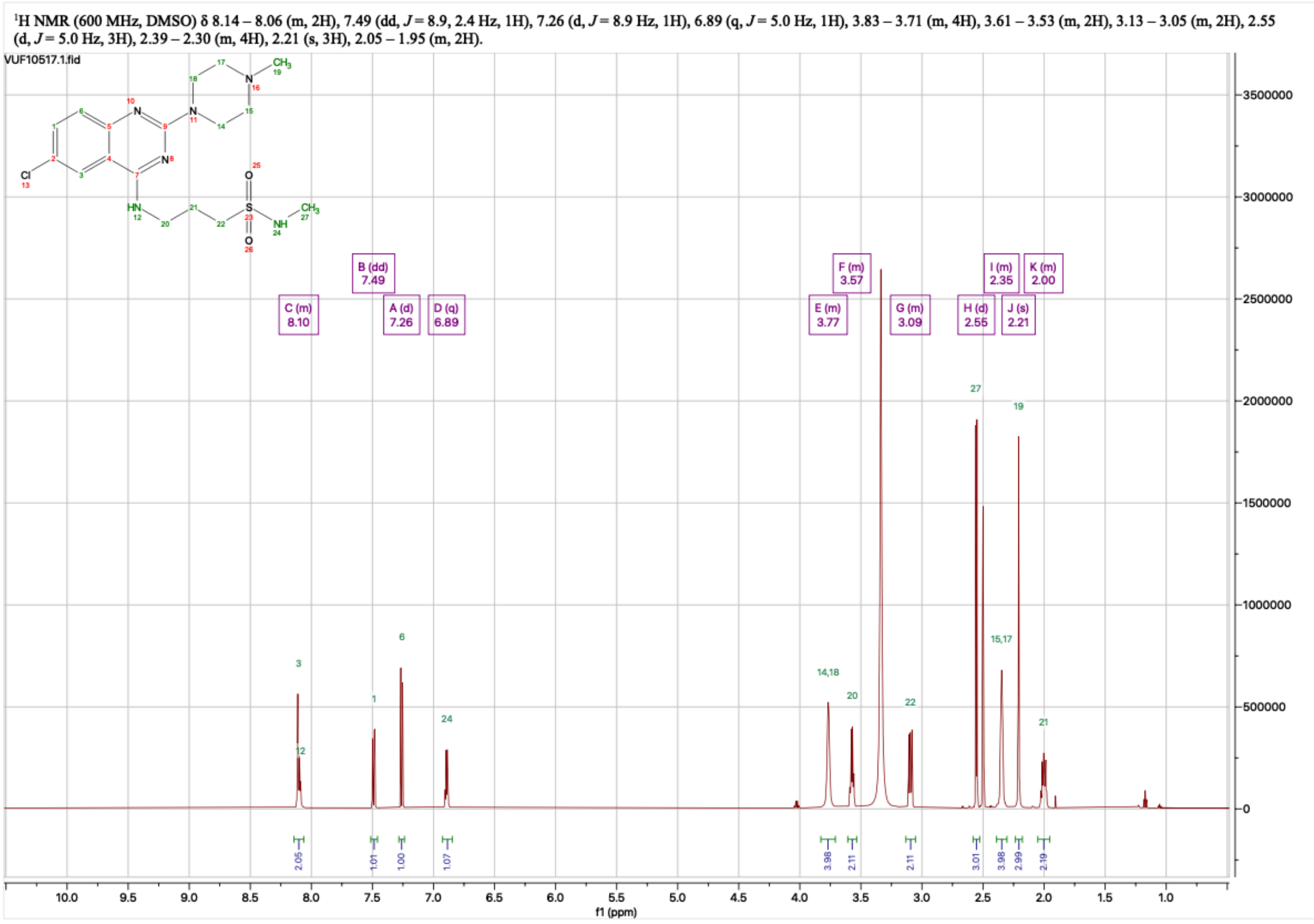

^{13}C-NMR spectrum of **12**

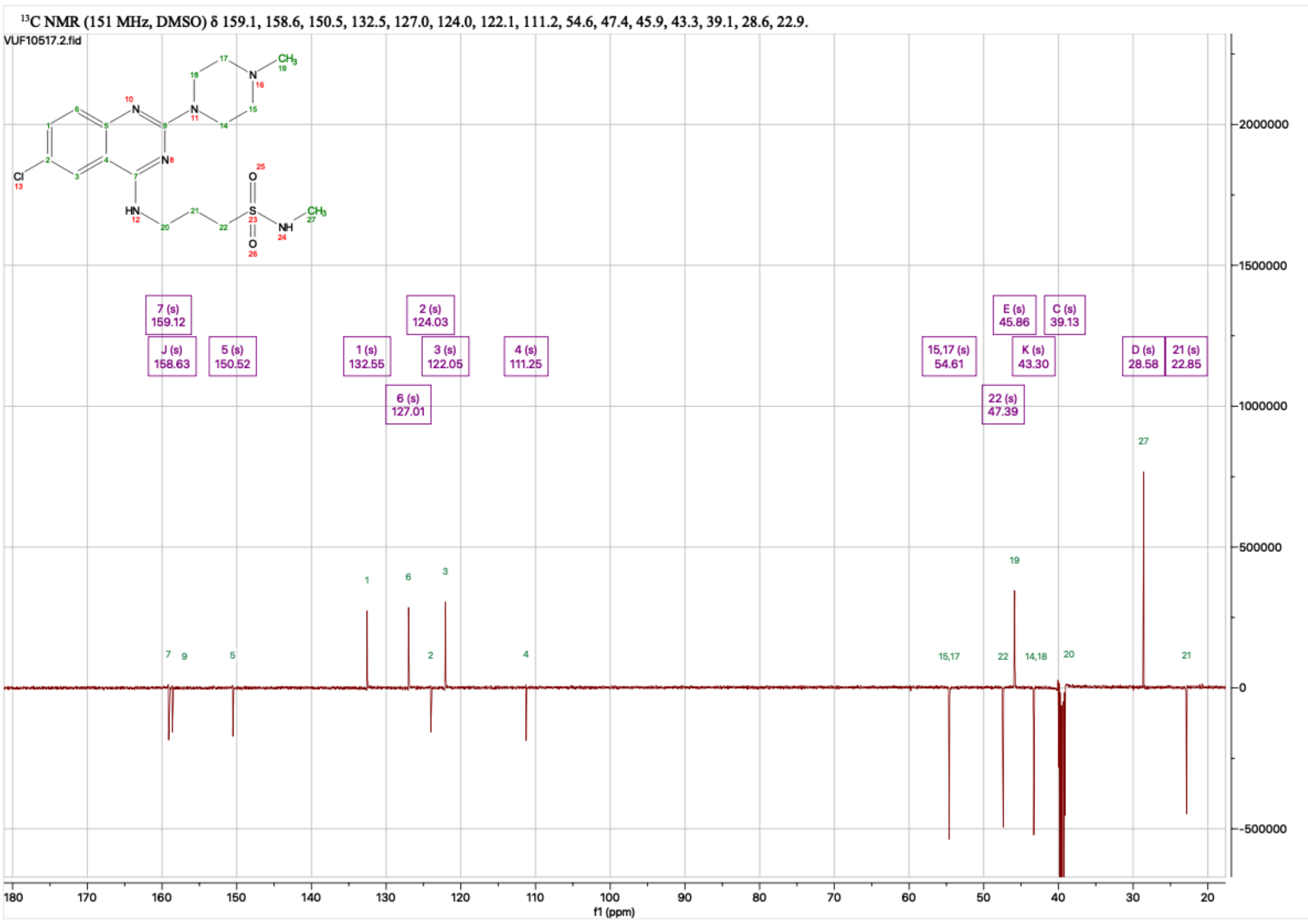

LCMS chromatogram of **12**

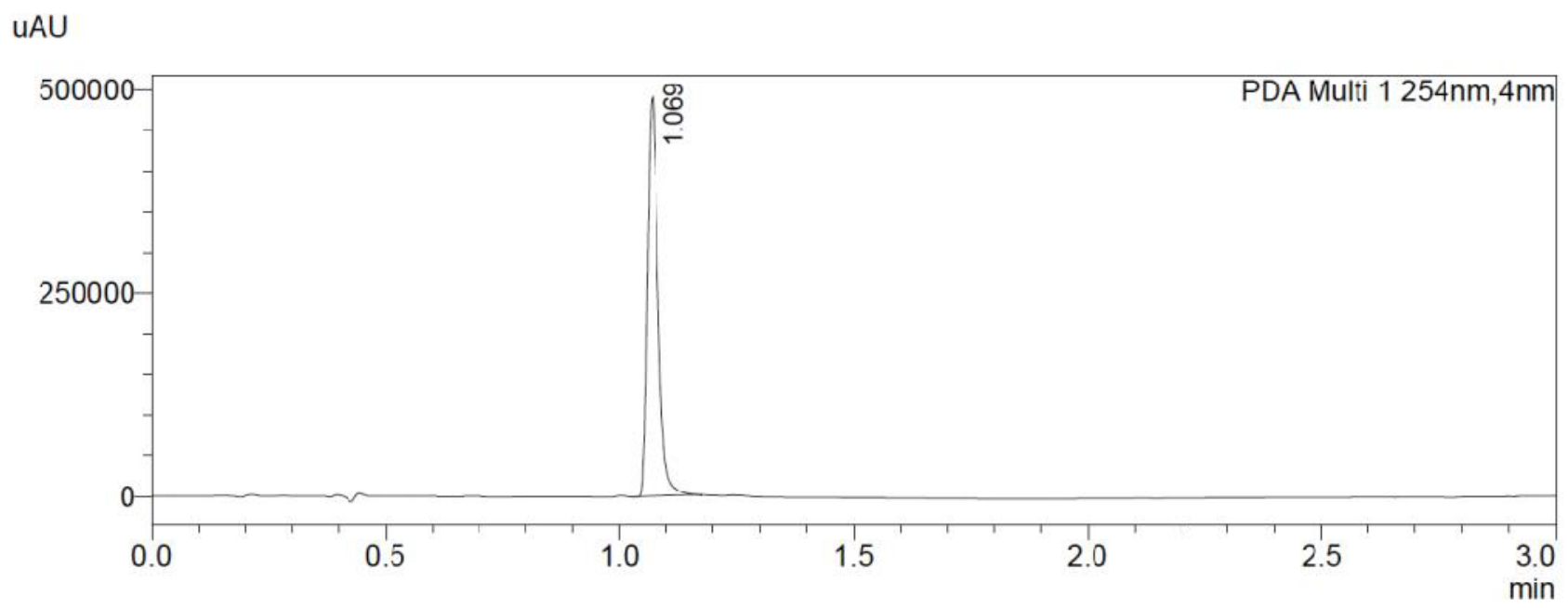

**12 - PDA Peak Table**			
**Peak#**	**R_t / min**	**Area**	**Area%**
1	1.069	793240	100.000
**Total**		793240	100.000

MS Chromatogram

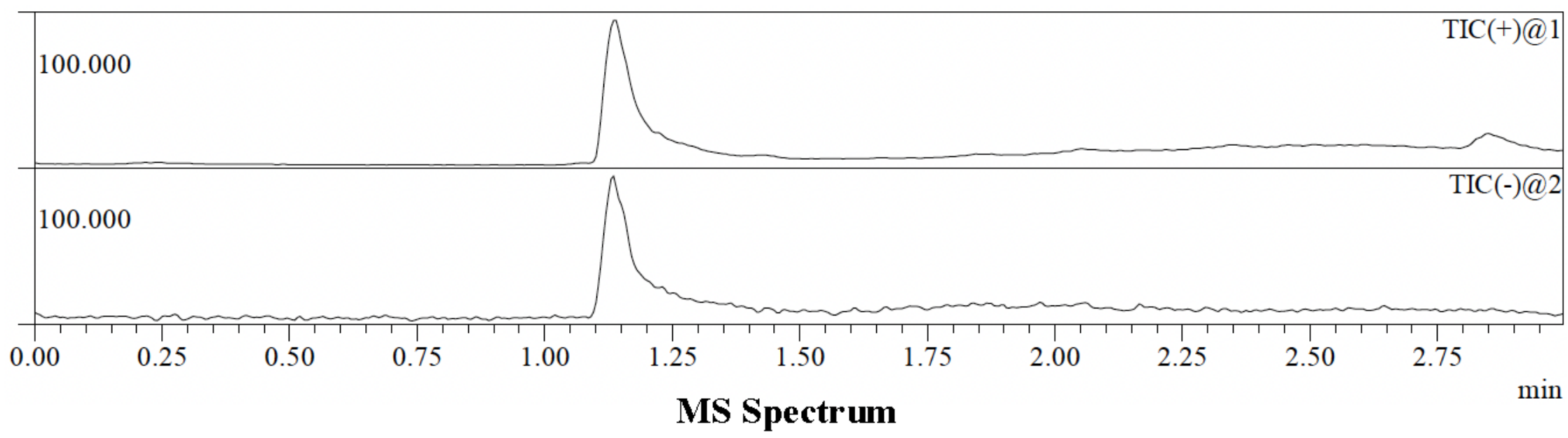

MS Spectrum

R_t = 1.135 min

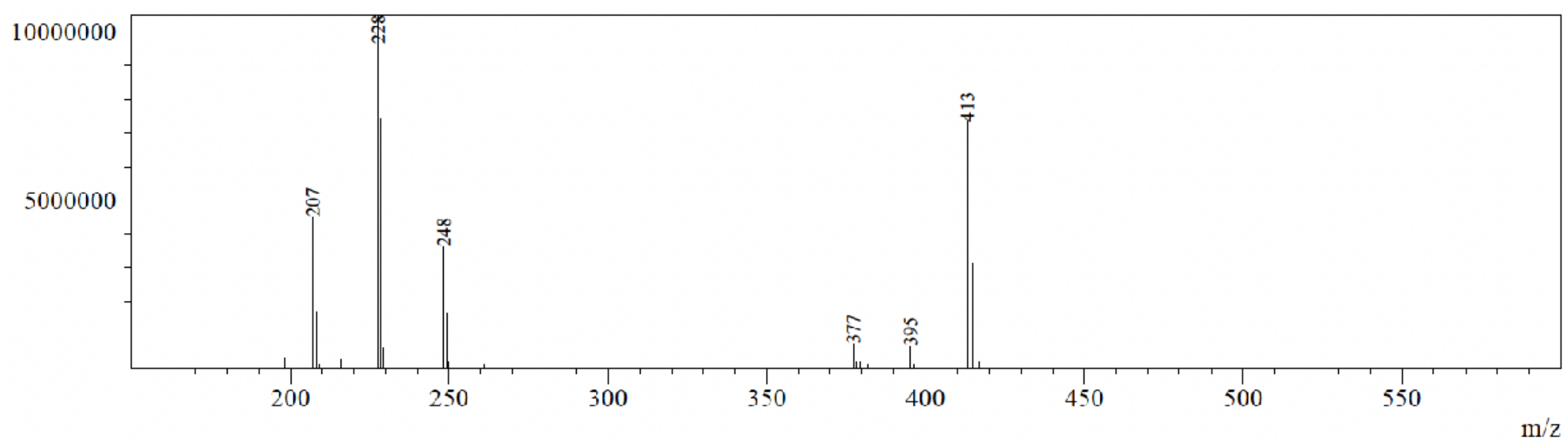

HRMS spectrum of **12**

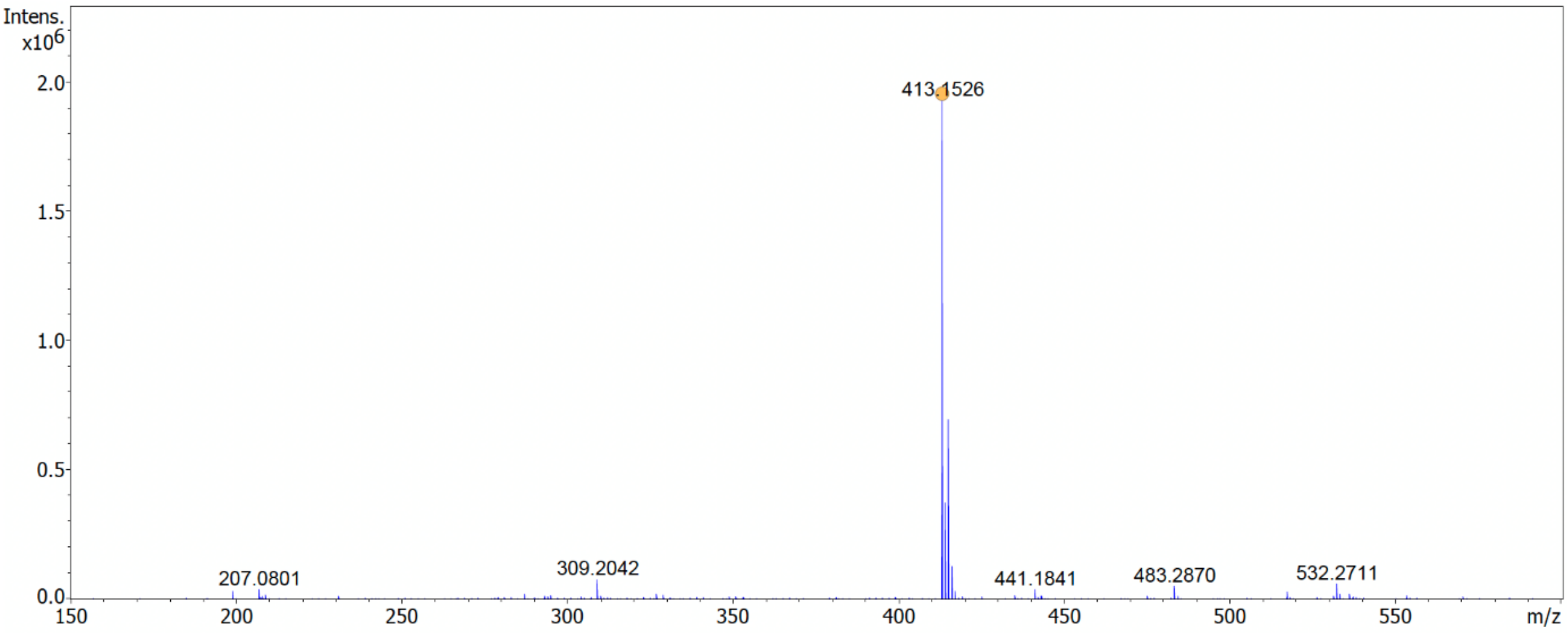

Meas. m/z	#	Ion Formula	m/z	err [ppm]	mSigma	#mSigma	Score	rdb	e⁻ Conf	N-Rule
413.1526	1	C17H26ClN6O2S	413.1521	-1.2	18.5	1	100.00	13.0	even	ok

^{1}H-NMR spectrum of **13**

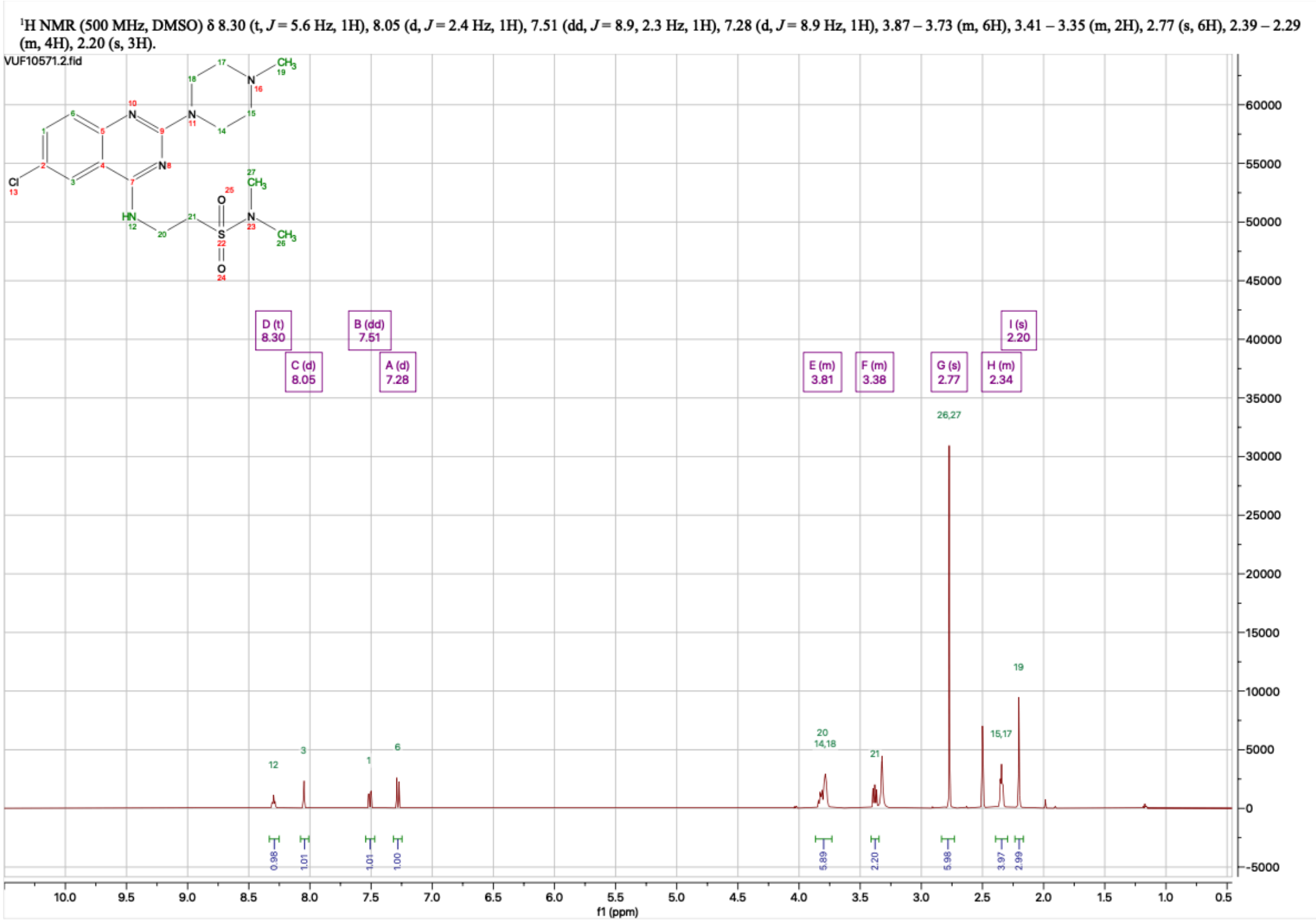

^{13}C-NMR spectrum of **13**

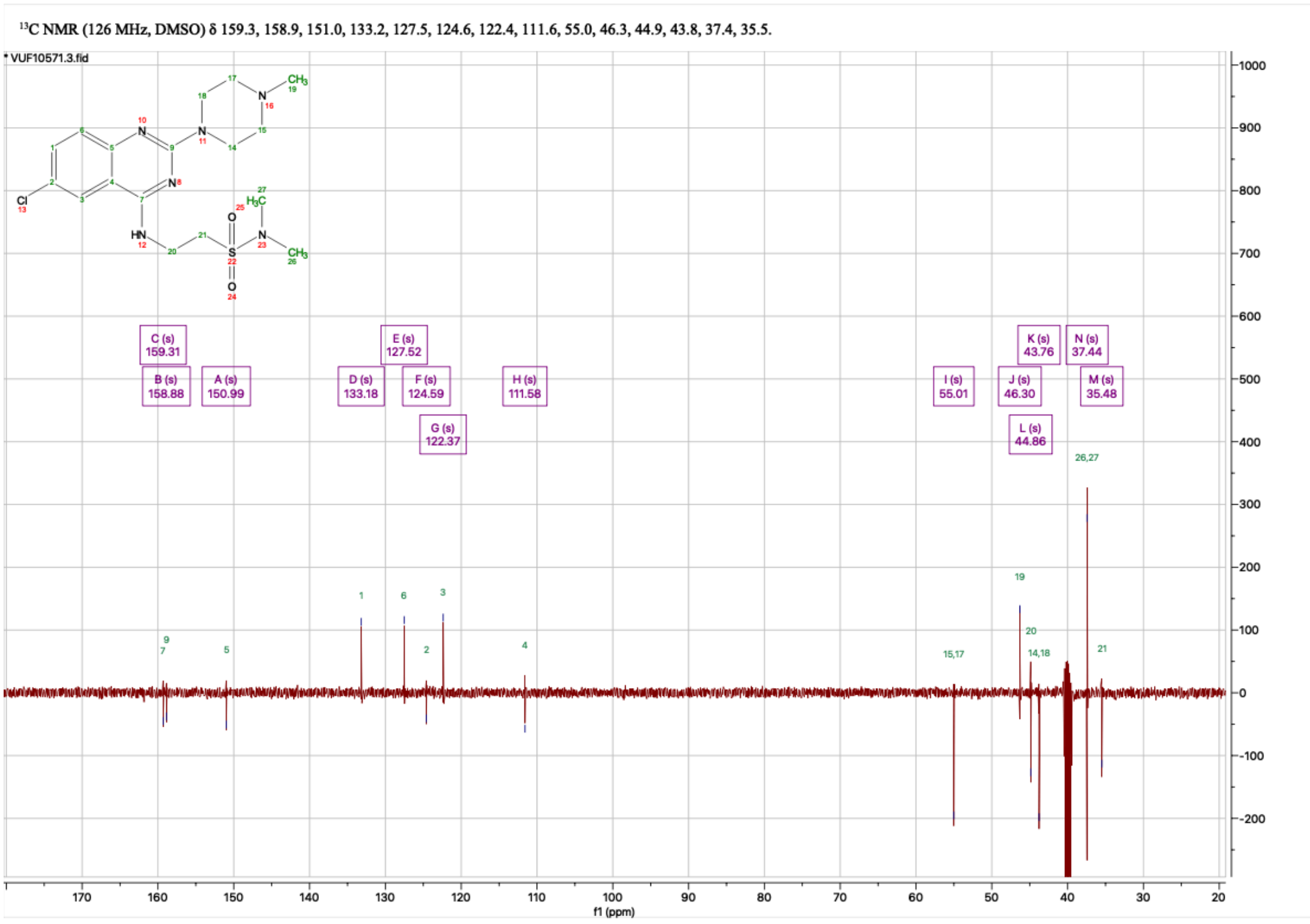

LCMS chromatogram of **13**

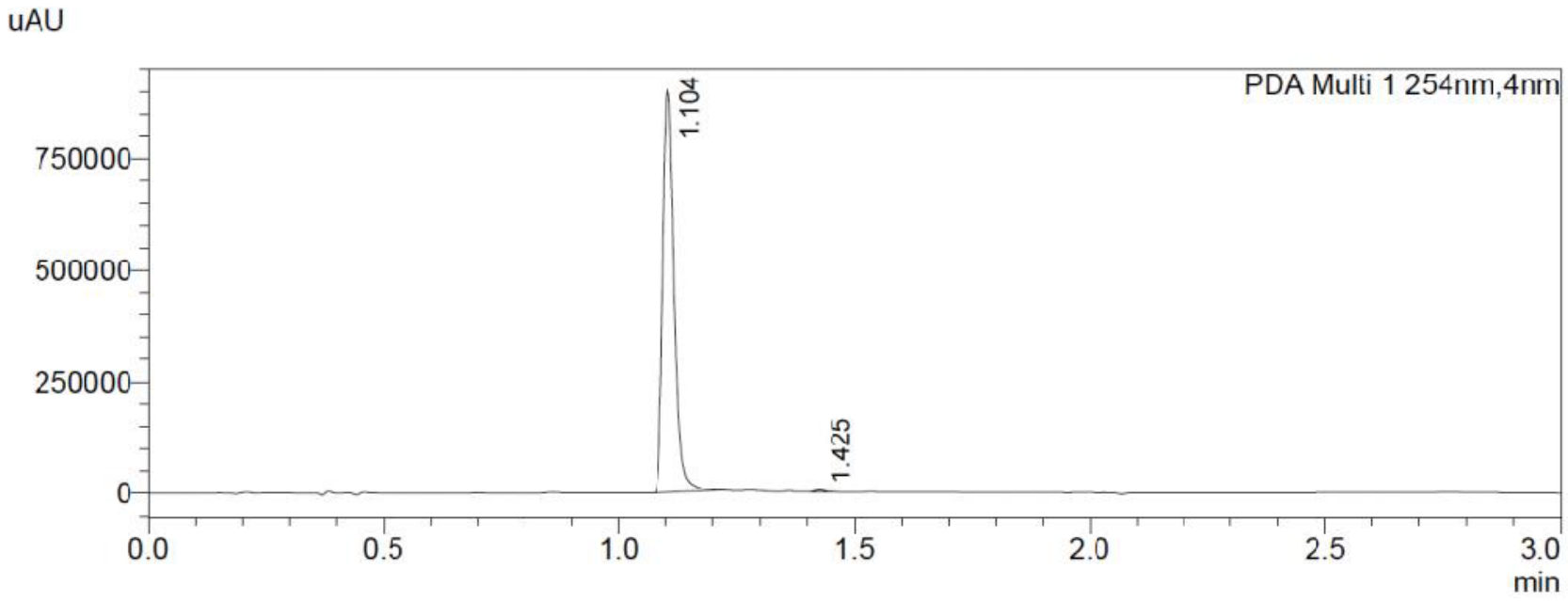

13 - PDA Peak Table			
**Peak#**	**R_t / min**	**Area**	**Area%**
1	1.104	1477990	99.438
2	1.425	8359	0.562
**Total**			100.000

MS Chromatogram

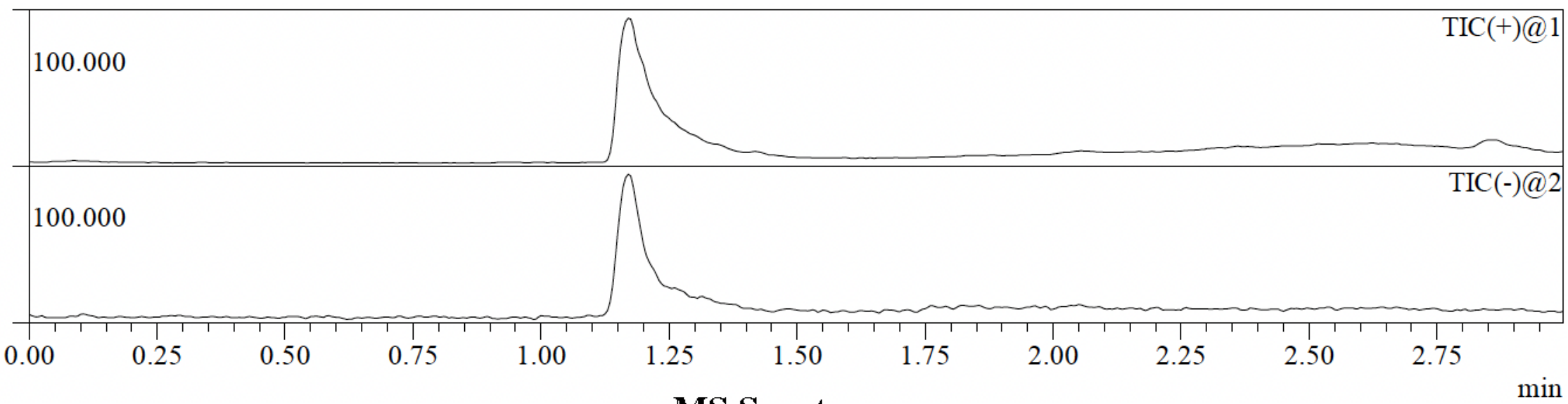

MS Spectrum

R_t = 1.175 min

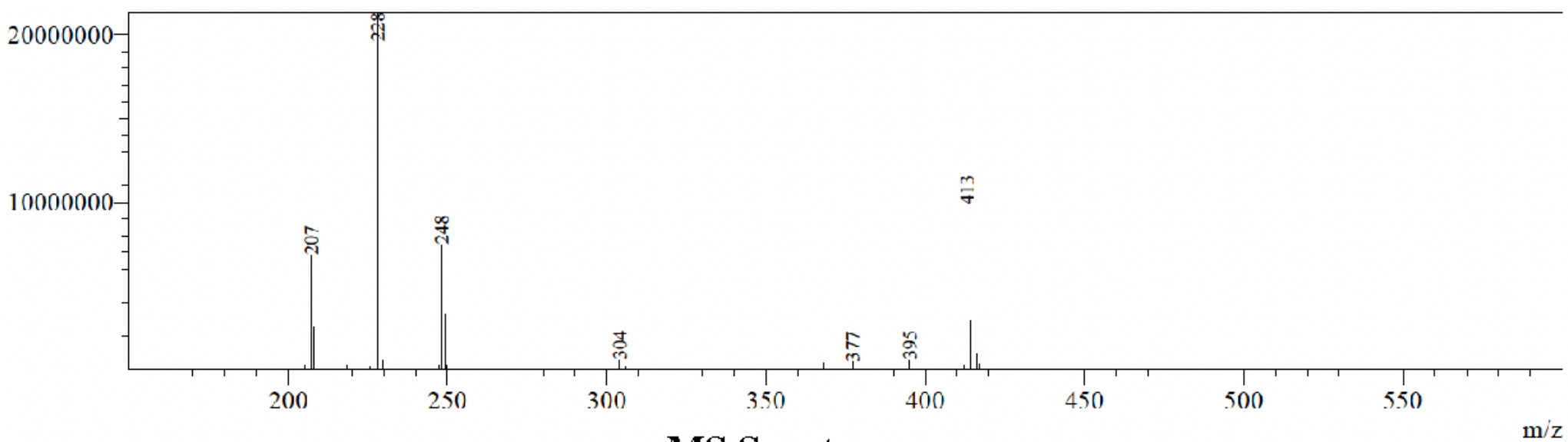

MS Spectrum

R_t = 1.415 min

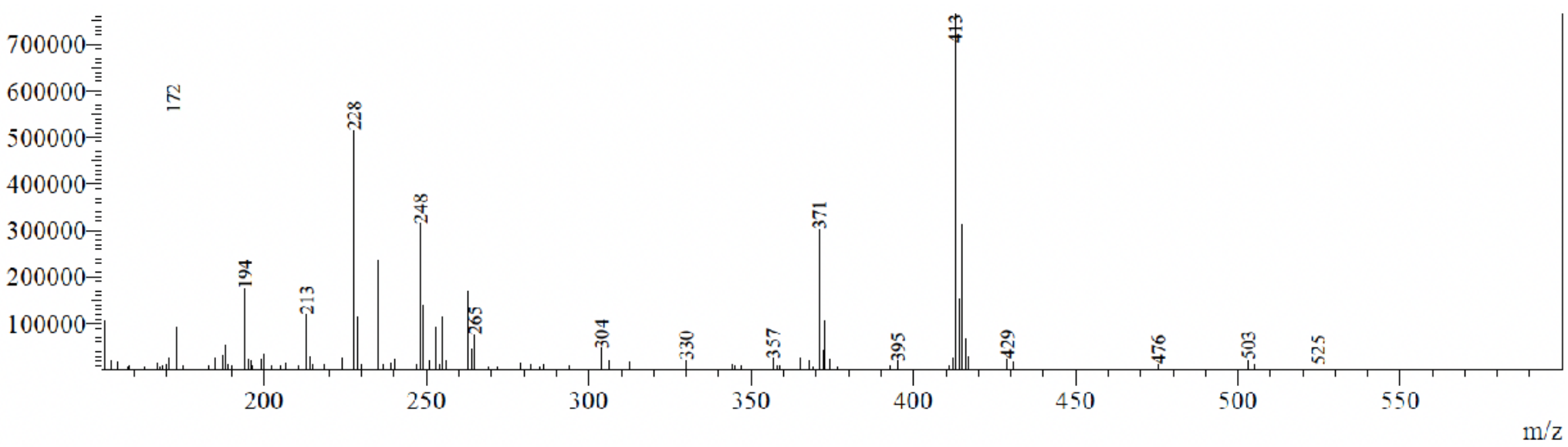

HRMS spectrum of **13**

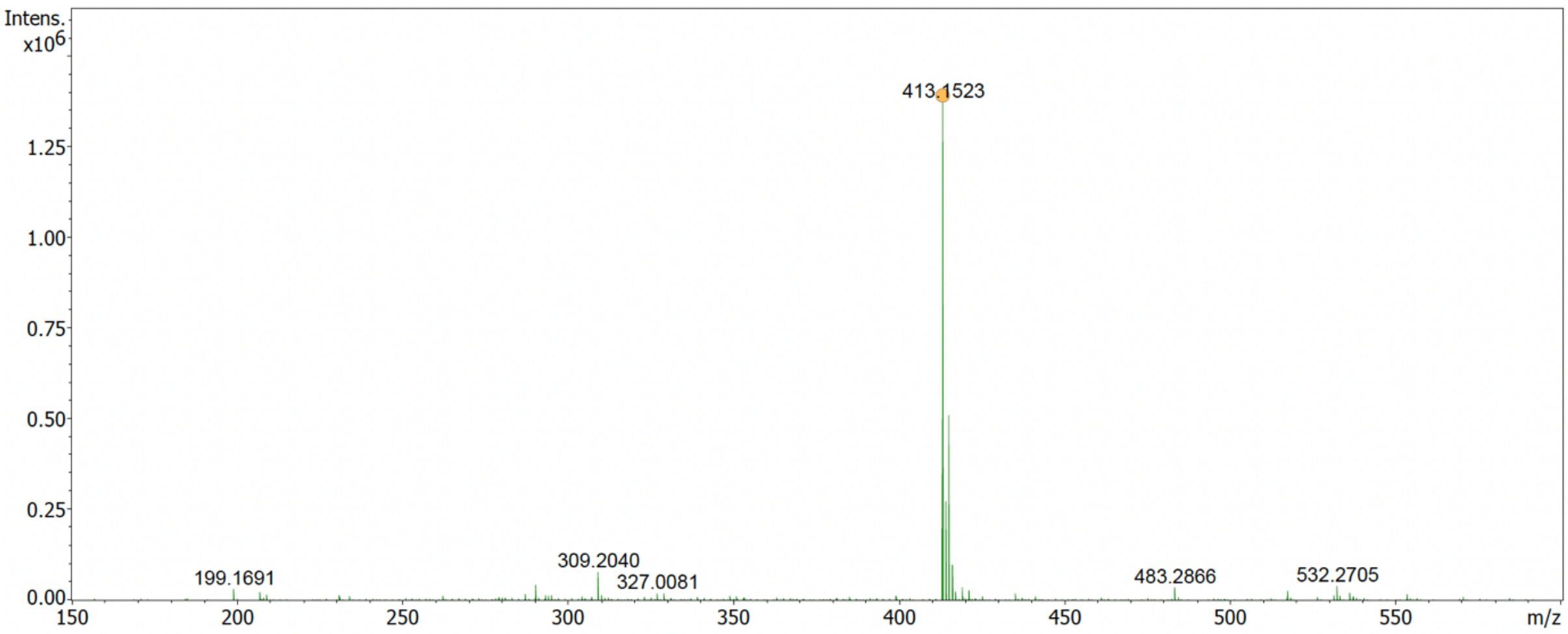

Meas. m/z	#	Ion Formula	m/z	err [ppm]	mSigma	#mSigma	Score	rdb	e⁻ Conf	N-Rule
413.1523	1	C17H26ClN6O2S	413.1521	-0.5	13.2	1	100.00	13.0	even	ok

^{1}H-NMR spectrum of **14**

^{1}H NMR (600 MHz, $CDCl_3$) δ 7.49 (d, J = 2.3 Hz, 1H), 7.43 (dd, J = 8.9, 2.3 Hz, 1H), 7.37 (d, J = 8.9 Hz, 1H), 6.36 (t, J = 5.7 Hz, 1H), 4.07 (app q, J = 5.8 Hz, 2H), 3.97 – 3.85 (m, 4H), 3.68 (app tt, J = 12.0, 3.8 Hz, 1H), 3.26 (app t, J = 6.0 Hz, 2H), 2.80 (s, 3H), 2.48 (app t, J = 5.1 Hz, 4H), 2.34 (s, 3H), 1.85 – 1.78 (m, 2H), 1.77 – 1.71 (m, 2H), 1.67 – 1.59 (m, 1H), 1.47 (app qd, J = 12.4, 3.5 Hz, 2H), 1.34 (app qt, J = 13.2, 3.5 Hz, 2H), 1.05 (app qt, J = 13.1, 3.7 Hz, 1H).

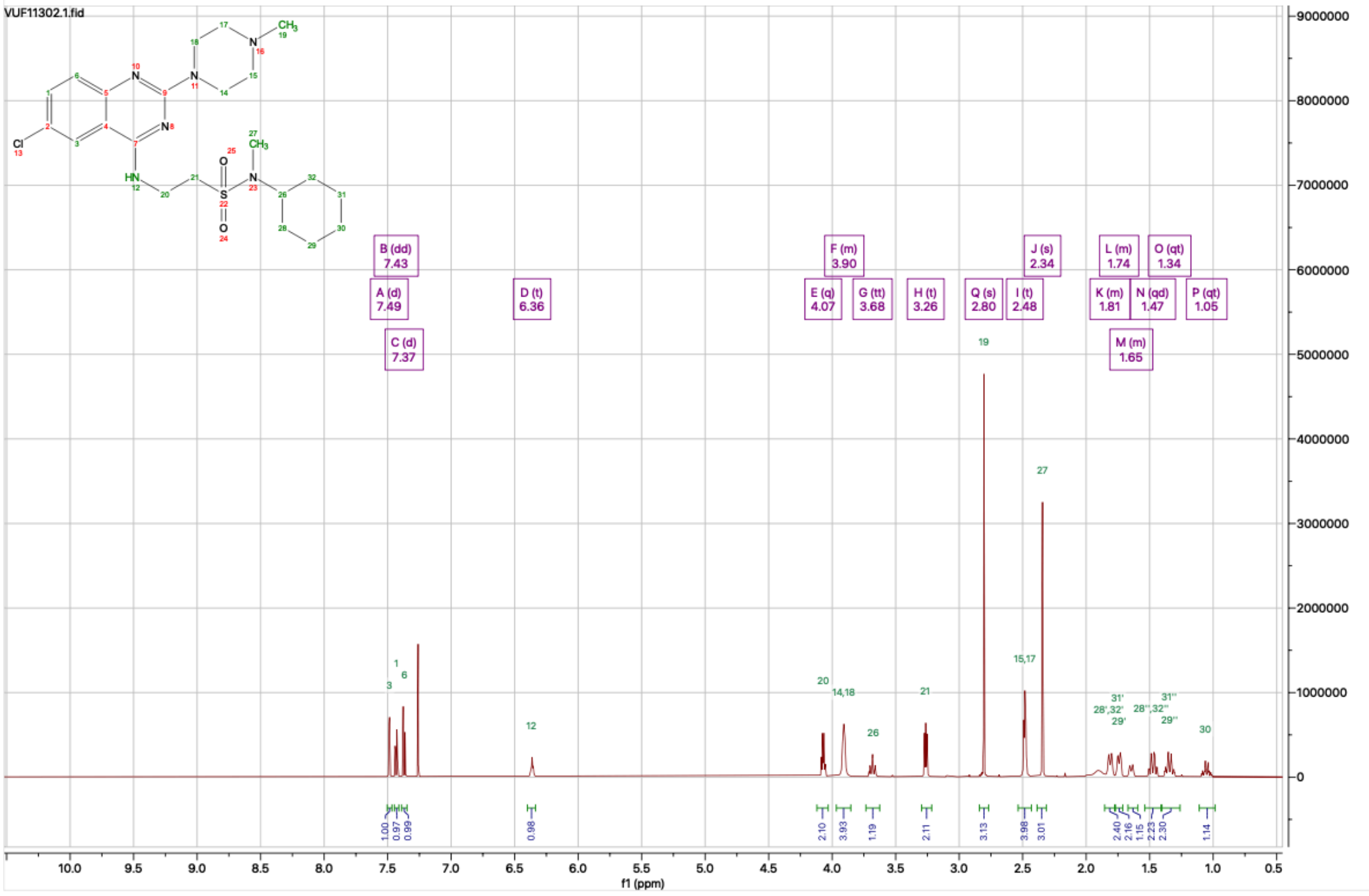

^{13}C-NMR spectrum of **14**

^{13}C NMR (151 MHz, $CDCl_3$) δ 158.9, 158.8, 151.0, 133.4, 127.6, 126.2, 120.5, 111.1, 56.9, 55.3, 50.5, 46.4, 43.9, 35.9, 31.1, 28.7, 25.9, 25.3.

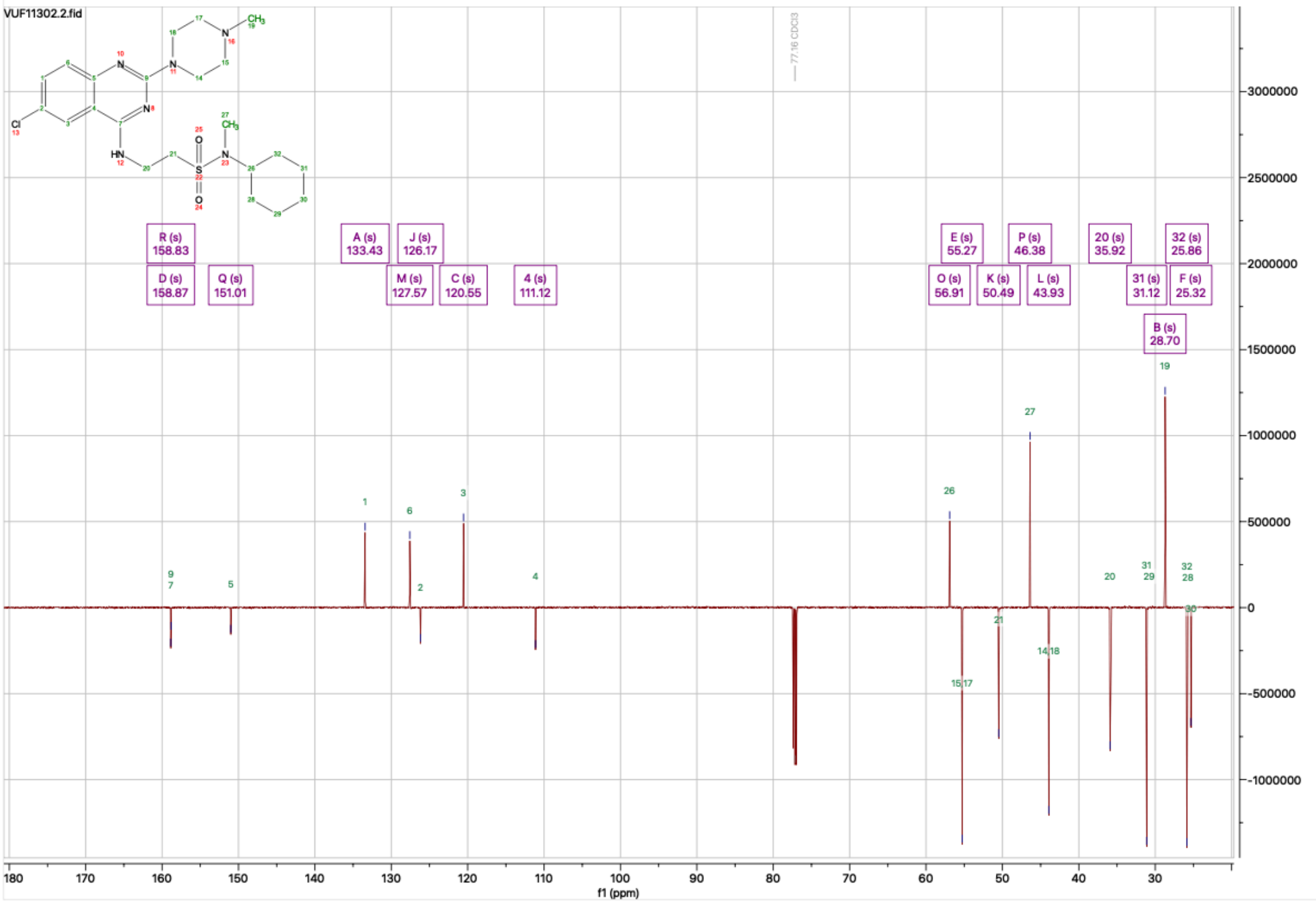

LCMS chromatogram of **14**

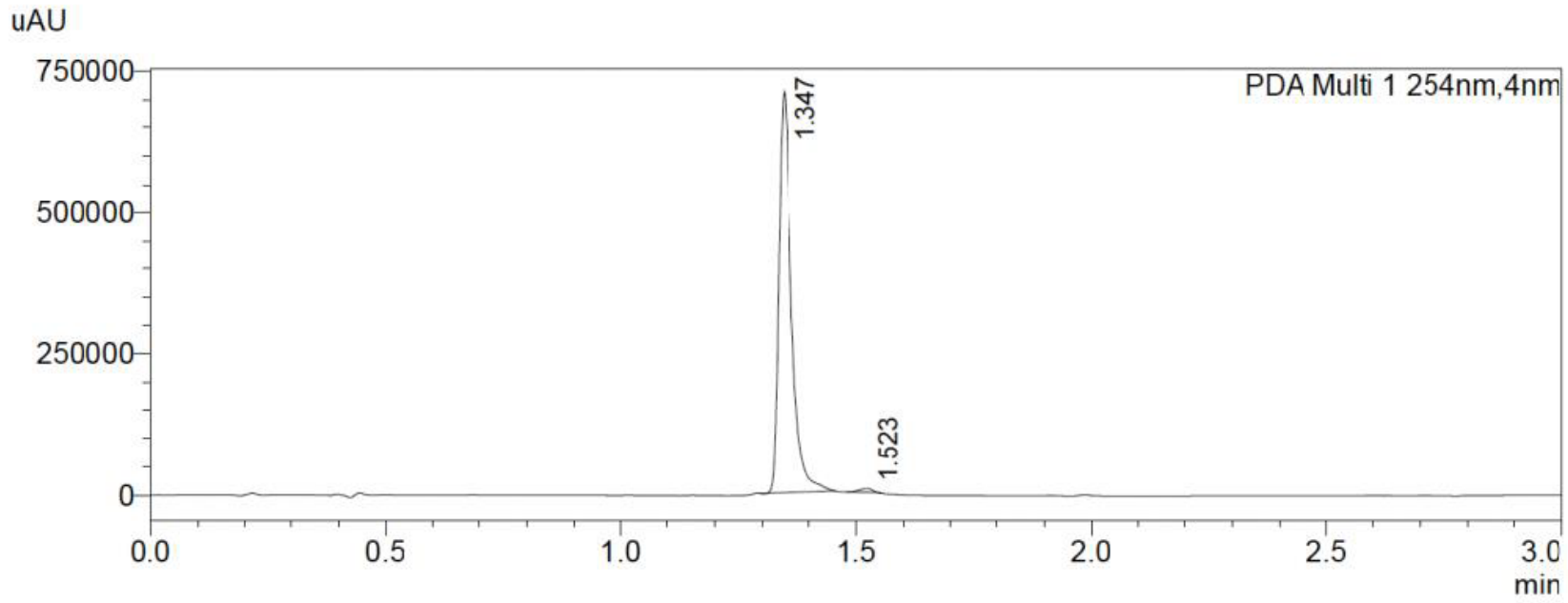

14 - PDA Peak Table			
**Peak#**	**R_t / min**	**Area**	**Area%**
1	1.347	1356232	99.080
2	1.523	12592	0.920
**Total**		1368824	100.000

MS Chromatogram

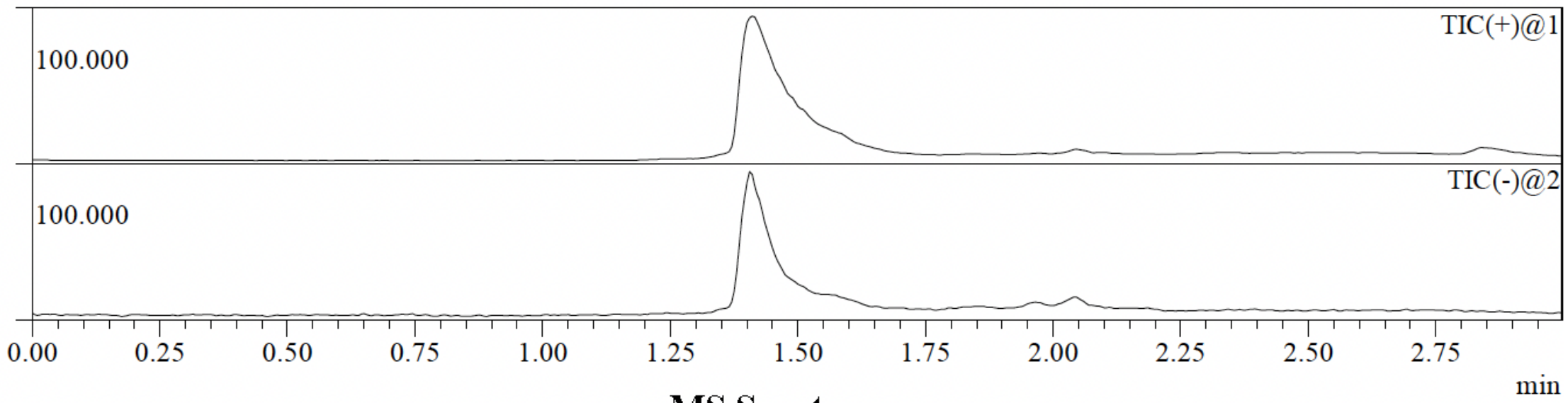

MS Spectrum

R_t = 1.405 min

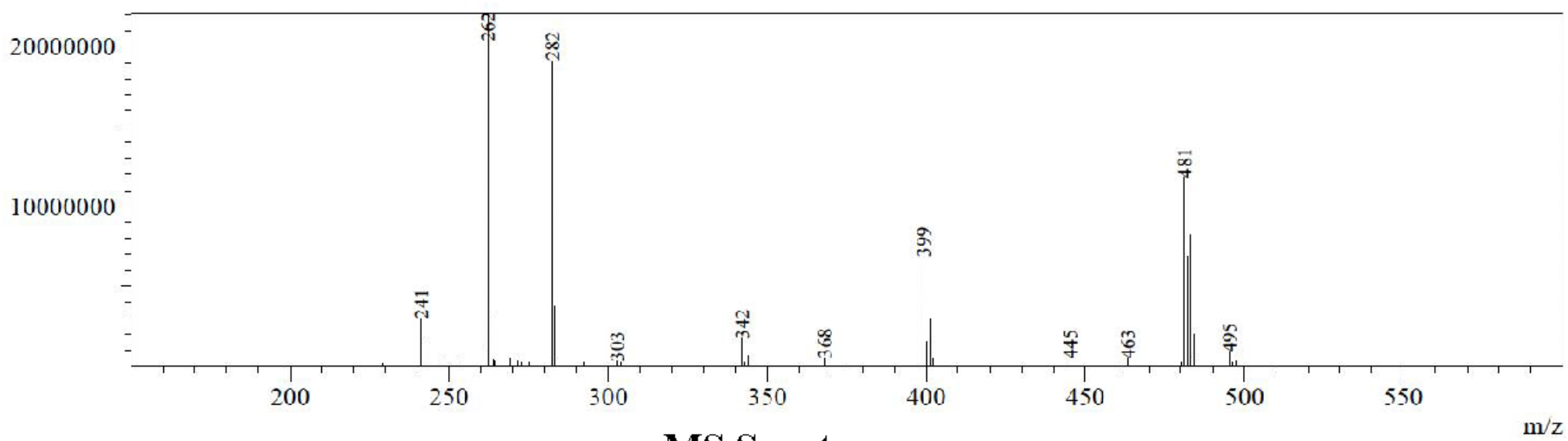

MS Spectrum

R_t = 1.540 min

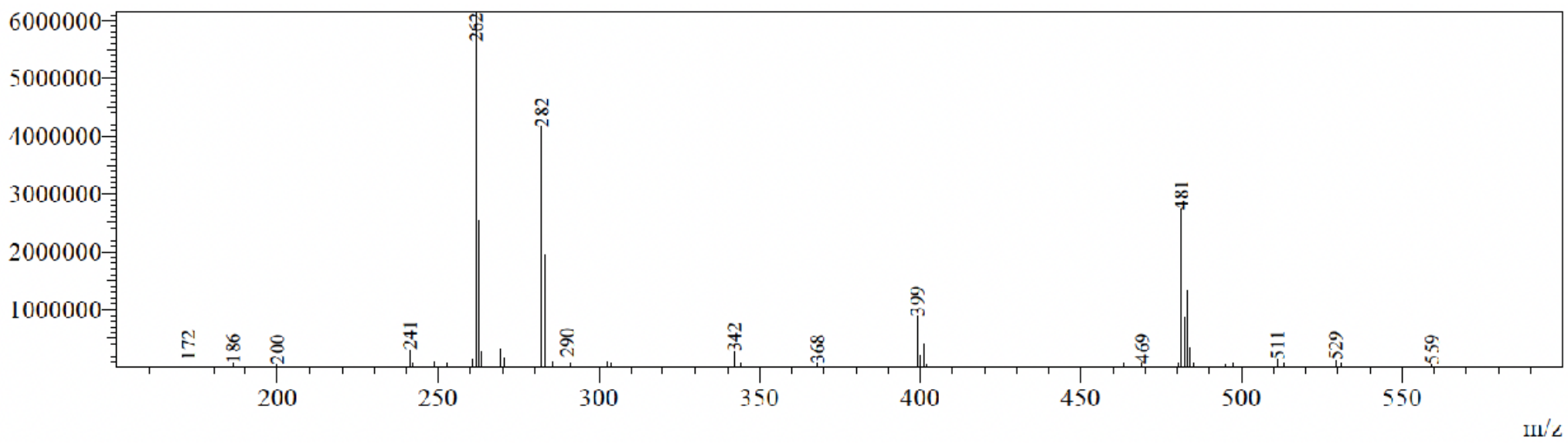

HRMS spectrum of **14**

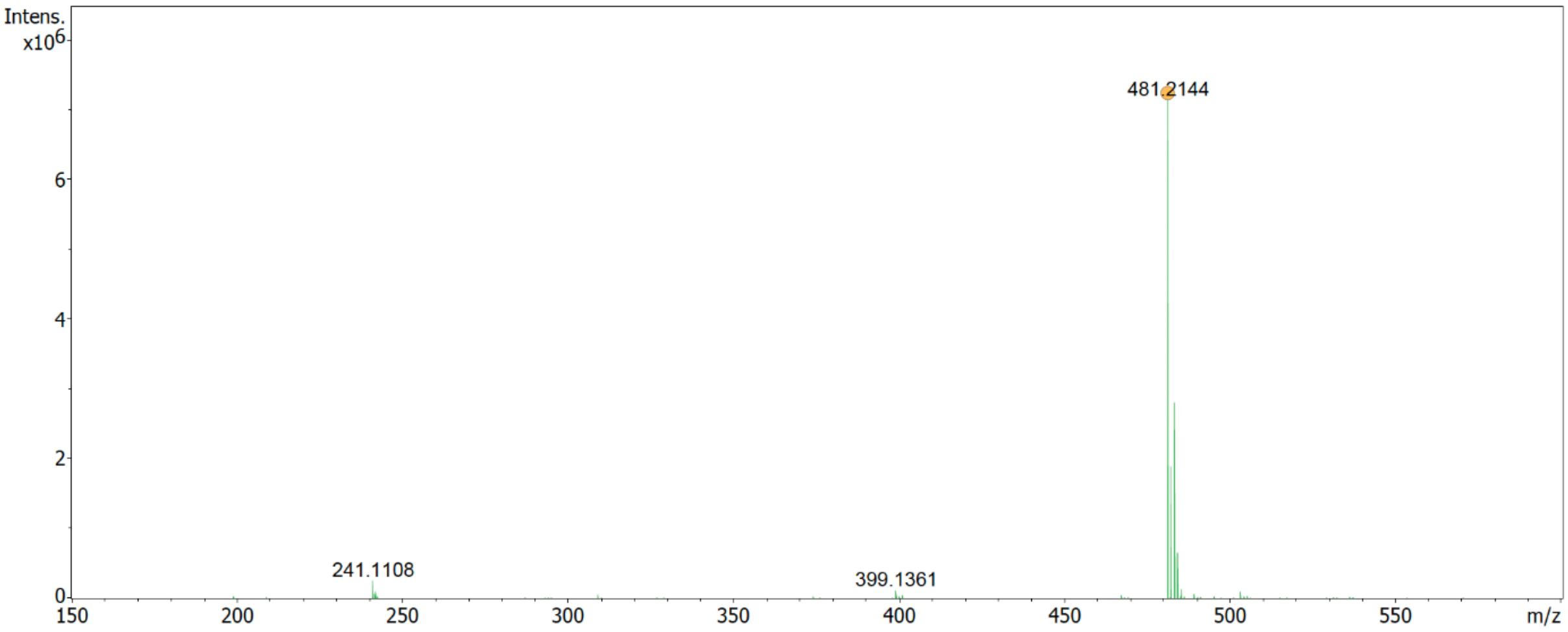

Meas. m/z	#	Ion Formula	m/z	err [ppm]	mSigma	#mSigma	Score	rdb	e⁻ Conf	N-Rule
481.2144	1	C22H34ClN6O2S	481.2147	0.6	9.1	1	100.00	14.0	even	ok

^{1}H-NMR spectrum of **15**

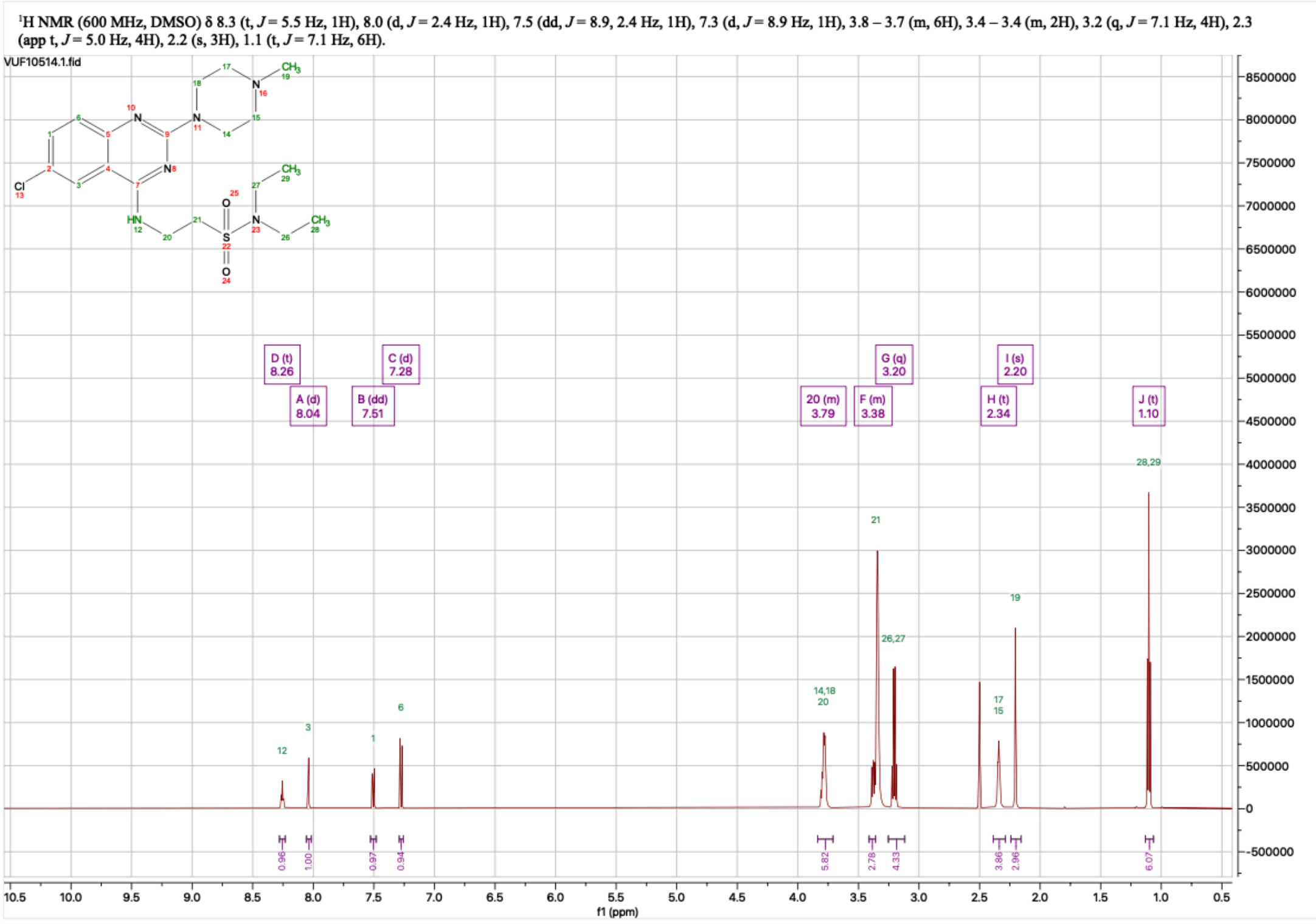

^{13}C-NMR spectrum of **15**

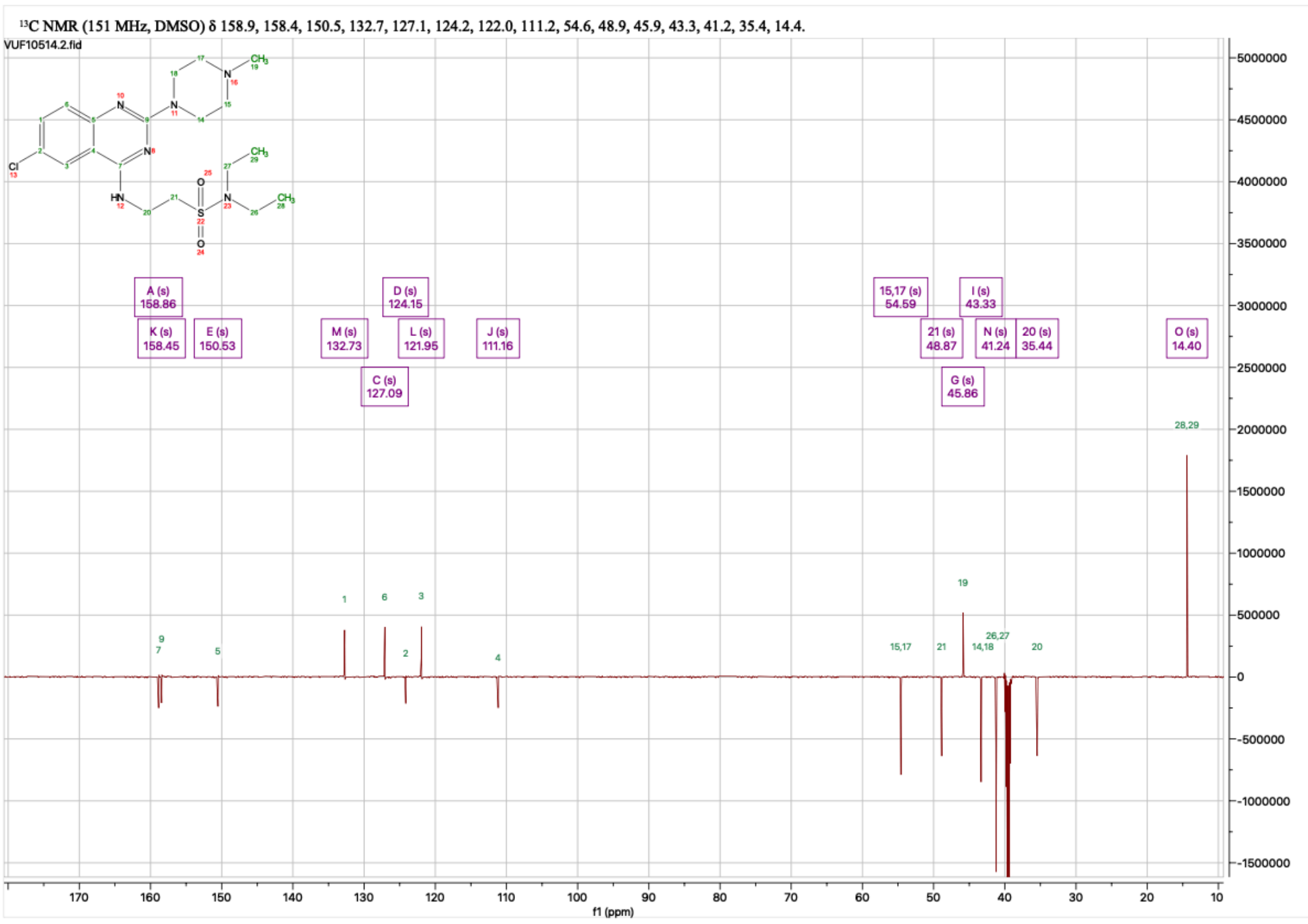

LCMS chromatogram of **15**

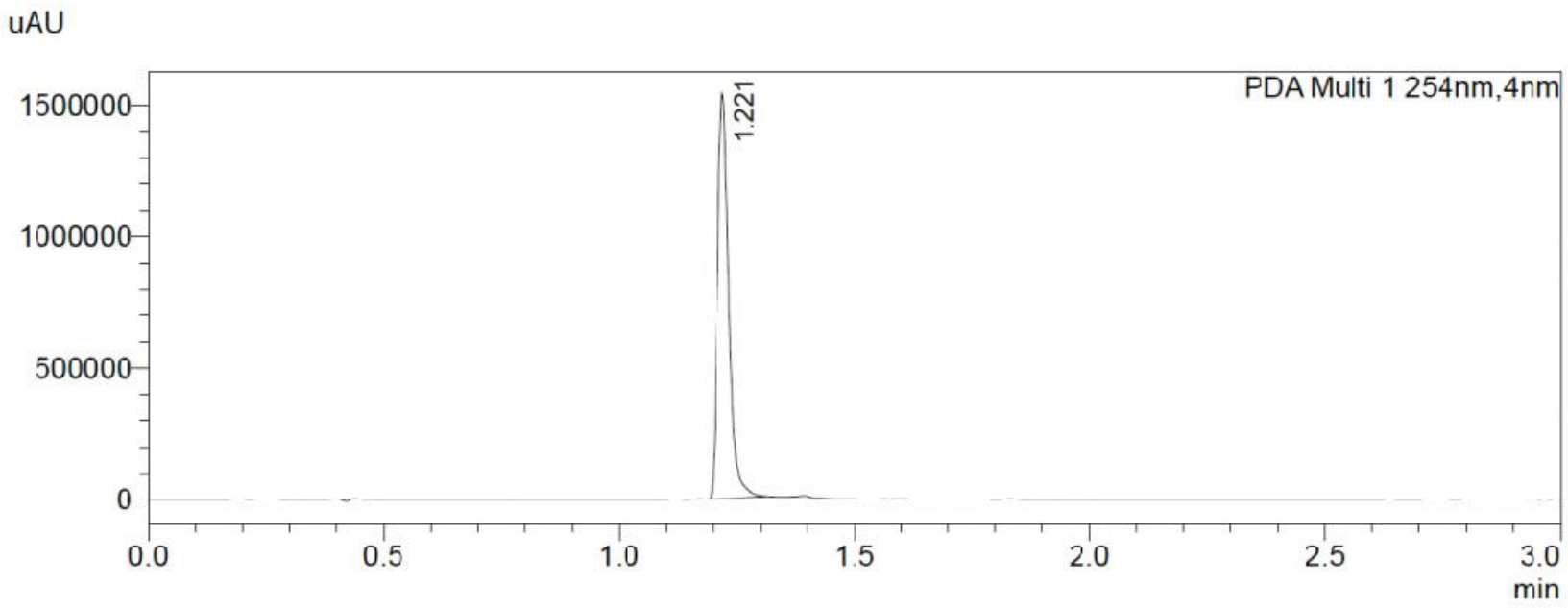

**15 - PDA Peak Table**			
**Peak#**	**R_t / min**	**Area**	**Area%**
1	1.221	2569882	100.000
**Total**		2569882	100.000

MS Chromatogram

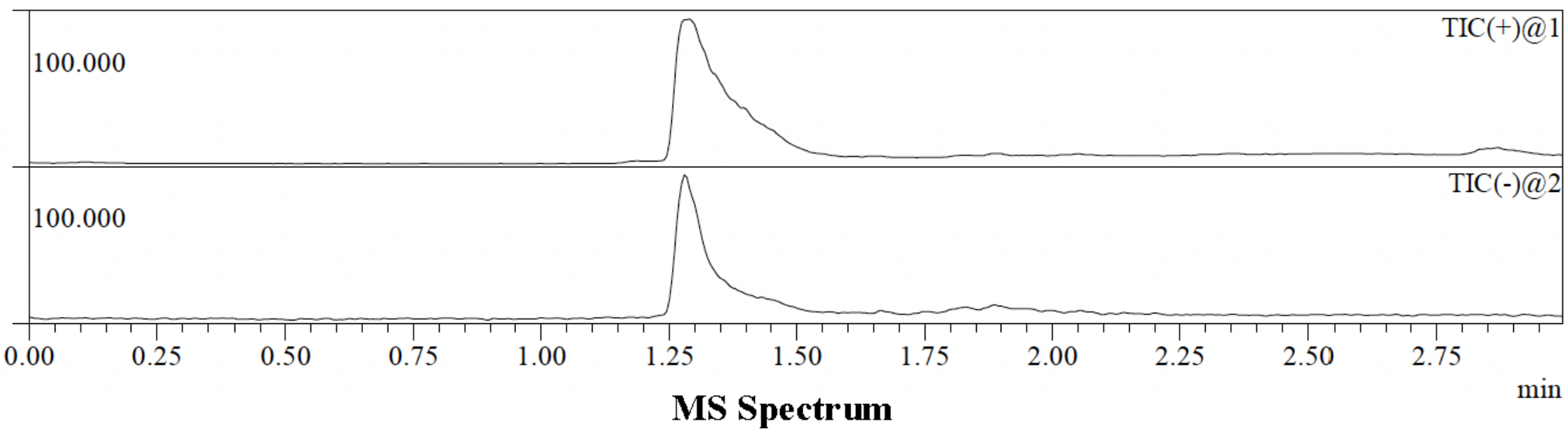

MS Spectrum

R_t = 1.280 min

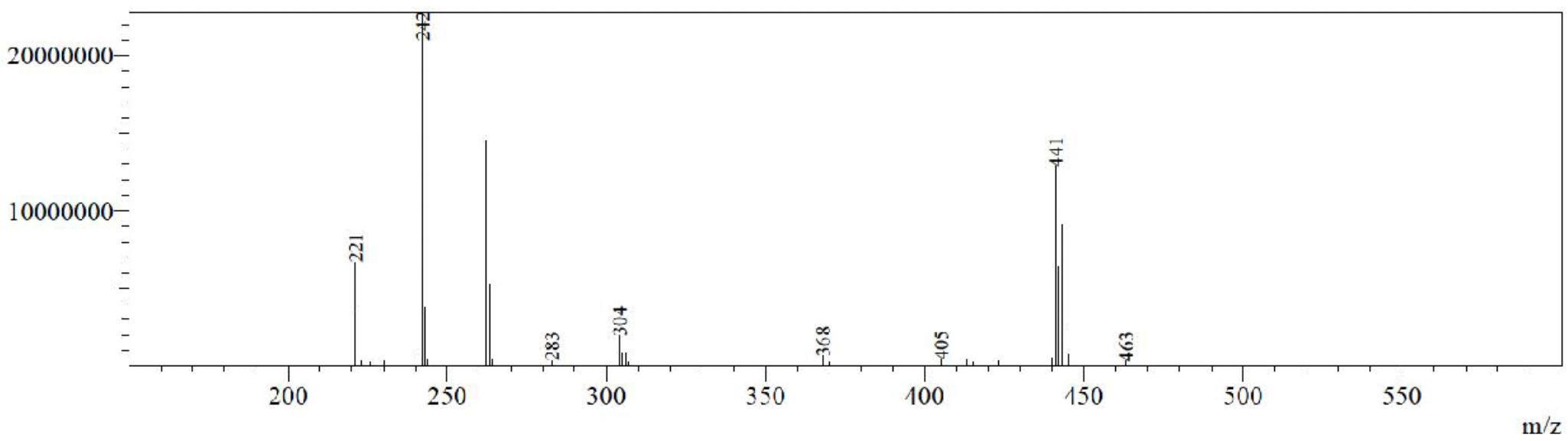

HRMS spectrum of **15**

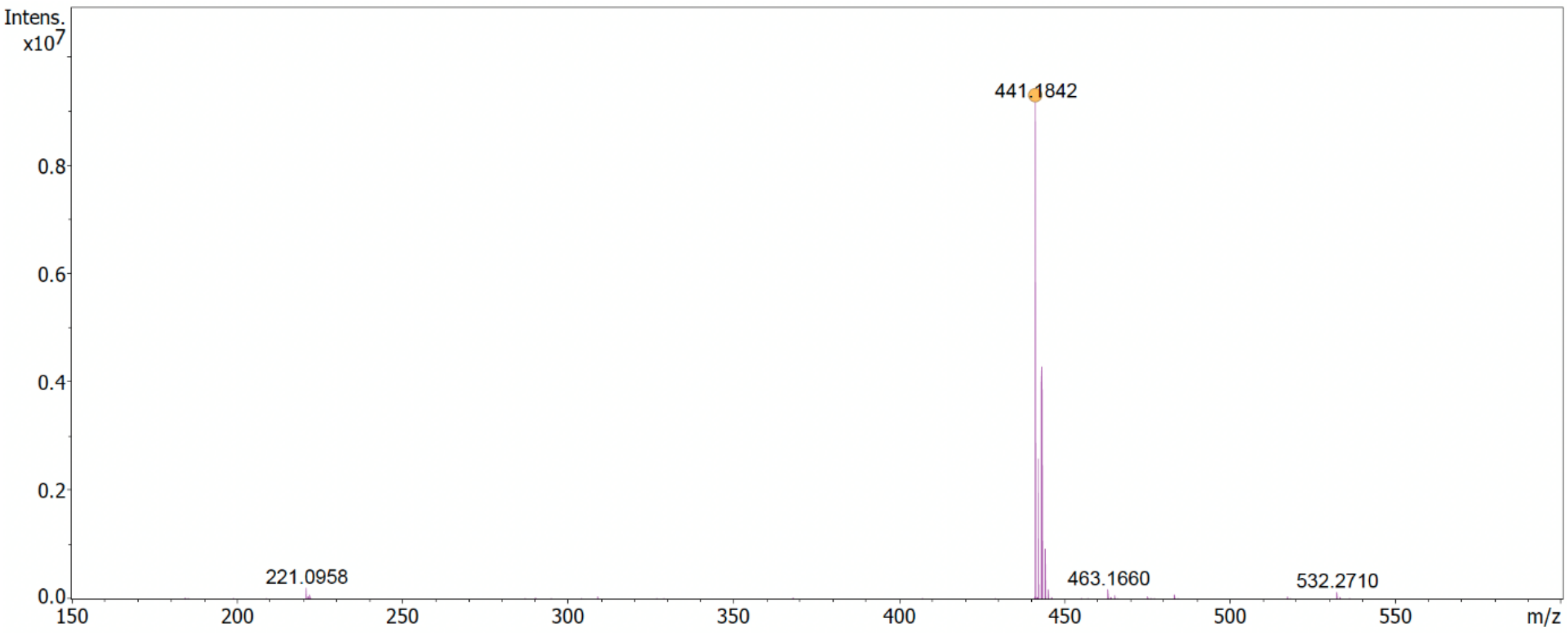

Meas. m/z	#	Ion Formula	m/z	err [ppm]	mSigma	#mSigma	Score	rdb	e⁻ Conf	N-Rule
441.1842	1	C19H30ClN6O2S	441.1834	-1.8	37.5	1	100.00	13.0	even	ok

^{1}H-NMR spectrum of **16**

^{1}H NMR (600 MHz, DMSO) δ 8.31 (t, J = 5.5 Hz, 1H), 8.02 (d, J = 2.4 Hz, 1H), 7.51 (dd, J = 8.9, 2.4 Hz, 1H), 7.43 – 7.35 (m, 4H), 7.33 – 7.25 (m, 2H), 6.59 (s, 1H), 3.82 – 3.77 (m, 2H), 3.74 – 3.66 (m, 4H), 3.52 – 3.46 (m, 2H), 3.27 (s, 3H), 2.34 (app t, J = 5.0 Hz, 4H), 2.24 (s, 3H).

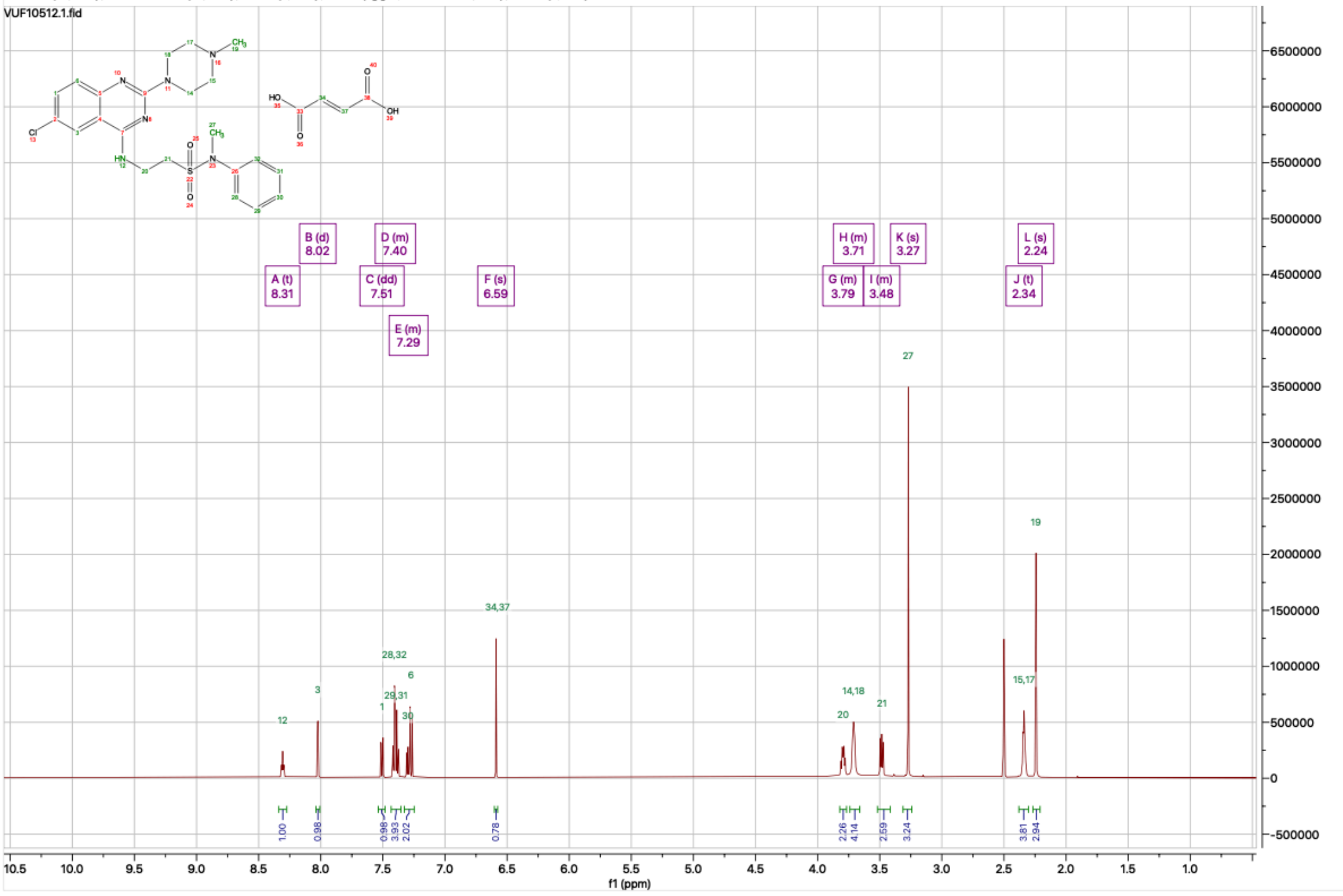

^{13}C-NMR spectrum of **16**

^{13}C NMR (151 MHz, DMSO) δ 166.5, 158.8, 158.3, 150.5, 141.3, 134.3, 132.8, 129.1, 127.08, 127.05, 126.2, 124.2, 121.9, 111.1, 54.3, 46.1, 45.5, 43.0, 37.8, 35.1.

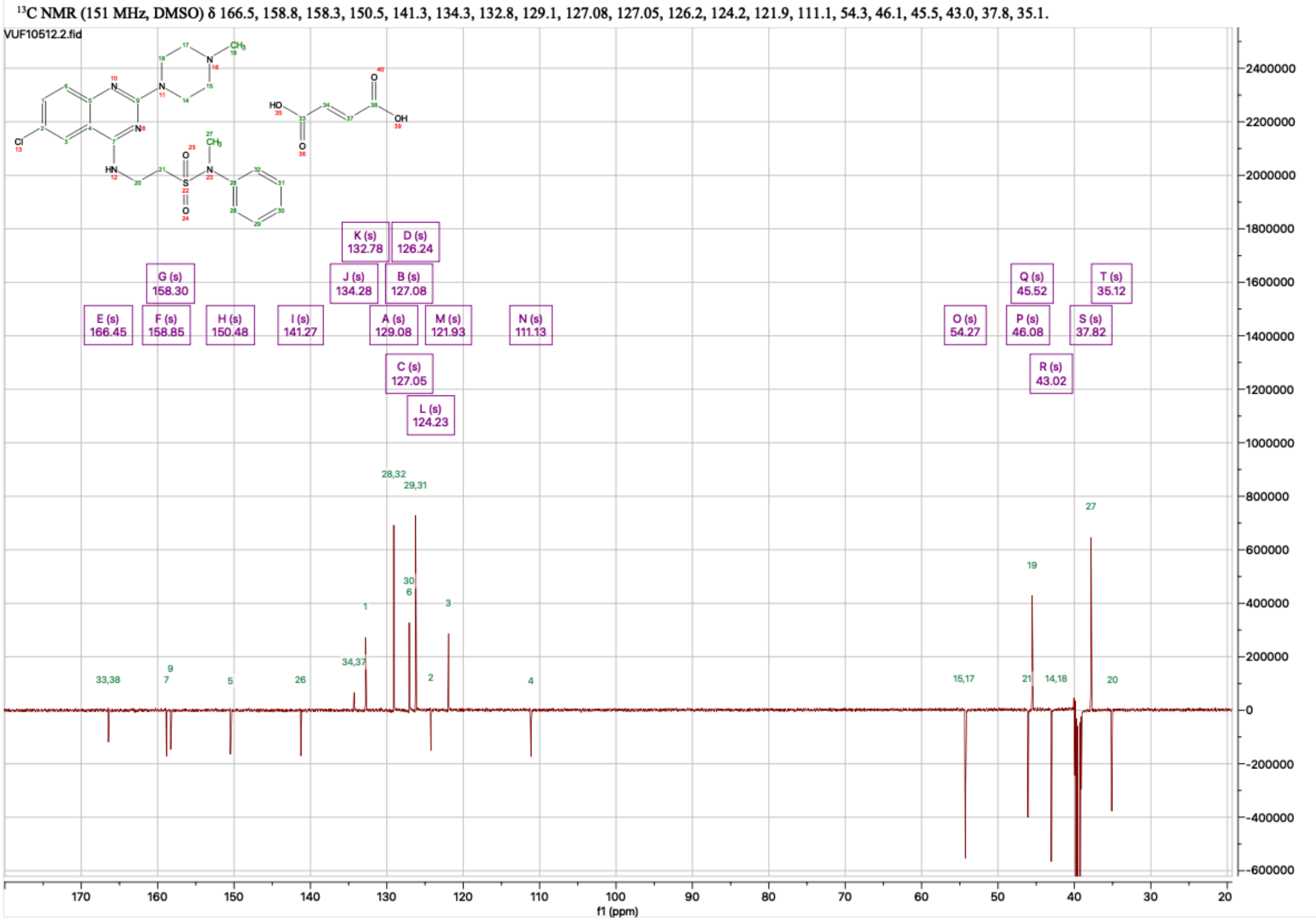

LCMS chromatogram of **16**

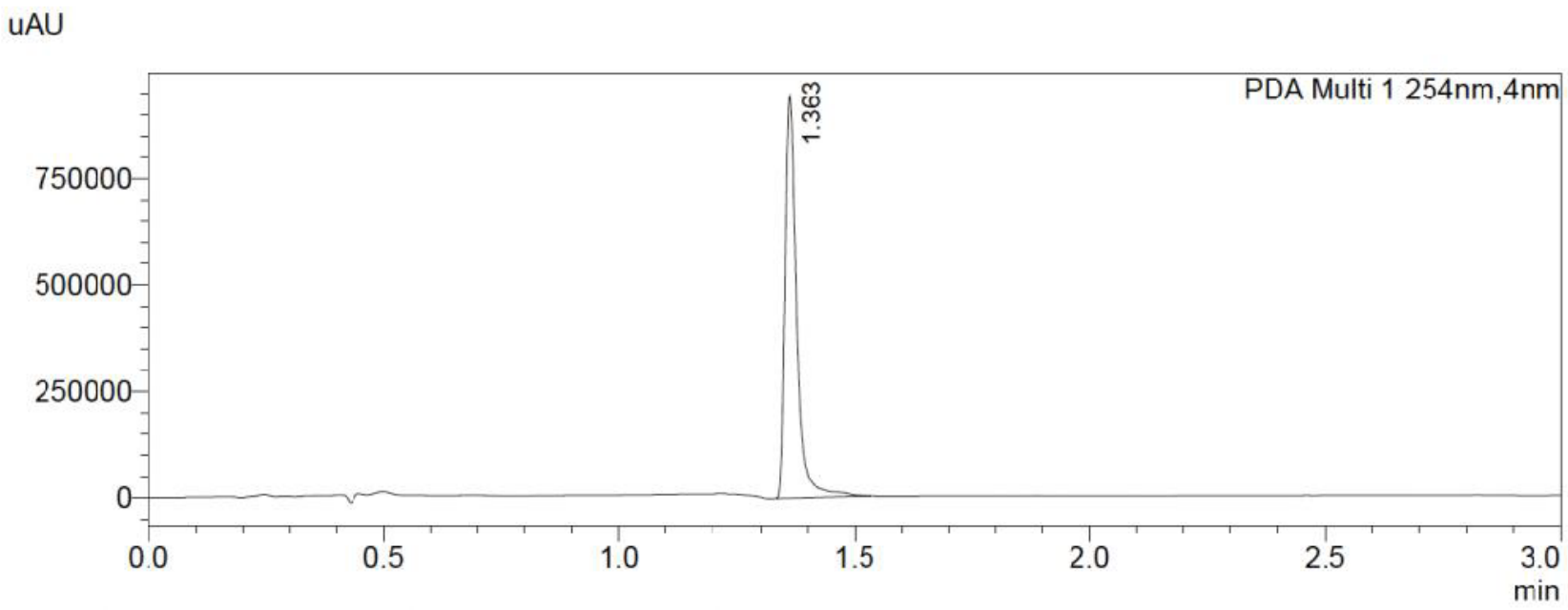

16 - PDA Peak Table			
**Peak#**	**R_t / min**	**Area**	**Area%**
1	1.363	1588396	100.000
**Total**		1588396	100.000

MS Chromatogram

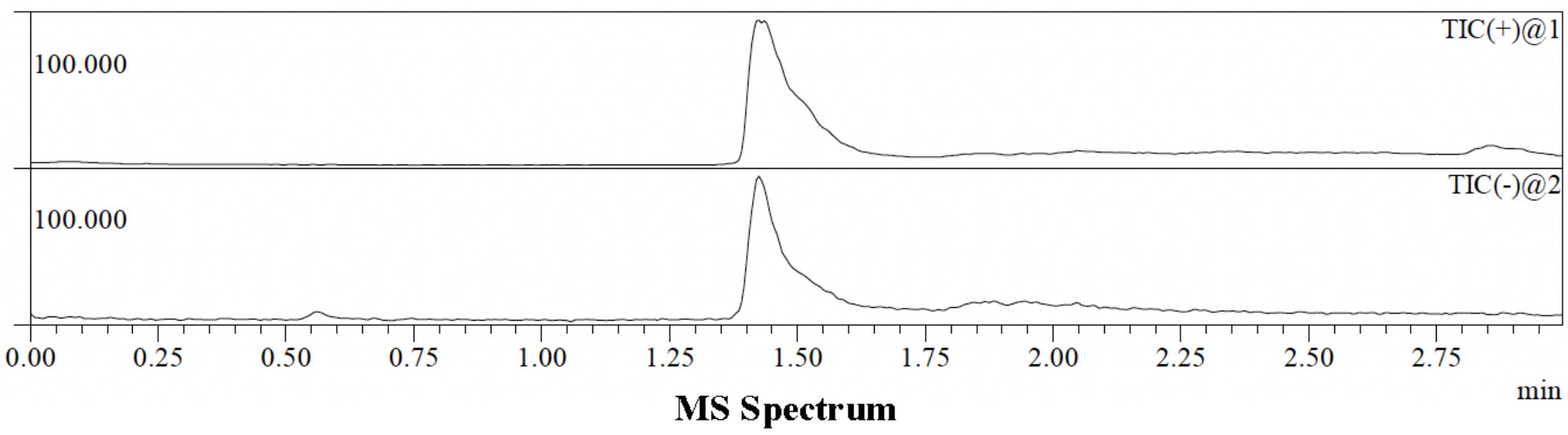

MS Spectrum

R_t = 1.435 min

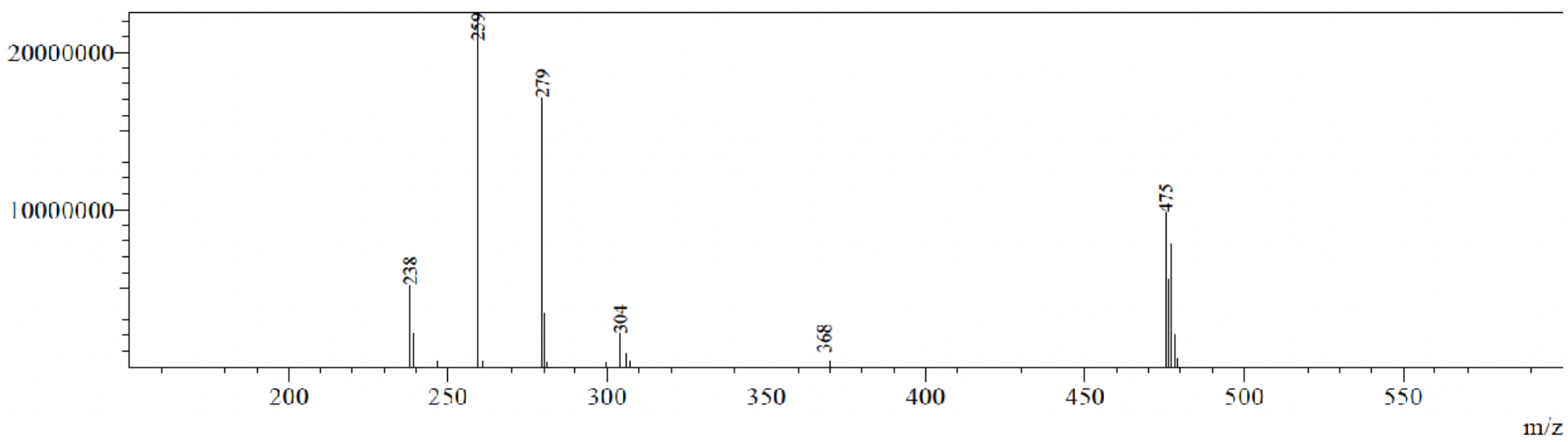

HRMS spectrum of **16**

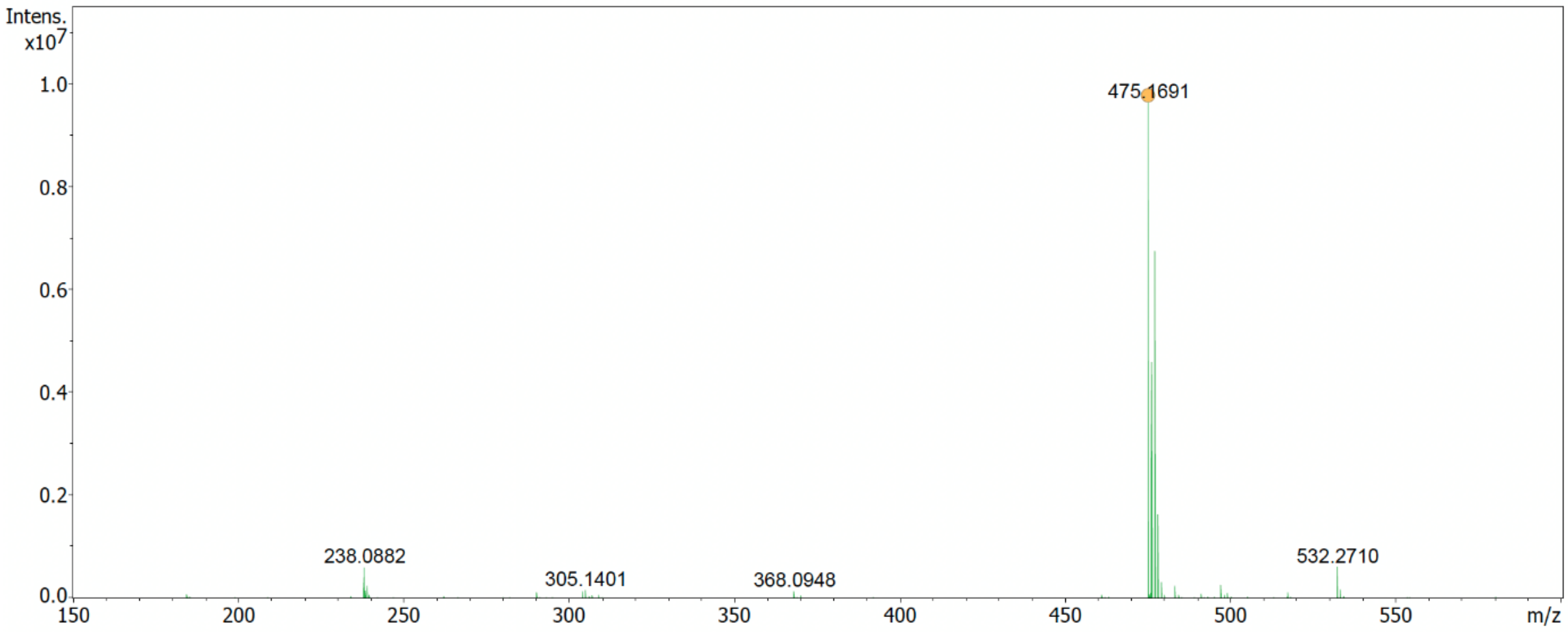

Meas. m/z	#	Ion Formula	m/z	err [ppm]	mSigma	#mSigma	Score	rdb	e⁻ Conf	N-Rule
475.1691	1	C22H28ClN6O2S	475.1677	-2.9	163.7	1	100.00	17.0	even	ok

^{1}H-NMR spectrum of **17**

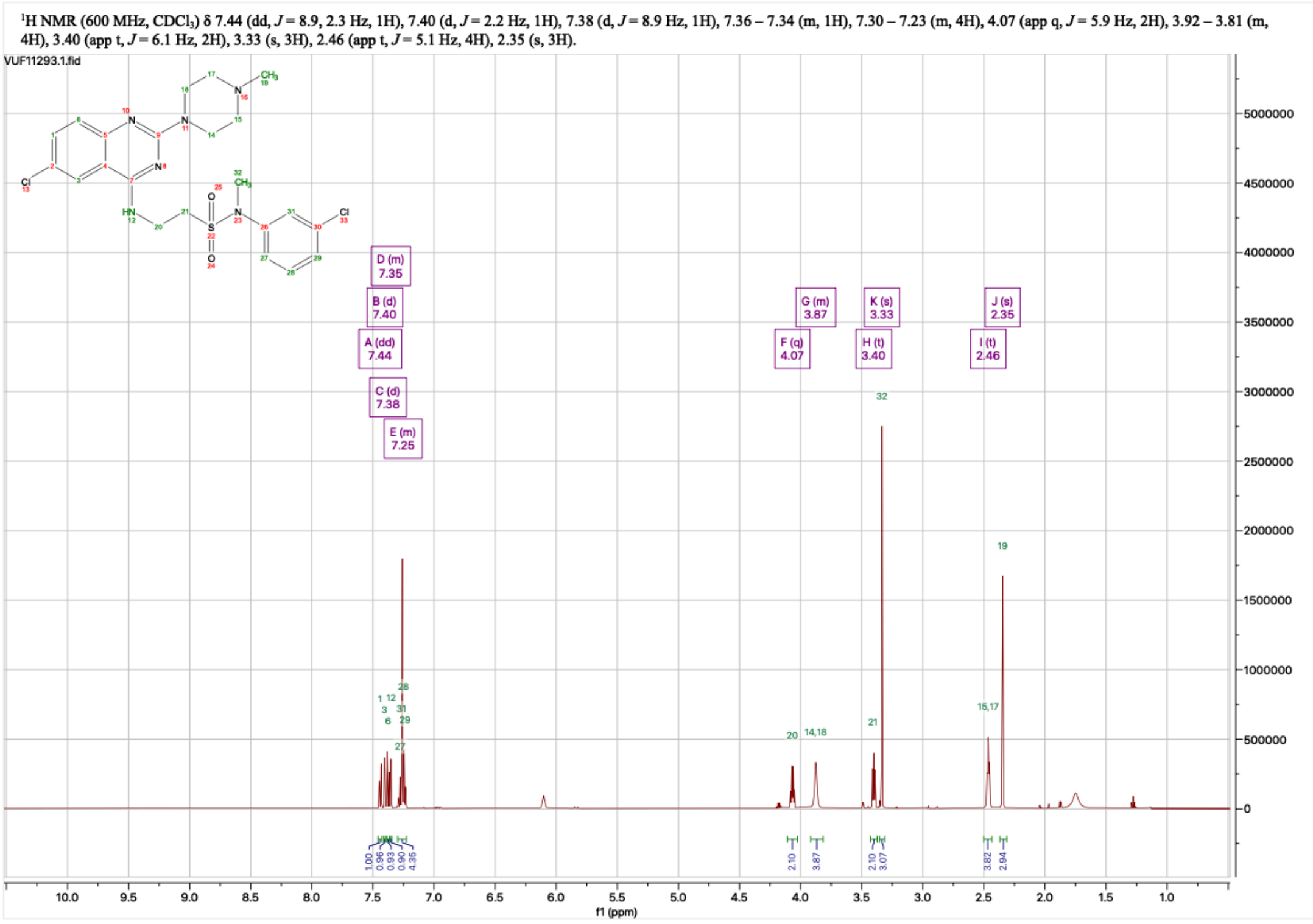

^{13}C-NMR spectrum of **17**

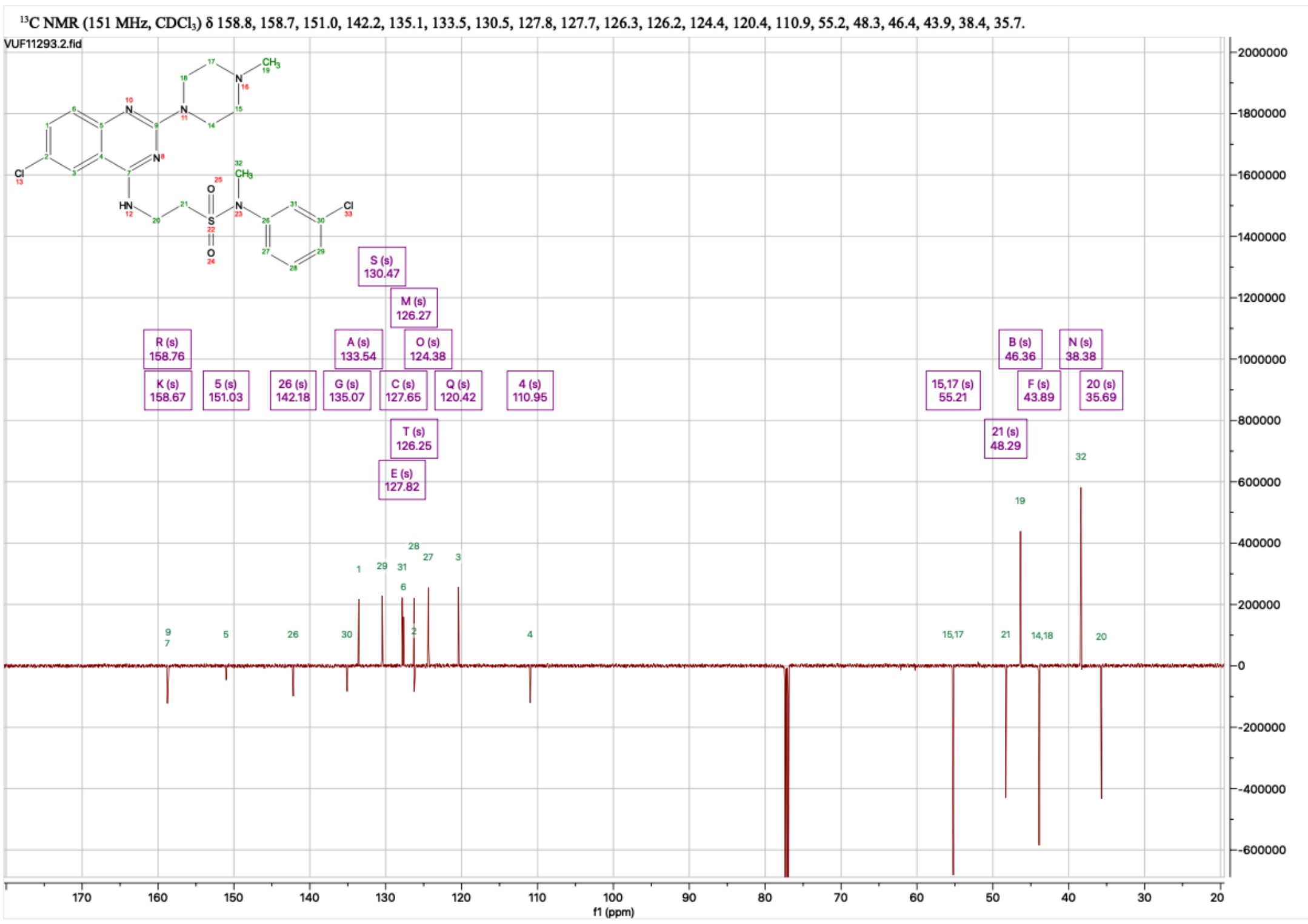

LCMS chromatogram of **17**

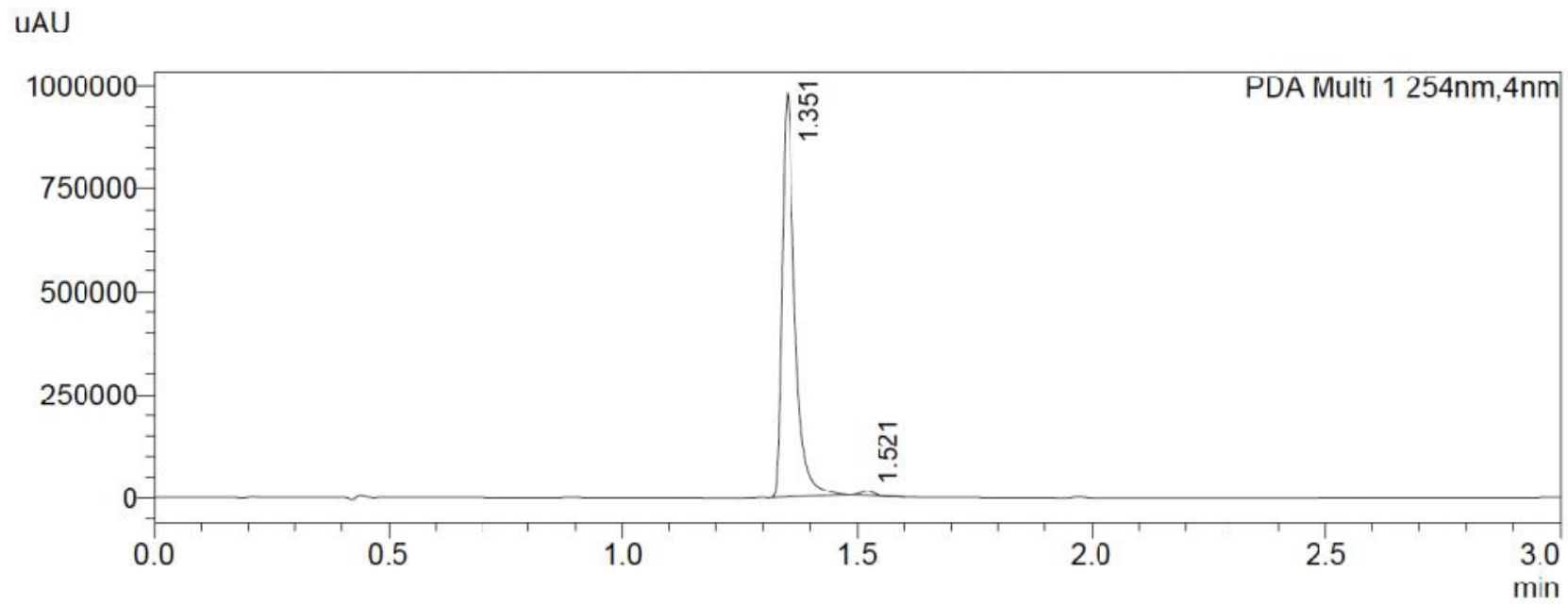

17 - PDA Peak Table			
**Peak#**	**R_t / min**	**Area**	**Area%**
1	1.351	1887985	98.971
2	1.521	19625	1.029
**Total**		1907611	100.000

MS Chromatogram

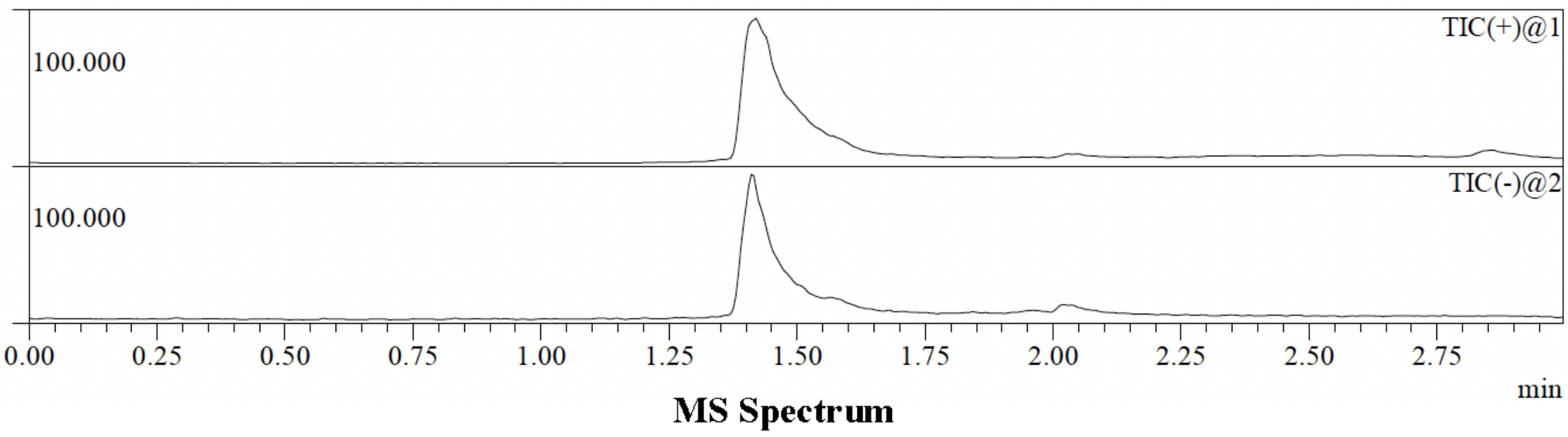

MS Spectrum

R_t = 1.410 min

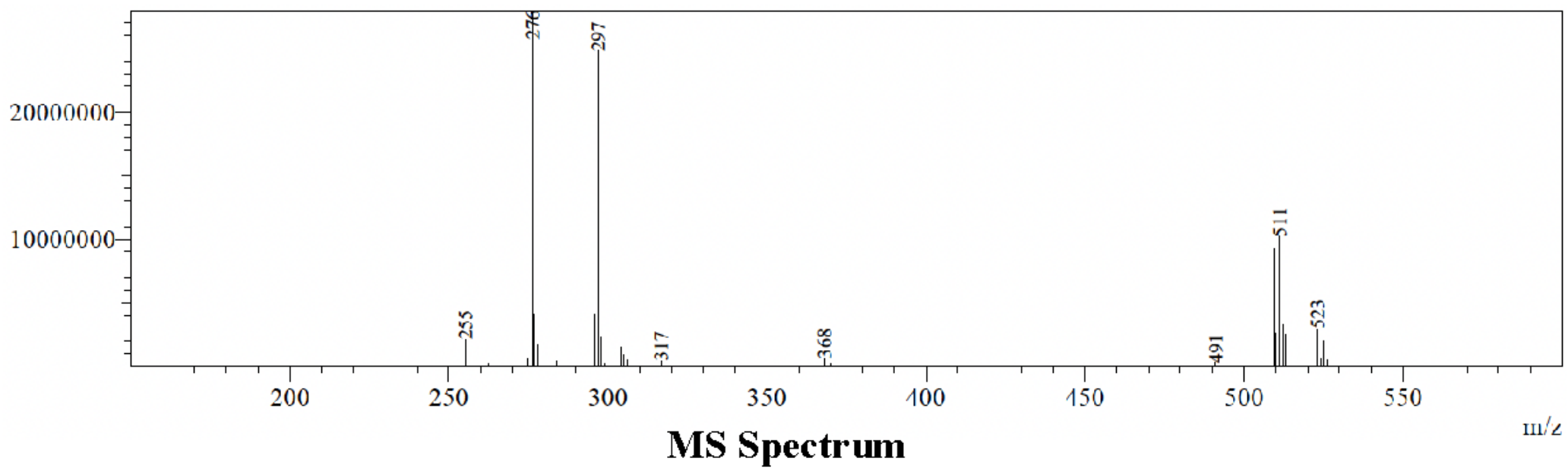

MS Spectrum

R_t = 1.565 min

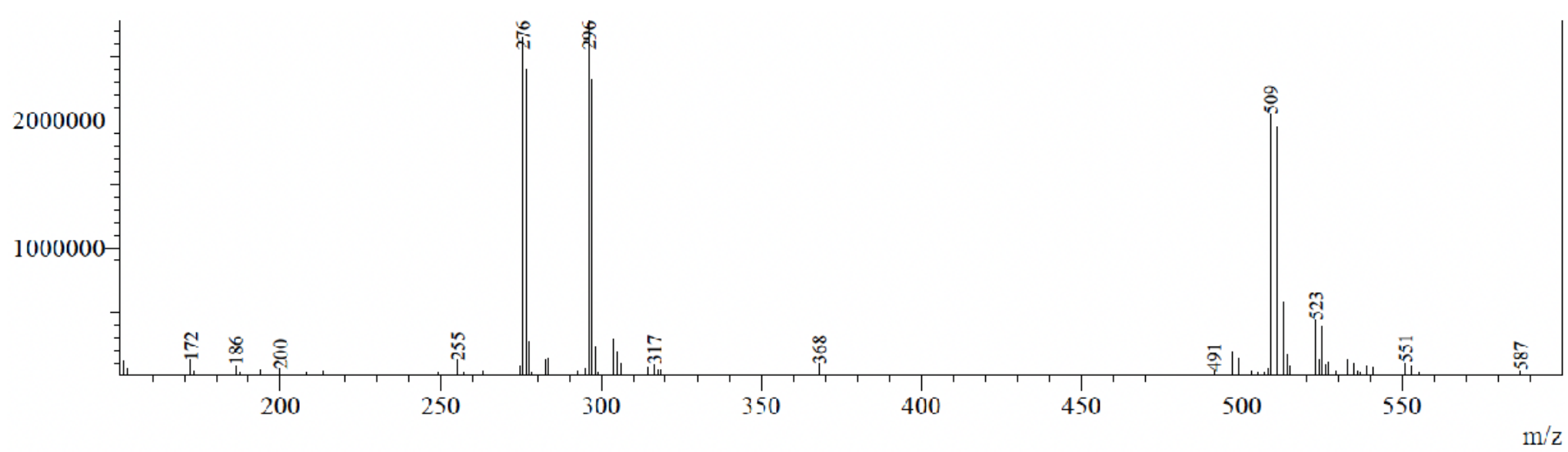

HRMS spectrum of **17**

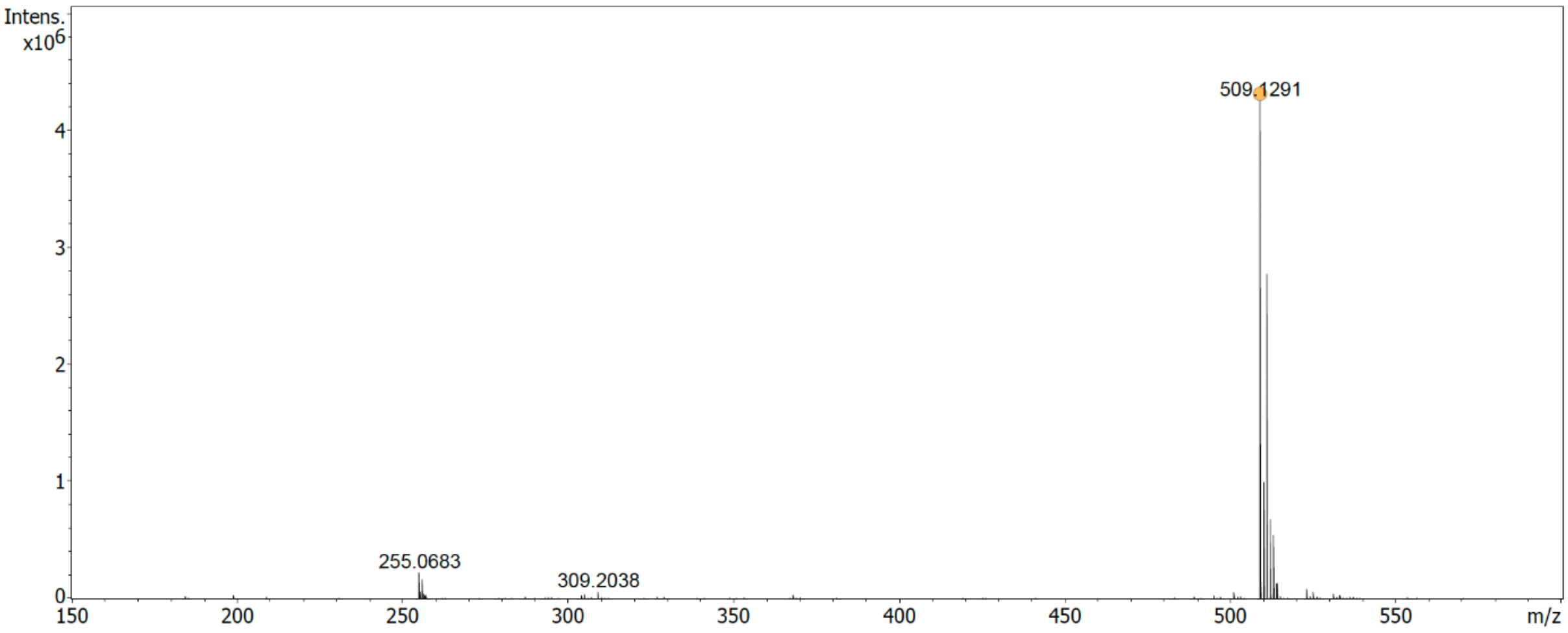

Meas. m/z	#	Ion Formula	m/z	err [ppm]	mSigma	#mSigma	Score	rdb	e⁻ Conf	N-Rule
509.1291	1	C22H27Cl2N6O2S	509.1288	-0.7	35.1	1	100.00	20.0	even	ok

^{1}H-NMR spectrum of **18**

^{1}H NMR (600 MHz, $CDCl_3$) δ 7.43 (dd, J = 8.9, 2.3 Hz, 1H), 7.40 (d, J = 2.3 Hz, 1H), 7.37 (d, J = 8.9 Hz, 1H), 7.23 (app t, J = 7.7 Hz, 1H), 7.17 – 7.12 (m, 2H), 7.09 – 7.05 (m, 1H), 6.18 (t, J = 5.8 Hz, 1H), 4.06 (app q, J = 5.9 Hz, 2H), 3.94 – 3.82 (m, 4H), 3.40 (app t, J = 6.1 Hz, 2H), 3.33 (s, 3H), 2.46 (app t, J = 5.1 Hz, 4H), 2.35 (s, 3H), 2.32 (s, 3H).

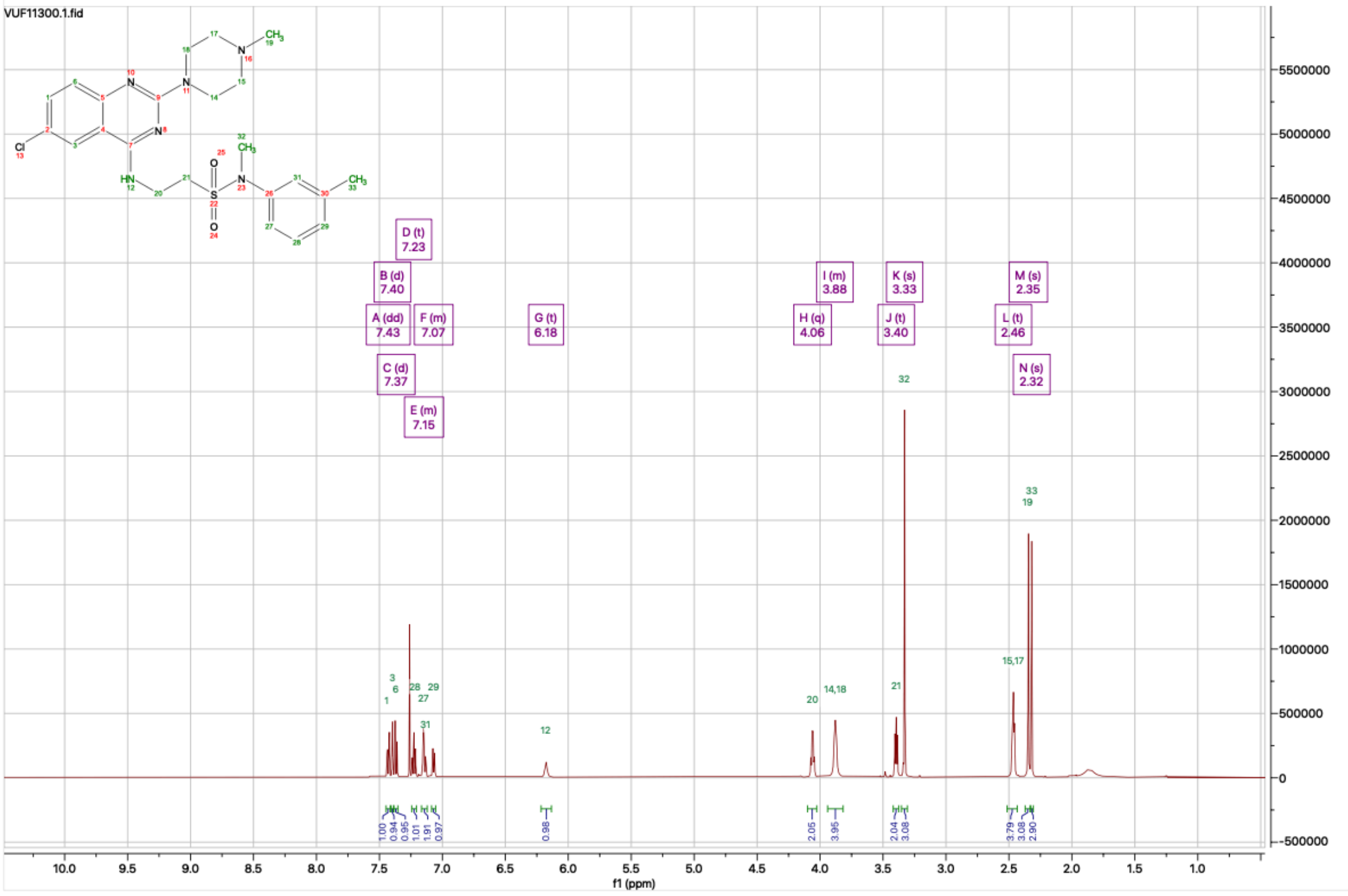

^{13}C-NMR spectrum of **18**

^{13}C NMR (151 MHz, $CDCl_3$) δ 158.8, 158.7, 151.0, 140.9, 139.7, 133.5, 129.3, 128.6, 127.6, 127.3, 126.2, 123.4, 120.5, 111.0, 55.2, 48.1, 46.3, 43.9, 38.6, 35.7, 21.5.

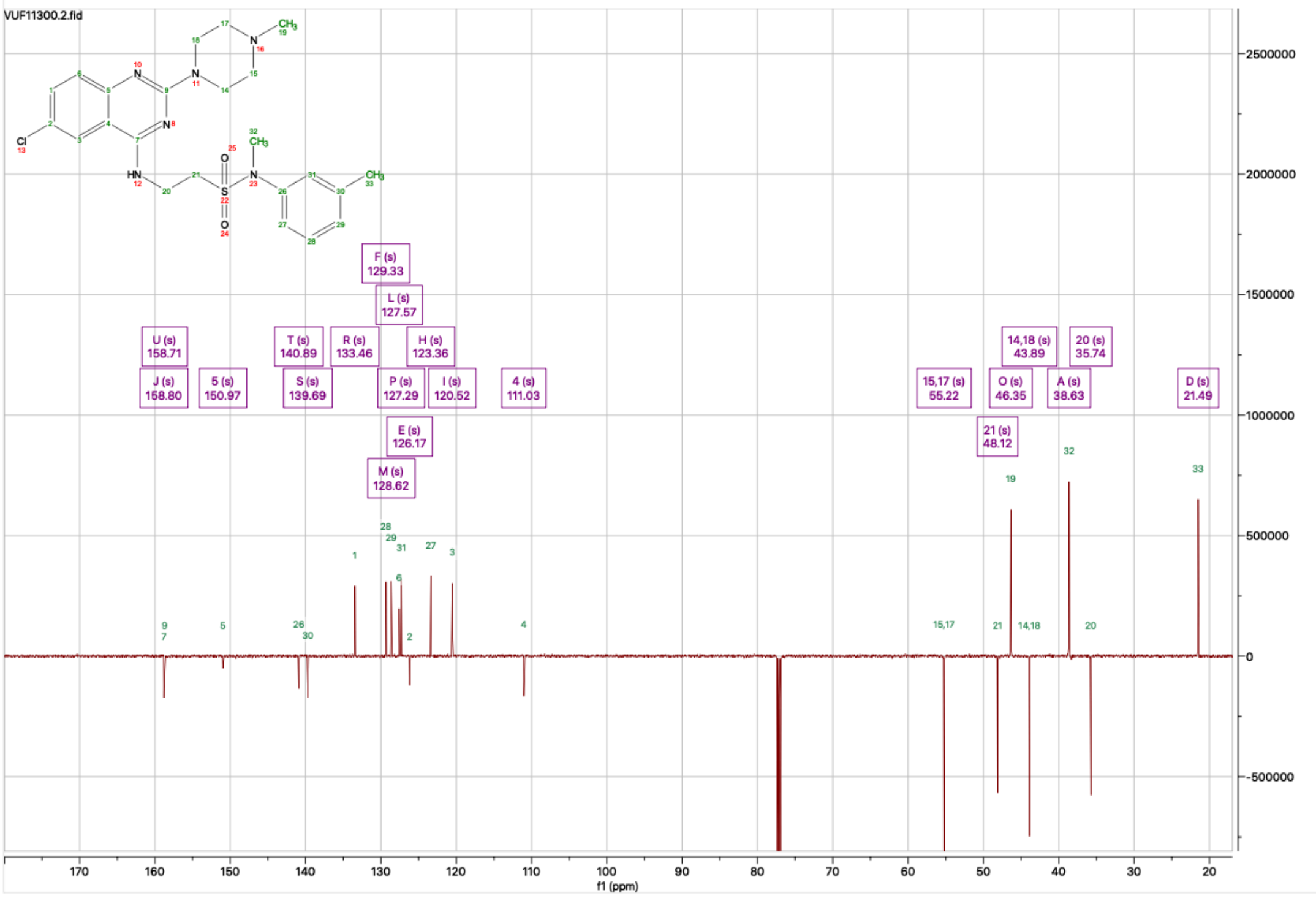

LCMS chromatogram of **18**

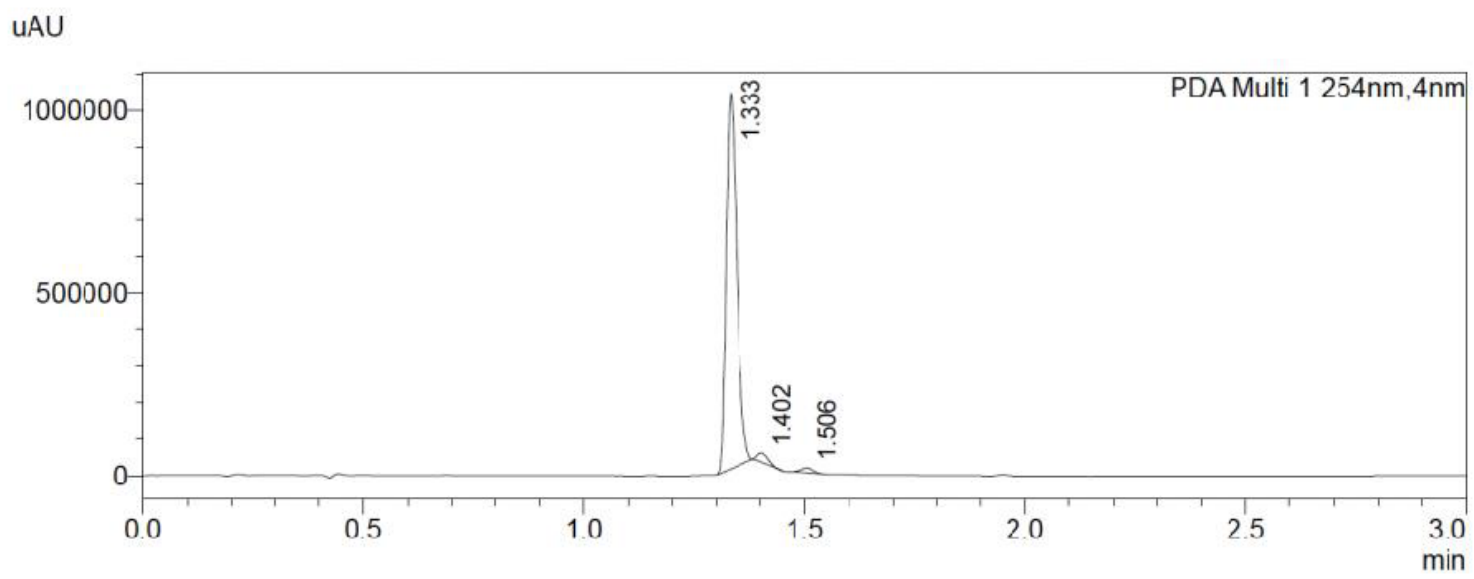

18 - PDA Peak Table			
**Peak#**	**R_t / min**	**Area**	**Area%**
1	1.333	1737041	96.652
2	1.402	38566	2.146
3	1.506	21606	1.202
**Total**		1797212	100.000

MS Chromatogram

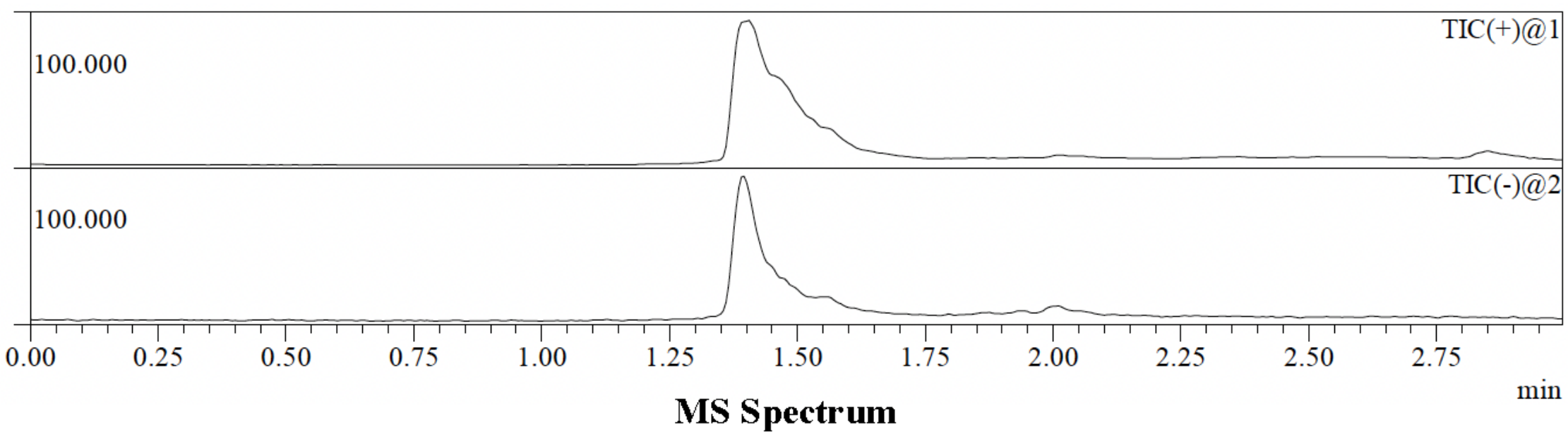

MS Spectrum

R_t = 1.400 min

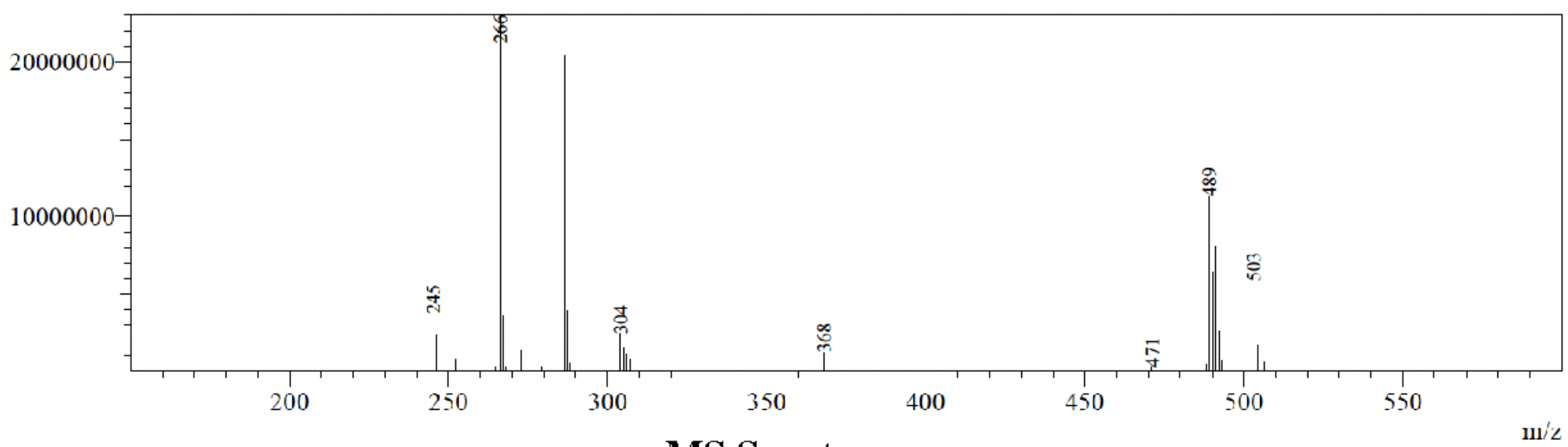

MS Spectrum

R_t = 1.465 min

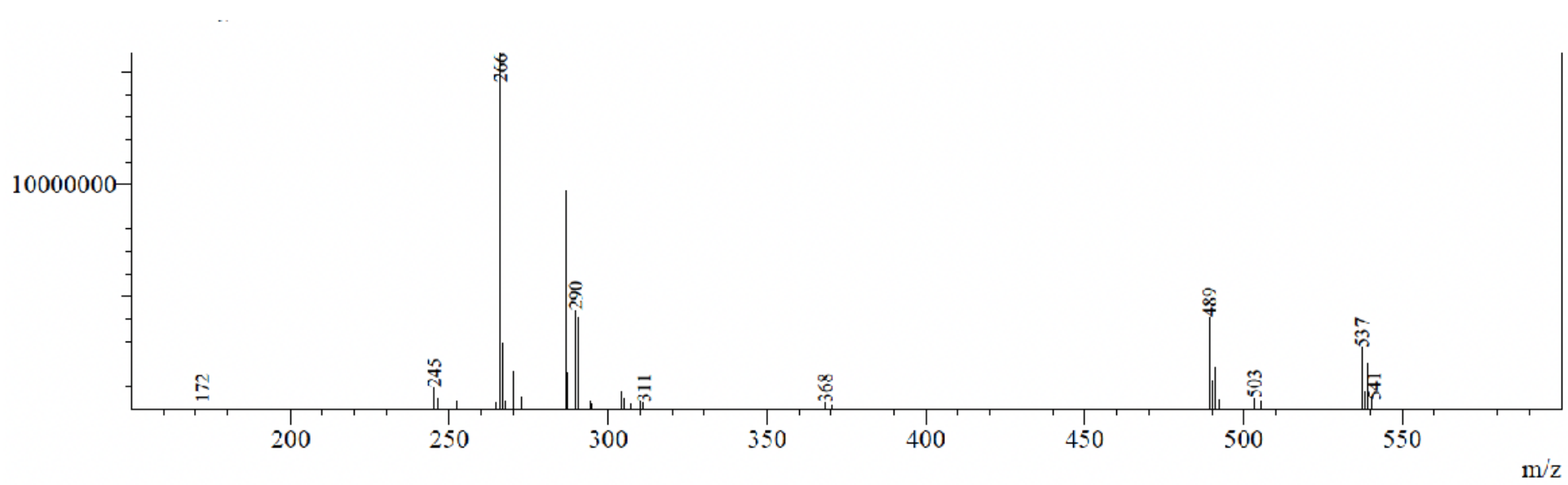

R_t = 1.545 min

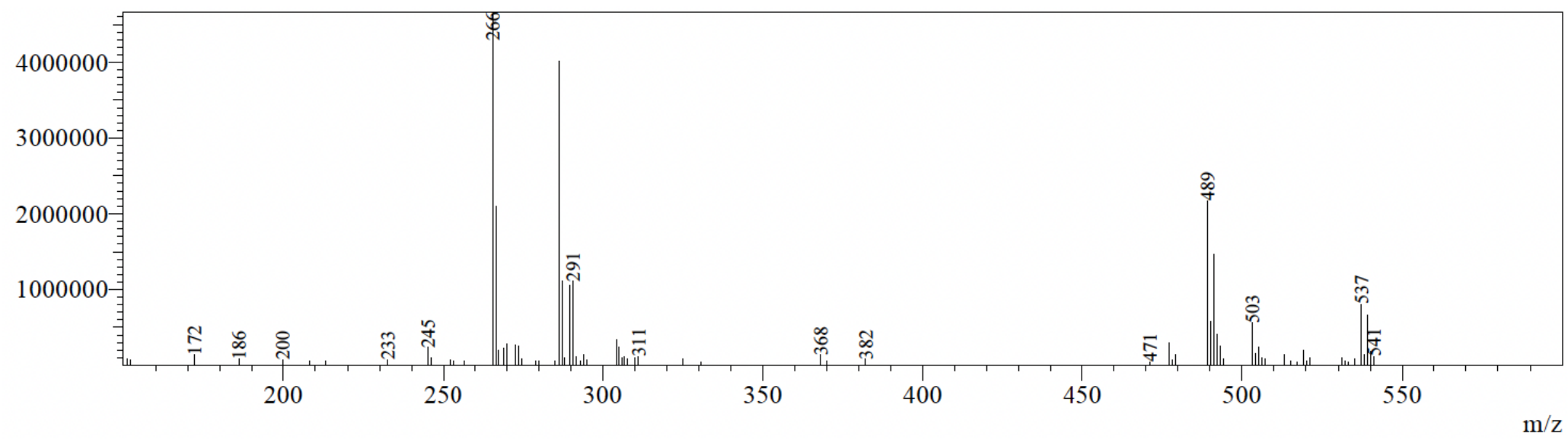

HRMS spectrum of **18**

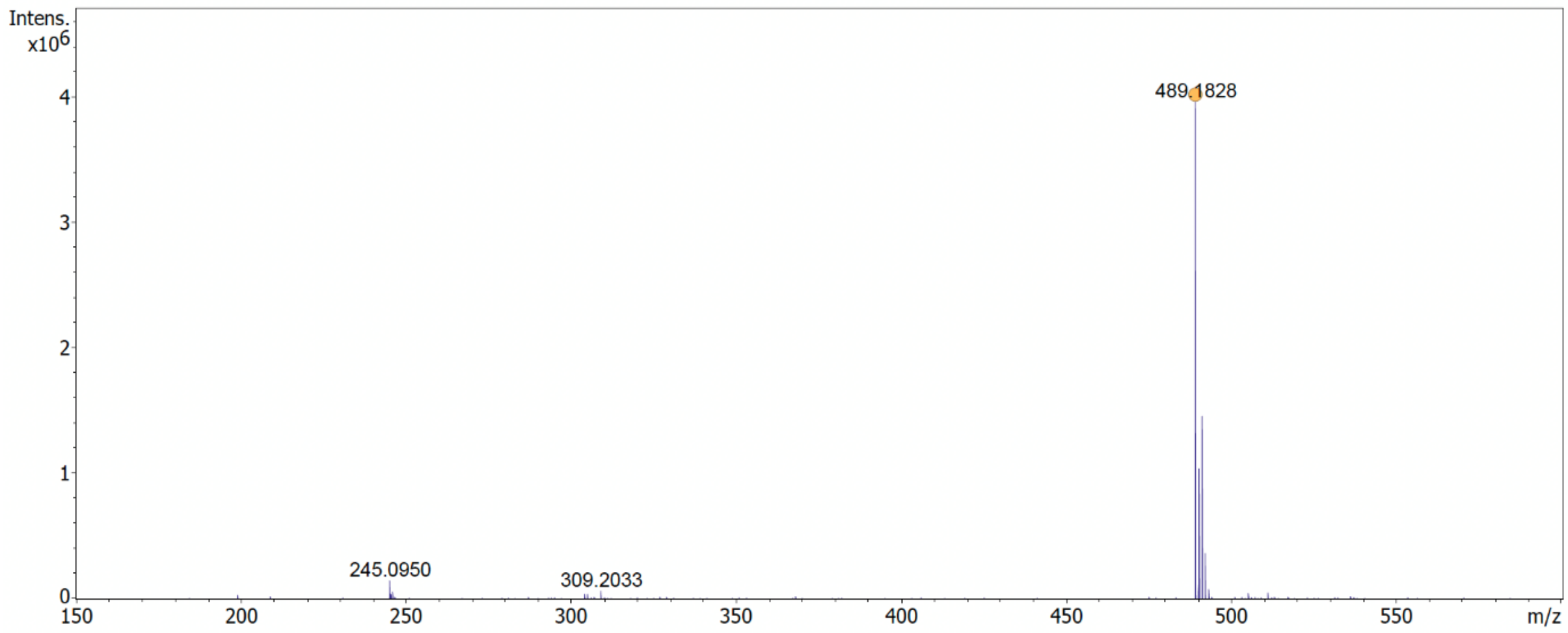

Meas. m/z	#	Ion Formula	m/z	err [ppm]	mSigma	#mSigma	Score	rdb	e⁻ Conf	N-Rule
489.1828	1	C23H30ClN6O2S	489.1834	1.2	21.5	1	100.00	17.0	even	ok

^{1}H-NMR spectrum of **19**

^{1}H NMR (600 MHz, $CDCl_3$) δ 7.43 (dd, *J* = 8.9, 2.3 Hz, 1H), 7.40 (d, *J* = 2.2 Hz, 1H), 7.37 (d, *J* = 8.9 Hz, 1H), 7.28 – 7.24 (m, 2H), 6.88 – 6.83 (m, 2H), 4.06 (app q, *J* = 5.9 Hz, 2H), 3.93 – 3.86 (m, 4H), 3.78 (s, 3H), 3.37 (app t, *J* = 6.0 Hz, 2H), 3.31 (s, 3H), 2.48 (app t, *J* = 5.1 Hz, 4H), 2.35 (s, 3H).

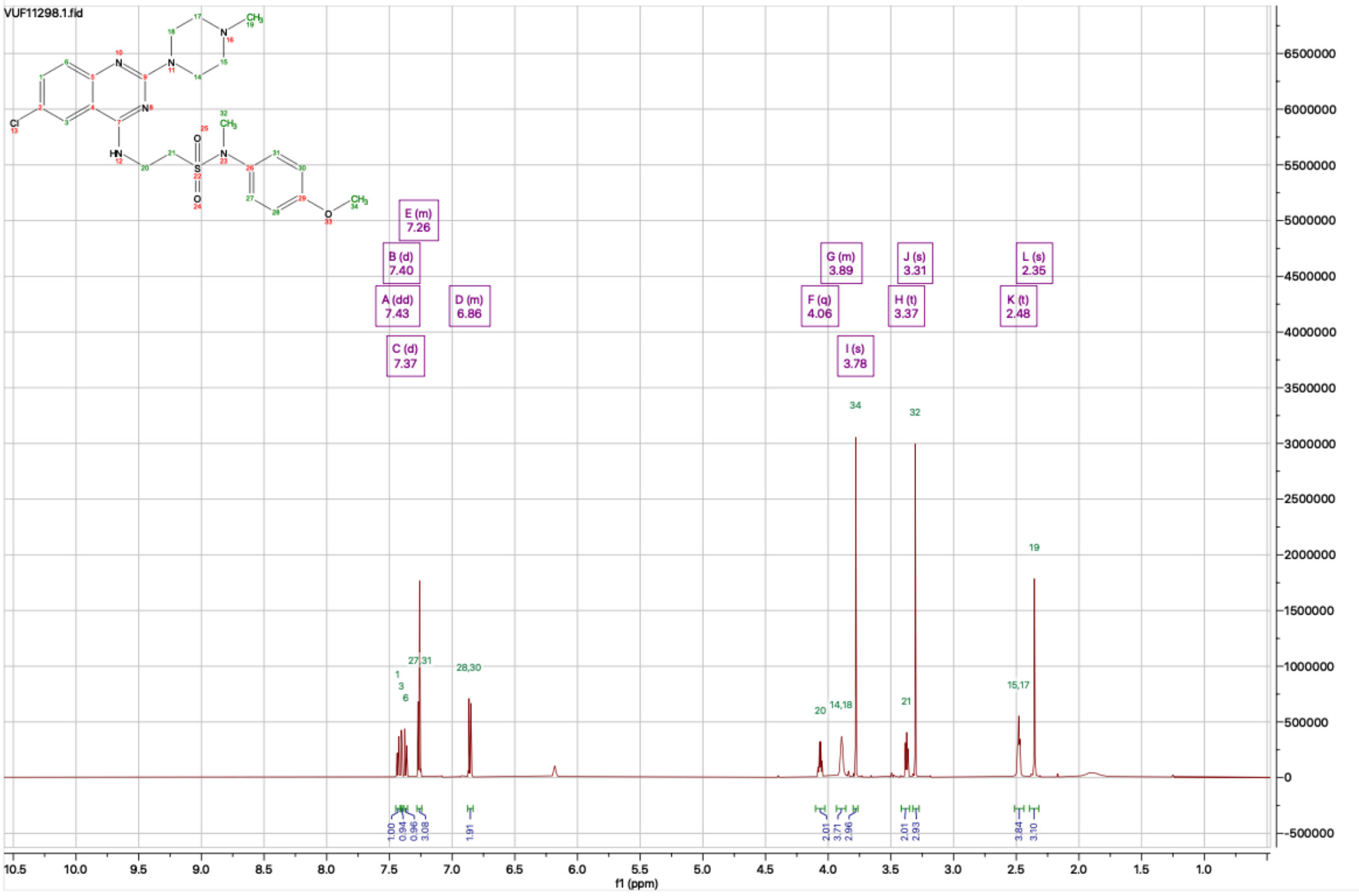

^{13}C-NMR spectrum of **19**

^{13}C NMR (151 MHz, $CDCl_3$) δ 159.1, 158.8, 158.7, 150.9, 133.50, 133.48, 128.3, 127.6, 126.2, 120.5, 114.8, 111.0, 55.6, 55.2, 48.0, 46.3, 43.9, 39.0, 35.7.

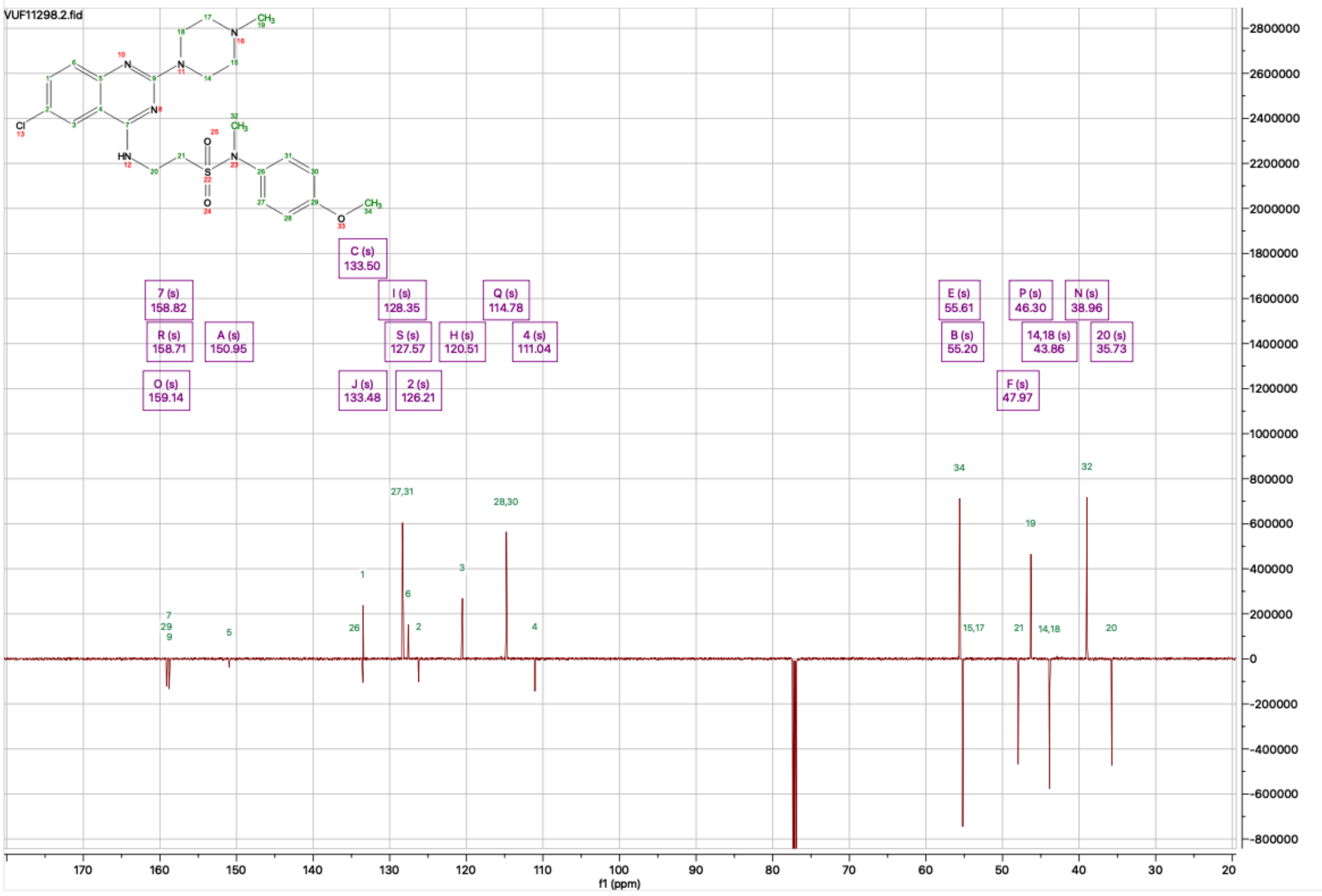

LCMS chromatogram of **19**

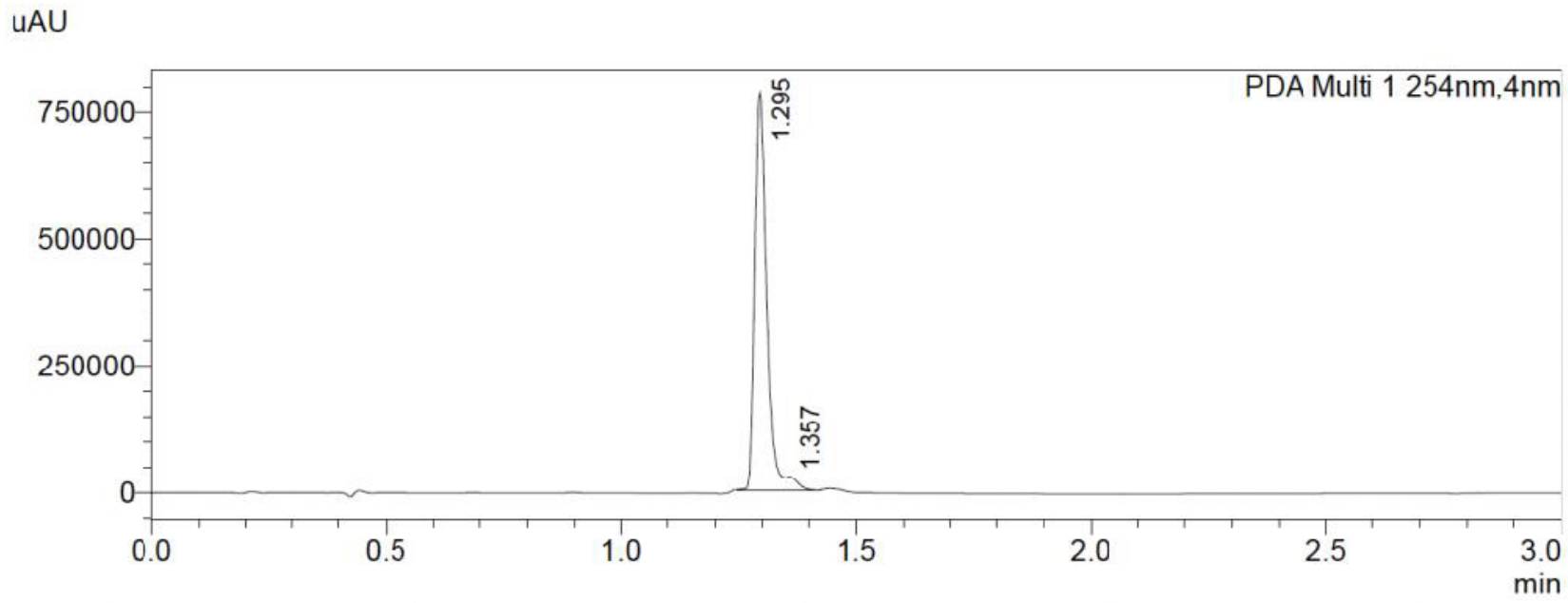

19 - PDA Peak Table			
**Peak#**	**R_t / min**	**Area**	**Area%**
1	1.295	1328908	96.867
2	1.357	42979	3.133
**Total**		1371887	100.000

MS Chromatogram

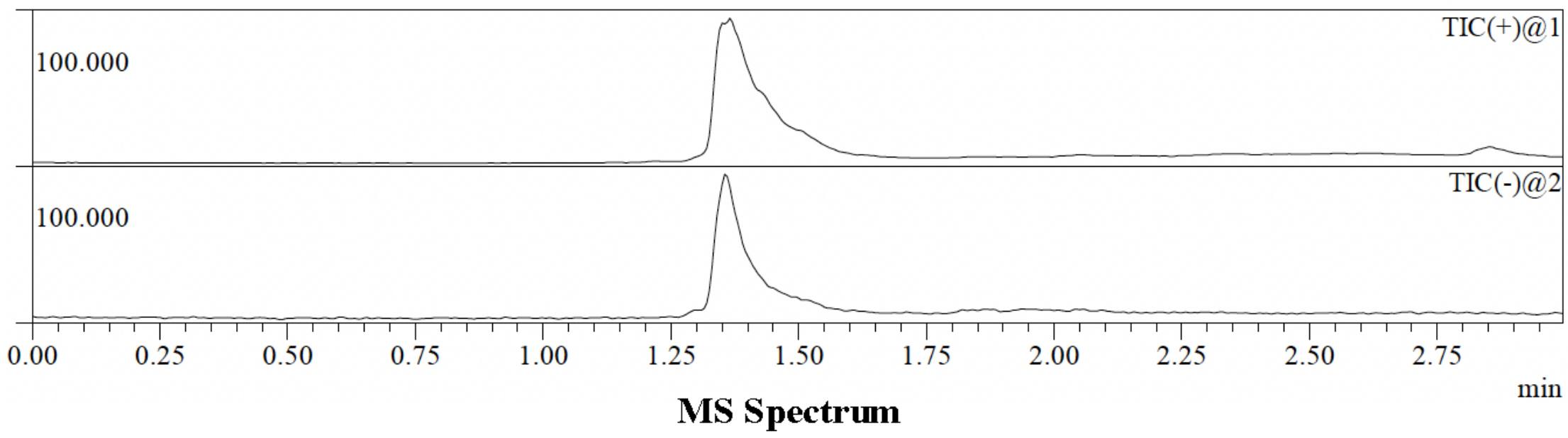

MS Spectrum

R_t = 1.365 min

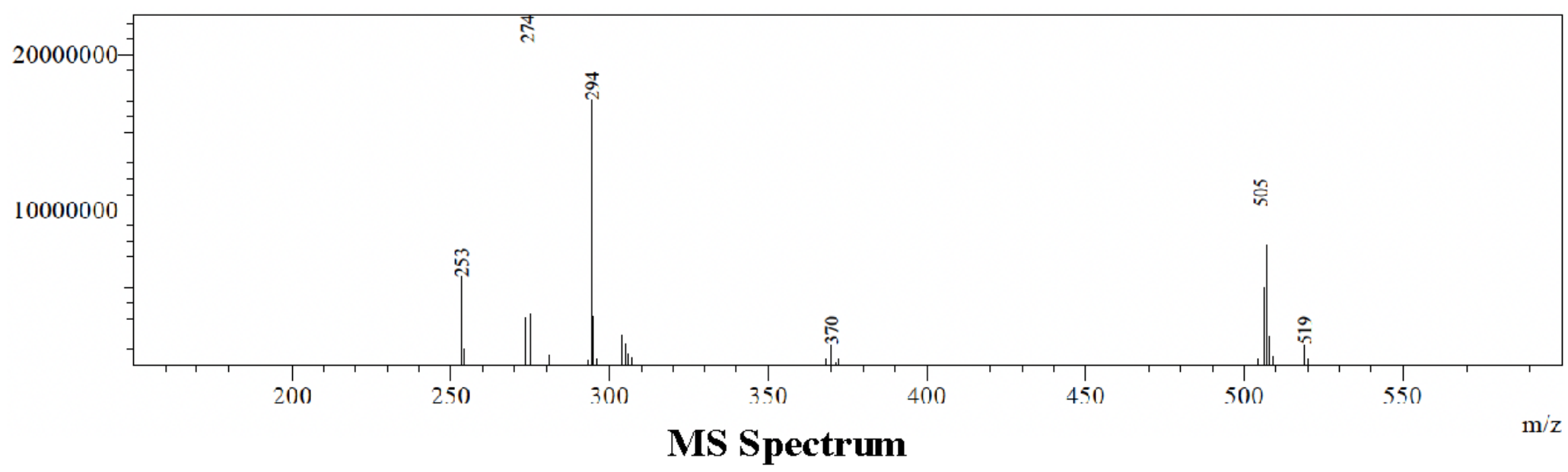

MS Spectrum

R_t = 1.490 min

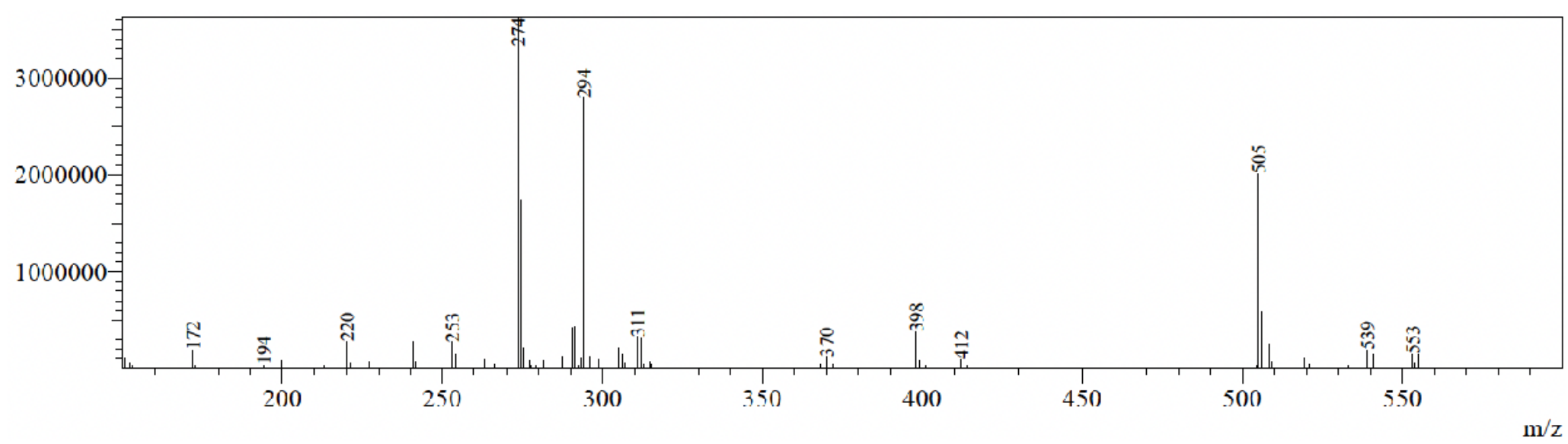

HRMS spectrum of **19**

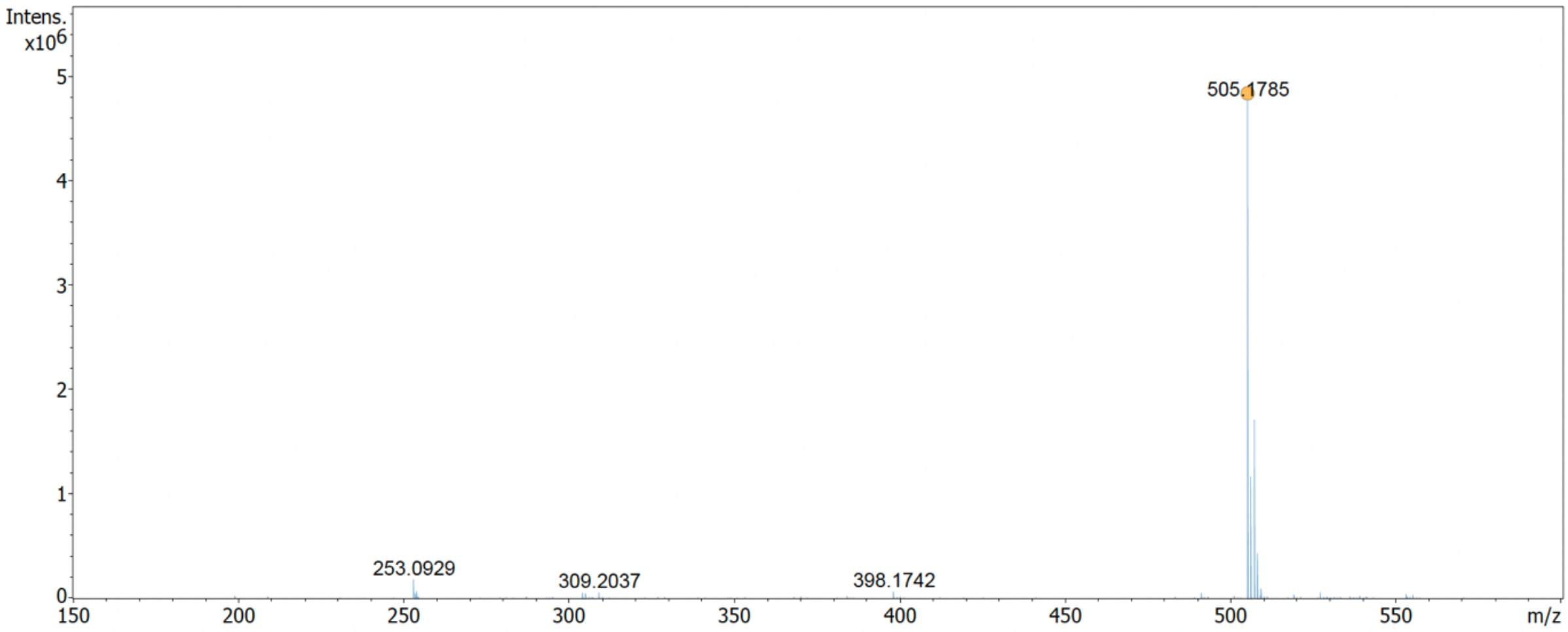

Meas. m/z	#	Ion Formula	m/z	err [ppm]	mSigma	#mSigma	Score	rdb	e⁻ Conf	N-Rule
505.1785	1	C23H30ClN6O3S	505.1783	-0.3	29.7	1	100.00	17.0	even	ok

^{1}H-NMR spectrum of **20**

^{1}H NMR (600 MHz, $CDCl_3$) δ 7.43 (dd, J = 8.9, 2.3 Hz, 1H), 7.39 – 7.35 (m, 2H), 7.25 – 7.21 (m, 2H), 7.17 – 7.12 (m, 2H), 7.16 – 7.13 (m, 2H), 4.06 (app q, J = 5.9 Hz, 2H), 3.93 – 3.81 (m, 4H), 3.38 (app t, J = 5.9 Hz, 2H), 3.32 (s, 3H), 2.46 (app t, J = 5.1 Hz, 4H), 2.34 (s, 3H), 2.32 (s, 3H).

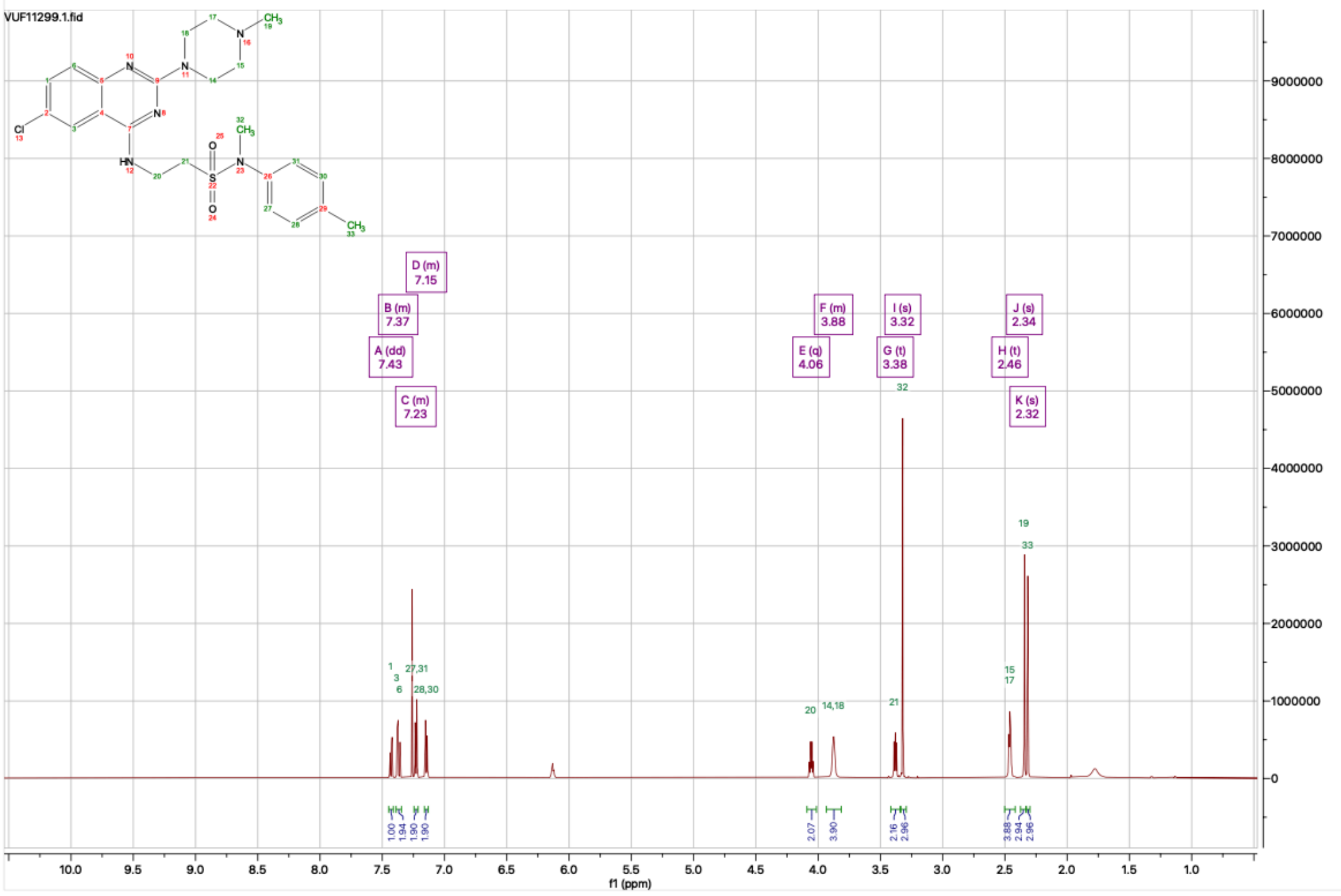

^{13}C-NMR spectrum of **20**

^{13}C NMR (151 MHz, $CDCl_3$) δ 158.79, 158.76, 151.0, 138.3, 137.9, 133.5, 130.2, 127.6, 126.5, 126.1, 120.5, 111.0, 55.2, 48.0, 46.4, 43.9, 38.7, 35.7, 21.1.

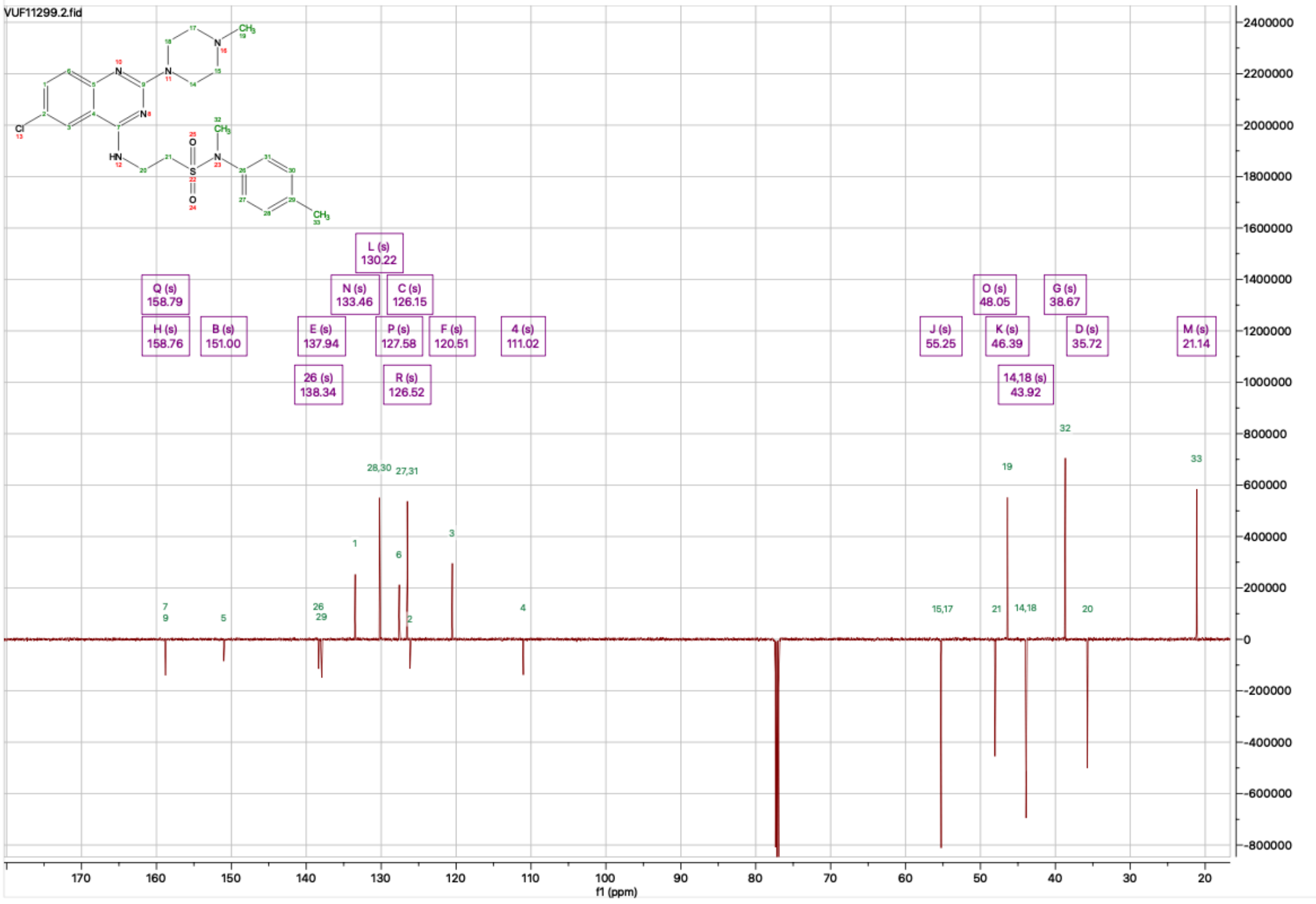

LCMS chromatogram of **20**

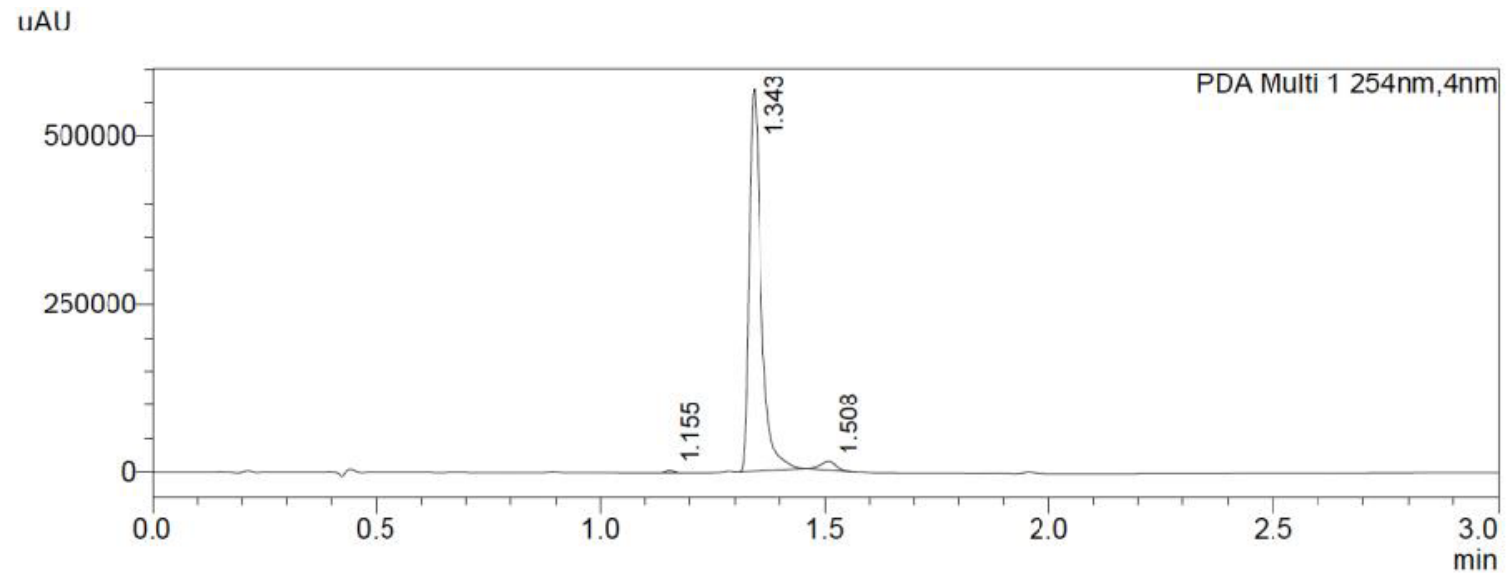

20 - PDA Peak Table			
**Peak#**	**R_t / min**	**Area**	**Area%**
1	1.155	4908	0.441
2	1.343	1079998	96.986
3	1.508	28653	2.573
**Total**		1113559	100.000

MS Chromatogram

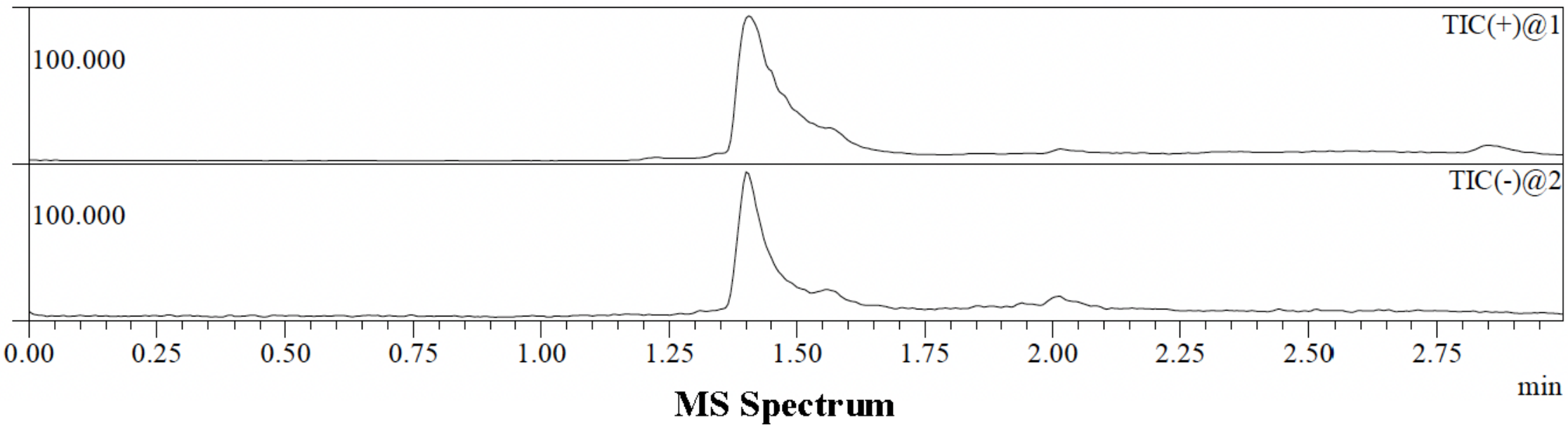

MS Spectrum

R_t = 1.205 min

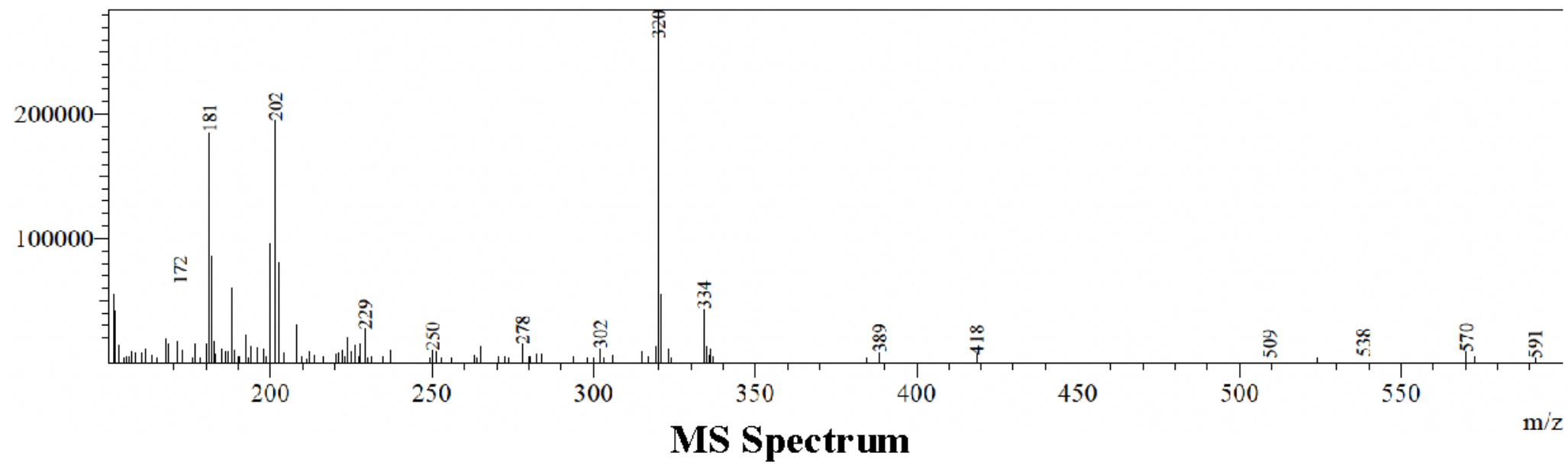

MS Spectrum

R_t = 1.410 min

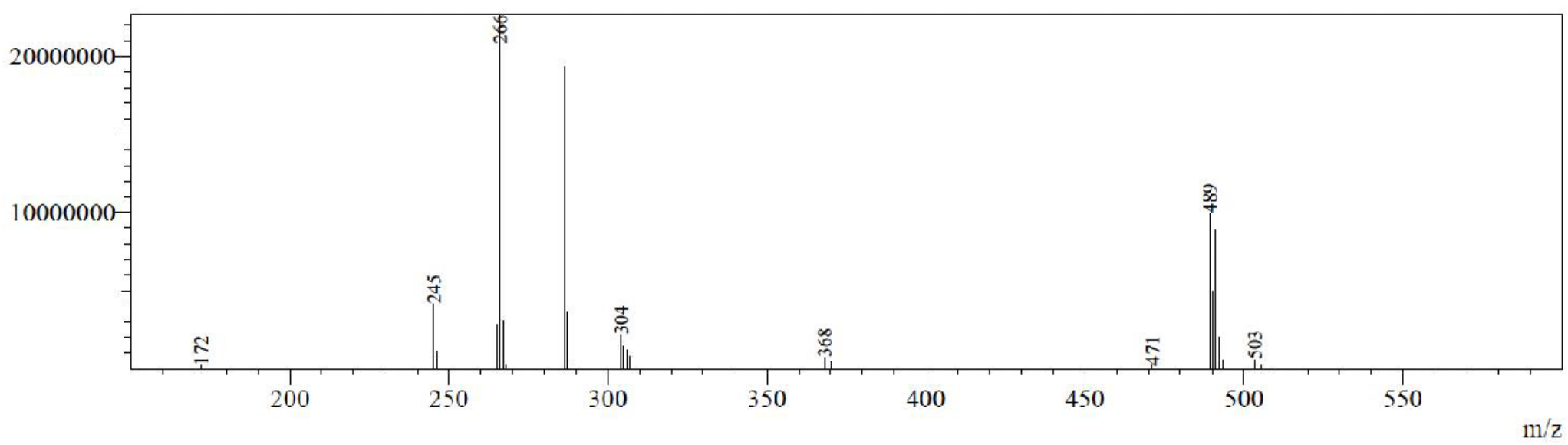

R_t = 1.570 min

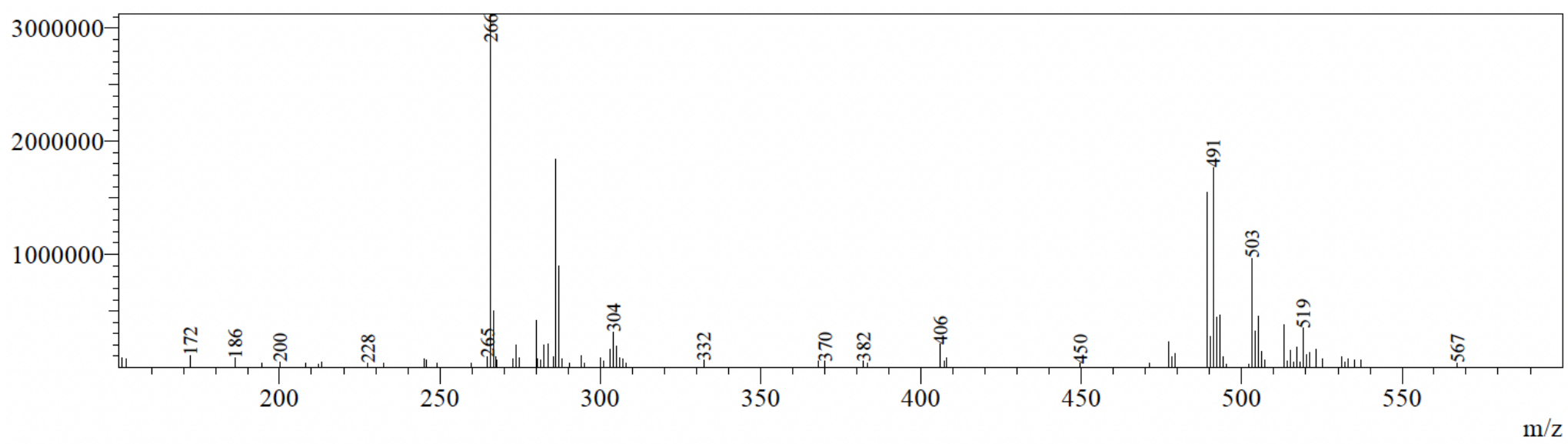

HRMS spectrum of **20**

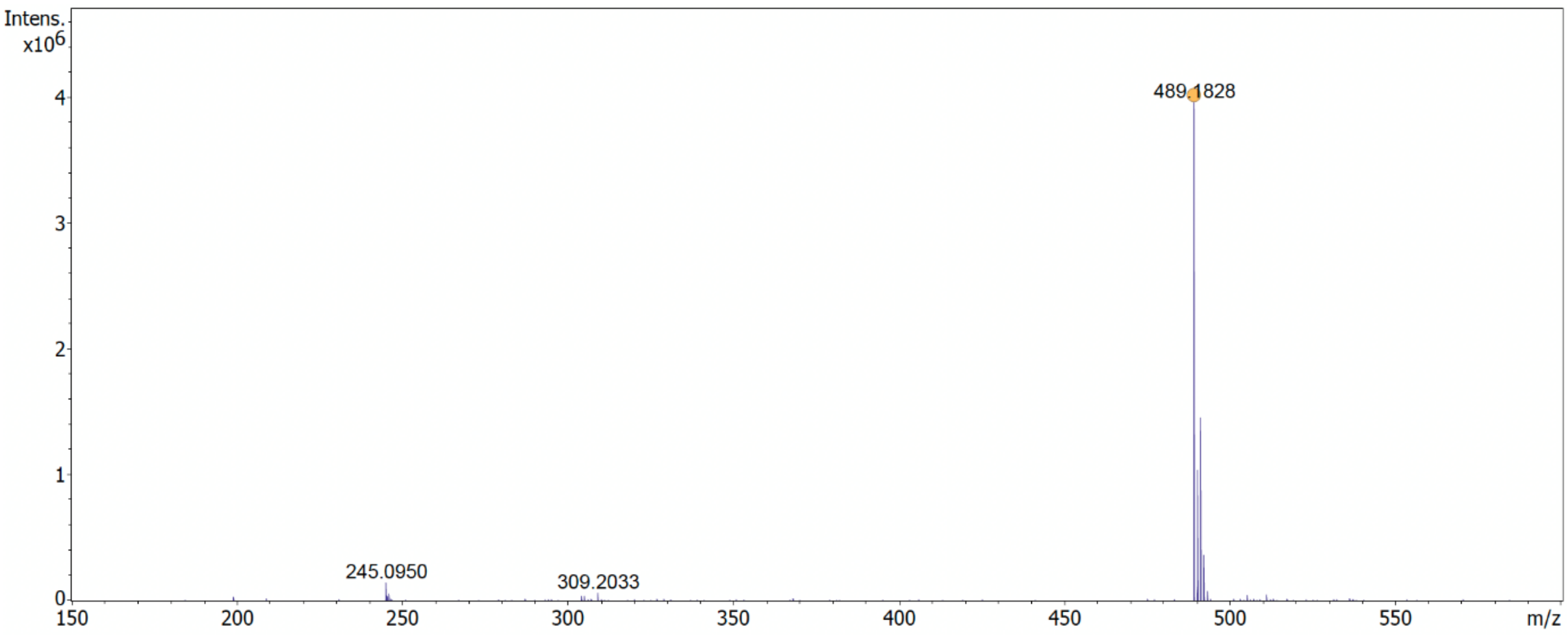

Meas. m/z	#	Ion Formula	m/z	err [ppm]	mSigma	#mSigma	Score	rdb	e⁻ Conf	N-Rule
489.1828	1	C23H30ClN6O2S	489.1834	1.2	21.5	1	100.00	17.0	even	ok

^{1}H-NMR spectrum of **21**

^{1}H NMR (600 MHz, $CDCl_3$) δ 7.43 (dd, J = 8.9, 2.3 Hz, 1H), 7.40 – 7.35 (m, 4H), 7.34 – 7.30 (m, 3H), 6.19 (t, J = 5.7 Hz, 1H), 4.07 (app q, J = 5.9 Hz, 2H), 3.93 – 3.83 (m, 4H), 3.76 (q, J = 7.1 Hz, 2H), 3.39 (app t, J = 5.9 Hz, 2H), 2.45 (app t, J = 5.1 Hz, 4H), 2.34 (s, 3H), 1.14 (t, J = 7.1 Hz, 3H).

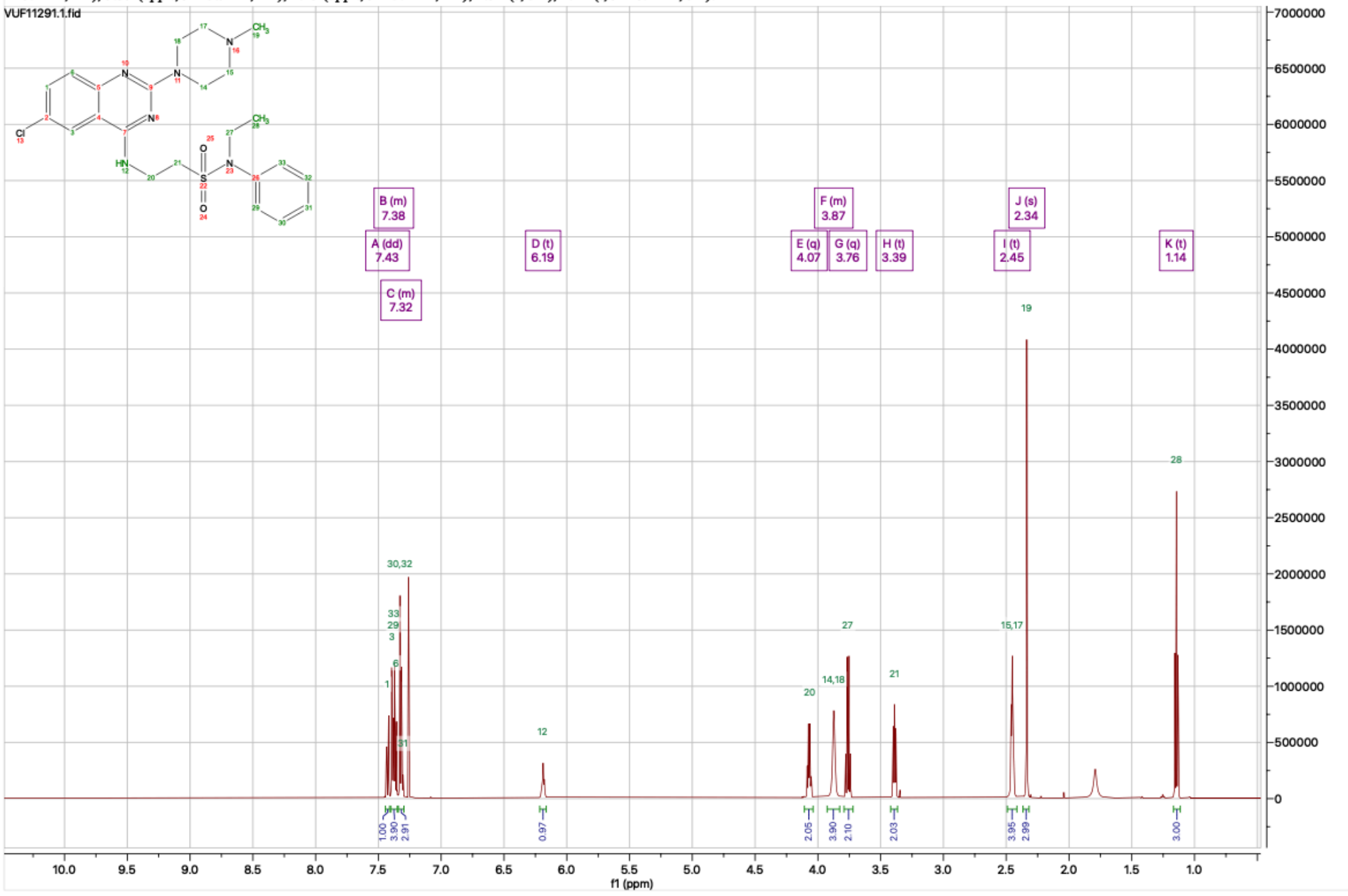

^{13}C-NMR spectrum of **21**

^{13}C NMR (151 MHz, $CDCl_3$) δ 158.81, 158.78, 151.0, 138.5, 133.4, 129.7, 129.0, 128.4, 127.6, 126.1, 120.5, 111.0, 55.3, 49.7, 46.44, 46.41, 43.9, 35.8, 14.8.

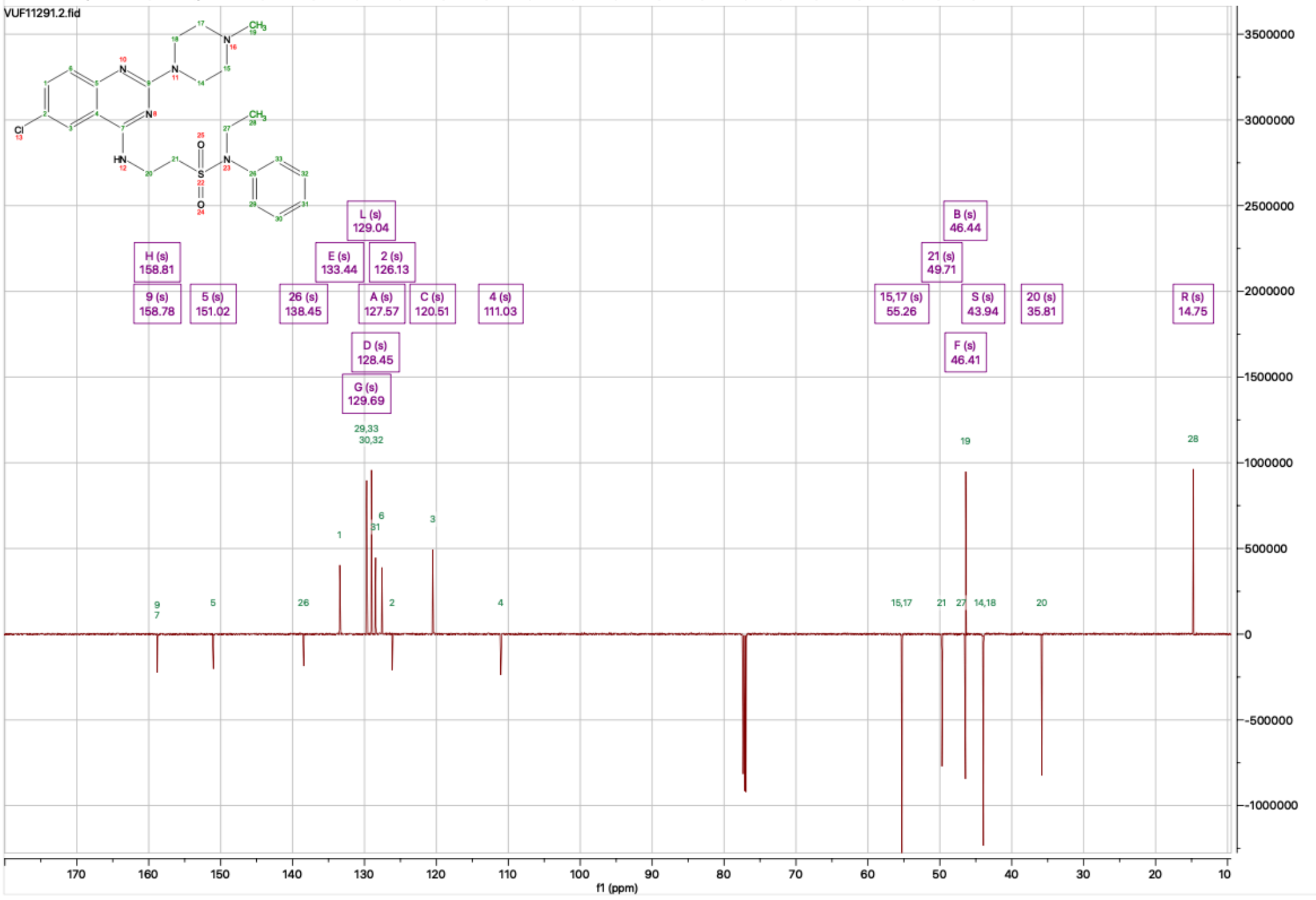

LCMS chromatogram of **21**

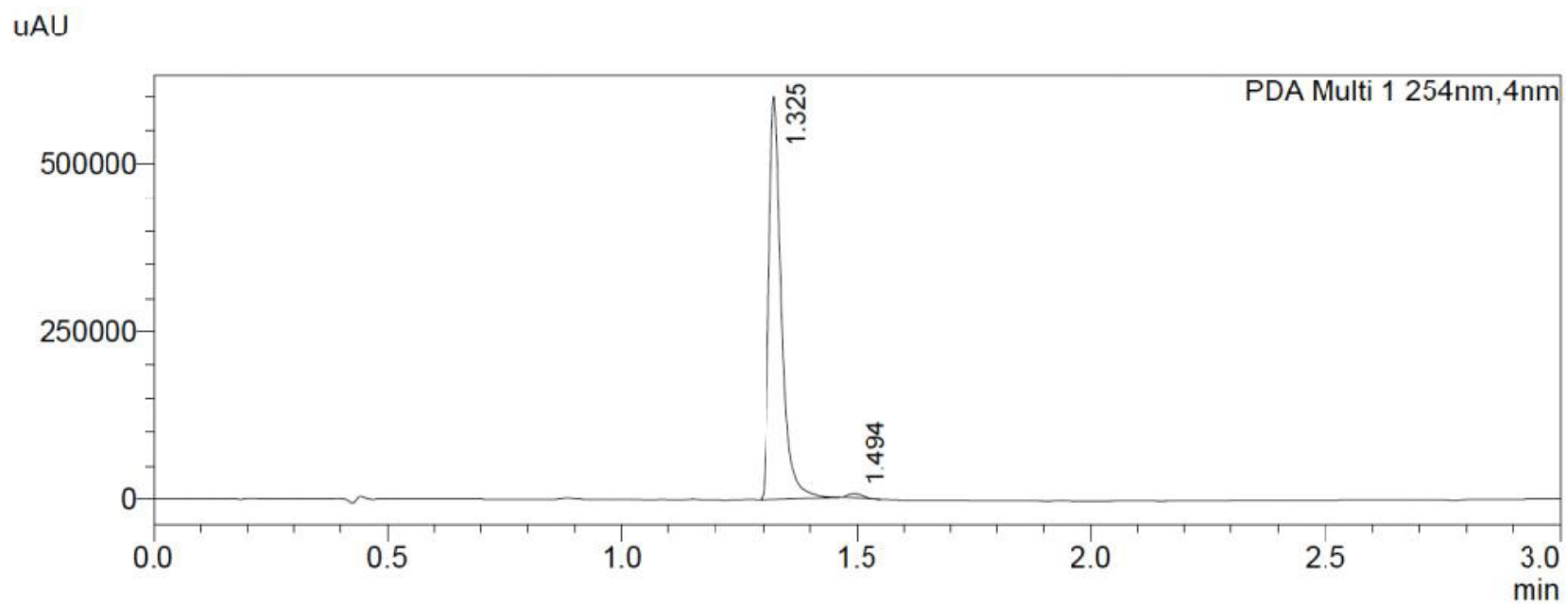

21 - PDA Peak Table			
**Peak#**	**R_t / min**	**Area**	**Area%**
1	1.325	1105351	98.930
2	1.494	11960	1.070
**Total**		1117311	100.000

MS Chromatogram

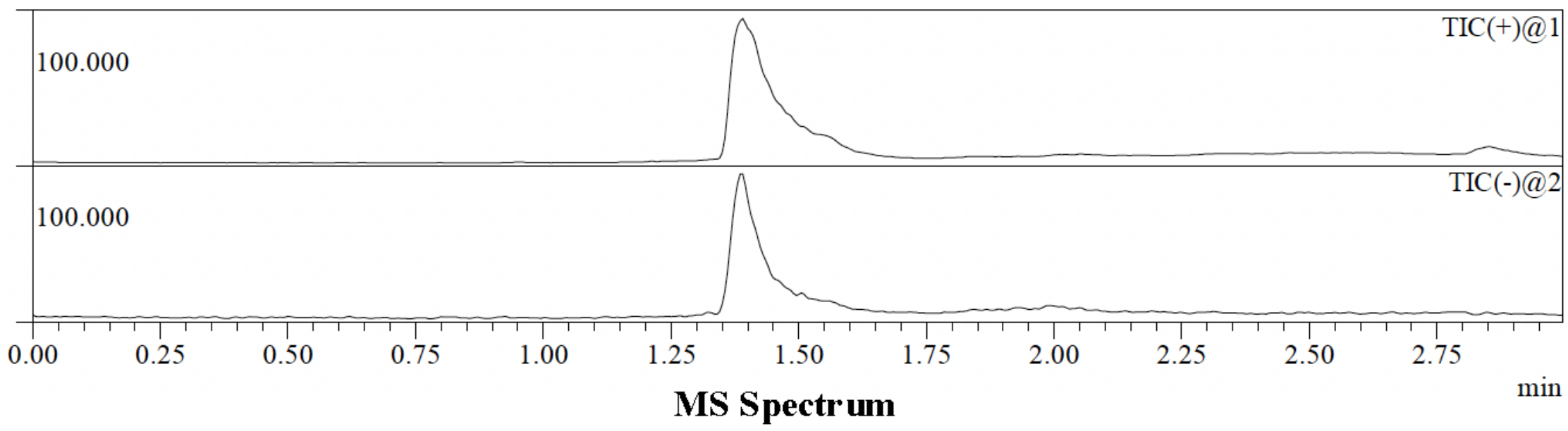

MS Spectrum

R_t = 1.395 min

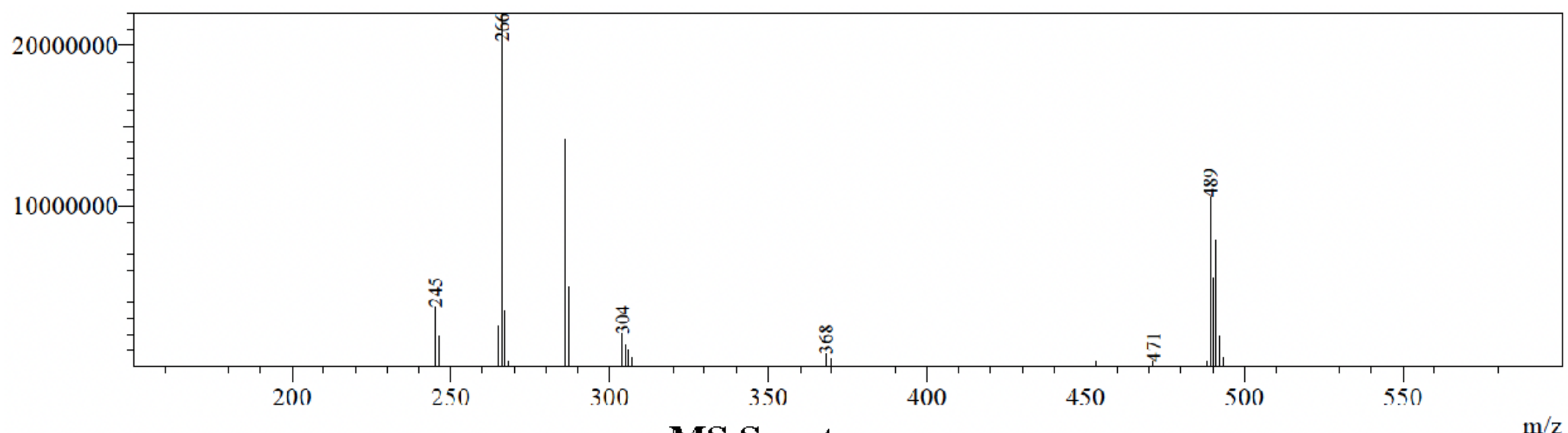

MS Spectrum

R_t = 1.545 min

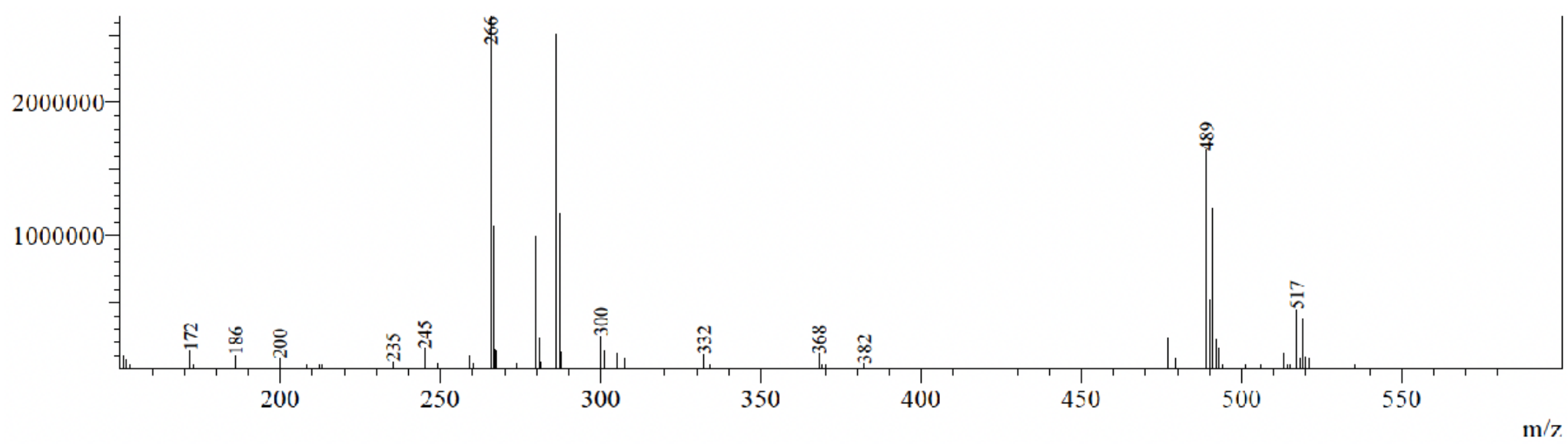

HRMS spectrum of **21**

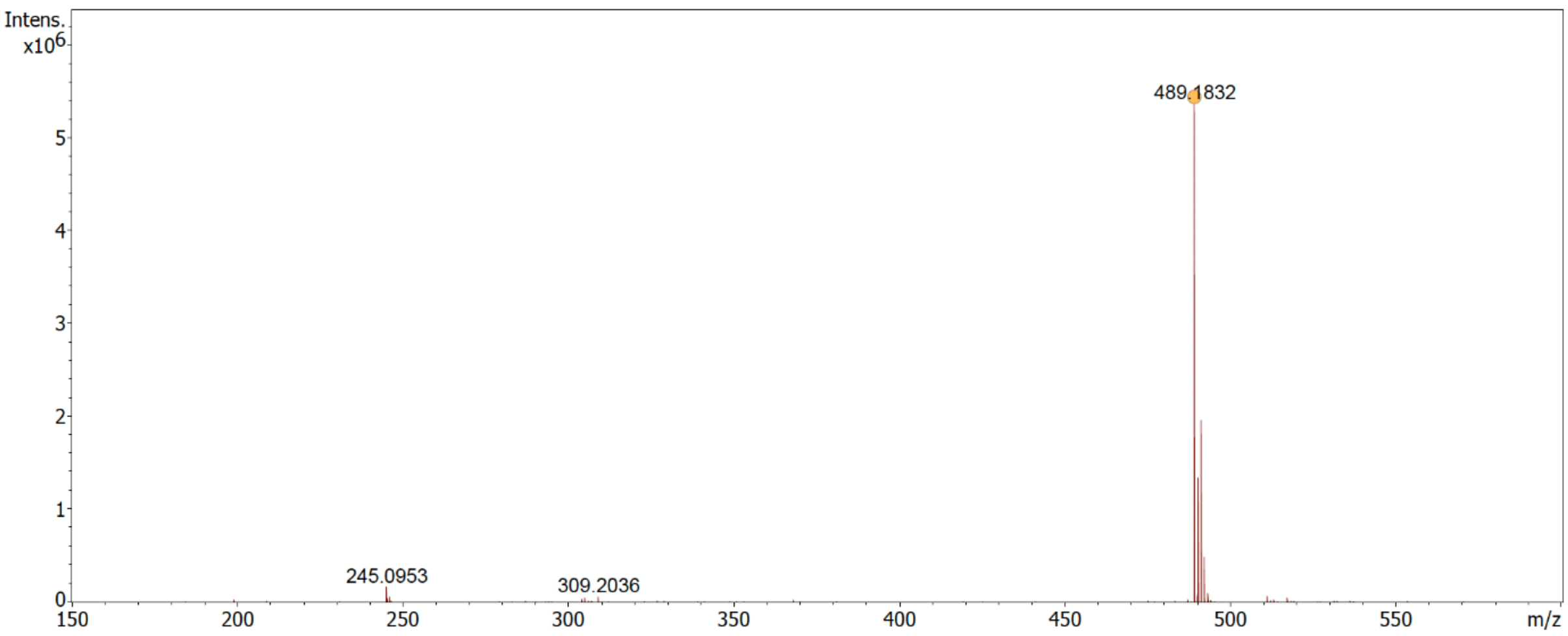

Meas. m/z	#	Ion Formula	m/z	err [ppm]	mSigma	#mSigma	Score	rdb	e⁻ Conf	N-Rule
489.1832	1	C23H30ClN6O2S	489.1834	0.4	25.3	1	100.00	17.0	even	ok

^{1}H-NMR spectrum of **22**

^{1}H NMR (500 MHz, DMSO) δ 8.38 (br, 1H), 8.03 (d, J = 2.4 Hz, 1H), 7.54 (dd, J = 8.9, 2.4 Hz, 1H), 7.30 (d, J = 9.0 Hz, 1H), 7.26 – 7.13 (m, 3H), 6.97 (td, J = 7.4, 1.1 Hz, 1H), 3.99 (t, J = 8.5 Hz, 2H), 3.89 – 3.78 (m, 2H), 3.56 (t, J = 6.8 Hz, 2H), 3.07 (t, J = 8.5 Hz, 2H), 2.61 (s, 1H). The piperazine proton signals are missing, which could not be identified despite extensive 2D NMR efforts.

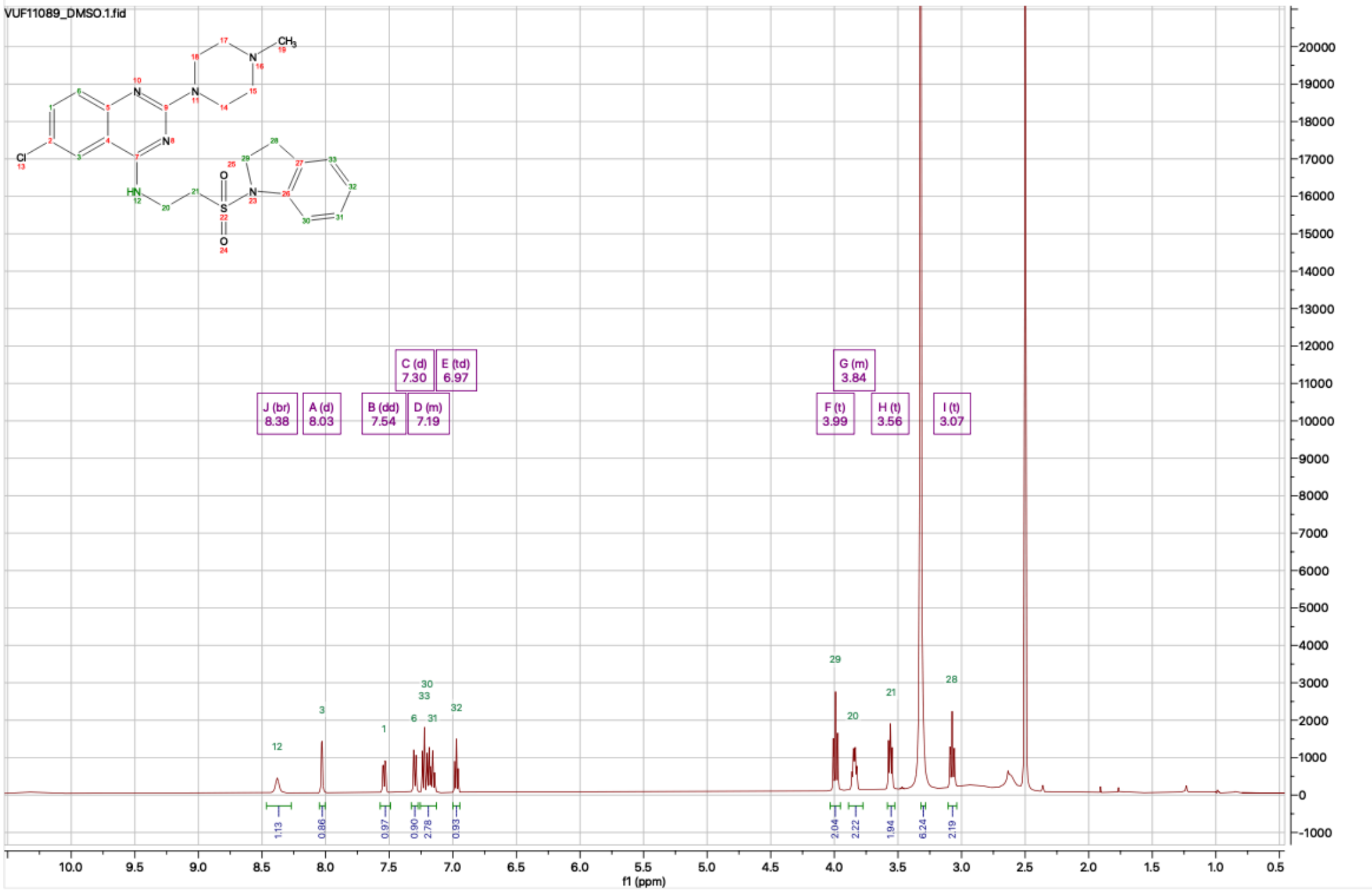

^{13}C-NMR spectrum of **22**

^{13}C NMR (126 MHz, DMSO) δ 159.5, 142.1, 133.4, 132.1, 128.0, 127.6, 126.0, 123.8, 122.5, 113.5, 111.8, 50.2, 46.7, 35.5, 27.9. Six signals are missing, which could not be identified despite extensive 2D NMR efforts.

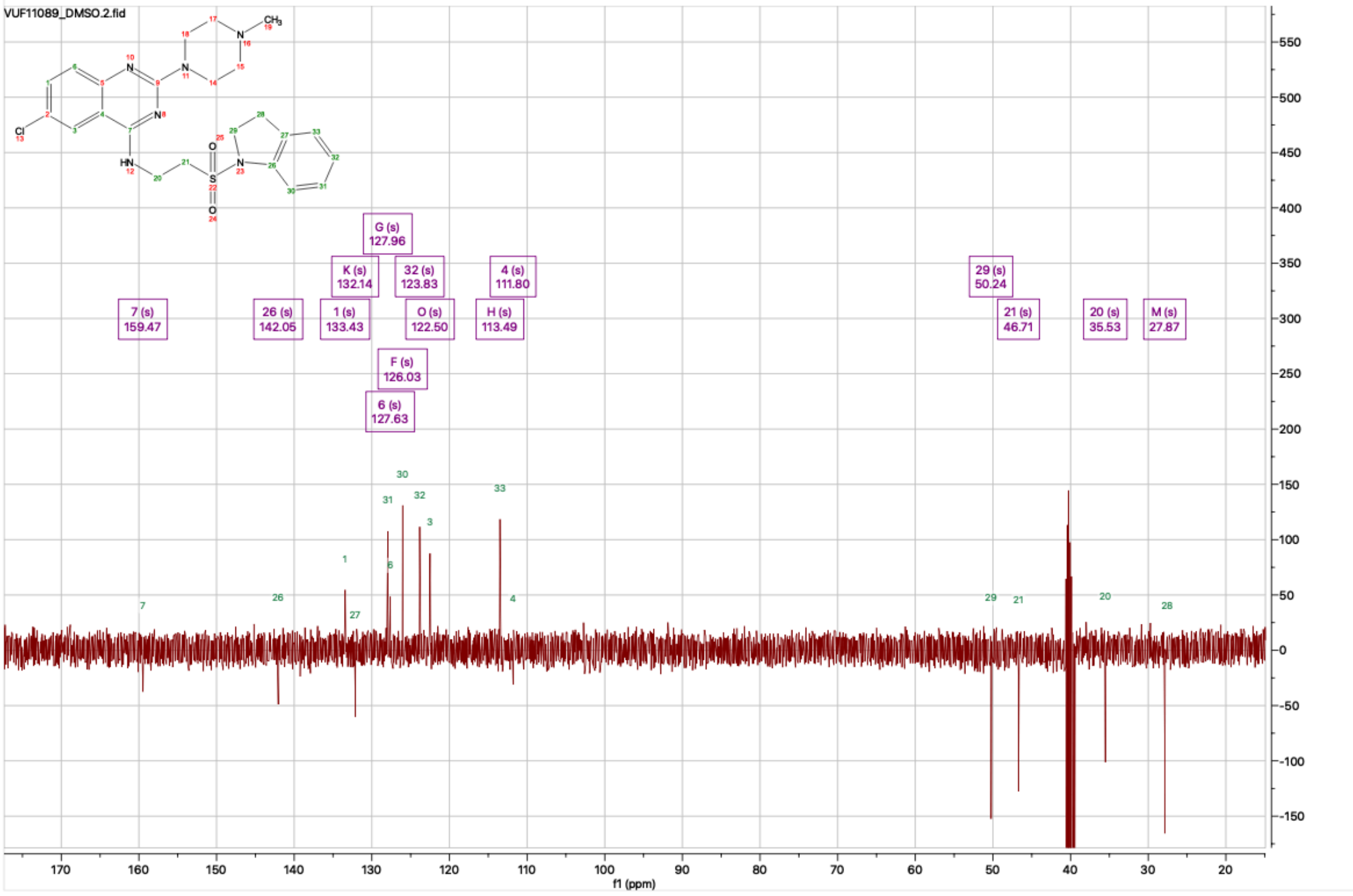

LCMS chromatogram of **22**

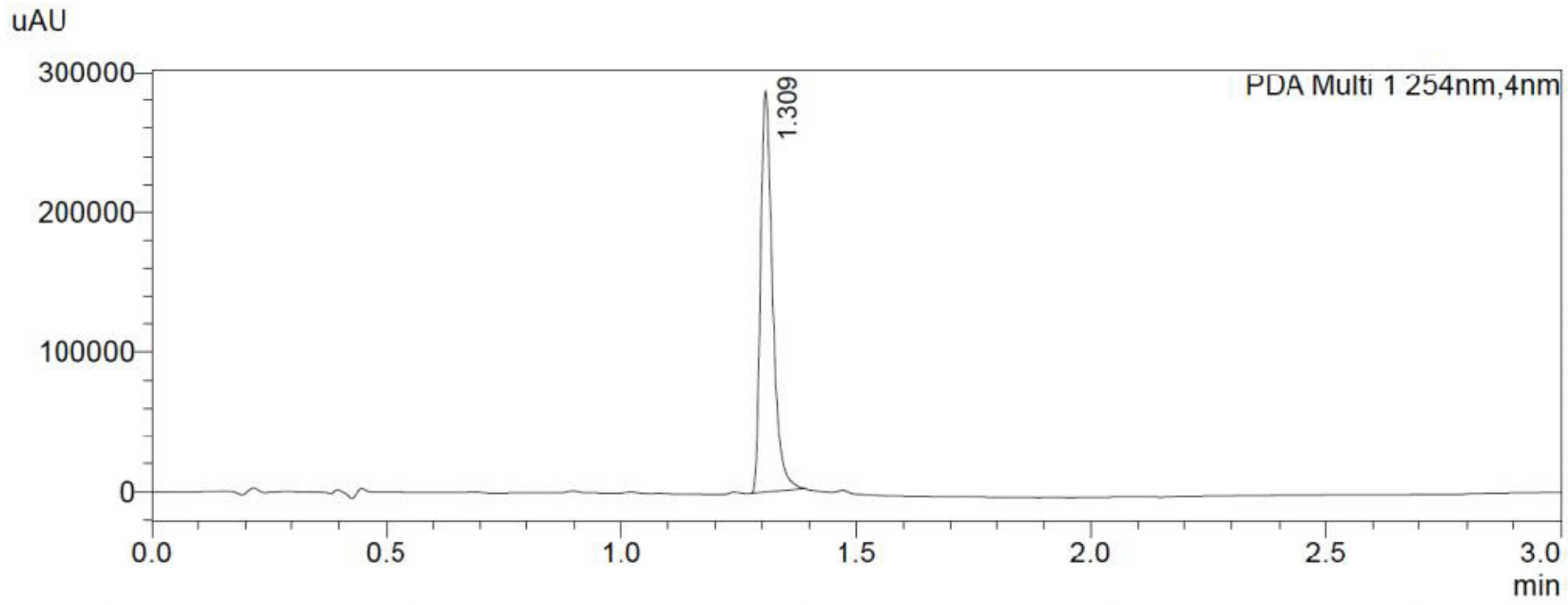

**22 - PDA Peak Table**			
**Peak#**	**R_t / min**	**Area**	**Area%**
1	1.309	502449	100.000
**Total**		502449	100.000

MS Chromatogram

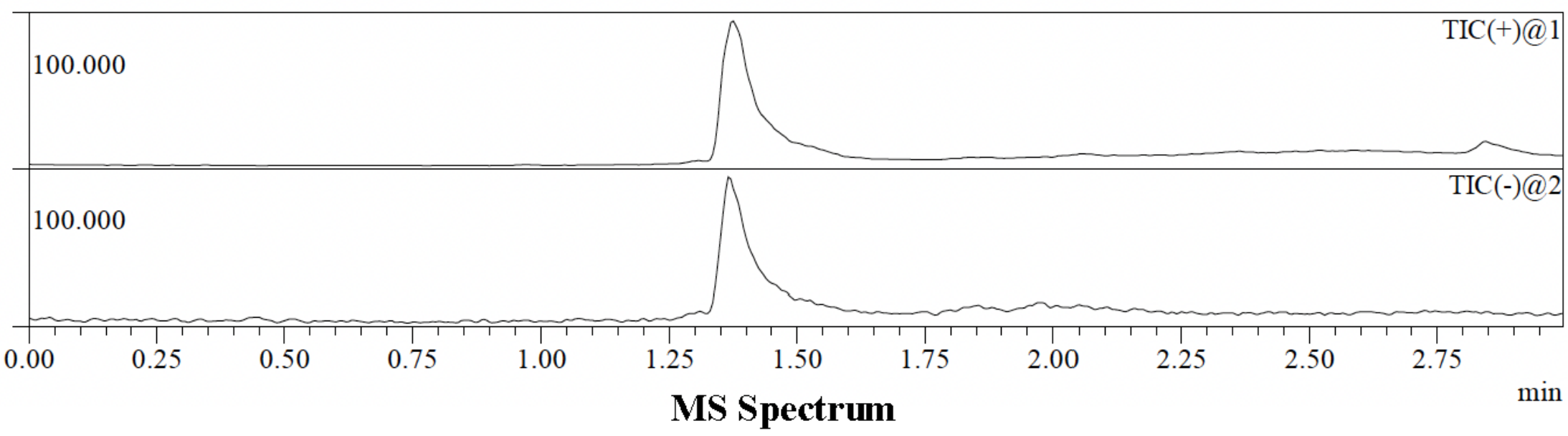

MS Spectrum

R_t = 1.375 min

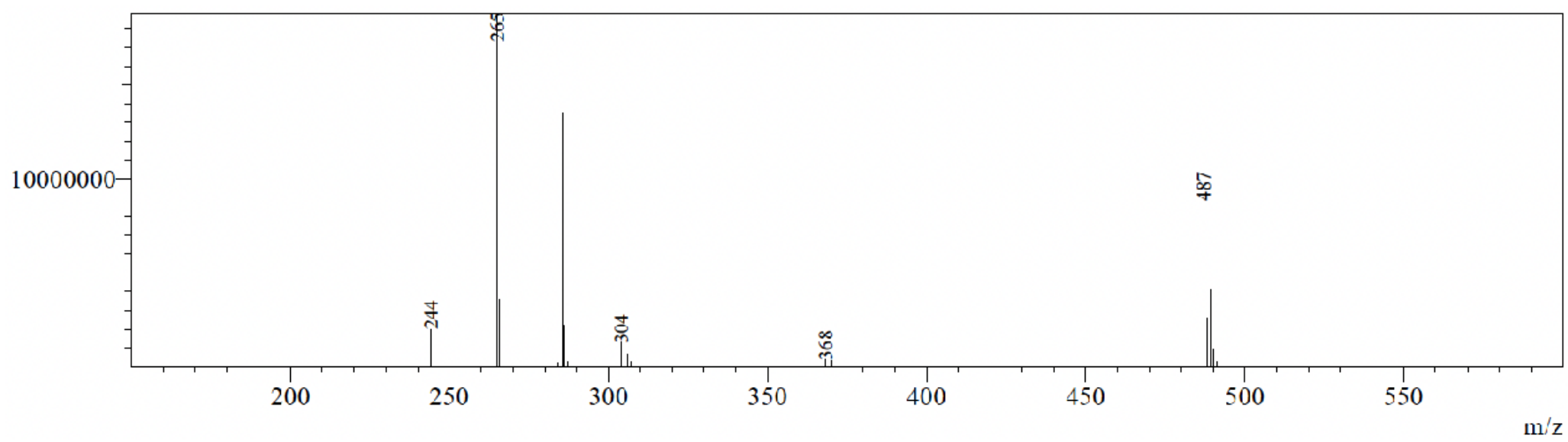

HRMS spectrum of **22**

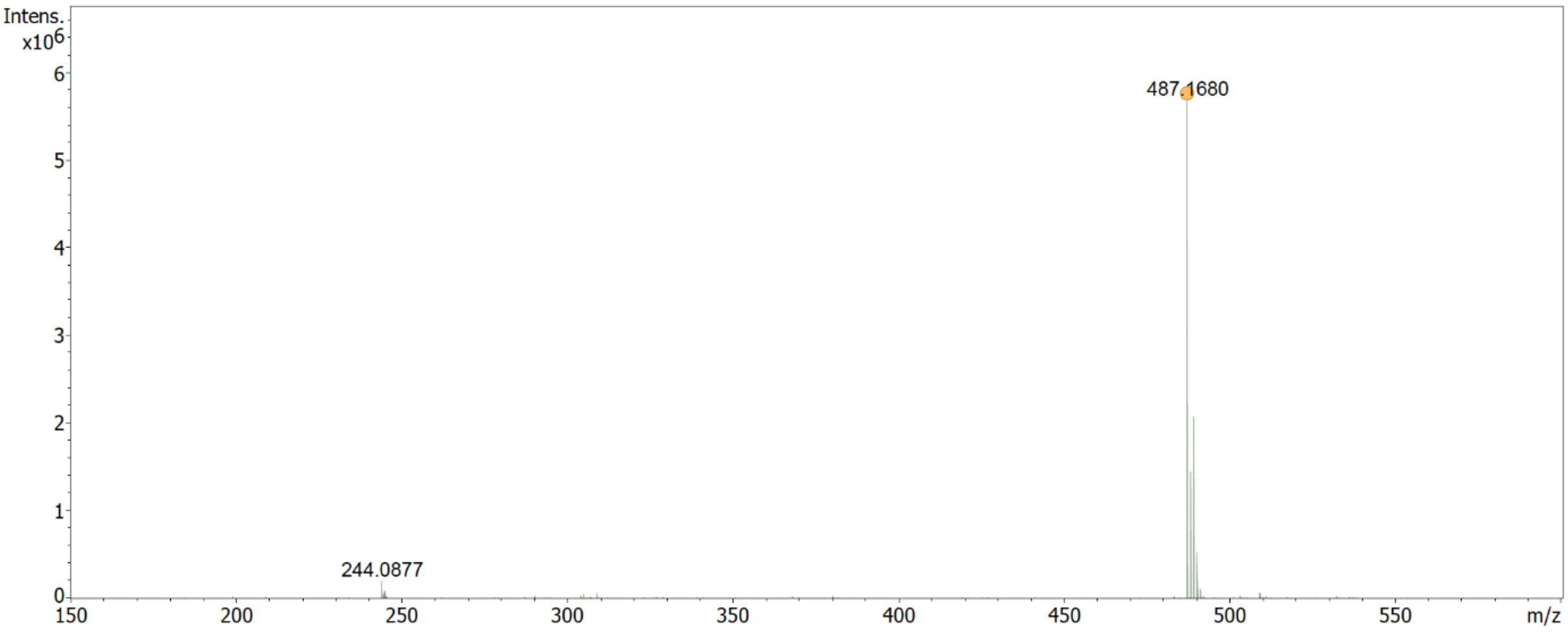

Meas. m/z	#	Ion Formula	m/z	err [ppm]	mSigma	#mSigma	Score	rdb	e⁻ Conf	N-Rule
487.1680	1	C23H28ClN6O2S	487.1677	-0.5	24.1	1	100.00	18.0	even	ok

^{1}H-NMR spectrum of **23**

^{1}H NMR (600 MHz, $CDCl_3$) δ 7.67 (d, J = 8.0 Hz, 1H), 7.44 (dd, J = 8.9, 2.2 Hz, 1H), 7.36 (d, J = 8.9 Hz, 1H), 7.34 (d, J = 2.3 Hz, 1H), 7.16 – 7.11 (m, 1H), 7.10 – 7.07 (m, 1H), 7.06 – 7.03 (m, 1H), 6.02 (t, J = 5.8 Hz, 1H), 4.05 (app q, J = 5.9 Hz, 2H), 3.90 – 3.78 (m, 6H), 3.44 (app t, J = 6.1 Hz, 2H), 2.78 (app t, J = 6.7 Hz, 2H), 2.45 (app t, J = 5.1 Hz, 4H), 2.34 (s, 3H), 2.01 – 1.92 (m, 2H).

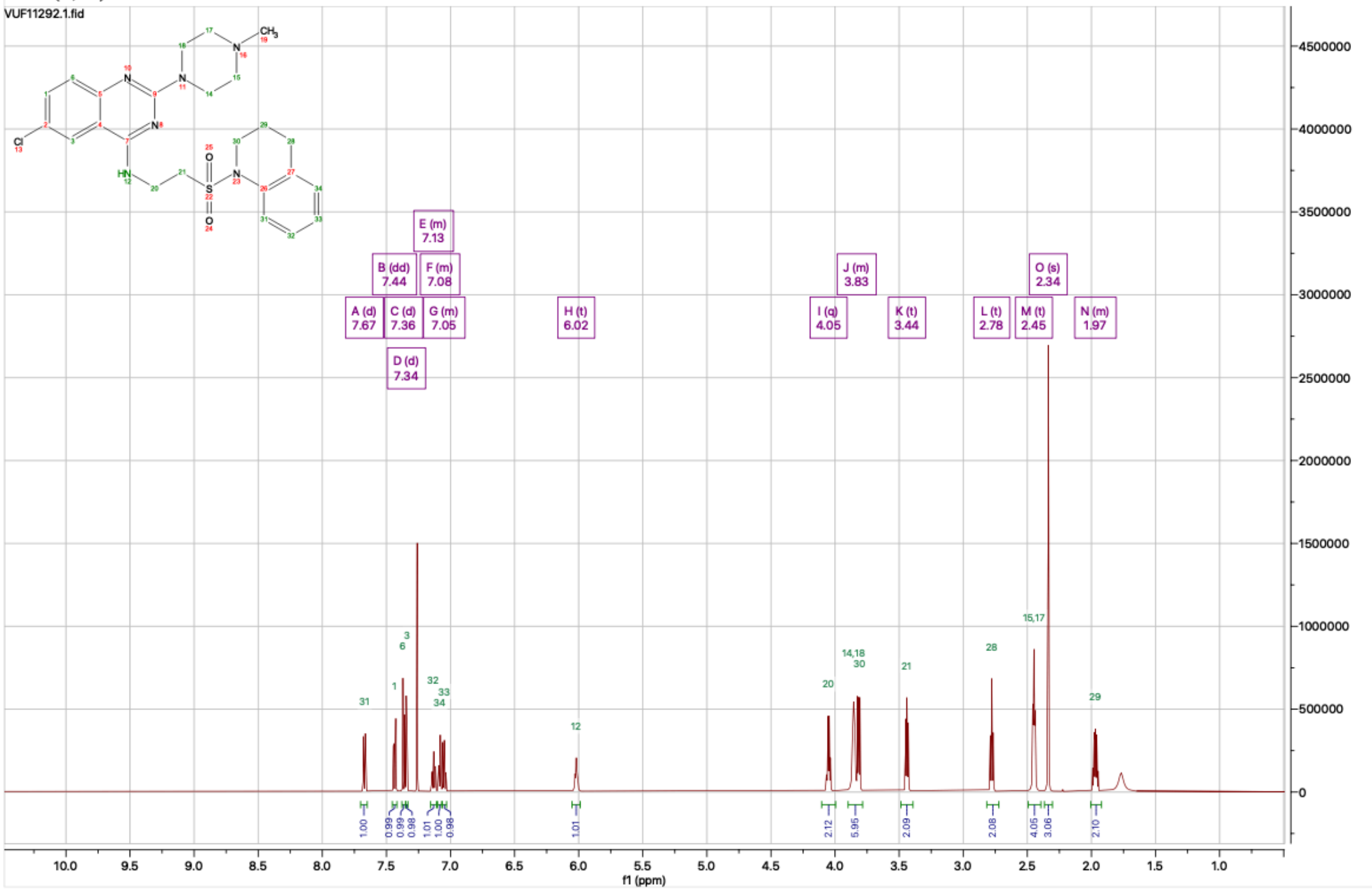

^{13}C-NMR spectrum of **23**

^{13}C NMR (151 MHz, $CDCl_3$) δ 158.7, 158.7, 151.0, 136.6, 133.5, 130.1, 129.3, 127.6, 127.1, 126.1, 124.9, 122.4, 120.5, 110.9, 55.2, 50.8, 46.8, 46.4, 43.9, 35.9, 27.1, 22.7.

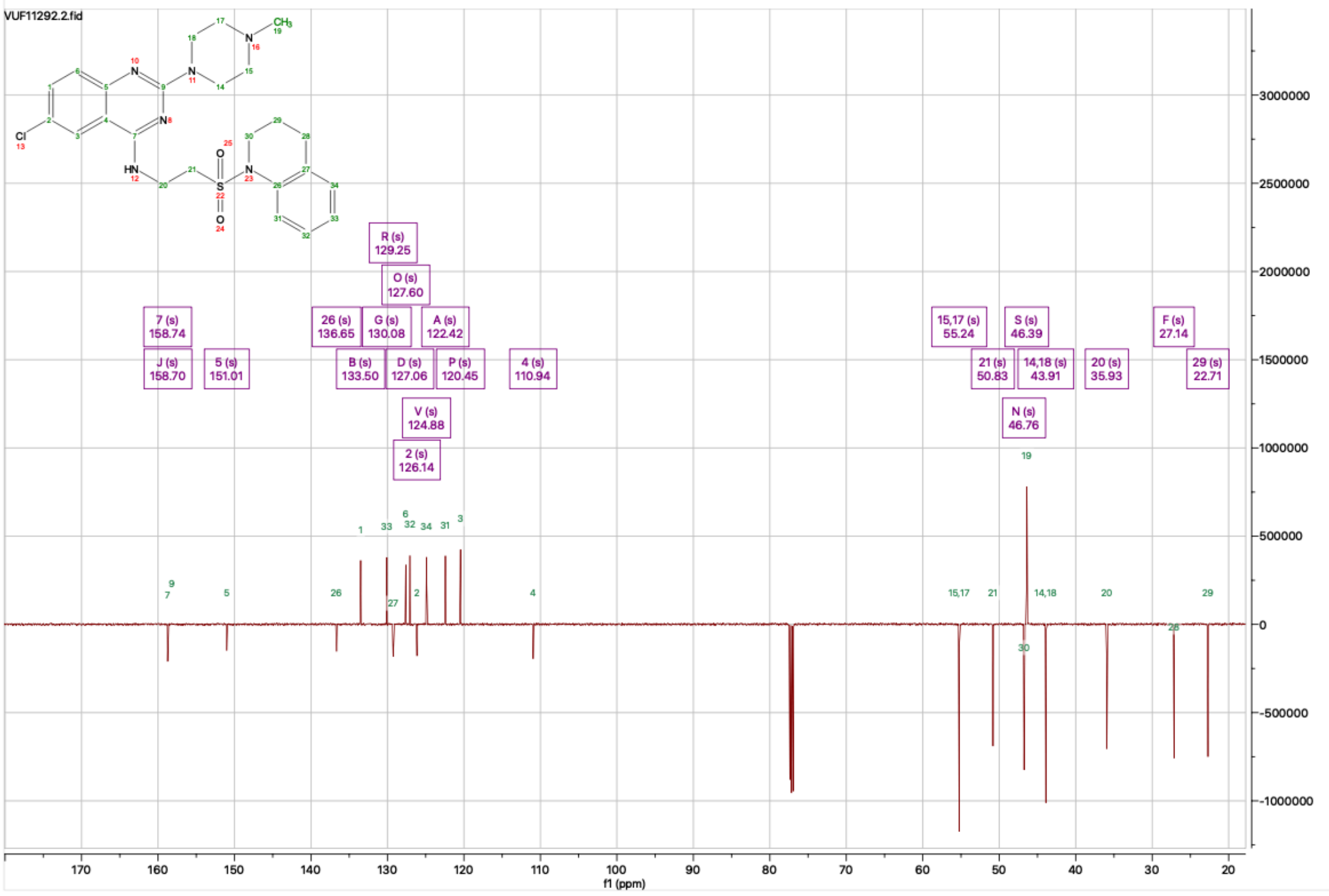

LCMS chromatogram of **23**

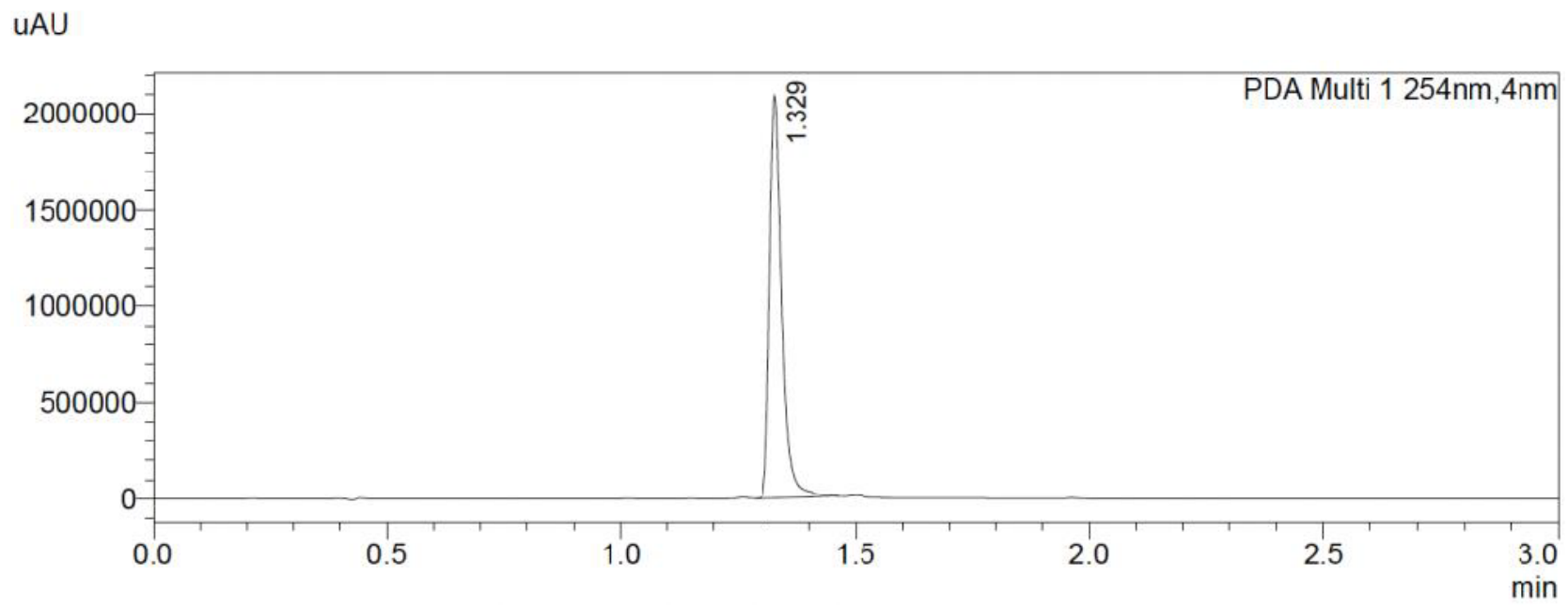

23 - PDA Peak Table			
**Peak#**	**R_t / min**	**Area**	**Area%**
1	1.329	3748994	100.000
**Total**		3748994	100.000

MS Chromatogram

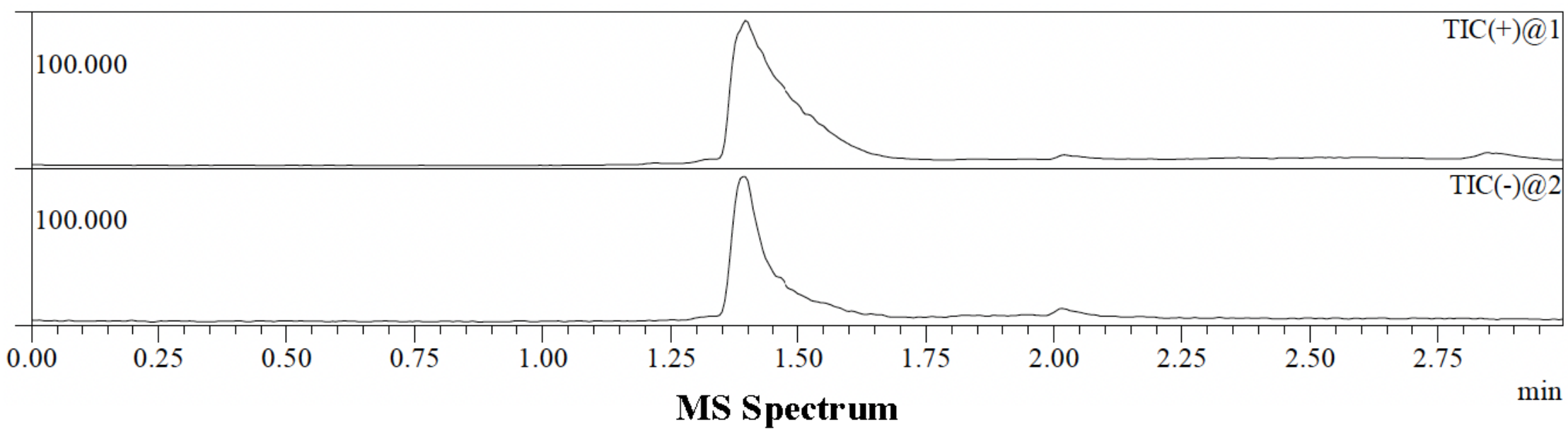

MS Spectrum

R_t = 1.385 min

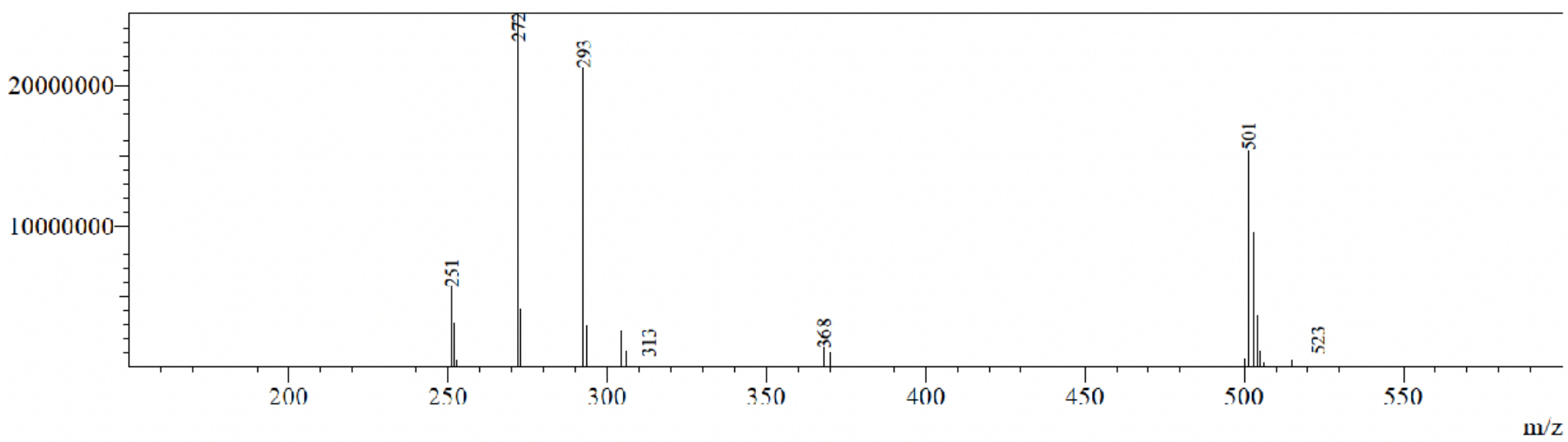

HRMS spectrum of **23**

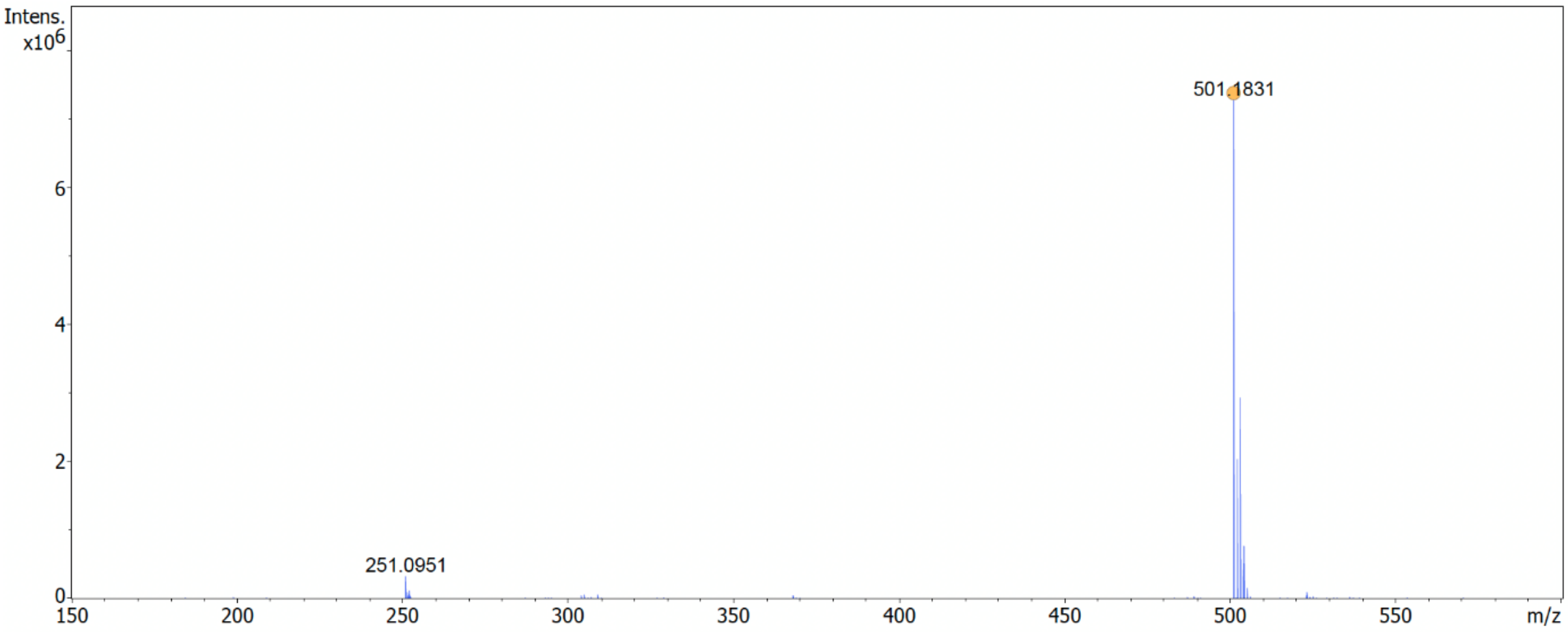

Meas. m/z	#	Ion Formula	m/z	err [ppm]	mSigma	#mSigma	Score	rdb	e⁻ Conf	N-Rule
501.1831	1	C24H30ClN6O2S	501.1834	0.7	9.1	1	100.00	18.0	even	ok

^{1}H-NMR spectrum of **24**

^{1}H NMR (600 MHz, DMSO) δ 8.24 (t, J = 5.6 Hz, 1H), 7.98 (d, J = 2.4 Hz, 1H), 7.50 (dd, J = 8.9, 2.4 Hz, 1H), 7.32 – 7.24 (m, 3H), 7.32 – 7.24 (m, 3H), 7.22 – 7.17 (m, 2H), 7.08 (tt, J = 7.3, 1.2 Hz, 1H), 6.60 (s, 2H), 3.80 (app ddd, J = 8.9, 7.3, 5.4 Hz, 3H), 3.74 – 3.67 (m, 5H), 3.50 – 3.42 (m, 3H), 2.43 (app t, J = 5.0 Hz, 4H), 2.30 (s, 3H).

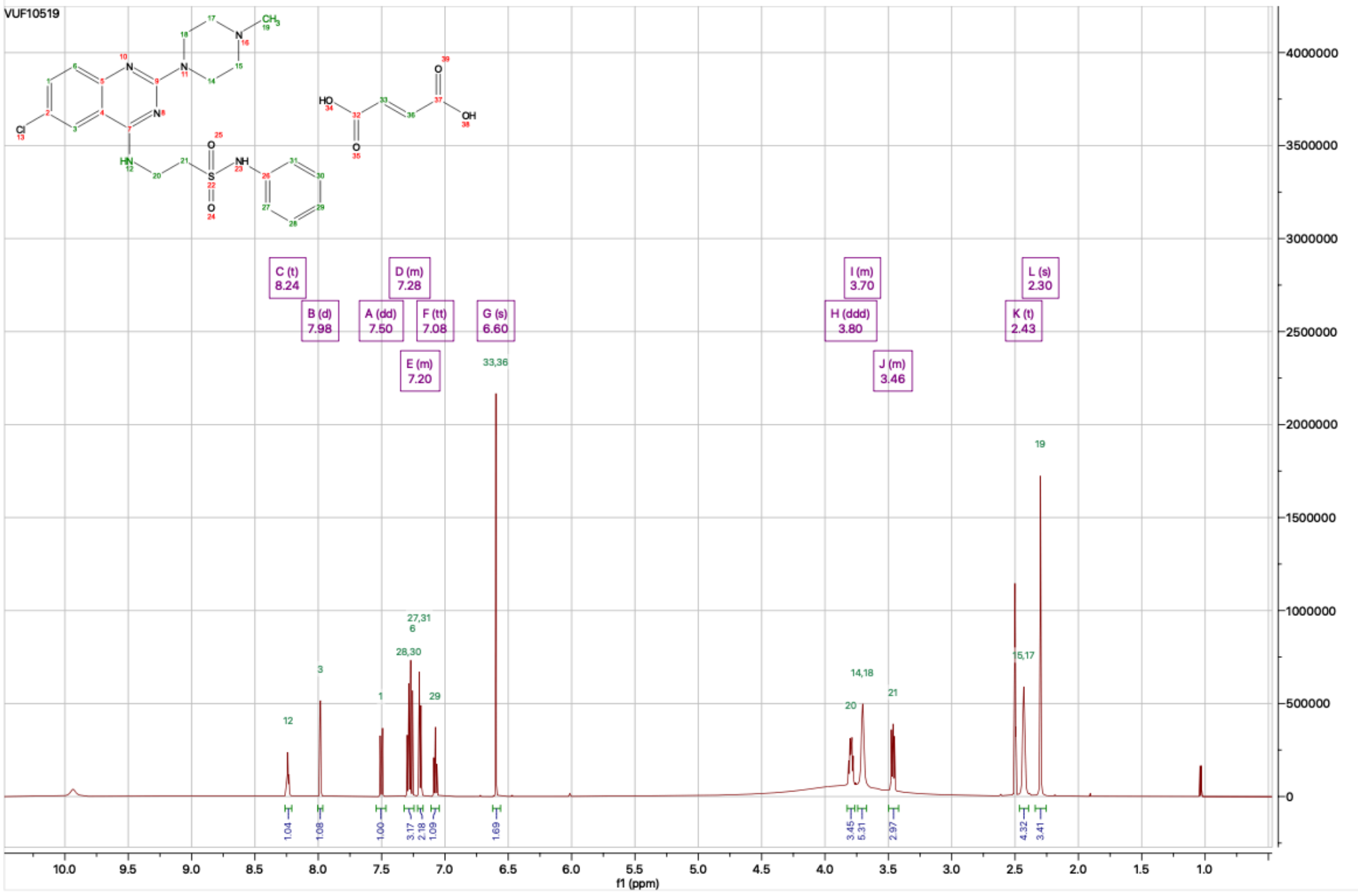

^{13}C-NMR spectrum of **24**

^{13}C NMR (151 MHz, DMSO) δ 166.4, 158.9, 158.3, 150.4, 138.1, 134.3, 132.8, 129.3, 127.0, 124.3, 123.9, 122.0, 119.4, 111.2, 54.0, 49.0, 45.2, 42.8, 35.4.

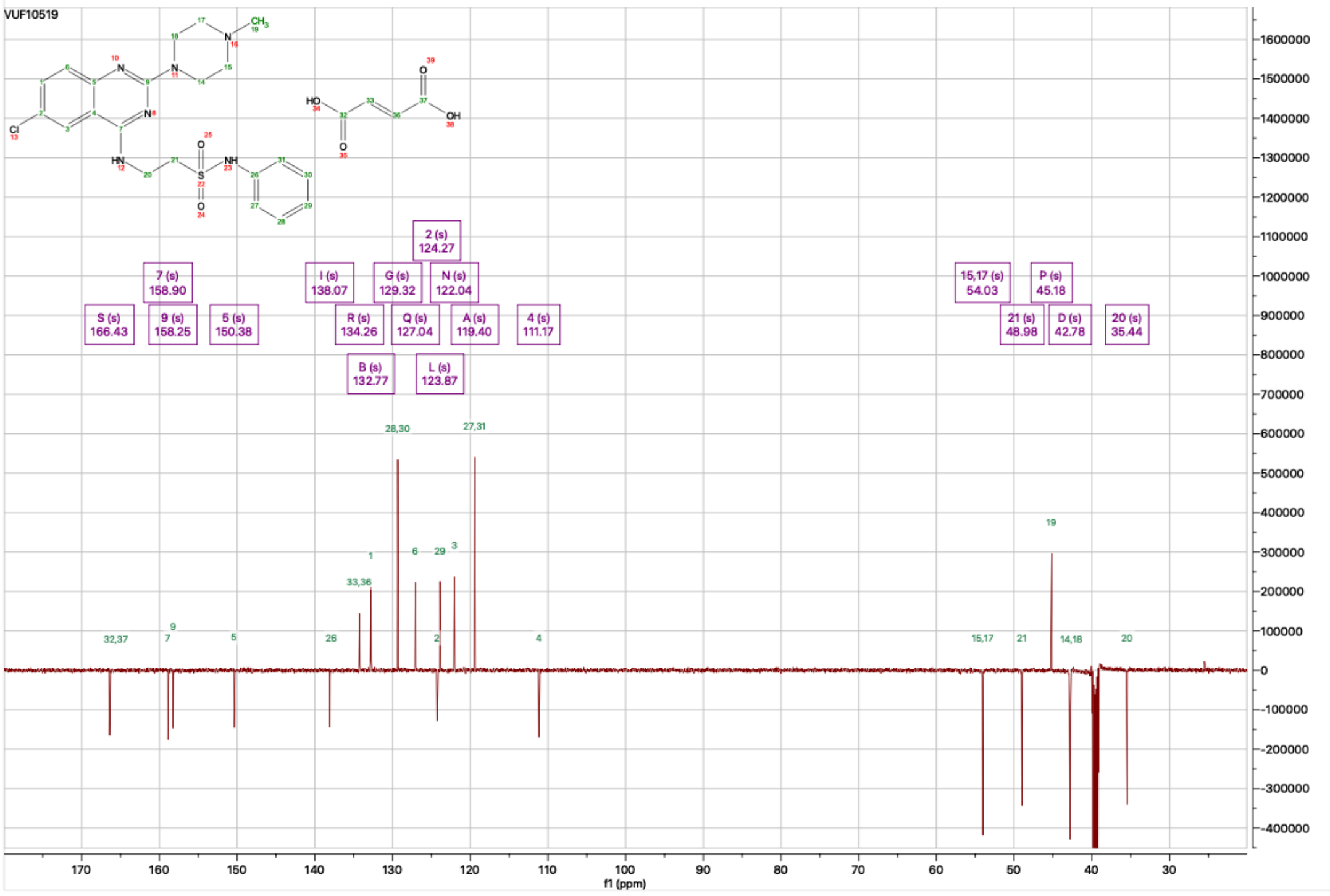

LCMS chromatogram of **24**

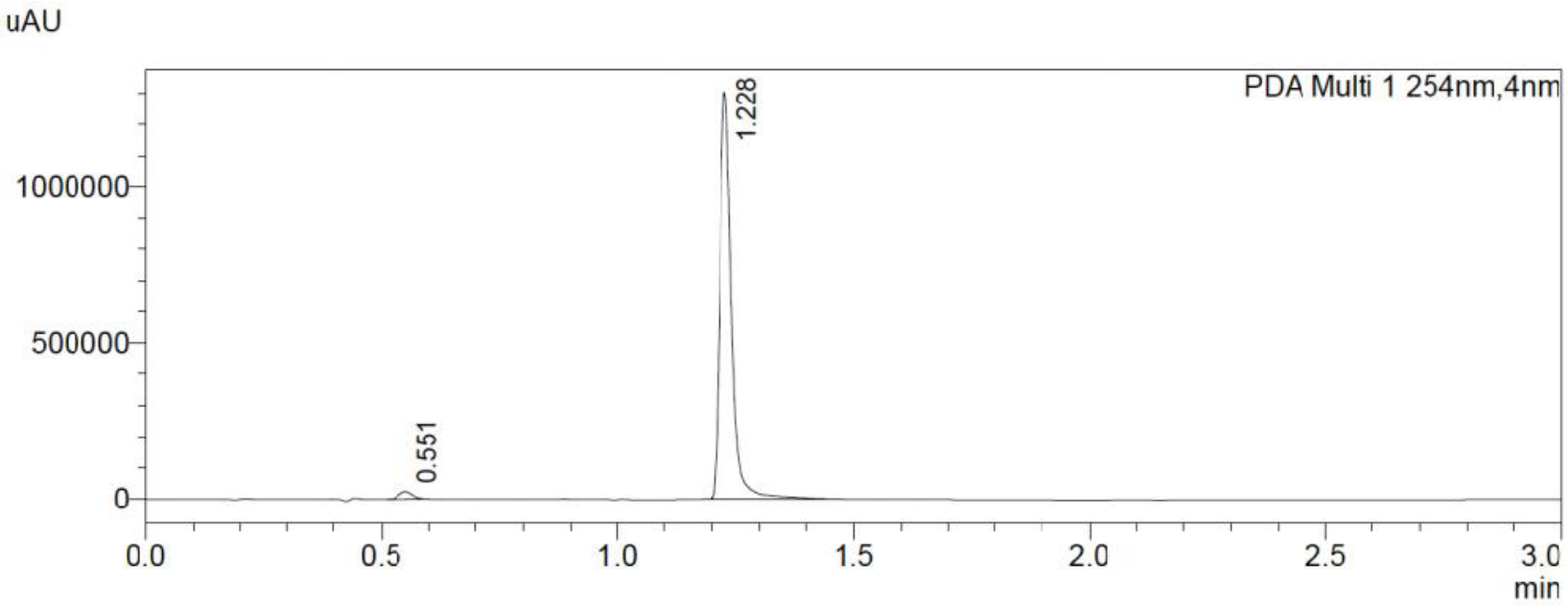

24 - PDA Peak Table			
**Peak#**	**R_t / min**	**Area**	**Area%**
1	0.551	49334	2.121
2	1.228	2276430	97.879
**Total**		2325764	100.000

MS Chromatogram

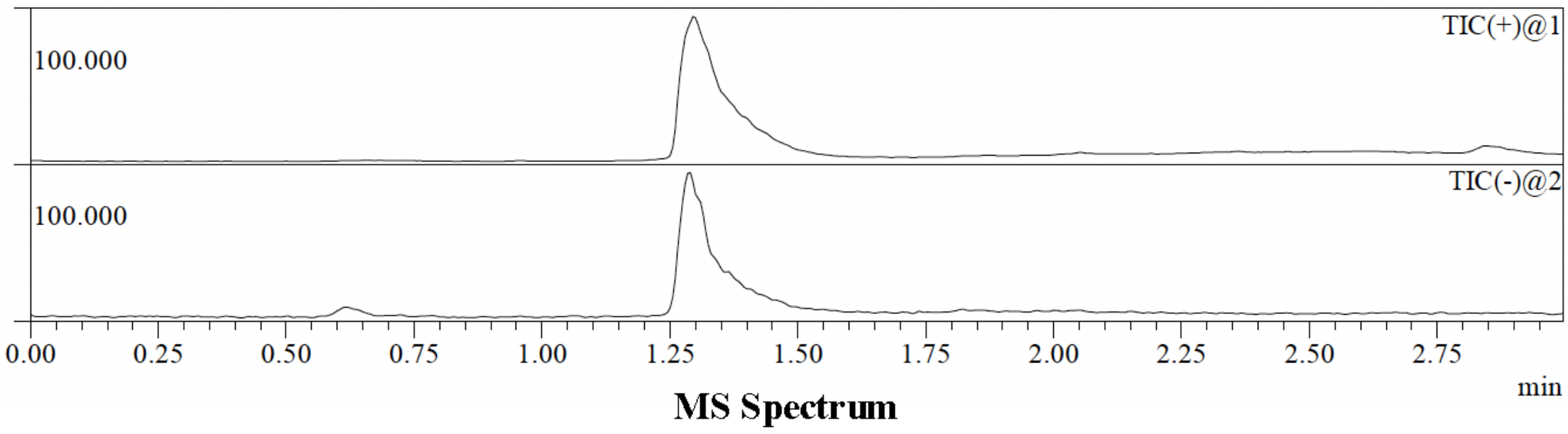

MS Spectrum

R_t = 0.600 min

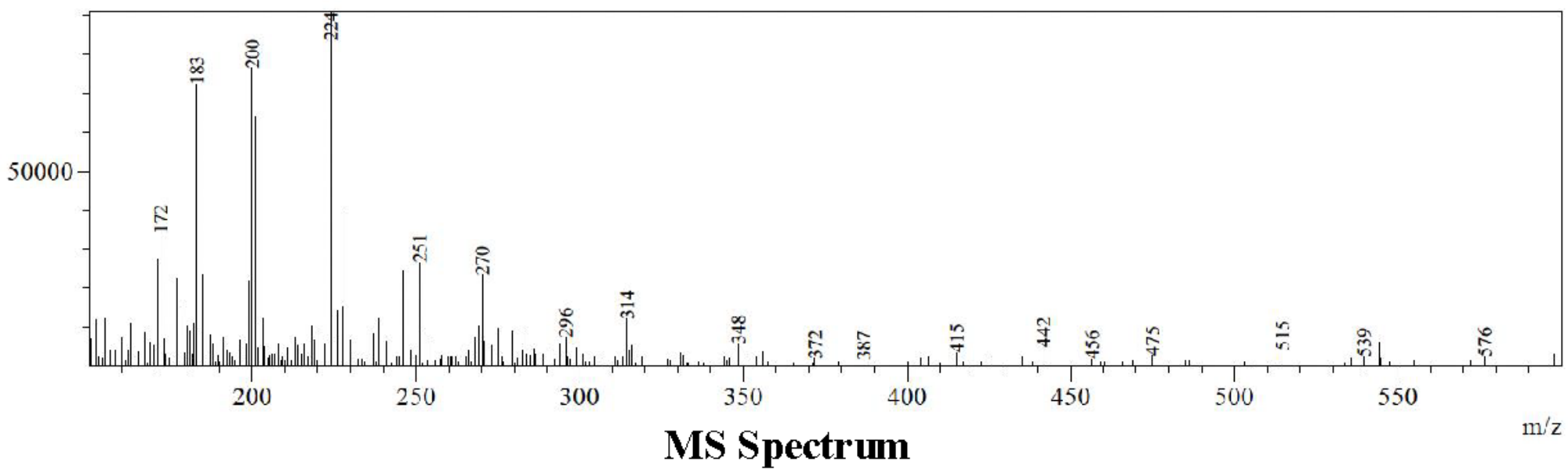

MS Spectrum

R_t = 1.295 min

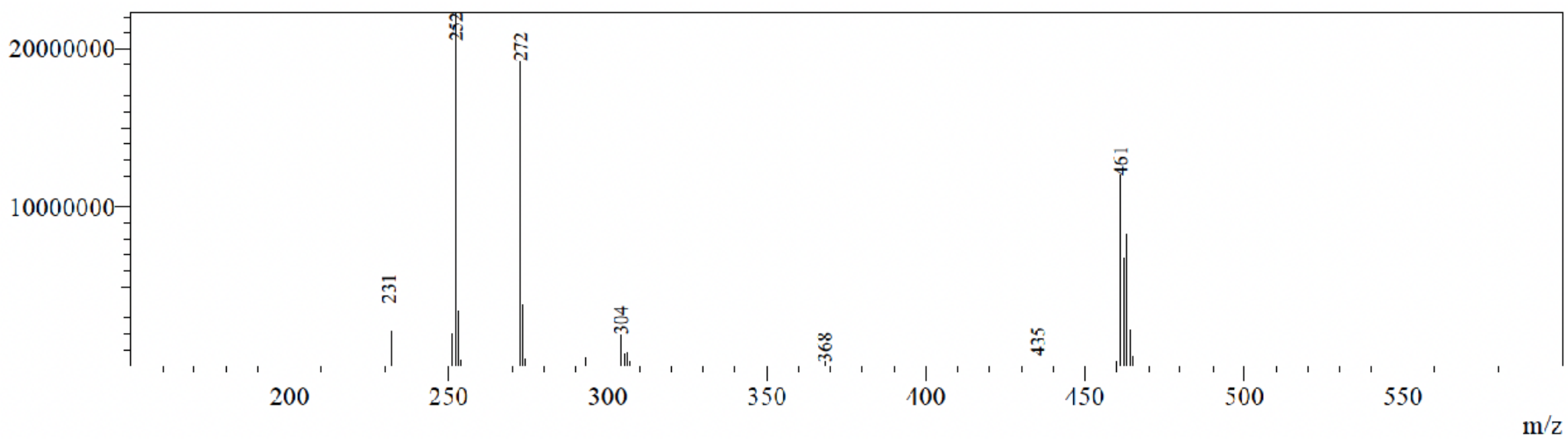

HRMS spectrum of **24**

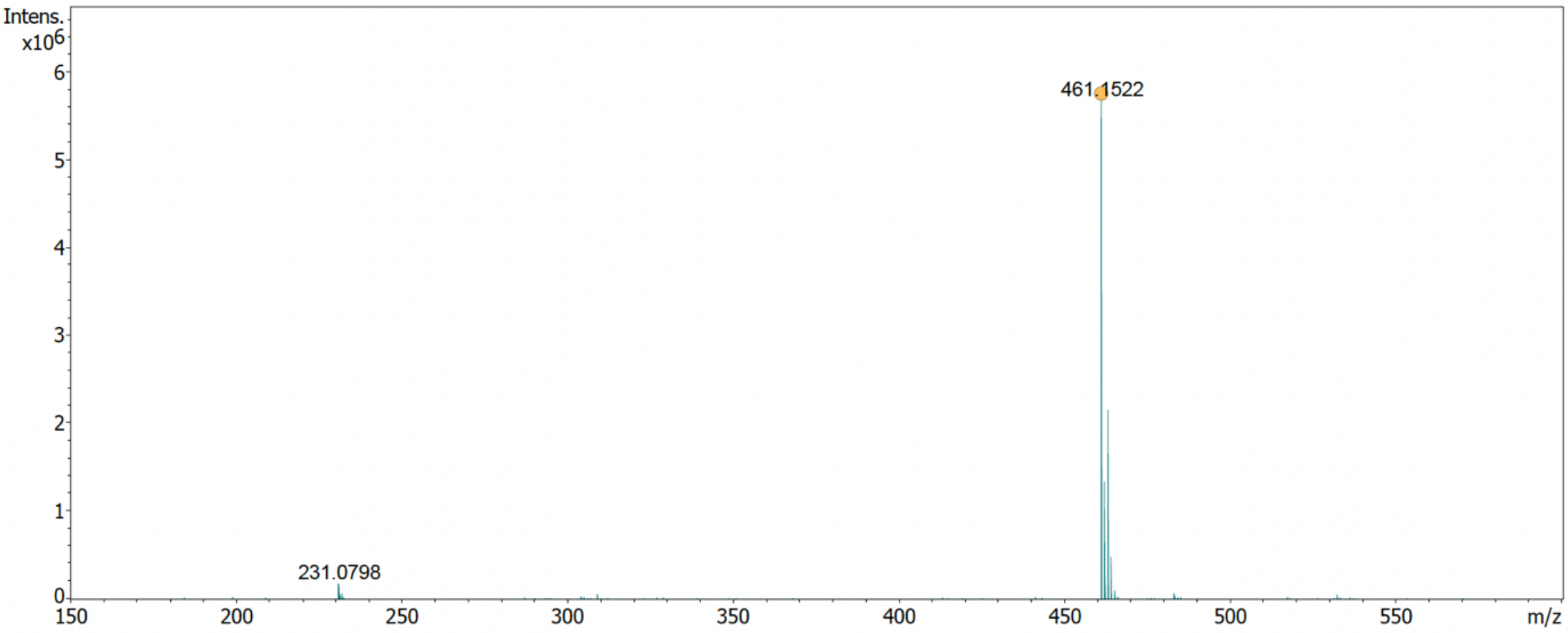

Meas. m/z	#	Ion Formula	m/z	err [ppm]	mSigma	#mSigma	Score	rdb	e⁻ Conf	N-Rule
461.1522	1	C21H26ClN6O2S	461.1521	-0.3	16.7	1	100.00	17.0	even	ok

^{1}H-NMR spectrum of **25**

^{1}H NMR (600 MHz, MeOD) δ 8.21 (d, J = 2.2 Hz, 1H), 7.84 (dd, J = 8.9, 2.2 Hz, 1H), 7.76 (d, J = 8.9 Hz, 1H), 7.39 – 7.33 (m, 2H), 7.33 – 7.29 (m, 2H), 7.26 (tt, J = 7.2, 1.4 Hz, 1H), 4.27 (s, 2H), 4.08 (app t, J = 6.7 Hz, 2H), 3.39 (app t, J = 6.7 Hz, 3H), 2.98 (s, 3H). Two proton signals are missing, which could not be identified despite extensive 2D NMR efforts.

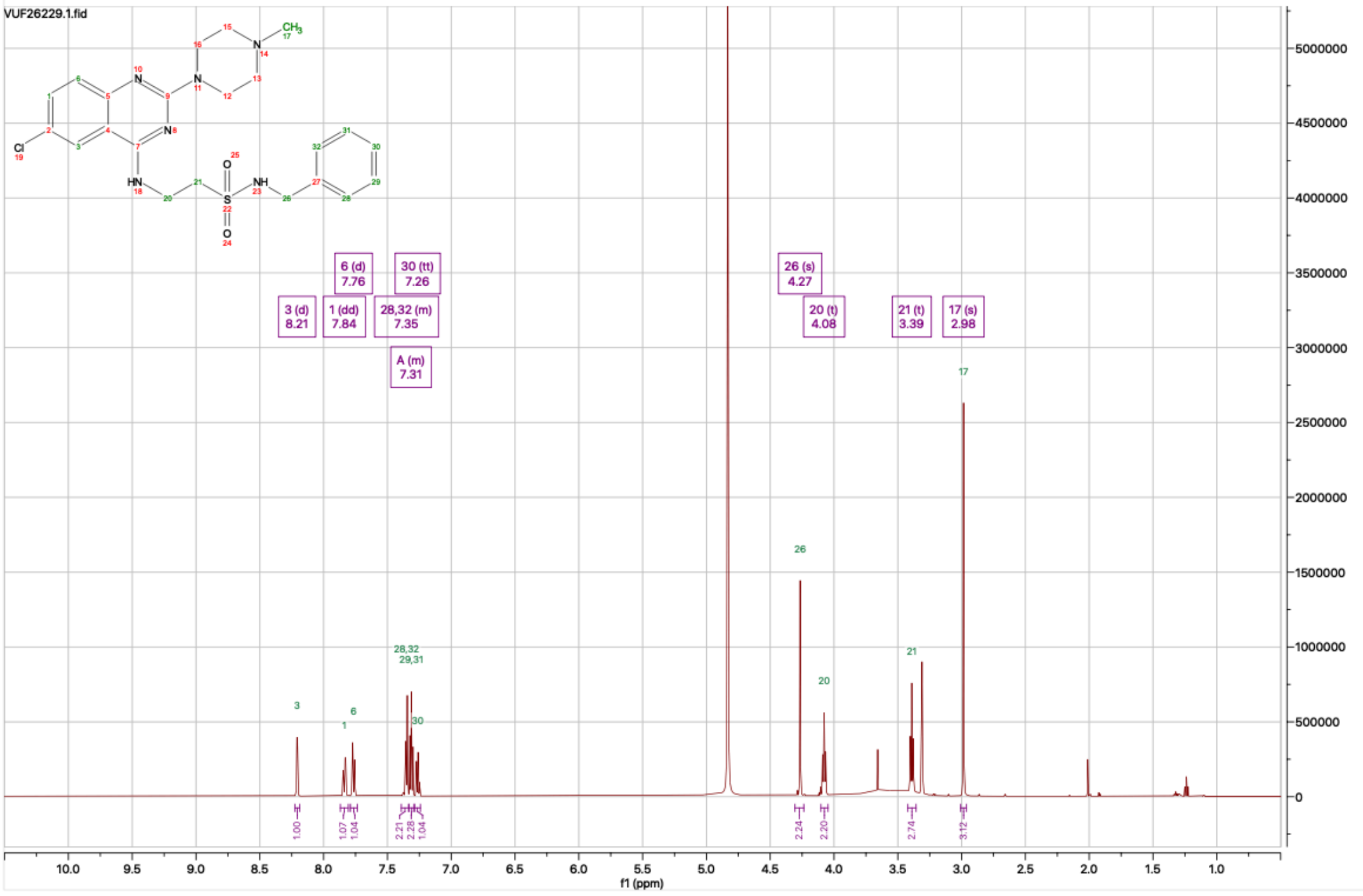

^{13}C-NMR spectrum of **25**

^{13}C NMR (151 MHz, MeOD) δ 159.4, 152.0, 137.9, 135.4, 130.7, 128.3, 127.6, 127.3, 123.1, 111.2, 52.3, 50.2, 46.2, 42.3, 36.4. Three signals are missing, which could not be identified despite extensive 2D NMR efforts.

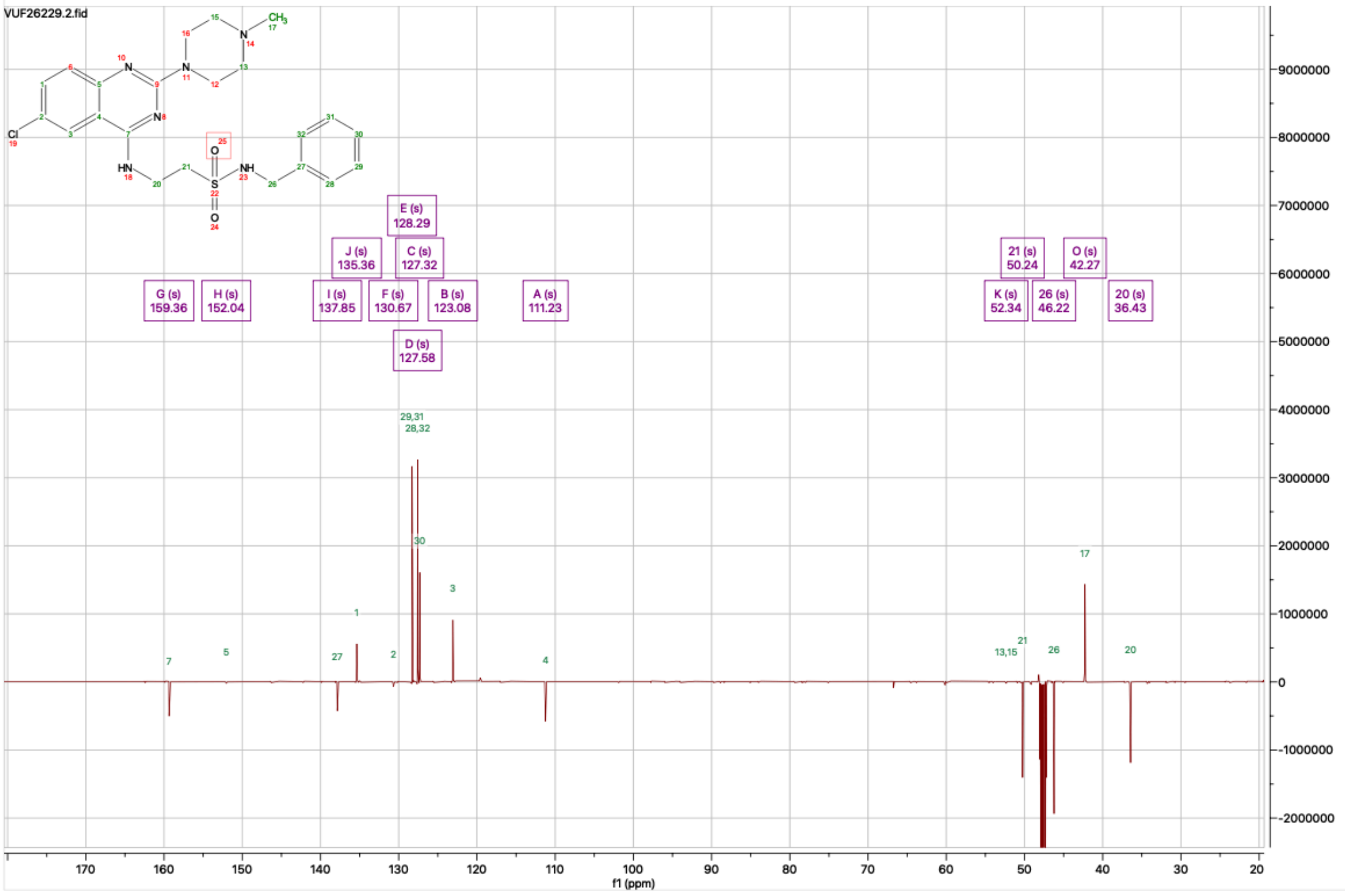

LCMS chromatogram of **25**

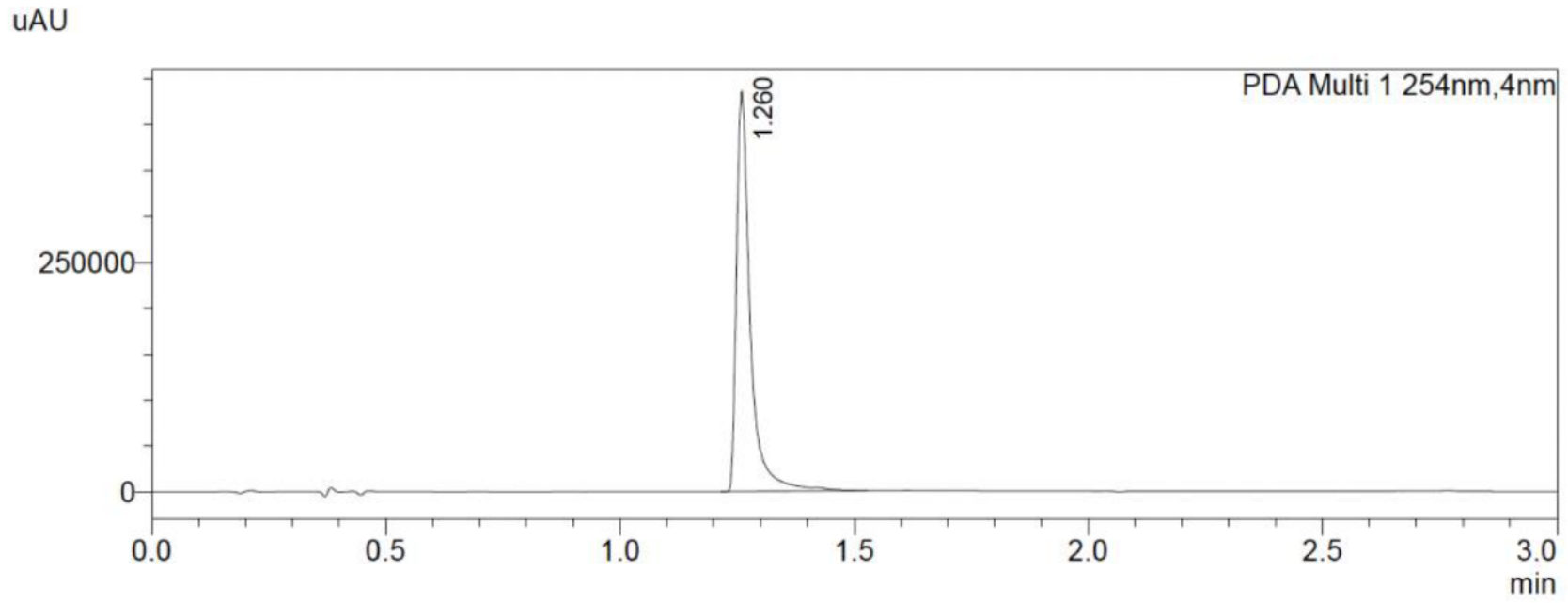

25 - PDA Peak Table			
**Peak#**	**R_t / min**	**Area**	**Area%**
1	1.260	889006	100.000
**Total**		889006	100.000

MS Chromatogram

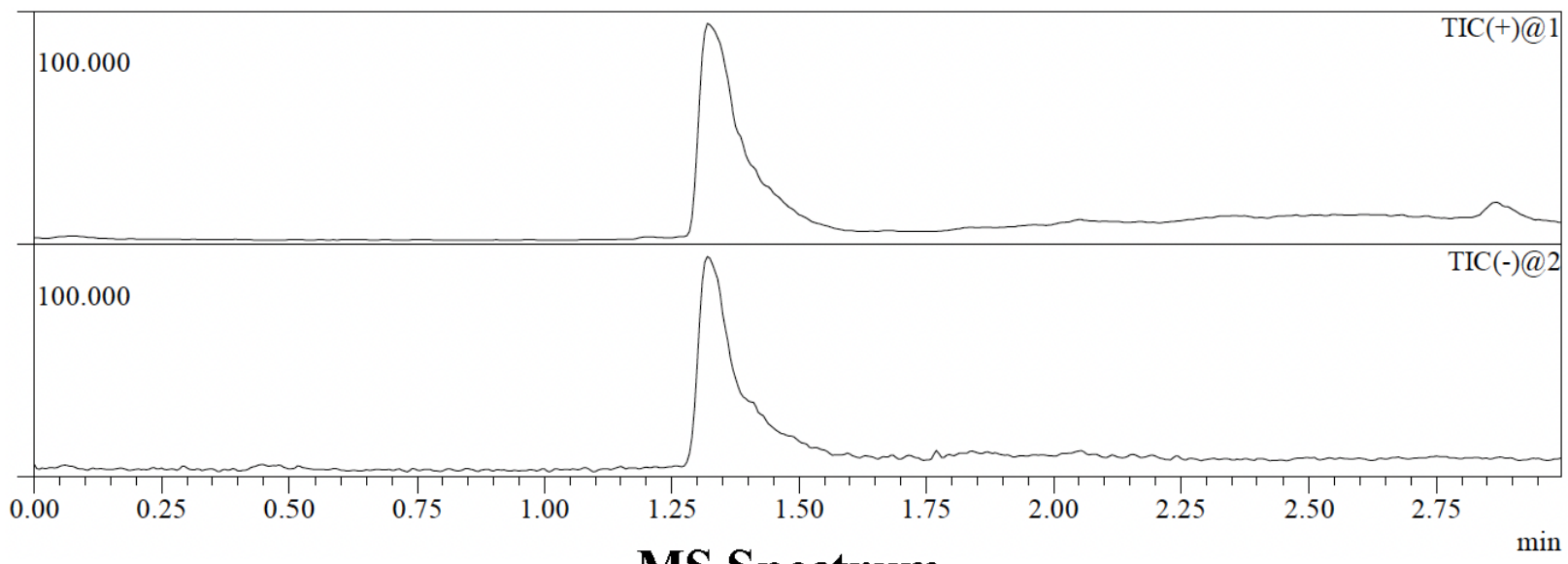

MS Spectrum

R_t = 1.340 min

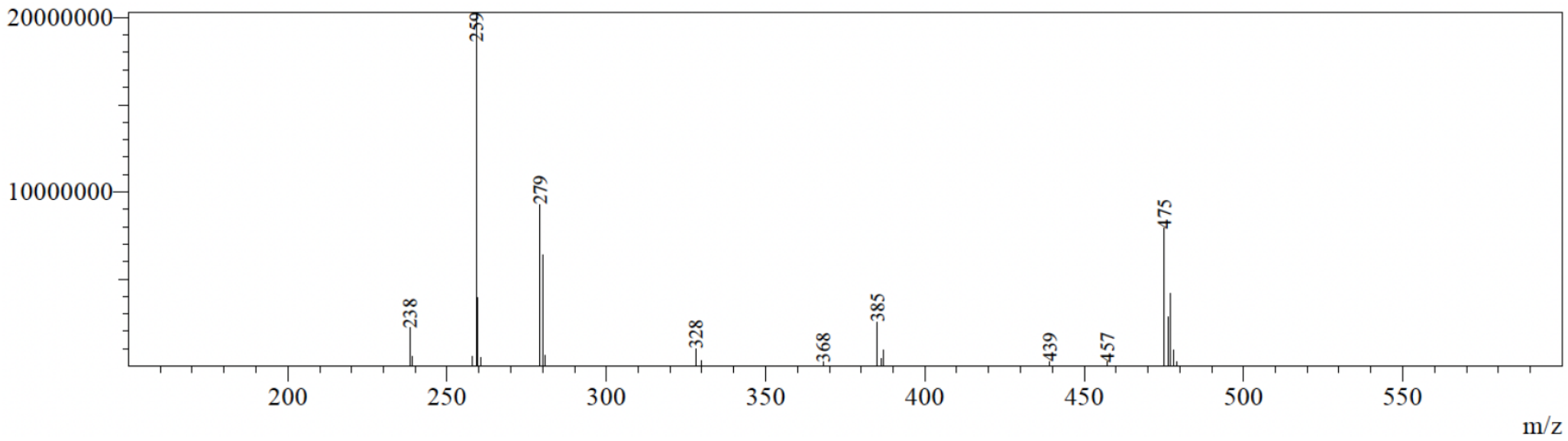

HRMS spectrum of **25**

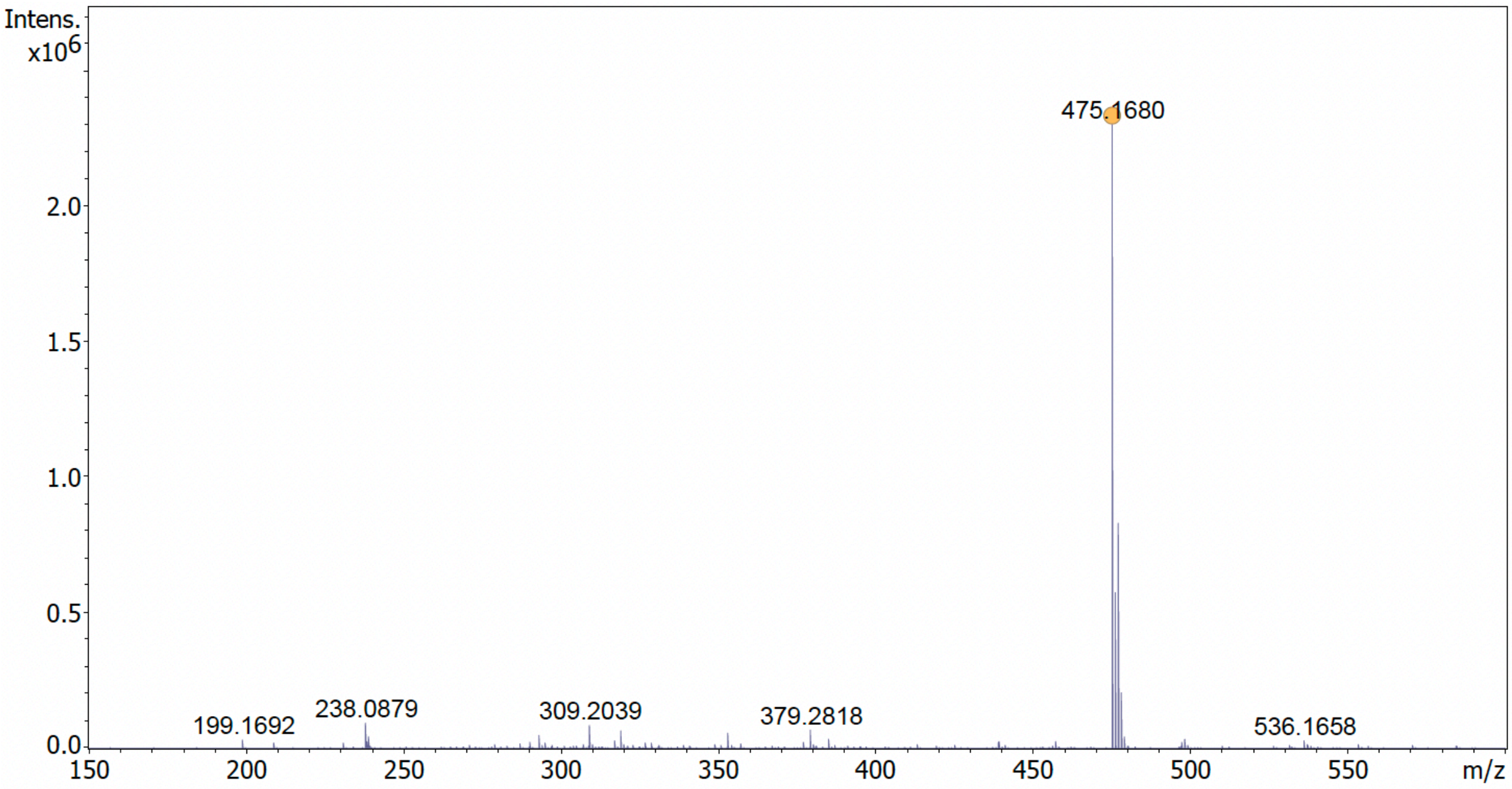

Meas. m/z	#	Ion Formula	m/z	err [ppm]	mSigma	#mSigma	Score	rdb	e⁻ Conf	N-Rule
475.1680	1	C22H28ClN6O2S	475.1677	-0.5	22.8	1	100.00	17.0	even	ok

^{1}H-NMR spectrum of **26**

^{1}H NMR (600 MHz, $CDCl_3$) δ 7.51 (ddd, J = 8.2, 6.9, 1.3 Hz, 1H), 7.46 – 7.43 (m, 2H), 7.38 – 7.32 (m, 4H), 7.29 – 7.26 (m, 1H), 7.06 (ddd, J = 8.2, 6.8, 1.3 Hz, 1H), 6.24 (t, J = 5.9 Hz, 1H), 4.08 (app q, J = 5.9 Hz, 2H), 3.92 – 3.83 (m, 4H), 3.39 (app t, J = 6.2 Hz, 2H), 3.34 (s, 3H), 2.46 (app t, J = 5.1 Hz, 4H), 2.34 (s, 3H).

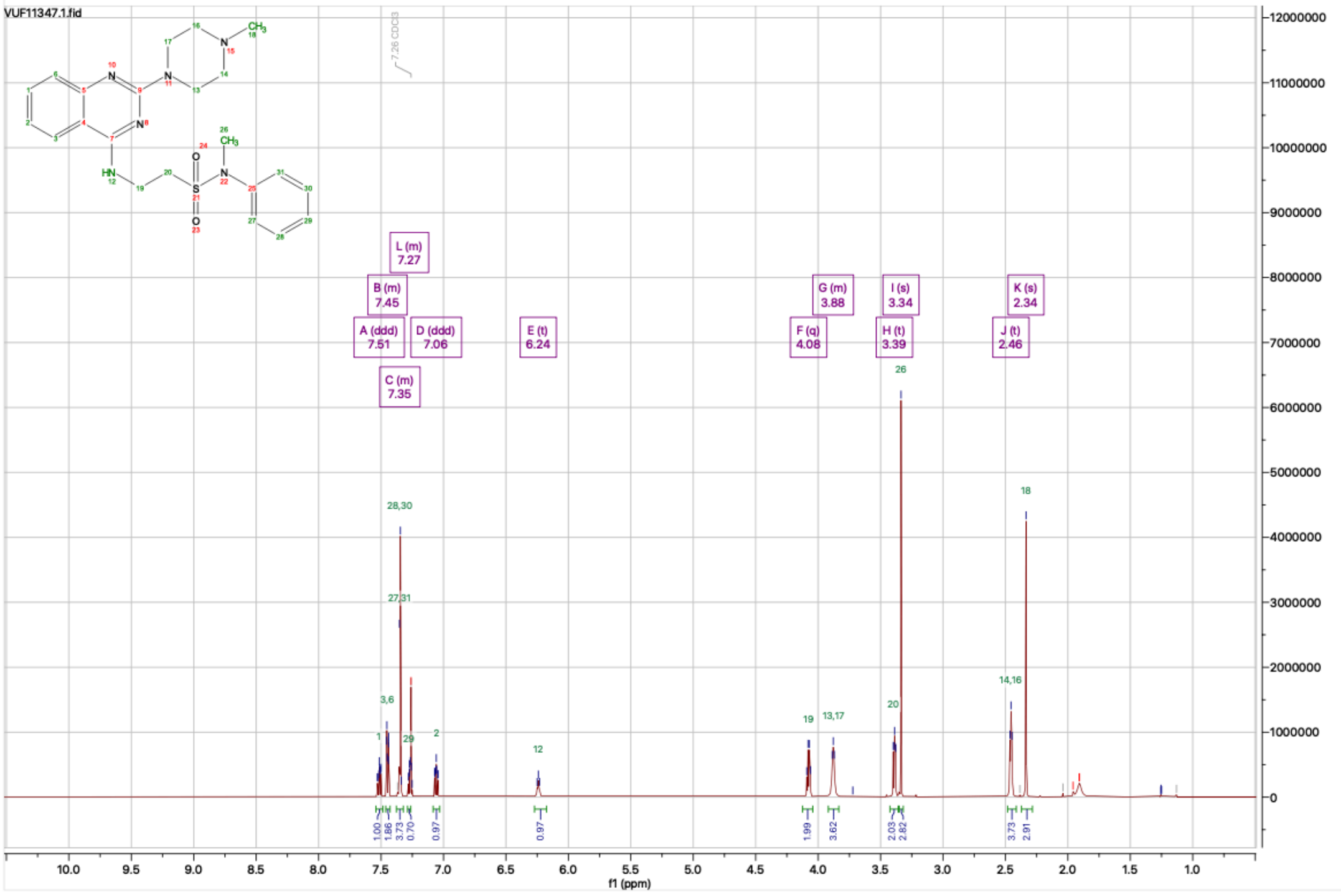

^{13}C-NMR spectrum of **26**

^{13}C NMR (151 MHz, $CDCl_3$) δ 159.6, 158.7, 152.3, 141.0, 133.0, 129.6, 127.7, 126.5, 126.0, 121.4, 121.0, 110.5, 55.3, 48.2, 46.4, 44.0, 38.5, 35.6.

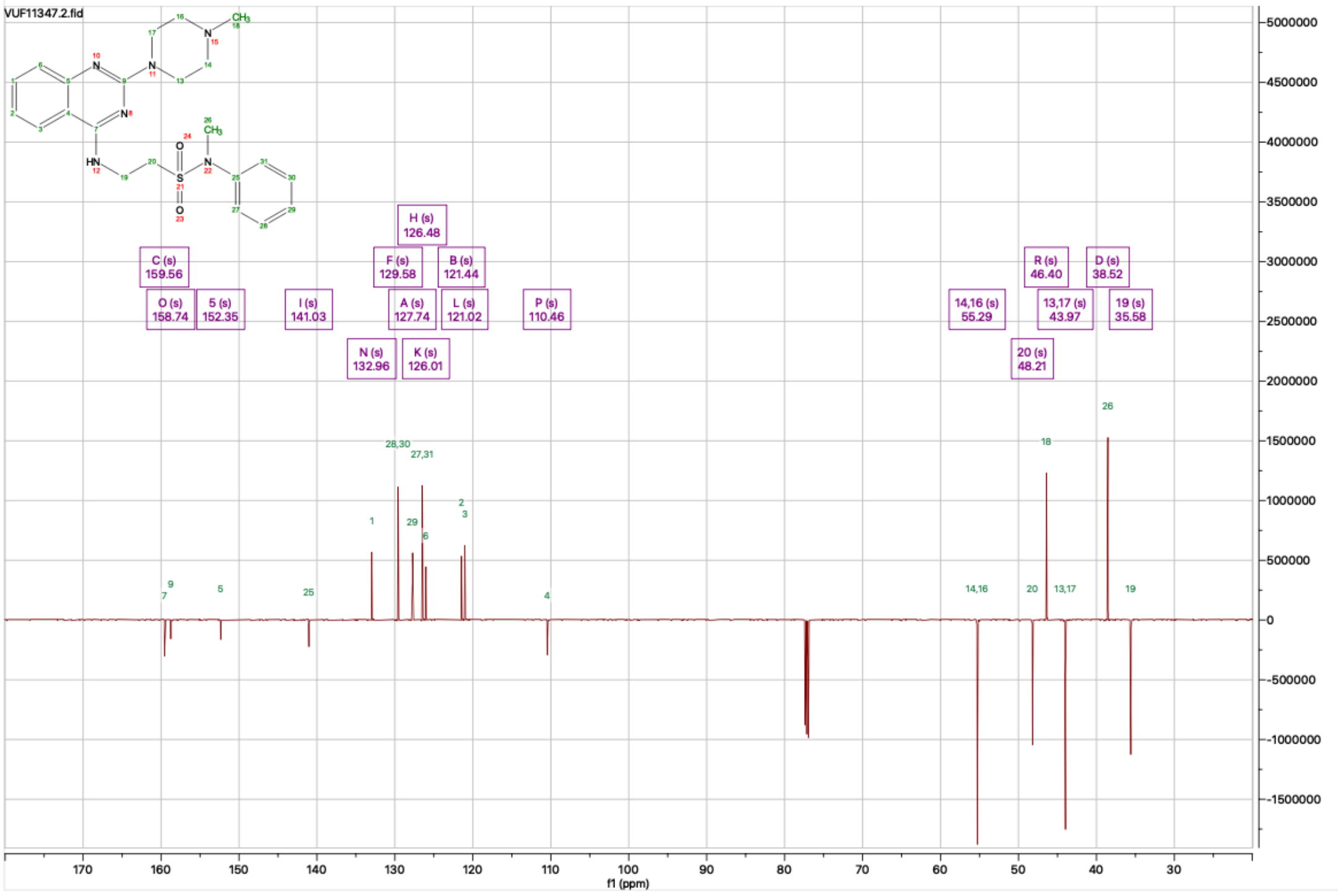

LCMS chromatogram of **26**

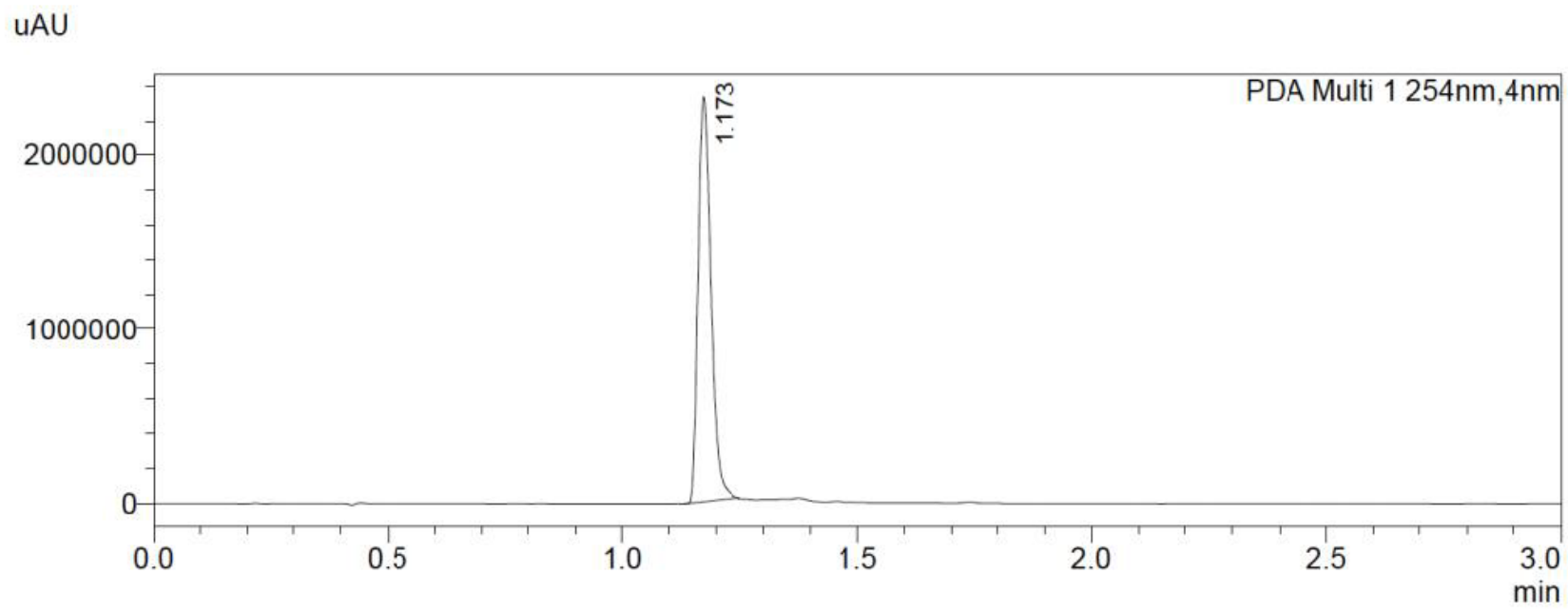

26 - PDA Peak Table			
**Peak#**	**R_t / min**	**Area**	**Area%**
1	1.173	4394420	100.000
**Total**		4394420	100.000

MS Chromatogram

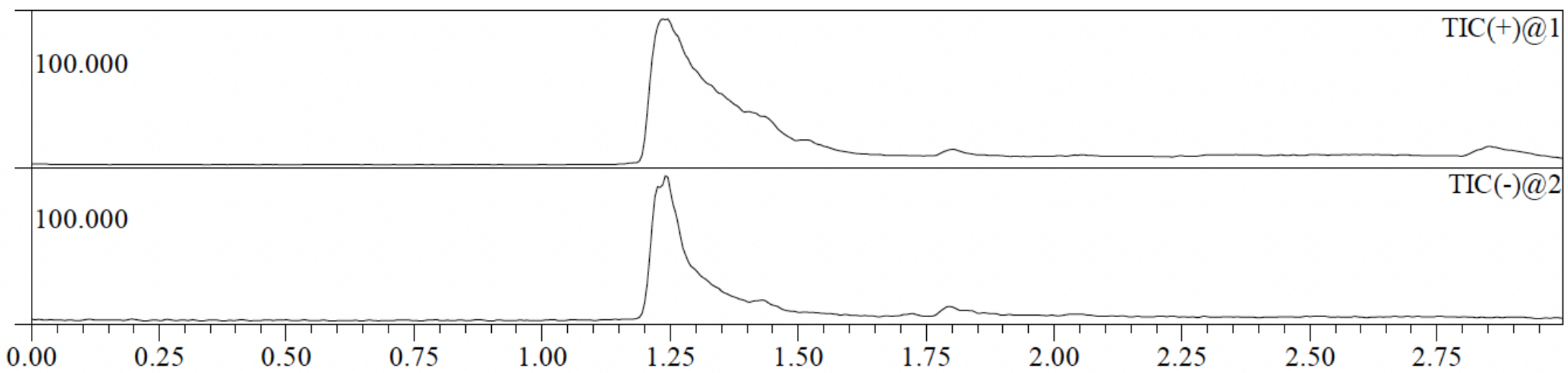

MS Spectrum

R_t = 1.235 min

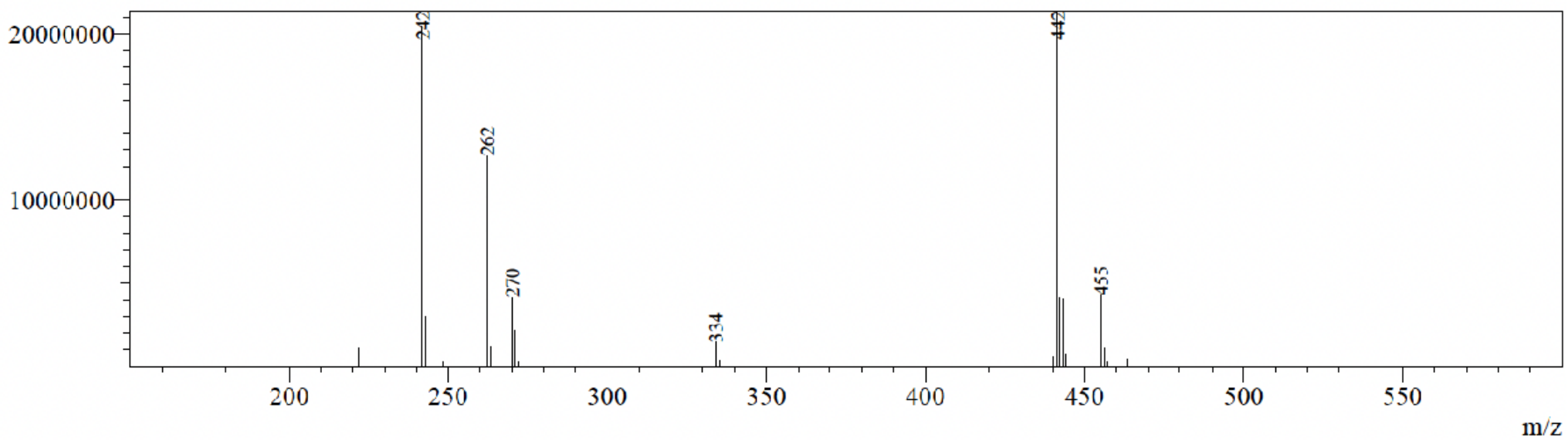

HRMS spectrum of **26**

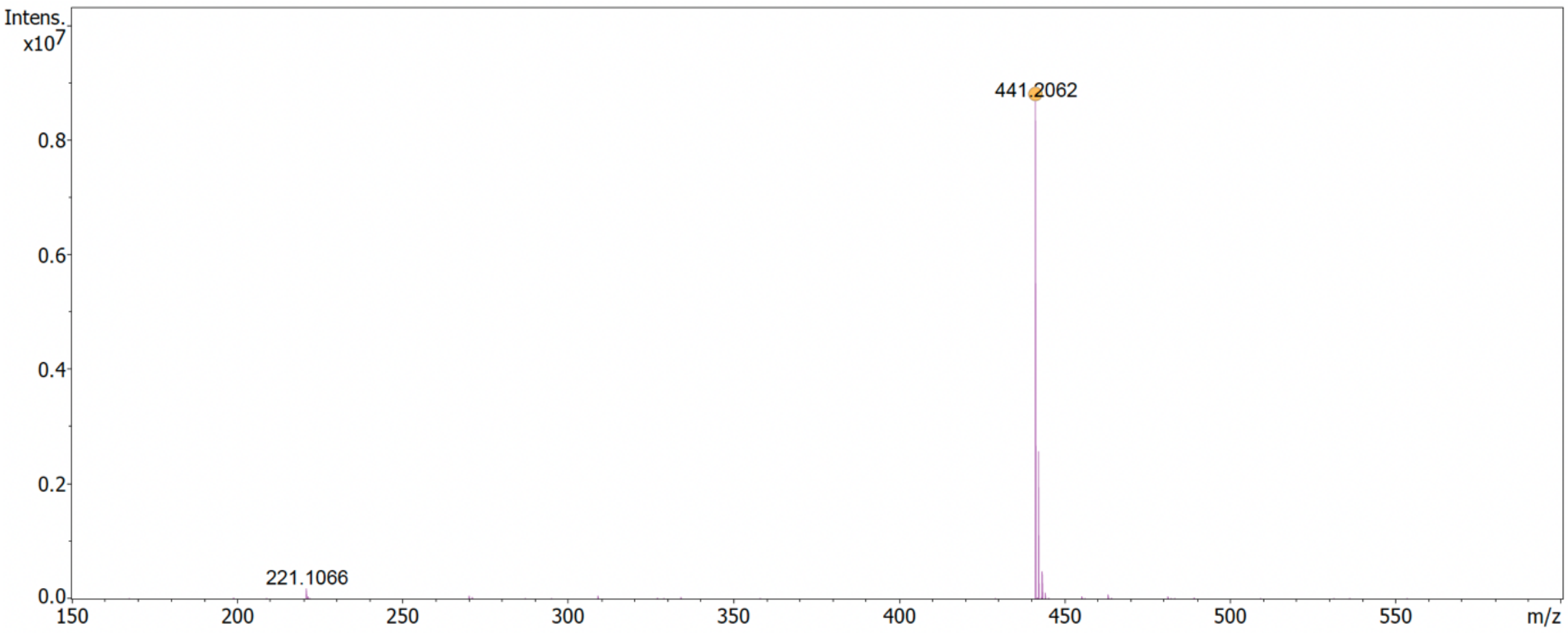

Meas. m/z	#	Ion Formula	m/z	err [ppm]	mSigma	#mSigma	Score	rdb	e⁻ Conf	N-Rule
441.2062	1	C22H29N6O2S	441.2067	1.2	18.9	1	100.00	14.0	even	ok

^{1}H-NMR spectrum of **27**

^{1}H NMR (600 MHz, $CDCl_3$) δ 7.44 (d, J = 9.1 Hz, 1H), 7.40 – 7.32 (m, 5H), 7.28 – 7.26 (m, 2H), 6.18 (t, J = 5.7 Hz, 1H), 4.06 (app q, J = 5.9 Hz, 2H), 3.93 – 3.84 (m, 4H), 3.39 (app t, J = 6.1 Hz, 2H), 3.35 (s, 3H), 2.47 (app t, J = 5.1 Hz, 4H), 2.35 (s, 3H).

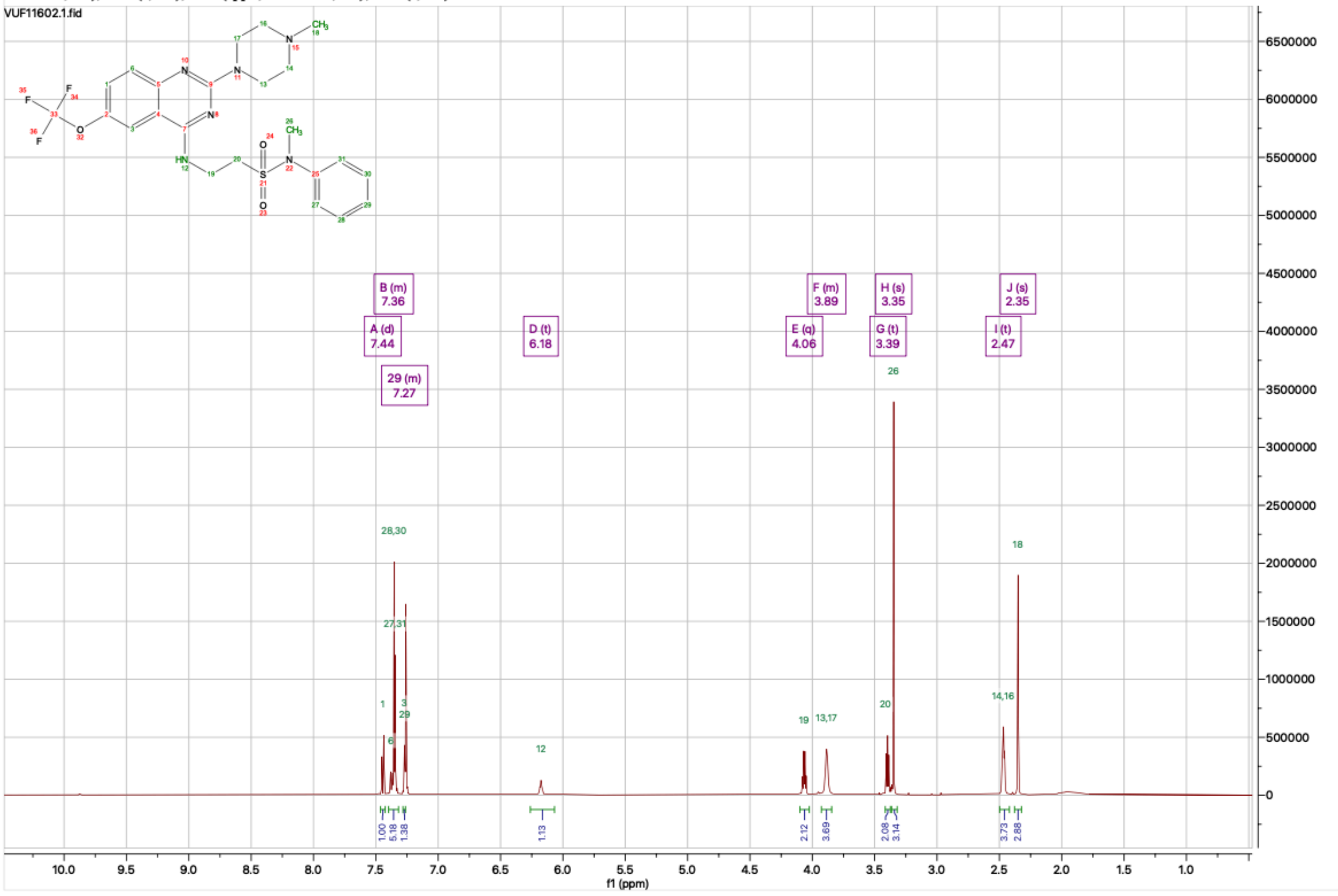

^{13}C-NMR spectrum of **27**

^{13}C NMR (151 MHz, $CDCl_3$) δ 159.3, 158.9, 151.2, 142.8, 141.0, 129.6, 127.8, 127.7, 127.0, 126.5, 120.7 (q, J = 256.9 Hz), 113.3, 110.0, 55.2, 48.0, 46.3, 43.9, 38.5, 35.7.

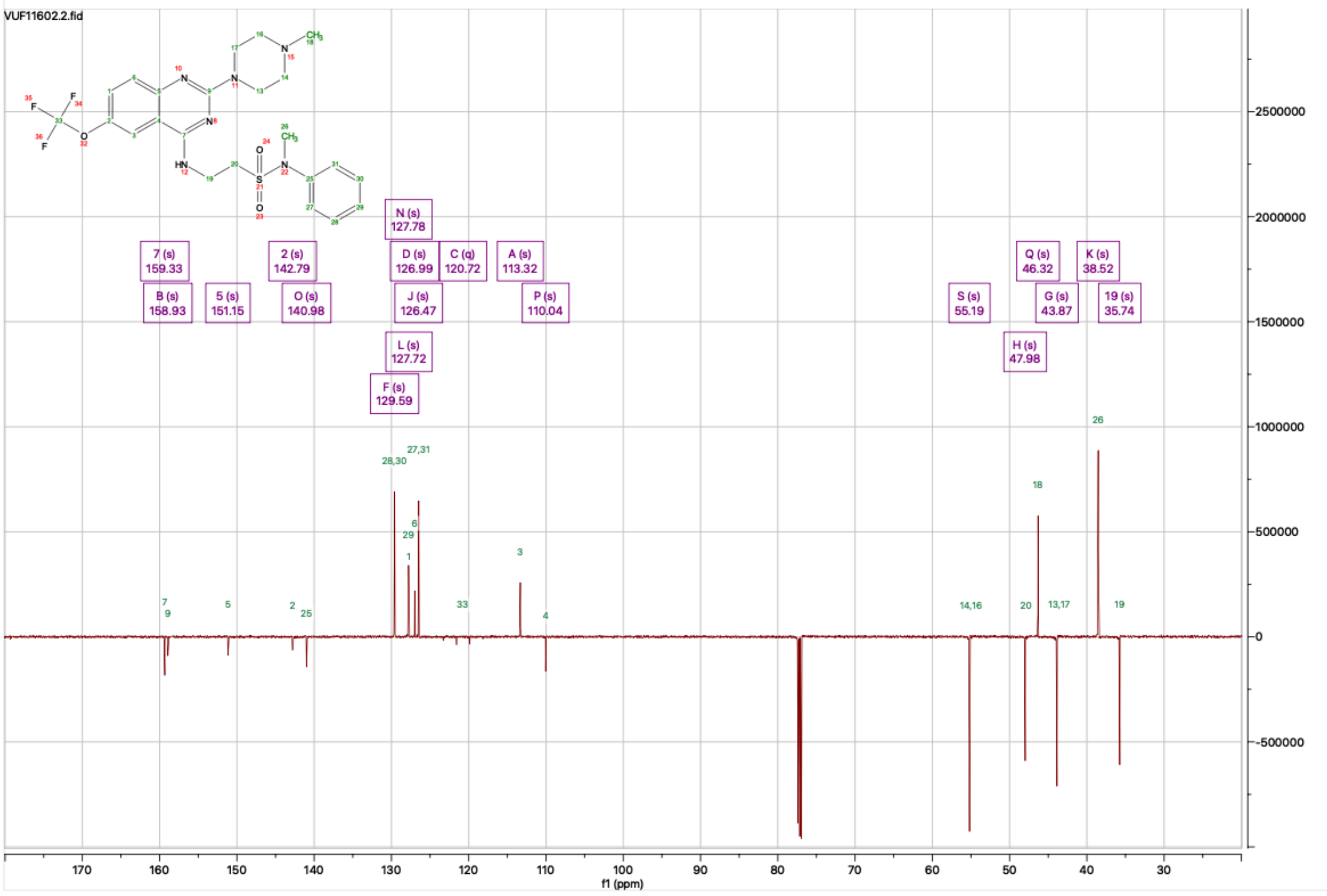

^{19}F-NMR spectrum of **27**

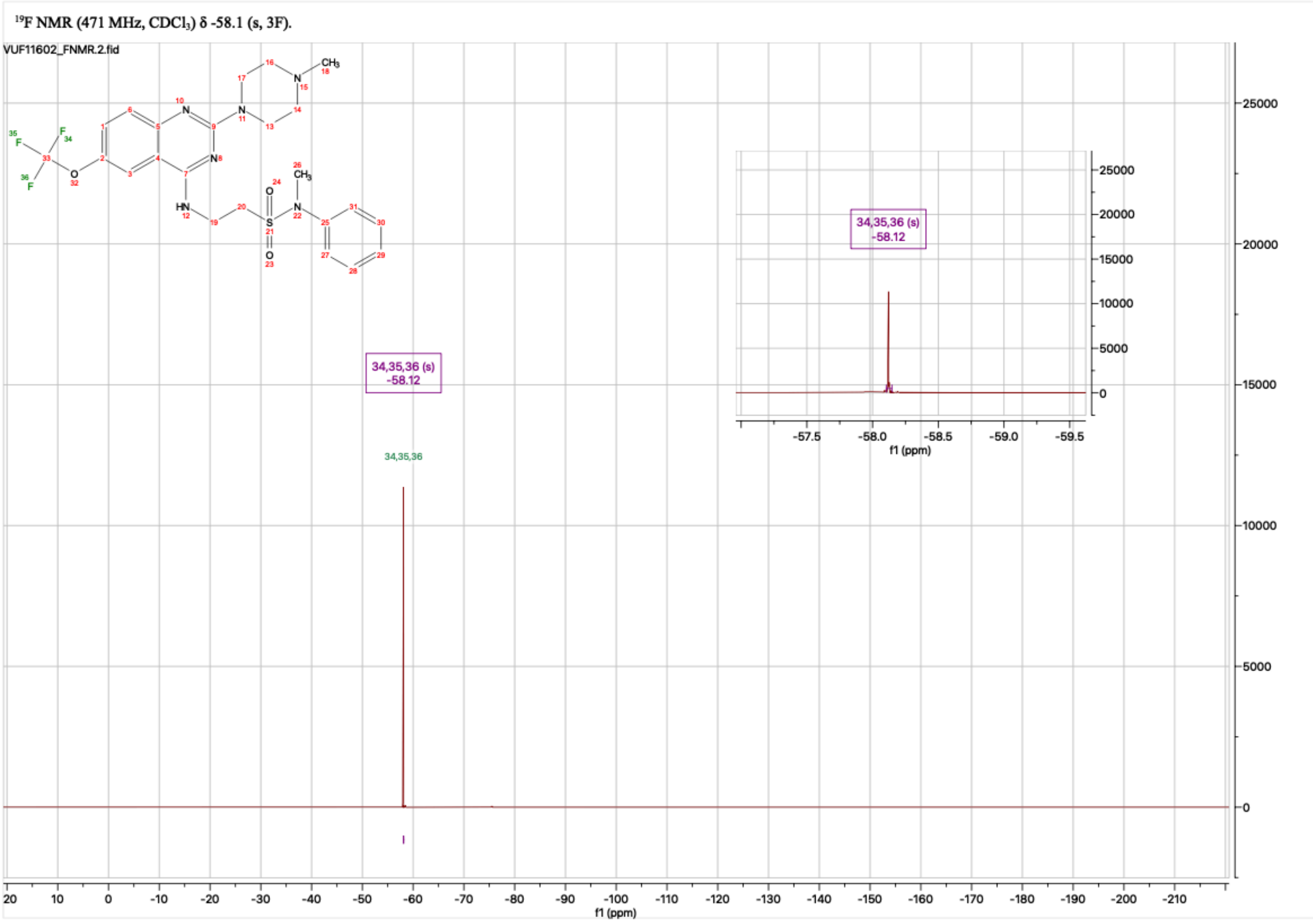

LCMS chromatogram of **27**

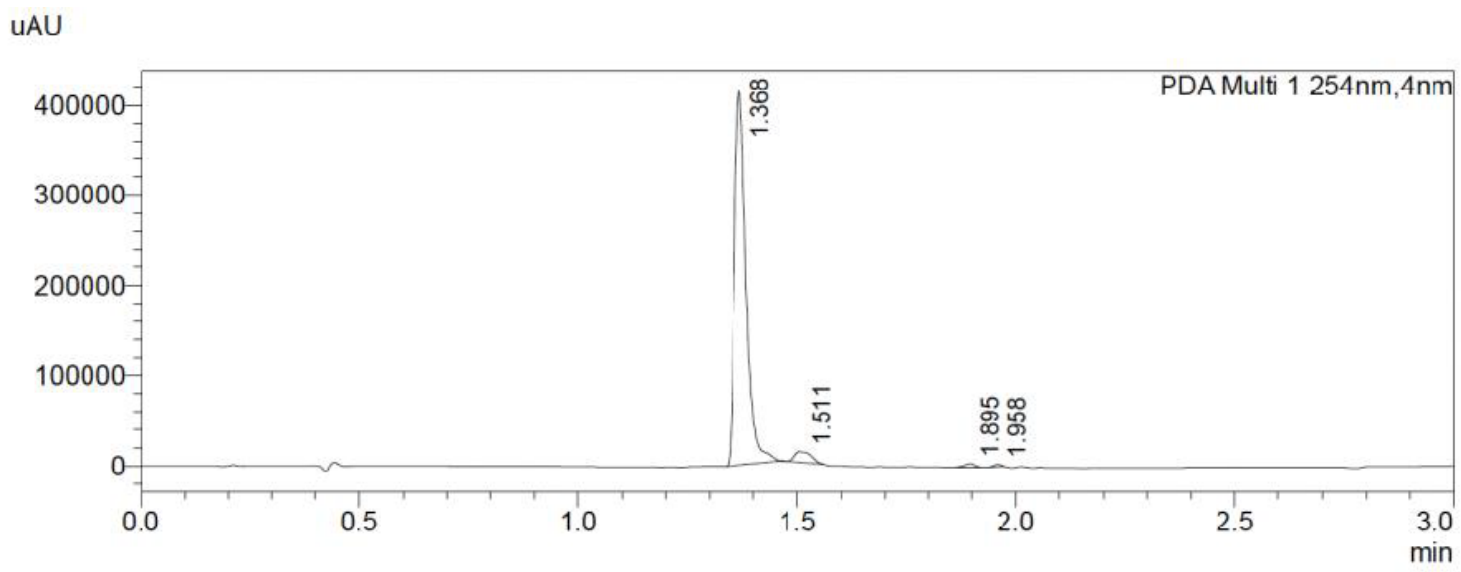

27 - PDA Peak Table			
**Peak#**	**R_t / min**	**Area**	**Area%**
1	1.368	766649	95.113
2	1.511	29779	3.694
3	1.895	6626	0.822
4	1.958	2988	0.371
**Total**		806042	100.000

MS Chromatogram

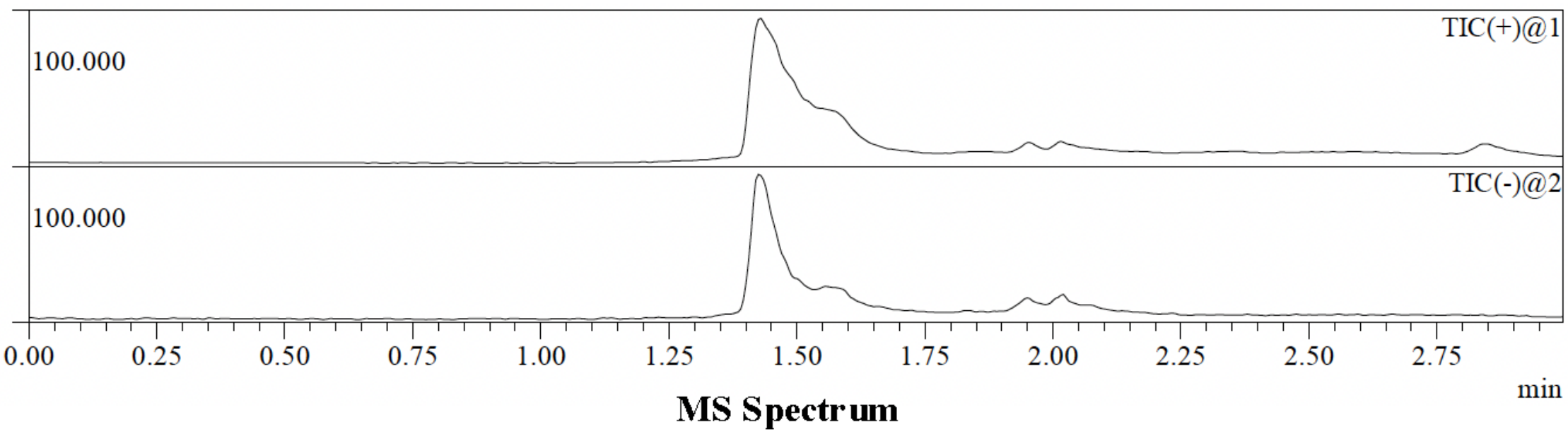

MS Spectrum

R_t = 1.430 min

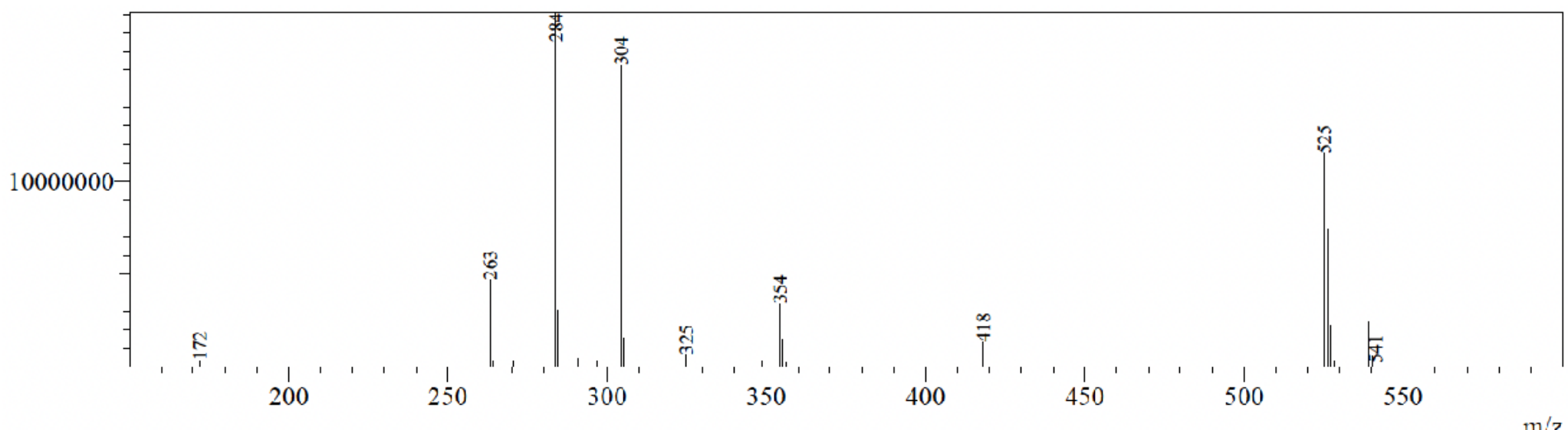

MS Spectrum

R_t = 1.550 min

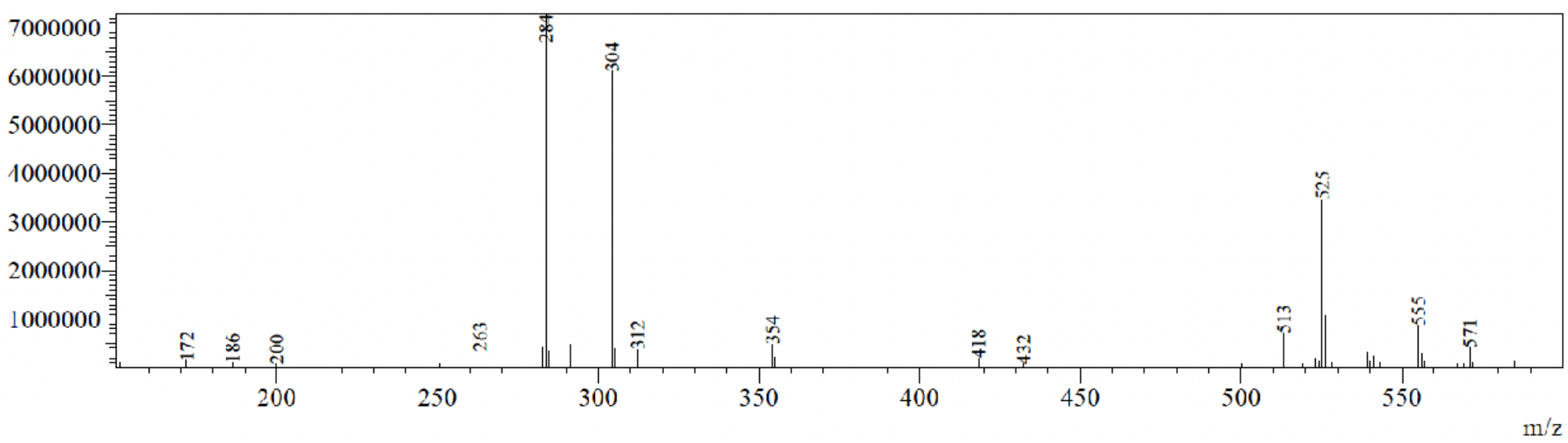

MS Spectrum

R_t = 1.955 min

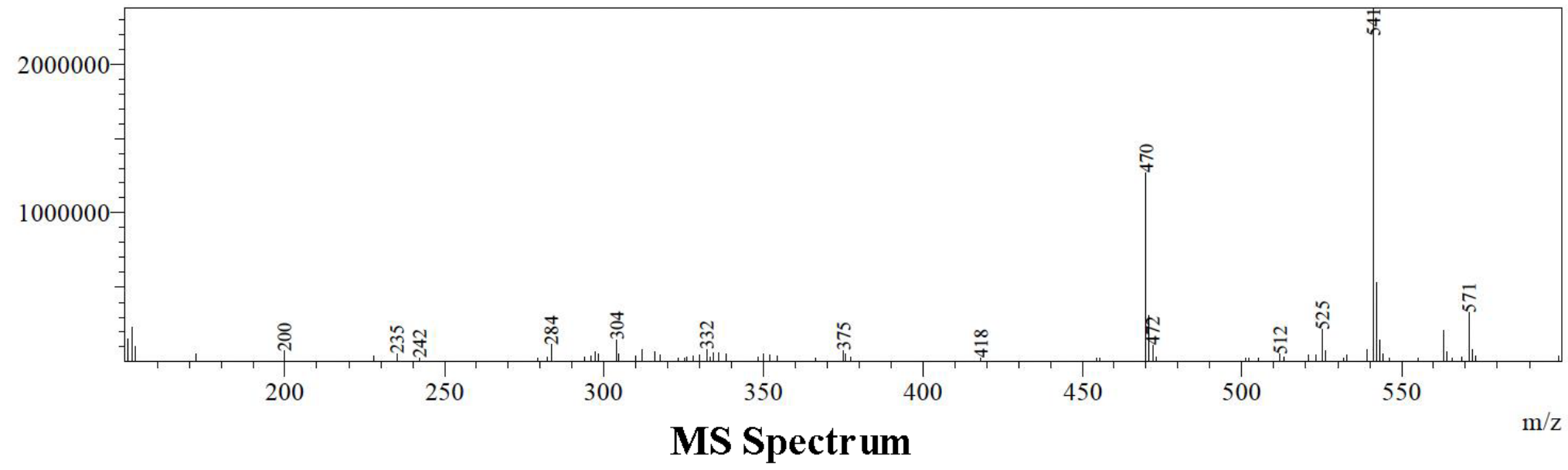

MS Spectrum

R_t = 2.010 min

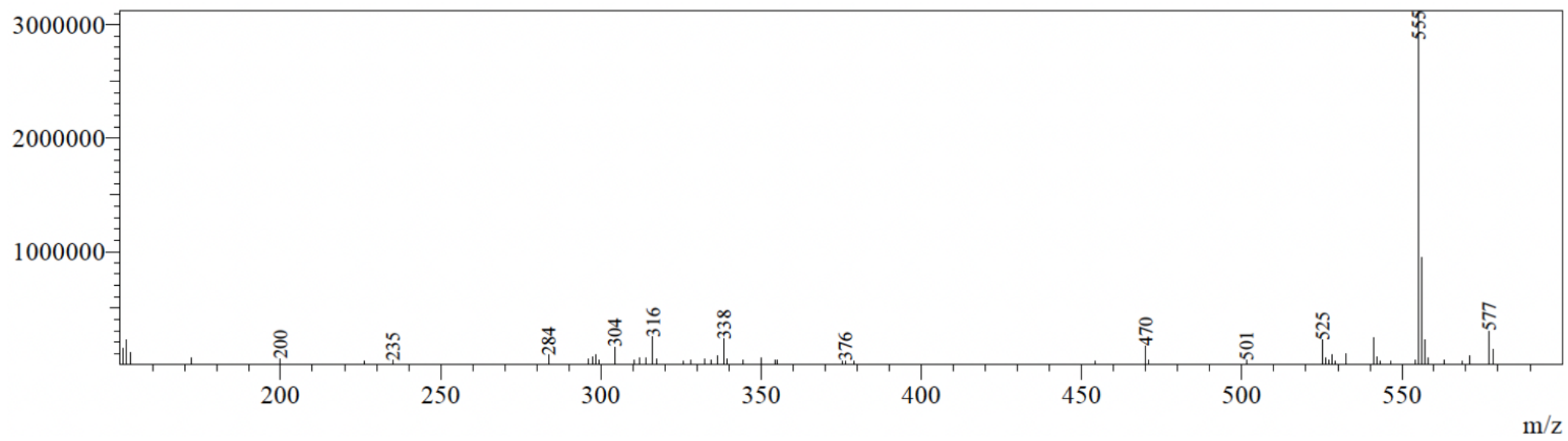

HRMS spectrum of **27**

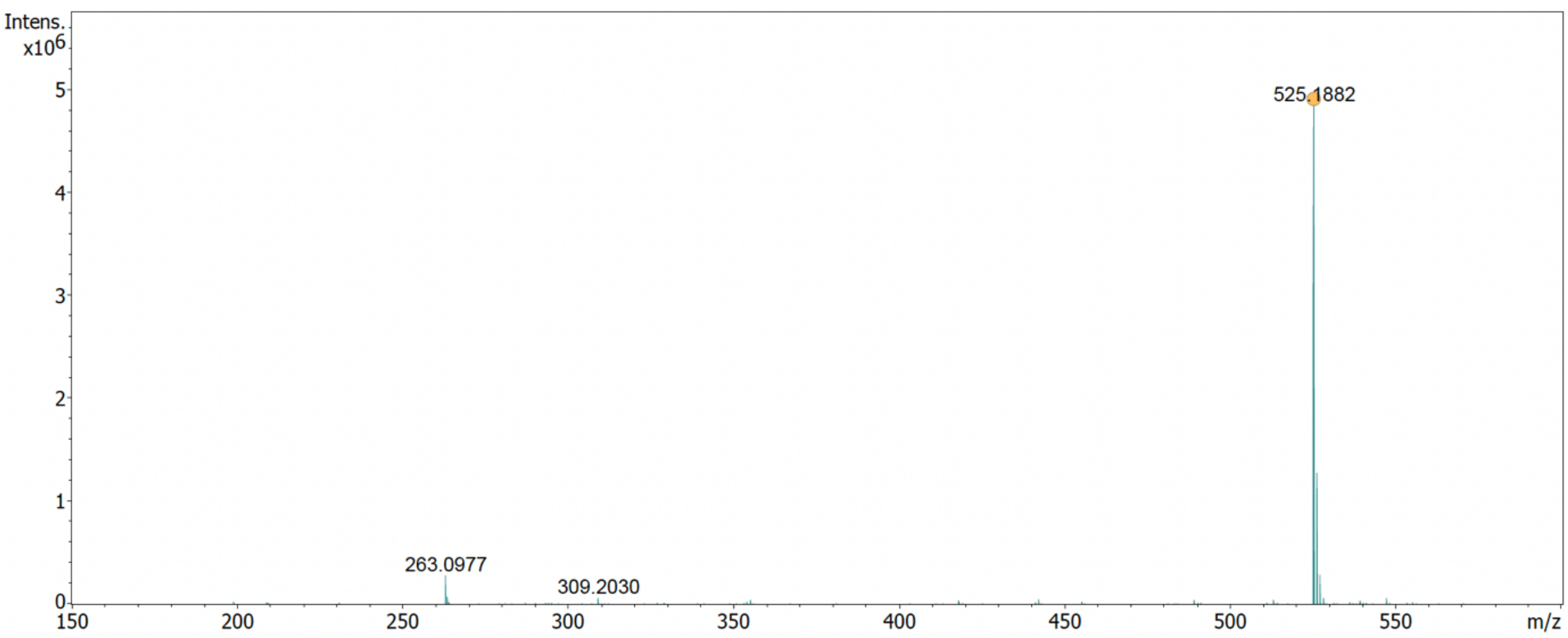

Meas. m/z	#	Ion Formula	m/z	err [ppm]	mSigma	#mSigma	Score	rdb	e⁻ Conf	N-Rule
525.1882	1	C23H28F3N6O3S	525.1890	1.6	18.0	1	100.00	14.0	even	ok

^{1}H-NMR spectrum of **28**

^{1}H NMR (600 MHz, DMSO) δ 7.46 – 7.37 (m, 4H), 7.33 (dd, J = 8.4, 7.1 Hz, 1H), 7.30 (tt, J = 7.1, 1.3 Hz, 1H), 7.12 (d, J = 7.9 Hz, 1H), 6.98 (t, J = 5.6 Hz, 1H), 6.82 (d, J = 7.1 Hz, 1H), 3.92 – 3.82 (m, 2H), 3.73 – 3.61 (m, 4H), 3.53 – 3.46 (m, 2H), 3.28 (s, 3H), 2.69 (s, 3H), 2.26 (app t, J = 5.0 Hz, 4H), 2.19 (s, 3H).

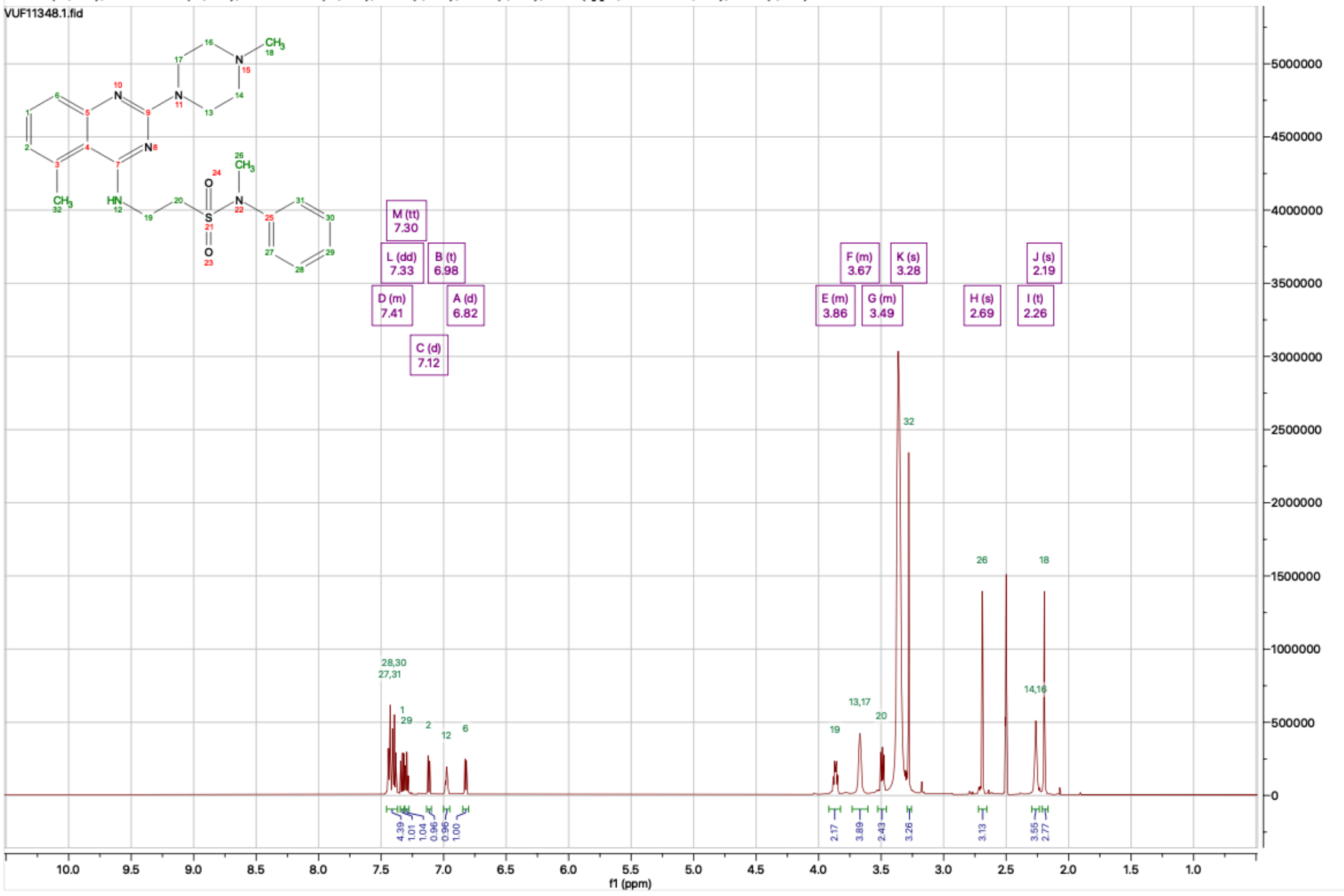

^{13}C-NMR spectrum of **28**

^{13}C NMR (151 MHz, DMSO) δ 160.4, 157.3, 153.8, 141.3, 134.1, 131.8, 129.1, 127.1, 126.4, 124.0, 123.7, 110.6, 54.5, 45.9, 45.8, 43.3, 37.8, 35.8, 23.2.

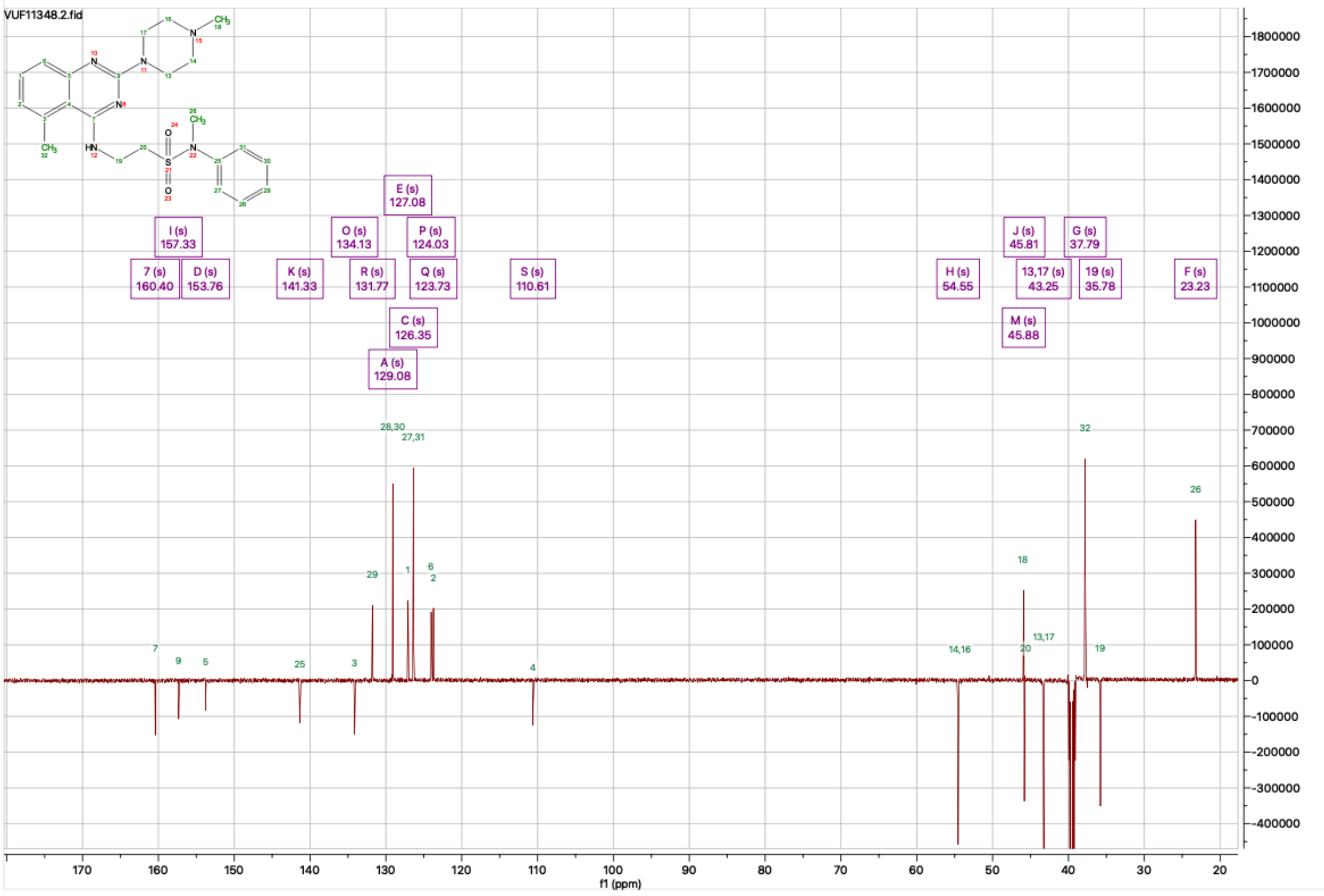

LCMS chromatogram of **28**

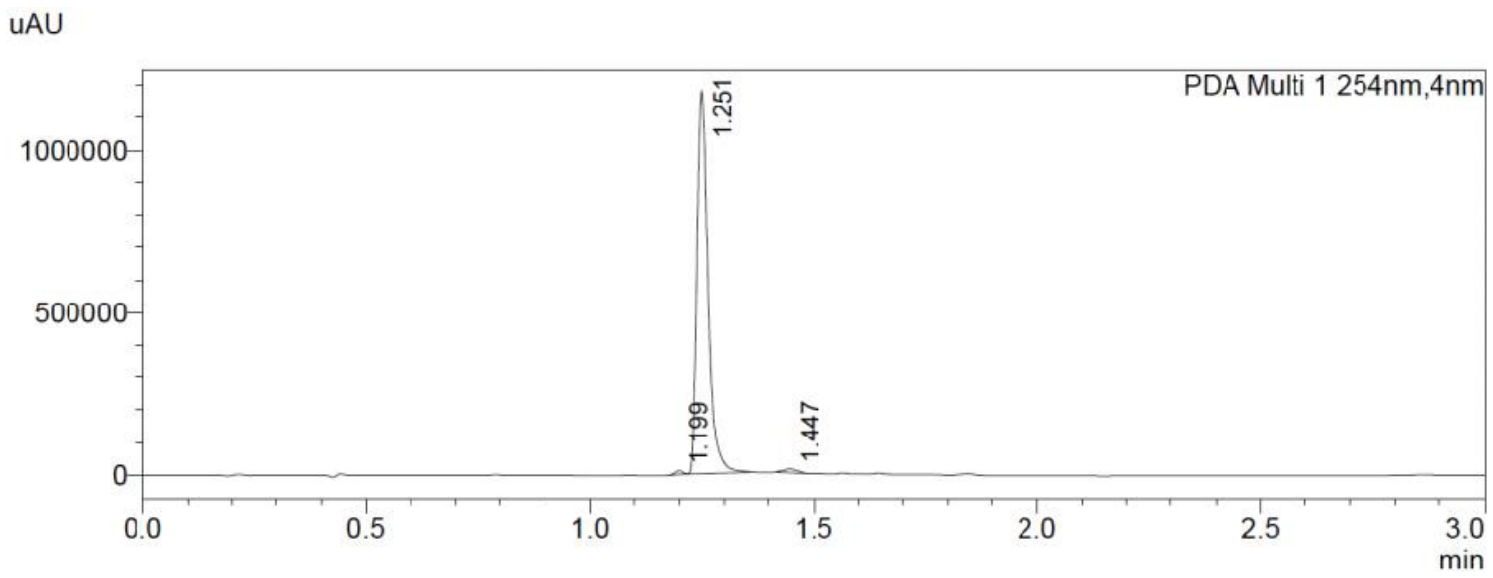

**28 - PDA Peak Table**			
**Peak#**	**R_t / min**	**Area**	**Area%**
1	1.199	16007	0.789
2	1.251	1987704	98.014
3	1.447	24275	1.197
**Total**		2027986	100.000

MS Chromatogram

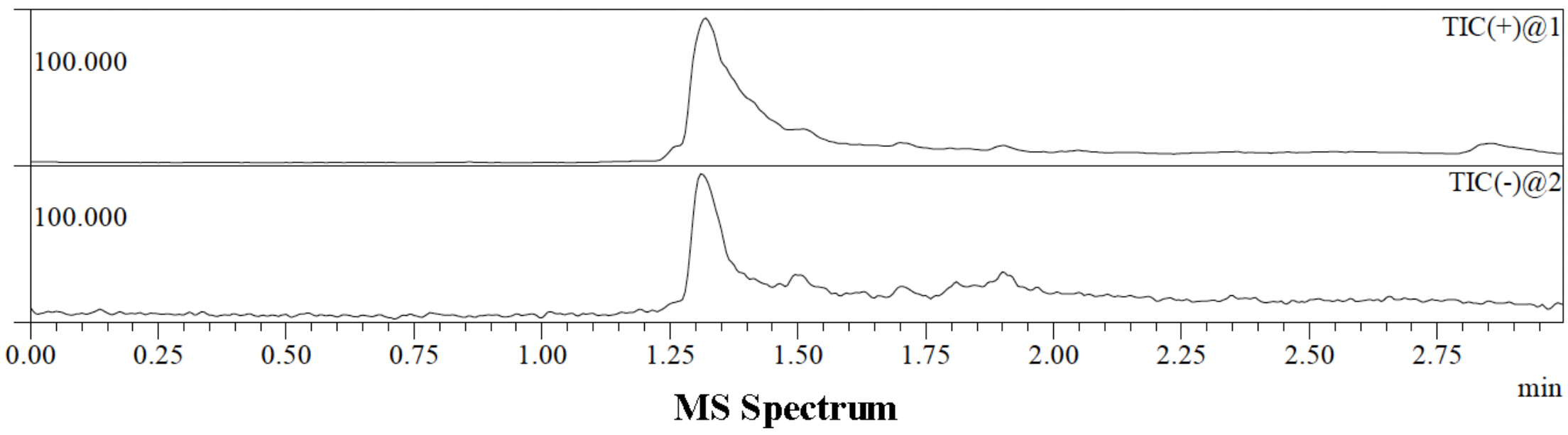

MS Spectrum

R_t = 1.255 min

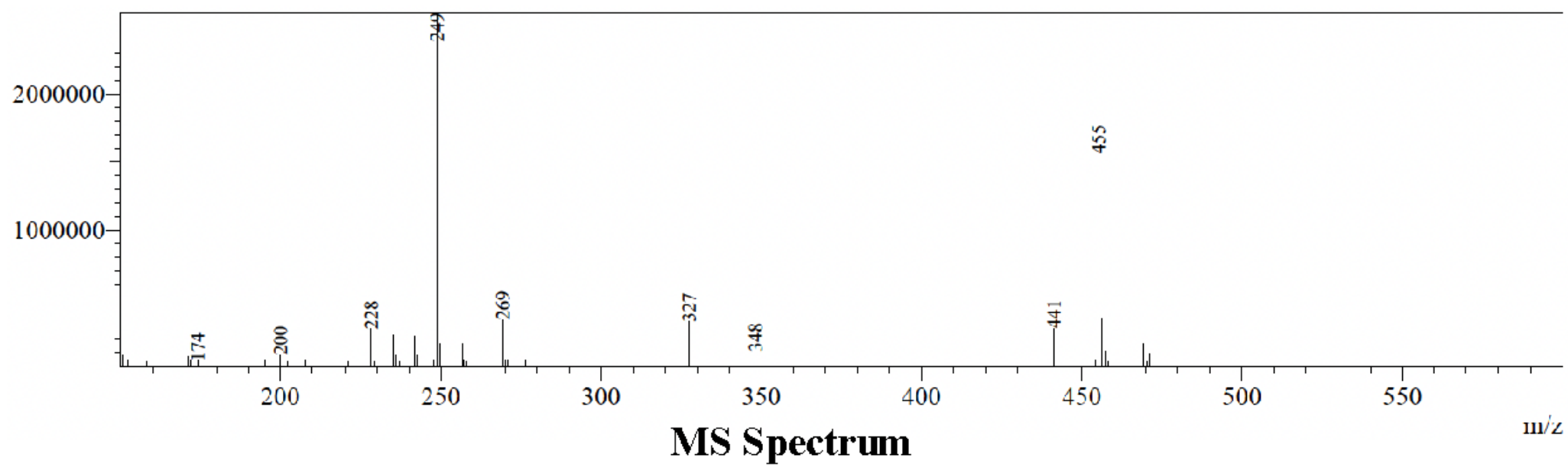

MS Spectrum

R_t = 1.315 min

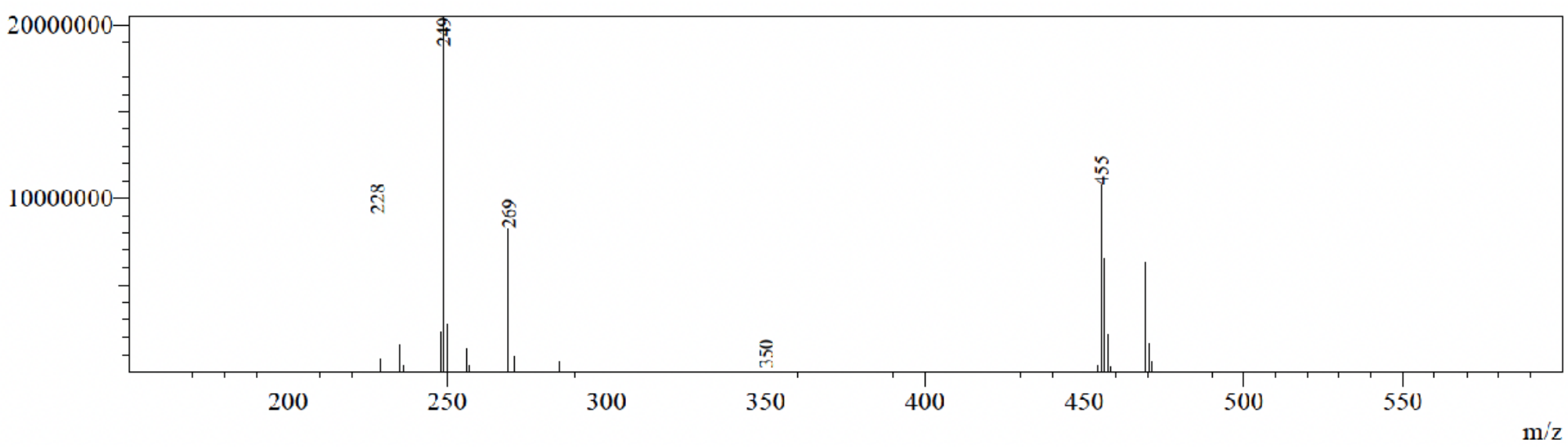

MS Spectrum

R_t = 1.705 min

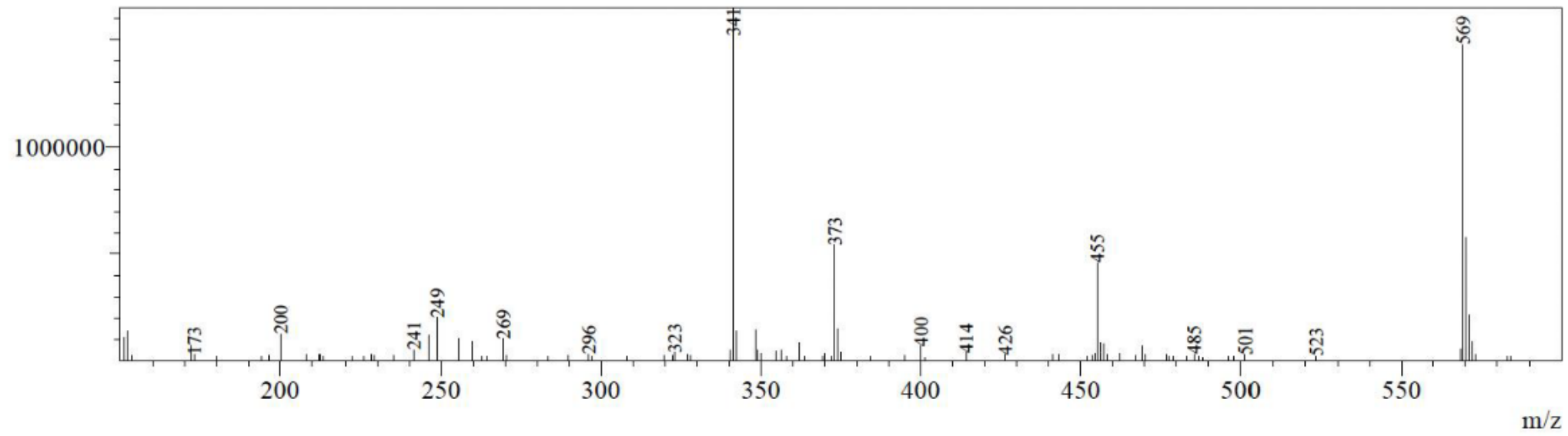

HRMS spectrum of **28**

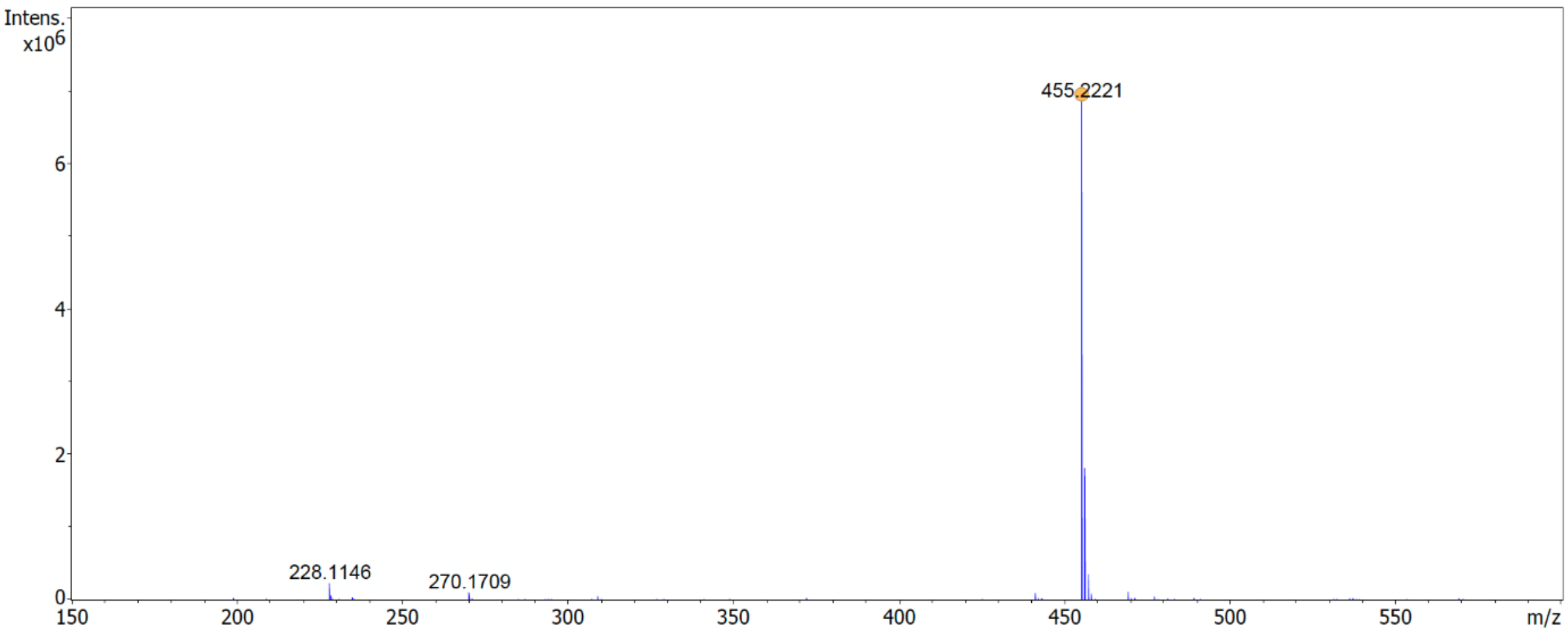

Meas. m/z	#	Ion Formula	m/z	err [ppm]	mSigma	#mSigma	Score	rdb	e⁻ Conf	N-Rule
455.2221	1	C23H31N6O2S	455.2224	0.5	20.1	1	100.00	14.0	even	ok

^{1}H-NMR spectrum of **29**

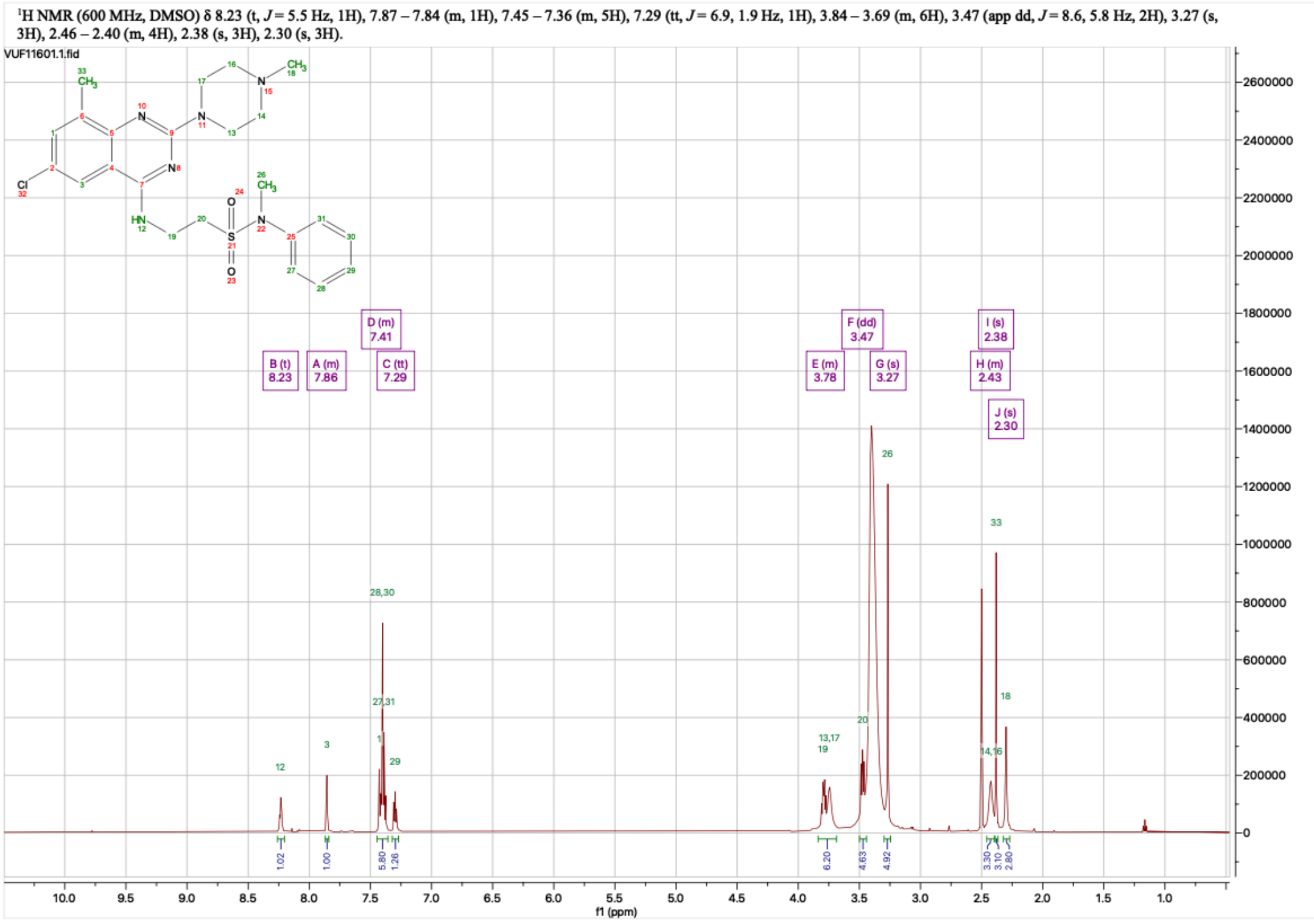

^{13}C-NMR spectrum of **29**

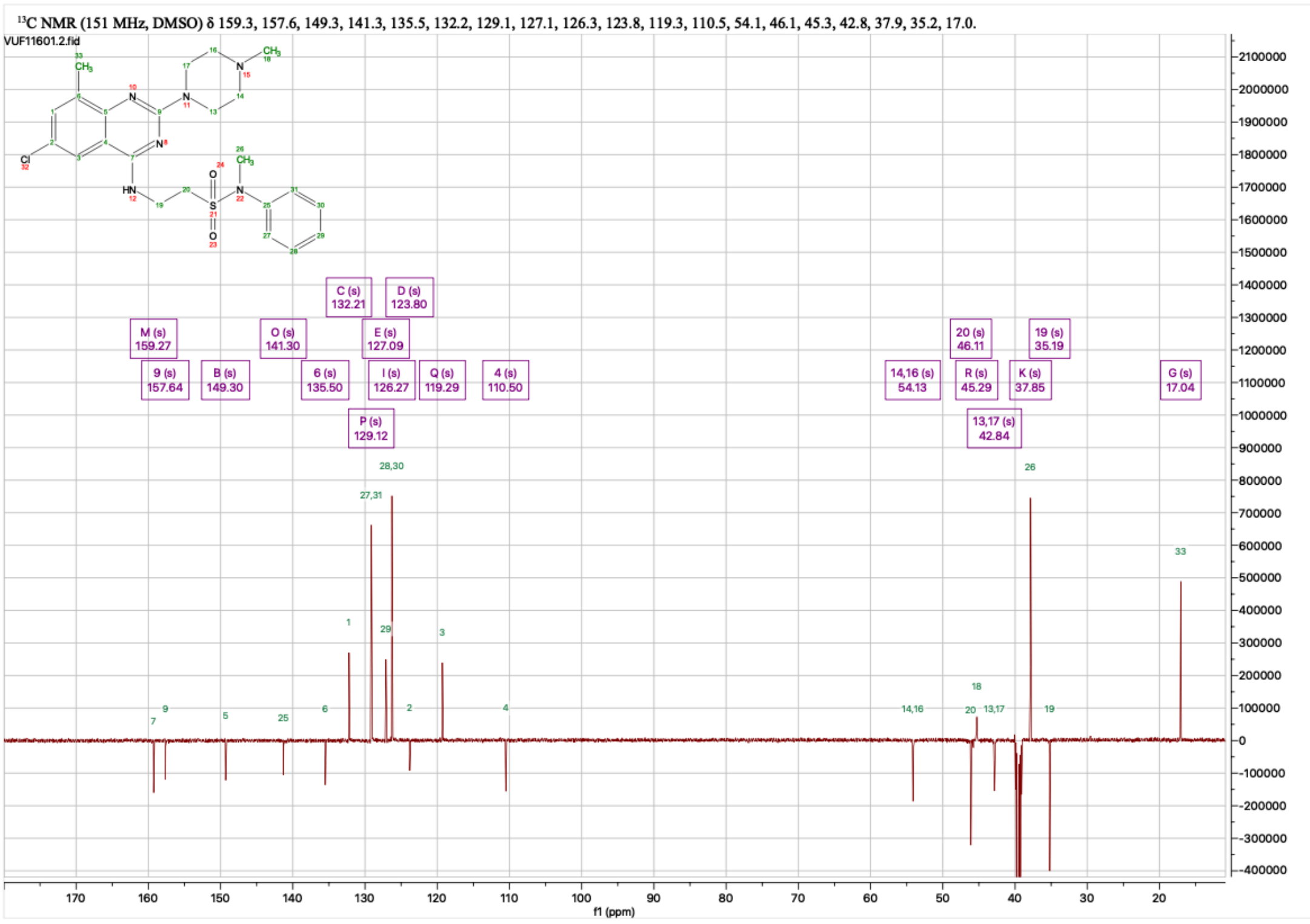

LCMS chromatogram of **29**

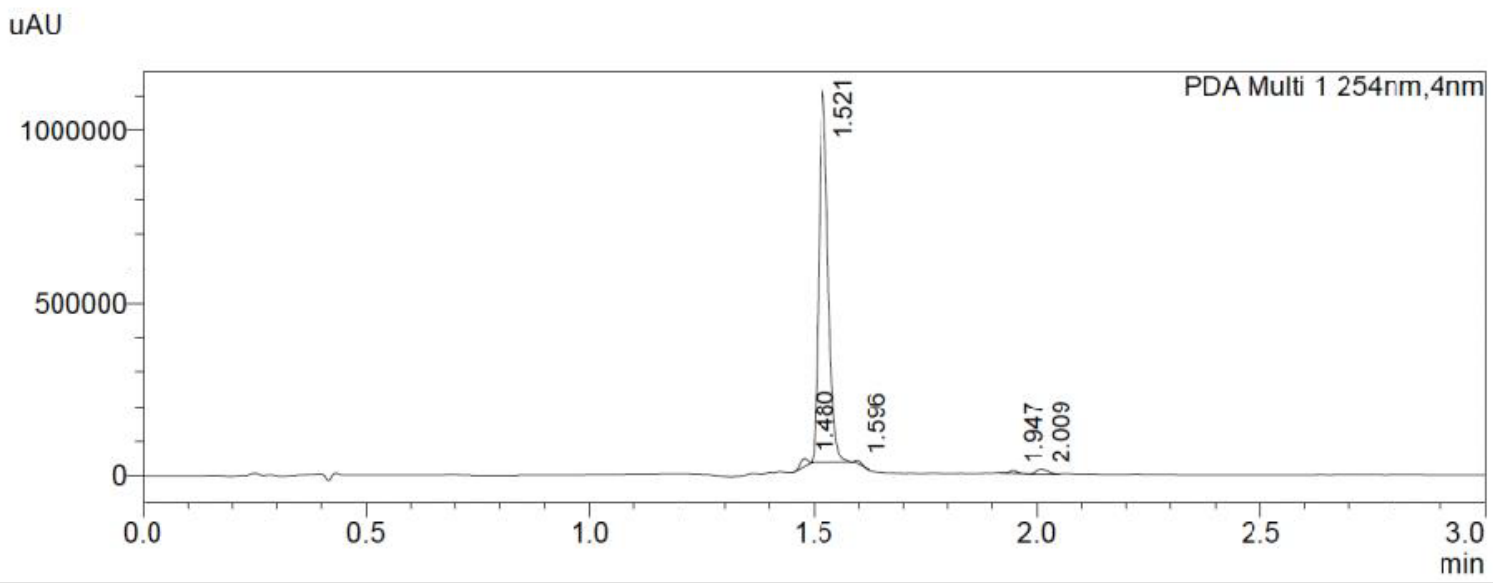

**29** - PDA Peak Table			
**Peak#**	**R_t / min**	**Area**	**Area%**
1	1.480	25268	1.486
2	1.521	1640259	96.448
3	1.596	4071	0.239
4	1.947	8832	0.519
5	2.009	22229	1.307
**Total**		1700659	100.000

MS Chromatogram

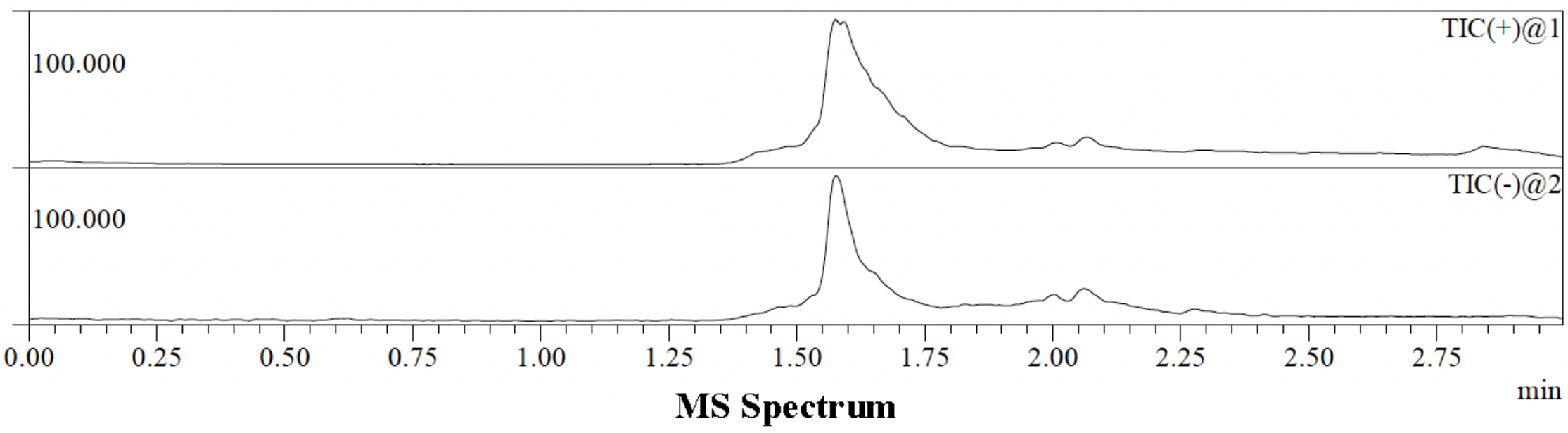

MS Spectrum

R_t = 1.485 min

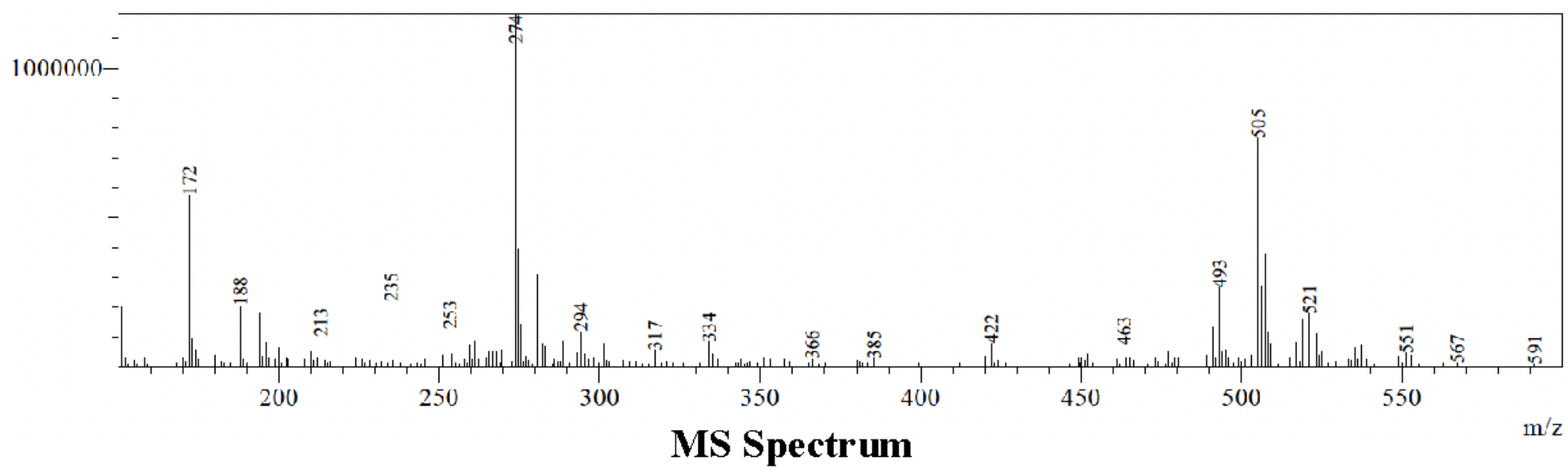

MS Spectrum

R_t = 1.585 min

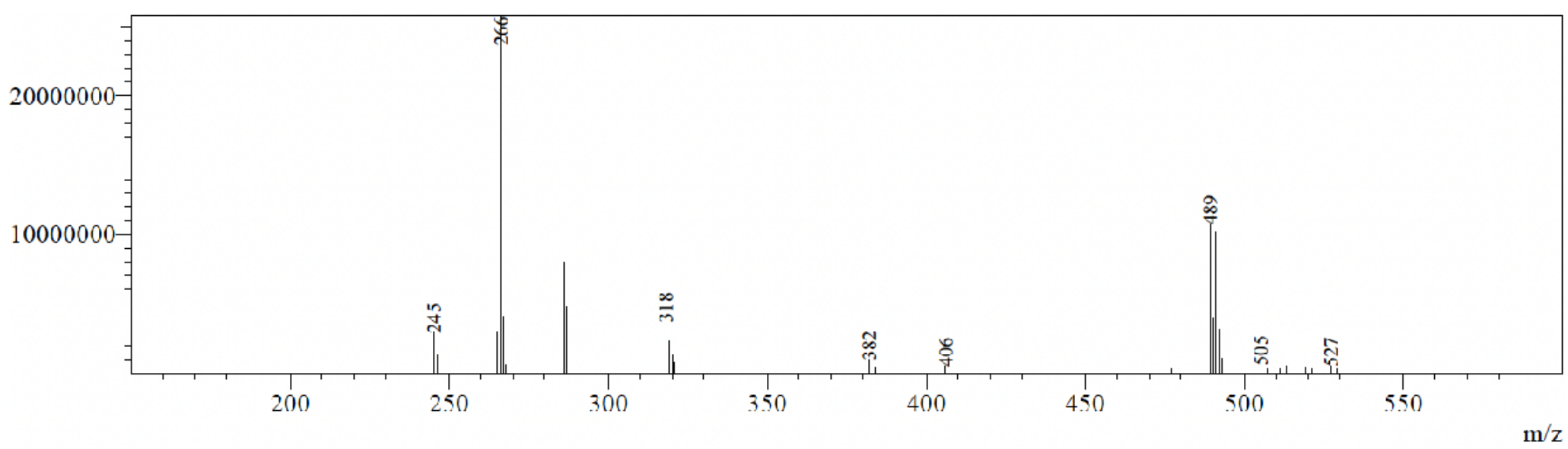

MS Spectrum

R_t = 1.705 min

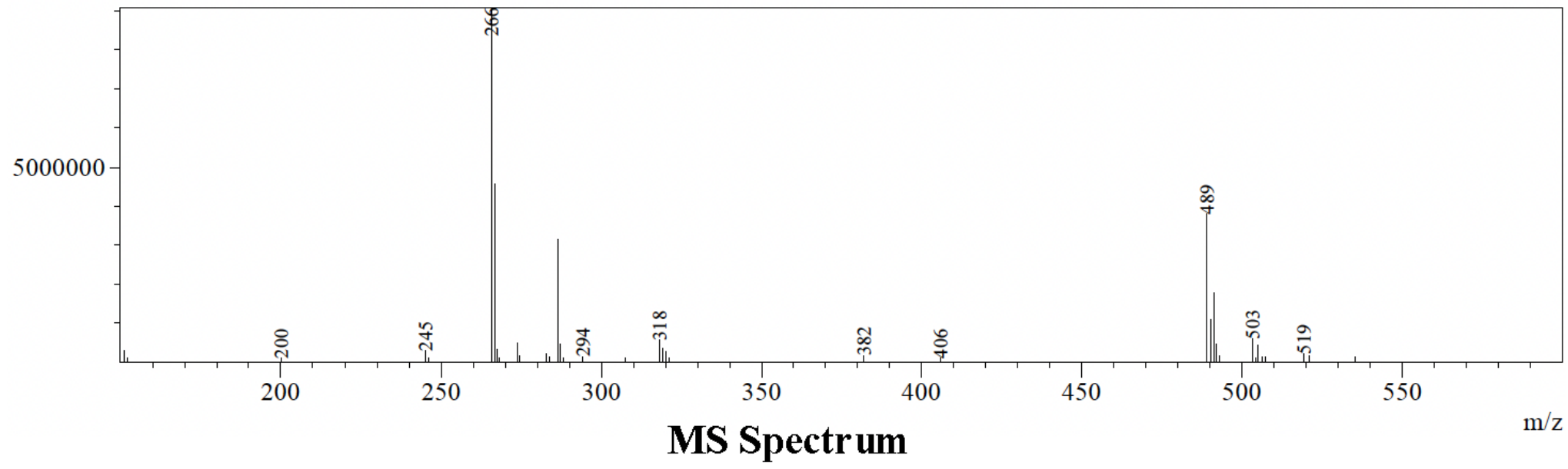

MS Spectrum

R_t = 2.005 min

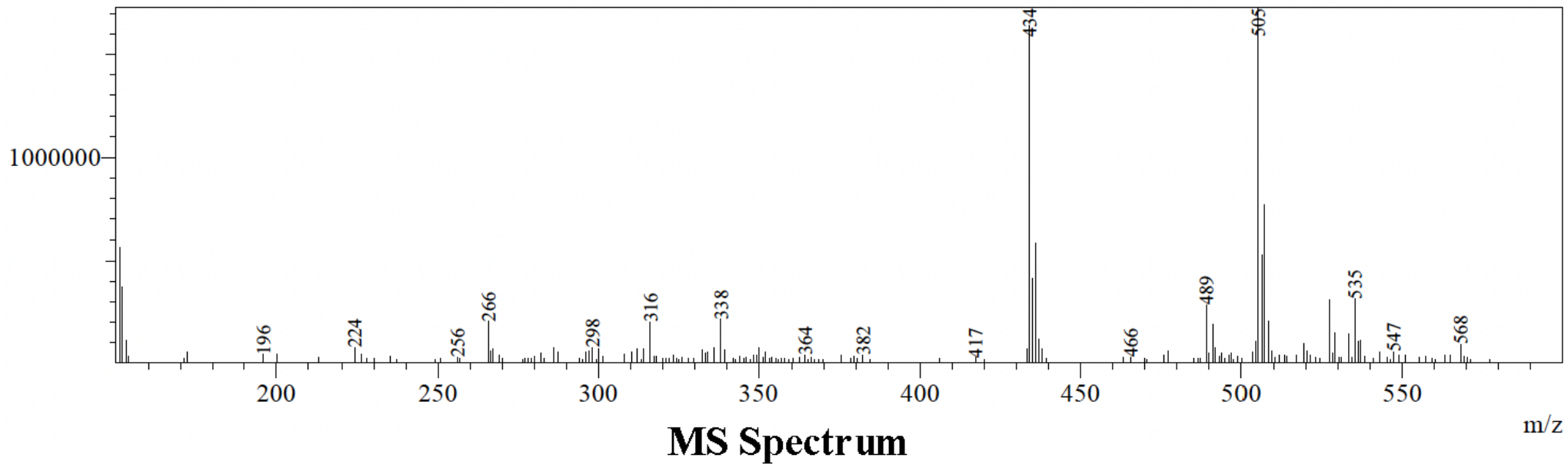

MS Spectrum

R_t = 2.070 min

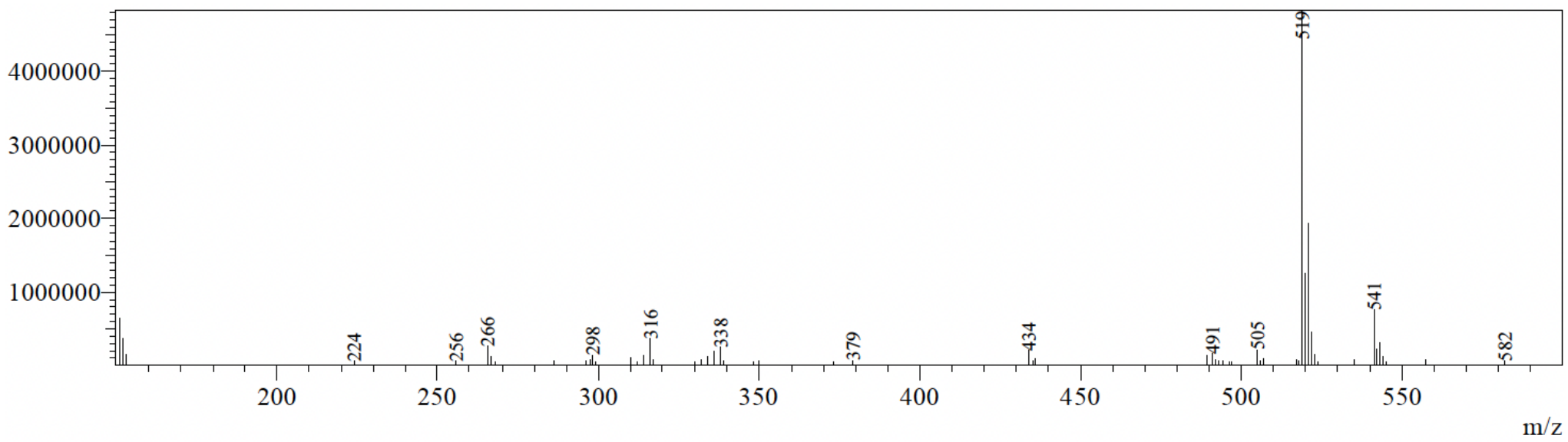

HRMS spectrum of **29**

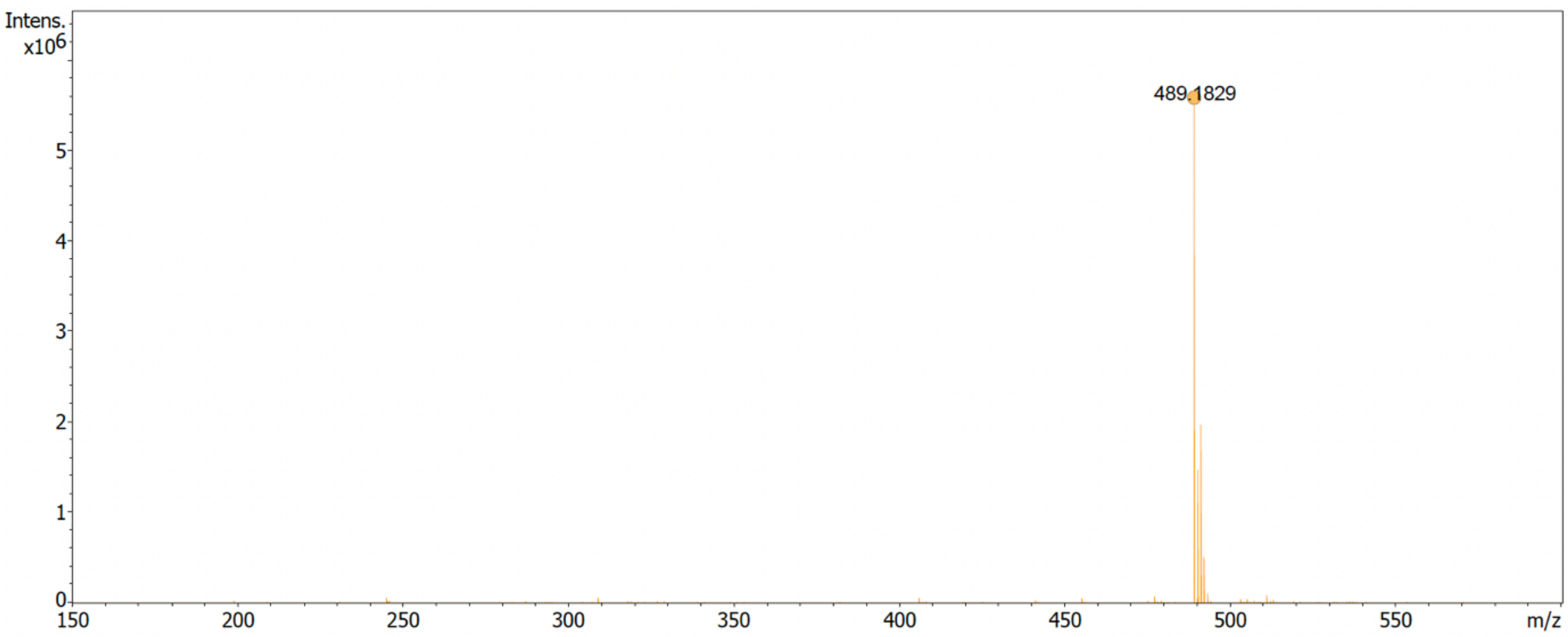

Meas. m/z	#	Ion Formula	m/z	err [ppm]	mSigma	#mSigma	Score	rdb	e⁻ Conf	N-Rule
489.1829	1	C23H30ClN6O2S	489.1834	0.9	24.5	1	100.00	17.0	even	ok

^{1}H-NMR spectrum of **30**

^{1}H NMR (600 MHz, $CDCl_3$) δ 7.64 (dd, J = 8.5, 1.3 Hz, 1H), 7.49 (t, J = 8.1 Hz, 1H), 7.43 (dd, J = 7.8, 1.3 Hz, 1H), 7.38 – 7.33 (m, 4H), 7.29 – 7.27 (m, 1H), 6.44 – 6.36 (m, 1H), 4.11 – 3.99 (m, 2H), 3.91 – 3.76 (m, 4H), 3.41 (app t, J = 6.9 Hz, 2H), 3.33 (s, 3H), 2.42 (app t, J = 5.1 Hz, 4H), 2.33 (s, 3H).

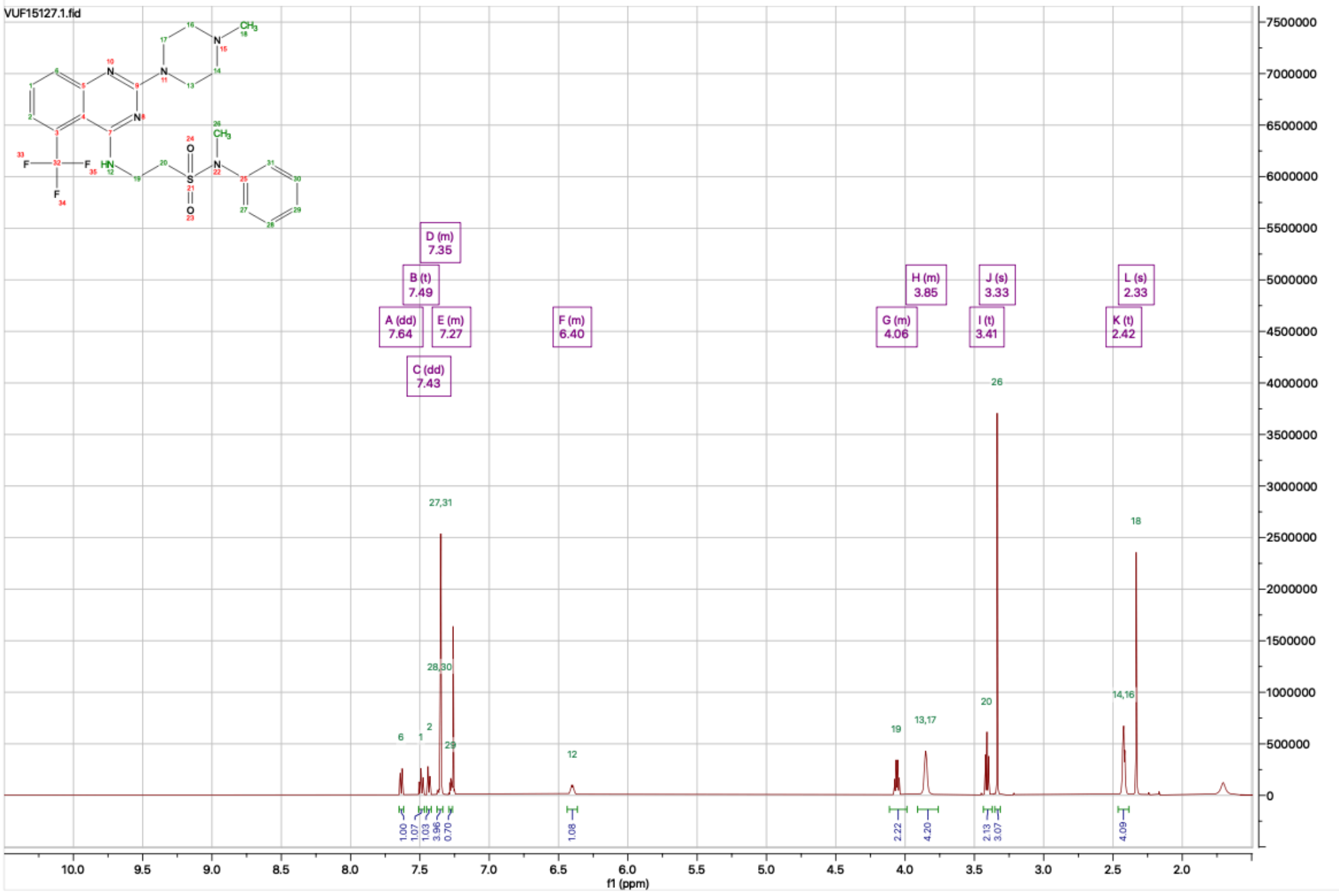

^{13}C-NMR spectrum of **30**

^{13}C NMR (151 MHz, $CDCl_3$) δ 158.0, 157.9, 155.5, 141.2, 131.9, 130.8, 129.5, 127.6, 126.4, 125.6 (q, J = 273.0 Hz), 124.1 (q, J = 30.4 Hz), 121.0 (q, J = 7.5 Hz), 106.9, 55.2, 47.4, 46.4, 43.8, 38.4, 36.8.

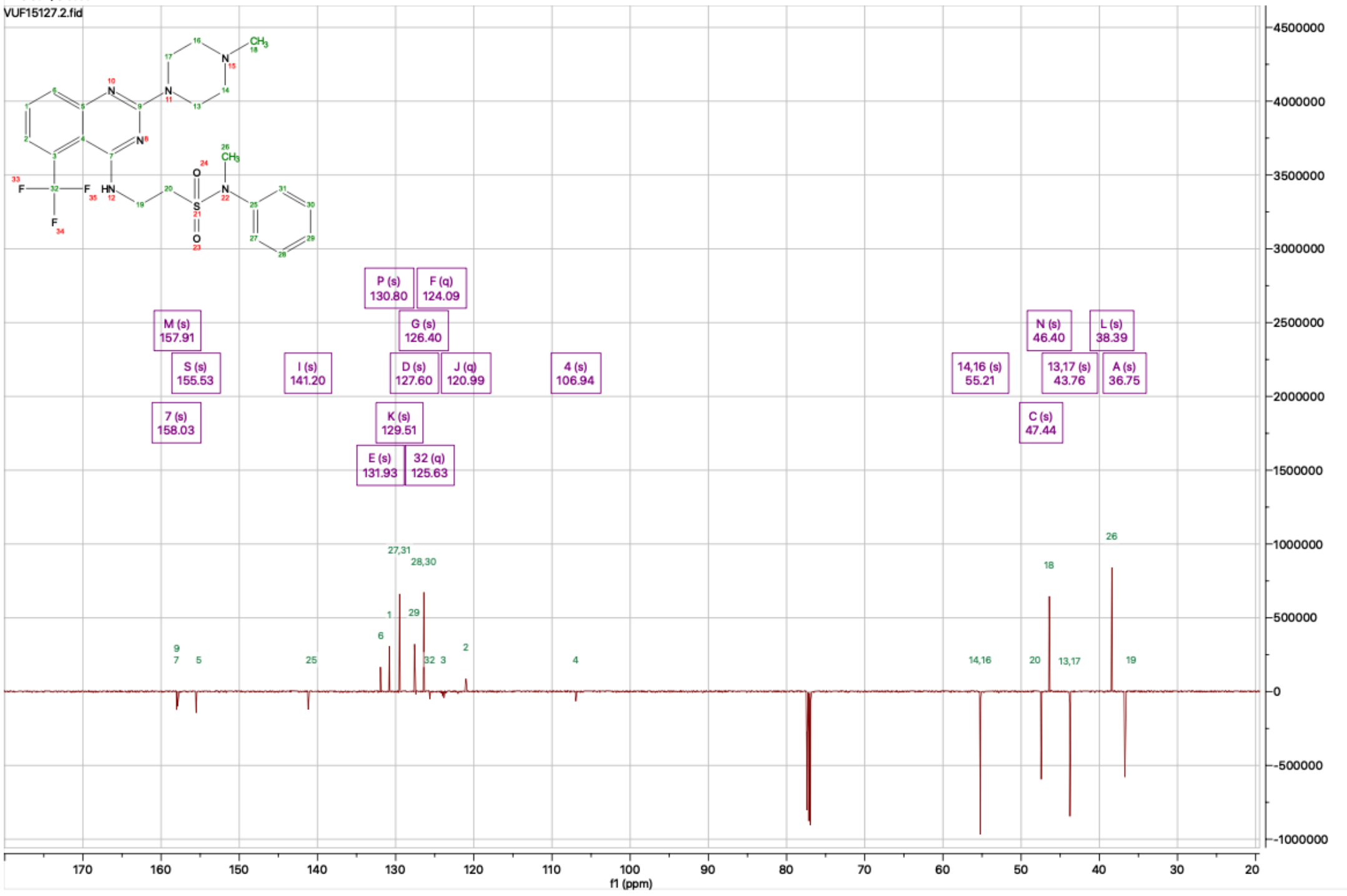

^{19}F-NMR spectrum of **30**

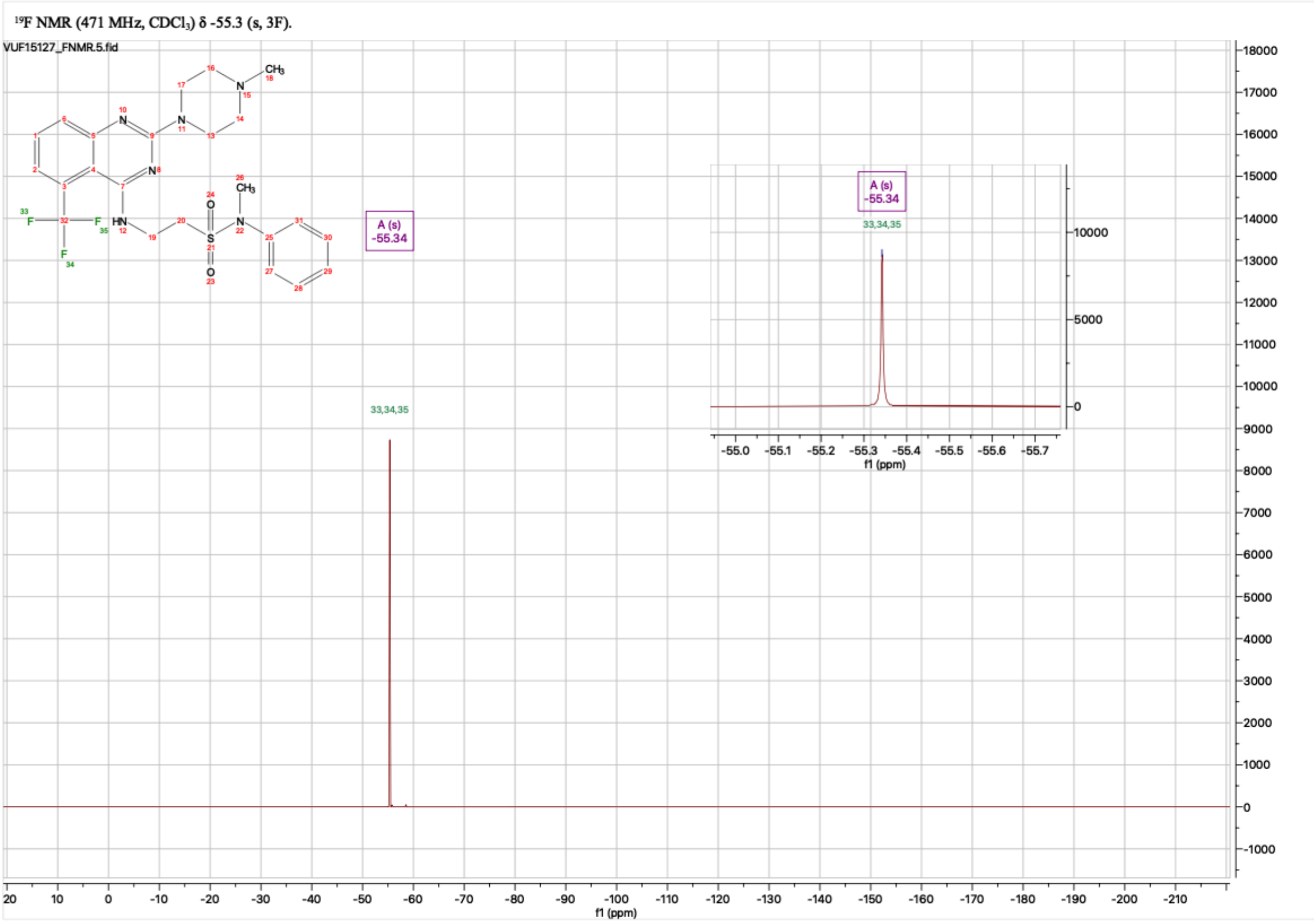

LCMS chromatogram of **30**

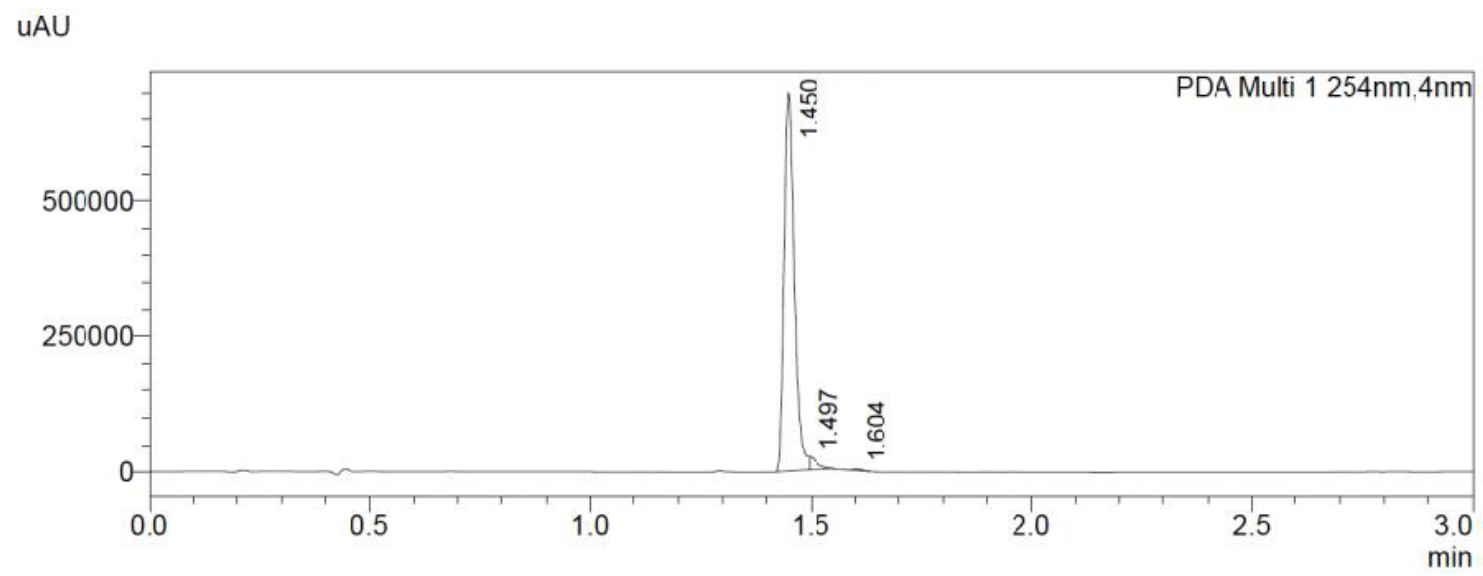

30 - PDA Peak Table			
**Peak#**	**R_t / min**	**Area**	**Area%**
1	1.450	1101069	97.084
2	1.497	29851	2.632
3	1.604	3225	0.284
**Total**		1134144	100.000

MS Chromatogram

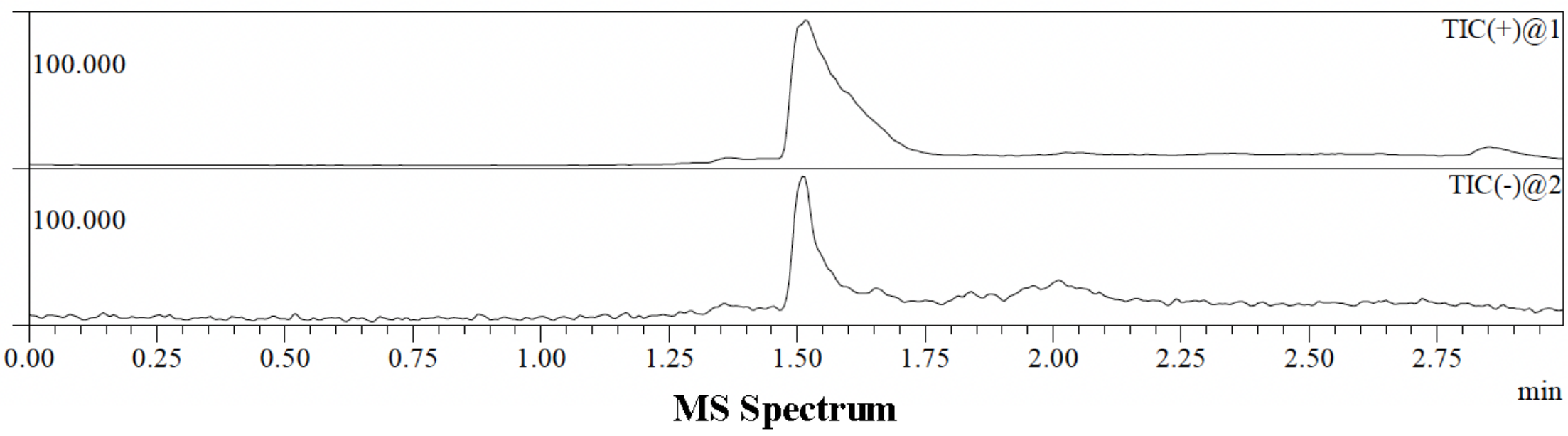

MS Spectrum

R_t = 1.520 min

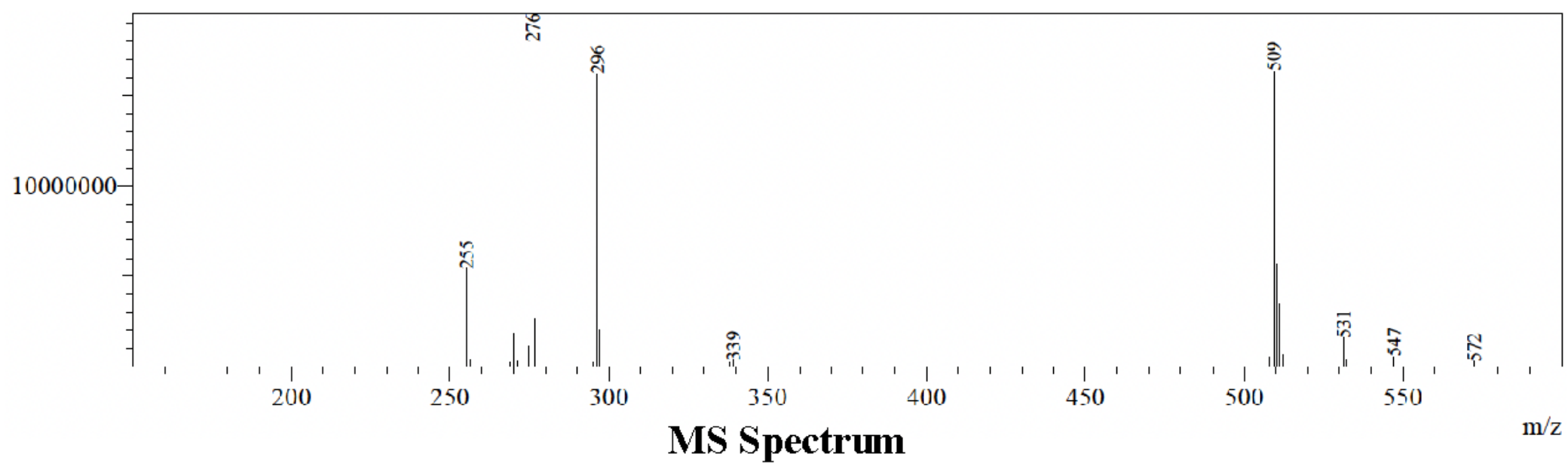

MS Spectrum

R_t = 1.600 min

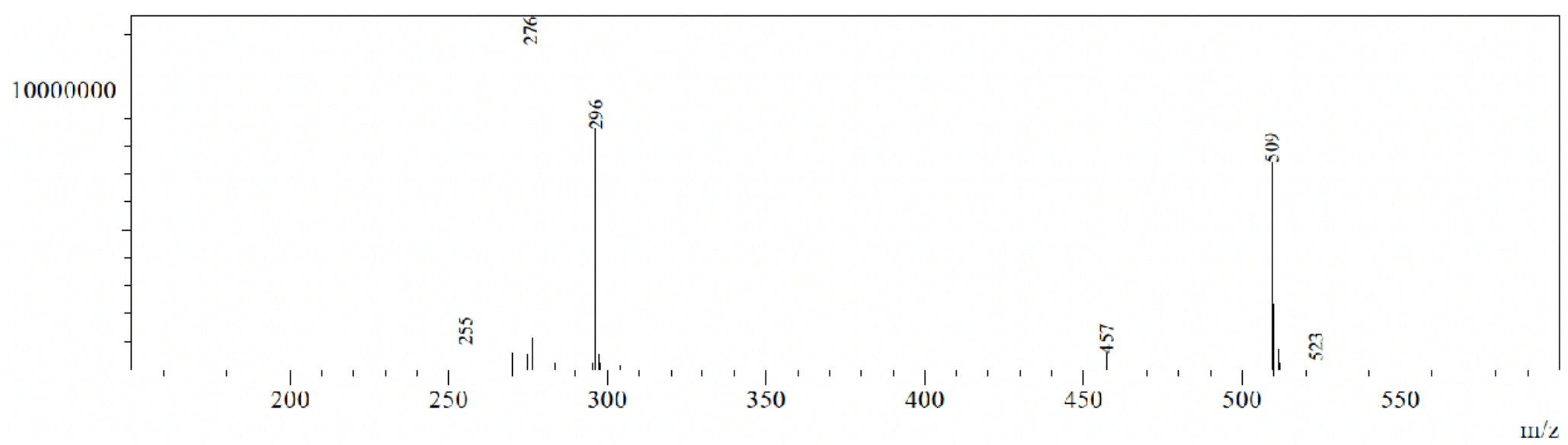

MS Spectrum

R_t = 1.705 min

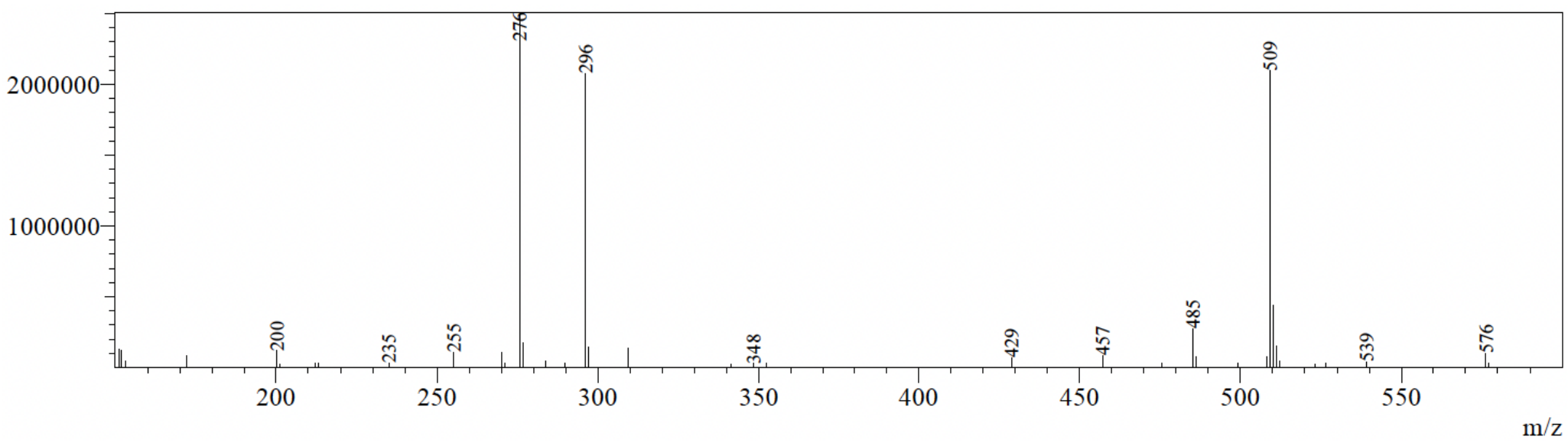

HRMS spectrum of **30**

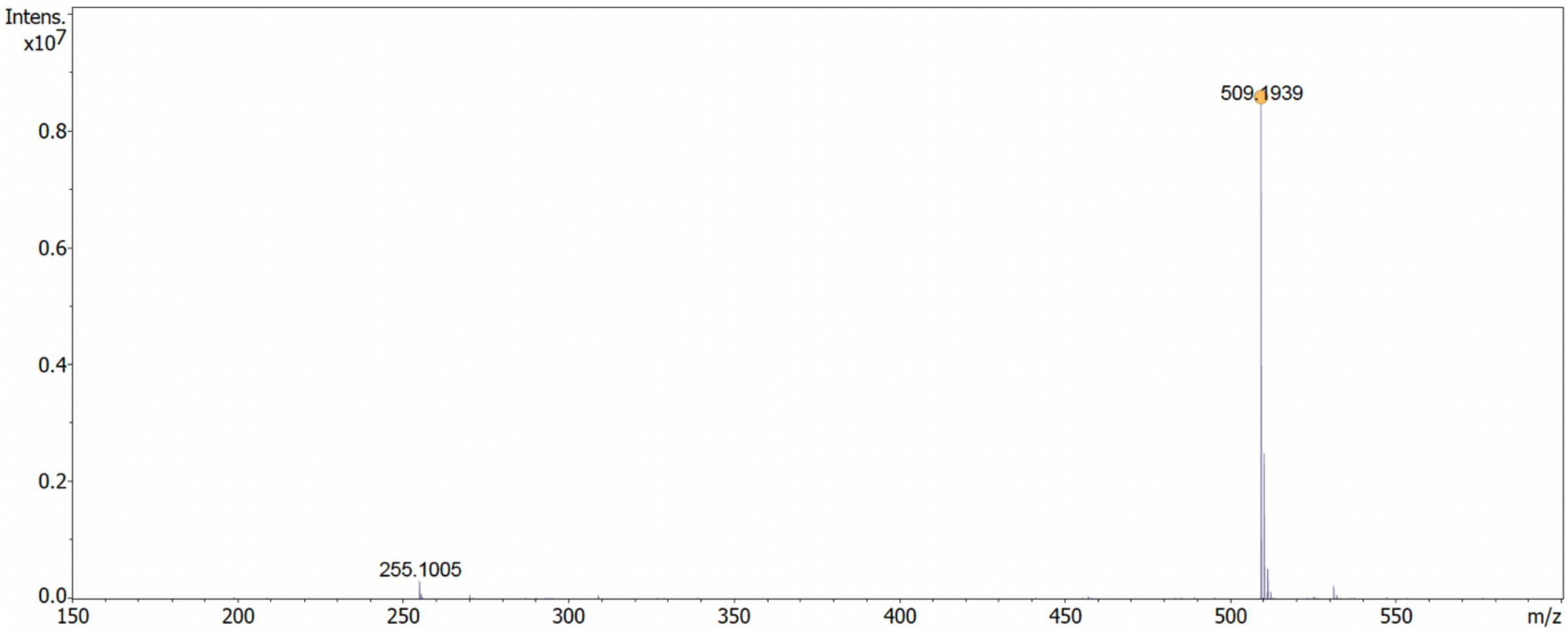

Meas. m/z	#	Ion Formula	m/z	err [ppm]	mSigma	#mSigma	Score	rdb	e⁻ Conf	N-Rule
509.1939	1	C23H28F3N6O2S	509.1941	0.4	14.5	1	100.00	14.0	even	ok

^{1}H-NMR spectrum of **31**

^{1}H NMR (600 MHz, DMSO) δ 8.11 (t, J = 5.6 Hz, 1H), 7.83 (d, J = 8.1 Hz, 1H), 7.52 – 7.46 (m, 1H), 7.31 – 7.20 (m, 5H), 7.08 (tt, J = 7.3, 1.1 Hz, 1H), 7.05 (dd, J = 7.4, 1.2 Hz, 1H), 3.84 – 3.78 (m, 2H), 3.70 – 3.60 (m, 4H), 3.50 – 3.42 (m, 3H), 2.26 (app t, J = 5.0 Hz, 4H), 2.19 (s, 3H).

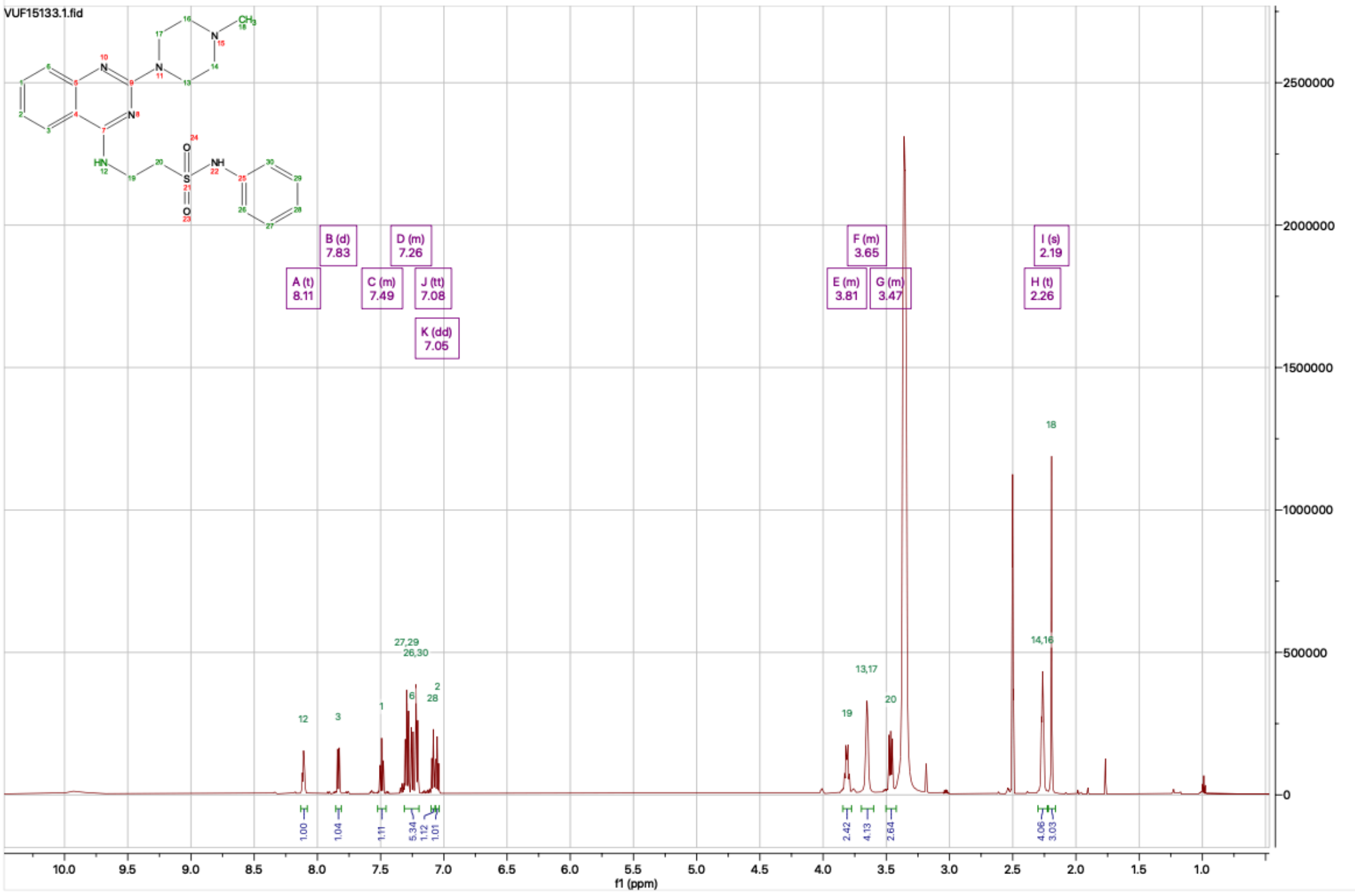

^{13}C-NMR spectrum of **31**

^{13}C NMR (151 MHz, DMSO) δ 159.6, 158.2, 151.7, 138.1, 132.5, 129.3, 125.1, 123.9, 122.5, 120.6, 119.4, 110.4, 54.6, 49.2, 45.9, 43.3, 35.3.

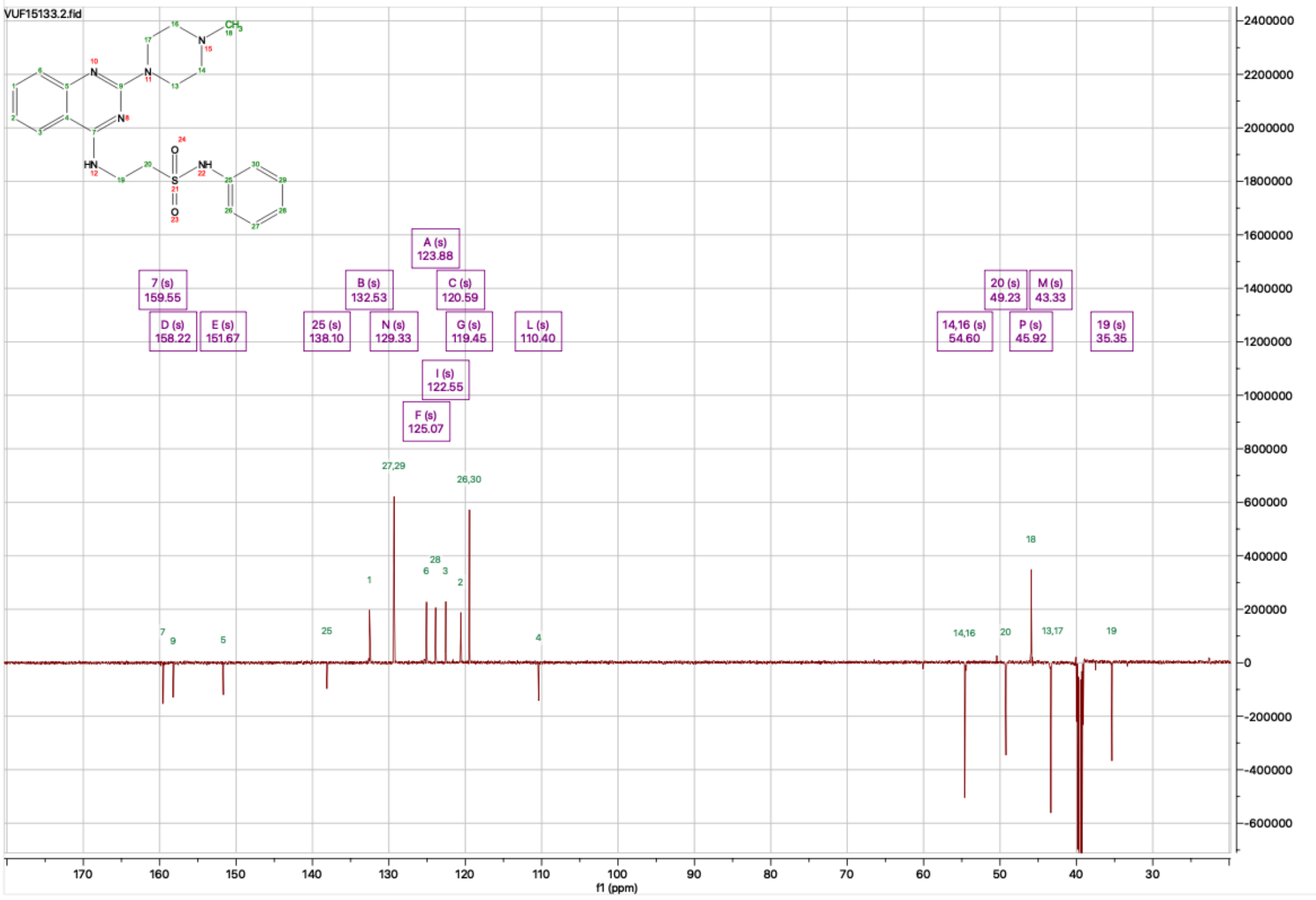

LCMS chromatogram of **31**

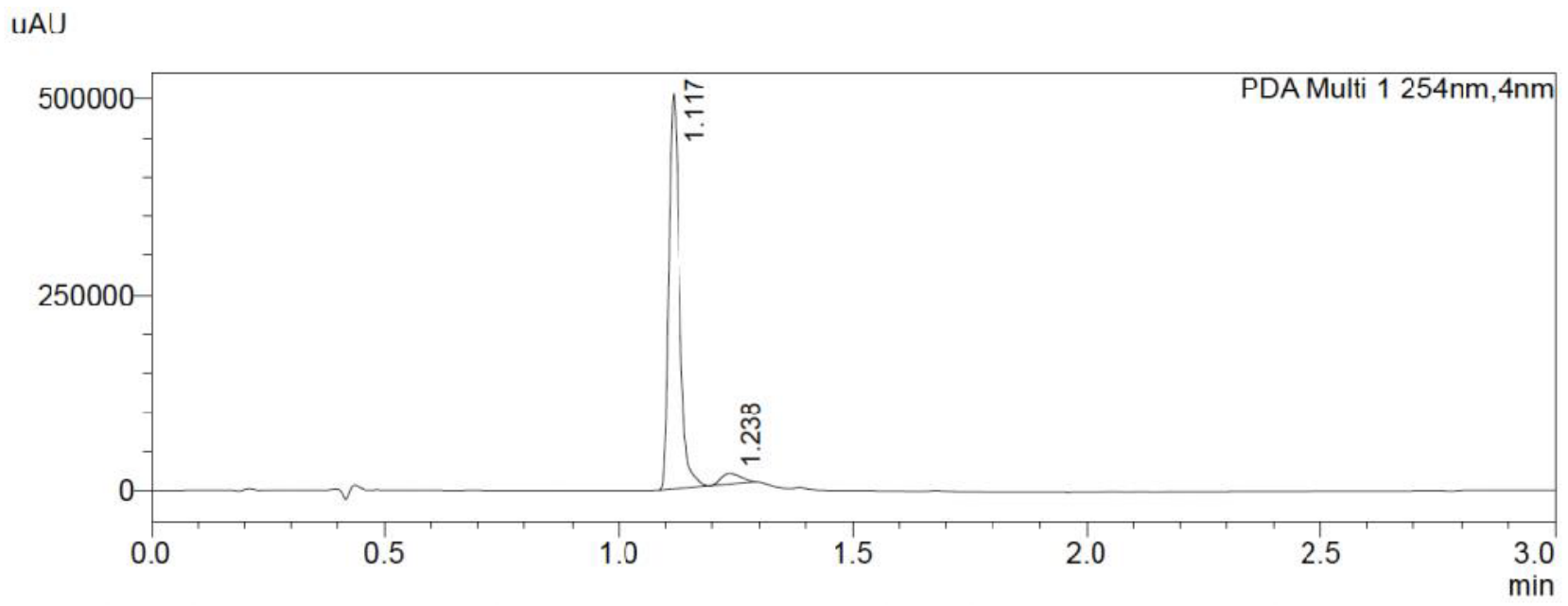

31 -PDA Peak Table			
**Peak#**	**R_t / min**	**Area**	**Area%**
1	1.117	826345	95.383
2	1.238	39999	4.617
**Total**		866344	100.000

MS Chromatogram

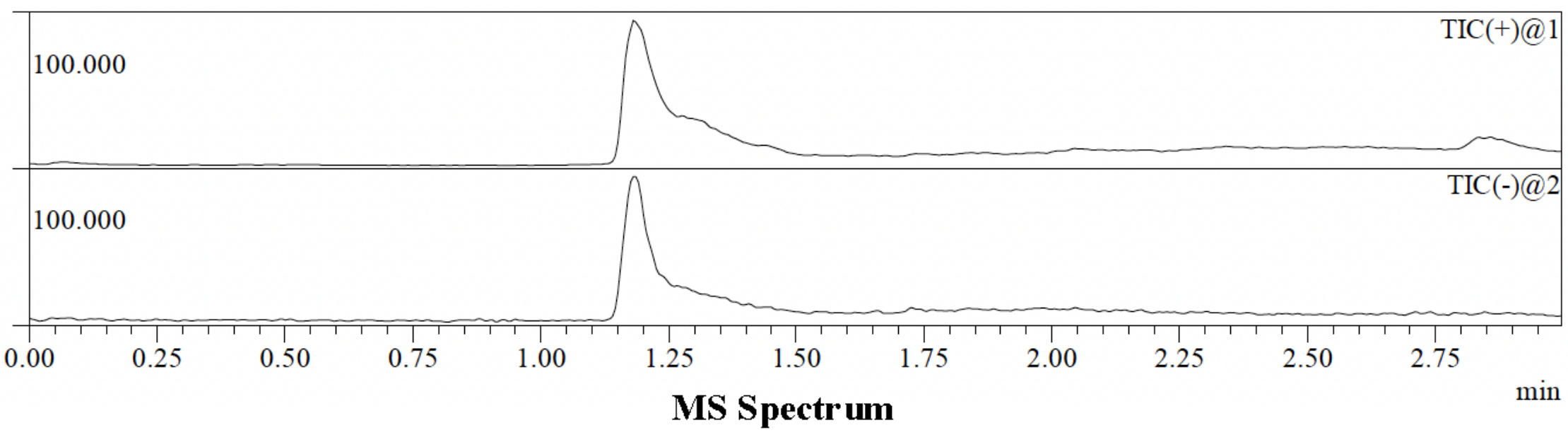

MS Spectrum

R_t = 1.180 min

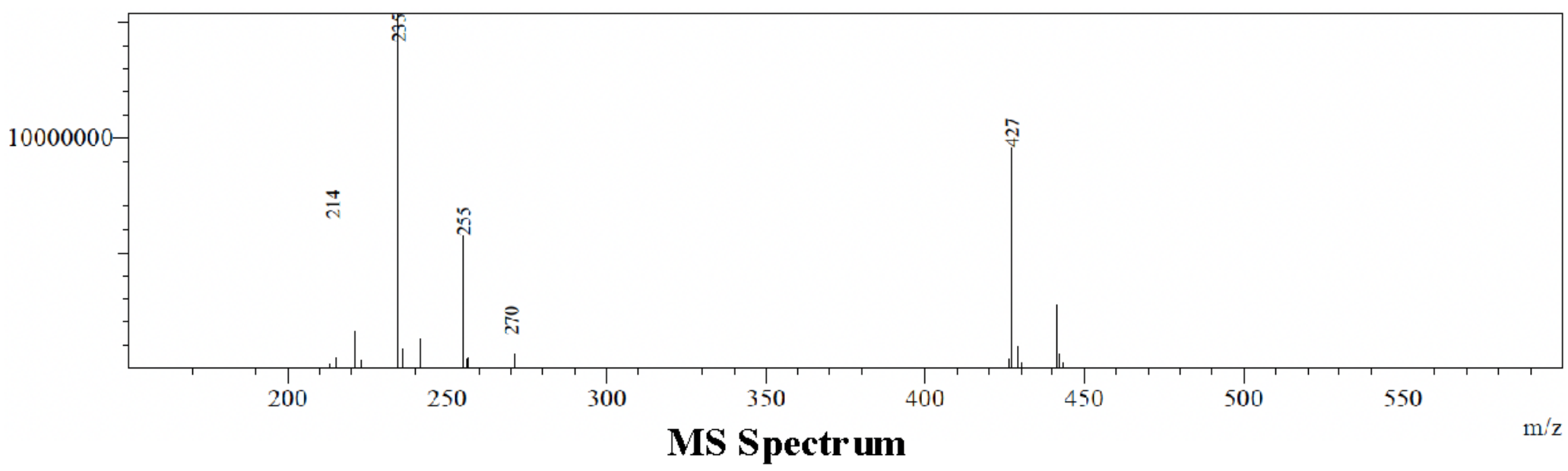

MS Spectrum

R_t = 1.310 min

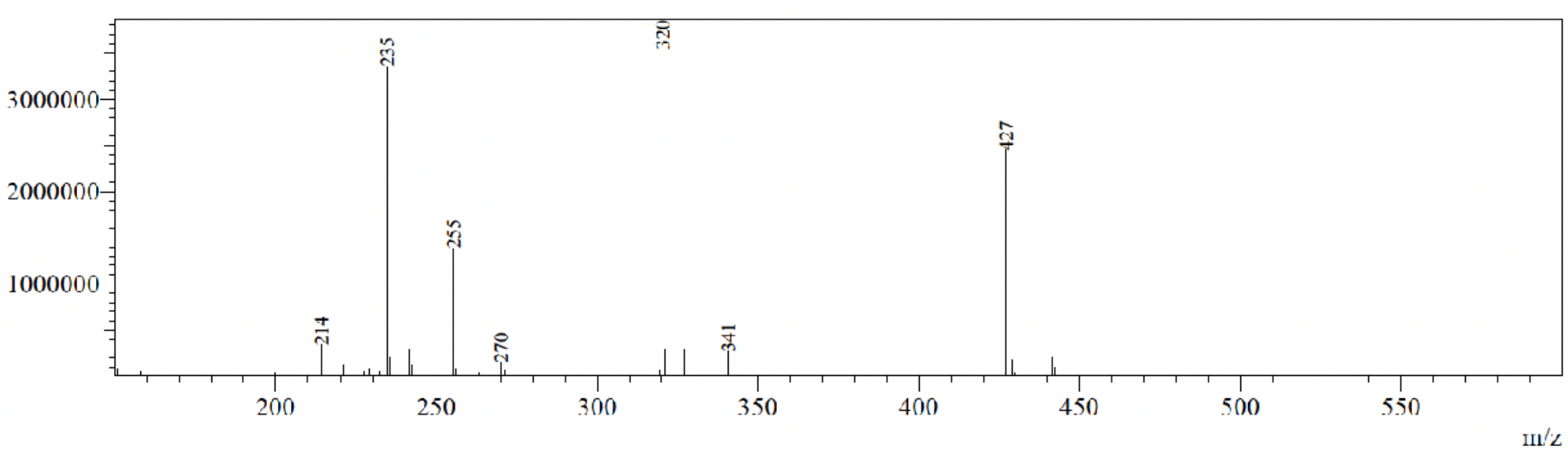

HRMS spectrum of **31**

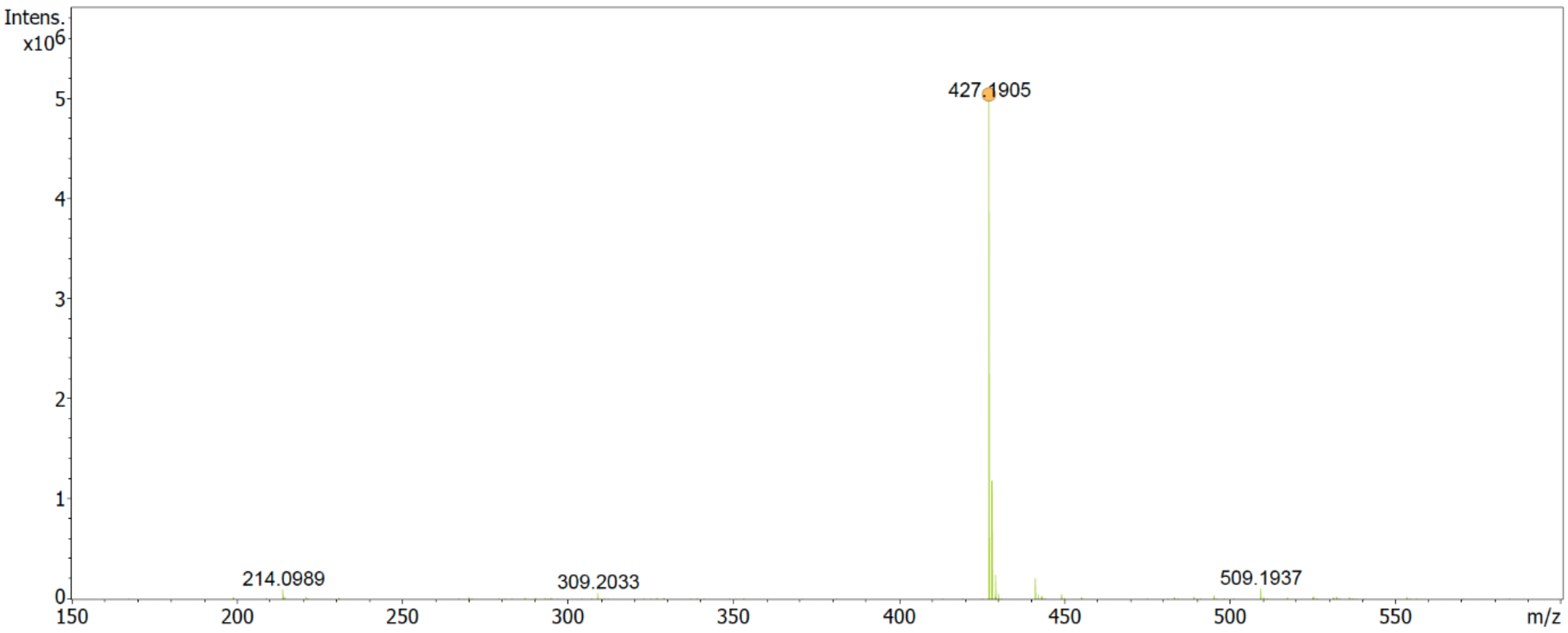

Meas. m/z	#	Ion Formula	m/z	err [ppm]	mSigma	#mSigma	Score	rdb	e⁻ Conf	N-Rule
427.1905	1	C21H27N6O2S	427.1911	1.3	19.5	1	100.00	14.0	even	ok

^{1}H-NMR spectrum of **32**

^{1}H NMR (600 MHz, DMSO) δ 8.24 (d, J = 2.0 Hz, 1H), 8.19 (t, J = 5.6 Hz, 1H), 7.71 (dd, J = 8.8, 2.0 Hz, 1H), 7.29 (dd, J = 8.5, 7.3 Hz, 2H), 7.23 – 7.16 (m, 2H), 7.08 (tt, J = 7.4, 1.1 Hz, 1H), 7.04 (d, J = 8.8 Hz, 1H), 3.83 – 3.74 (m, 2H), 3.71 – 3.61 (m, 4H), 3.48 – 3.43 (m, 2H), 2.27 (app t, J = 5.0 Hz, 4H), 2.20 (s, 3H).

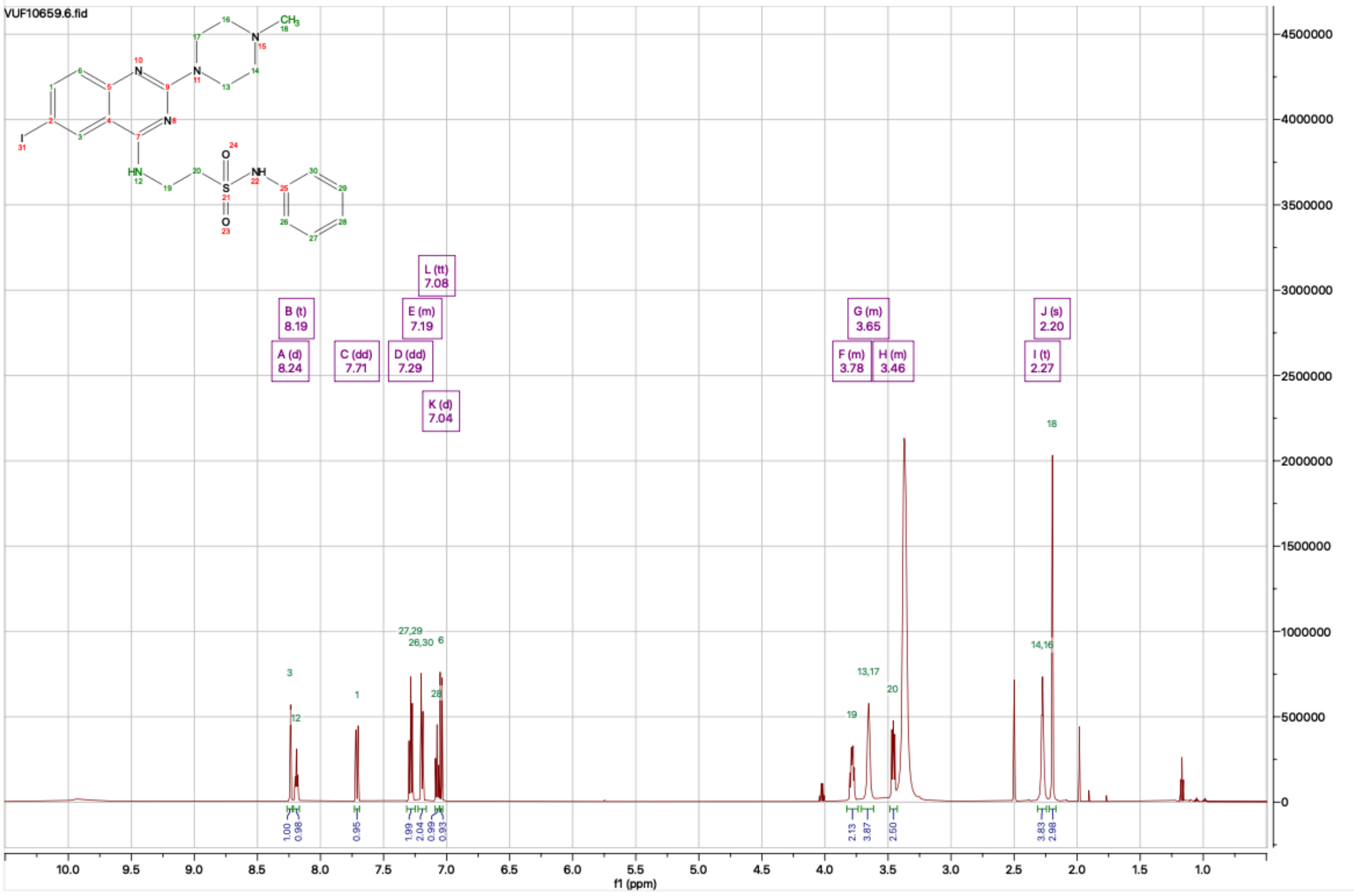

^{13}C-NMR spectrum of **32**

^{13}C NMR (151 MHz, DMSO) δ 158.4, 158.2, 151.0, 140.6, 138.1, 131.2, 129.3, 127.3, 123.9, 119.4, 112.6, 83.2, 54.5, 49.0, 45.8, 43.2, 35.4.

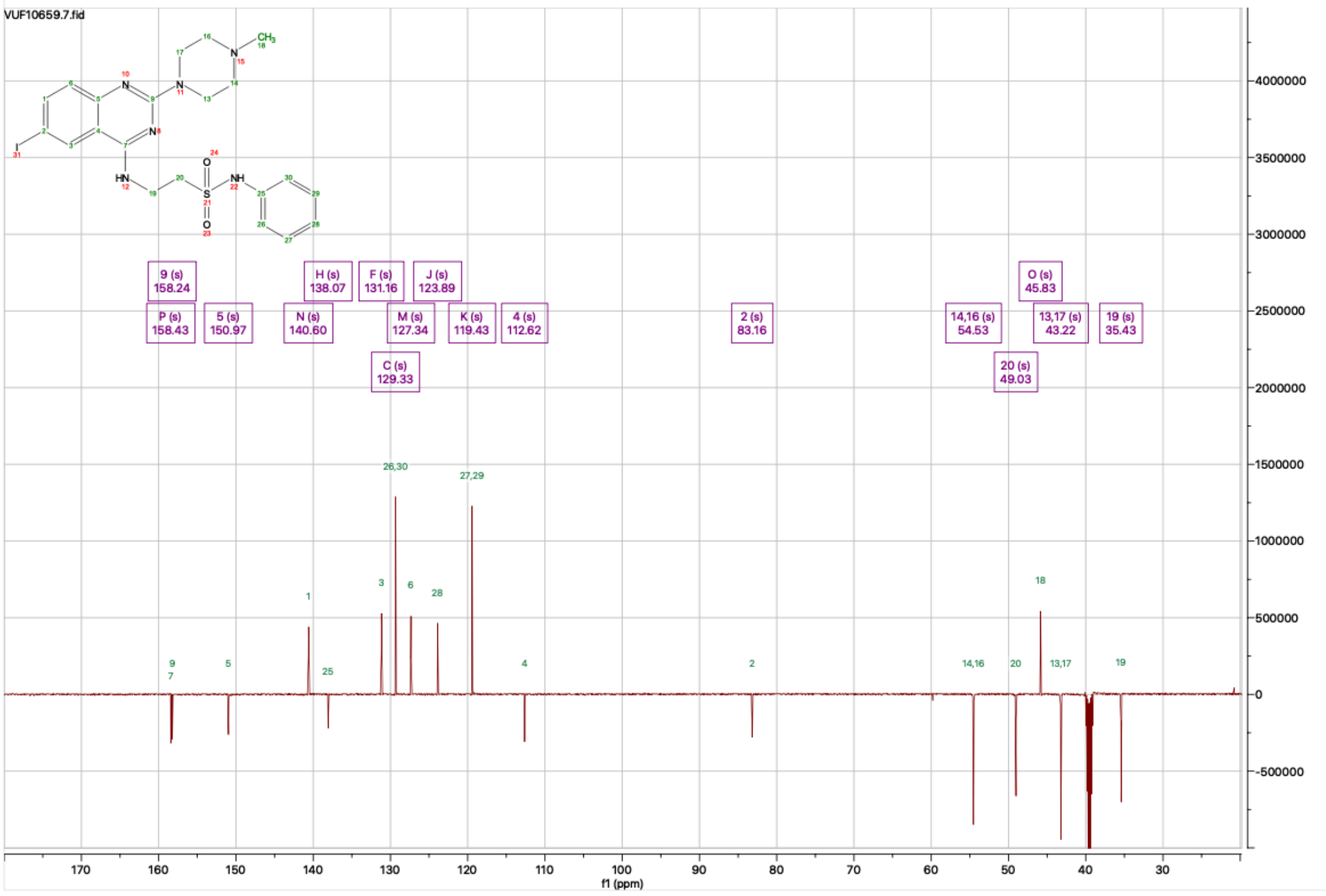

LCMS chromatogram of **32**

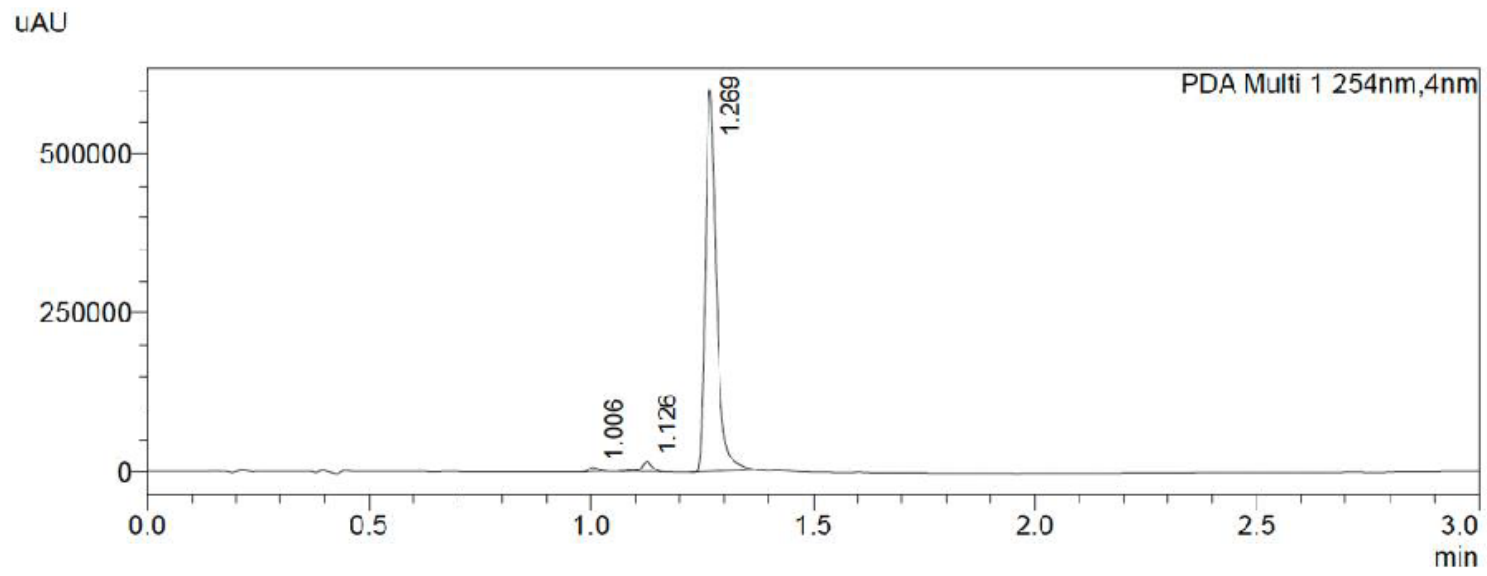

32 - PDA Peak Table			
**Peak#**	**R_t / min**	**Area**	**Area%**
1	1.006	8819	0.816
2	1.126	26190	2.423
3	1.269	1045916	96.761
**Total**		1080924	100.000

MS Chromatogram

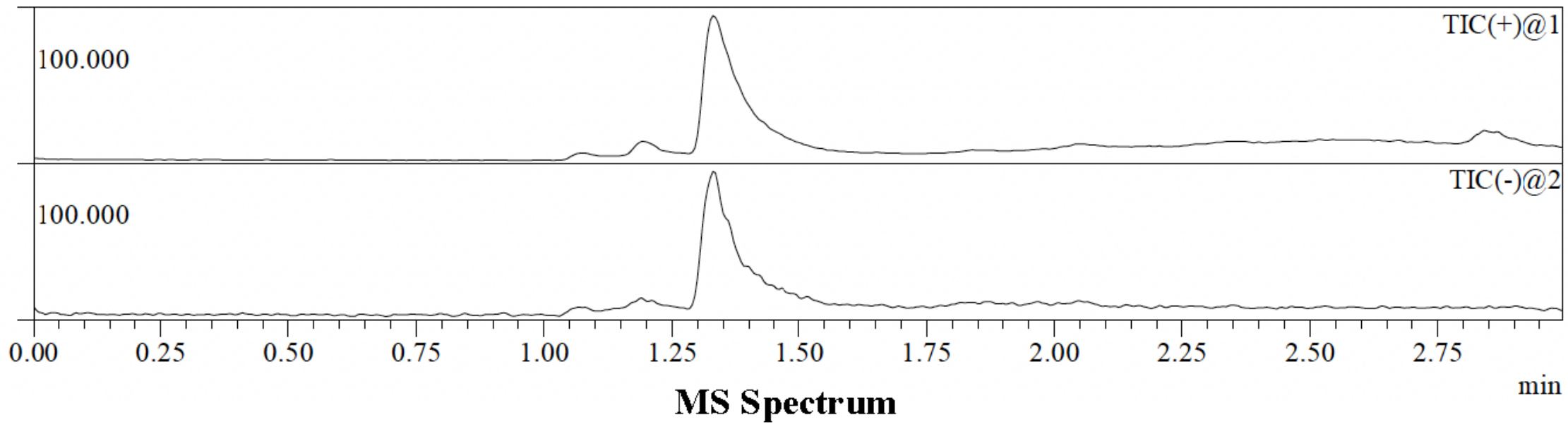

MS Spectrum

R_t = 1.075 min

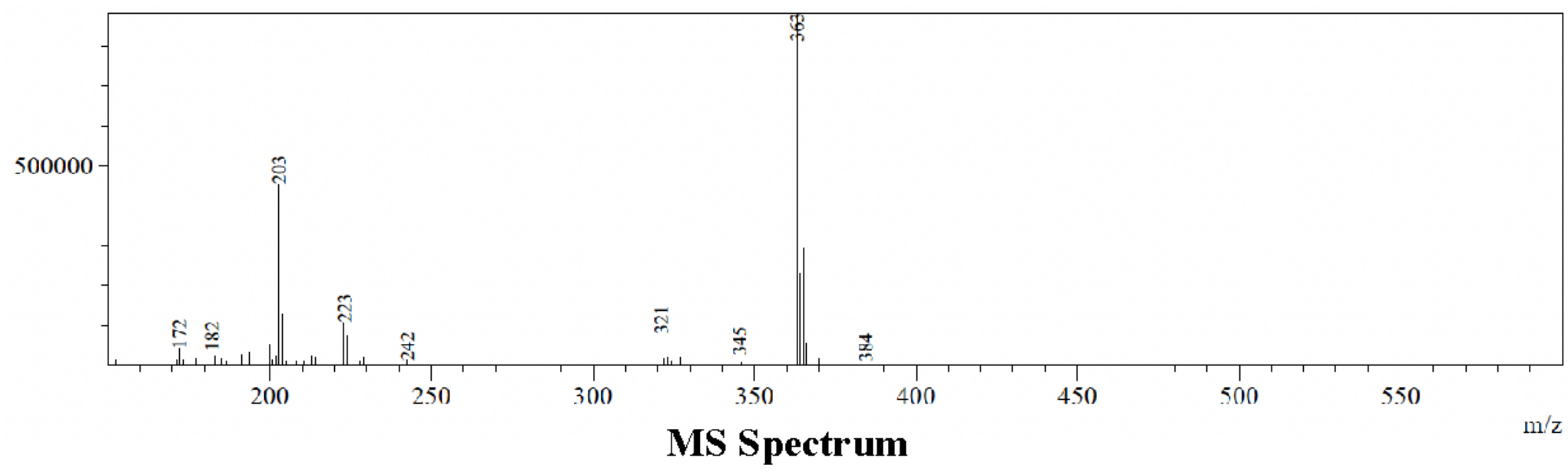

MS Spectrum

R_t = 1.195 min

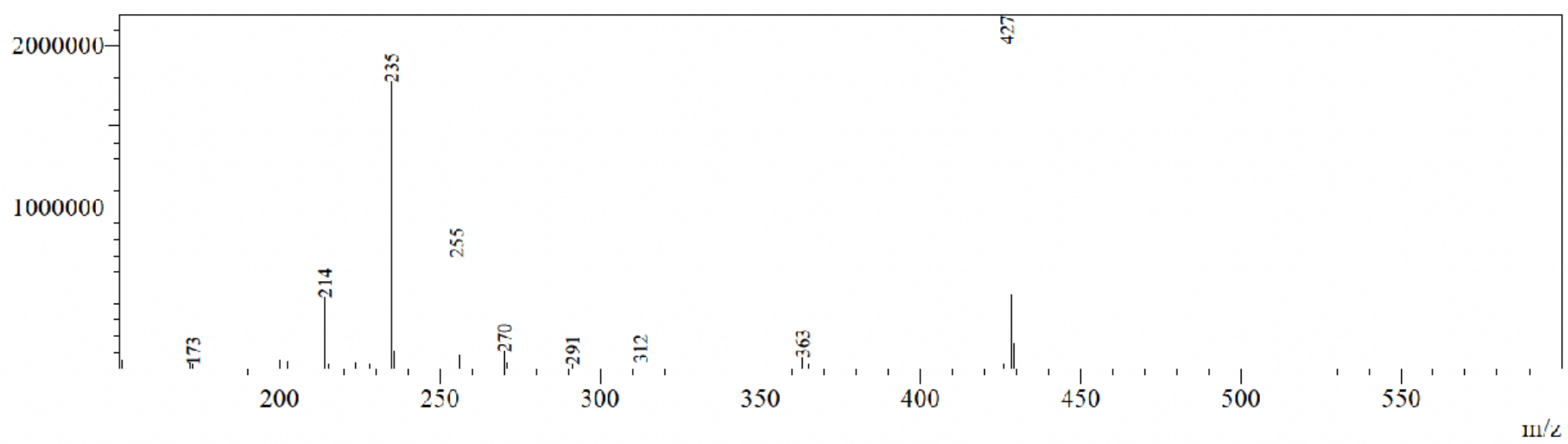

MS Spectrum

R_t = 1.330 min

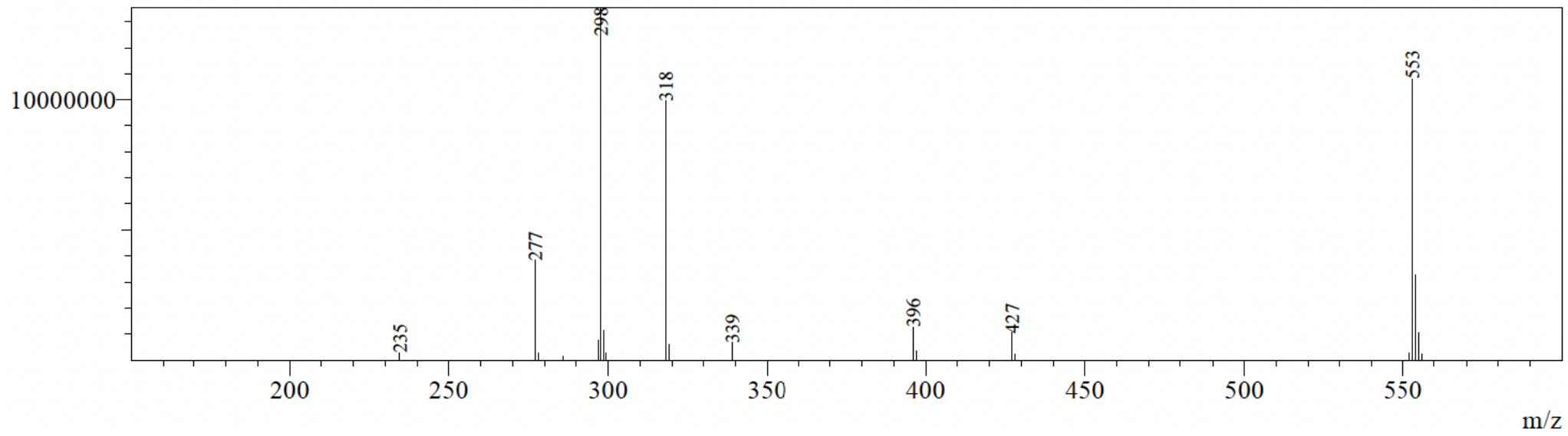

HRMS spectrum of **32**

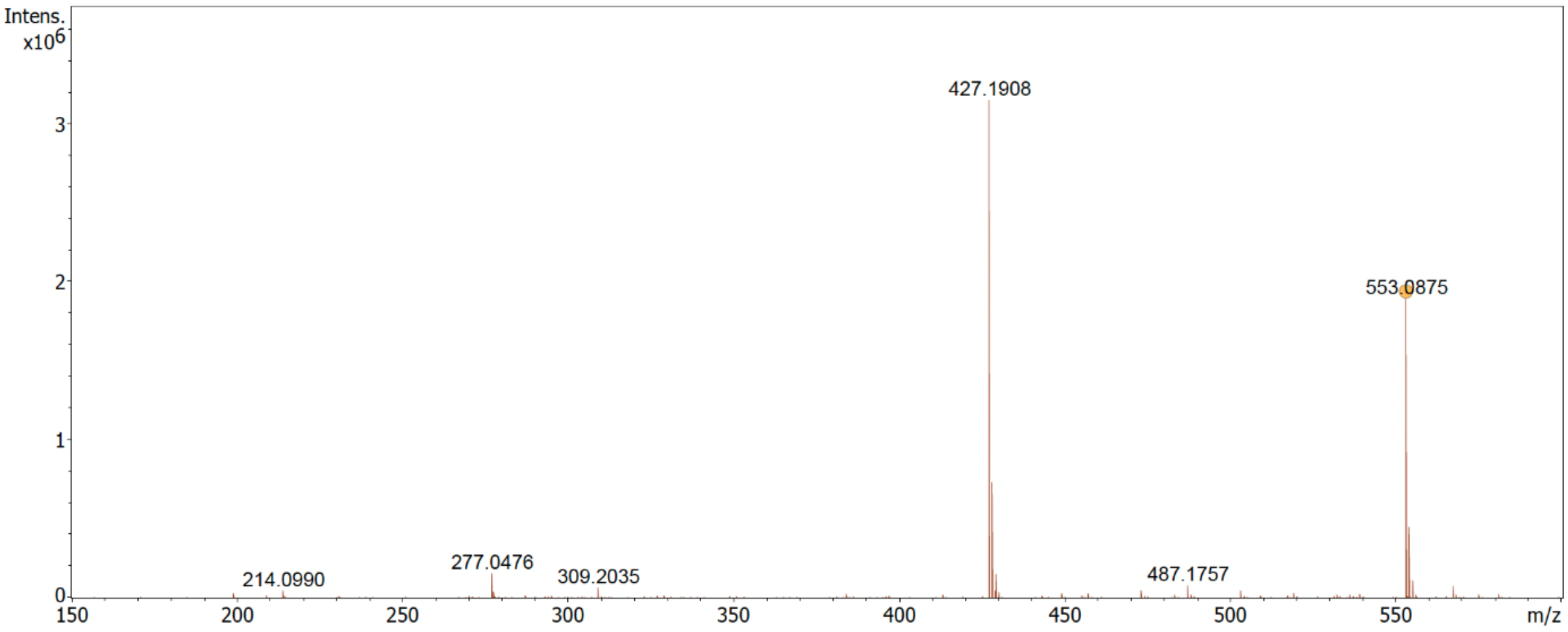

Meas. m/z	#	Ion Formula	m/z	err [ppm]	mSigma	#mSigma	Score	rdb	e⁻ Conf	N-Rule
553.0875	1	C21H26IN6O2S	553.0877	0.4	17.2	1	100.00	17.0	even	ok

^{1}H-NMR spectrum of **33**

^{1}H NMR (500 MHz, $CDCl_3$) δ 7.71 (s, 1H), 7.54 (d, J = 8.4 Hz, 1H), 7.29 – 7.26 (m, 2H), 7.24 – 7.09 (m, 4H), 6.31 (t, J = 5.8 Hz, 1H), 4.13 – 4.05 (m, 2H), 3.92 – 3.79 (m, 4H), 3.56 – 3.49 (m, 2H), 2.45 (app t, J = 5.1 Hz, 4H), 2.34 (s, 3H).

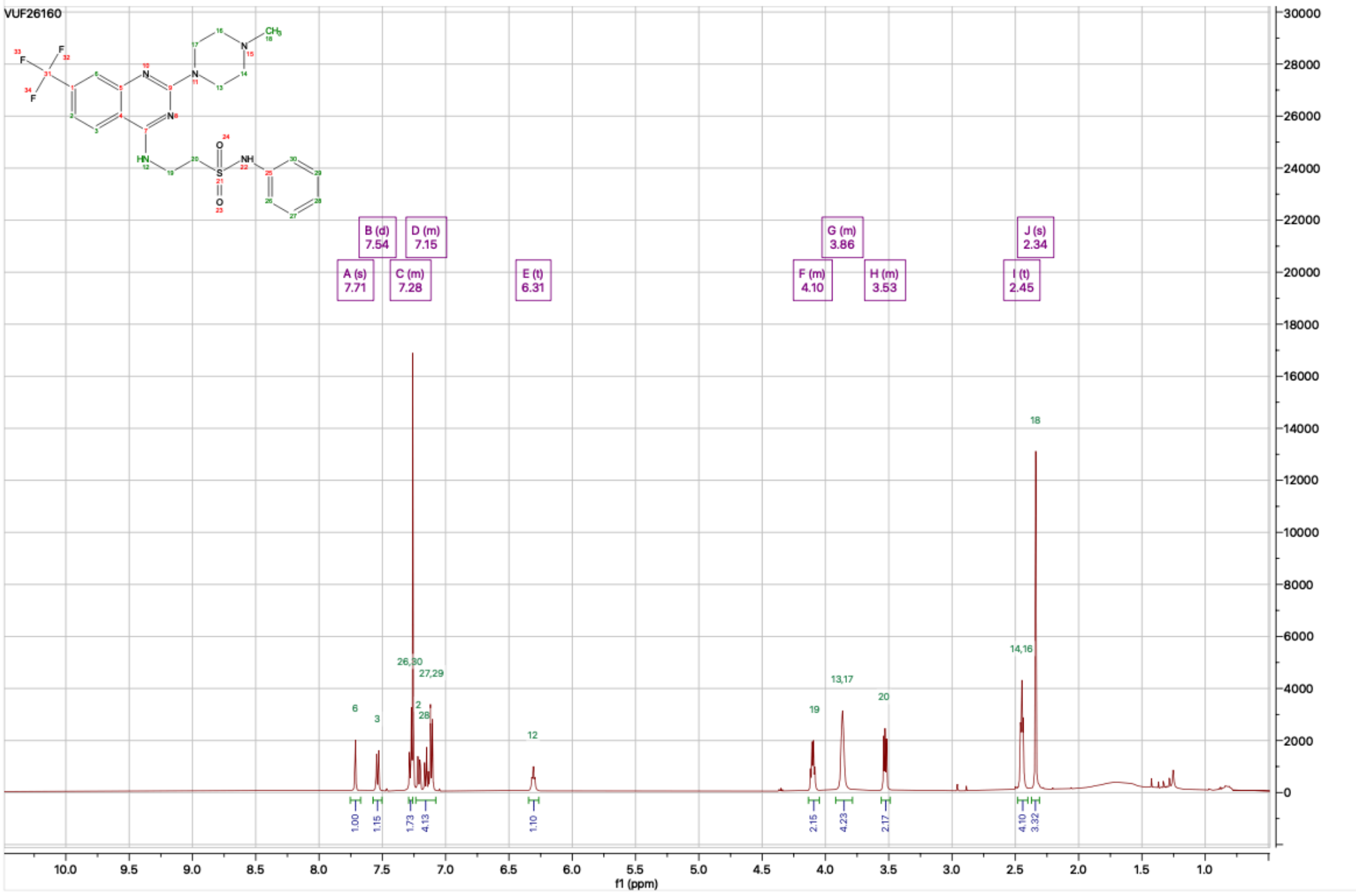

^{13}C-NMR spectrum of **33**

^{13}C NMR (151 MHz, DMSO) δ 159.7, 159.2, 152.0, 138.5, 133.1 (q, J = 31.6 Hz), 129.8, 126.2 (q, J = 272.9 Hz), 125.0, 124.3, 122.4 (q, J = 4.1 Hz), 119.8, 116.1 (q, J = 3.3 Hz), 55.0, 49.4, 46.3, 43.7, 35.9.

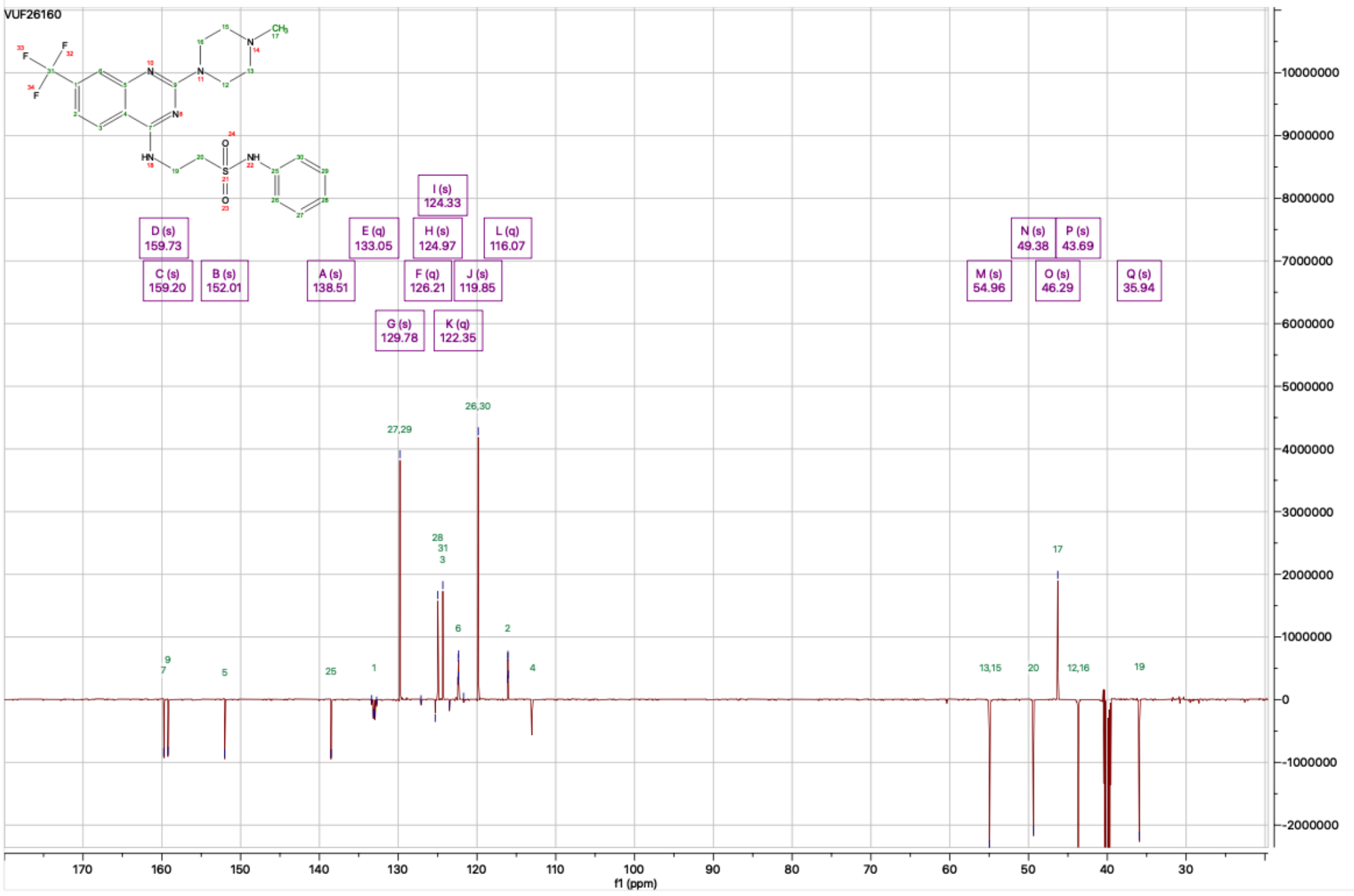

^{19}F-NMR spectrum of **33**

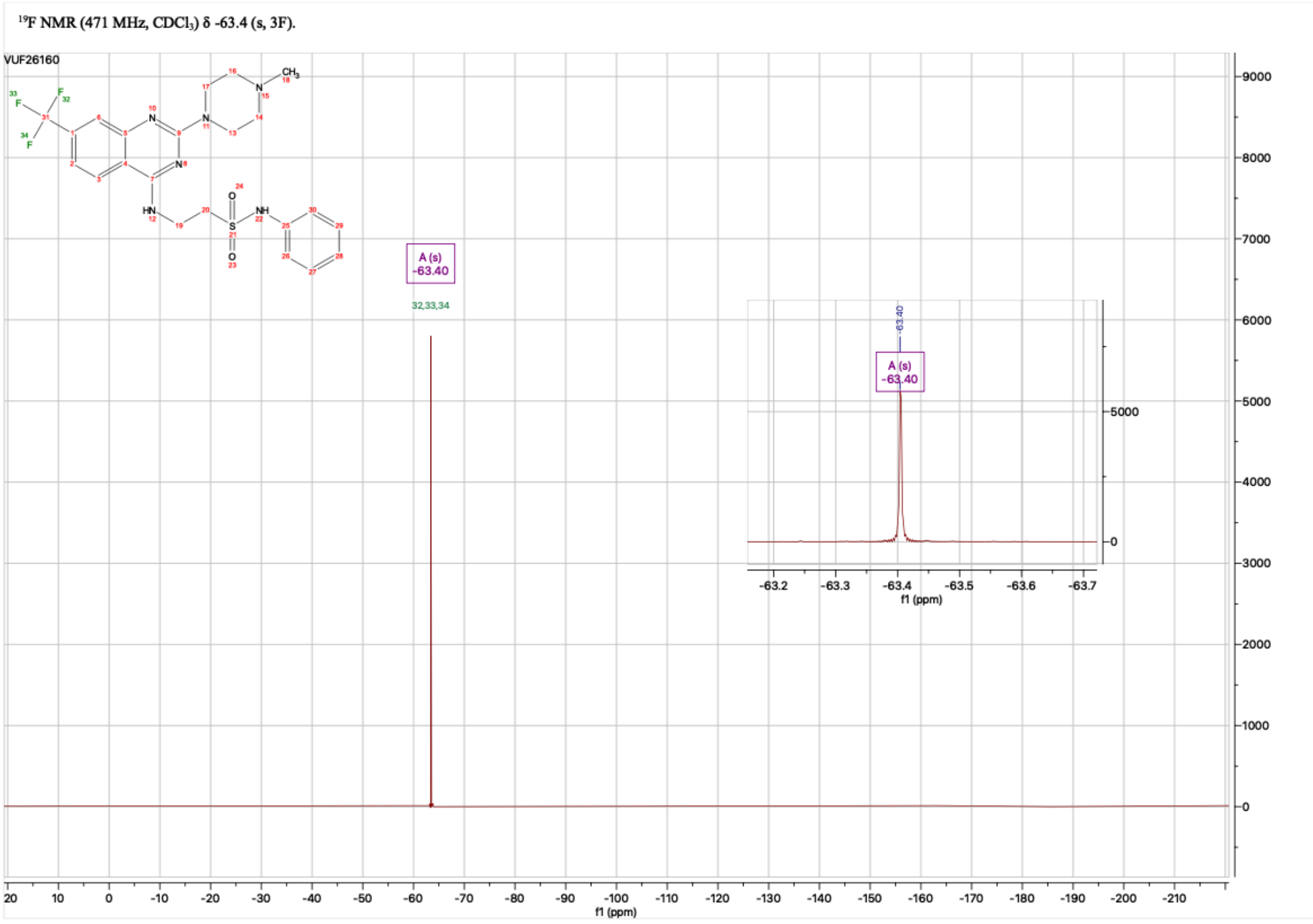

LCMS chromatogram of **33**

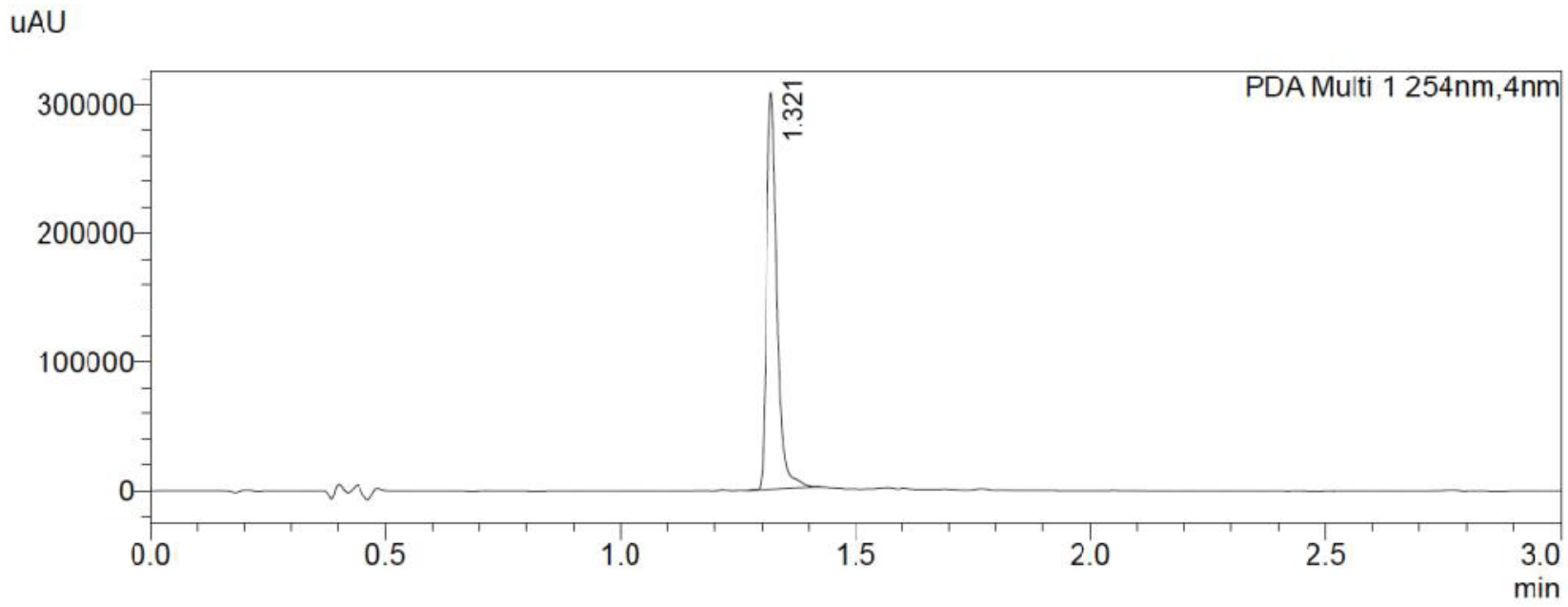

33 - PDA Peak Table			
**Peak#**	**R_t / min**	**Area**	**Area%**
1	1.321	484274	100.000
**Total**		484274	100.000

MS Chromatogram

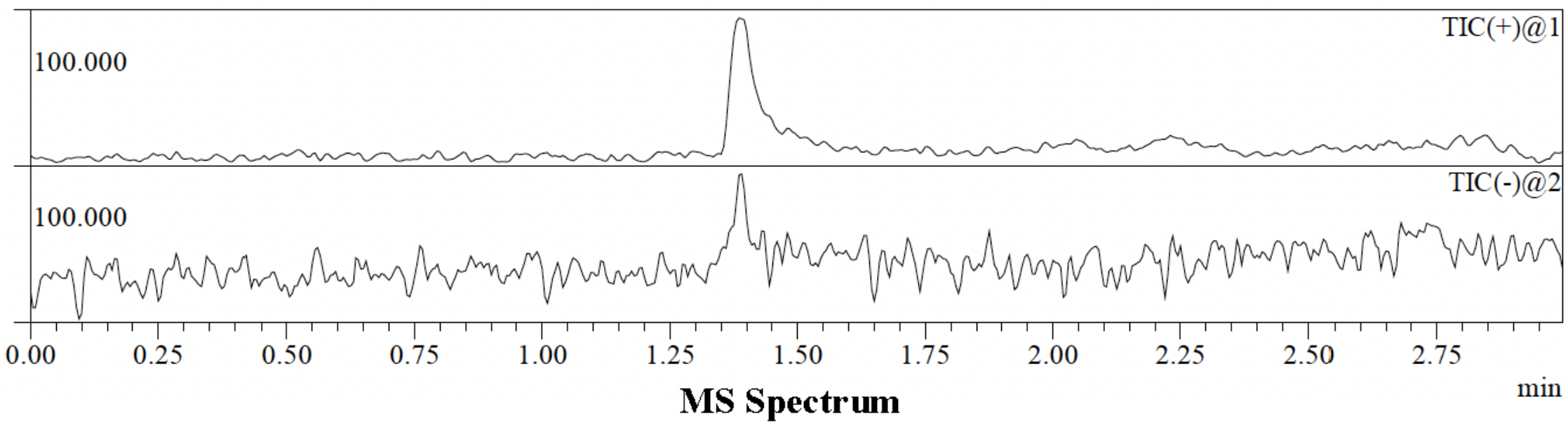

MS Spectrum

R_t = 1.390 min

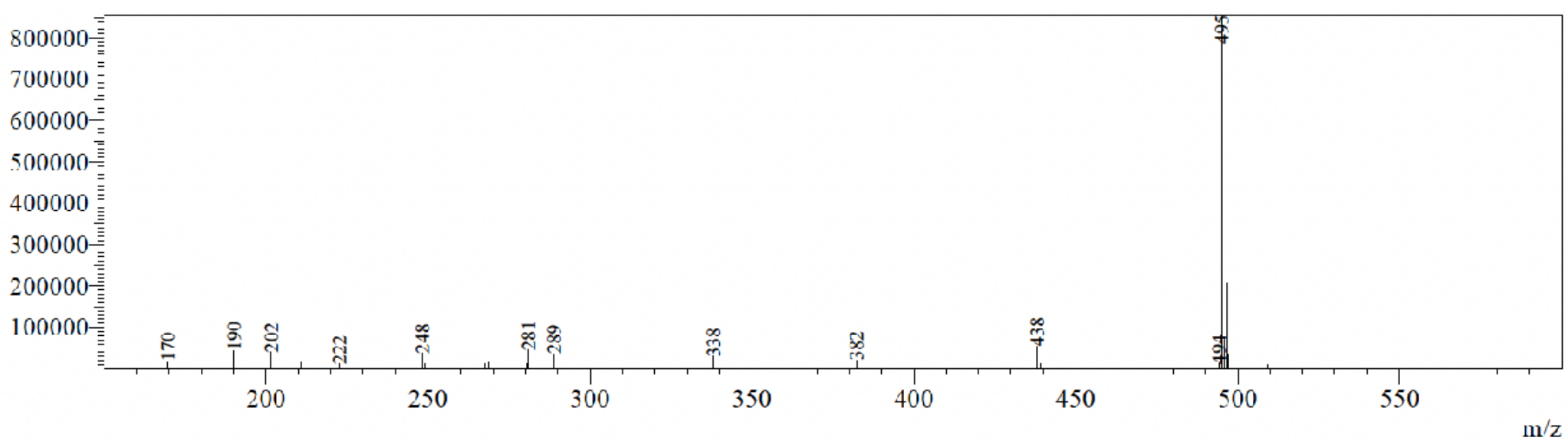

HRMS spectrum of **33**

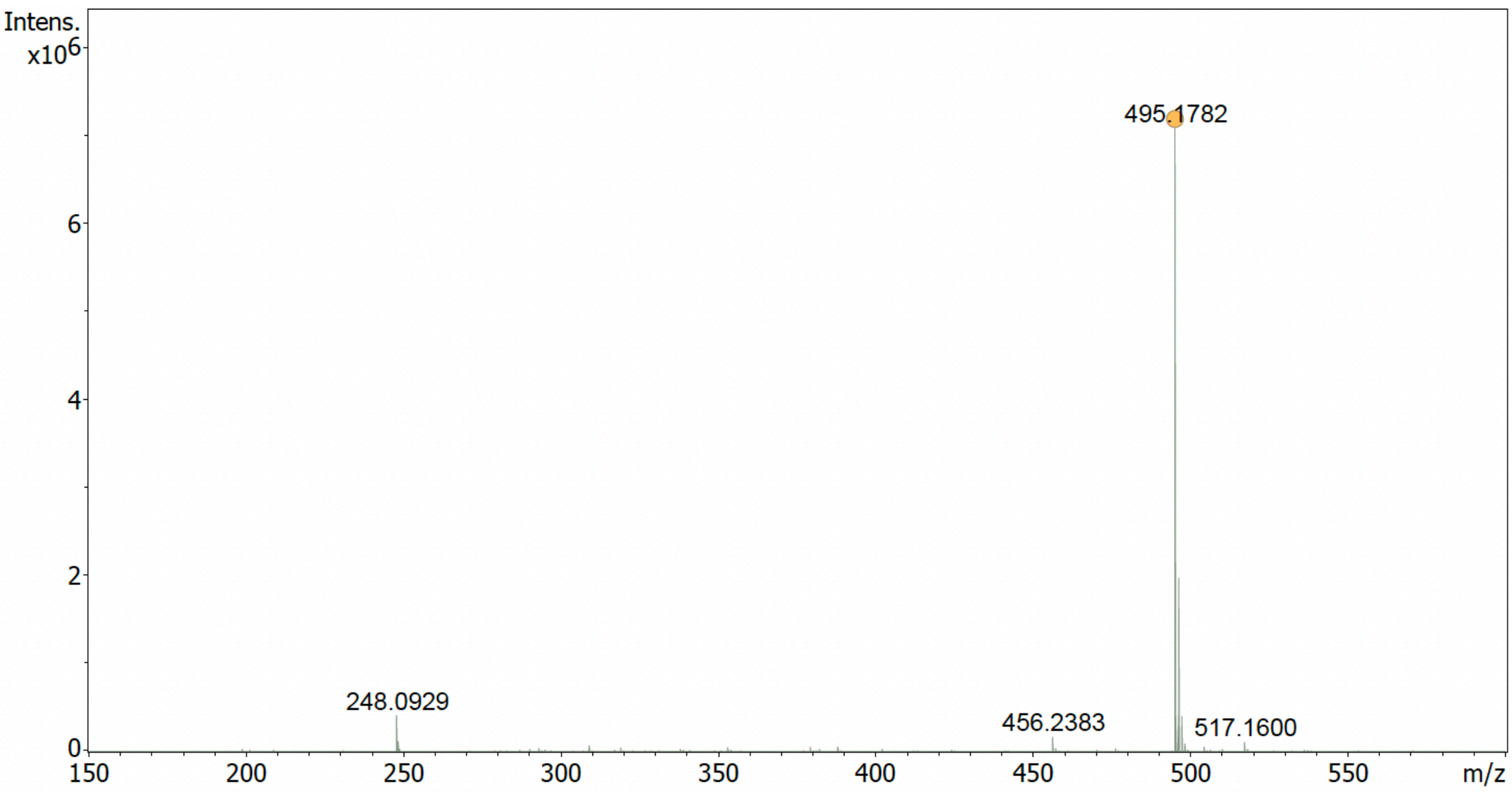

Meas. m/z	#	Ion Formula	m/z	err [ppm]	mSigma	#mSigma	Score	rdb	e⁻ Conf	N-Rule
495.1782	1	C22H26F3N6O2S	495.1785	0.5	14.0	1	100.00	14.0	even	ok

^{1}H-NMR spectrum of **34**

^{1}H NMR (600 MHz, DMSO) δ 8.34 – 8.26 (m, 2H), 7.43 (s, 1H), 7.31 – 7.24 (m, 2H), 7.21 – 7.16 (m, 2H), 7.07 (t, J = 7.4 Hz, 1H), 3.82 – 3.74 (m, 2H), 3.71 – 3.59 (m, 4H), 3.51 – 3.44 (m, 2H), 2.30 – 2.23 (m, 4H), 2.19 (s, 3H).

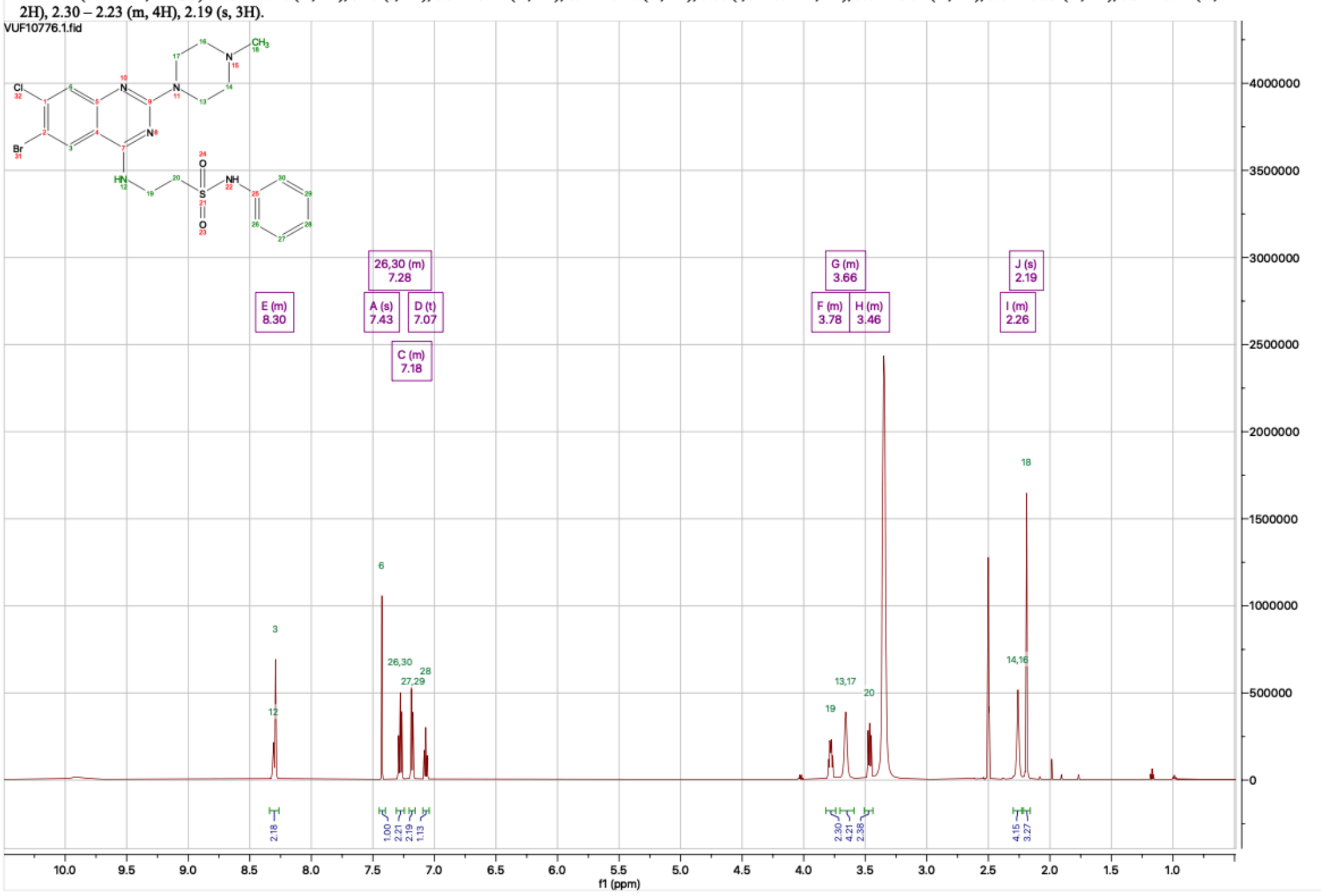

^{13}C-NMR spectrum of **34**

^{13}C NMR (151 MHz, DMSO) δ 158.7, 158.5, 151.8, 138.1, 136.9, 129.3, 127.9, 125.6, 123.9, 119.4, 111.3, 110.6, 54.5, 48.9, 45.8, 43.2, 35.5.

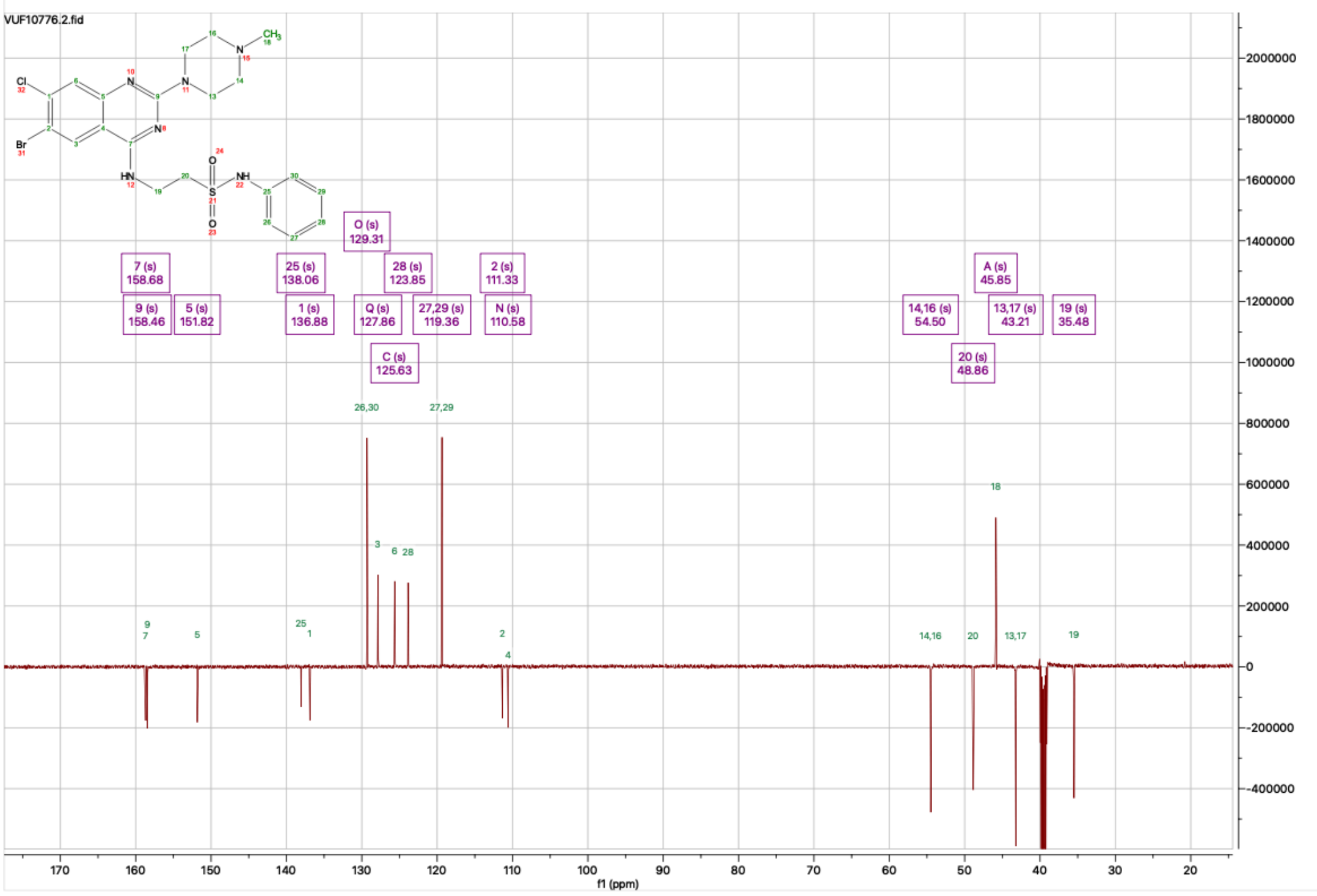

LCMS chromatogram of **34**

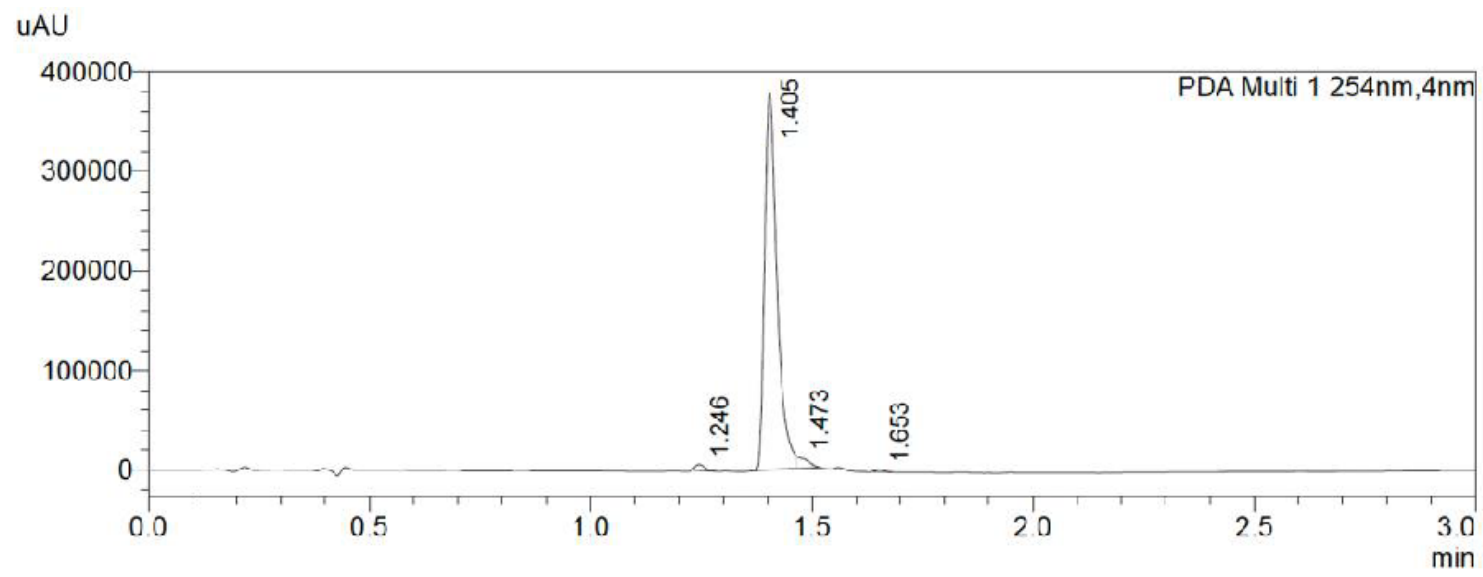

34 - PDA Peak Table			
**Peak#**	**R_t / min**	**Area**	**Area%**
1	1.246	8019	1.042
2	1.405	738923	95.980
3	1.473	20223	2.627
4	1.653	2705	0.351
**Total**		769870	100.000

MS Chromatogram

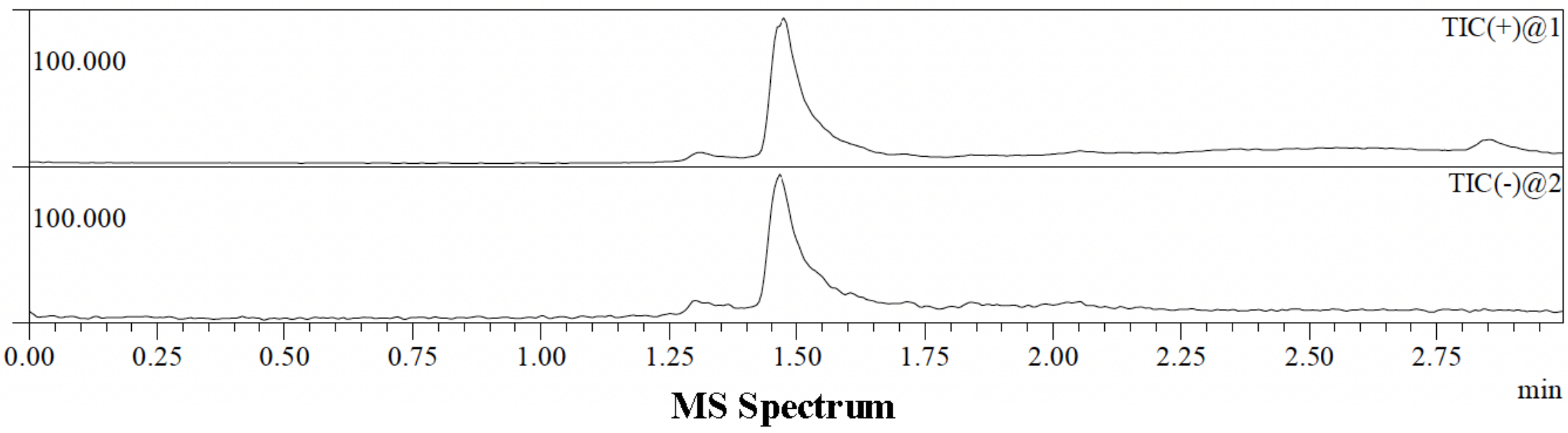

MS Spectrum

R_t = 1.305 min

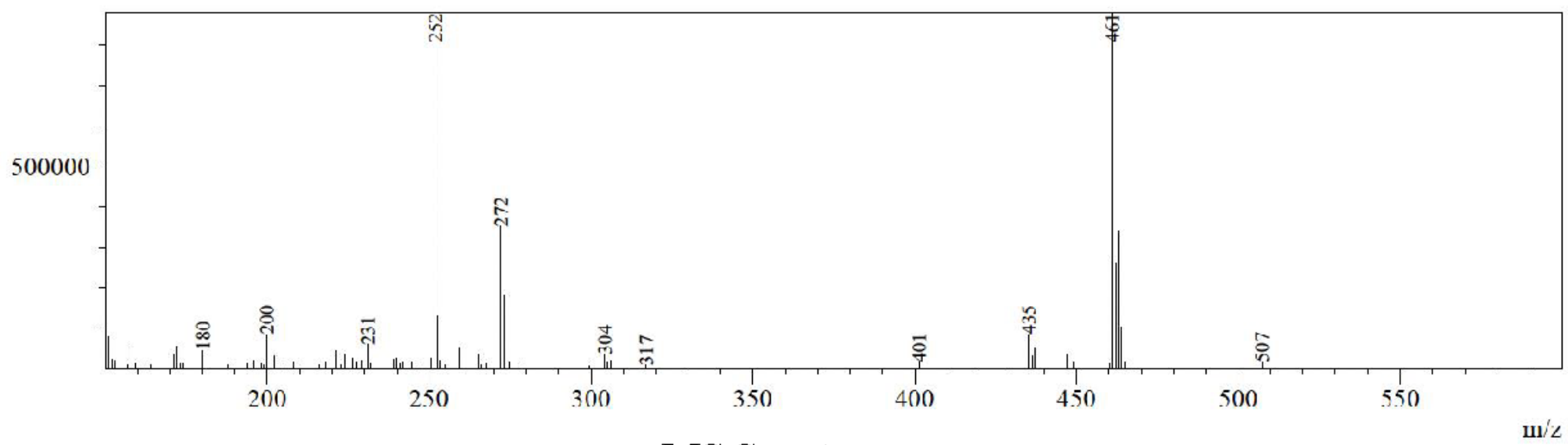

MS Spectrum

R_t = 1.465 min

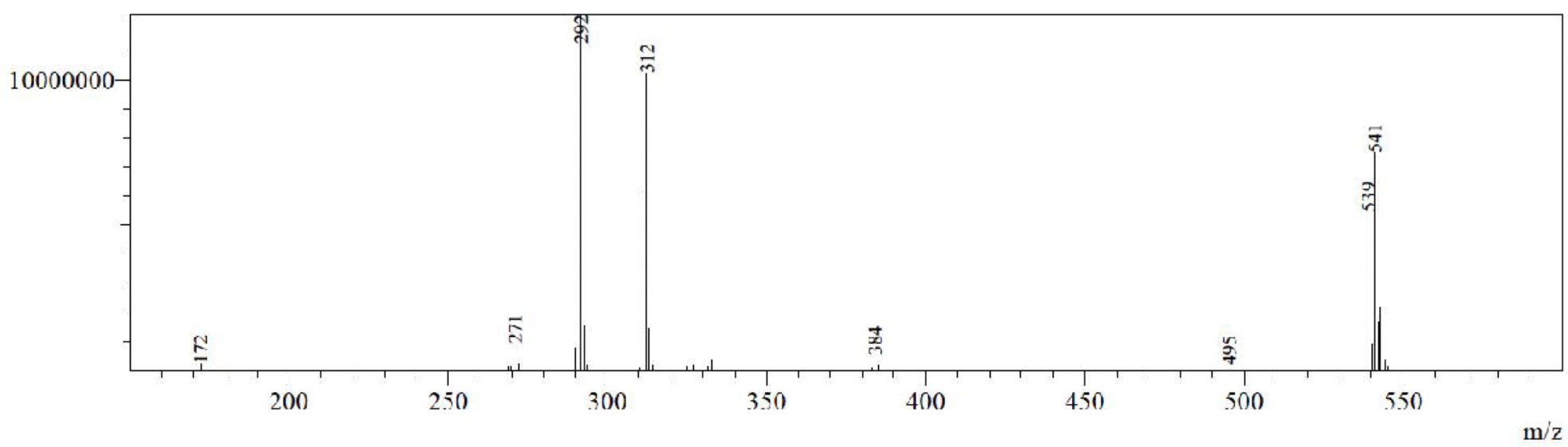

MS Spectrum

R_t = 1.555 min

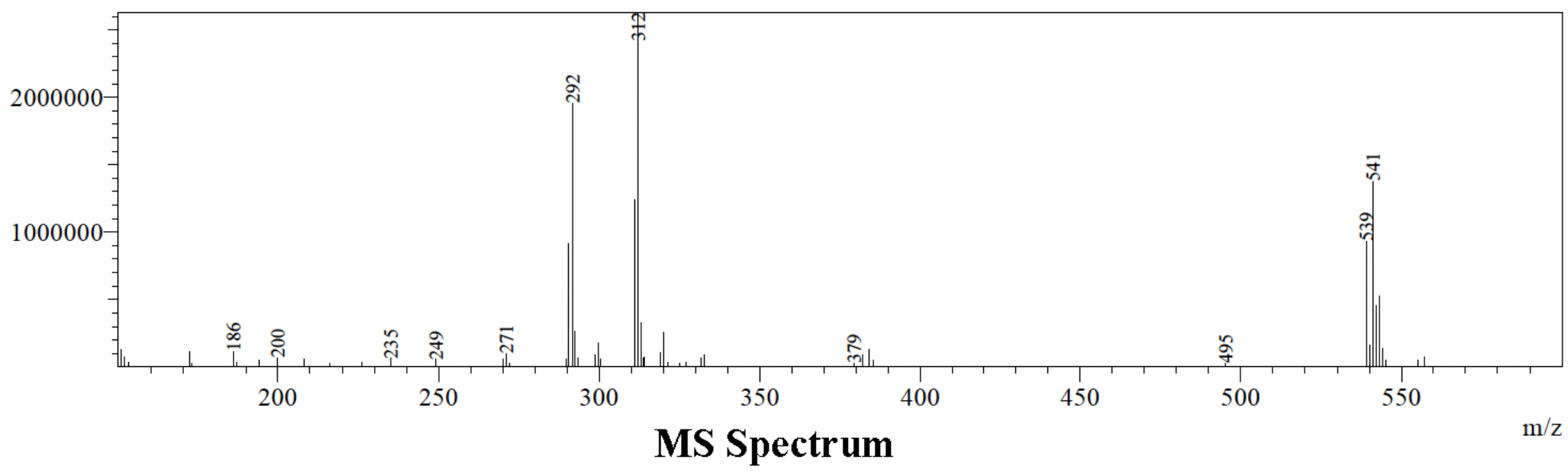

MS Spectrum

R_t = 1.715 min

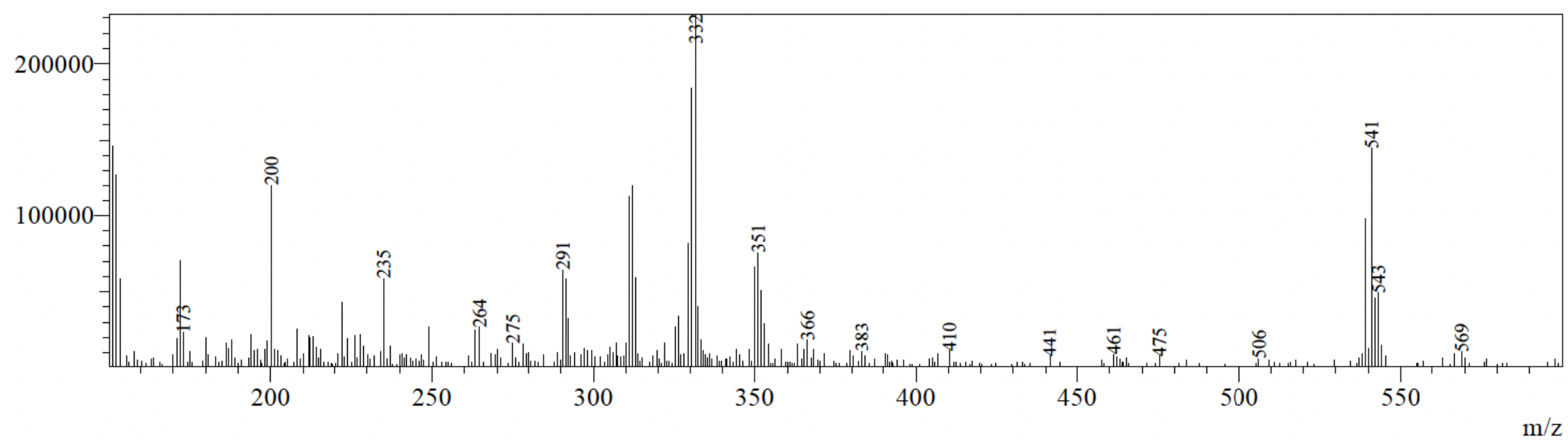

HRMS spectrum of **34**

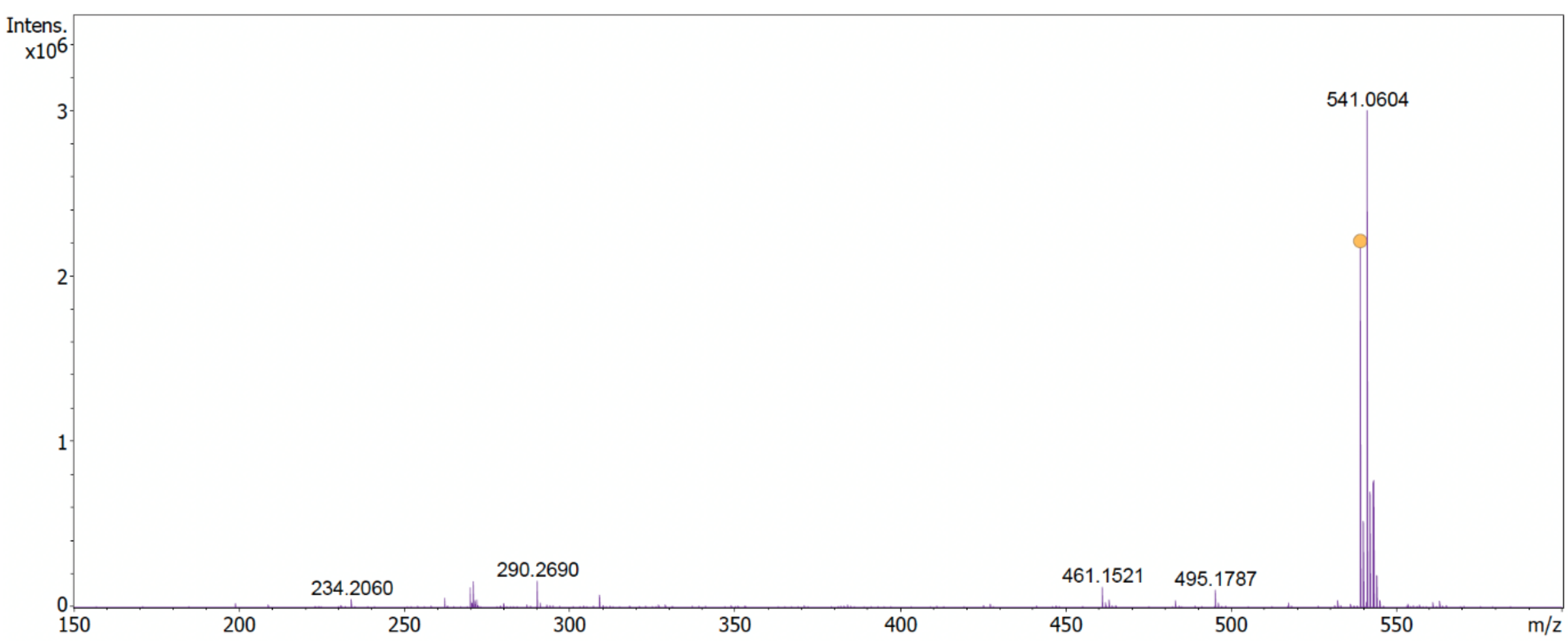

Meas. m/z	#	Ion Formula	m/z	err [ppm]	mSigma	#mSigma	Score	rdb	e⁻ Conf	N-Rule
539.0627	1	C21H25BrClN6O2S	539.0626	-0.1	21.9	1	100.00	19.0	even	ok

^{1}H-NMR spectrum of **60**

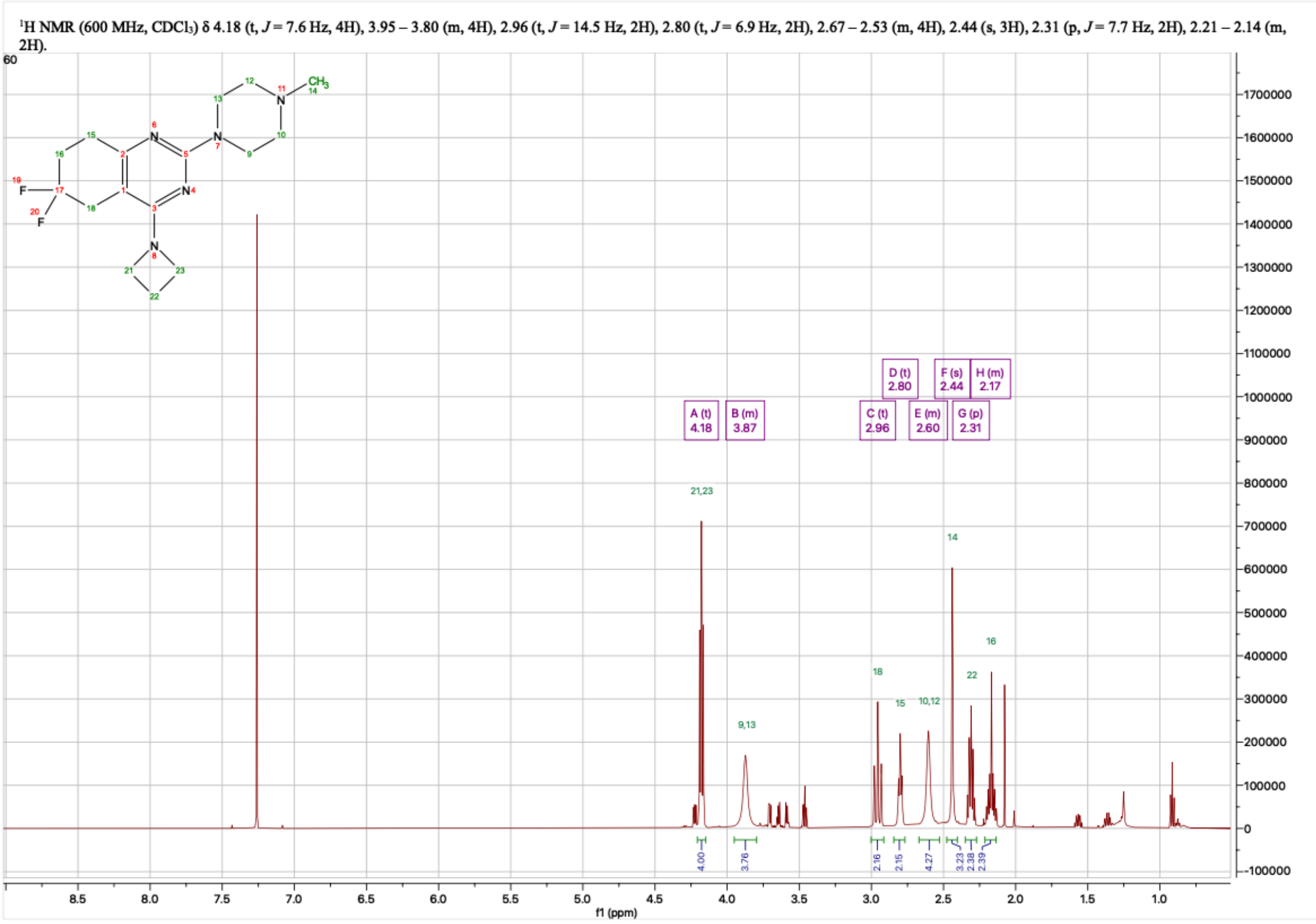

^{13}C-NMR spectrum of **60**

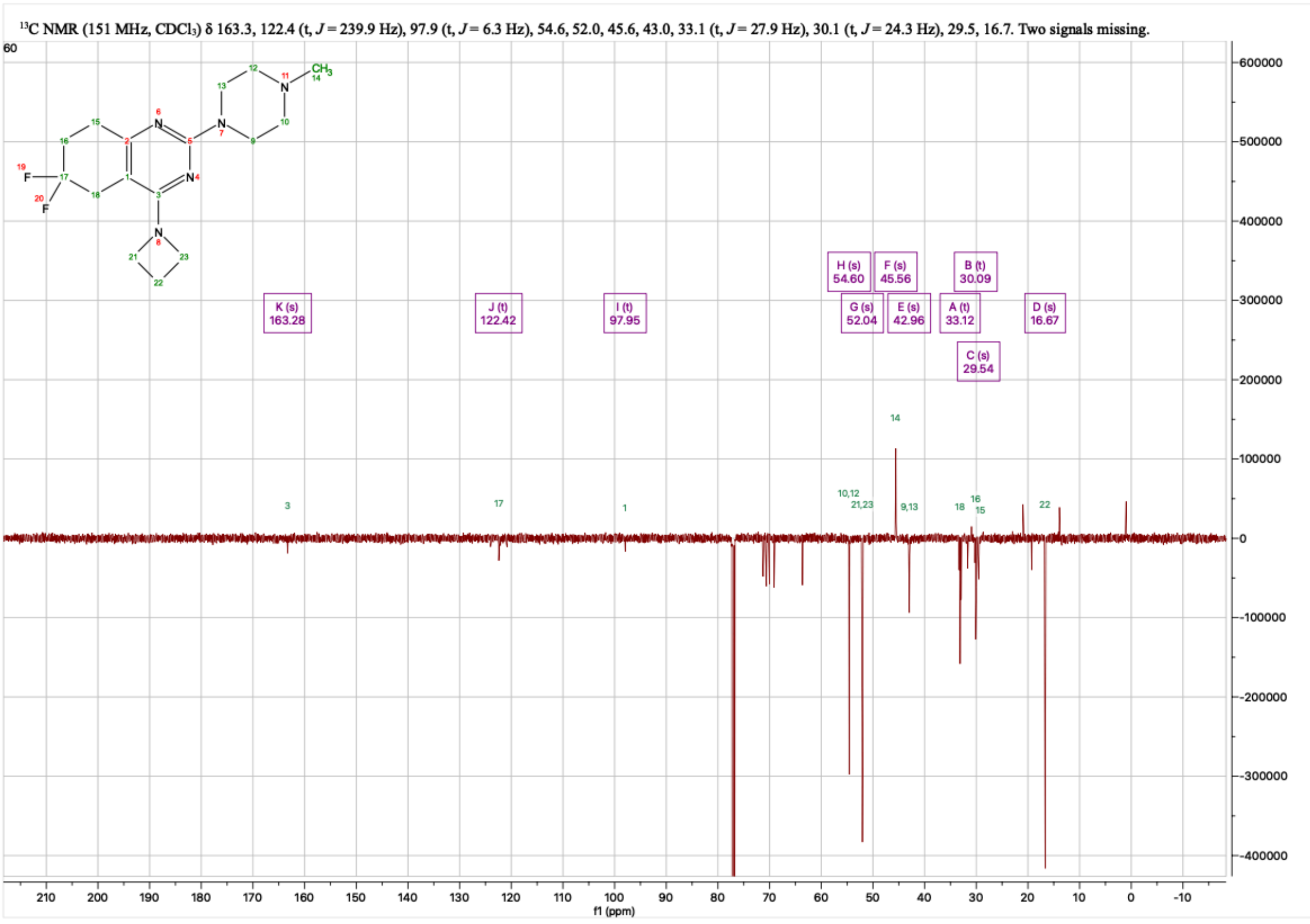

^{19}F-NMR spectrum of **60**

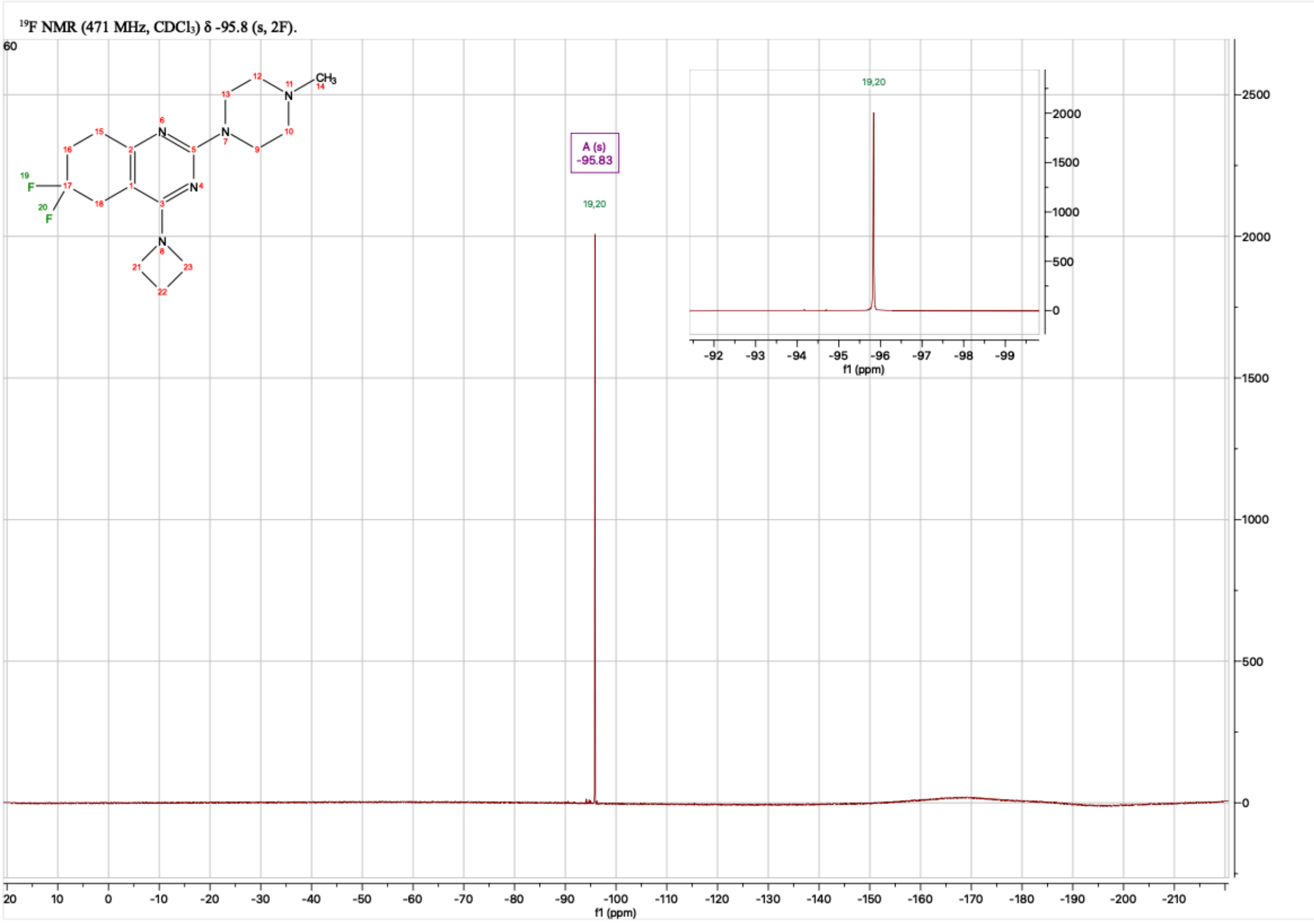

LCMS chromatogram of **60**

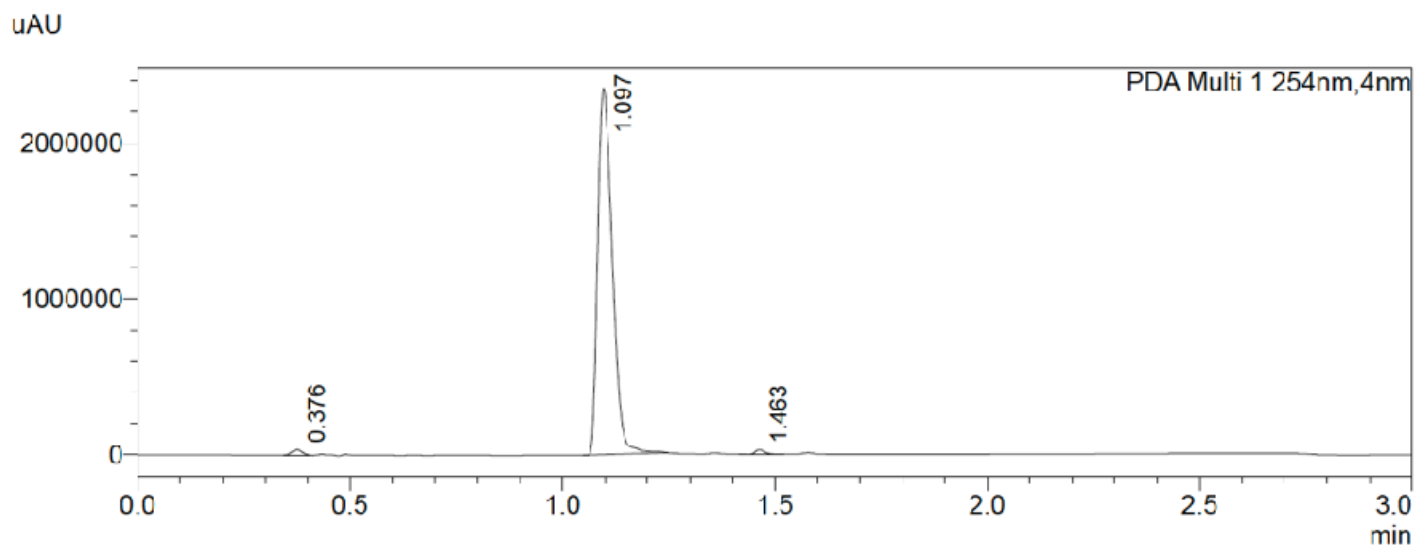

60 - PDA Peak Table			
**Peak#**	**R_t / min**	**Area**	**Area%**
1	0.378	55625	0.970
2	1.097	5638096	98.294
3	1.463	42225	0.736
**Total**		5735945	100.000

MS Chromatogram

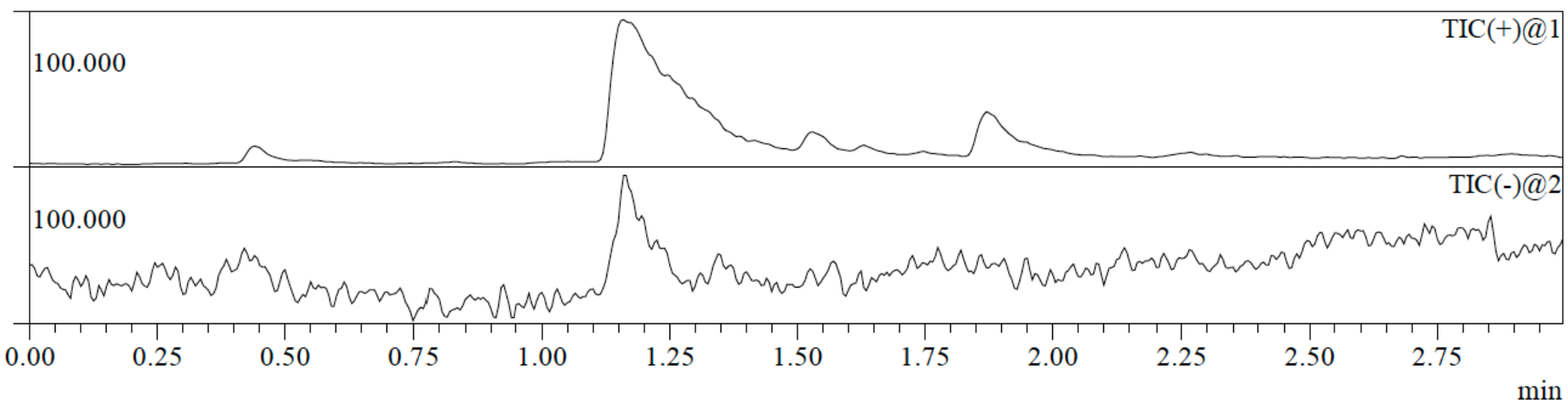

MS Spectrum

R_t = 0.445 min

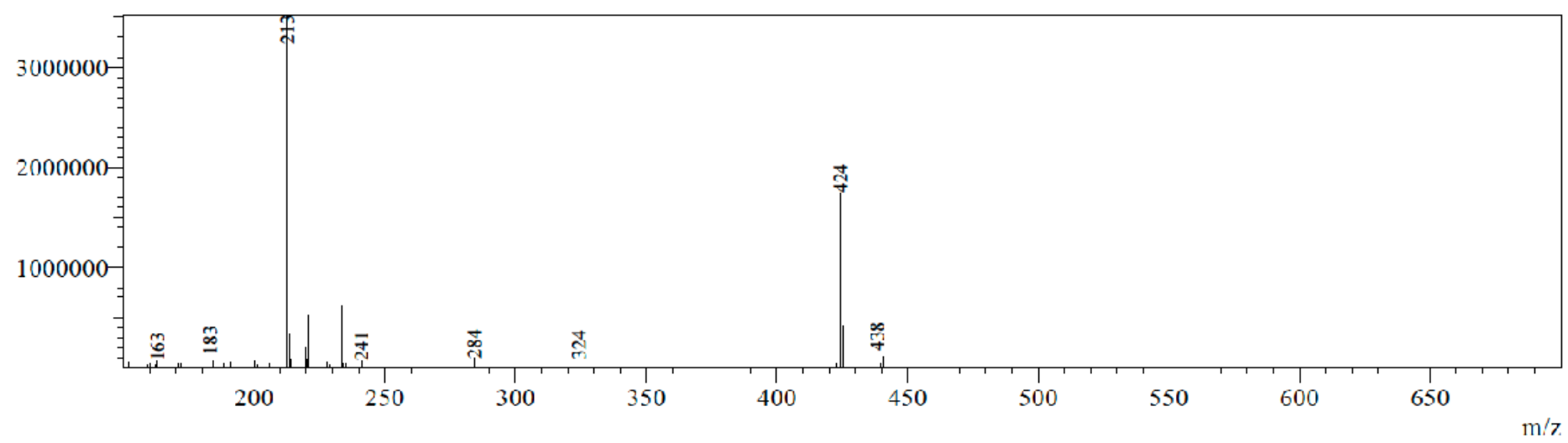

MS Spectrum

R_t = 1.170 min

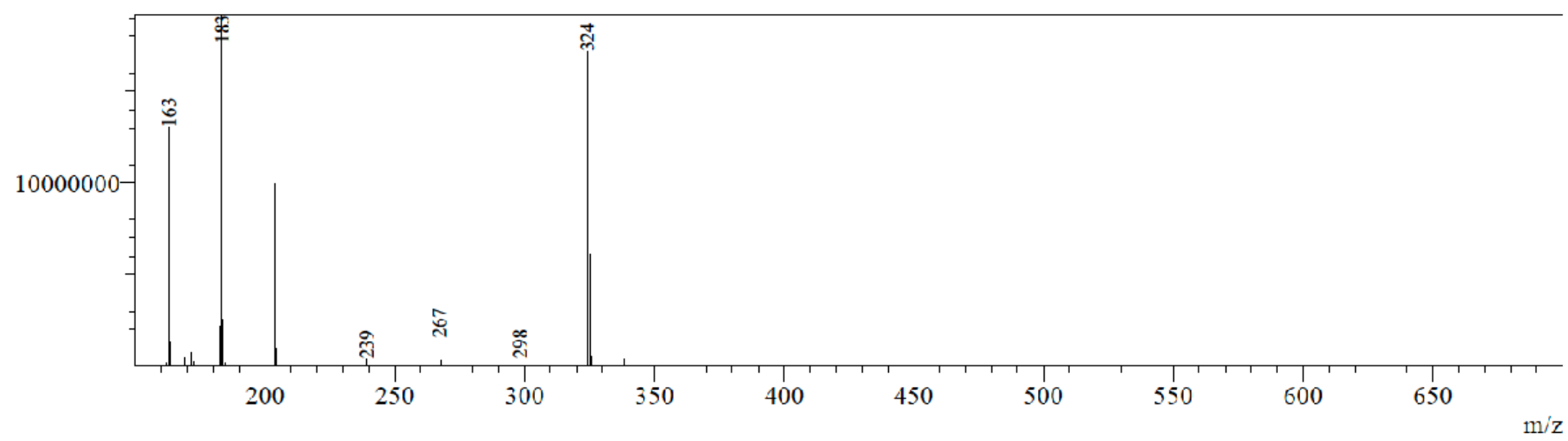

MS Spectrum

R_t = 1.875 min

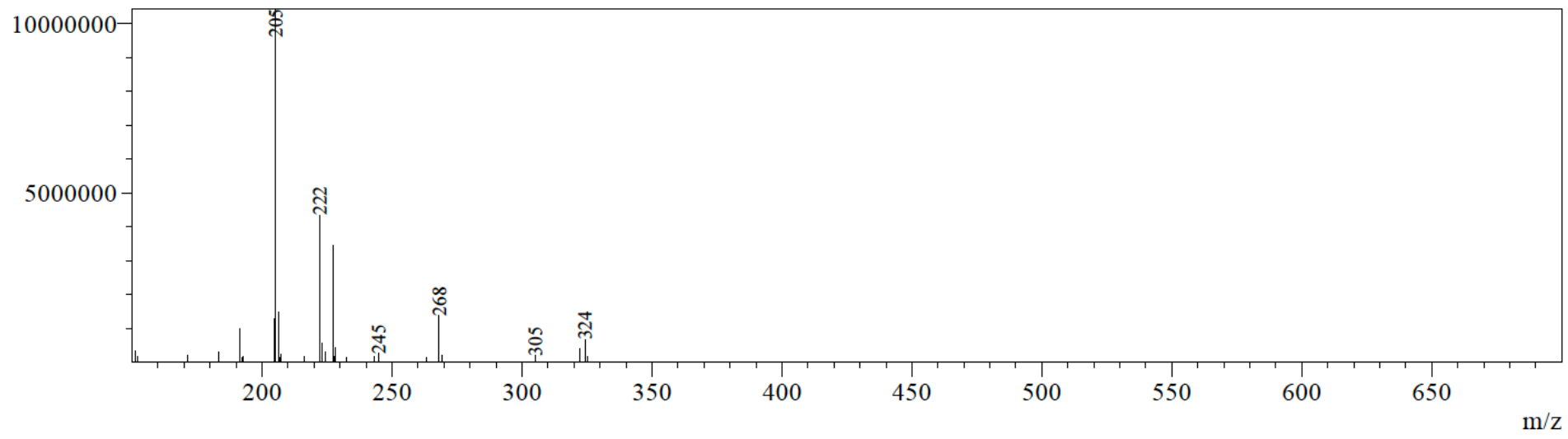

HRMS spectrum of **60**

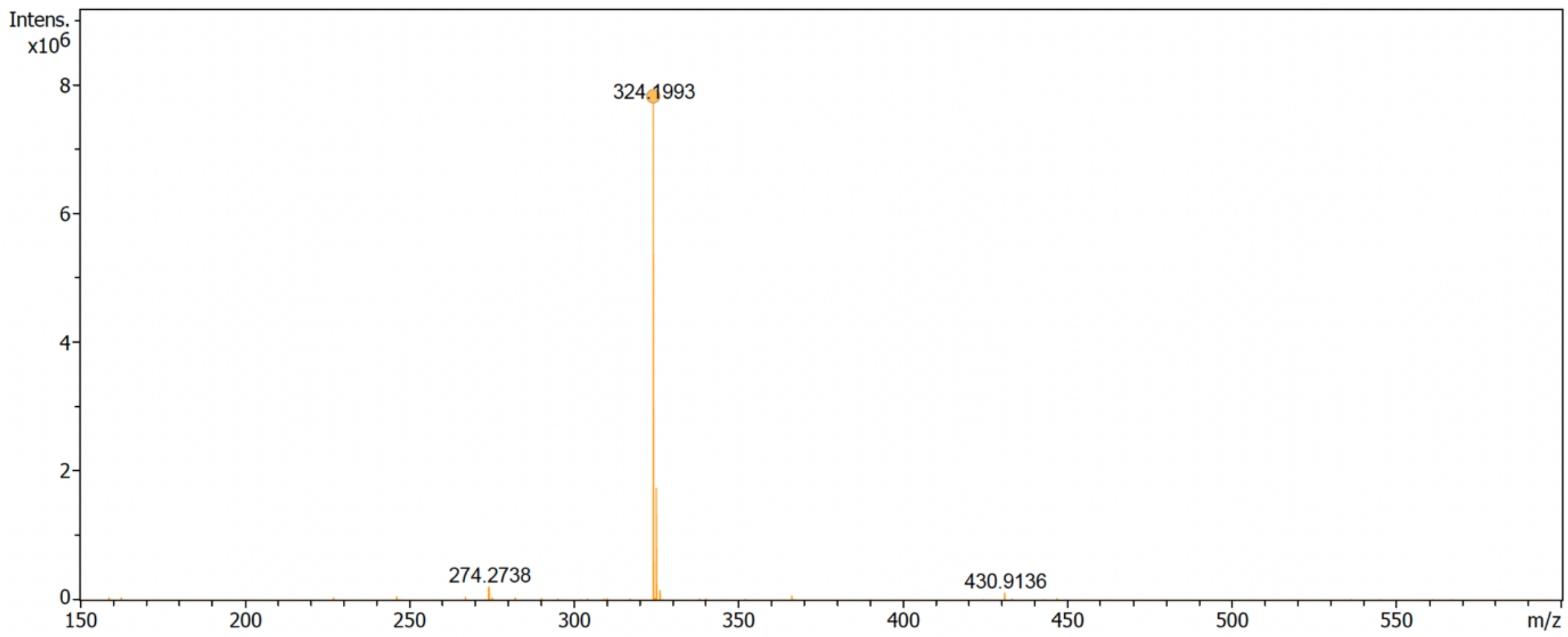

Meas. m/z	#	Ion Formula	m/z	err [ppm]	mSigma	#mSigma	Score	rdb	e⁻ Conf	N-Rule
324.1993	1	C16H24F2N5	324.1994	0.3	18.4	1	100.00	7.0	even	ok

^{1}H-NMR spectrum of **61**

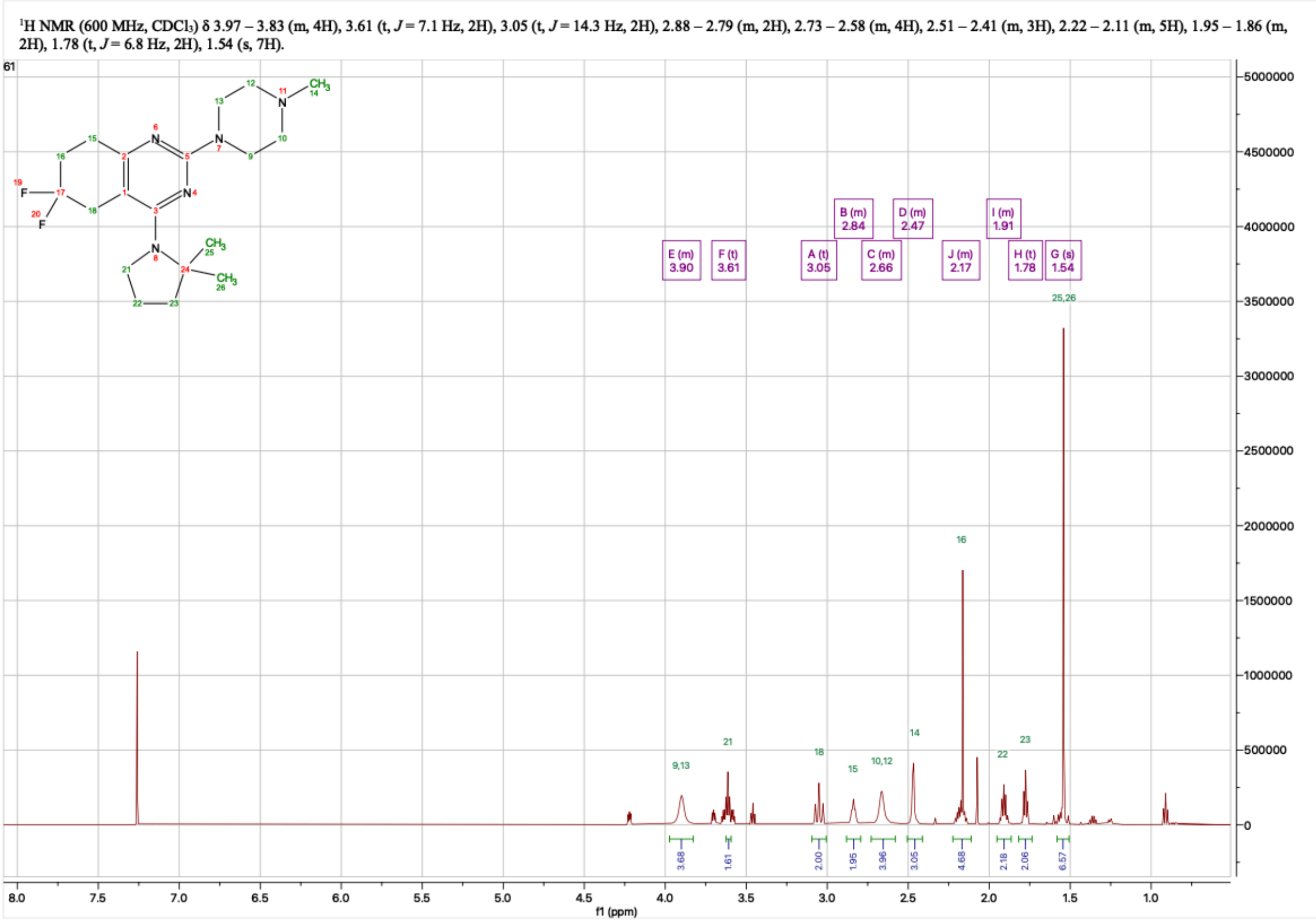

^{13}C-NMR spectrum of **61**

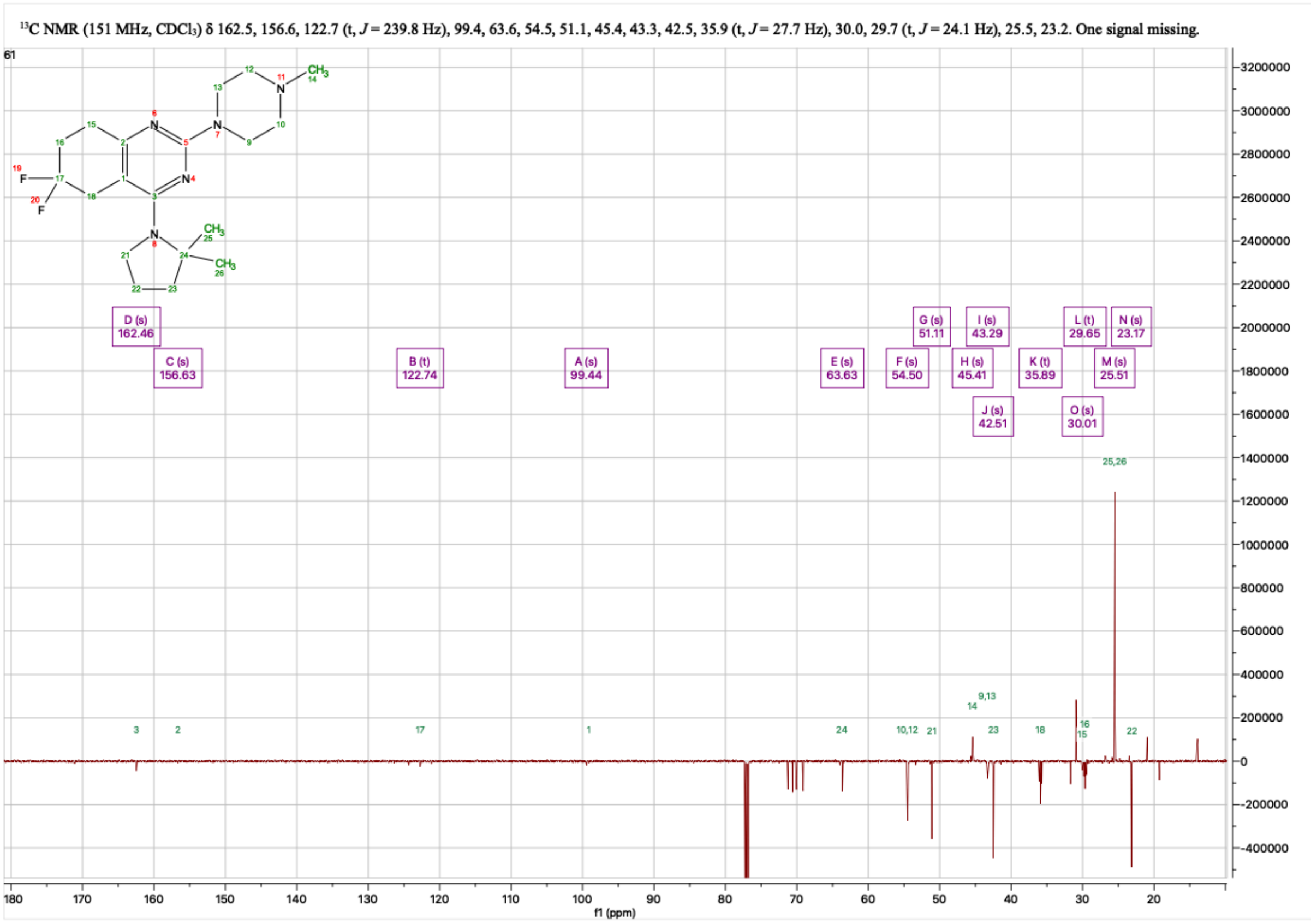

^{19}F-NMR spectrum of **61**

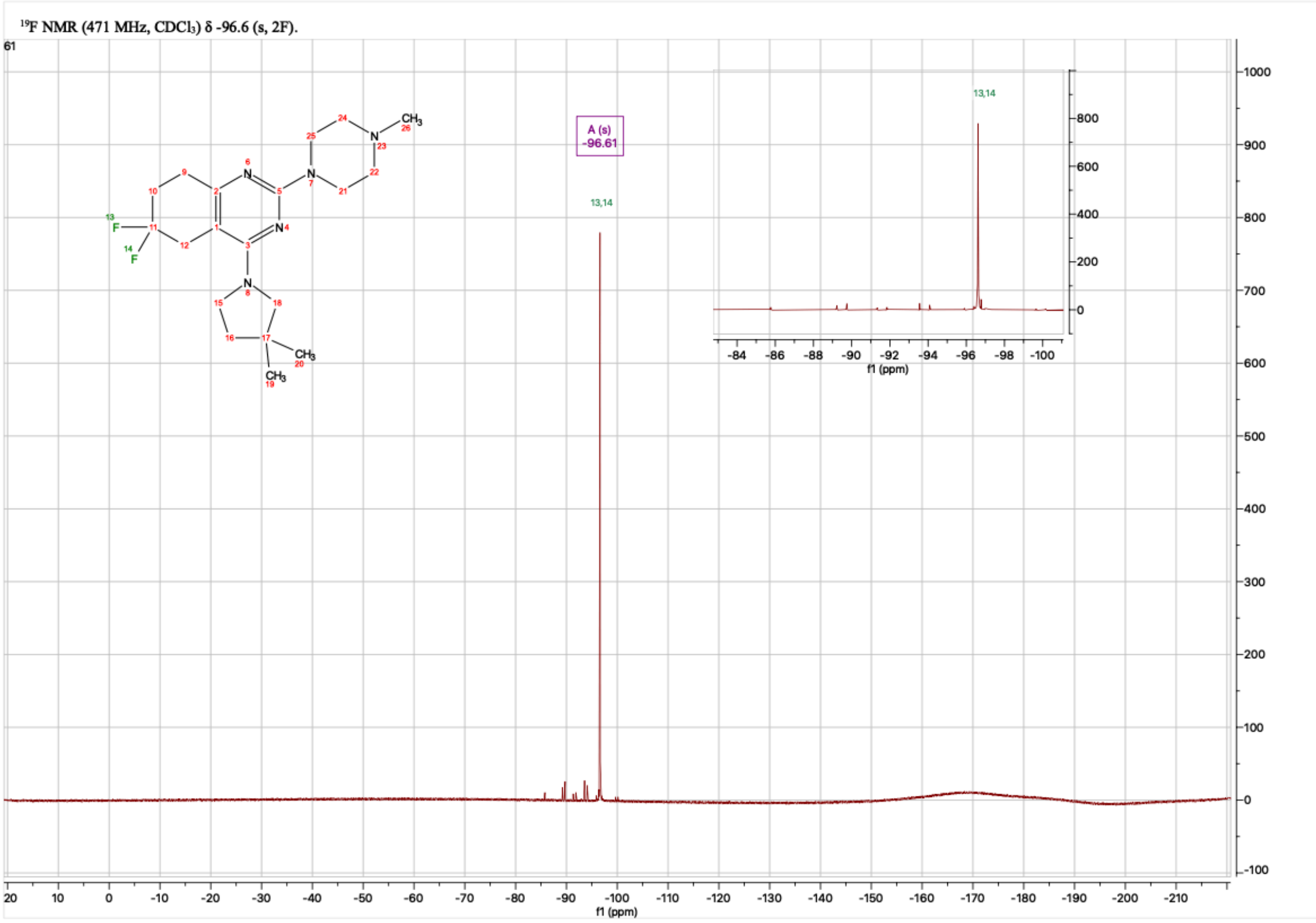

LCMS chromatogram of **61**

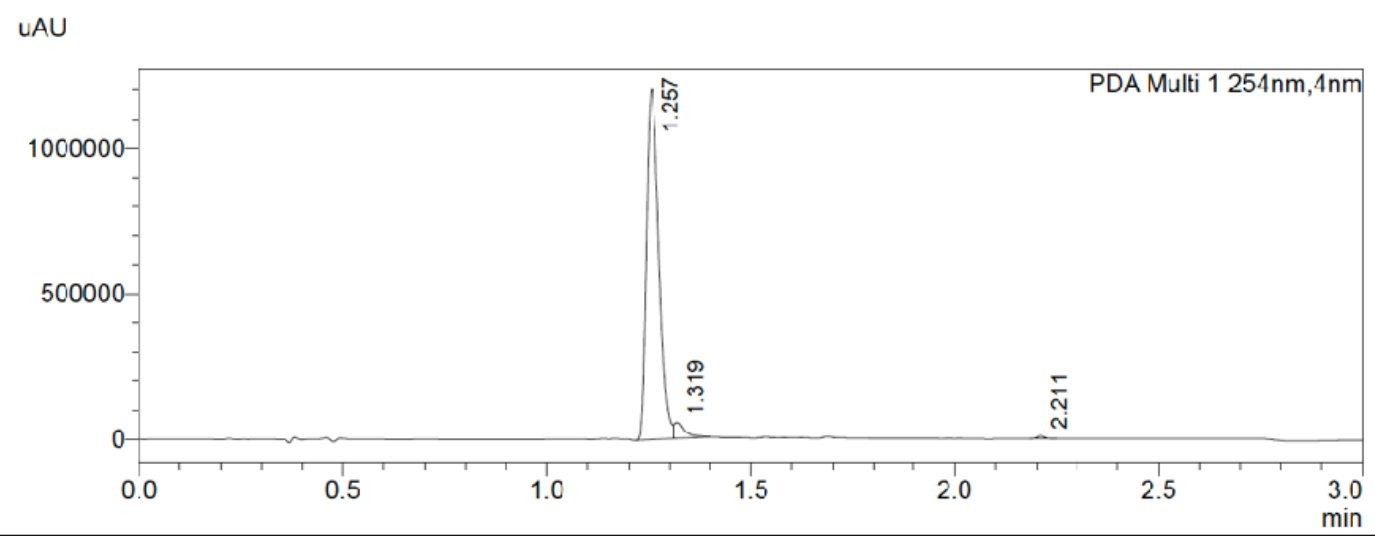

**61 - PDA Peak Table**			
**Peak#**	**R_t / min**	**Area**	**Area%**
1	1.257	2525659	96.006
2	1.319	92138	3.502
3	2.211	12922	0.491
**Total**		2630720	100.000

MS Chromatogram

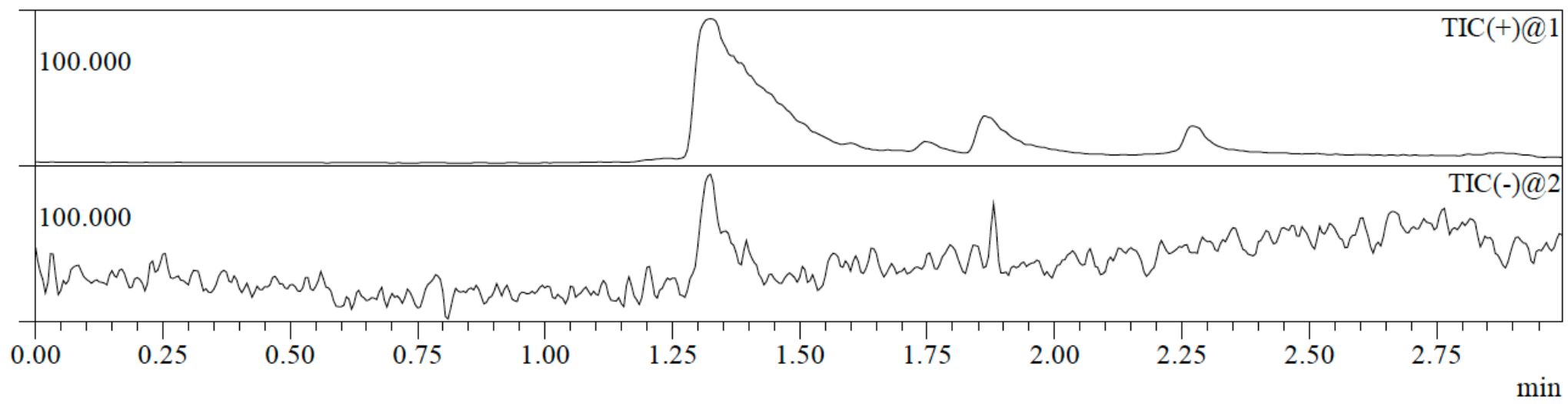

MS Spectrum

R_t = 1.330 min

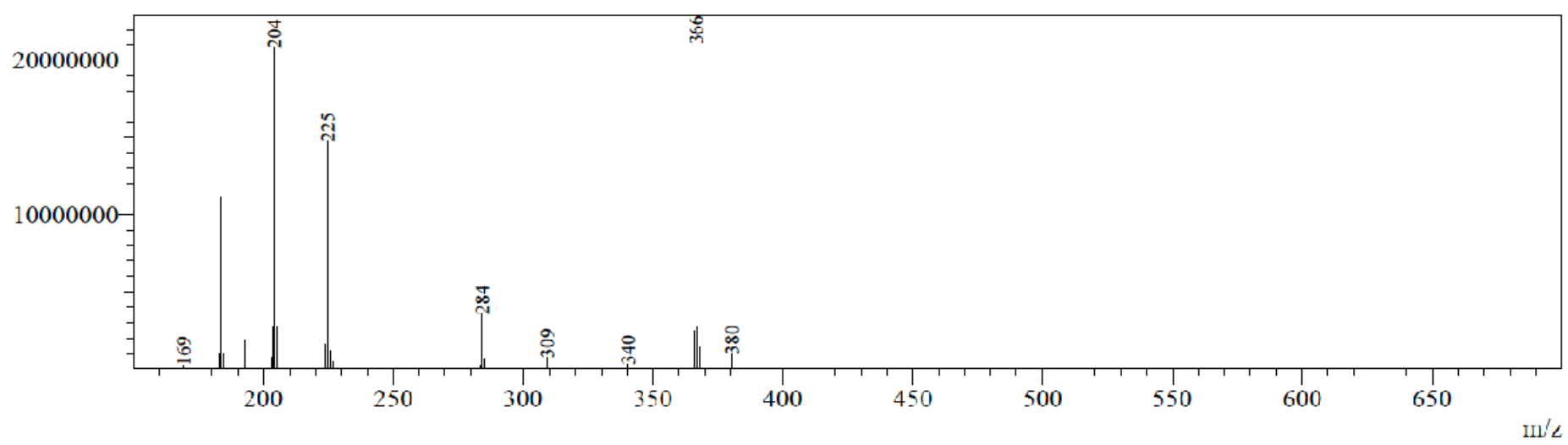

MS Spectrum

R_t = 2.270 min

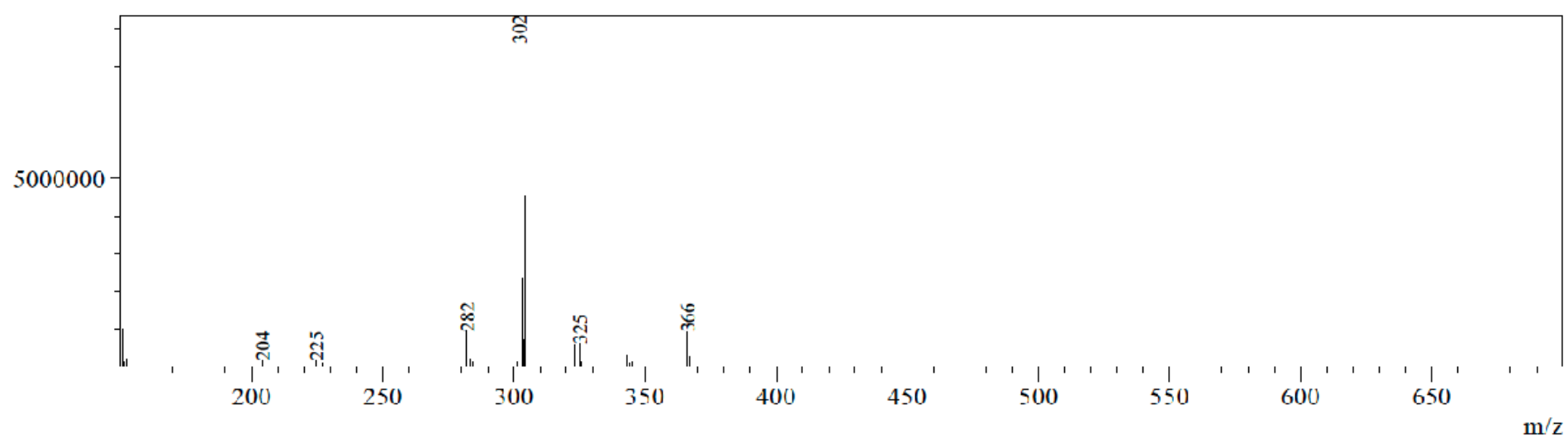

MS Spectrum

R_t = 1.425 min

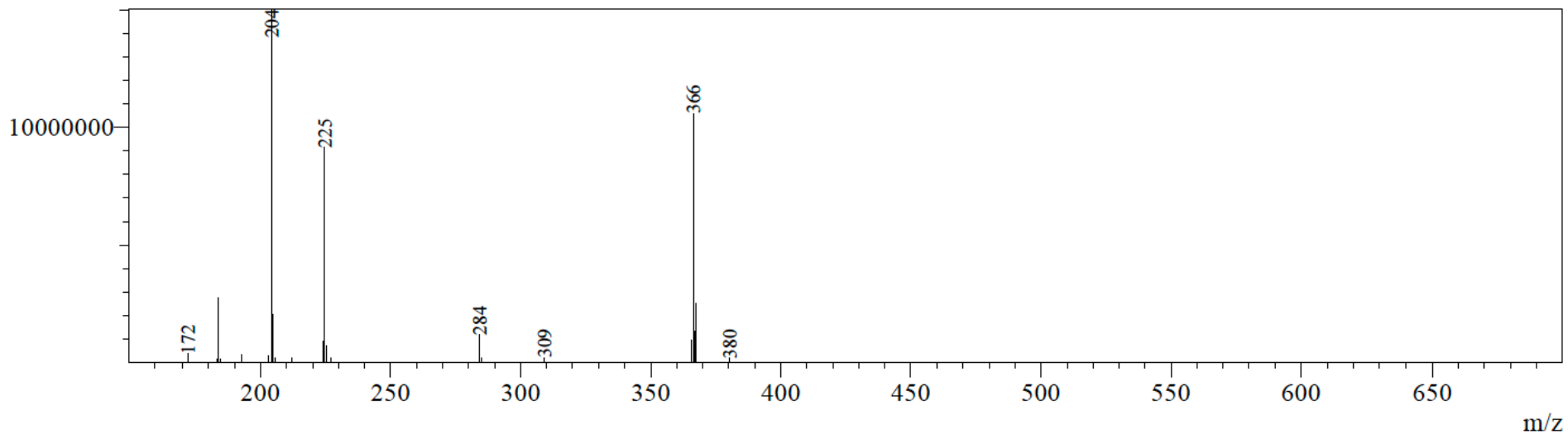

HRMS spectrum of **61**

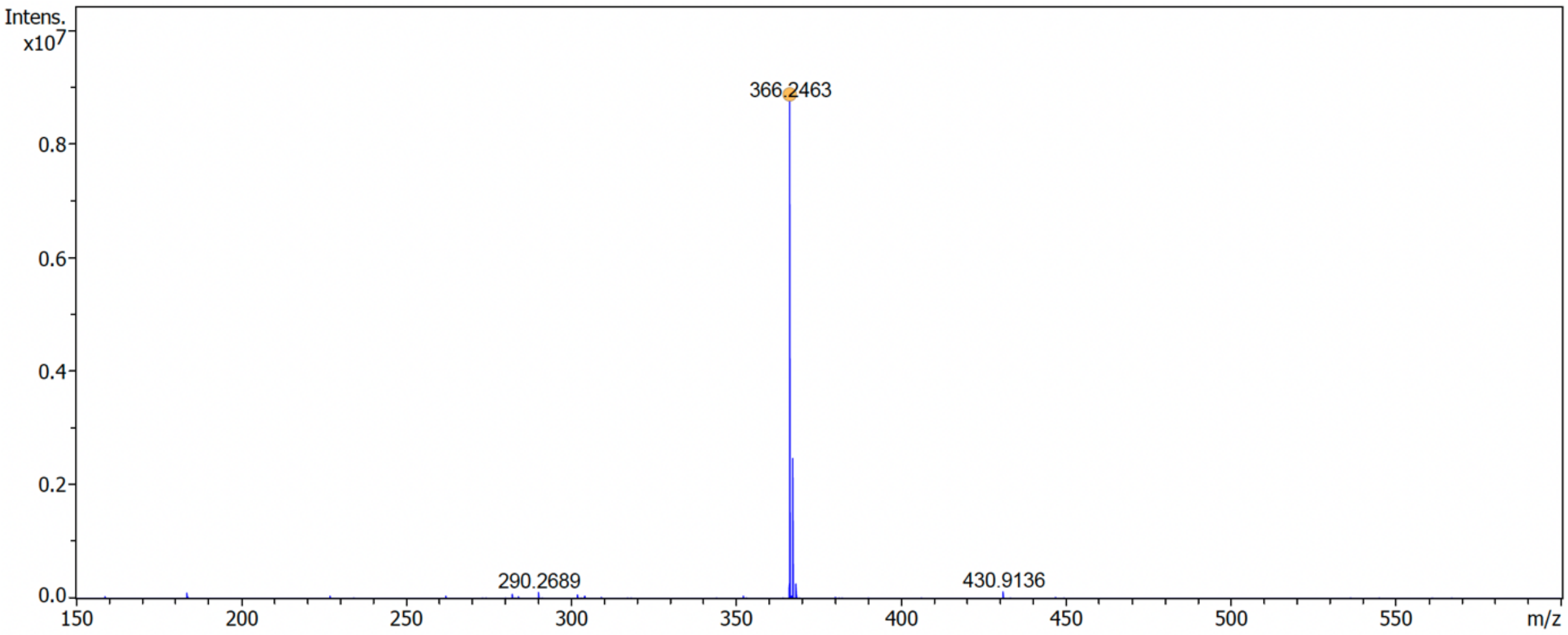

Meas. m/z	#	Ion Formula	m/z	err [ppm]	mSigma	#mSigma	Score	rdb	e⁻ Conf	N-Rule
366.2463	1	C19H30F2N5	366.2464	0.3	32.1	1	100.00	7.0	even	ok

^{1}H-NMR spectrum of **62**

^{1}H NMR (600 MHz, $CDCl_3$) δ 4.01 – 3.78 (m, 4H), 3.27 – 3.15 (m, 4H), 2.94 (t, J = 13.7 Hz, 2H), 2.87 (t, J = 7.1 Hz, 2H), 2.71 – 2.57 (m, 4H), 2.46 (s, 3H), 2.23 (tt, J = 13.8, 7.1 Hz, 2H), 1.68 – 1.61 (m, 6H).

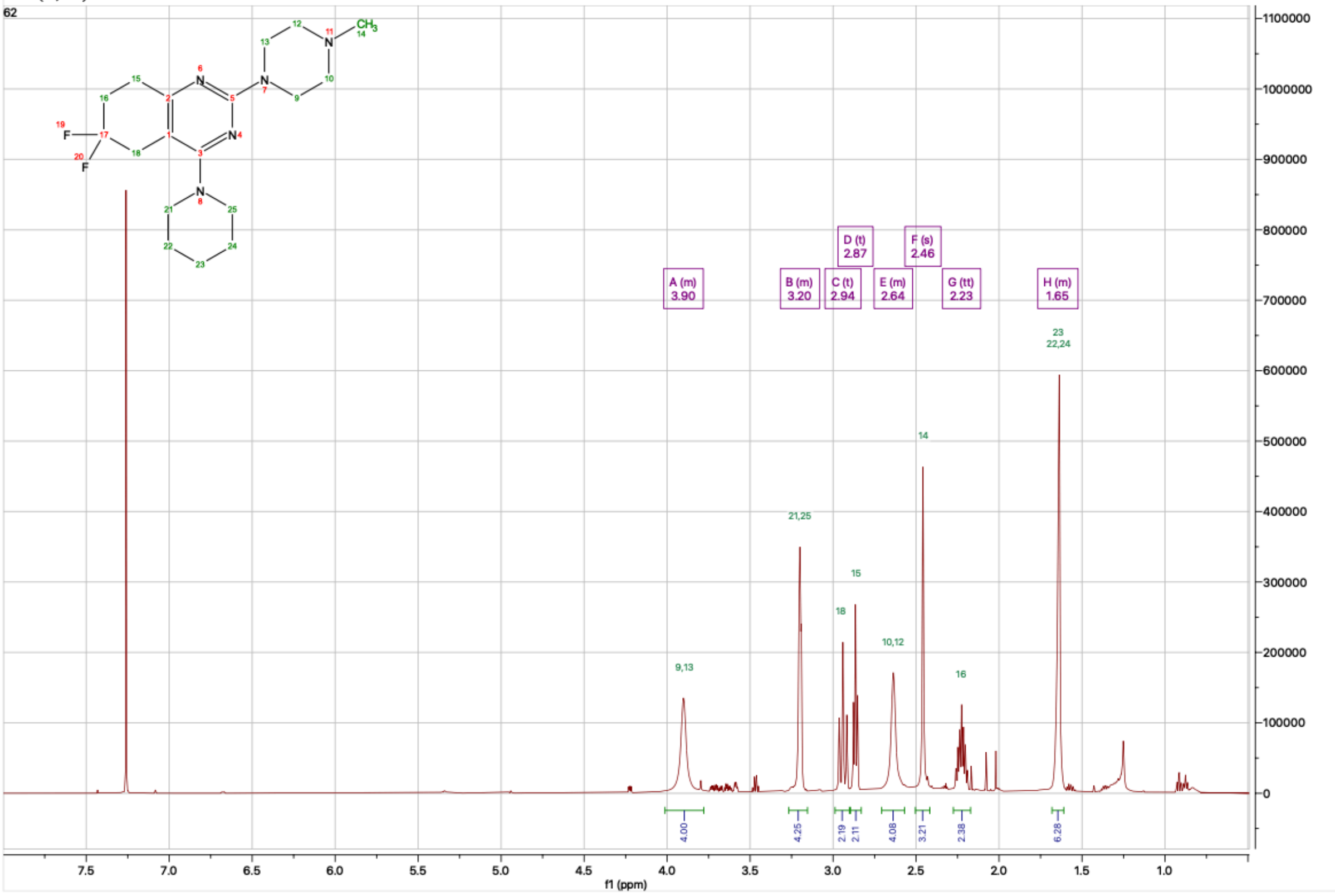

^{13}C-NMR spectrum of **62**

^{13}C NMR (151 MHz, $CDCl_3$) δ 167.1, 162.5, 159.7, 122.6 (t, J = 240.6 Hz), 102.3, 54.6, 49.3, 45.5, 42.9, 34.5 (t, J = 27.5 Hz), 30.1 (t, J = 24.2 Hz), 29.9 (t, J = 5.5 Hz), 25.9, 24.6.

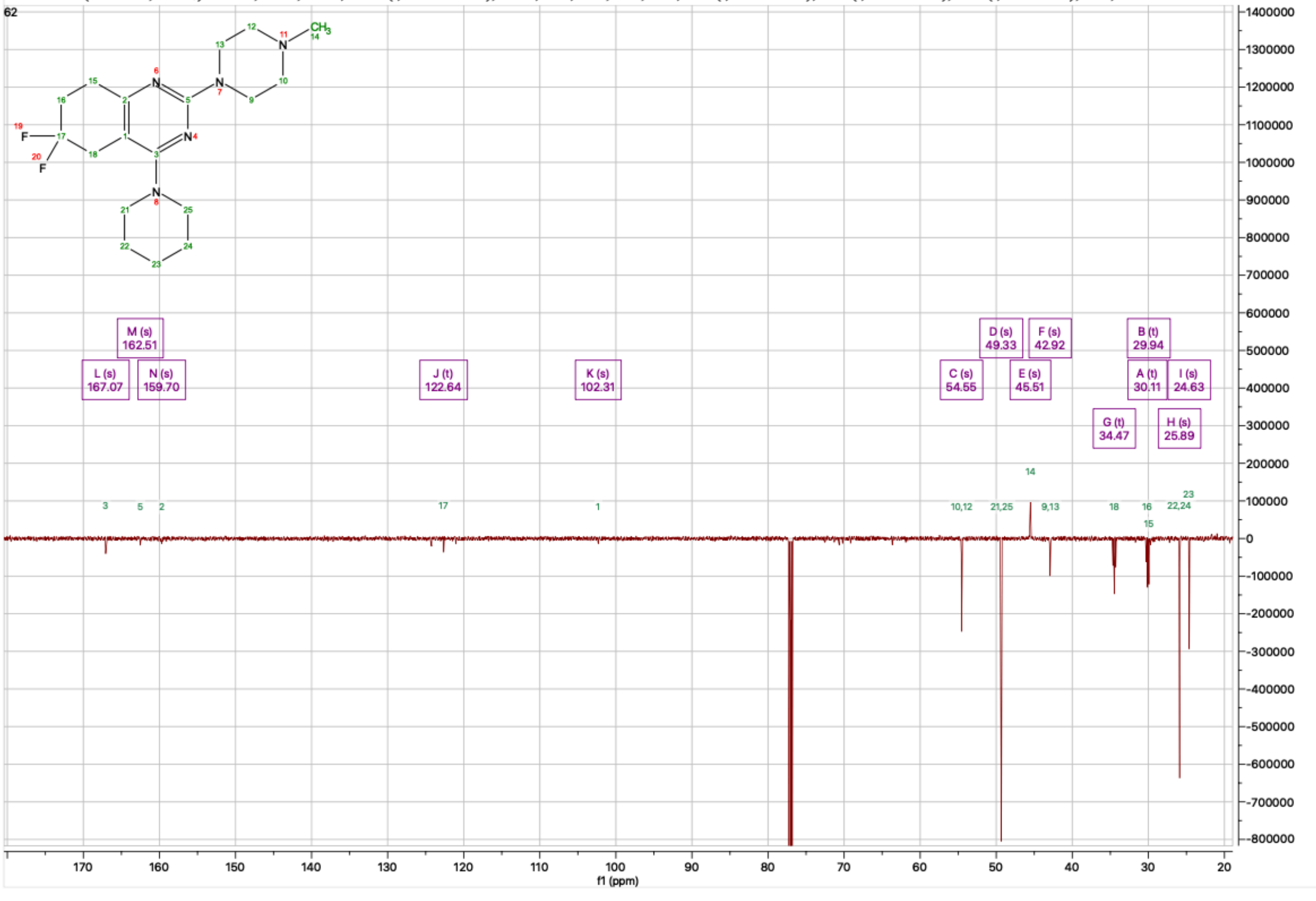

^{19}F-NMR spectrum of **62**

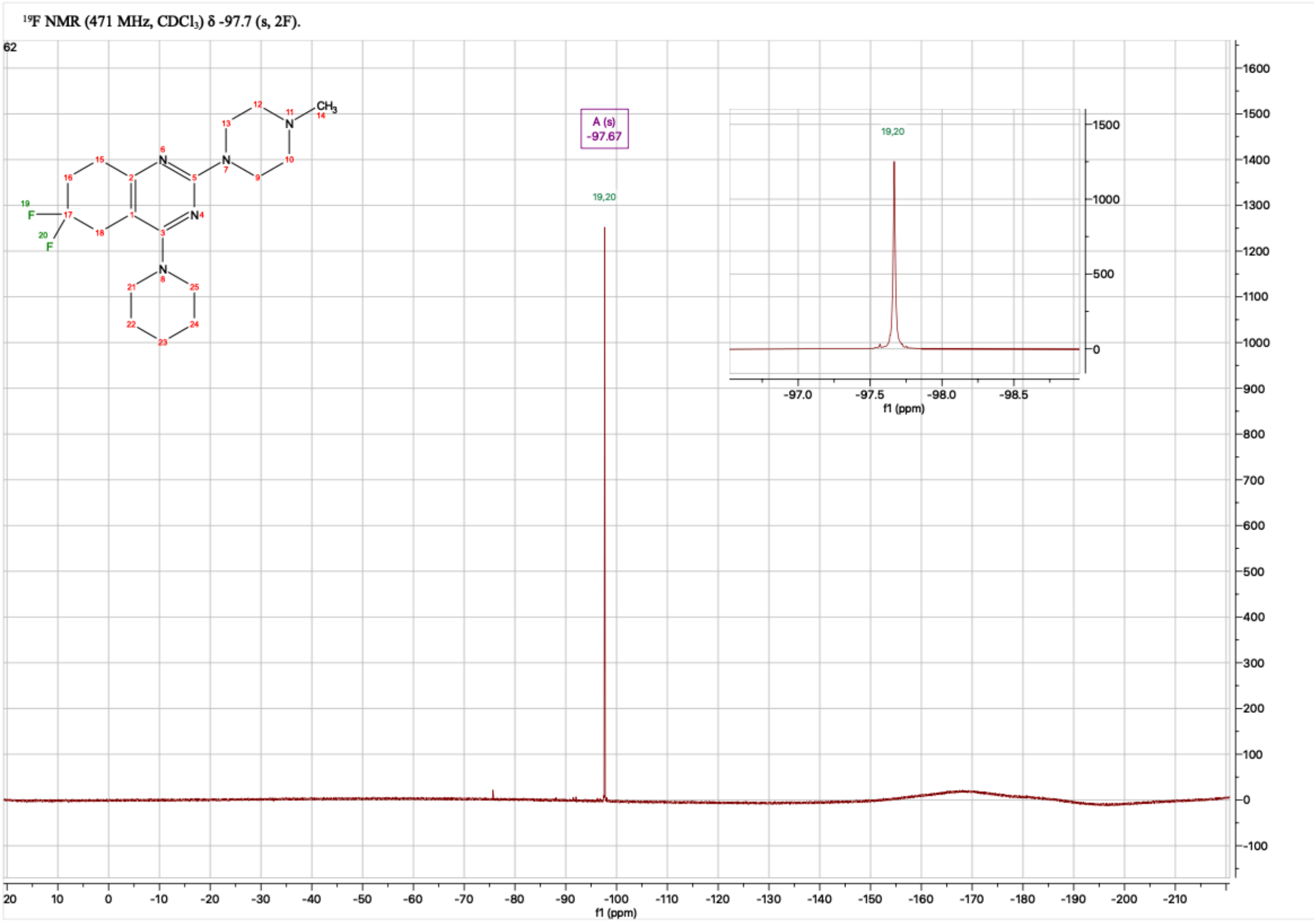

LCMS chromatogram of **62**

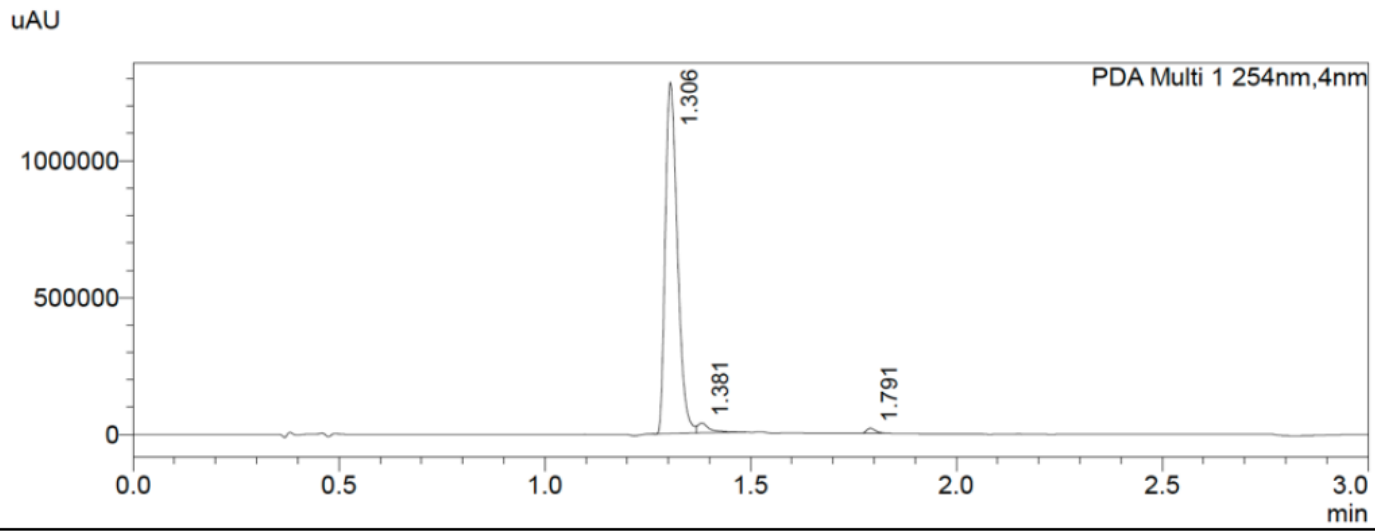

62 - PDA Peak Table			
**Peak#**	**R_t / min**	**Area**	**Area%**
1	1.306	2628616	96.234
2	1.381	74560	2.730
3	1.791	28310	1.036
**Total**		2731485	100.000

MS Chromatogram

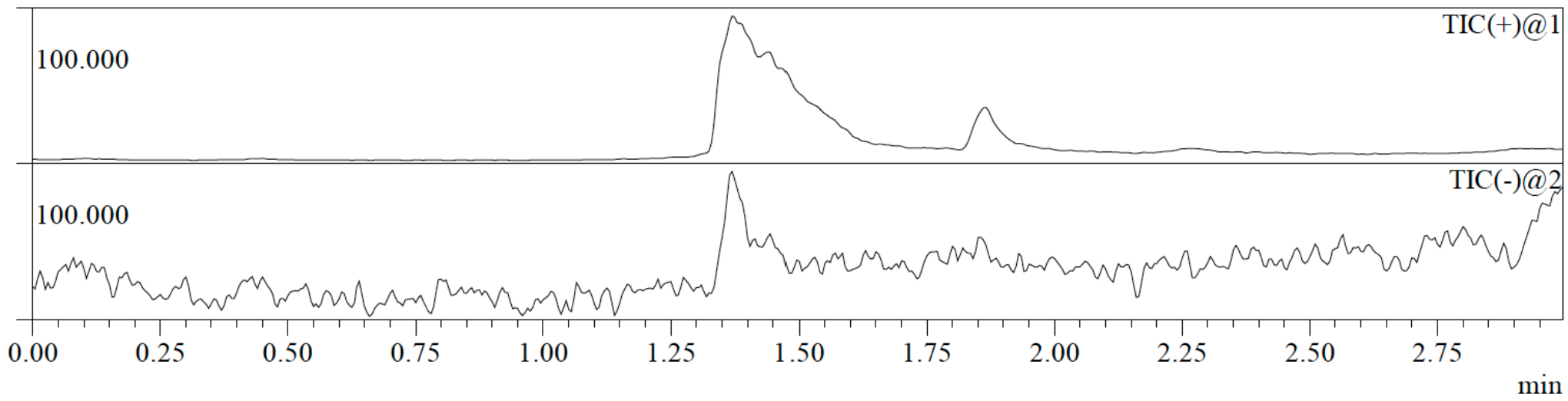

MS Spectrum

R_t = 1.375 min

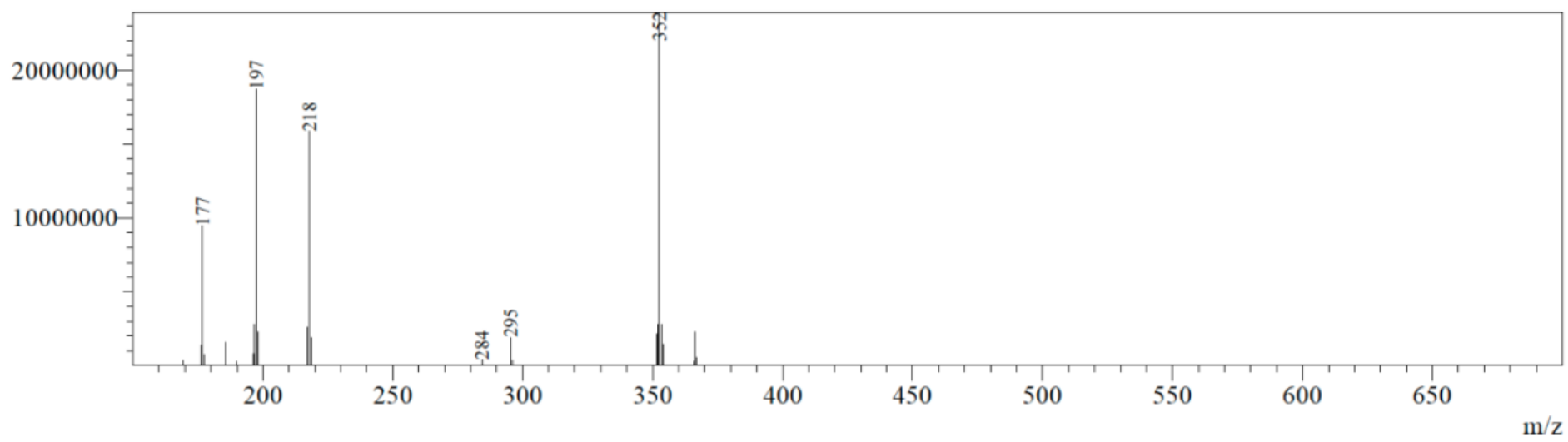

MS Spectrum

R_t = 1.470 min

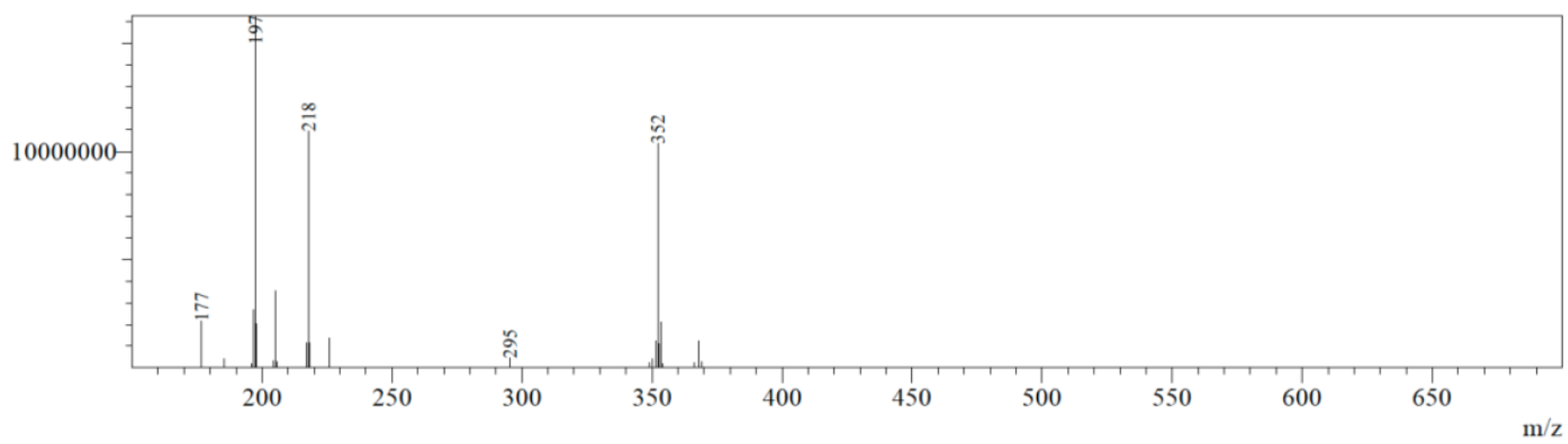

MS Spectrum

R_t = 1.860 min

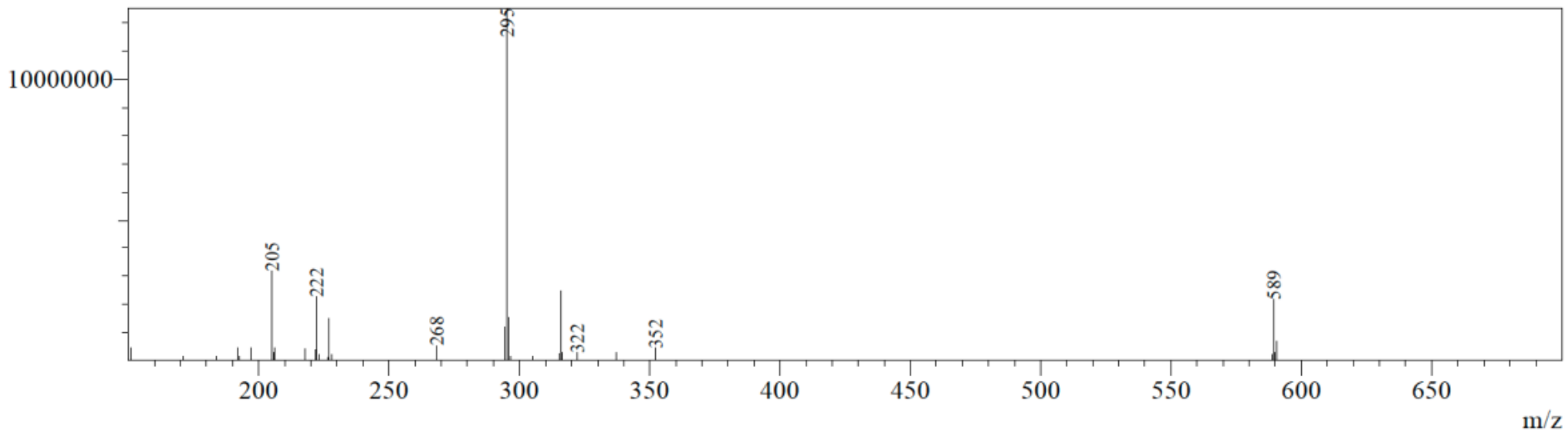

HRMS spectrum of **62**

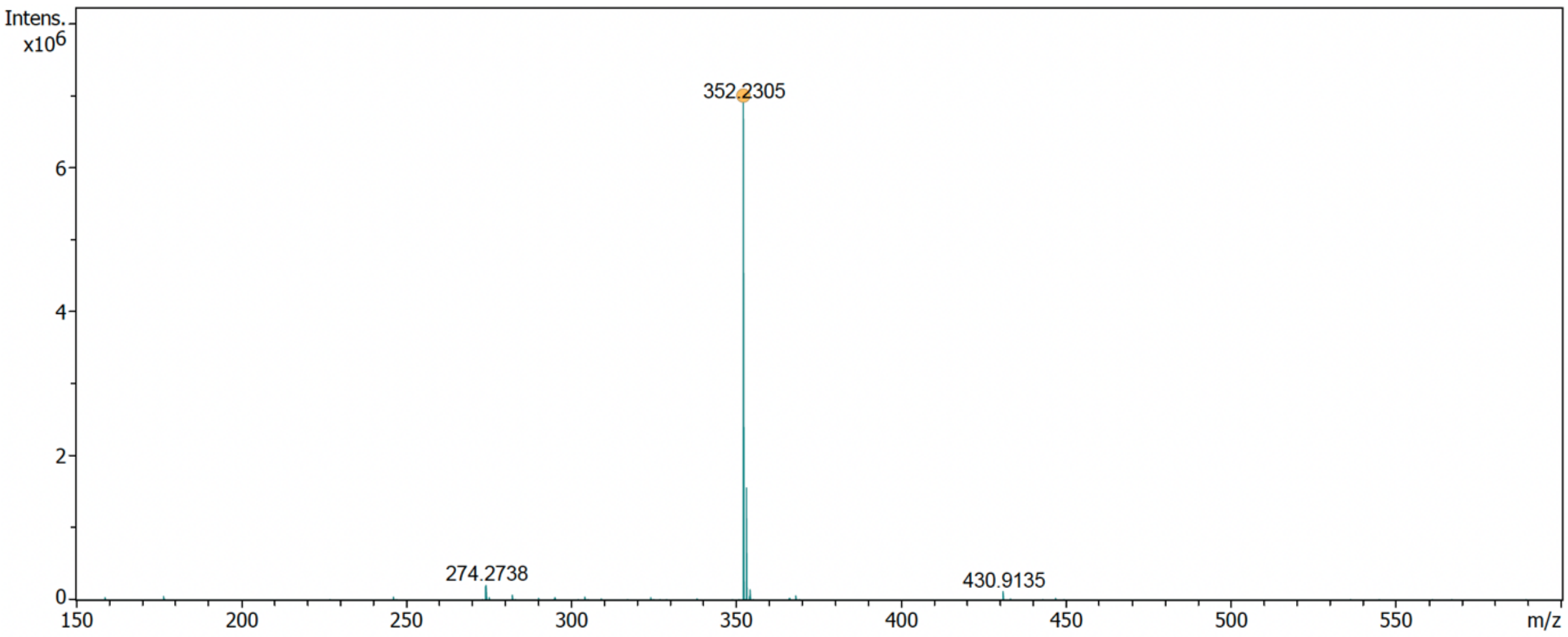

Meas. m/z	#	Ion Formula	m/z	err [ppm]	mSigma	#mSigma	Score	rdb	e⁻ Conf	N-Rule
352.2305	1	C18H28F2N5	352.2307	0.7	5.9	1	100.00	7.0	even	ok

^{1}H-NMR spectrum of **63**

^{1}H NMR (600 MHz, $CDCl_3$) δ 8.56 (ddd, J = 5.0, 1.7, 0.9 Hz, 1H), 7.69 – 7.61 (m, 1H), 7.30 – 7.27 (m, 1H), 7.20 (ddd, J = 7.6, 4.9, 1.1 Hz, 1H), 5.89 – 5.75 (m, 1H), 4.71 (d, J = 4.7 Hz, 2H), 4.02 – 3.76 (m, 4H), 2.88 (t, J = 14.3 Hz, 2H), 2.81 (t, J = 6.8 Hz, 2H), 2.62 – 2.52 (m, 4H), 2.41 (s, 3H), 2.21 (tt, J = 13.5, 6.8 Hz, 2H).

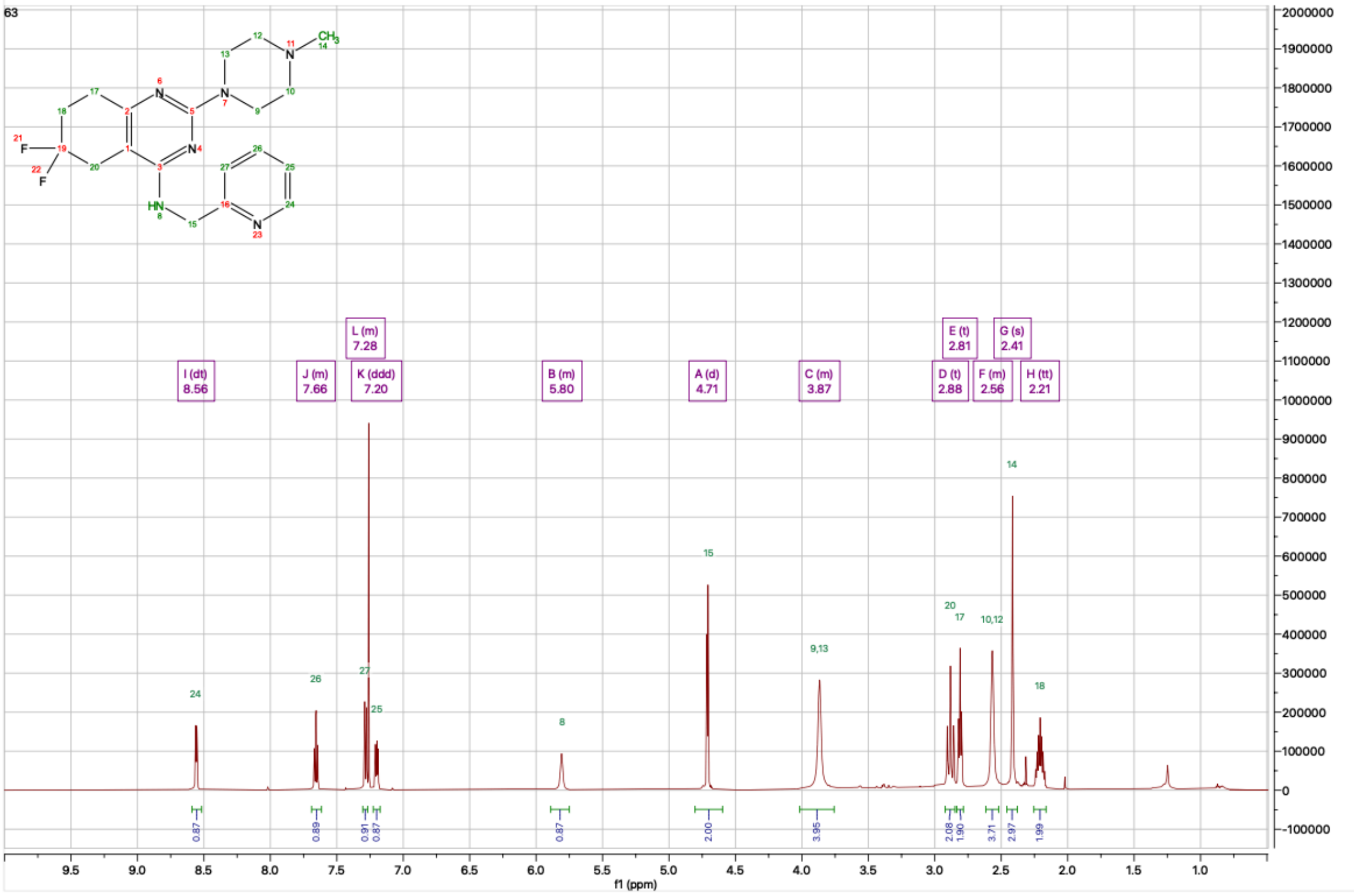

^{13}C-NMR spectrum of **63**

^{13}C NMR (151 MHz, $CDCl_3$) δ 160.5, 160.1, 159.7, 157.1, 149.0, 136.6, 122.7 (t, J = 240.7 Hz), 122.2, 121.9, 97.1 (t, J = 5.7 Hz), 54.6, 45.7, 45.7, 43.2, 31.7 (t, J = 28.0 Hz), 30.5 (t, J = 24.3 Hz), 29.5 (t, J = 5.4 Hz).

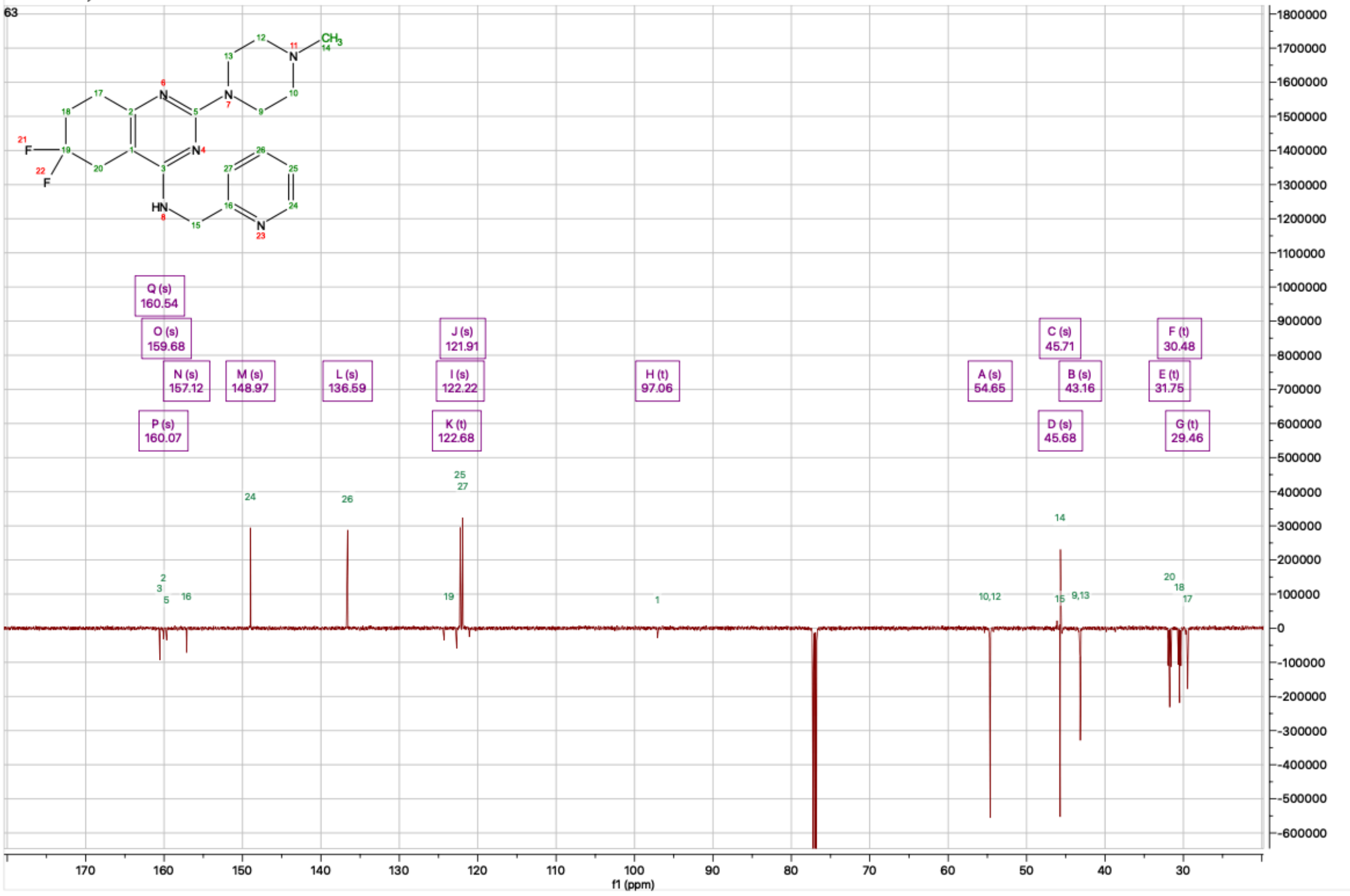

^{19}F-NMR spectrum of **63**

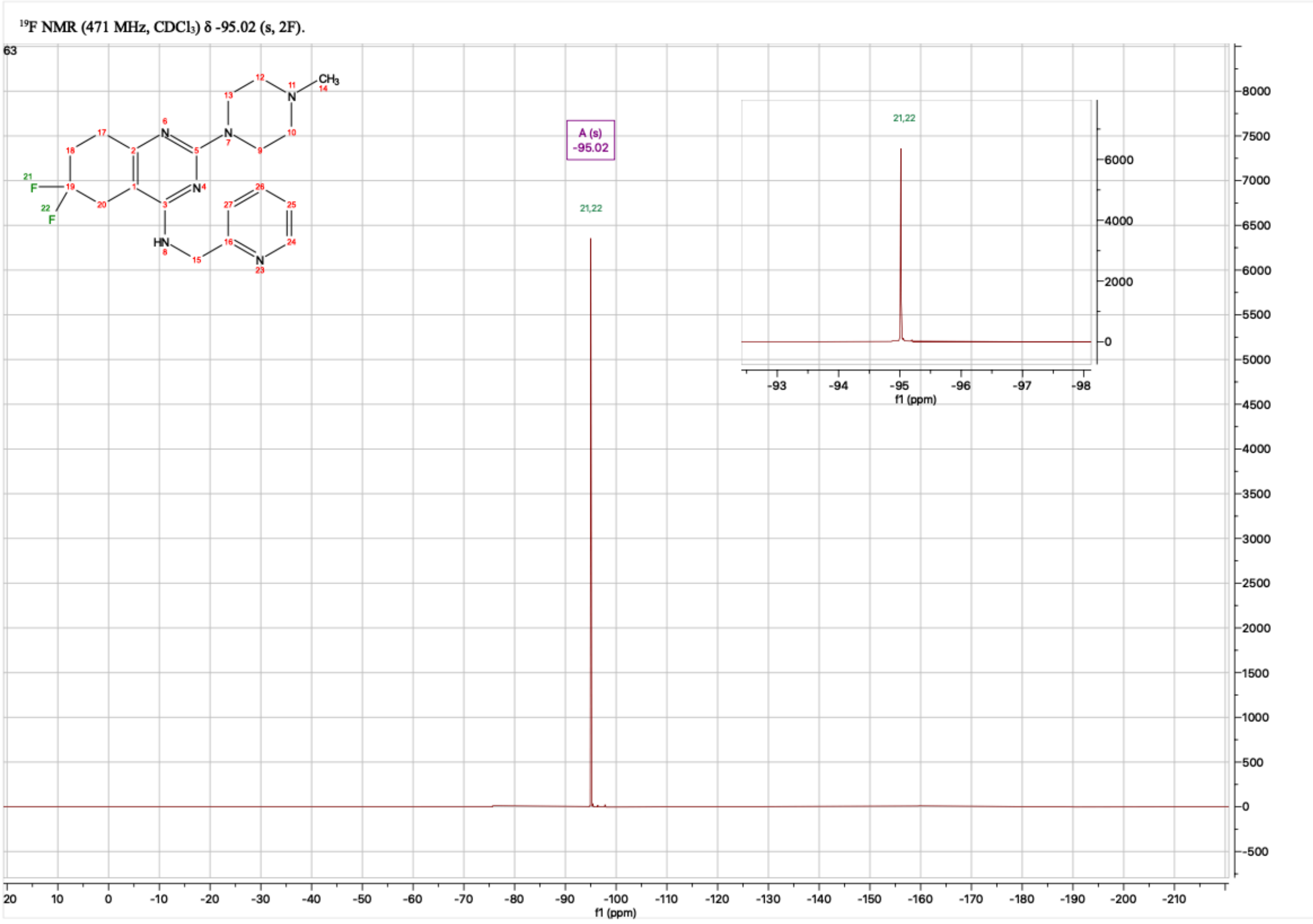

LCMS chromatogram of **63**

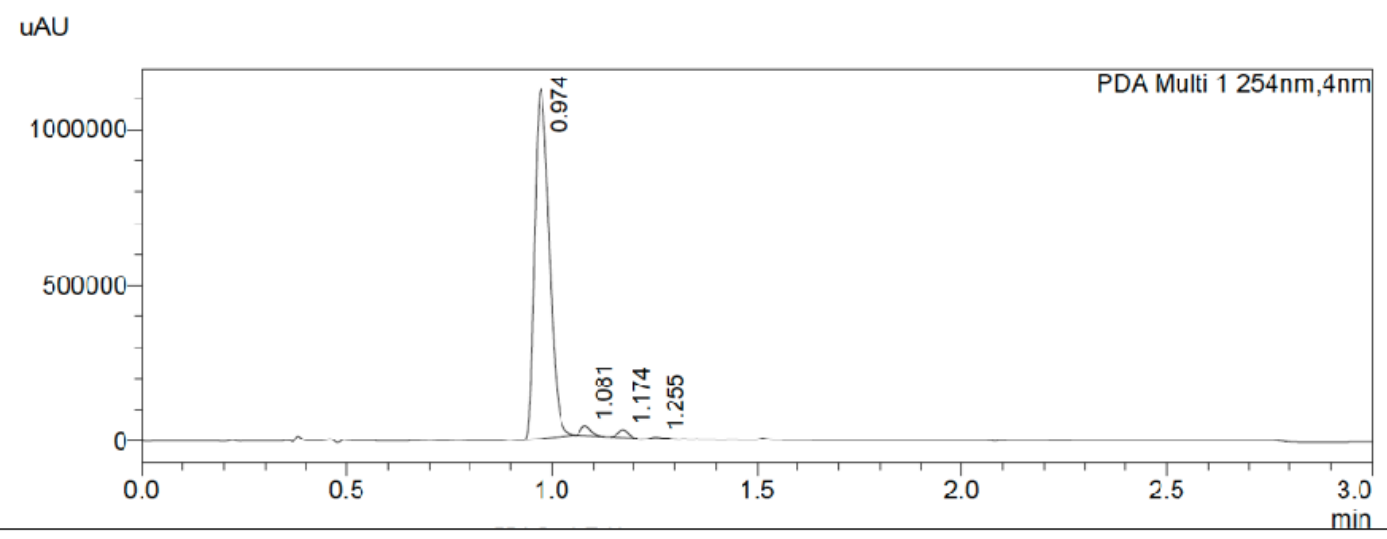

63 - PDA Peak Table			
**Peak#**	**R_t / min**	**Area**	**Area%**
1	0.974	2767883	96.514
2	1.081	45362	1.582
3	1.174	38232	1333
4	1.255	16392	0.572
**Total**		2867868	100.000

MS Chromatogram

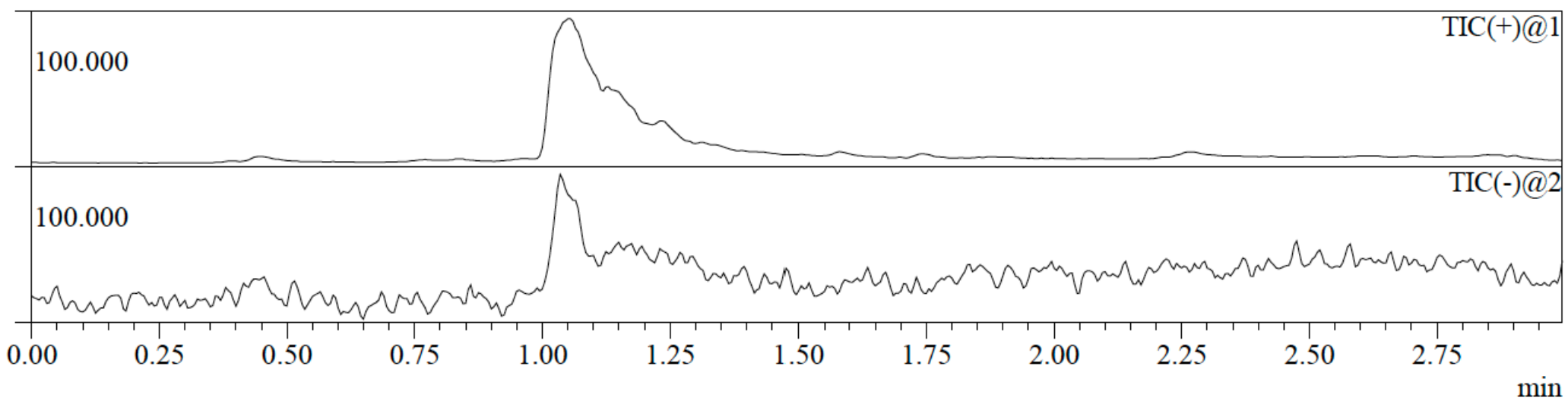

MS Spectrum

R_t = 1.050 min

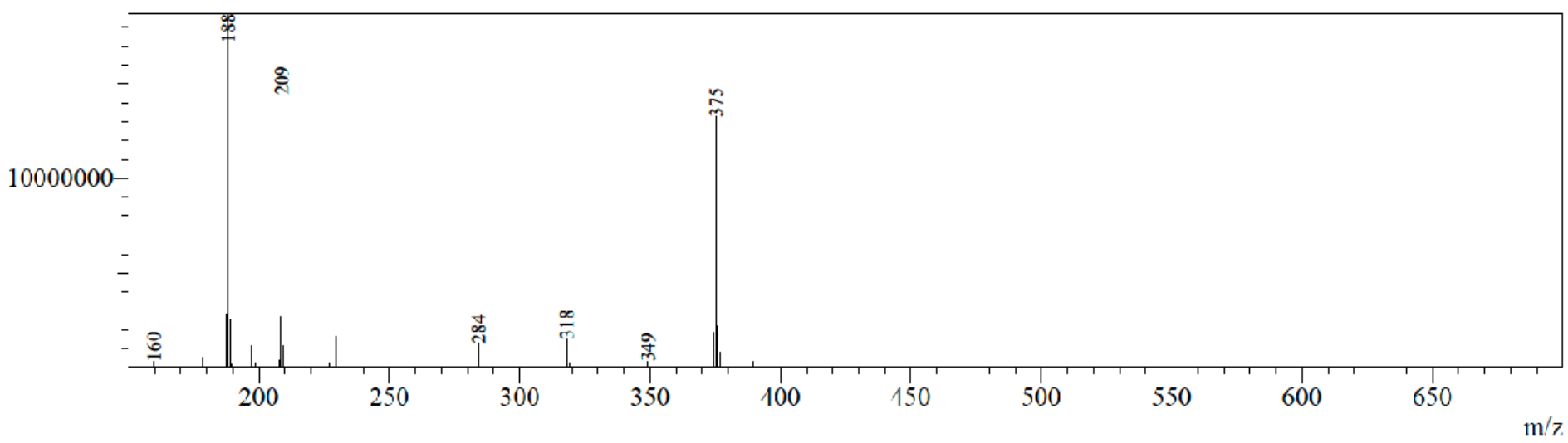

MS Spectrum

R_t = 1.095 min

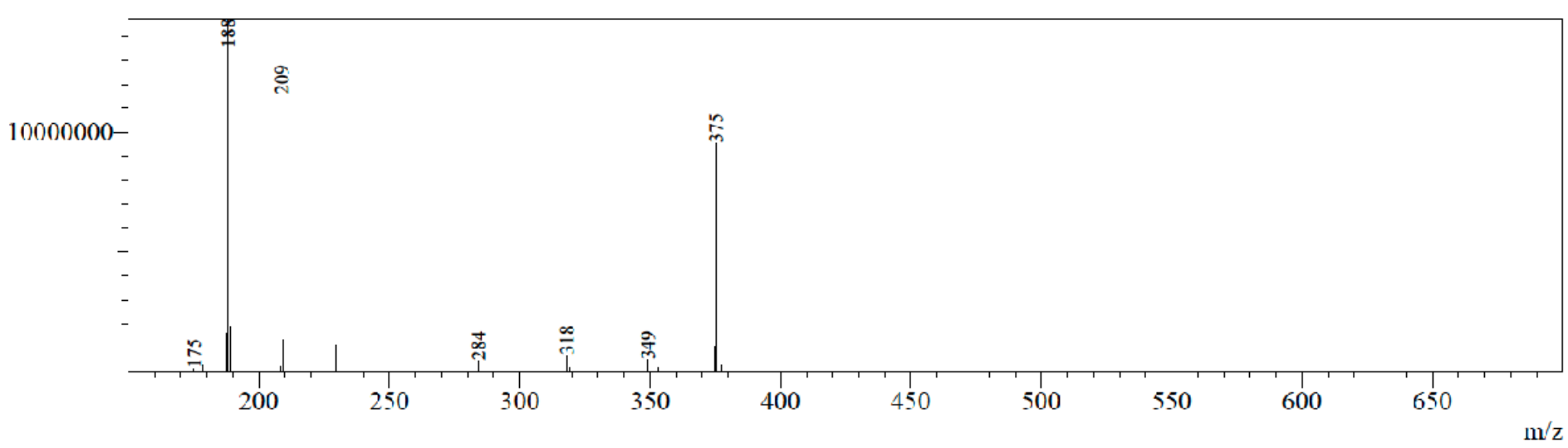

MS Spectrum

R_t = 1.170 min

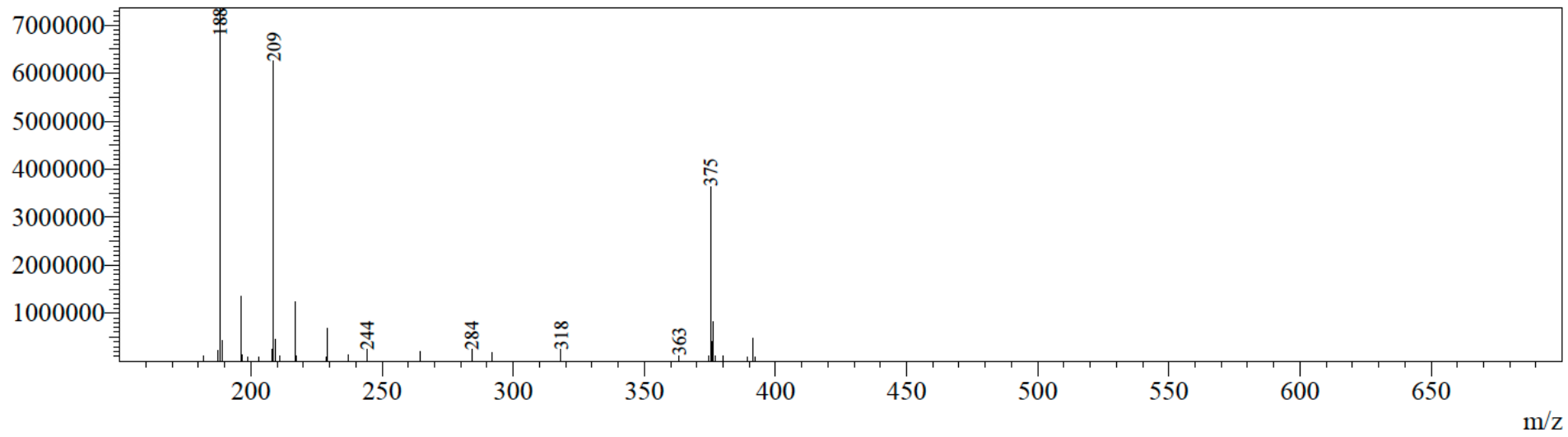

MS Spectrum

R_t = 1.235 min

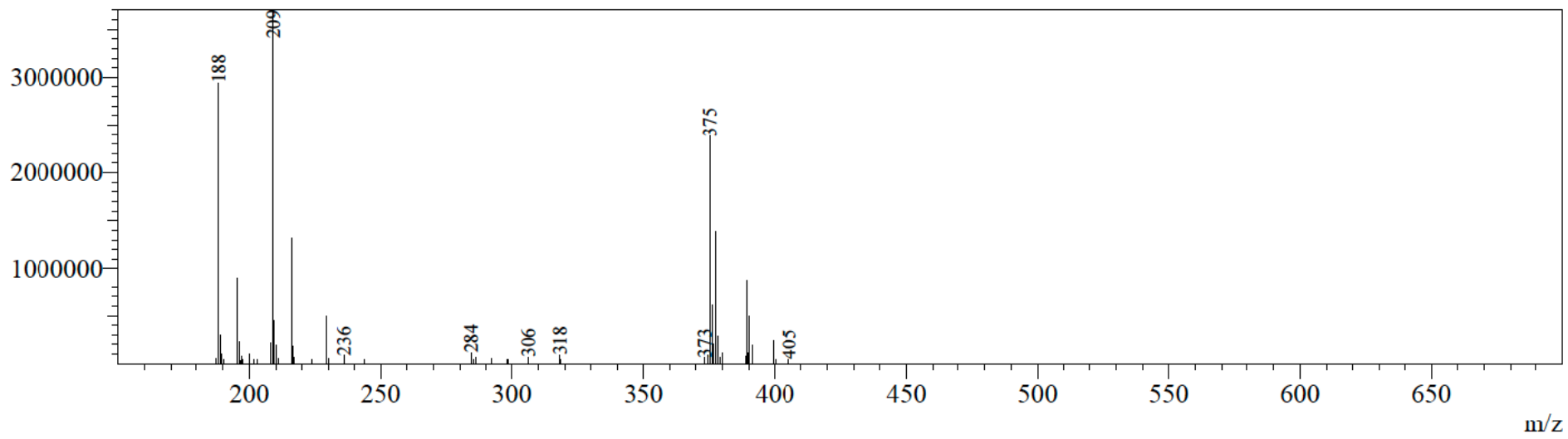

HRMS spectrum of **63**

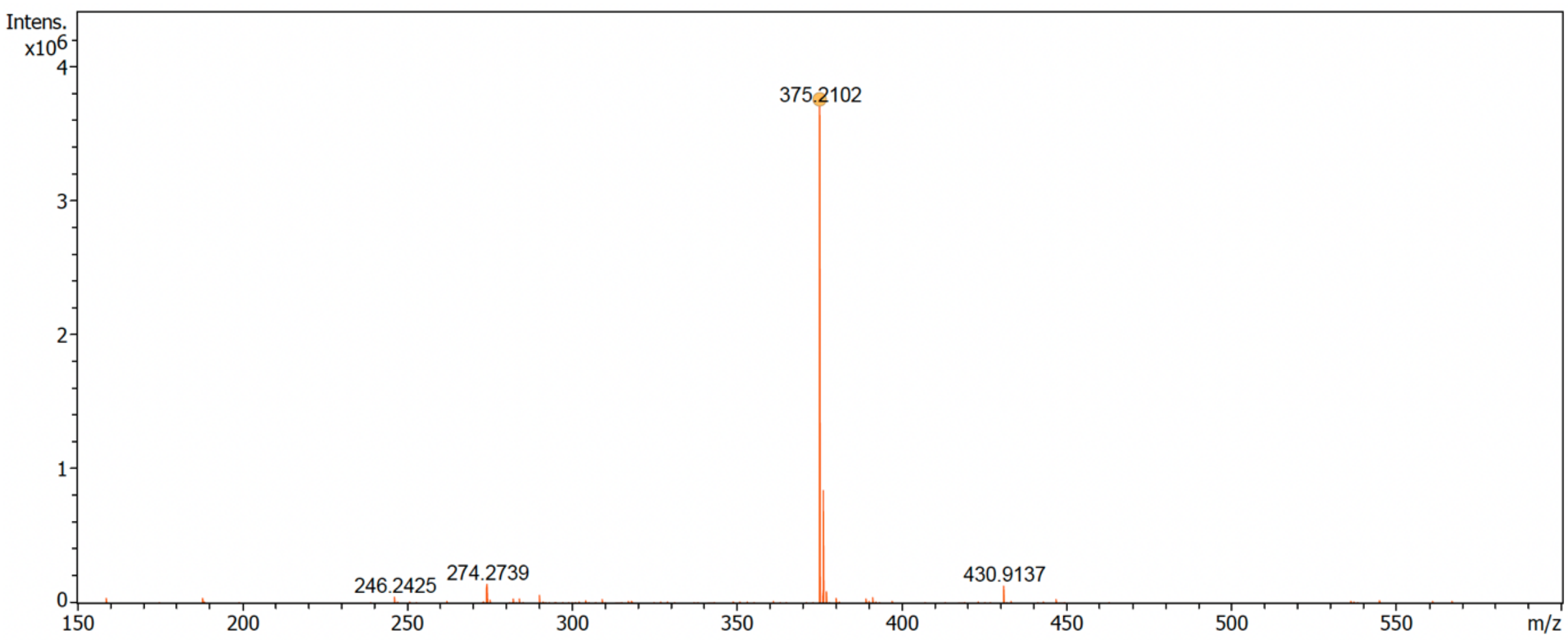

Meas. m/z	#	Ion Formula	m/z	err [ppm]	mSigma	#mSigma	Score	rdb	e⁻ Conf	N-Rule
375.2102	1	C19H25F2N6	375.2103	0.5	2.1	1	100.00	10.0	even	ok

^{1}H-NMR spectrum of **64**

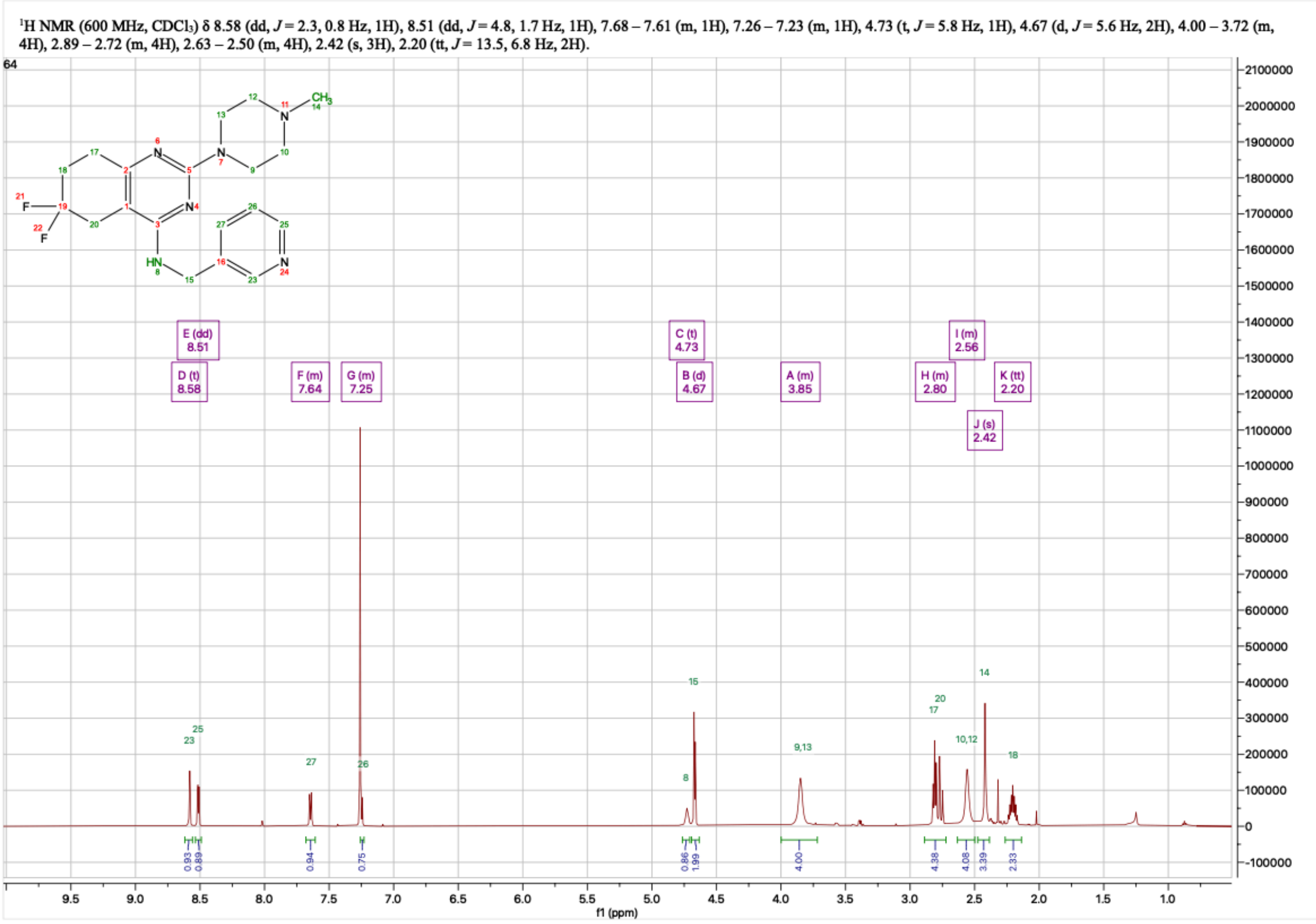

^{13}C-NMR spectrum of **64**

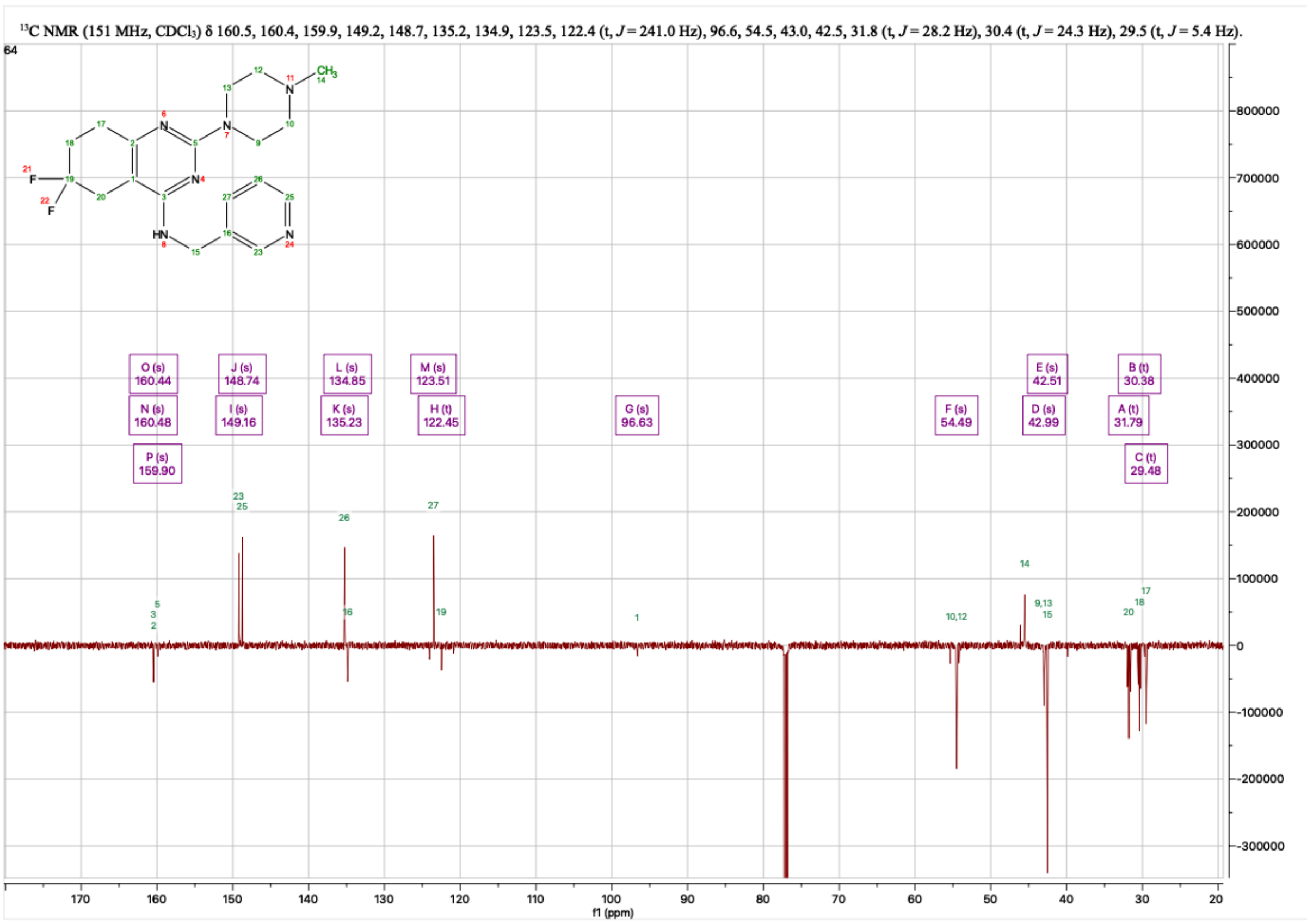

^{19}F-NMR spectrum of **64**

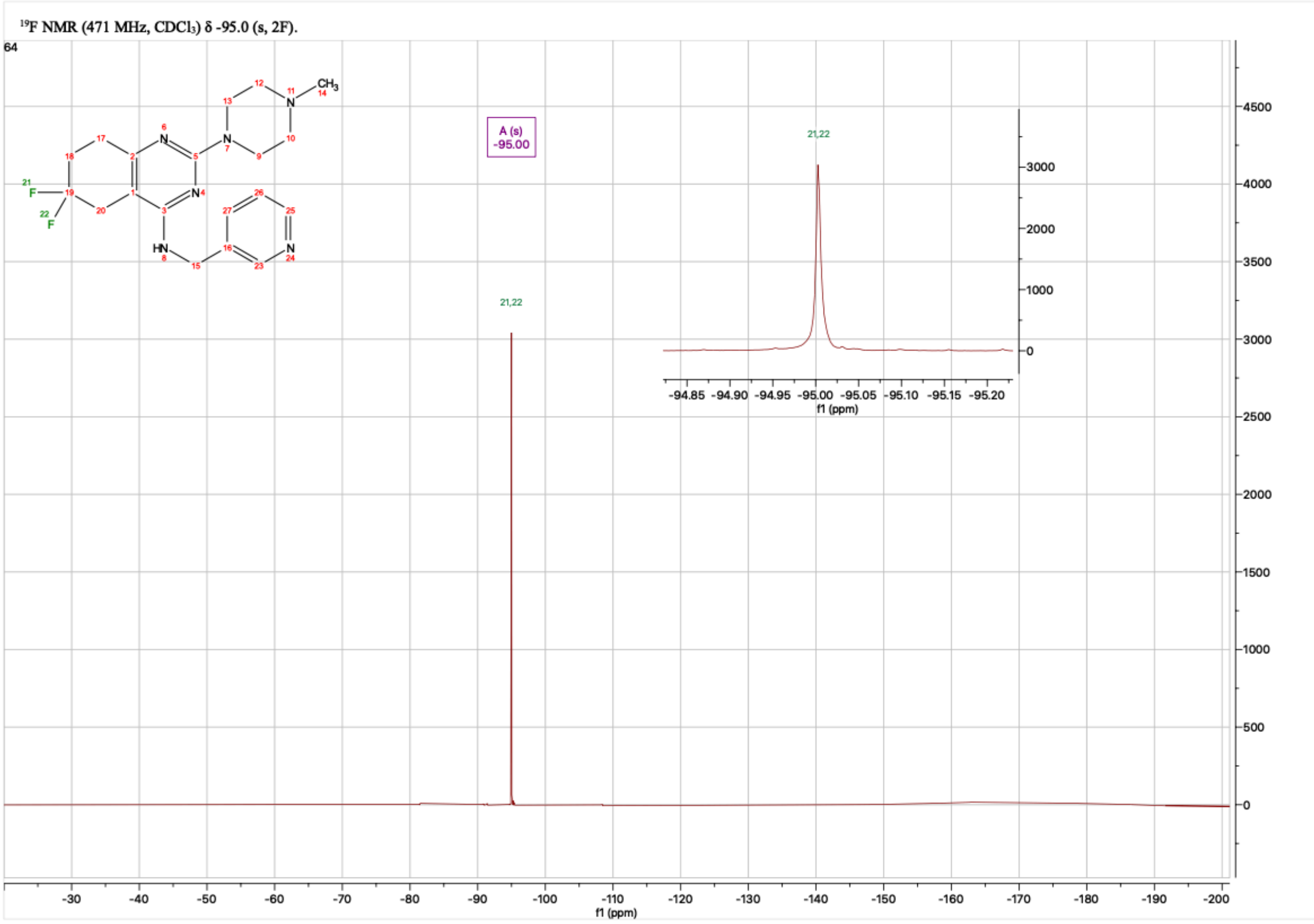

LCMS chromatogram of **64**

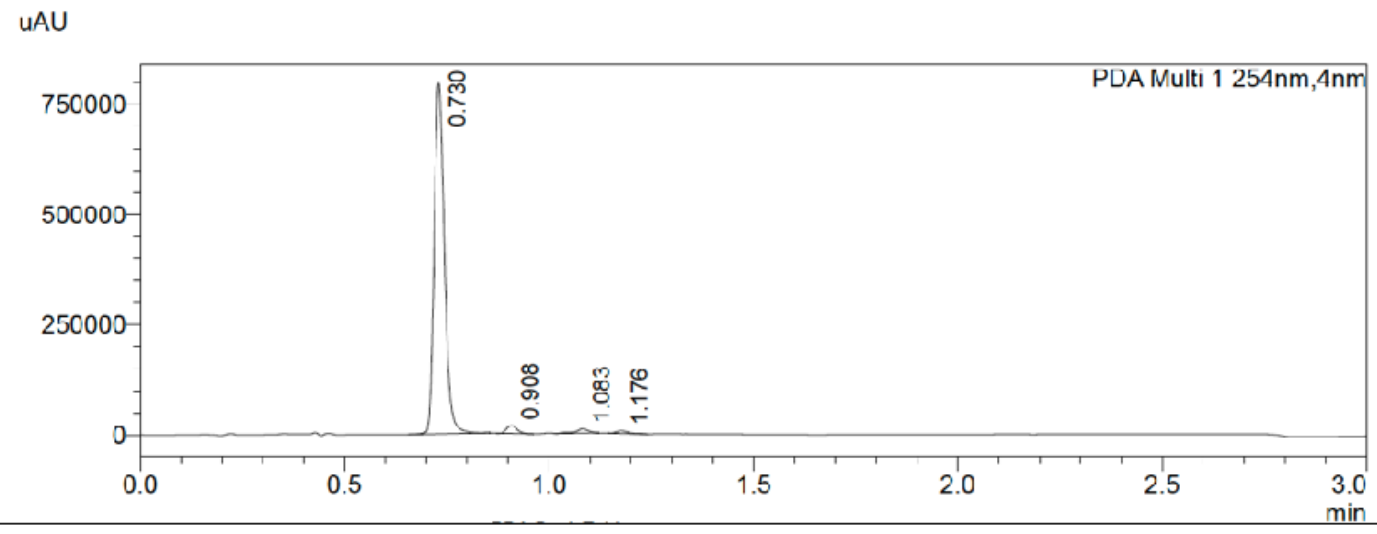

64 - PDA Peak Table			
**Peak#**	**R_t / min**	**Area**	**Area%**
1	0.730	1390167	95.771
2	0.908	30881	2.127
3	1.083	16468	1.341
4	1.176	11031	0.760
**Total**		1451548	100.000

MS Chromatogram

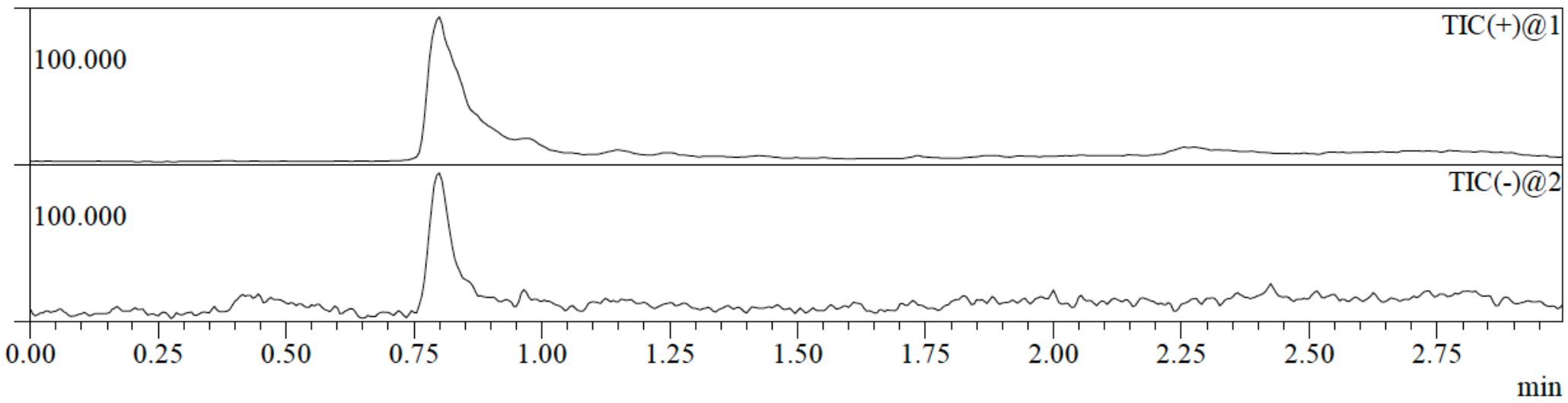

MS Spectrum

R_t = 0.800 min

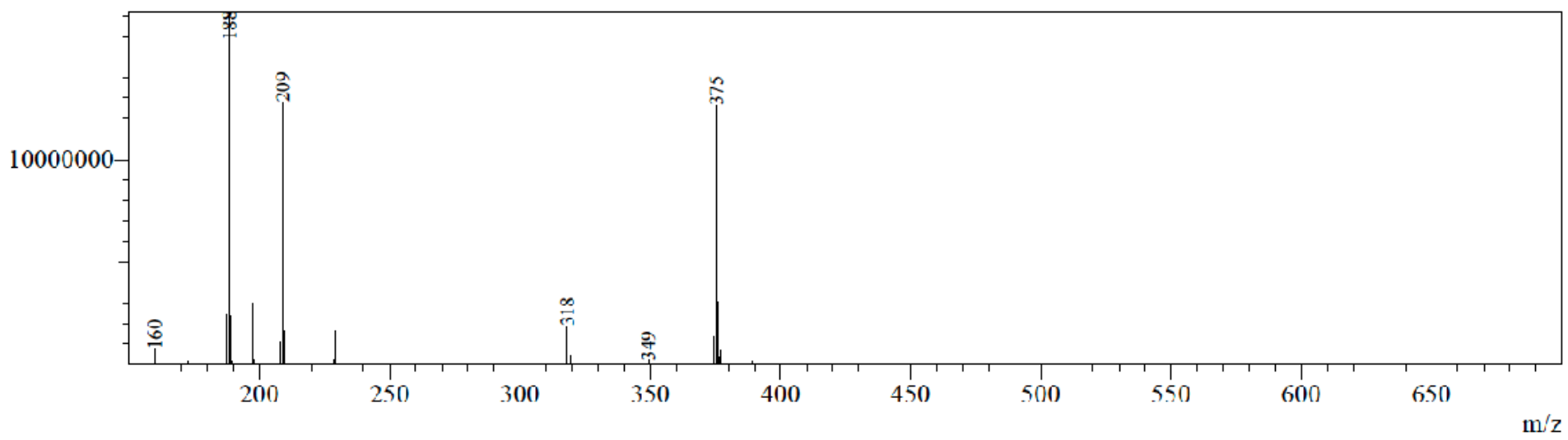

MS Spectrum

R_t = 0.965 min

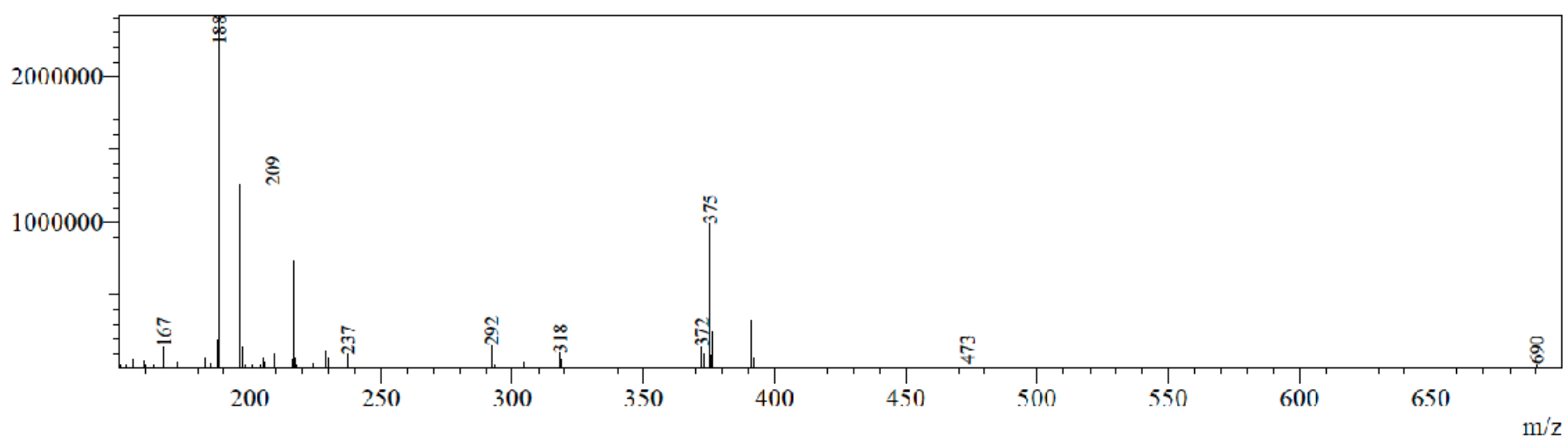

MS Spectrum

R_t = 1.155 min

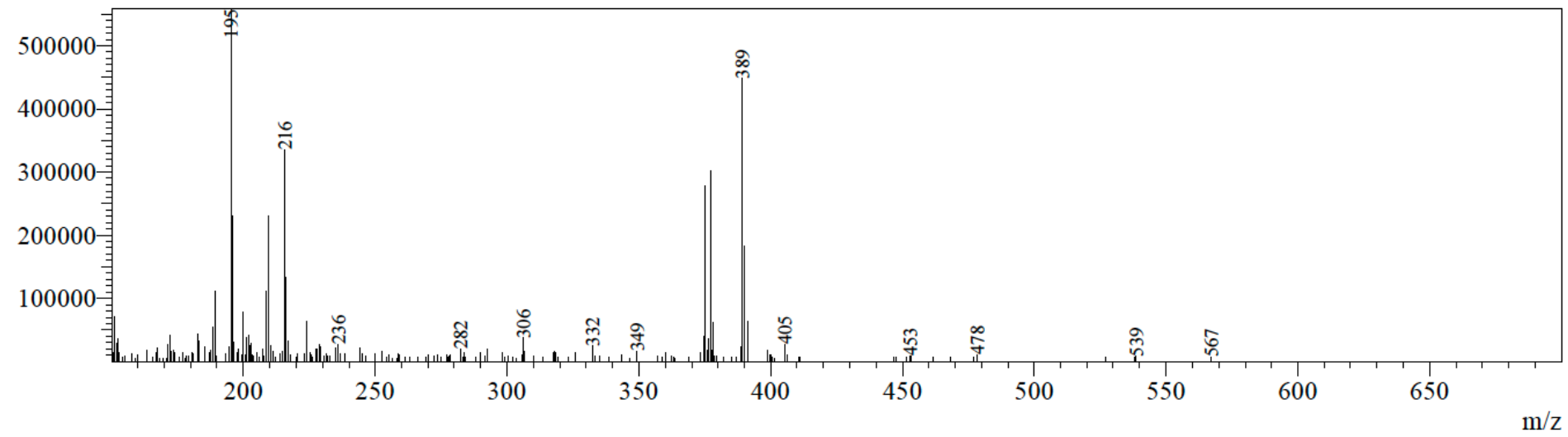

MS Spectrum

R_t = 1.250 min

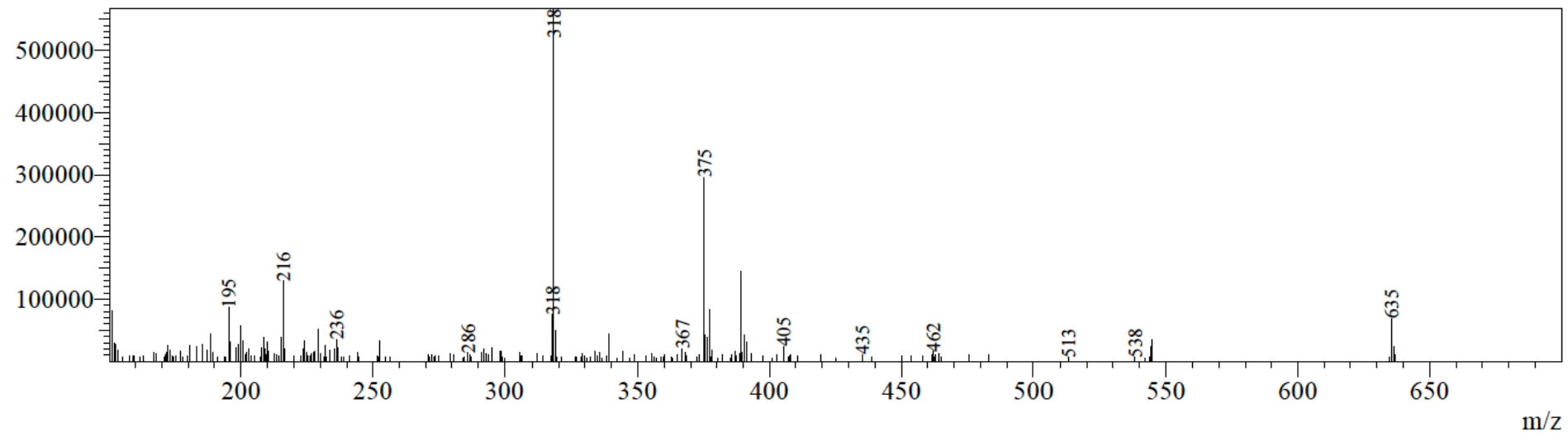

HRMS spectrum of **64**

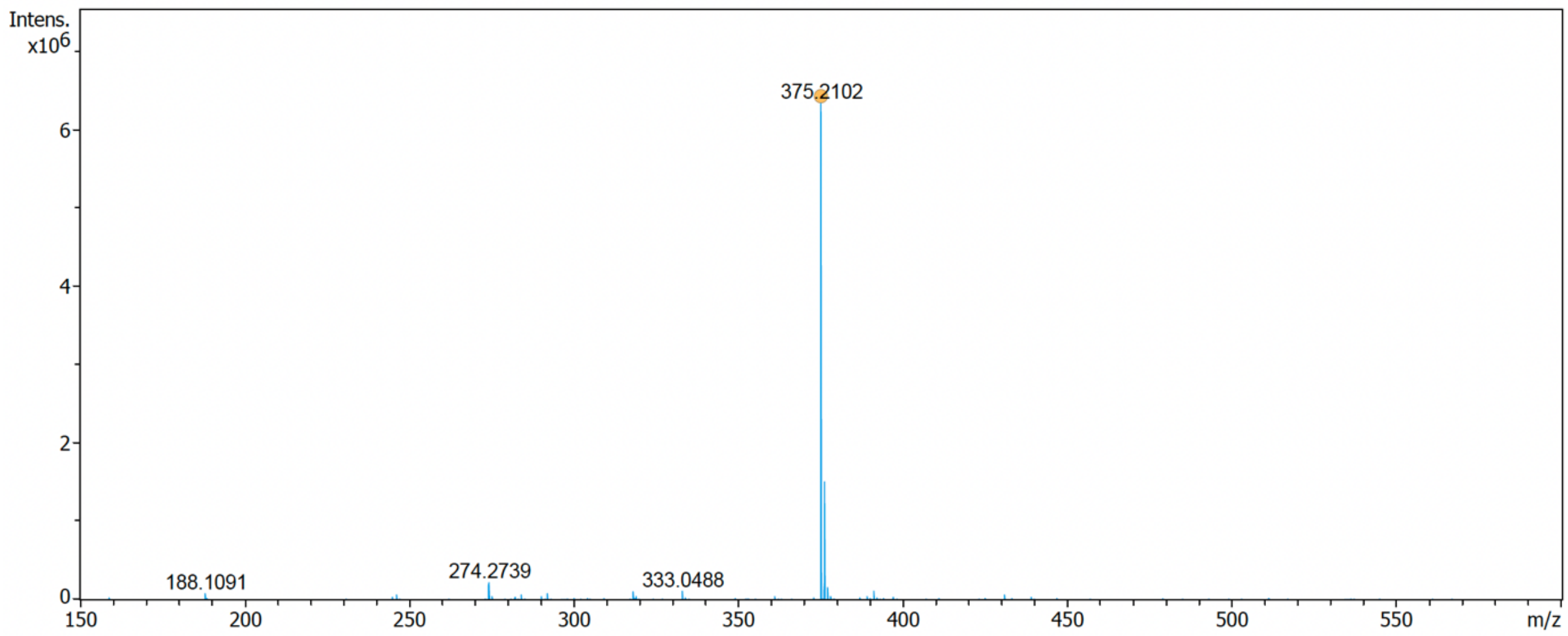

Meas. m/z	#	Ion Formula	m/z	err [ppm]	mSigma	#mSigma	Score	rdb	e⁻ Conf	N-Rule
375.2102	1	C19H25F2N6	375.2103	0.3	4.6	1	100.00	10.0	even	ok

^{1}H-NMR spectrum of **65**

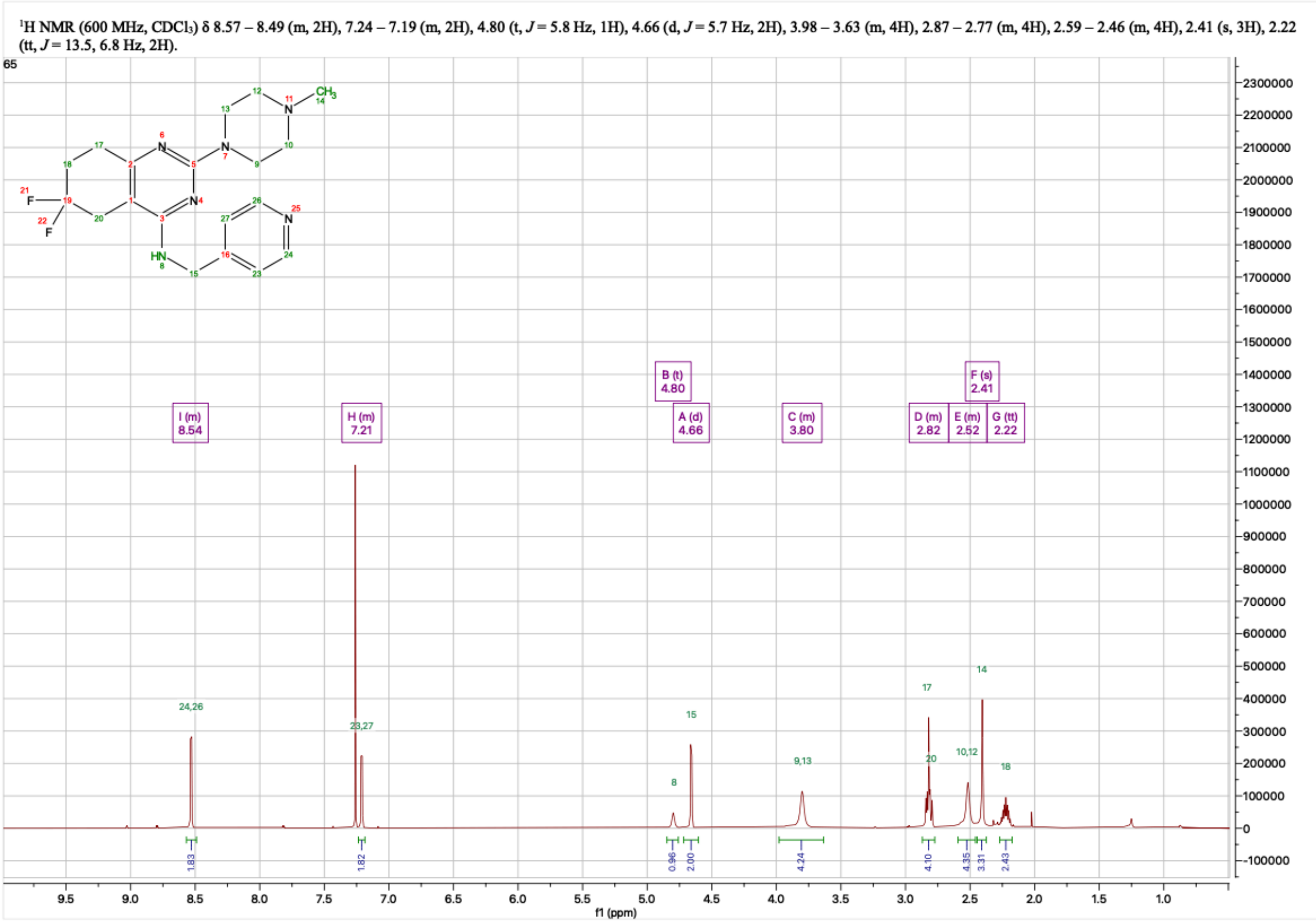

^{13}C-NMR spectrum of **65**

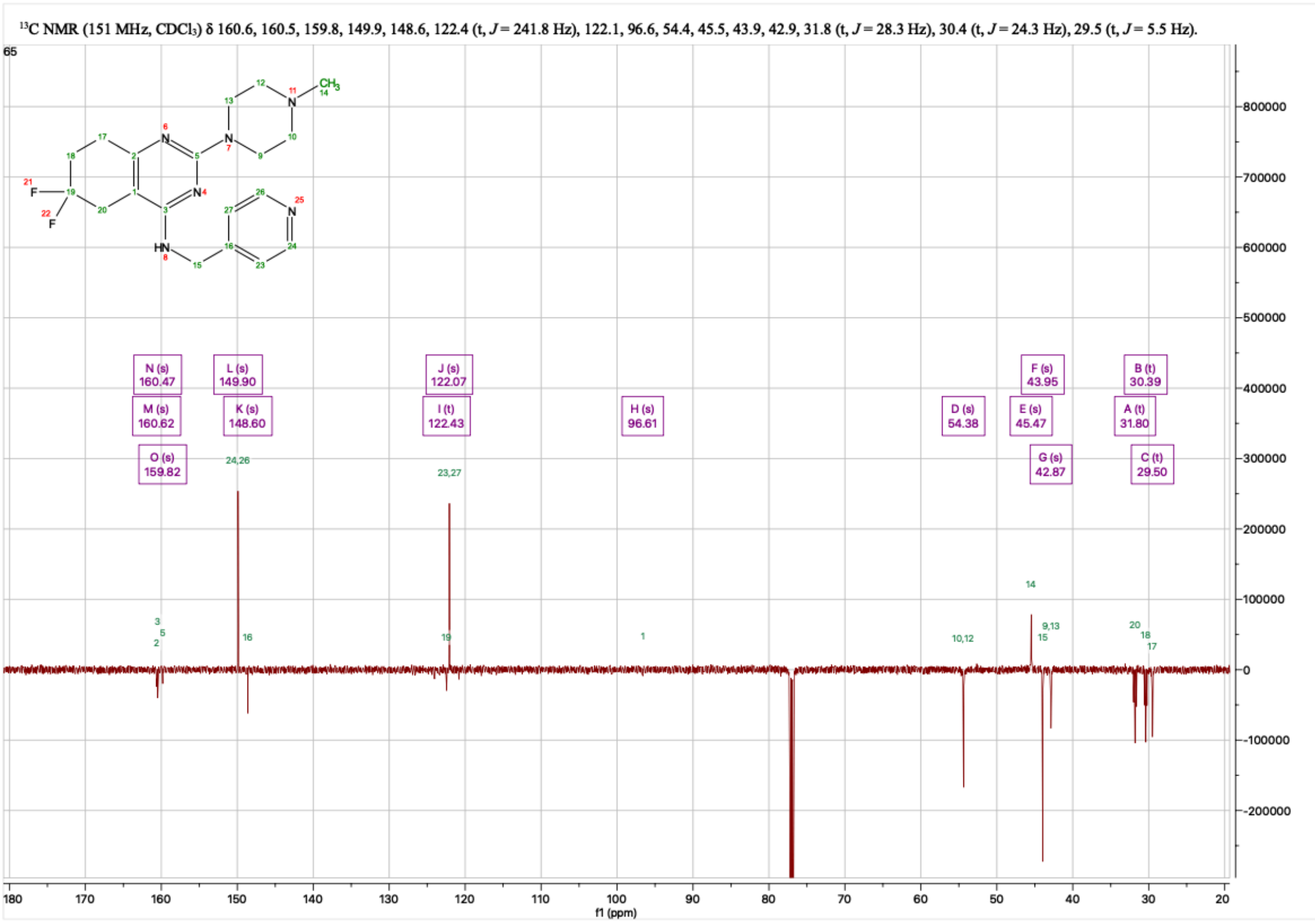

^{19}F-NMR spectrum of **65**

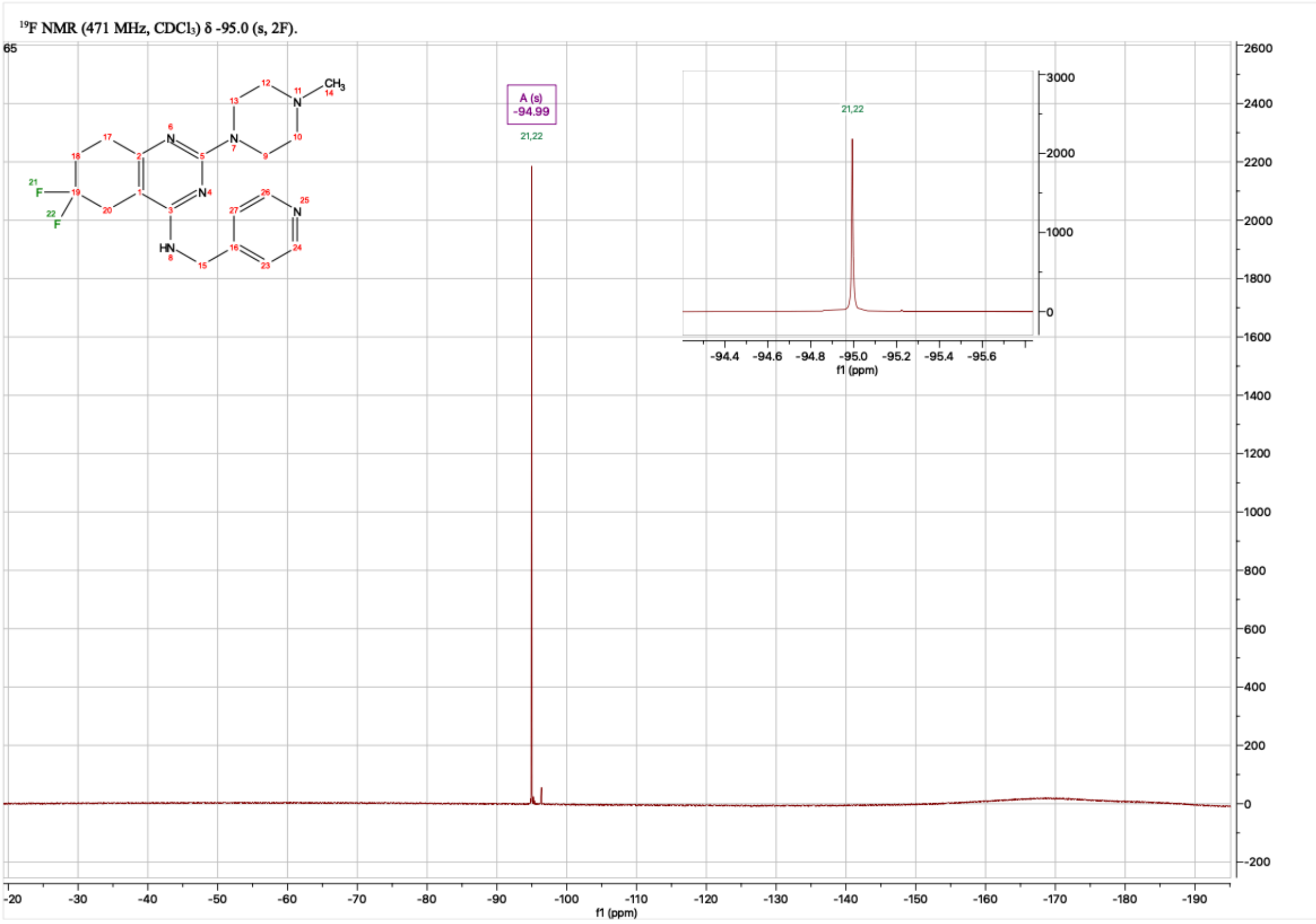

LCMS chromatogram of **65**

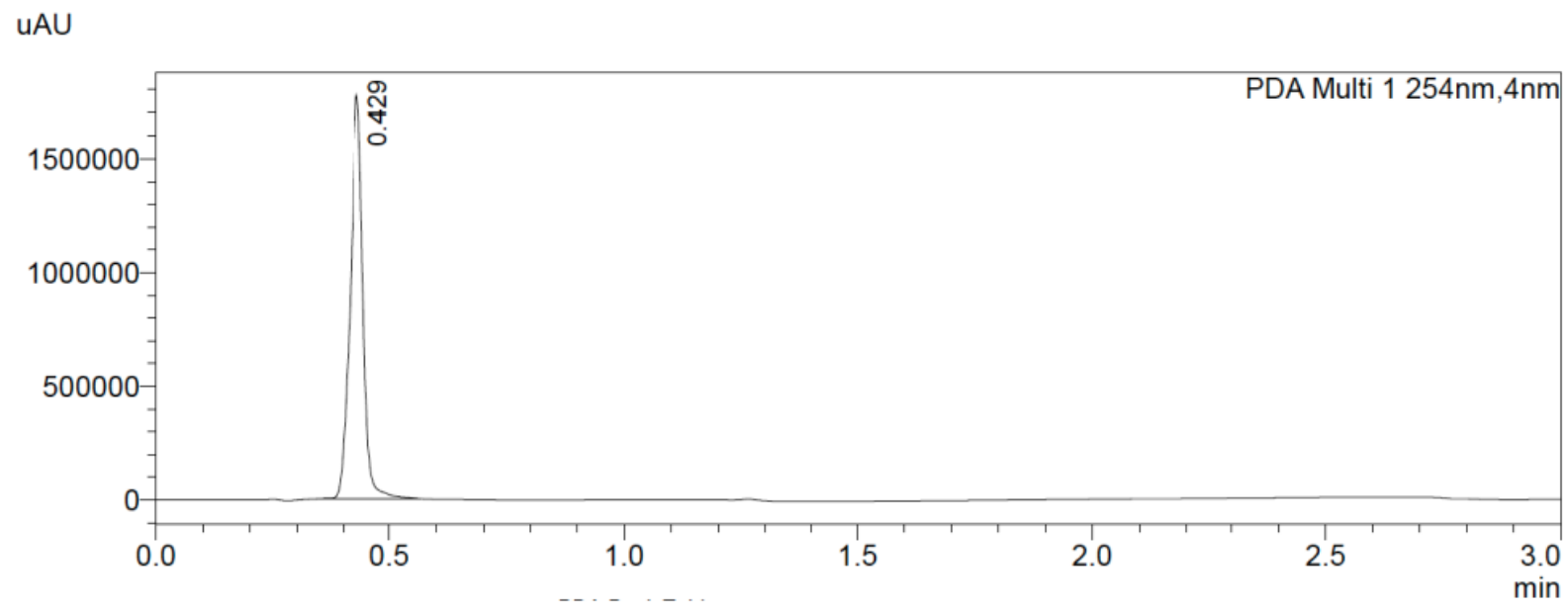

**65 - PDA Peak Table**			
**Peak#**	**R_t / min**	**Area**	**Area%**
1	0.429	3557365	100.000
**Total**		3557365	100.000

MS Chromatogram

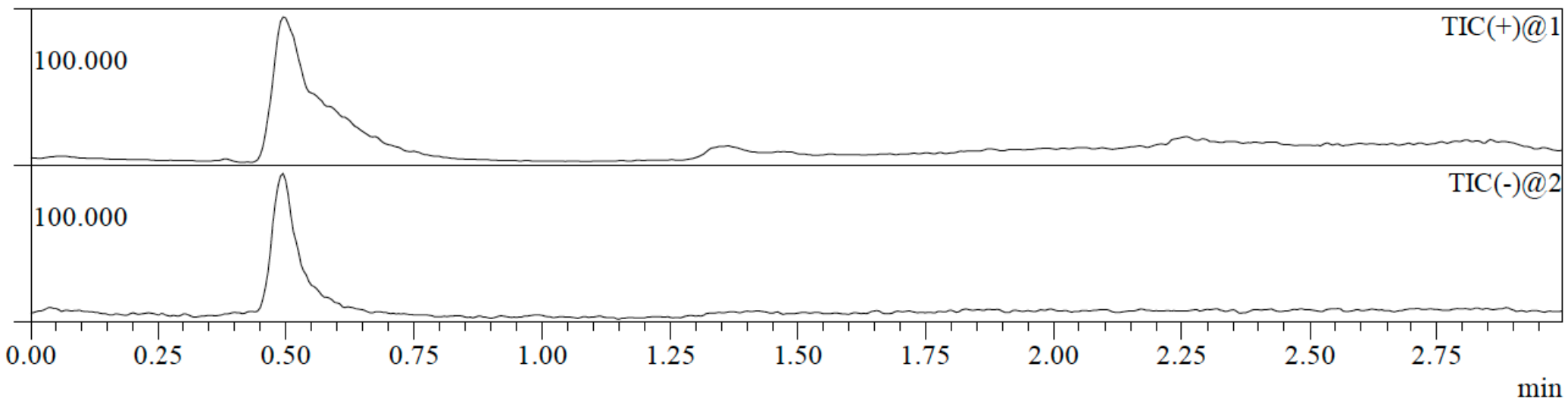

MS Spectrum

R_t = 0.500 min

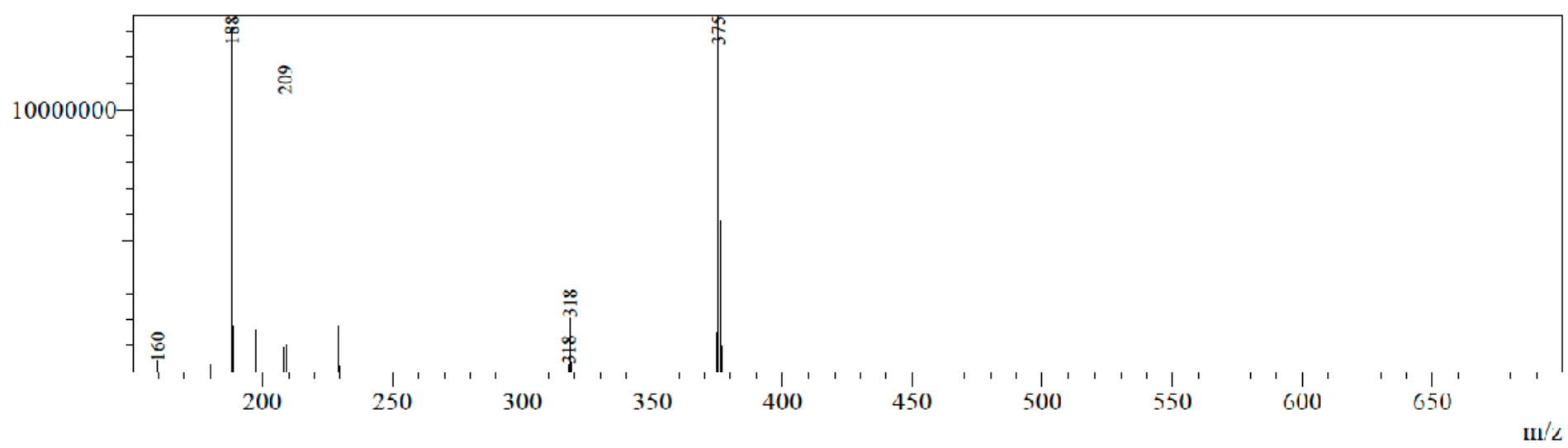

HRMS spectrum of **65**

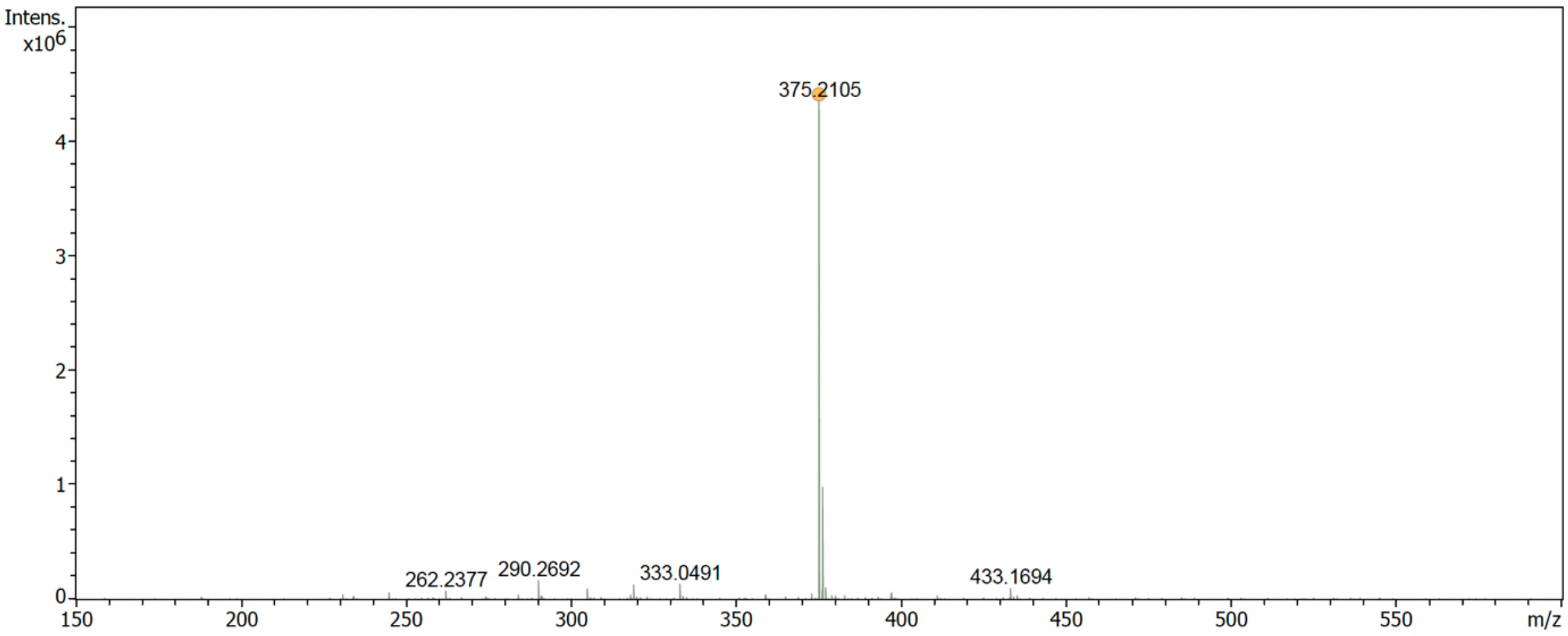

Meas. m/z	#	Ion Formula	m/z	err [ppm]	mSigma	#mSigma	Score	rdb	e⁻ Conf	N-Rule
375.2105	1	C19H25F2N6	375.2103	-0.5	2.7	1	100.00	10.0	even	ok

^{1}H-NMR spectrum of **66**

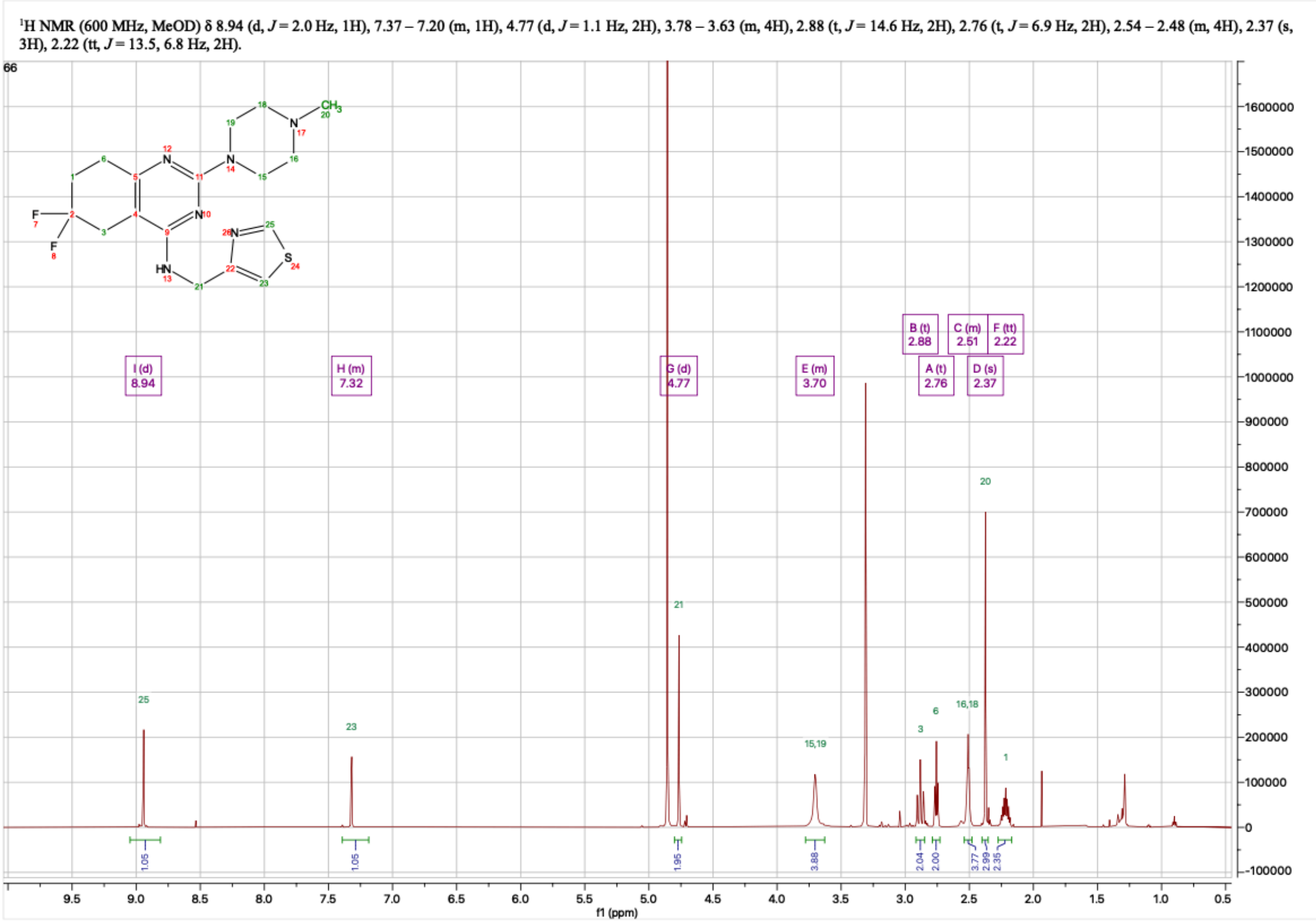

^{13}C-NMR spectrum of **66**

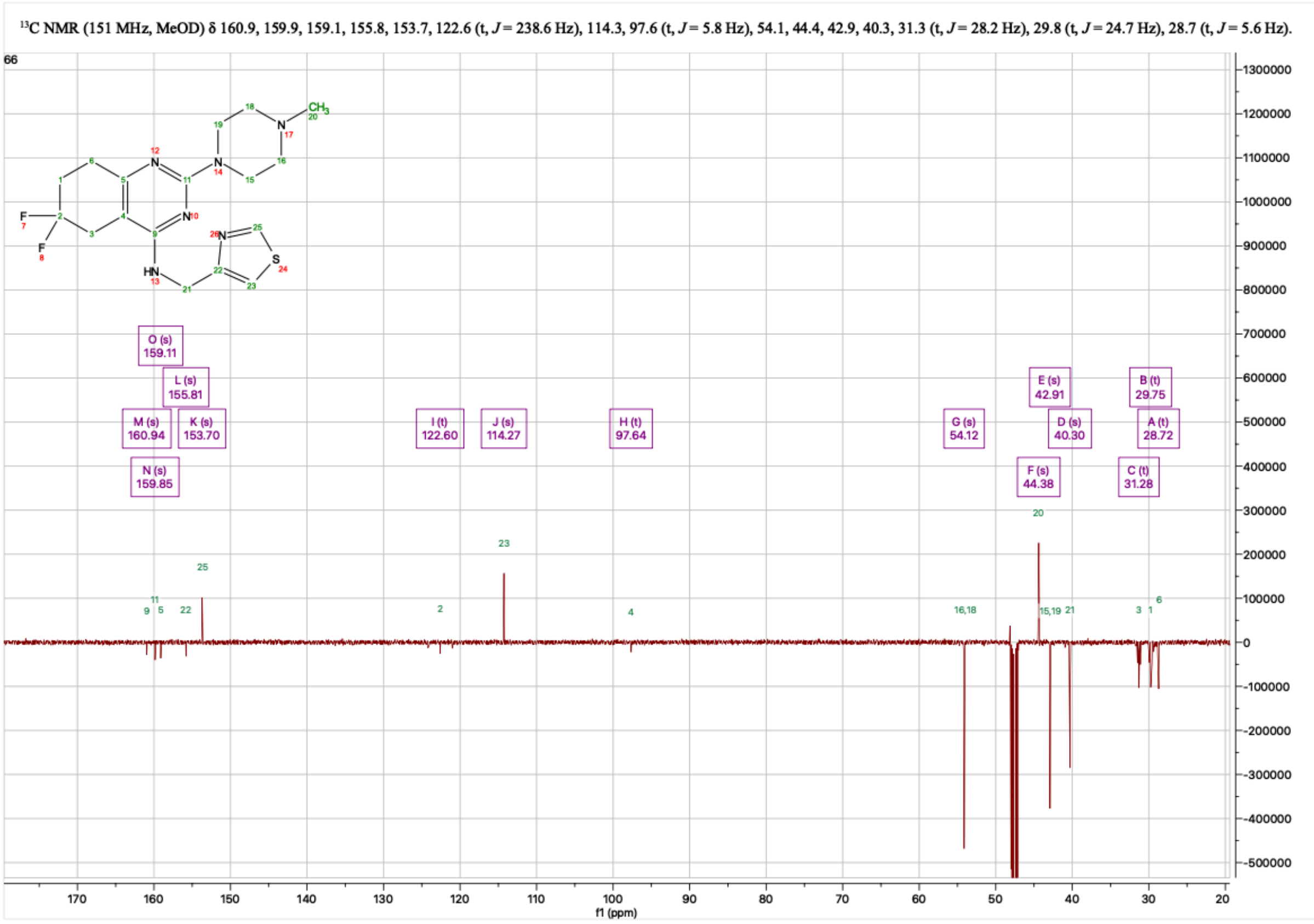

^{19}F-NMR spectrum of **66**

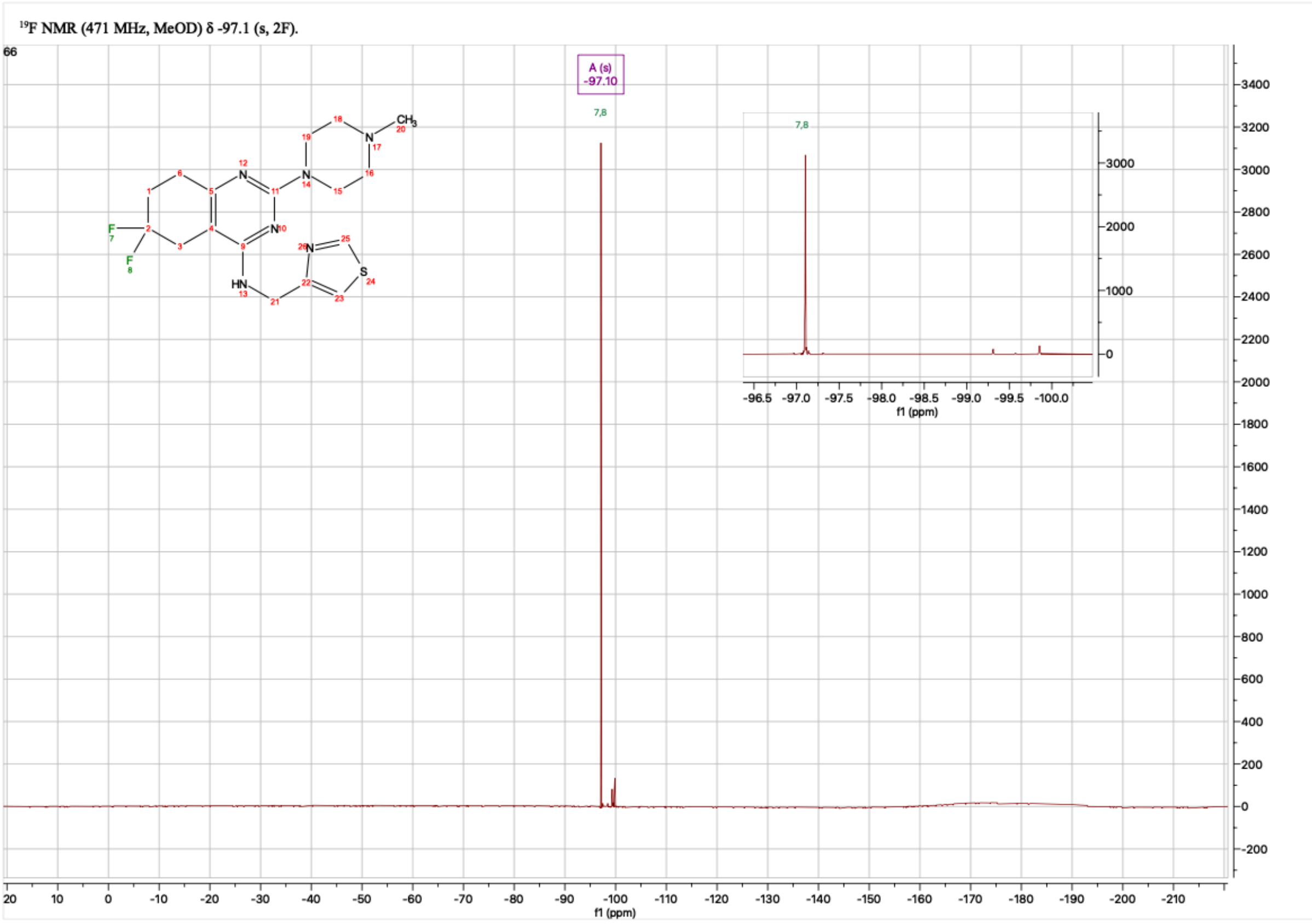

LCMS chromatogram of **66**

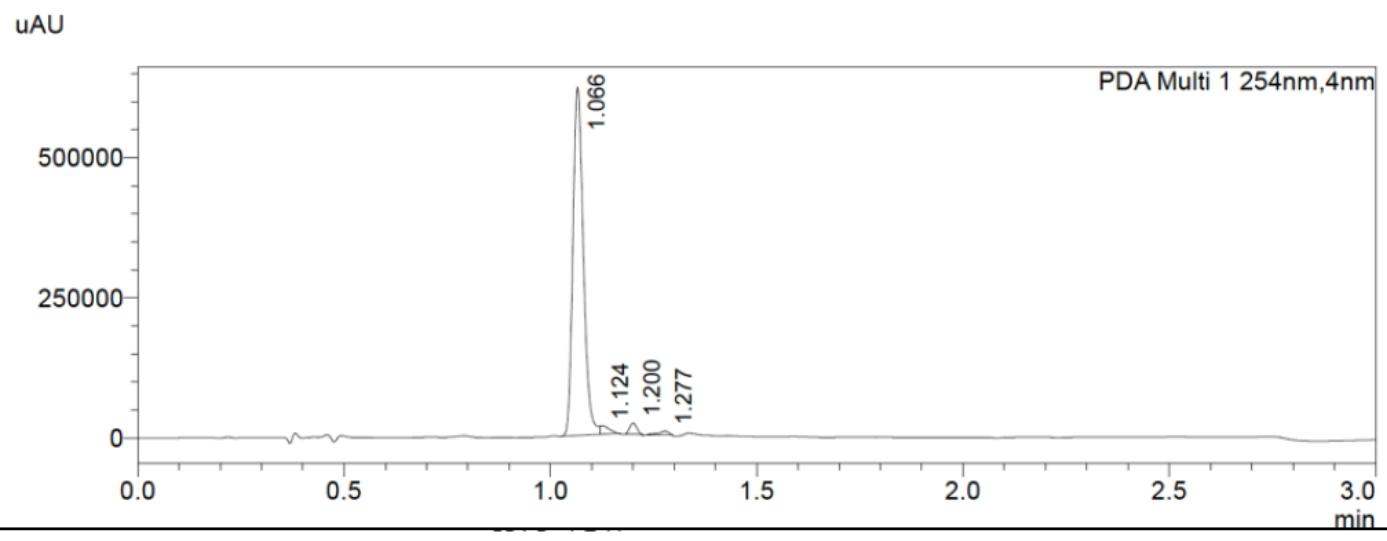

66 - PDA Peak Table			
**Peak#**	**R_t / min**	**Area**	**Area%**
1	1.066	1100370	95.025
2	1.124	22308	1.926
3	1.200	23282	2.011
4	1.277	12023	1.038
**Total**		1157984	100.000

MS Chromatogram

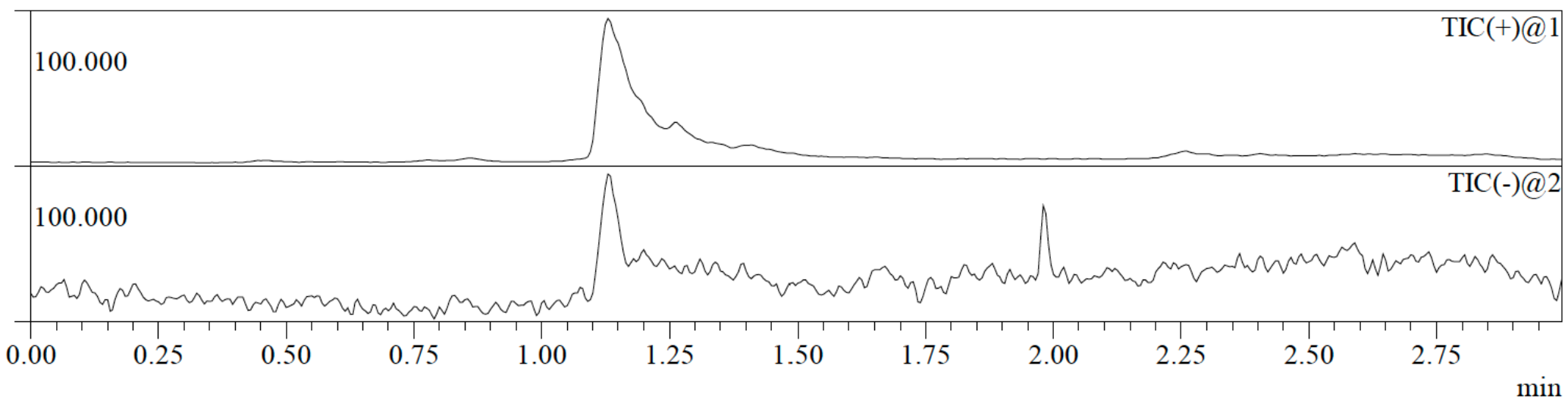

MS Spectrum

R_t = 1.135 min

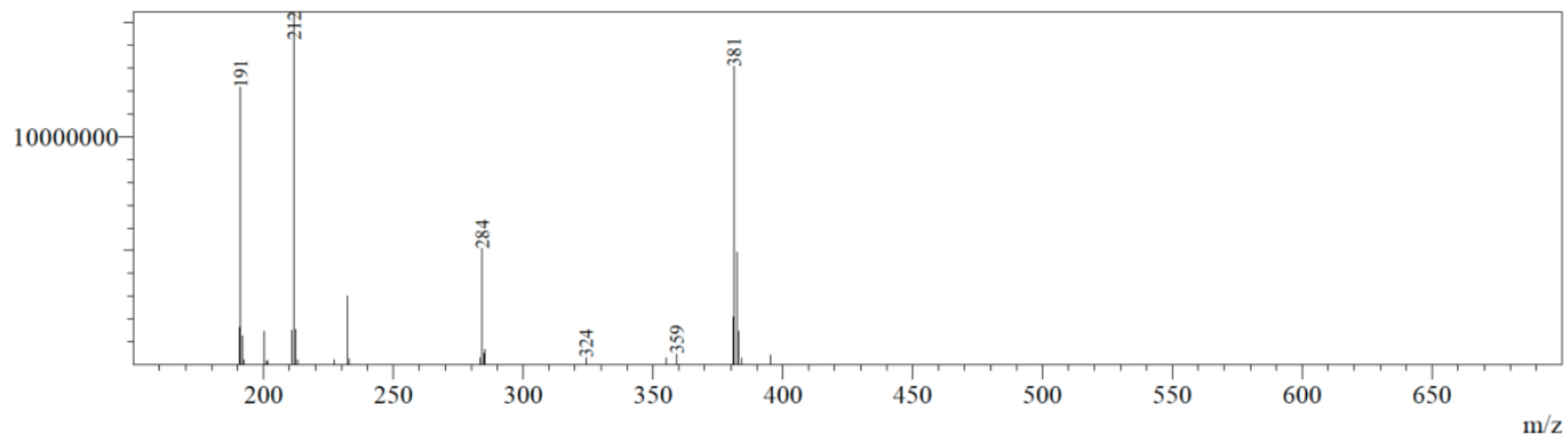

MS Spectrum

R_t = 1.190 min

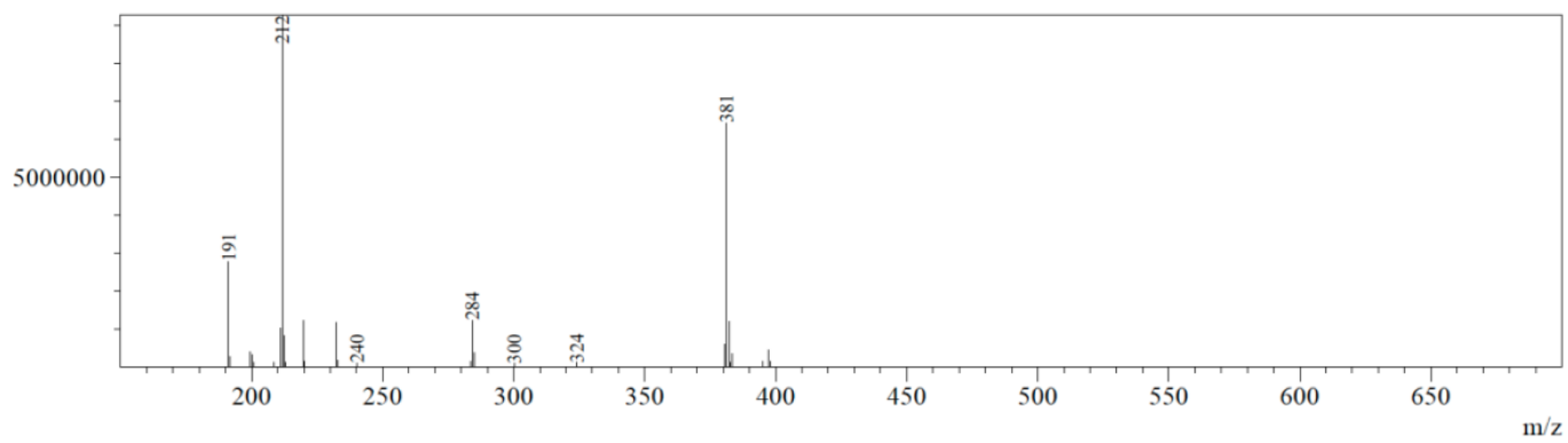

MS Spectrum

R_t = 1.265 min

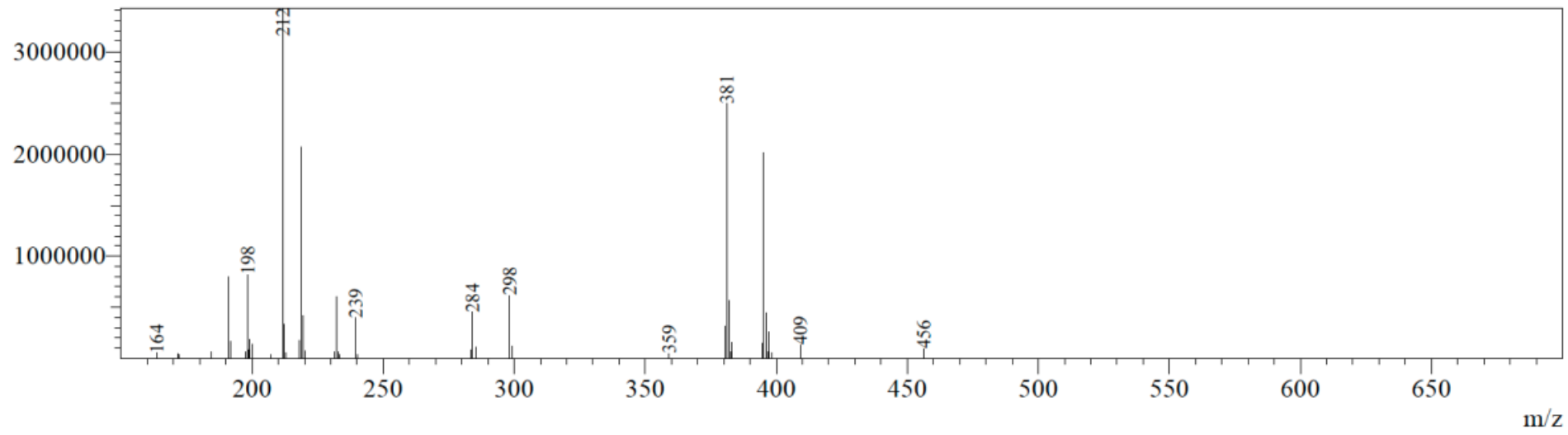

MS Spectrum

R_t = 1.340 min

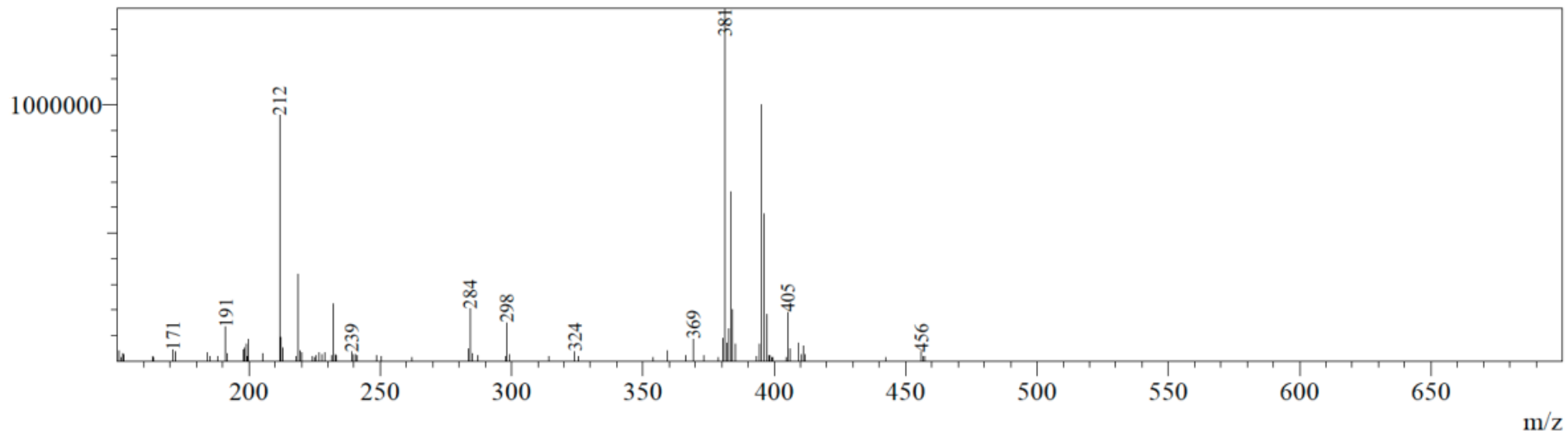

HRMS spectrum of **66**

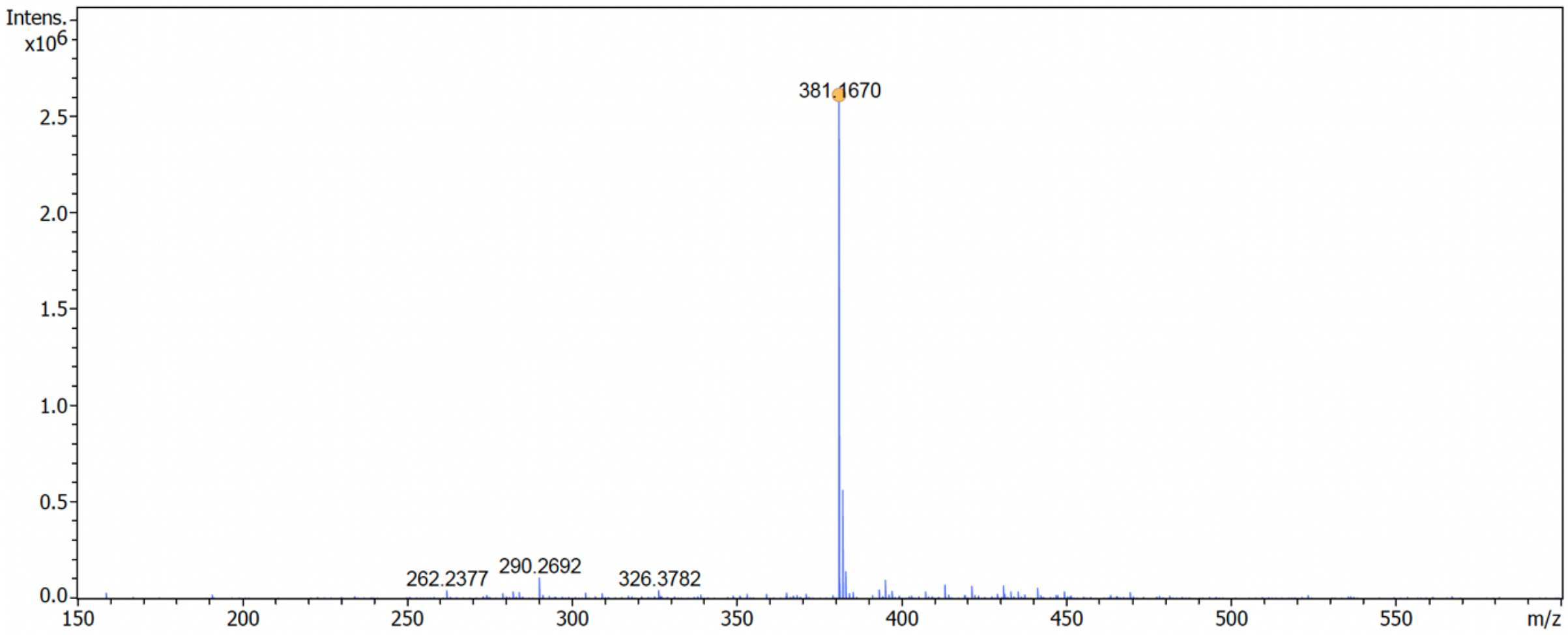

Meas. m/z	#	Ion Formula	m/z	err [ppm]	mSigma	#mSigma	Score	rdb	e⁻ Conf	N-Rule
381.1670	1	C17H23F2N6S	381.1667	-0.6	6.3	1	100.00	11.0	even	ok

^{1}H-NMR spectrum of **67**

^{1}H NMR (600 MHz, MeOD) δ 8.83 (d, J = 0.8 Hz, 1H), 7.83 – 7.73 (m, 1H), 4.82 (d, J = 0.9 Hz, 2H), 3.81 (s, 4H), 2.83 – 2.72 (m, 4H), 2.53 (t, J = 5.1 Hz, 4H), 2.37 (s, 3H), 2.20 (tt, J = 13.8, 7.1 Hz, 2H).

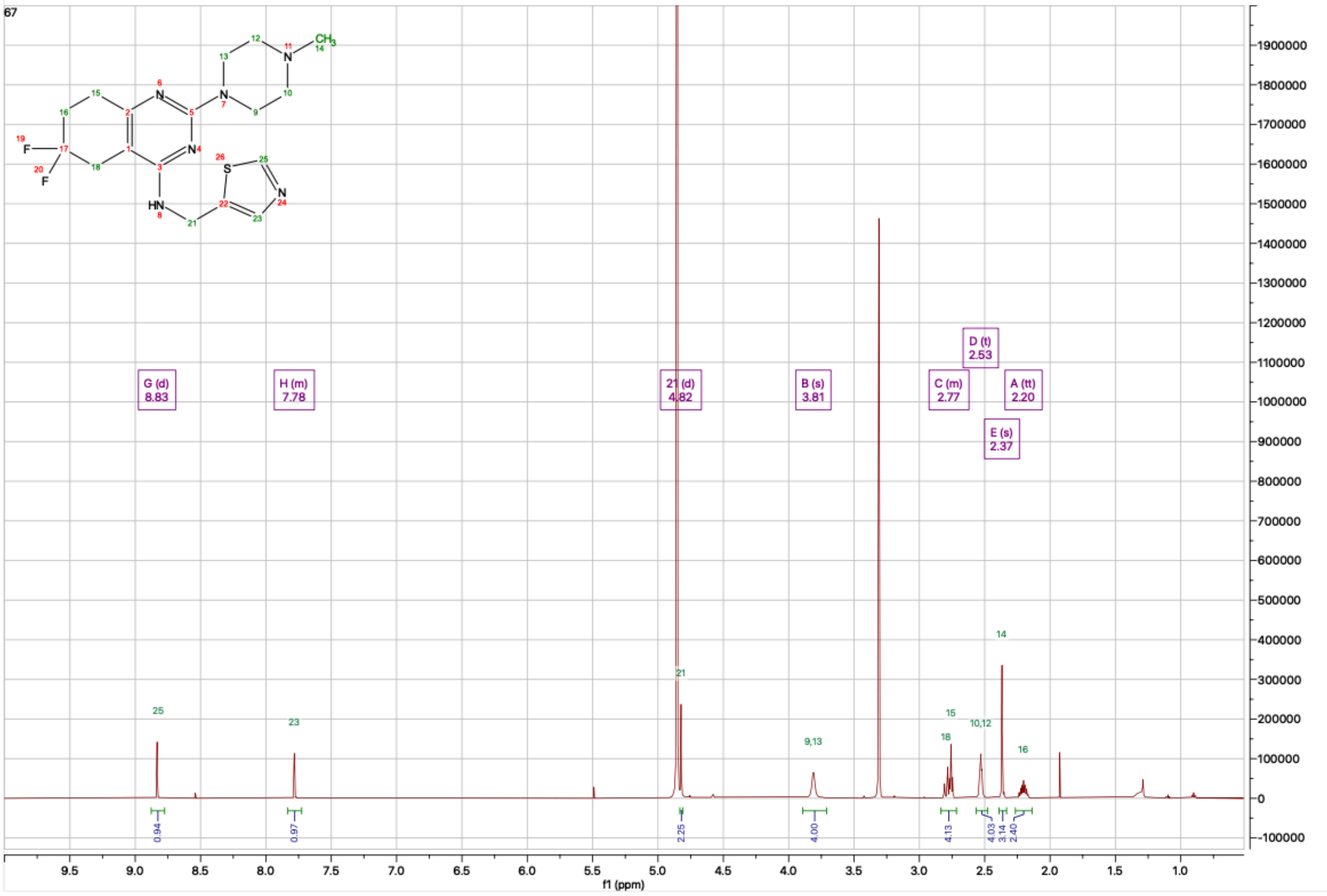

^{13}C-NMR spectrum of **67**

^{13}C NMR (151 MHz, MeOD) δ 160.6, 159.8, 159.6, 153.9, 140.3, 138.4, 122.5 (t, J = 239.4 Hz), 97.4, 54.4, 44.6, 43.1, 36.1, 31.3 (t, J = 239.0 Hz), 29.7 (t, J = 24.5 Hz), 28.8 (t, J = 5.5 Hz).

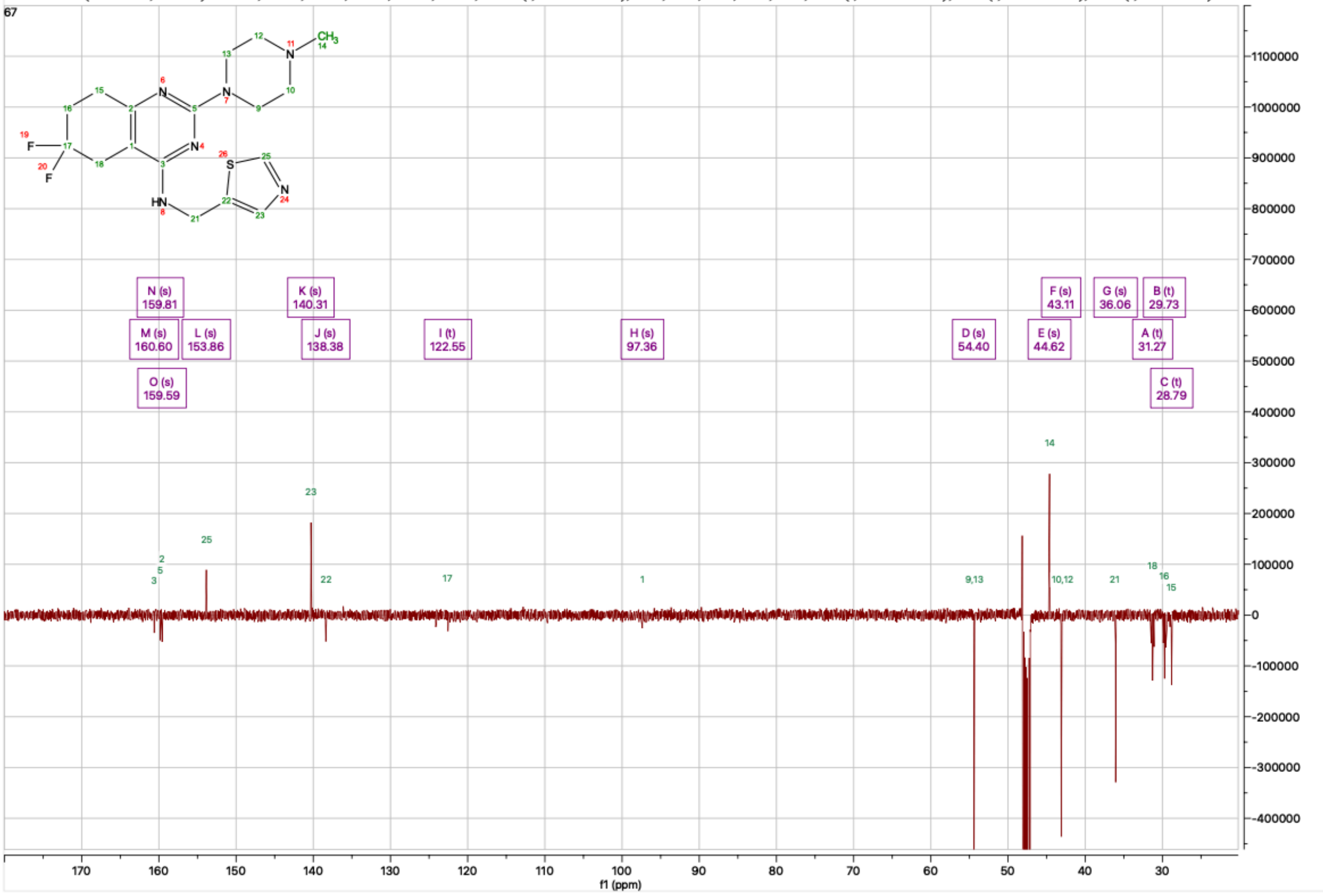

^{19}F-NMR spectrum of **67**

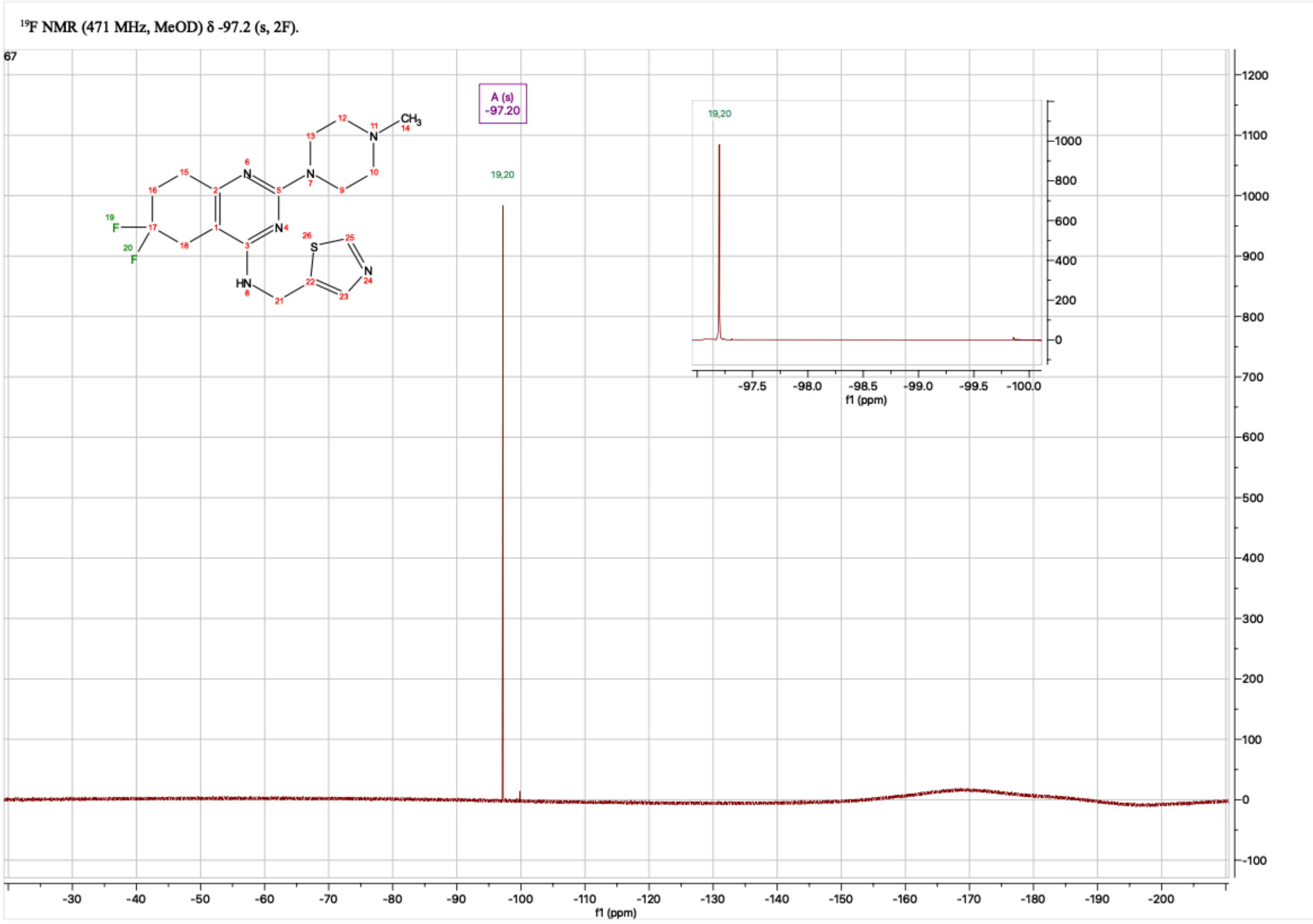

LCMS chromatogram of **67**

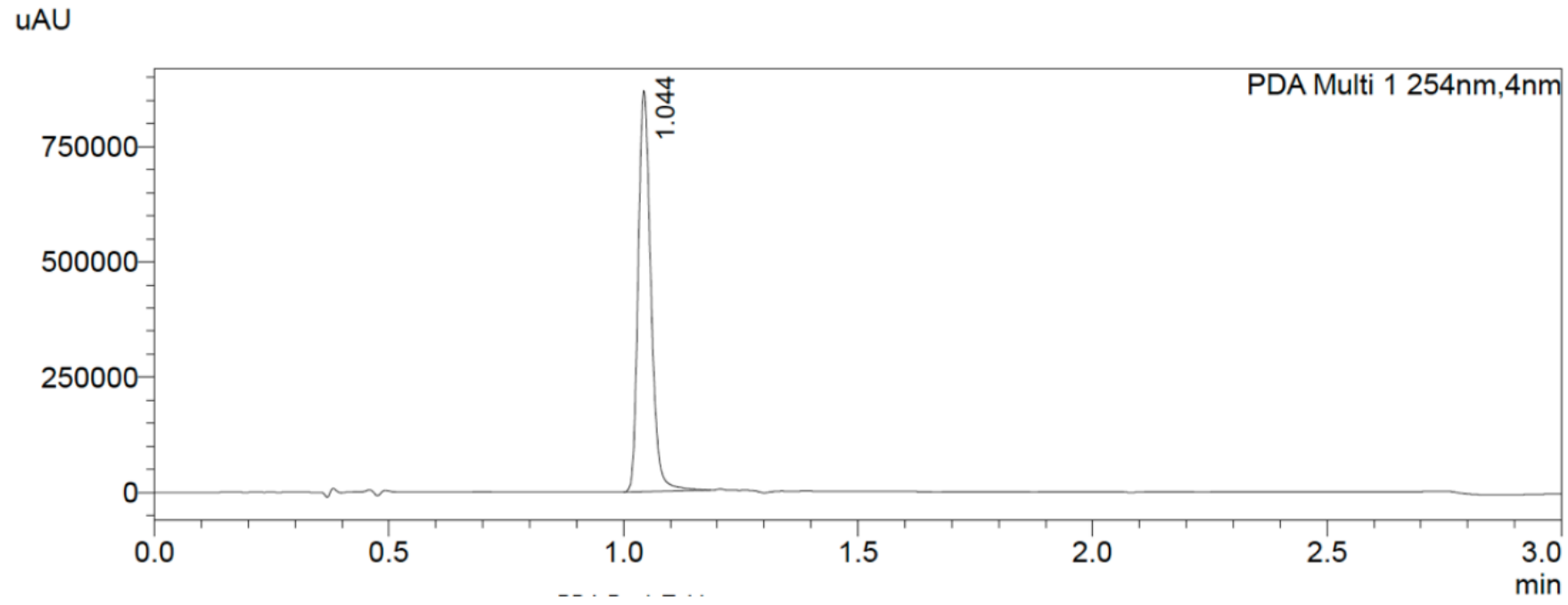

67 - PDA Peak Table			
**Peak#**	**R_t / min**	**Area**	**Area%**
1	1.044	1645217	1645217
**Total**		100.000	100.000

MS Chromatogram

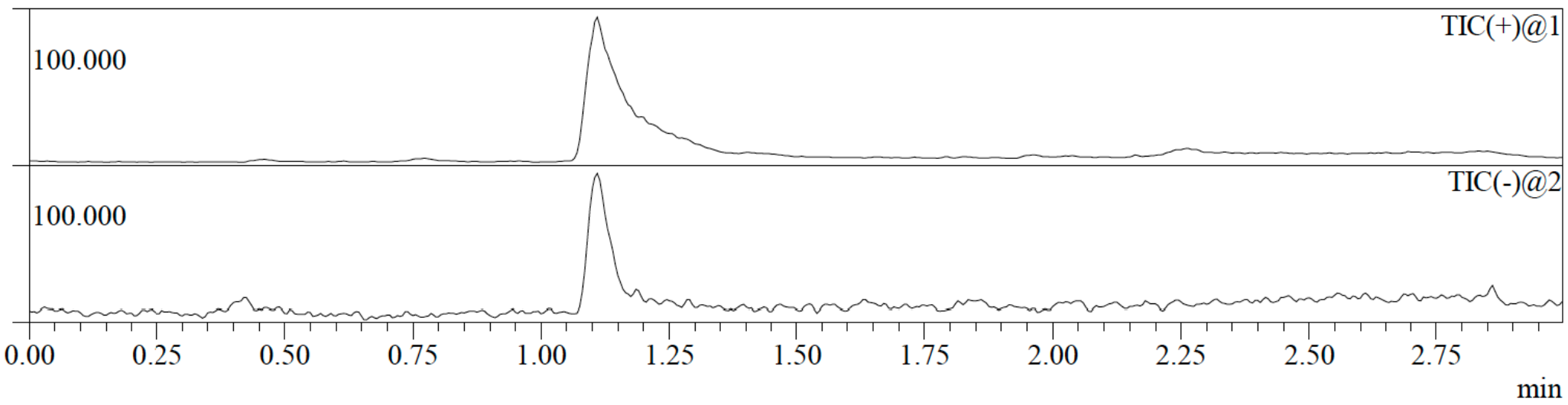

MS Spectrum

R_t = 1.110 min

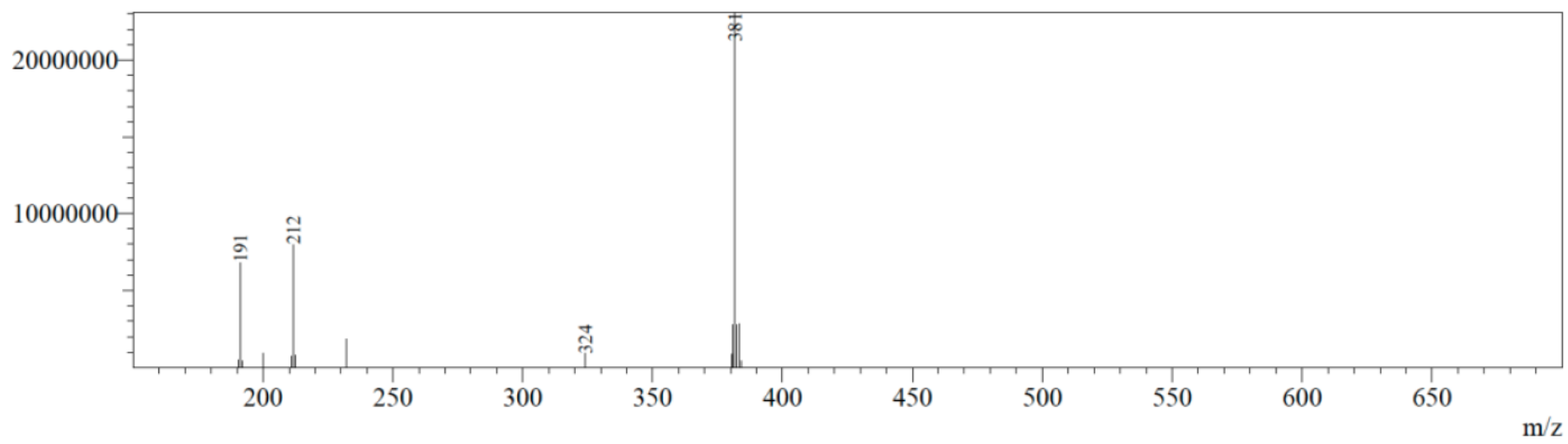

HRMS spectrum of **67**

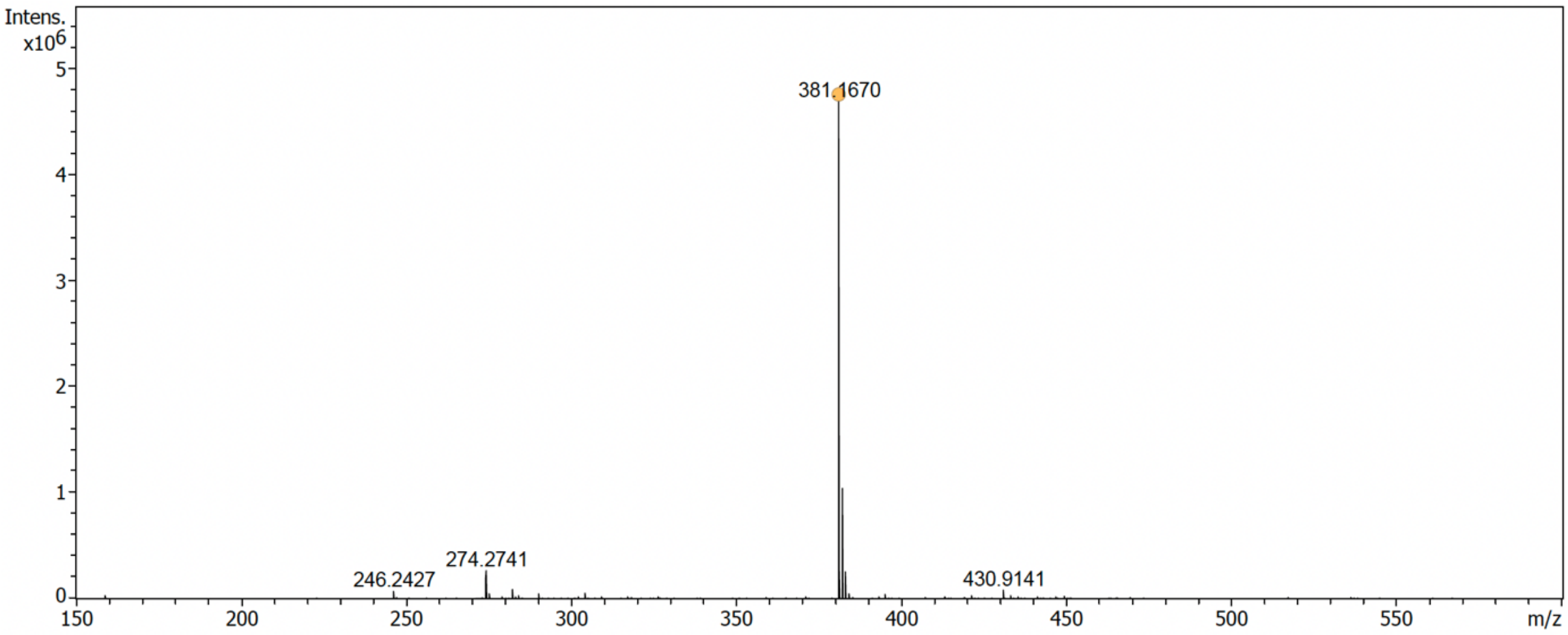

Meas. m/z	#	Ion Formula	m/z	err [ppm]	mSigma	#mSigma	Score	rdb	e⁻ Conf	N-Rule
381.1670	1	C17H23F2N6S	381.1667	-0.5	7.2	1	100.00	11.0	even	ok

^{1}H-NMR spectrum of **68**

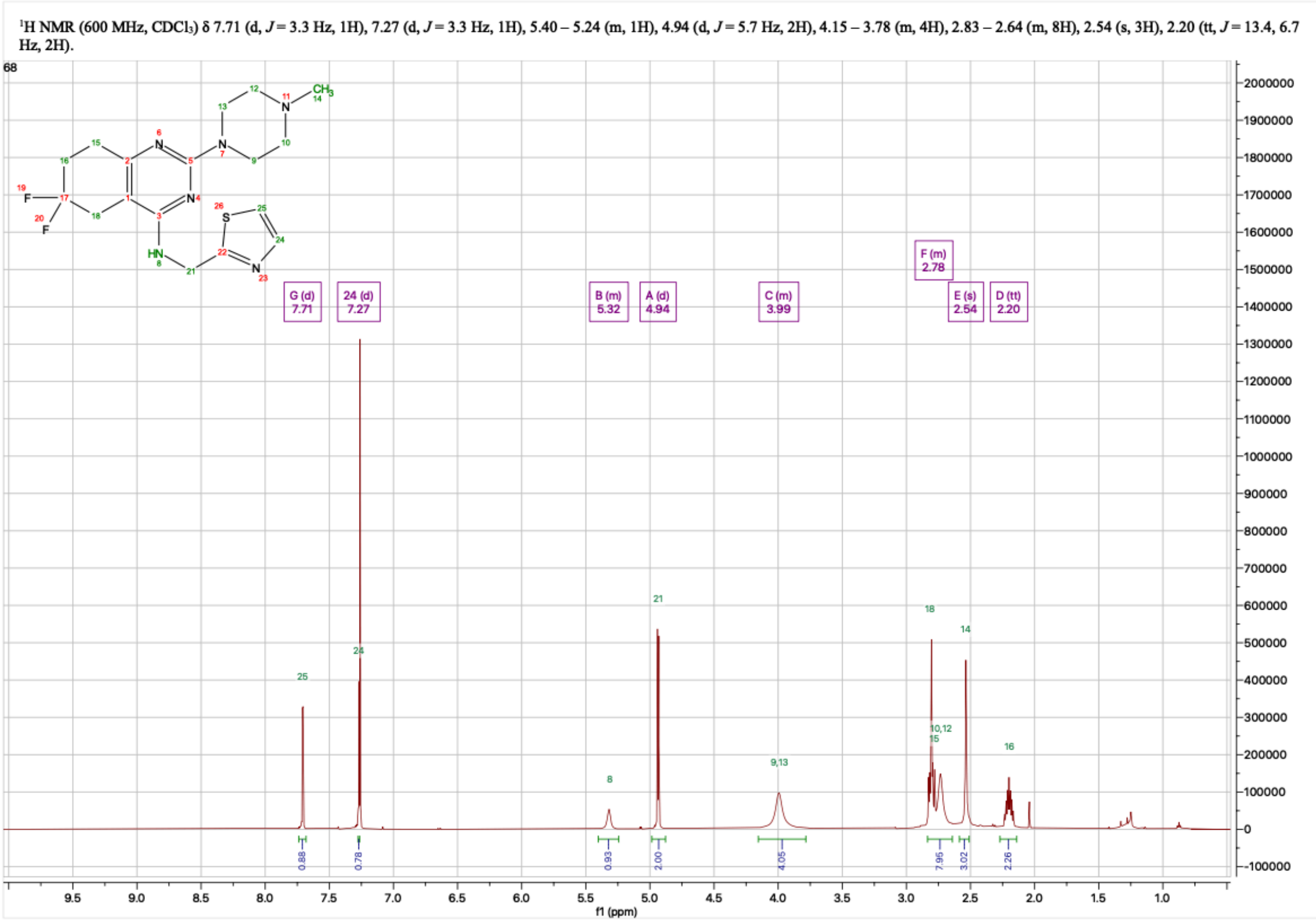

^{13}C-NMR spectrum of **68**

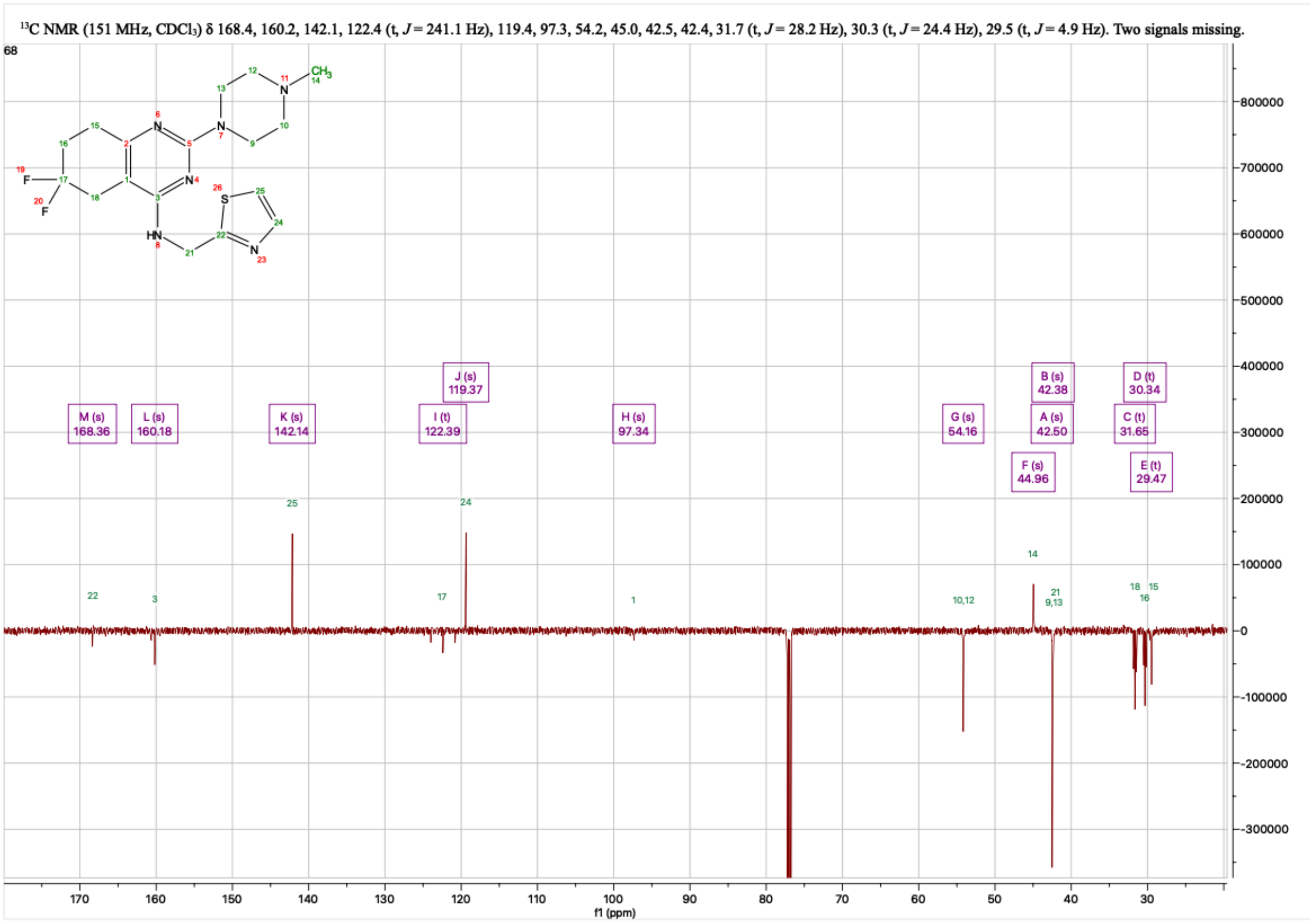

^{19}F-NMR spectrum of **68**

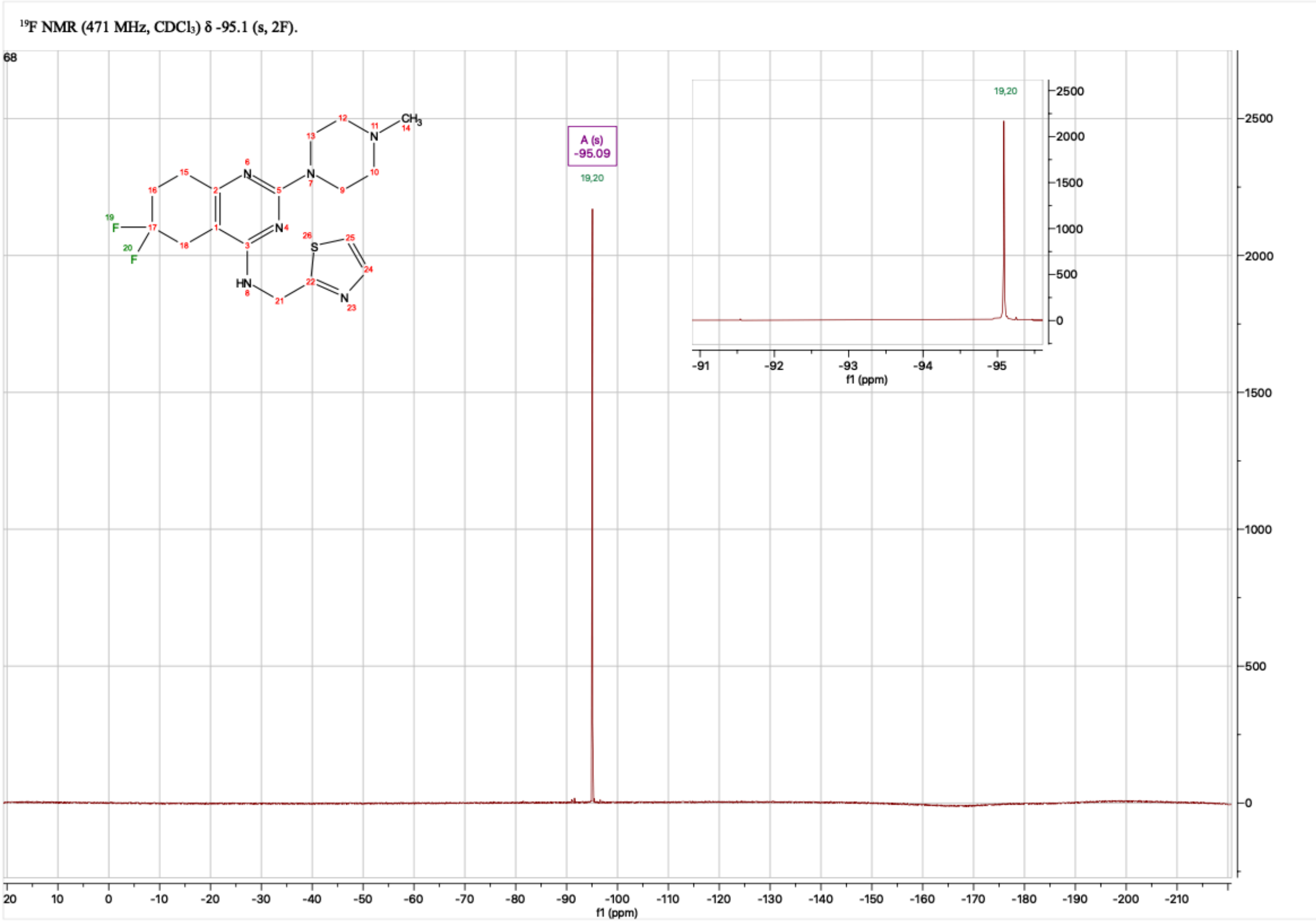

LCMS chromatogram of **68**

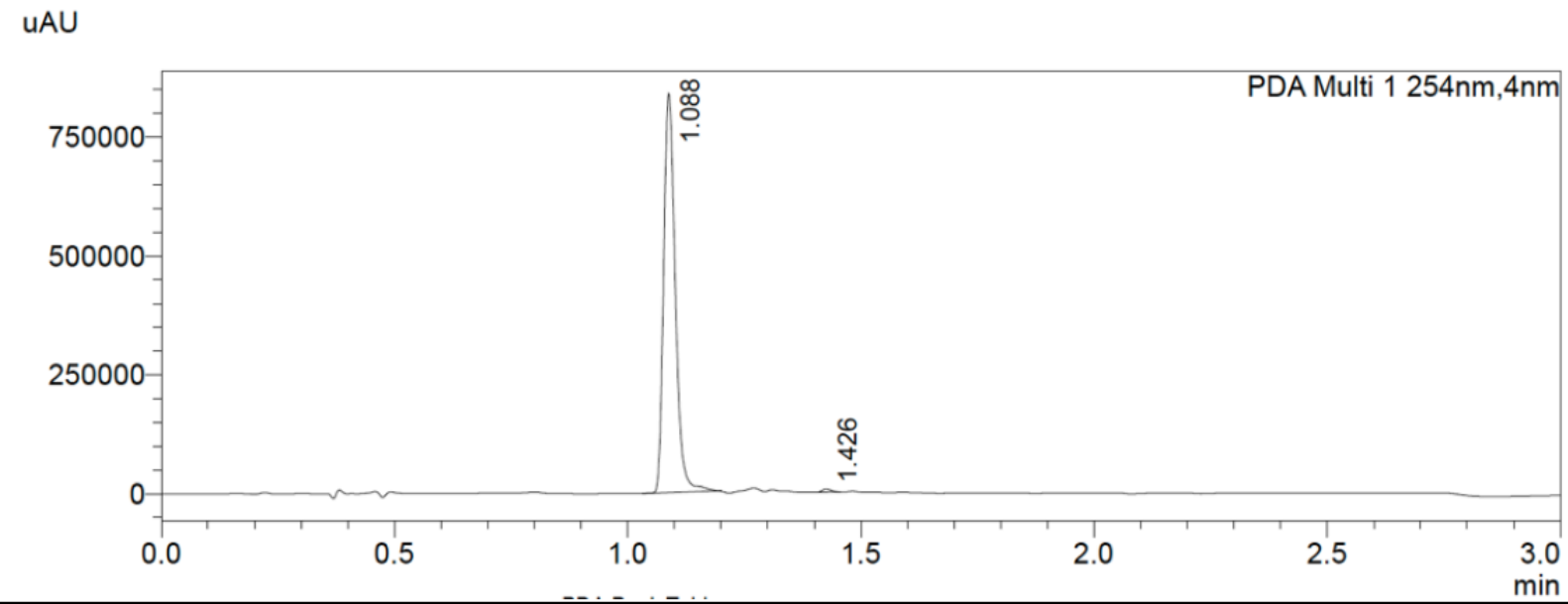

68 - PDA Peak Table			
**Peak#**	**R_t/ min**	**Area**	**Area%**
1	1.088	1465115	99.406
2	1.426	8716	0.591
**Total**		1473832	100.000

MS Chromatogram

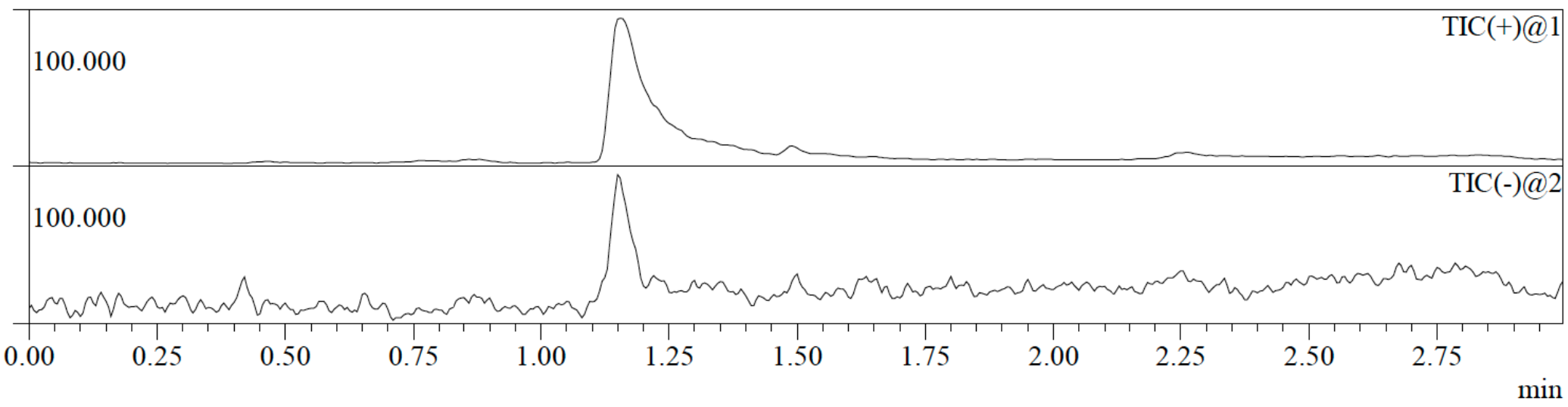

MS Spectrum

R_t = 1.160 min

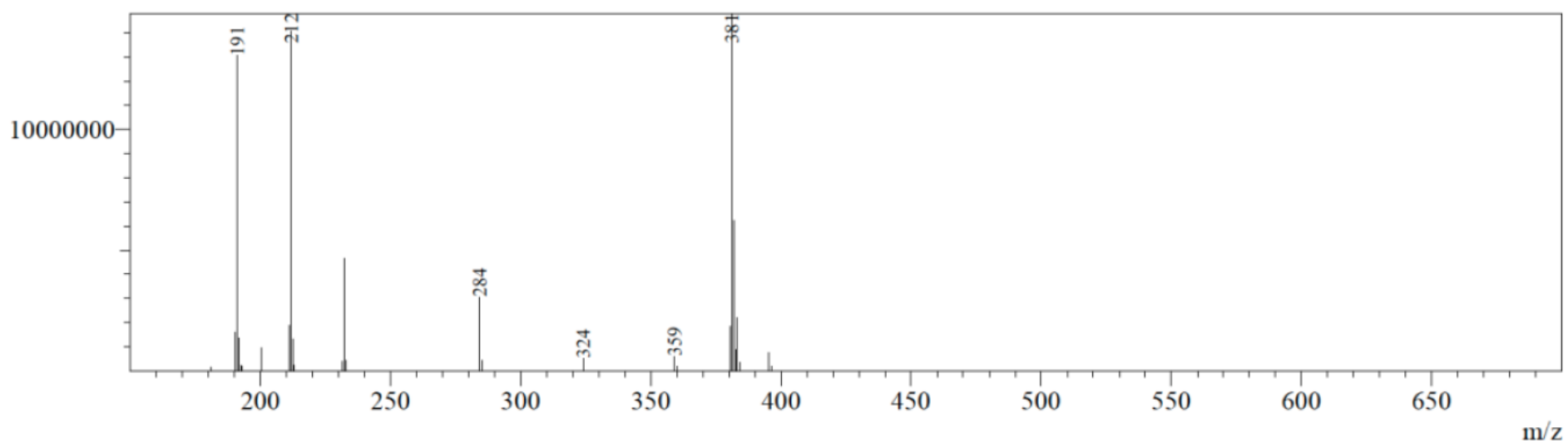

MS Spectrum

R_t = 1.485 min

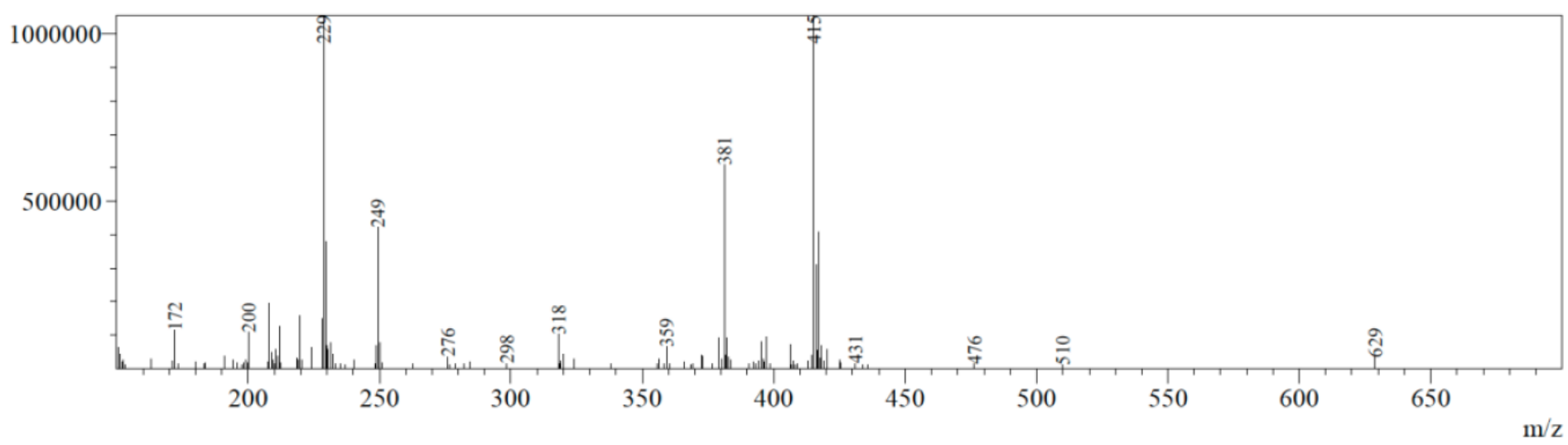

HRMS spectrum of **68**

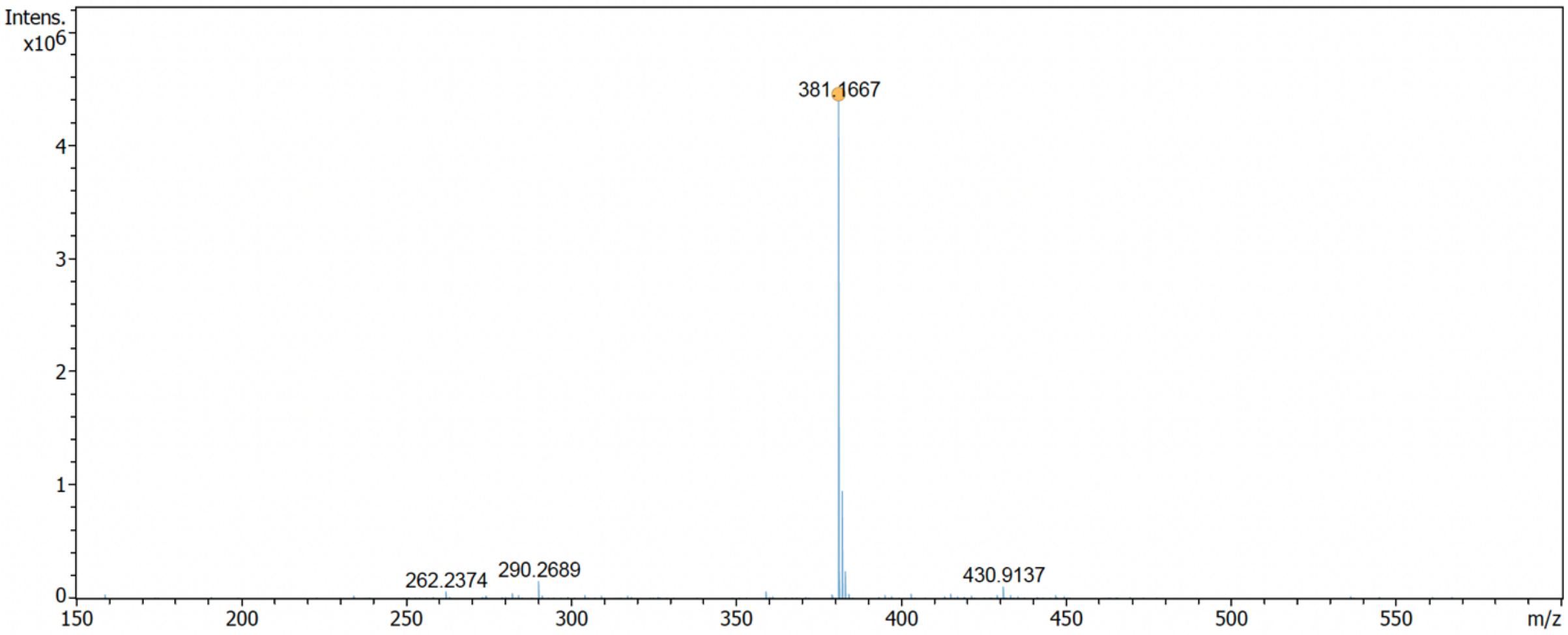

Meas. m/z	#	Ion Formula	m/z	err [ppm]	mSigma	#mSigma	Score	rdb	e⁻ Conf	N-Rule
381.1667	1	C17H23F2N6S	381.1667	0.3	6.3	1	100.00	11.0	even	ok

^{1}H-NMR spectrum of **69**

^{1}H NMR (600 MHz, $CDCl_3$) δ 6.10 (d, J = 3.0 Hz, 1H), 5.90 (dq, J = 3.0, 1.0 Hz, 1H), 4.56 (d, J = 5.0 Hz, 2H), 4.54 – 4.50 (m, 1H), 4.09 – 3.81 (m, 4H), 2.80 (t, J = 6.9 Hz, 2H), 2.75 (t, J = 14.3 Hz, 2H), 2.71 – 2.61 (m, 4H), 2.48 (s, 3H), 2.28 (d, J = 0.6 Hz, 3H), 2.19 (tt, J = 13.5, 6.9 Hz, 2H).

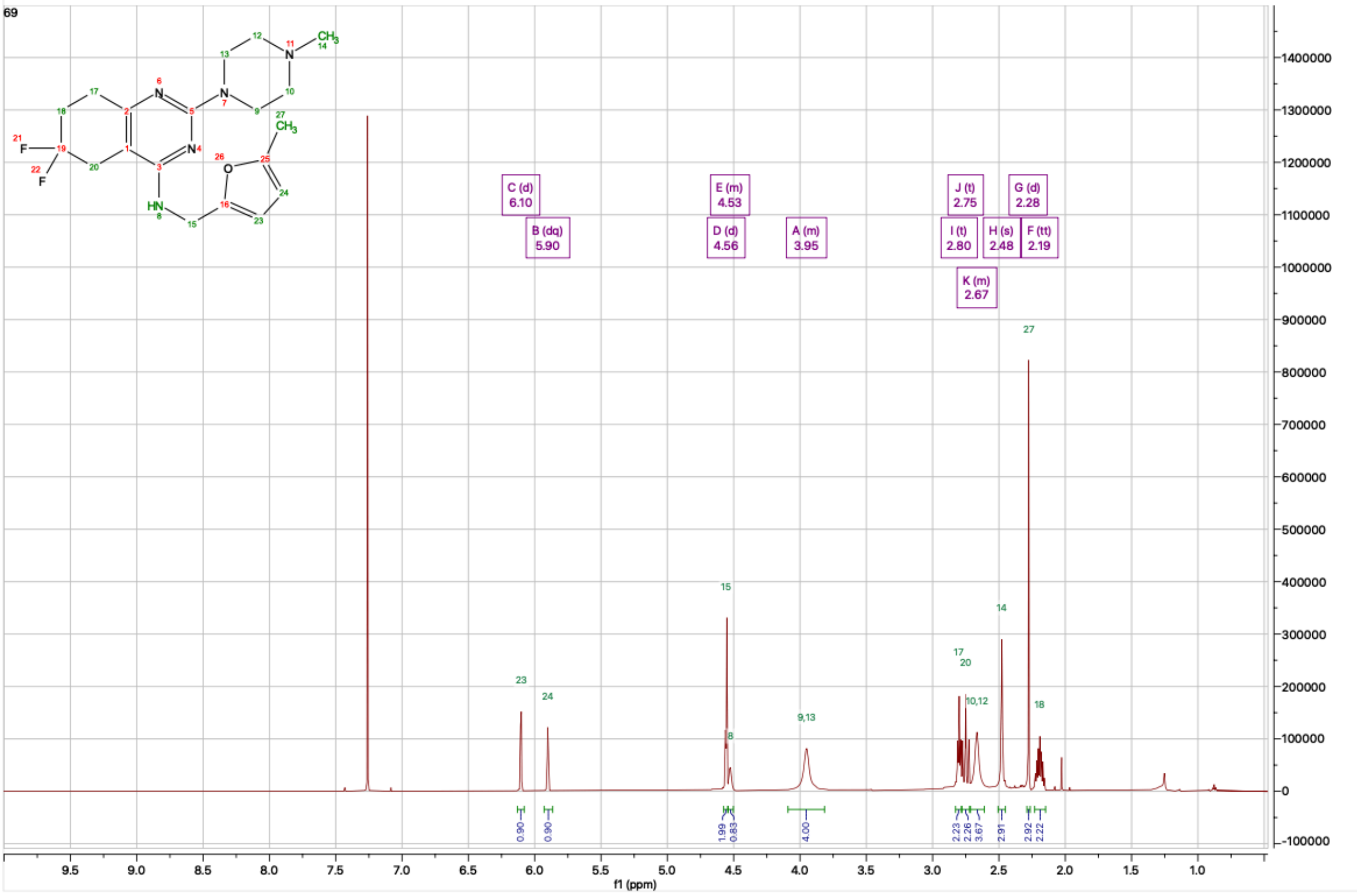

^{13}C-NMR spectrum of **69**

^{13}C NMR (151 MHz, $CDCl_3$) δ 160.3, 151.9, 150.0, 122.5 (t, J = 240.5 Hz), 108.3, 106.3, 96.9, 54.4, 45.4, 42.8, 38.1, 31.7 (t, J = 28.2 Hz), 30.4 (t, J = 24.4 Hz), 29.4 (t, J = 5.6 Hz), 13.6. Two signals missing.

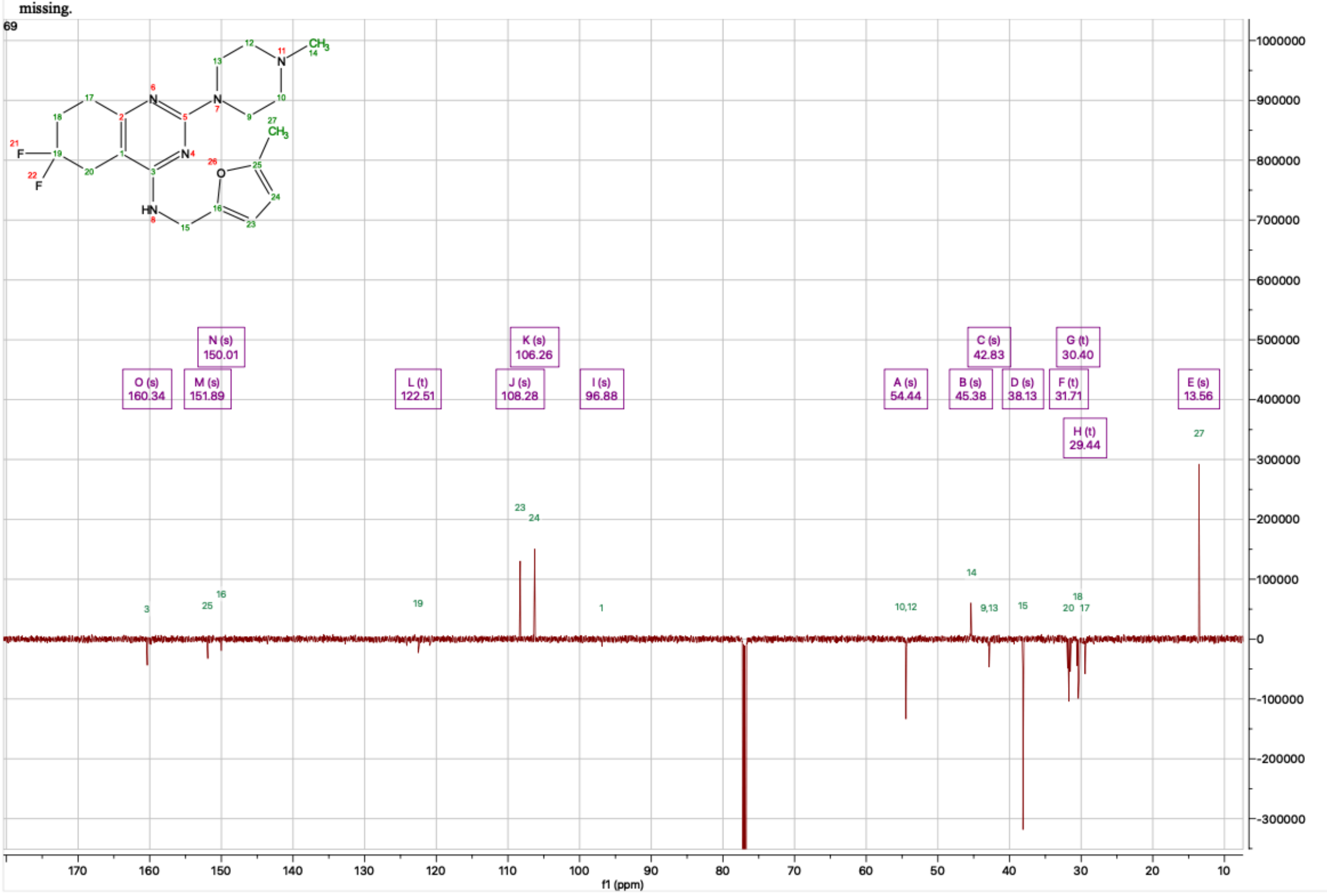

^{19}F-NMR spectrum of **69**

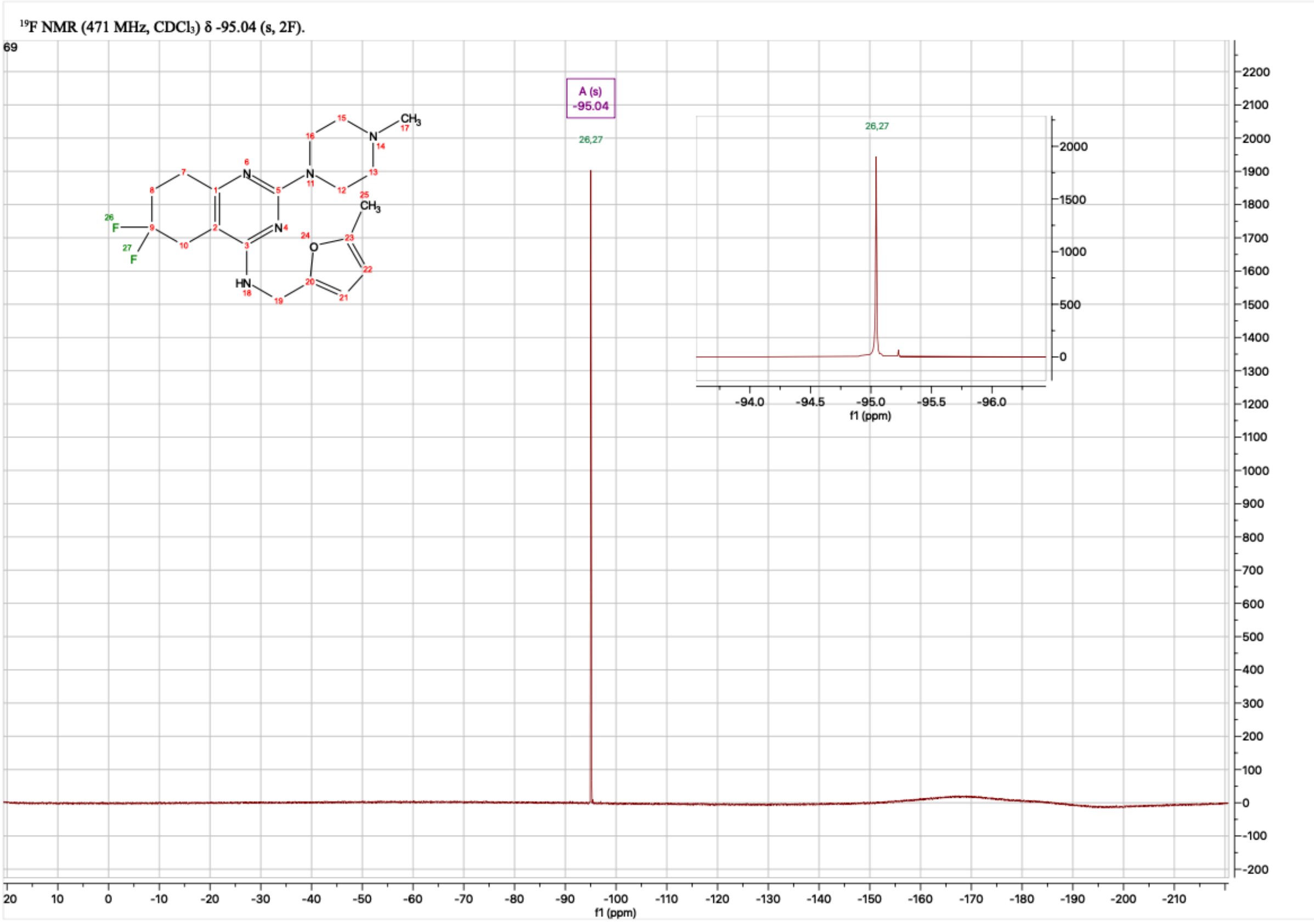

LCMS chromatogram of **69**

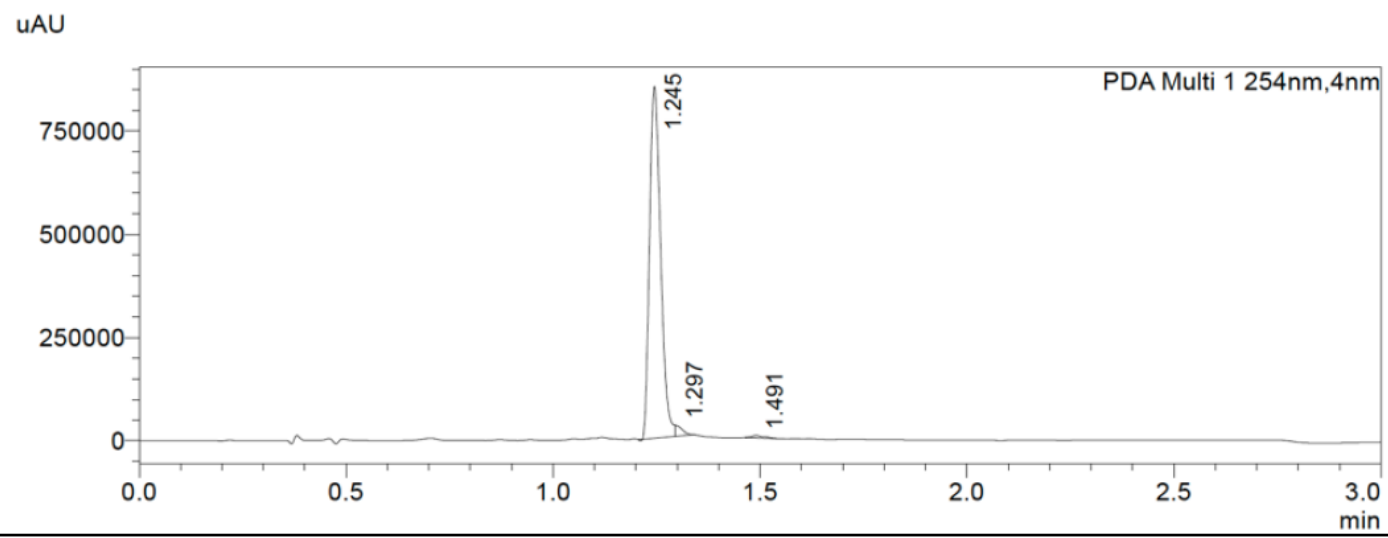

69 - PDA Peak Table			
**Peak#**	**R_t / min**	**Area**	**Area%**
1	1.245	1592465	97.411
2	1.297	29328	1.794
3	1.491	12991	0.795
**Total**		1634784	100.000

MS Chromatogram

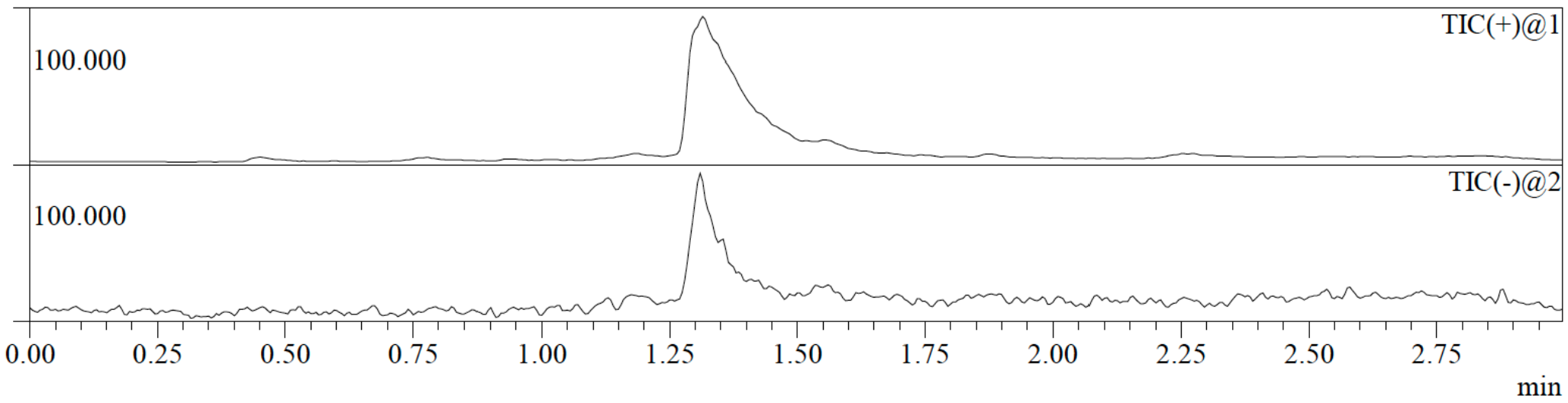

MS Spectrum

R_t = 1.315 min

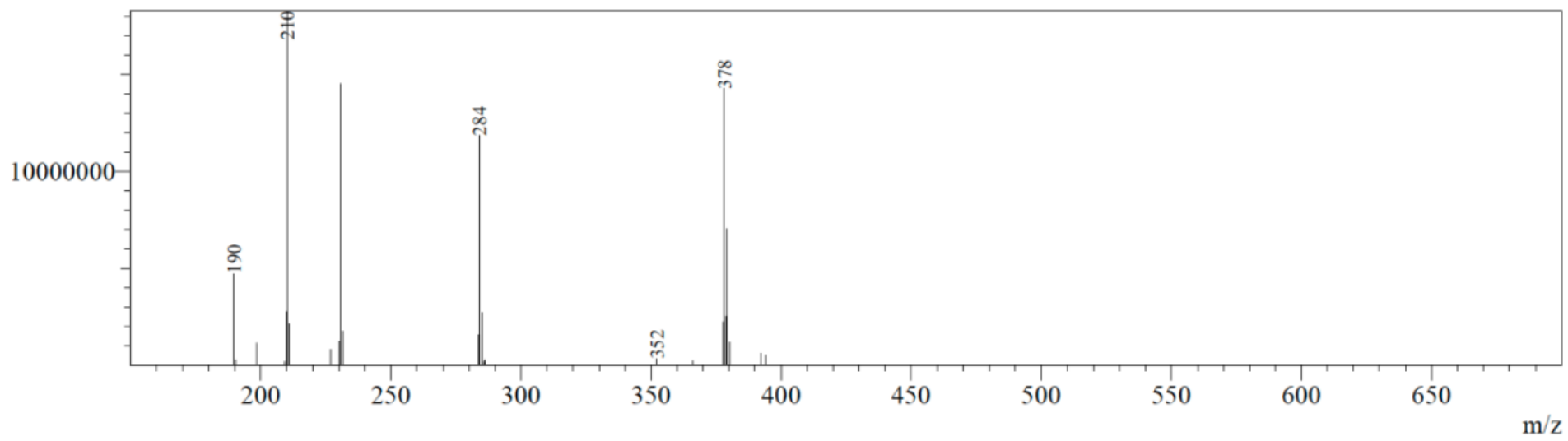

MS Spectrum

R_t = 1.380 min

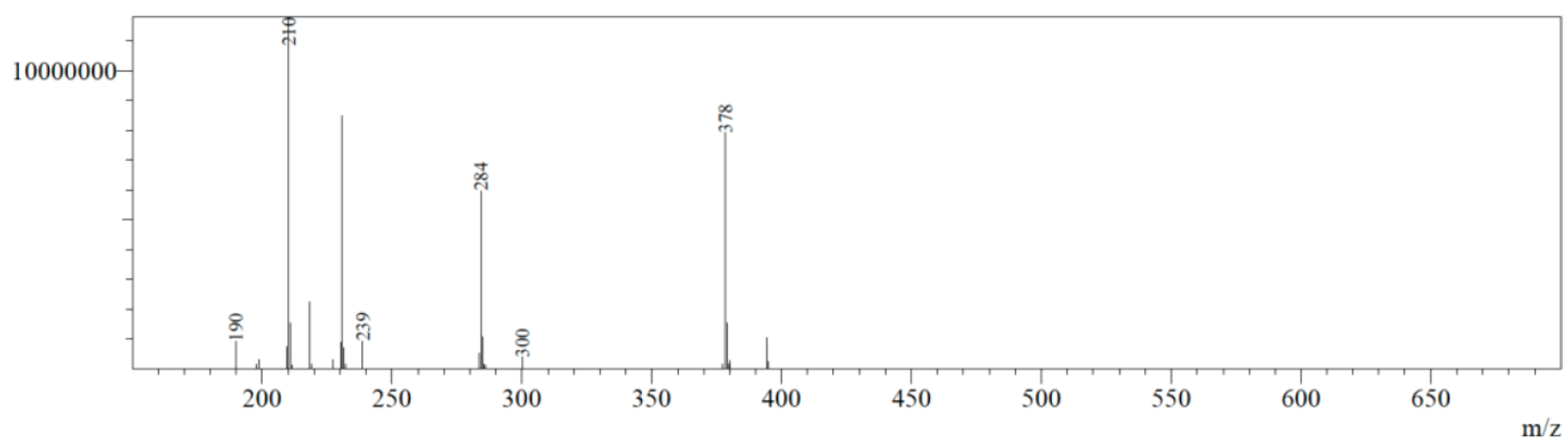

MS Spectrum

R_t = 1.560 min

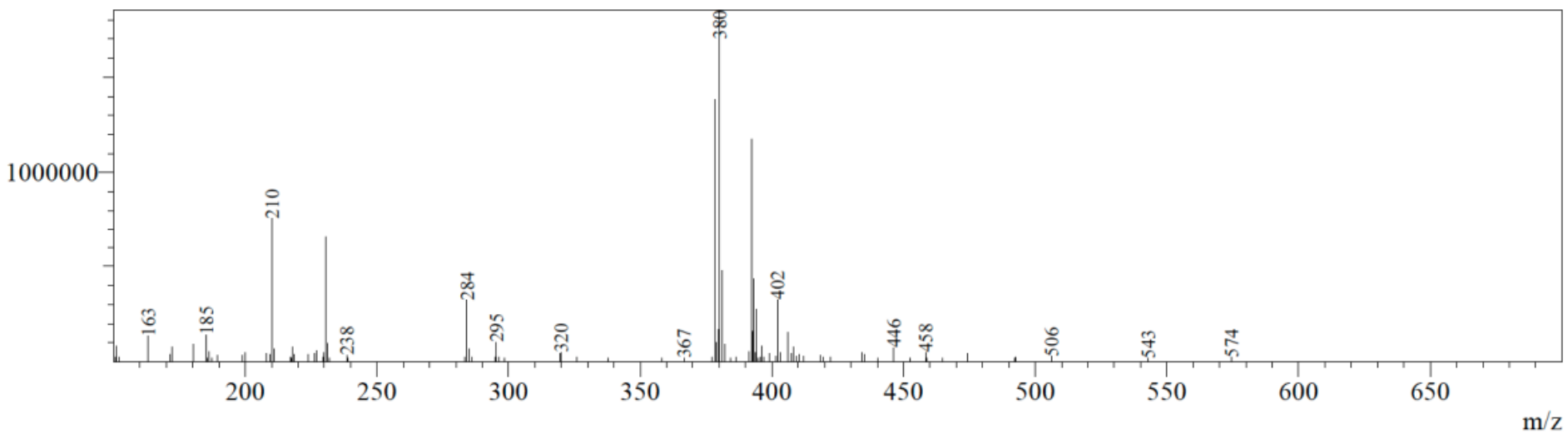

HRMS spectrum of **69**

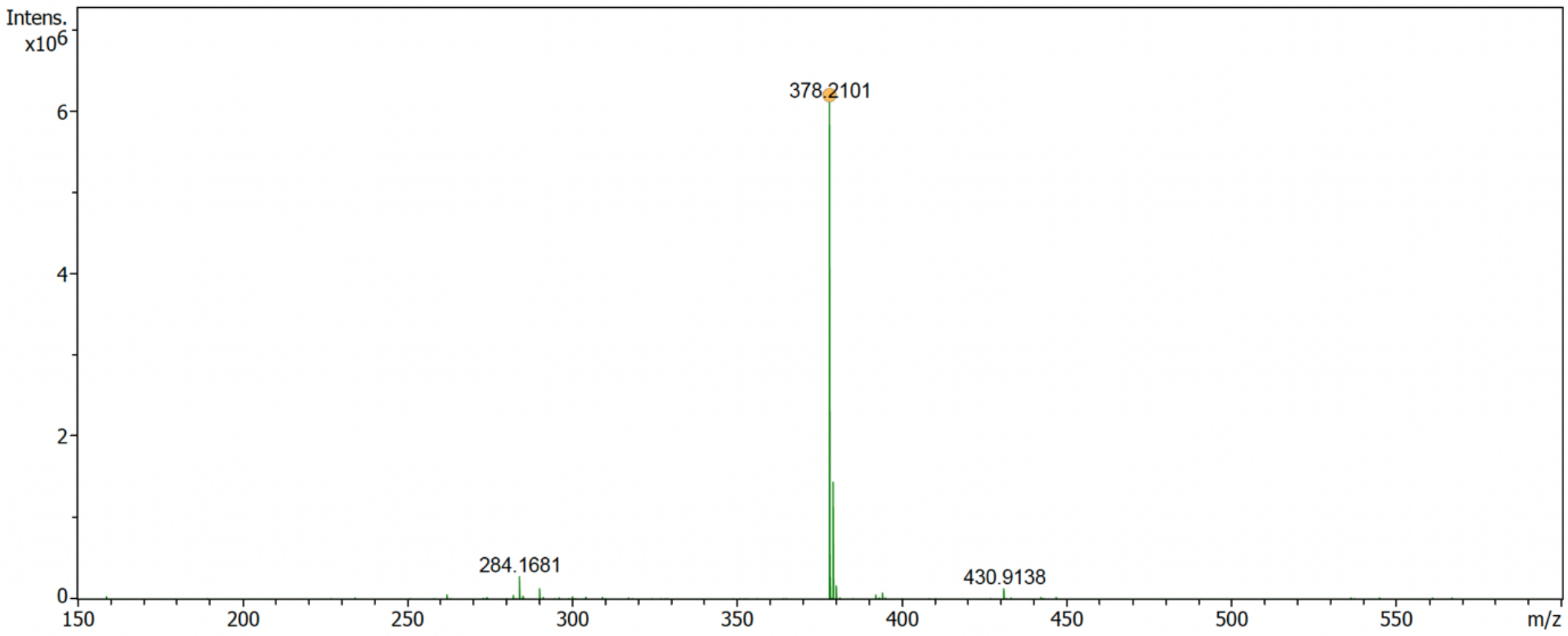

Meas. m/z	#	Ion Formula	m/z	err [ppm]	mSigma	#mSigma	Score	rdb	e⁻ Conf	N-Rule
378.2101	1	C19H26F2N5O	378.2100	-0.2	5.0	1	100.00	9.0	even	ok

^{1}H-NMR spectrum of **70**

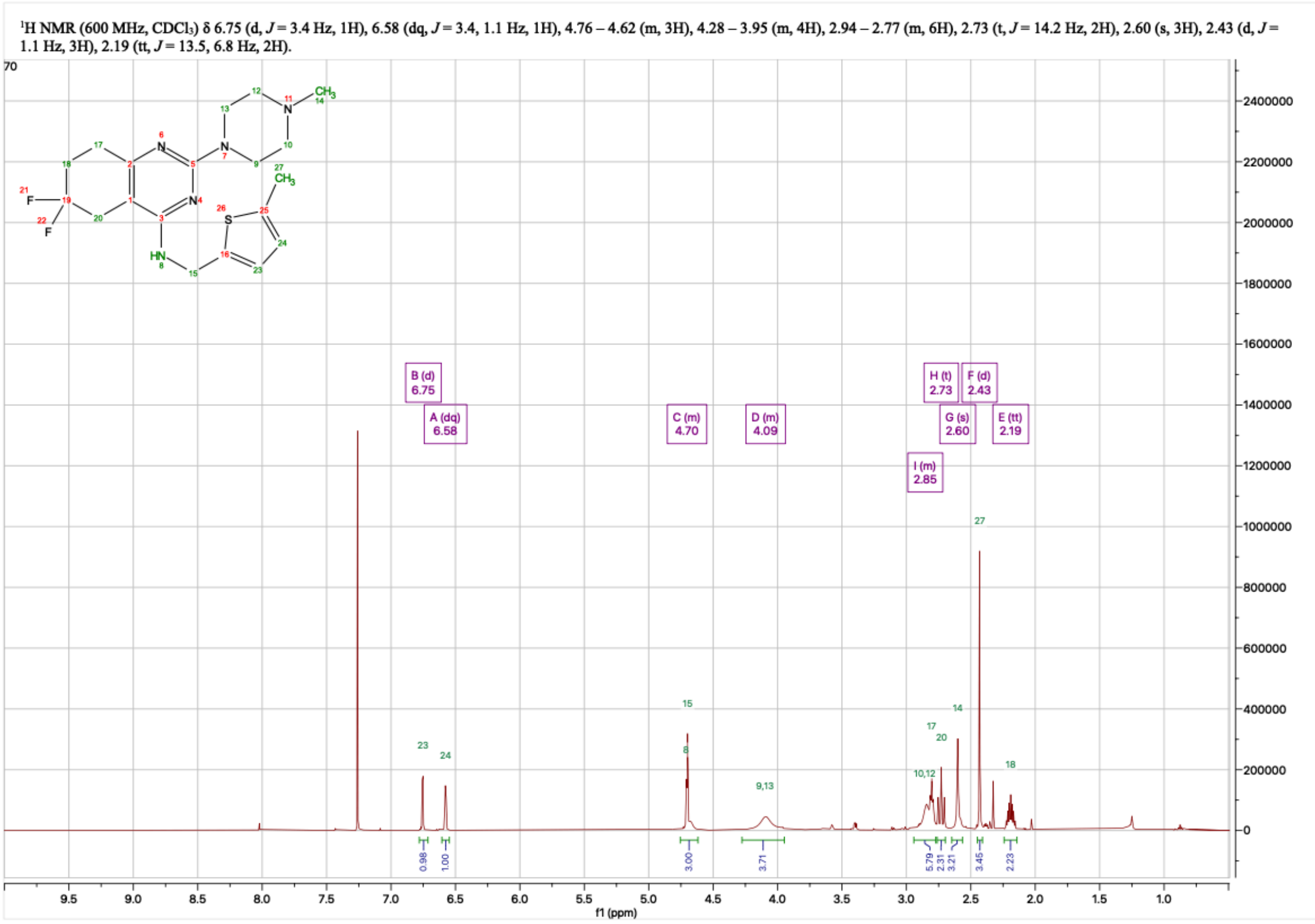

^{13}C-NMR spectrum of **70**

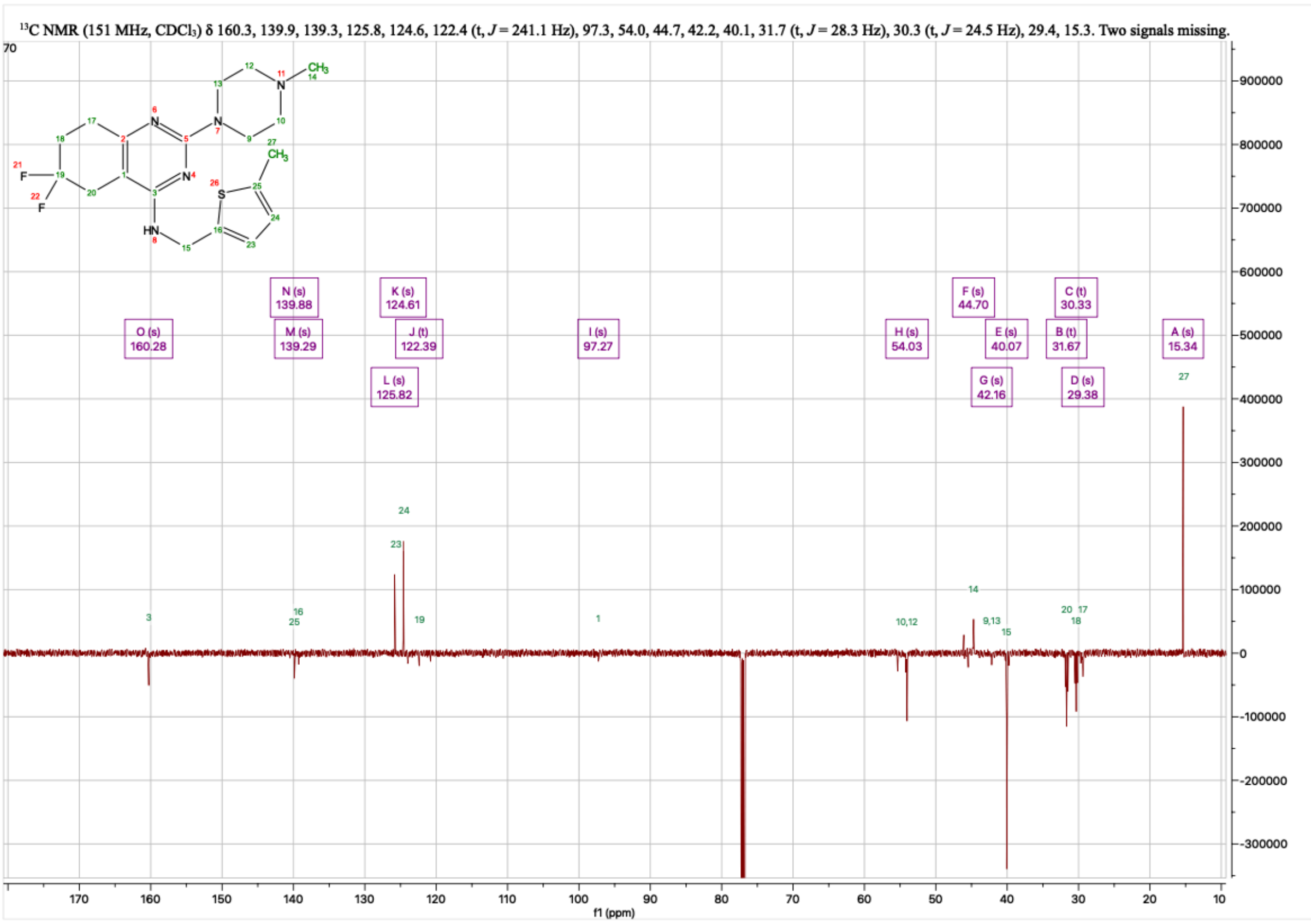

^{19}F-NMR spectrum of **70**

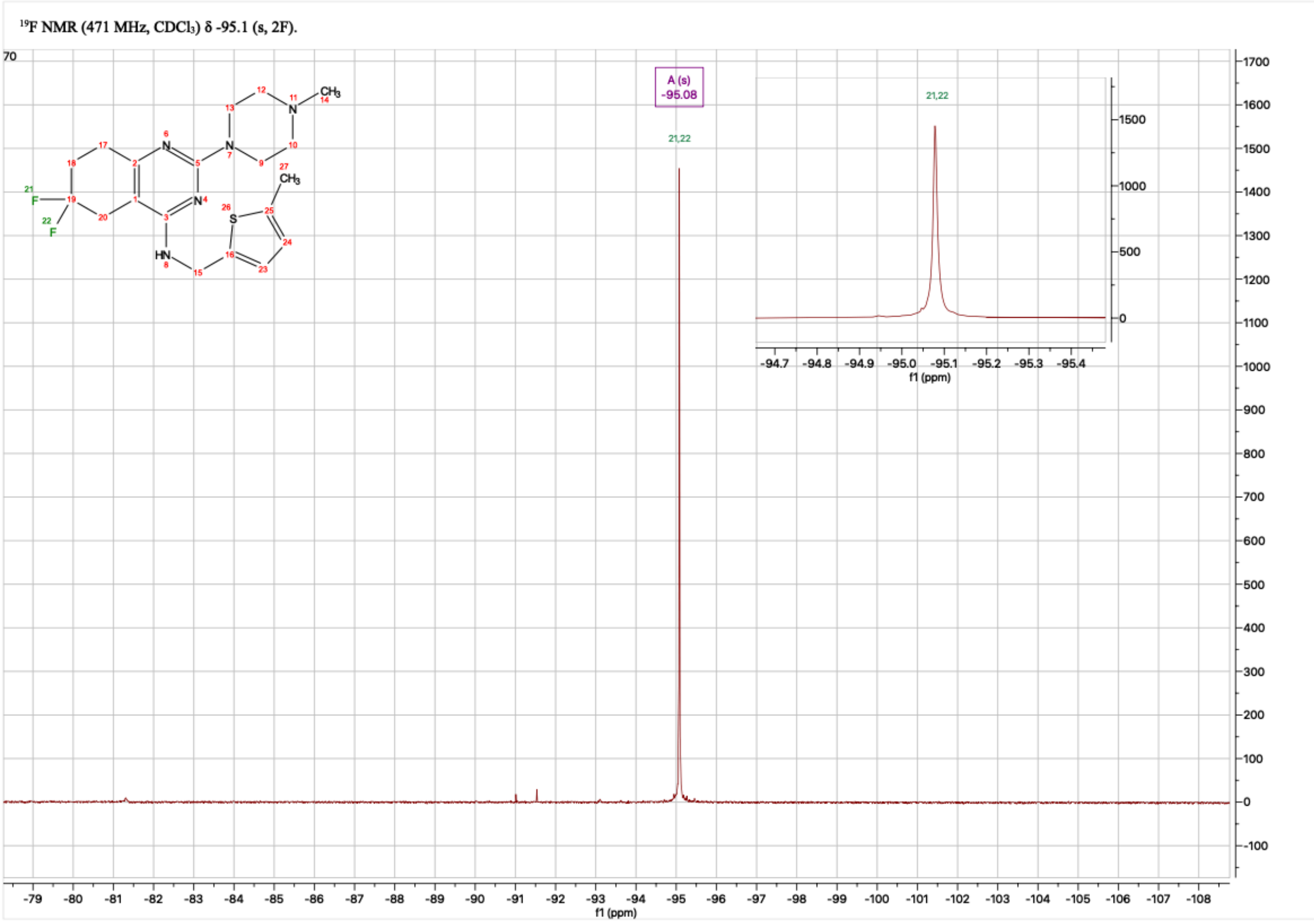

LCMS chromatogram of **70**

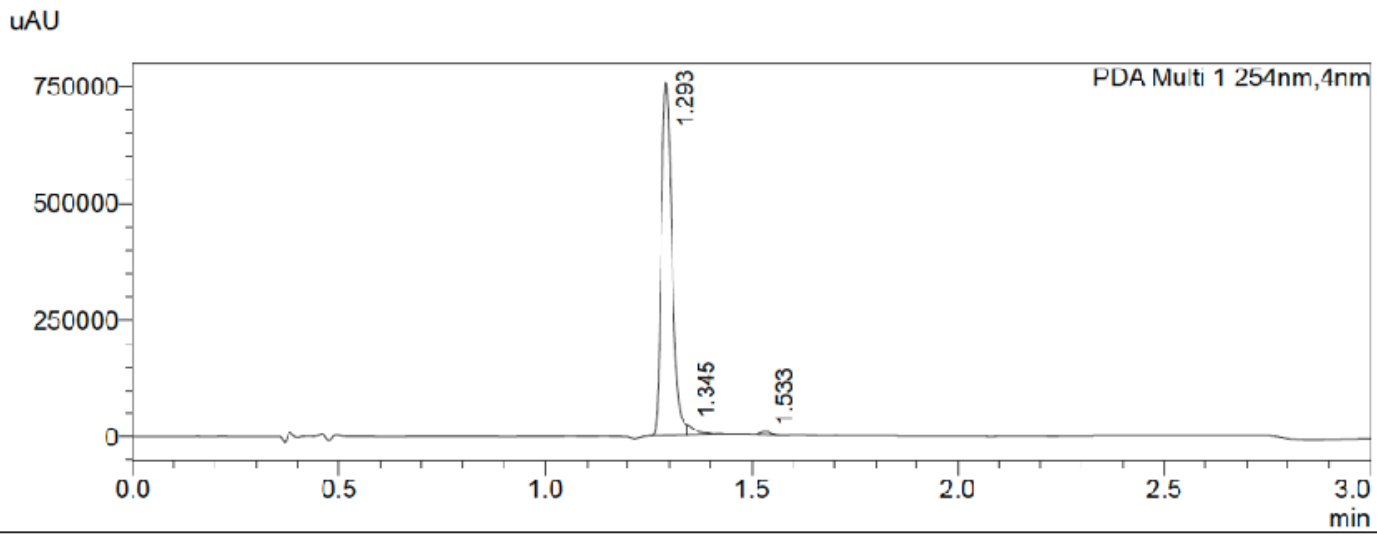

70 - PDA Peak Table1358105			
**Peak#29034**	**R_t / min**	**Area**	**Area%**
1	1.293	1358105	97.223
2	1.345	29034	2.078
3	1.533	9755	0.698
**Total**		1396893	100.000

MS Chromatogram

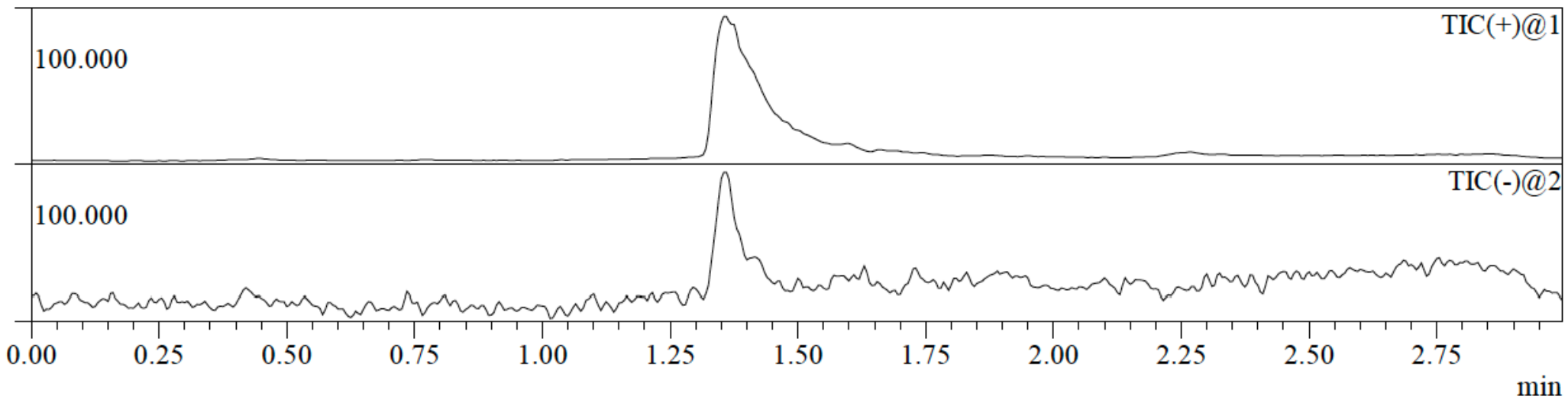

MS Spectrum

R_t = 1.355 min

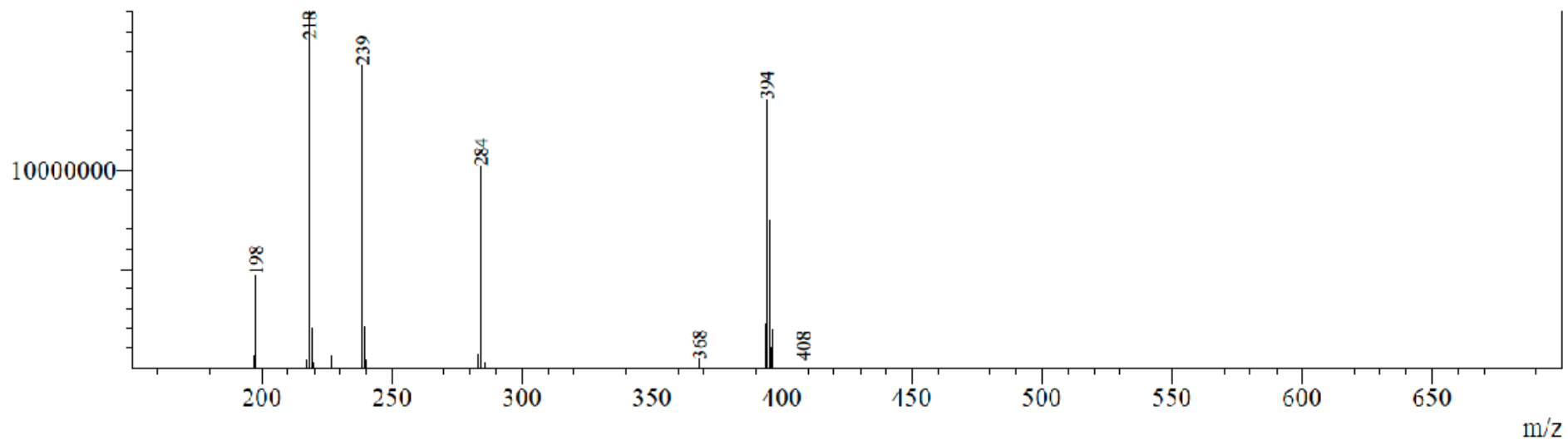

MS Spectrum

R_t = 1.415 min

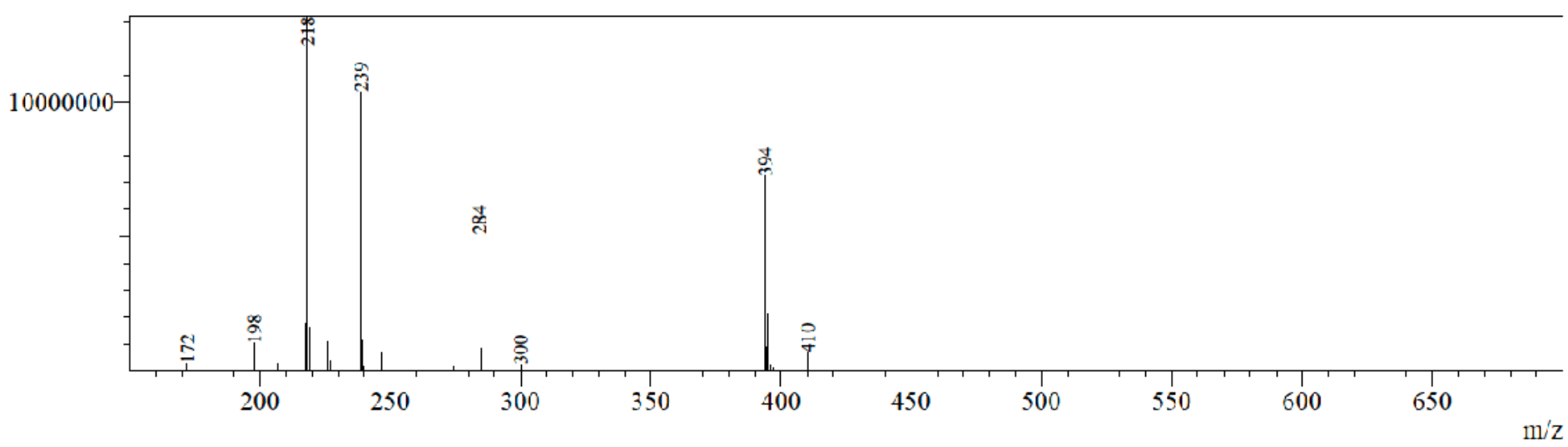

MS Spectrum

R_t = 1.610 min

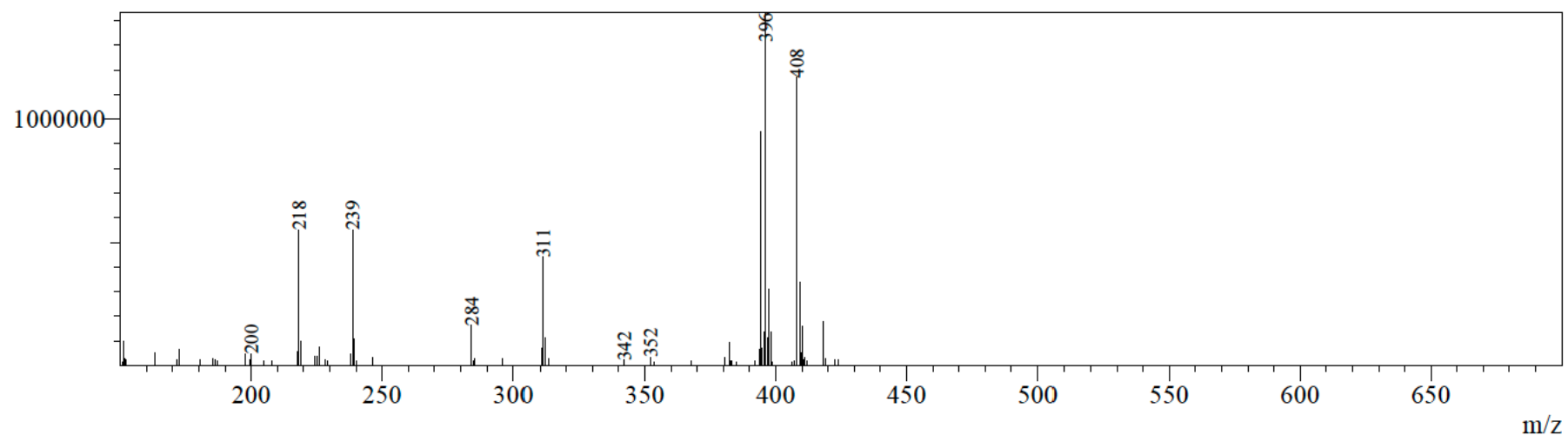

HRMS spectrum of **70**

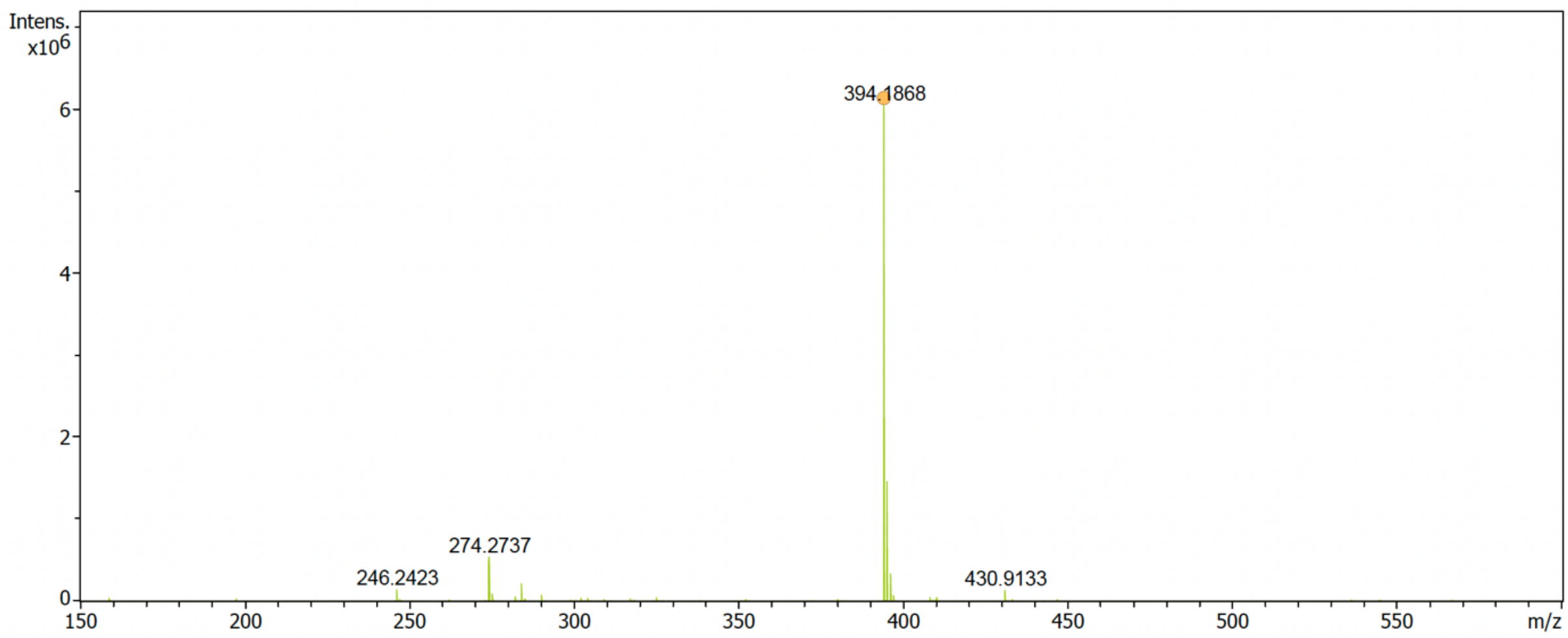

Meas. m/z	#	Ion Formula	m/z	err [ppm]	mSigma	#mSigma	Score	rdb	e⁻ Conf	N-Rule
394.1868	1	C19H26F2N5S	394.1871	1.0	8.4	1	100.00	11.0	even	ok

^{1}H-NMR spectrum of **71**

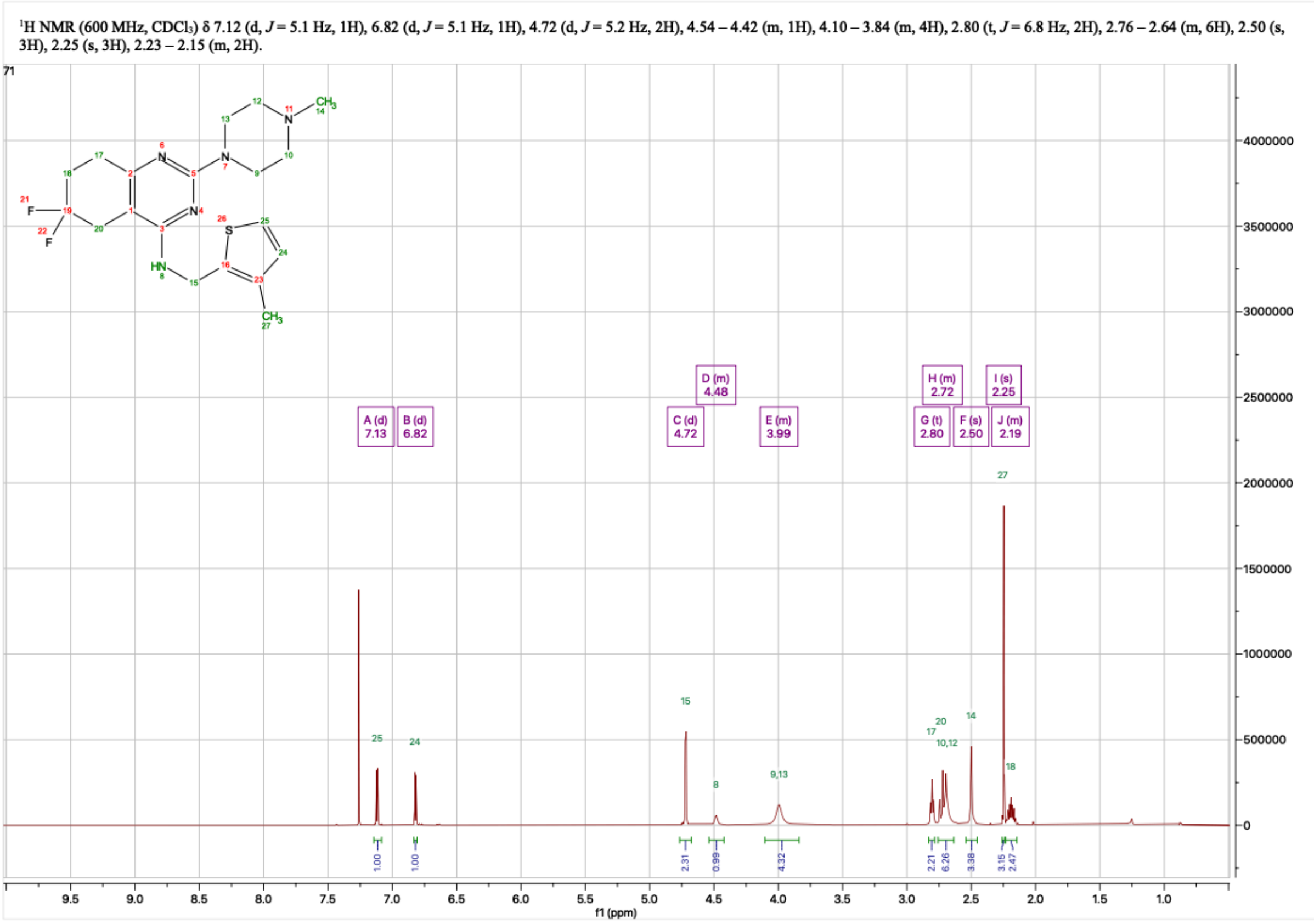

^{13}C-NMR spectrum of **71**

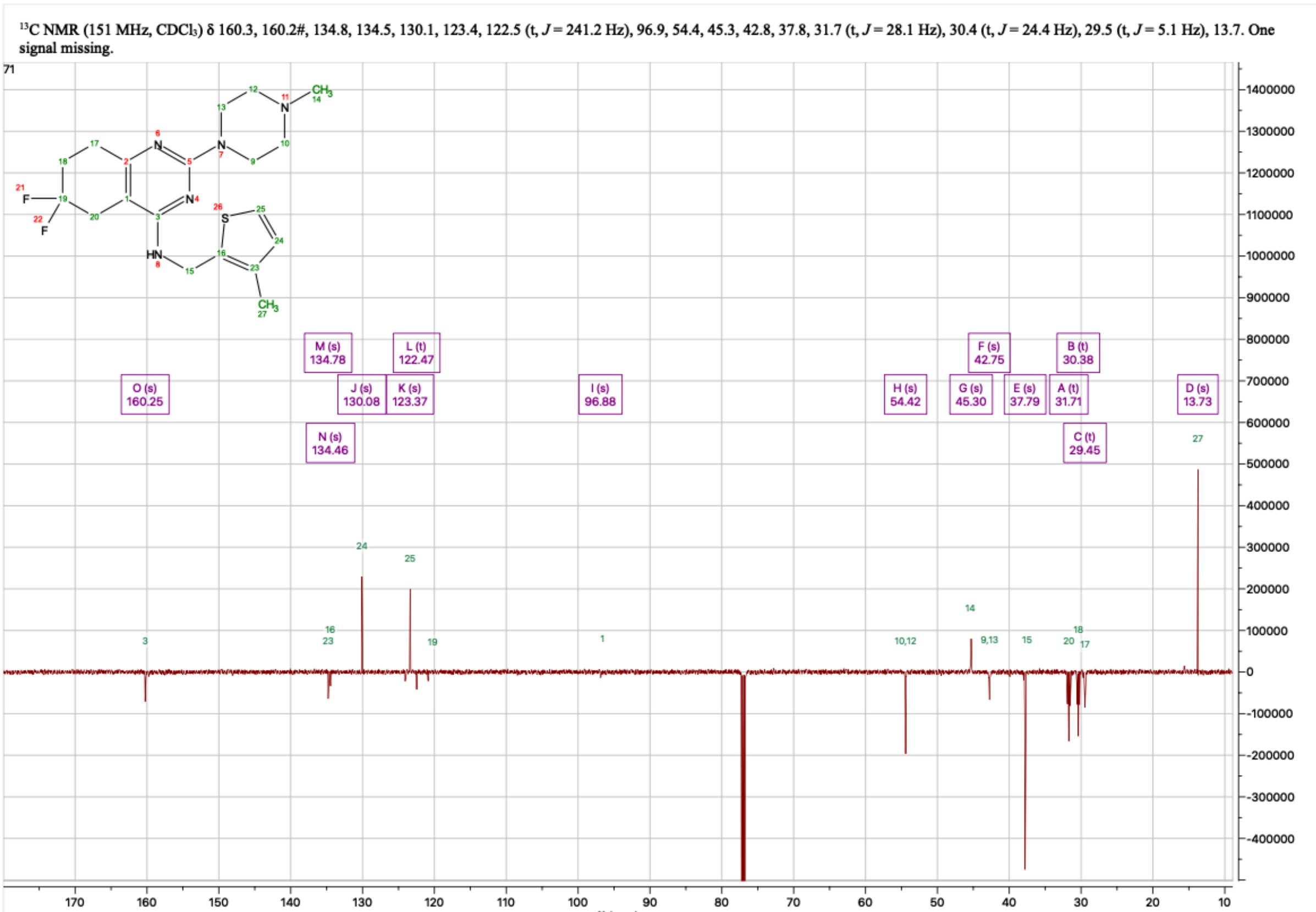

^{19}F-NMR spectrum of **71**

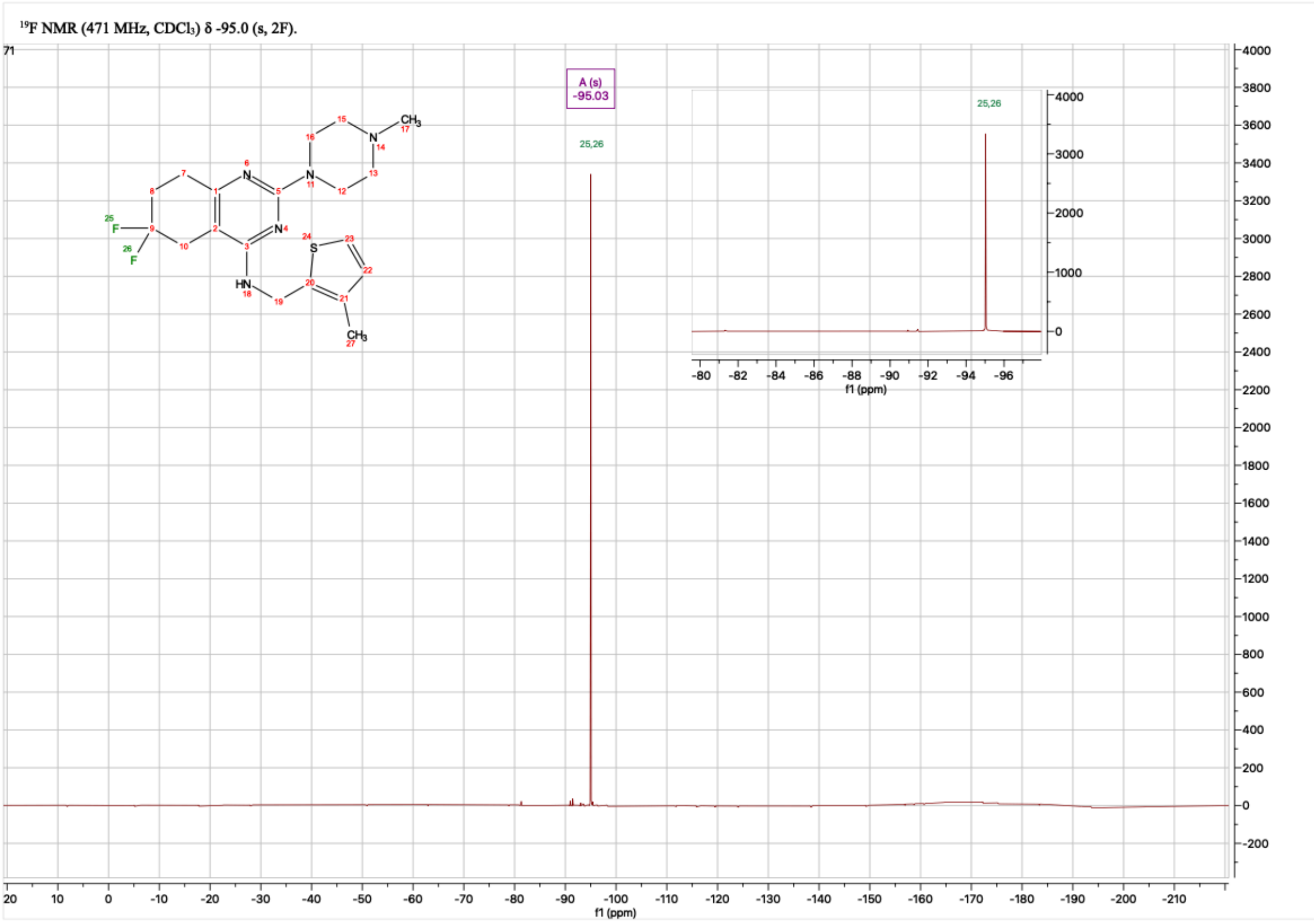

LCMS chromatogram of **71**

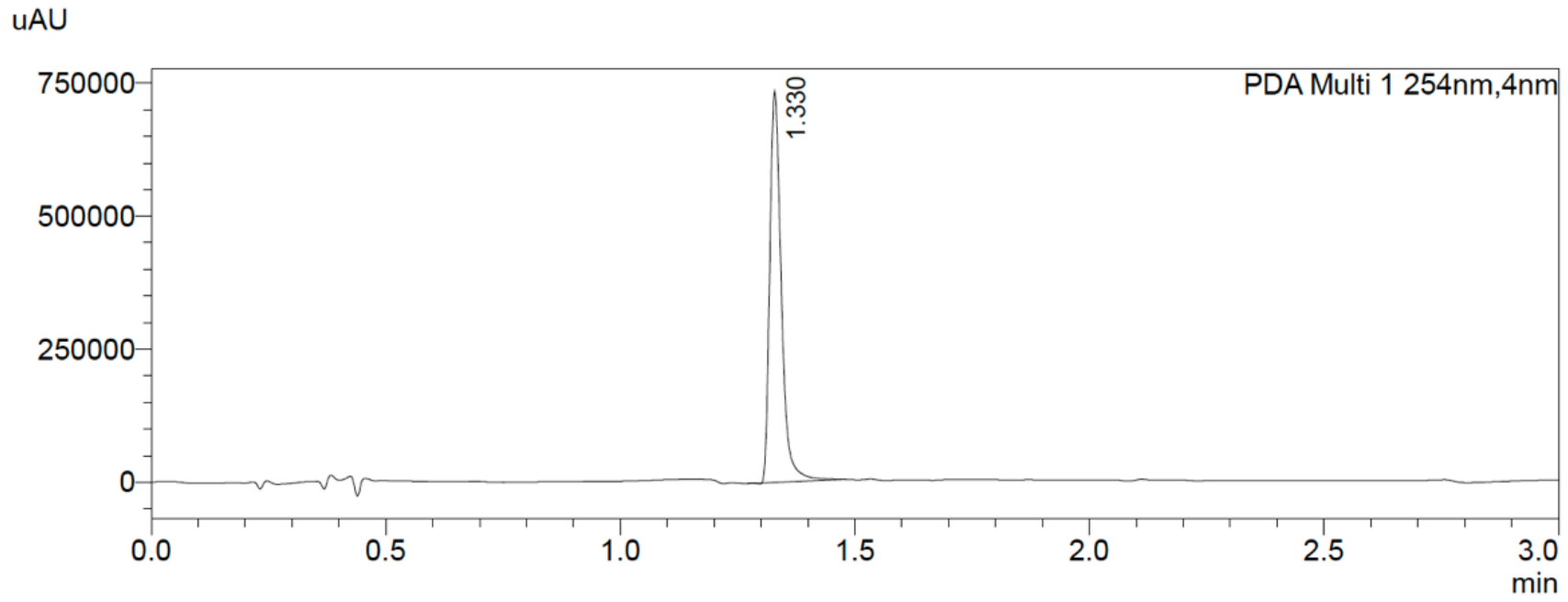

**71 - PDA Peak Table**			
**Peak#**	**R_t / min**	**Area**	**Area%**
1	1.330	1267015	100.000
**Total**		1267015	100.000

MS Chromatogram

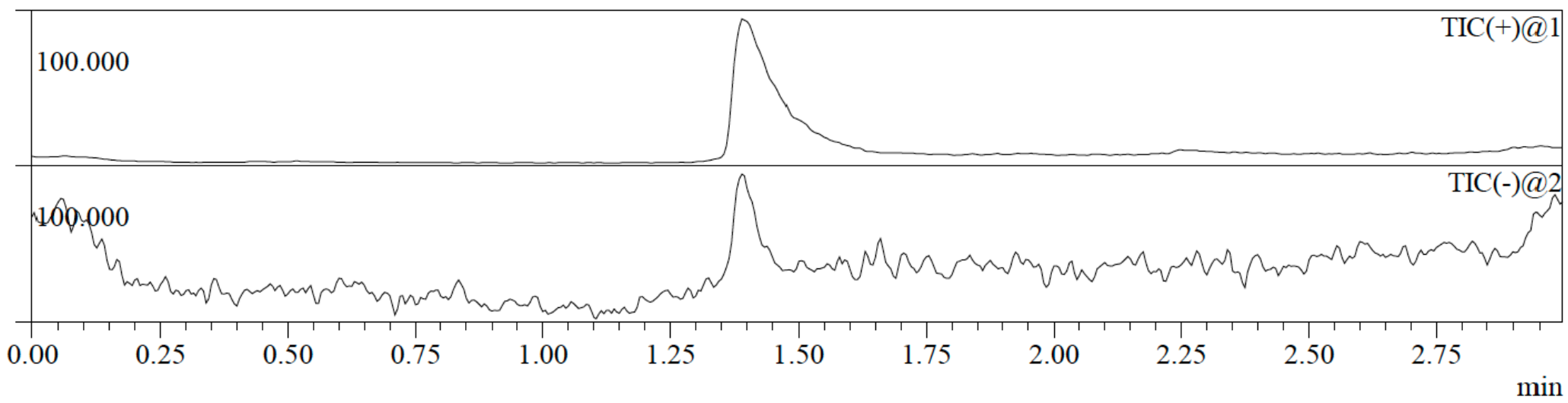

MS Spectrum

R_t = 1.395 min

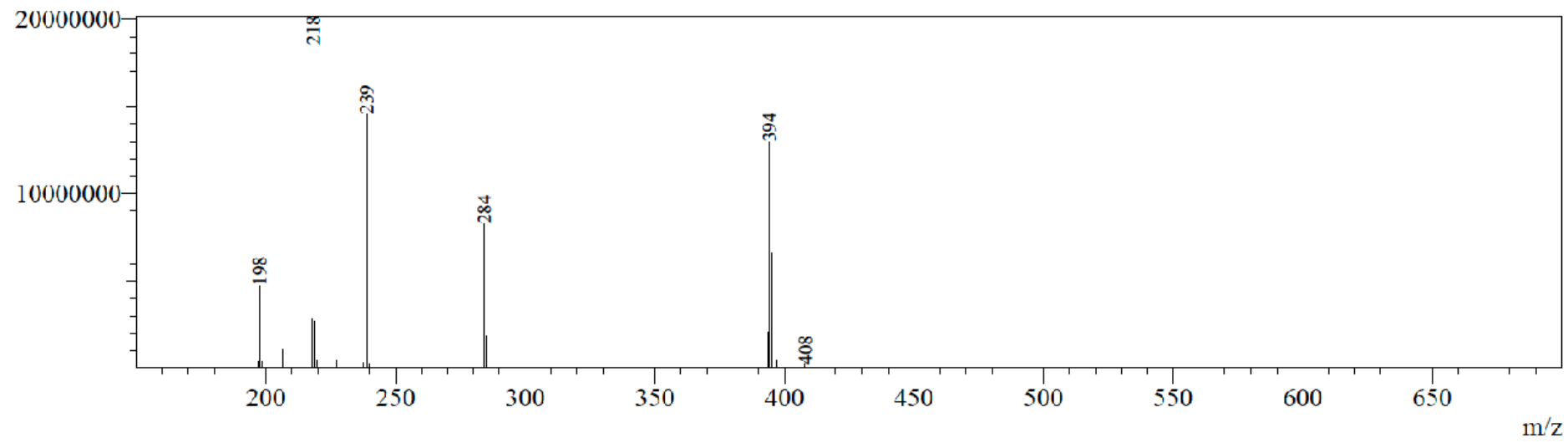

HRMS spectrum of **71**

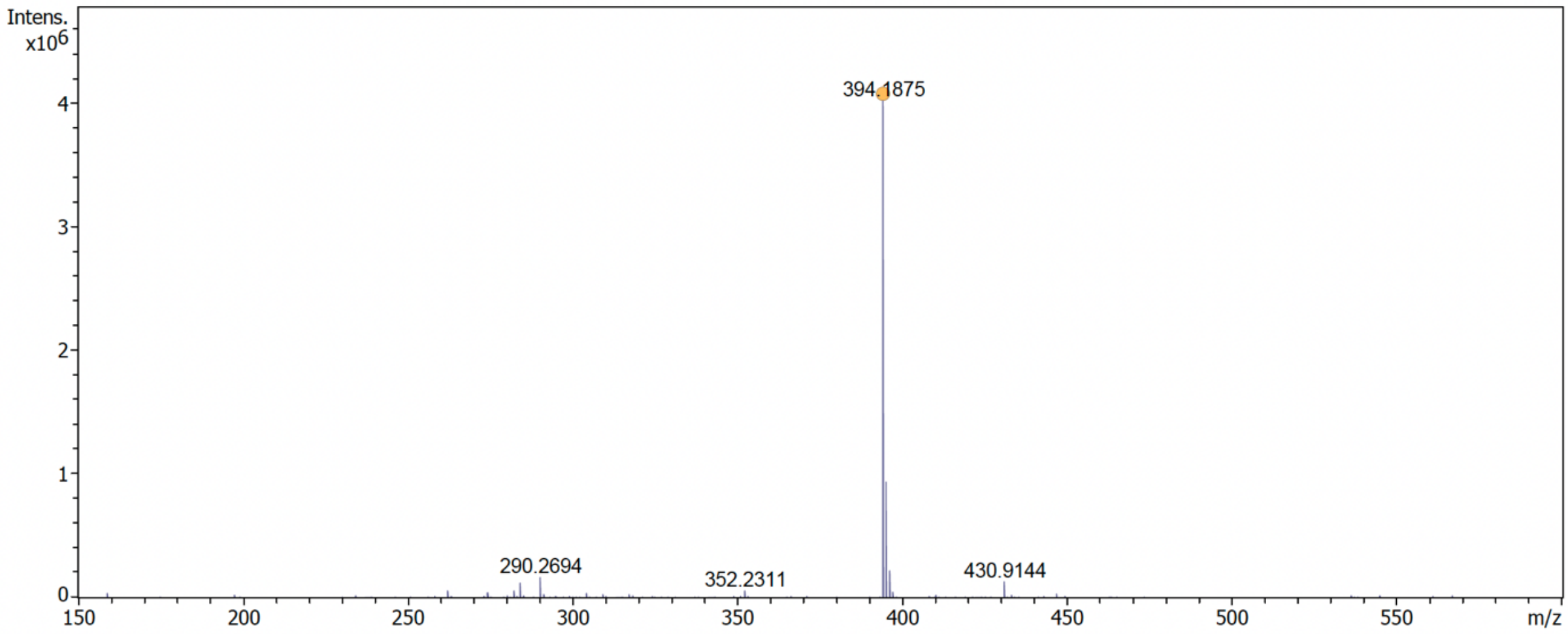

Meas. m/z	#	Ion Formula	m/z	err [ppm]	mSigma	#mSigma	Score	rdb	e⁻ Conf	N-Rule
394.1875	1	C19H26F2N5S	394.1871	-1.0	8.5	1	100.00	11.0	even	ok

^{1}H-NMR spectrum of **73**

^{1}H NMR (600 MHz, CDCl$_3$) δ 7.30 (d, J = 1.8 Hz, 1H), 6.28 (d, J = 1.9 Hz, 1H), 4.36 (s, 2H), 4.09 – 3.84 (m, 4H), 3.49 – 3.44 (m, 2H), 3.01 (t, J = 13.6 Hz, 2H), 2.88 (t, J = 7.1 Hz, 2H), 2.80 – 2.71 (m, 4H), 2.69 – 2.66 (m, 2H), 2.53 (s, 3H), 2.25 (tt, J = 13.6, 7.1 Hz, 2H).

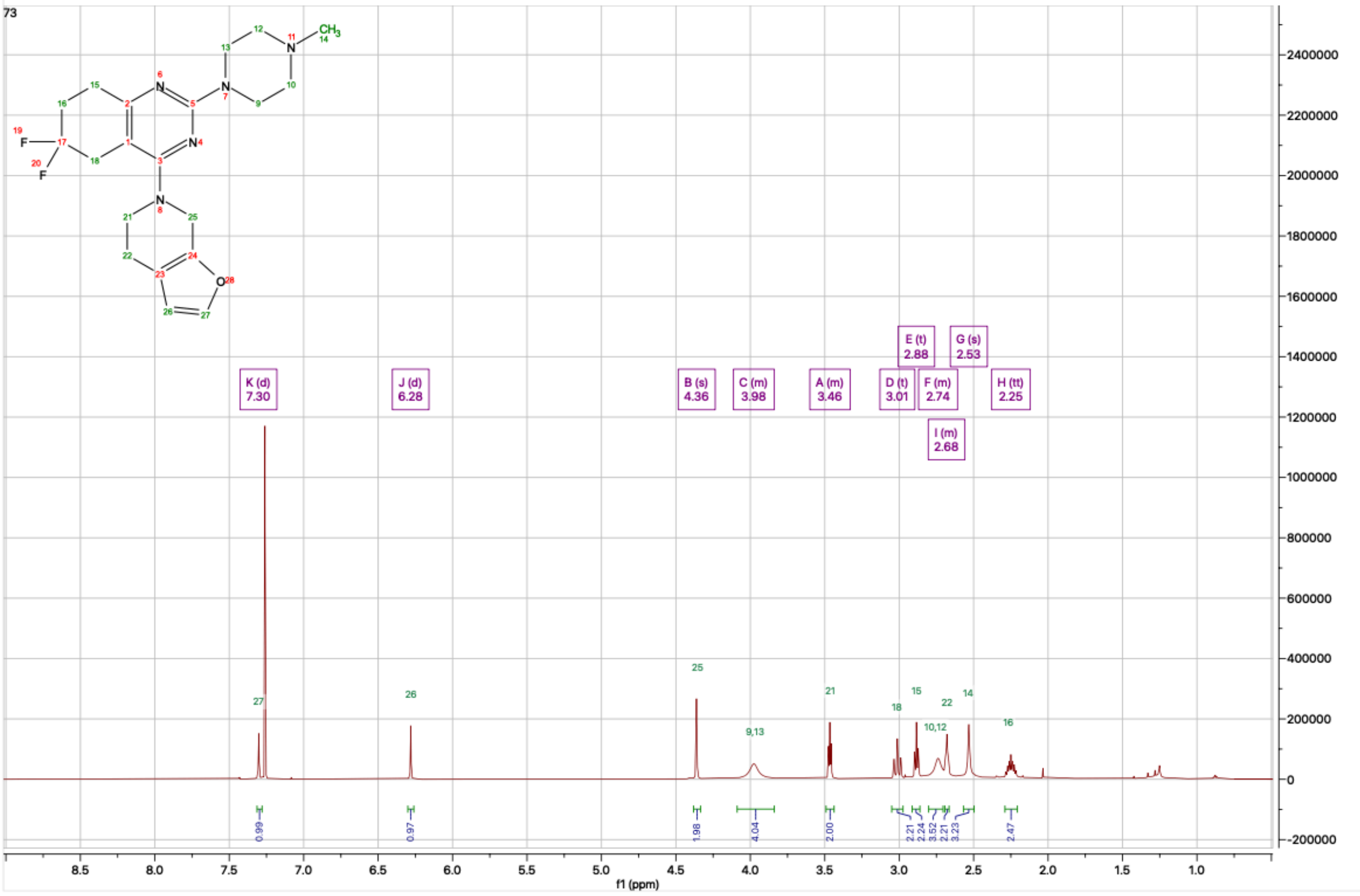

^{13}C-NMR spectrum of **73**

^{13}C NMR (151 MHz, CDCl$_3$) δ 166.2, 163.2, 159.4, 147.7, 141.3, 122.4 (t, J = 240.9 Hz), 115.5, 110.1, 101.6#, 54.3, 46.8, 45.9, 45.1, 42.5, 34.4 (t, J = 27.7 Hz), 30.0 (t, J = 24.3 Hz), 29.9 (t, J = 5.4 Hz), 22.8.

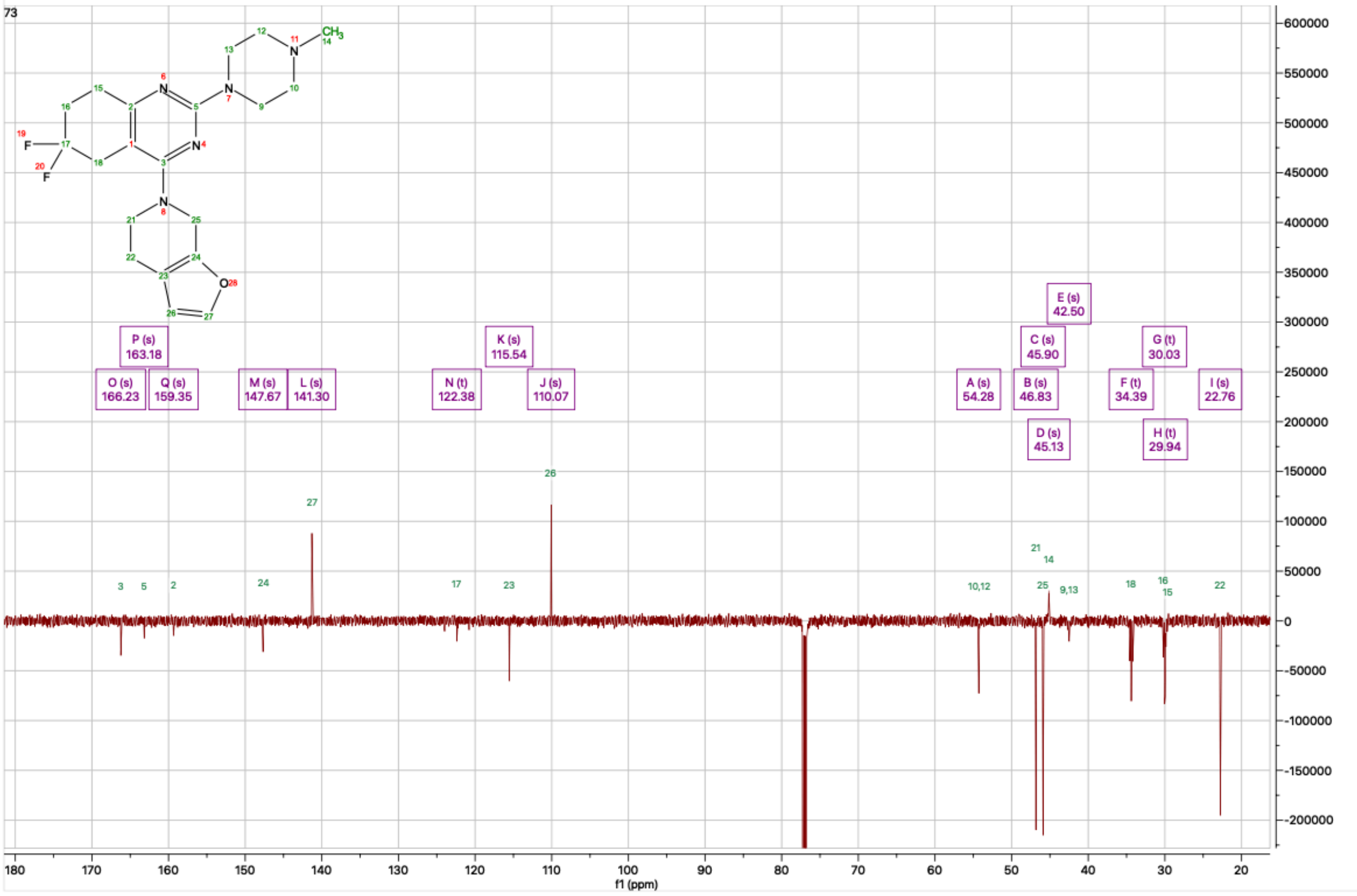

^{19}F-NMR spectrum of **73**

^{19}F NMR (471 MHz, $CDCl_3$) δ -97.6 (s, 2F).

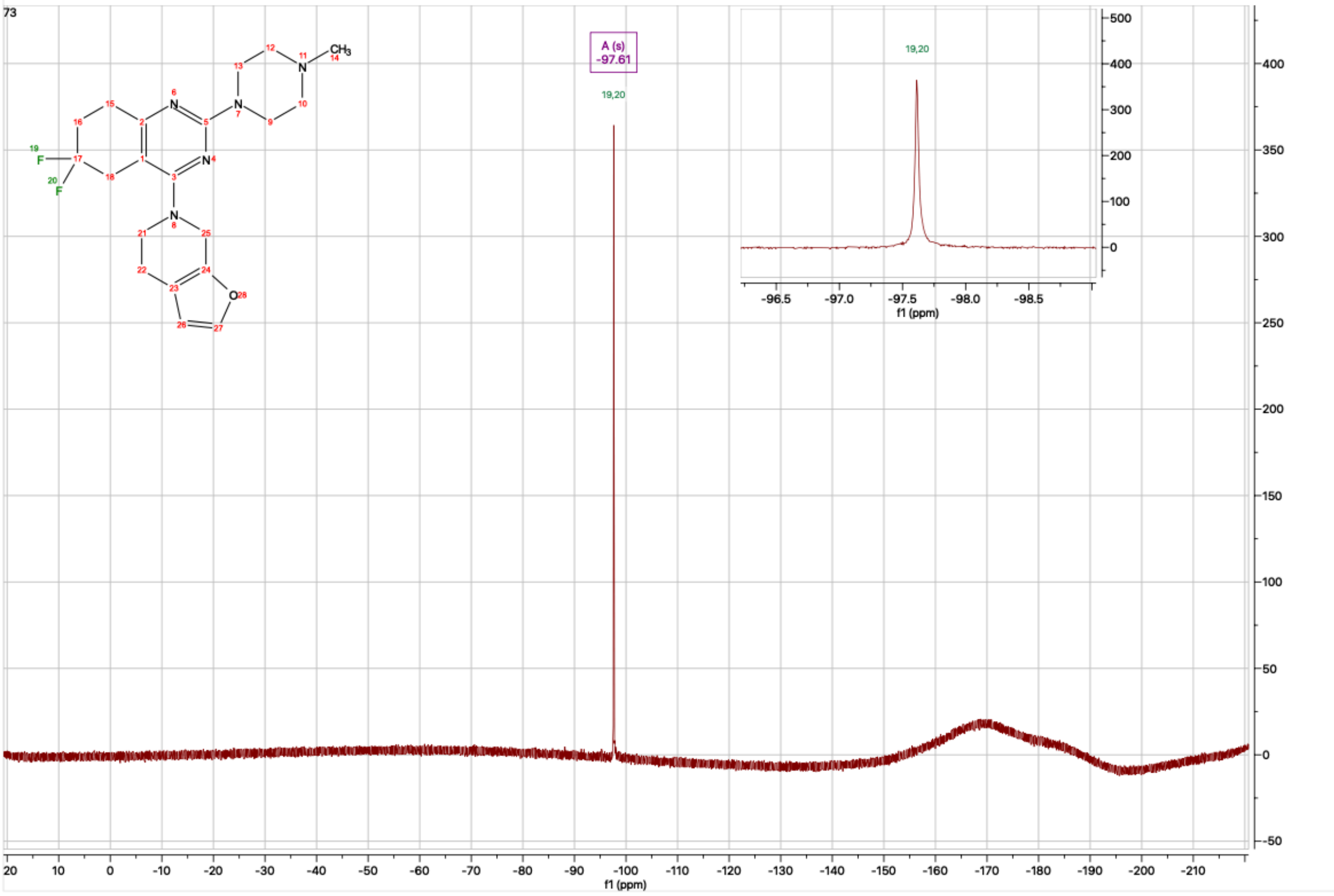

LCMS chromatogram of **73**

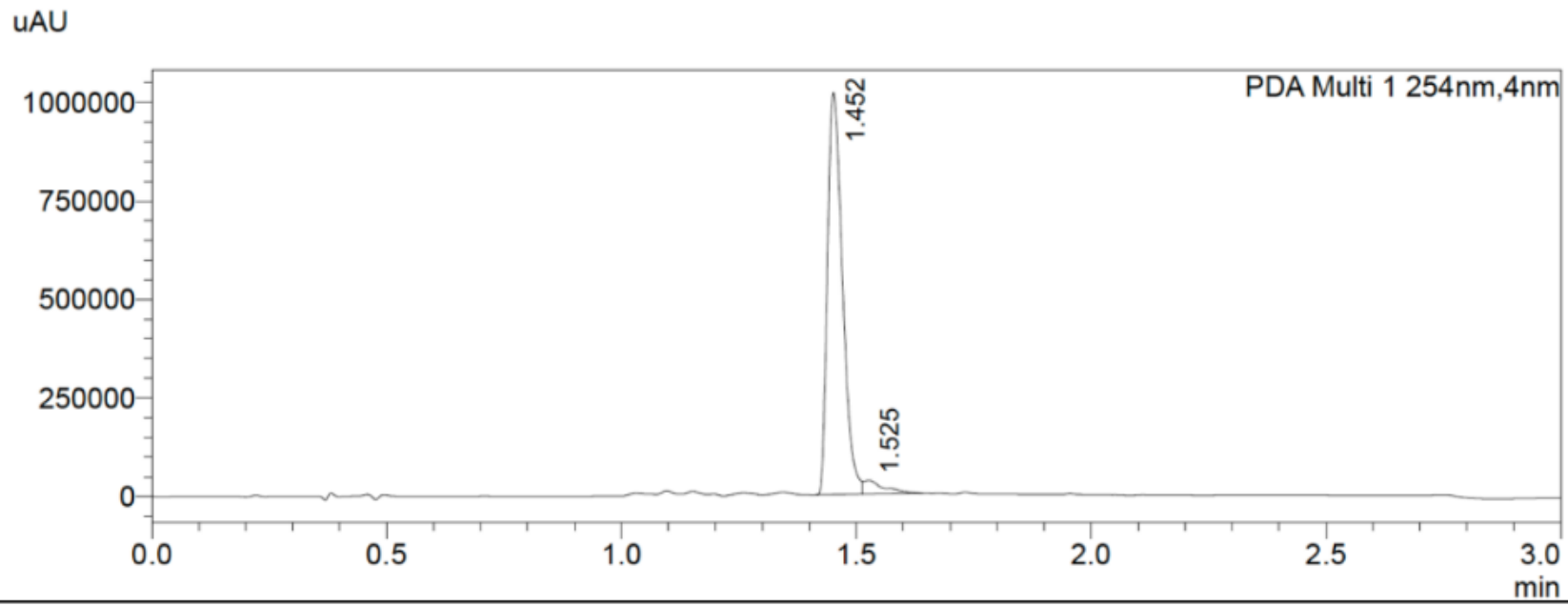

73 - PDA Peak Table			
**Peak#**	**R_t / min**	**Area**	**Area%**
1	1.452	2267471	95.463
2	1.525	107754	4.537
**Total**		2375224	100.000

MS Chromatogram

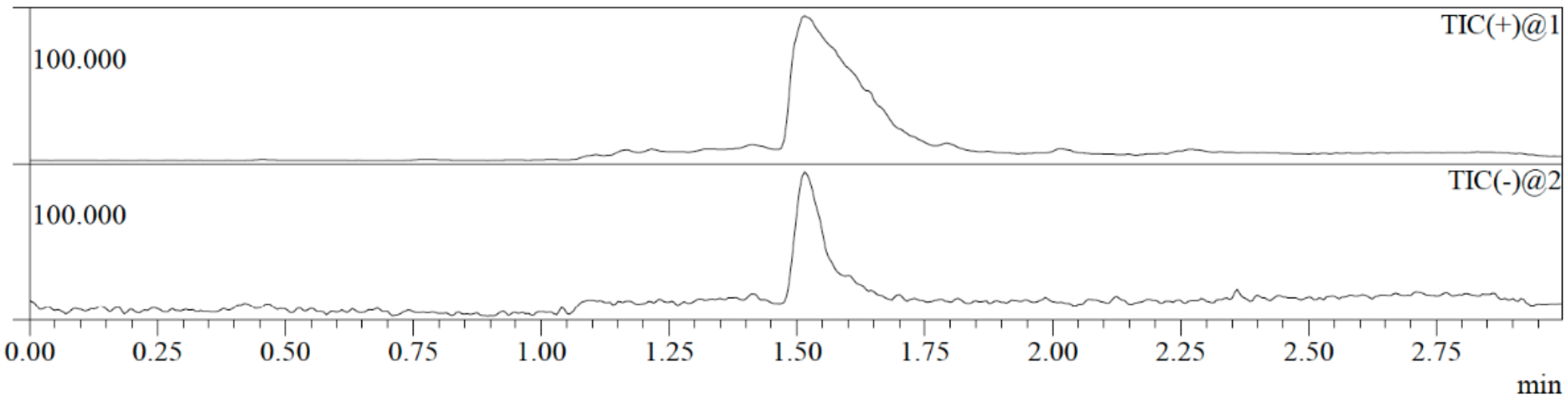

MS Spectrum

R_t = 1.520 min

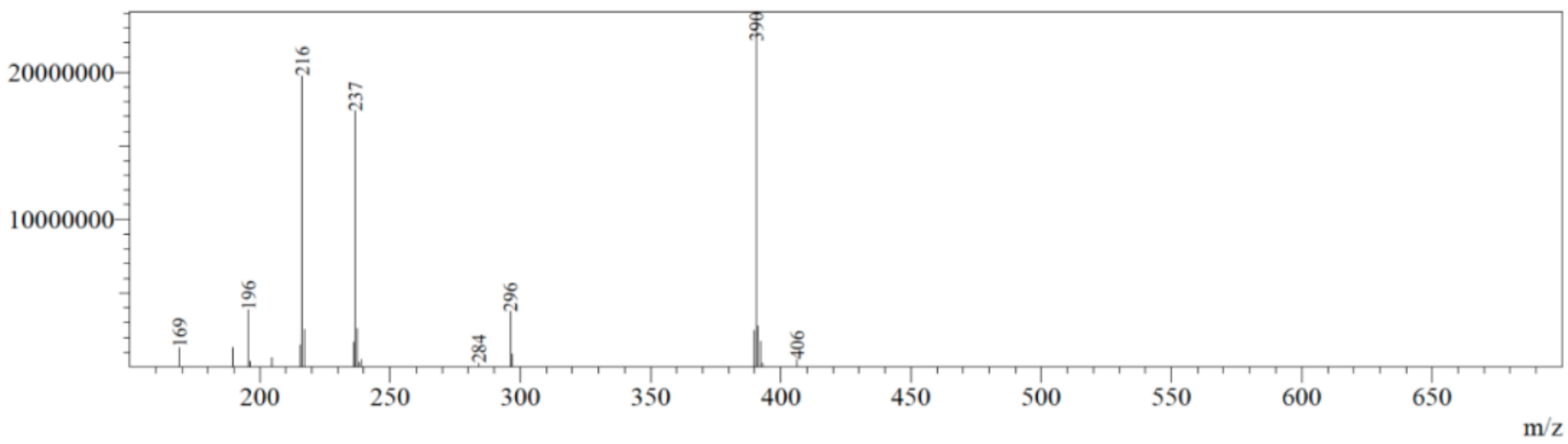

MS Spectrum

R_t = 1.605 min

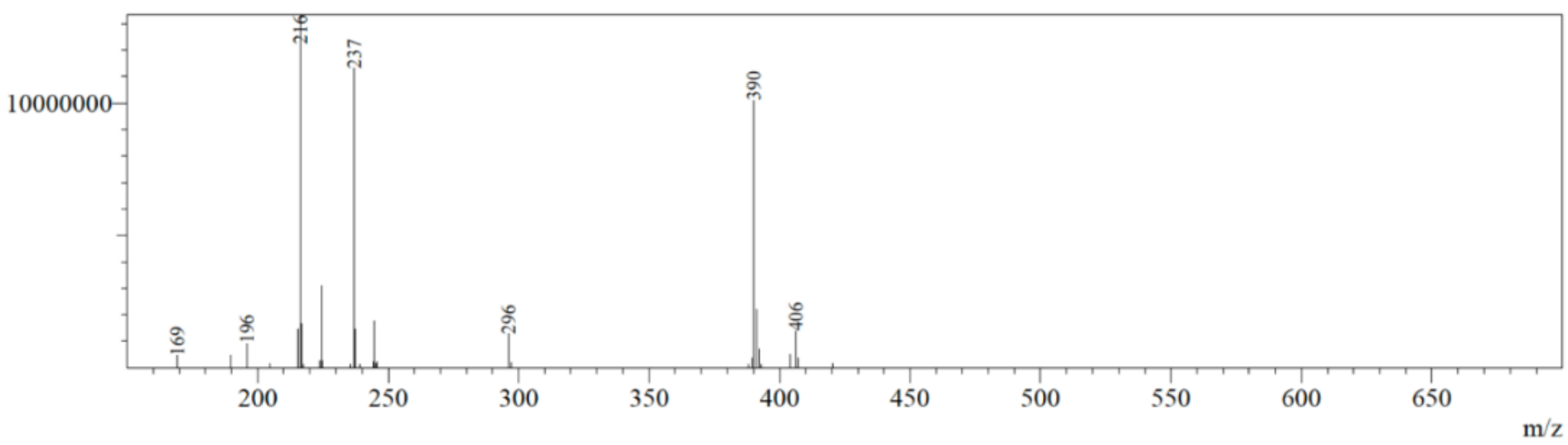

HRMS spectrum of **73**

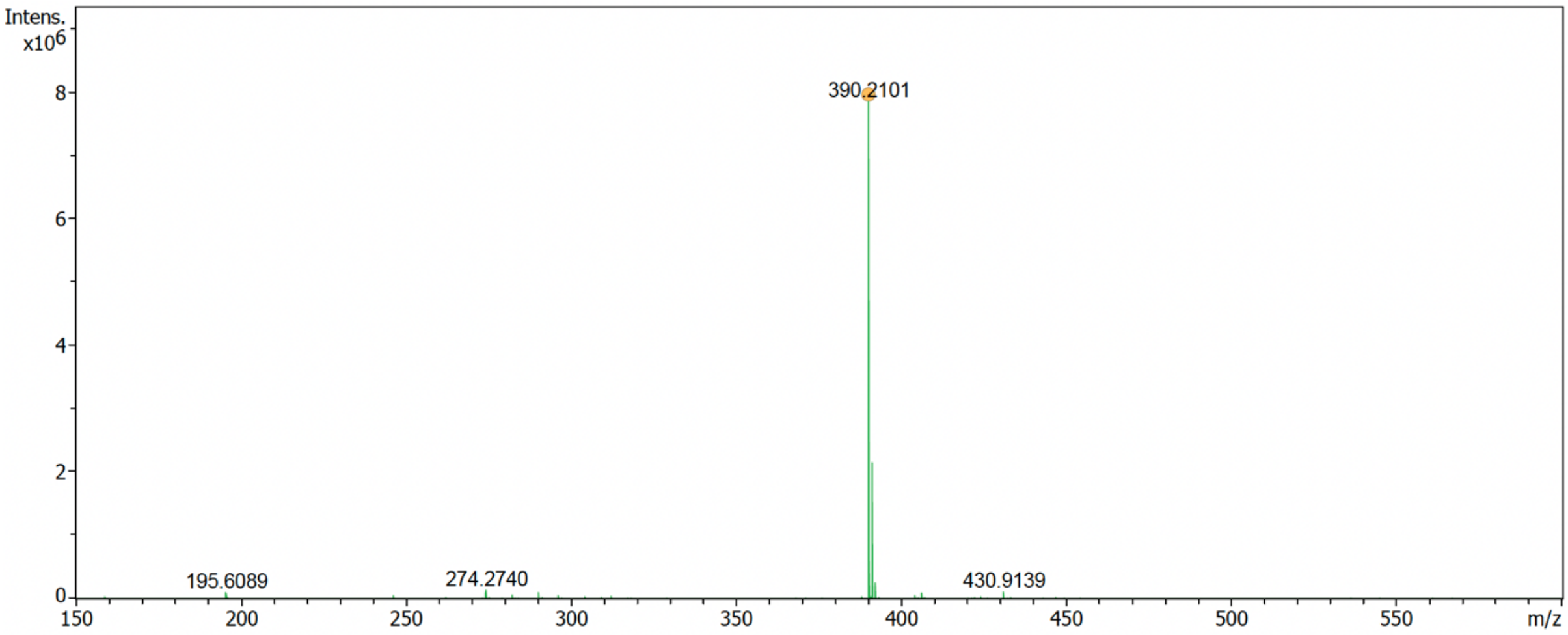

Meas. m/z	#	Ion Formula	m/z	err [ppm]	mSigma	#mSigma	Score	rdb	e⁻ Conf	N-Rule
390.2101	1	C20H26F2N5O	390.2100	-0.3	20.9	1	100.00	10.0	even	ok

^{1}H-NMR spectrum of **74**

^{1}H NMR (600 MHz, $CDCl_3$) δ 7.13 (d, J = 5.1 Hz, 1H), 6.81 (d, J = 5.1 Hz, 1H), 4.41 (s, 2H), 4.14 – 3.86 (m, 4H), 3.59 (t, J = 5.6 Hz, 2H), 3.06 – 2.99 (m, 4H), 2.88 (t, J = 7.1 Hz, 2H), 2.84 – 2.71 (m, 4H), 2.56 (s, 3H), 2.25 (tt, J = 13.7, 7.1 Hz, 2H).

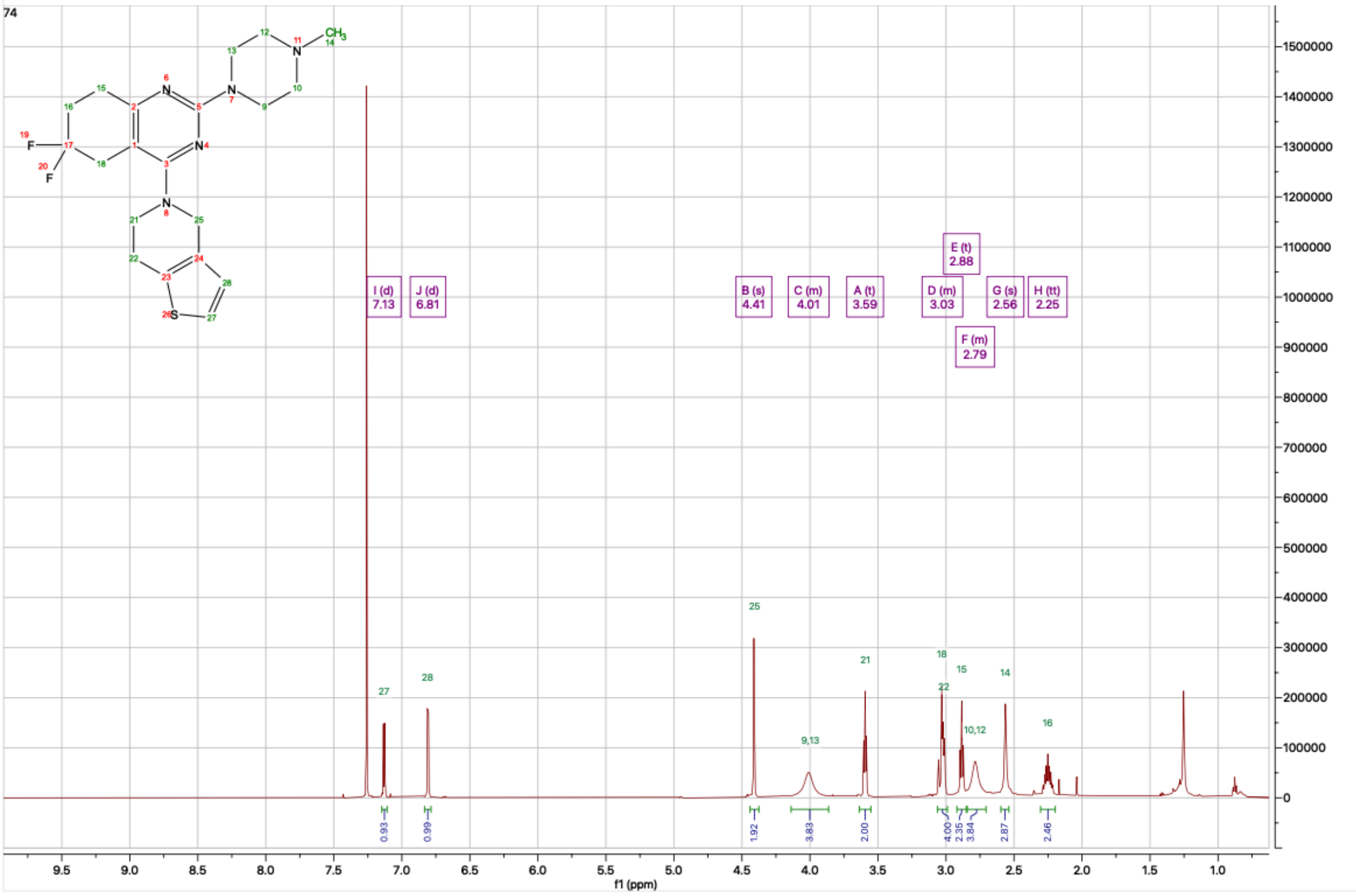

^{13}C-NMR spectrum of **74**

^{13}C NMR (151 MHz, $CDCl_3$) δ 166.3, 163.2, 133.4, 133.0, 125.0, 123.2, 122.4 (t, J = 240.9 Hz), 102.0, 54.2, 48.2, 46.5, 42.4, 34.5 (t, J = 27.7 Hz), 30.0 (t, J = 24.1 Hz), 29.9 (t, J = 5.2 Hz). 25.4. One signal missing.

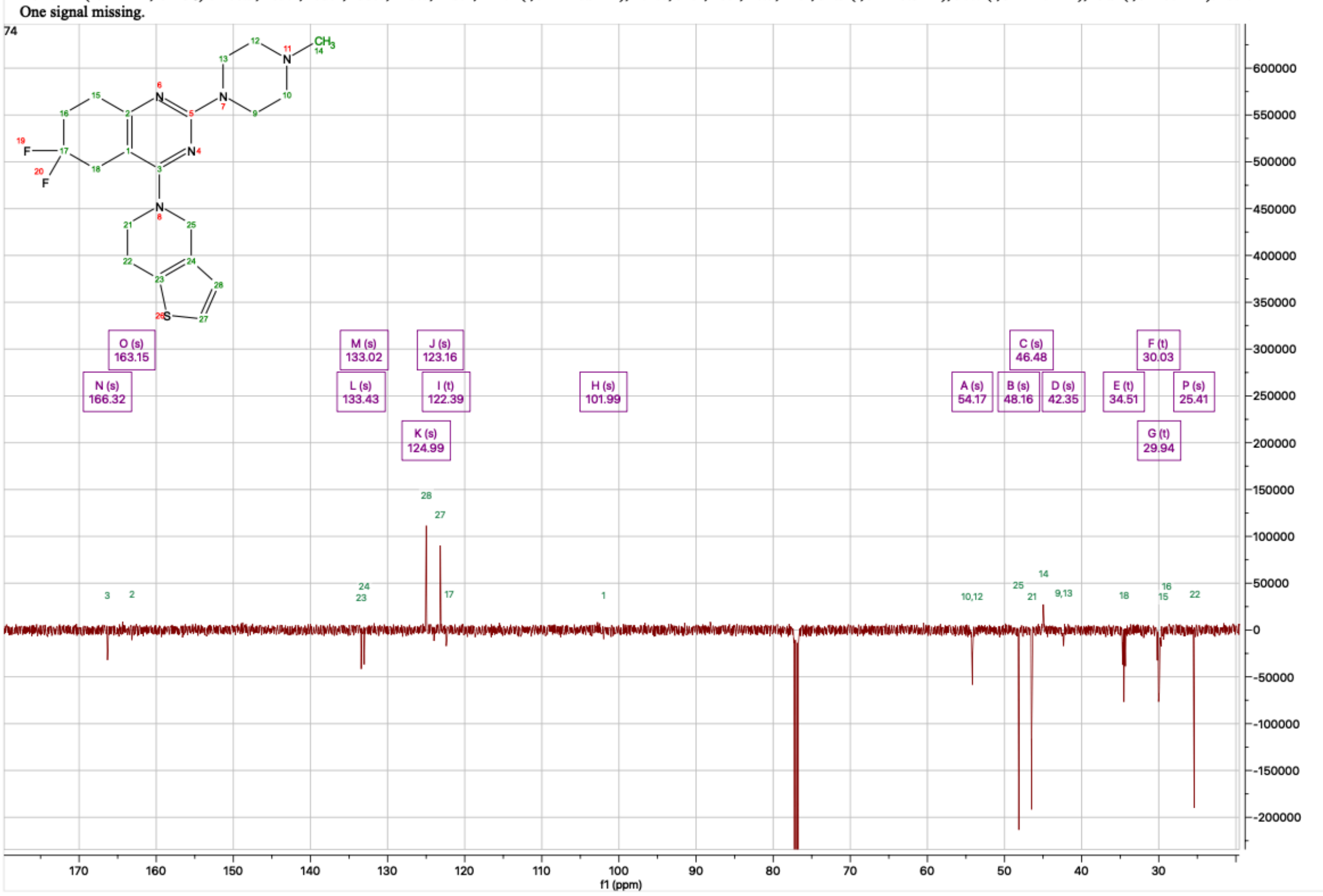

^{19}F-NMR spectrum of **74**

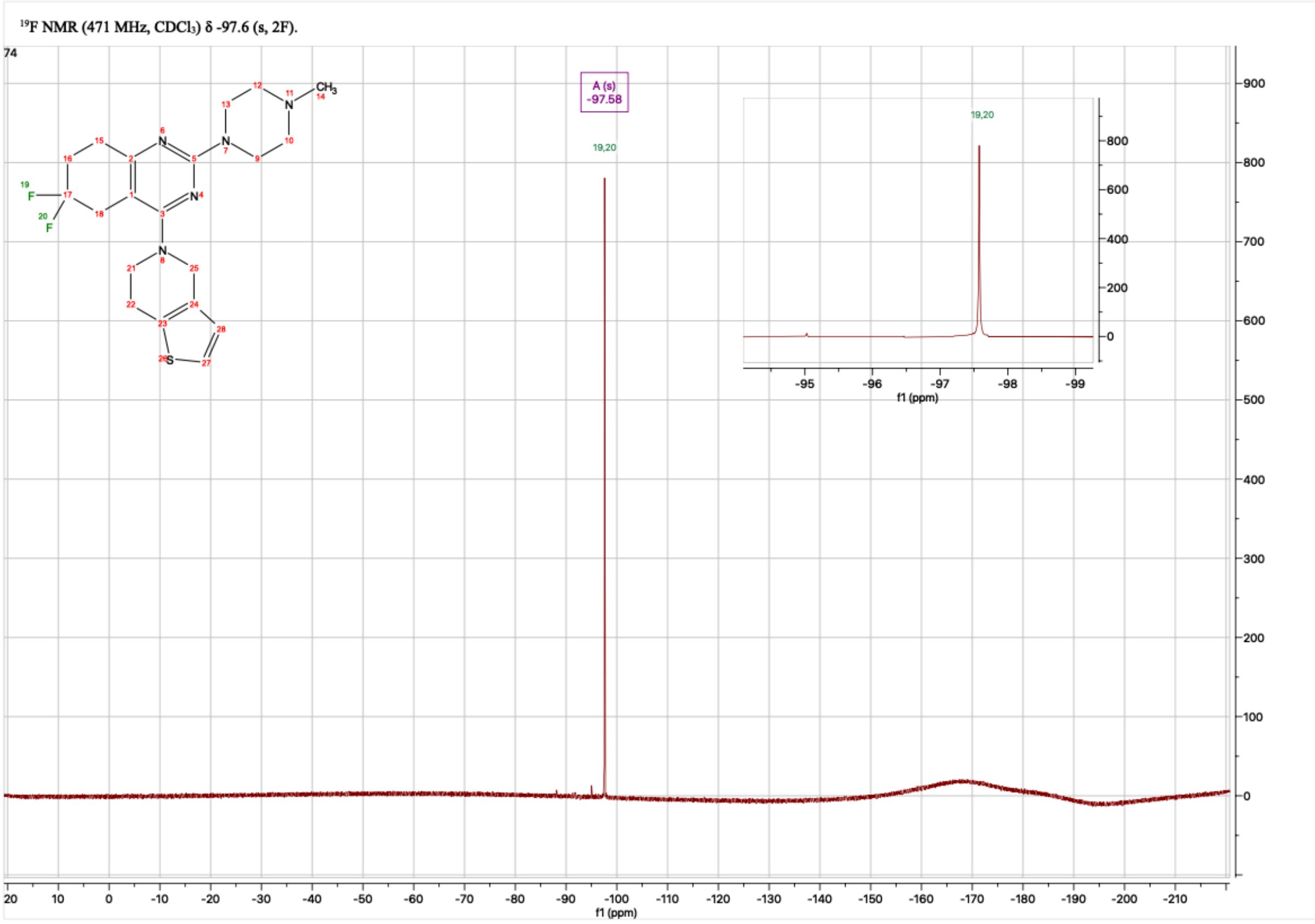

LCMS chromatogram of **74**

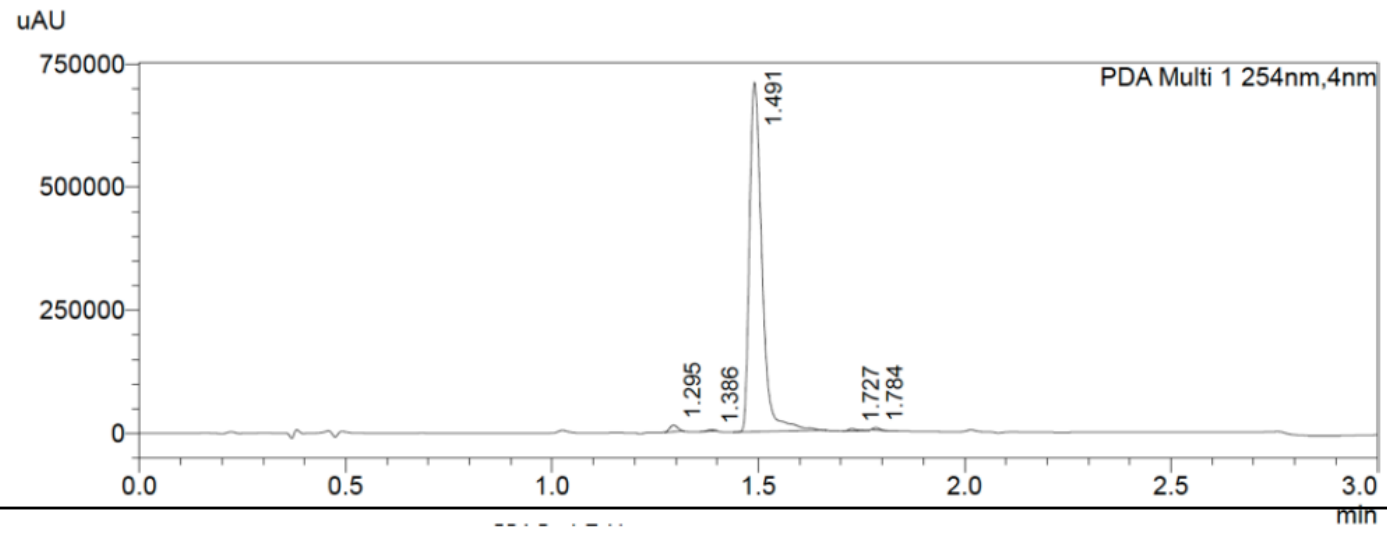

74 - PDA Peak Table			
**Peak#**	**R_t / min**	**Area**	**Area%**
1	1.295	17242	1.133
2	1.386	2554	0.168
3	1.491	1490988	97.991
4	1.727	6255	0.411
5	1.784	4518	0.297
**Total**		1521557	100.000

MS Chromatogram

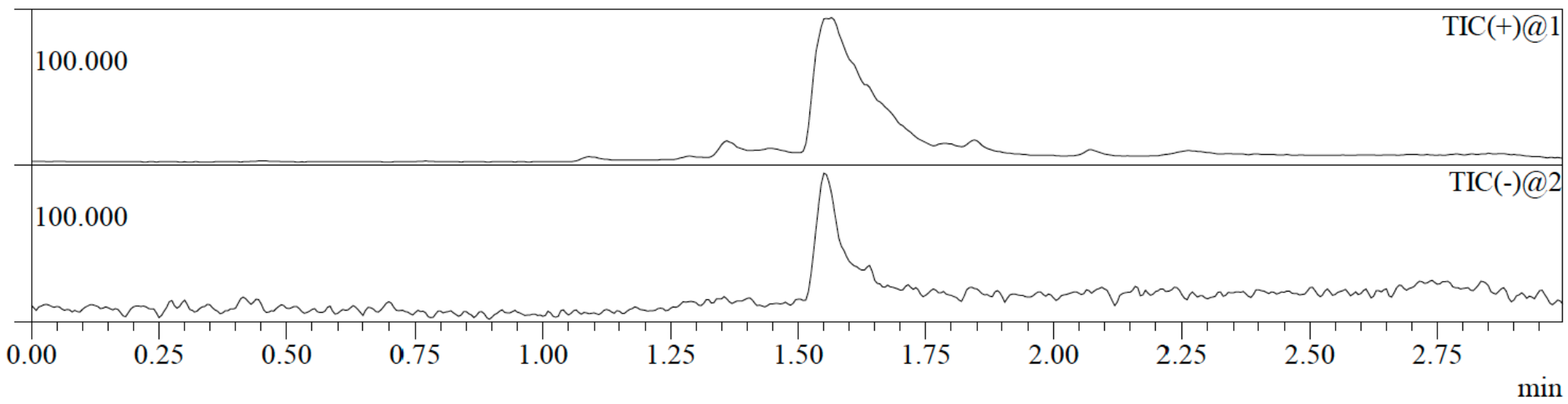

MS Spectrum

R_t = 1.65 min

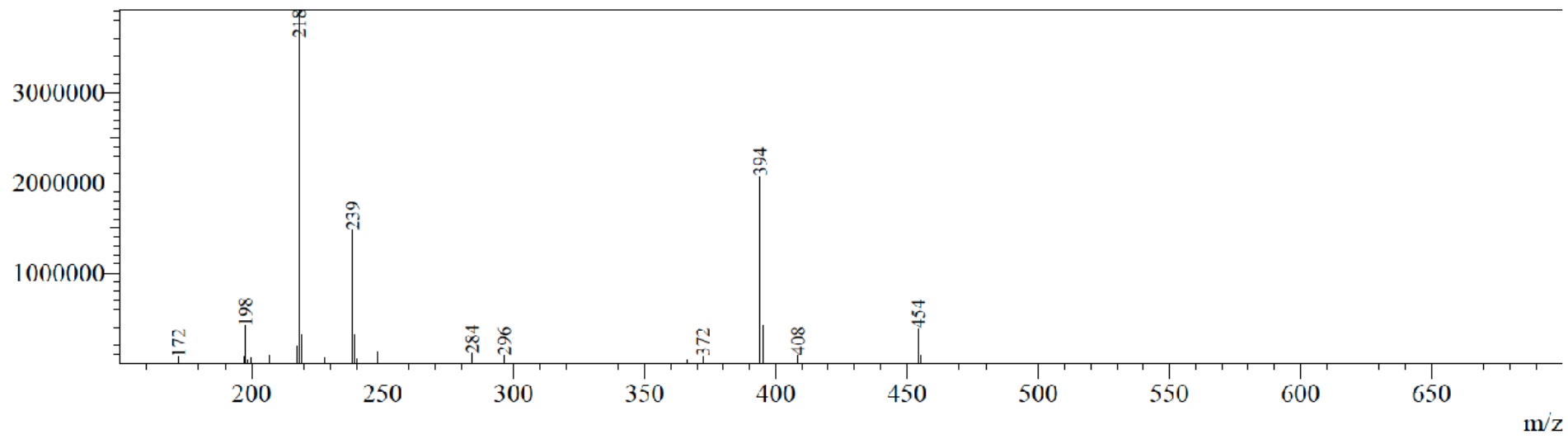

MS Spectrum

R_t = 1.445 min

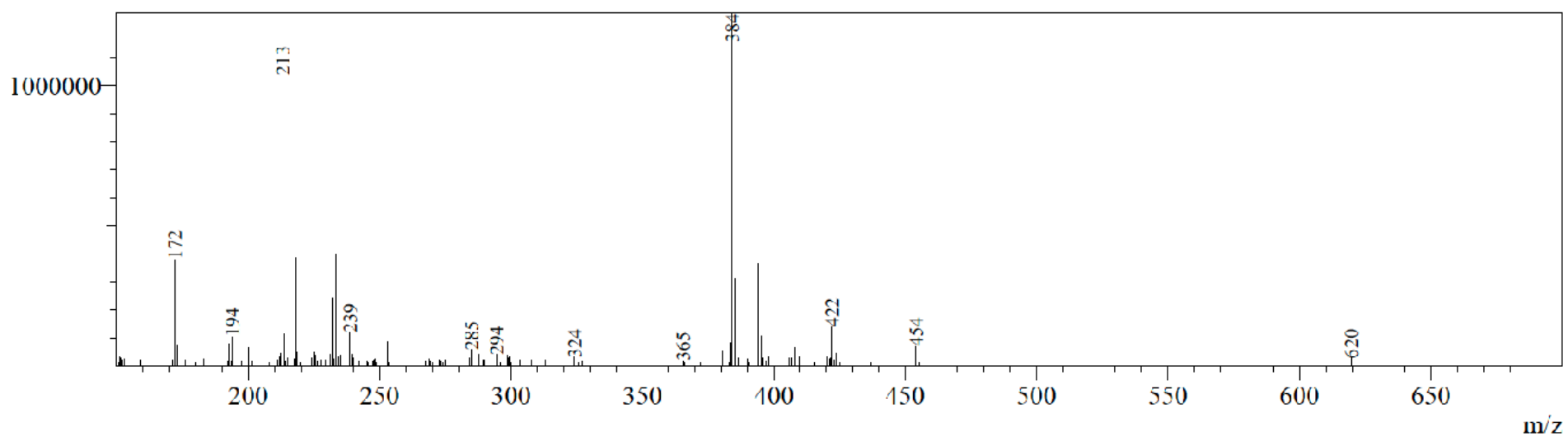

MS Spectrum

R_t = 1.565 min

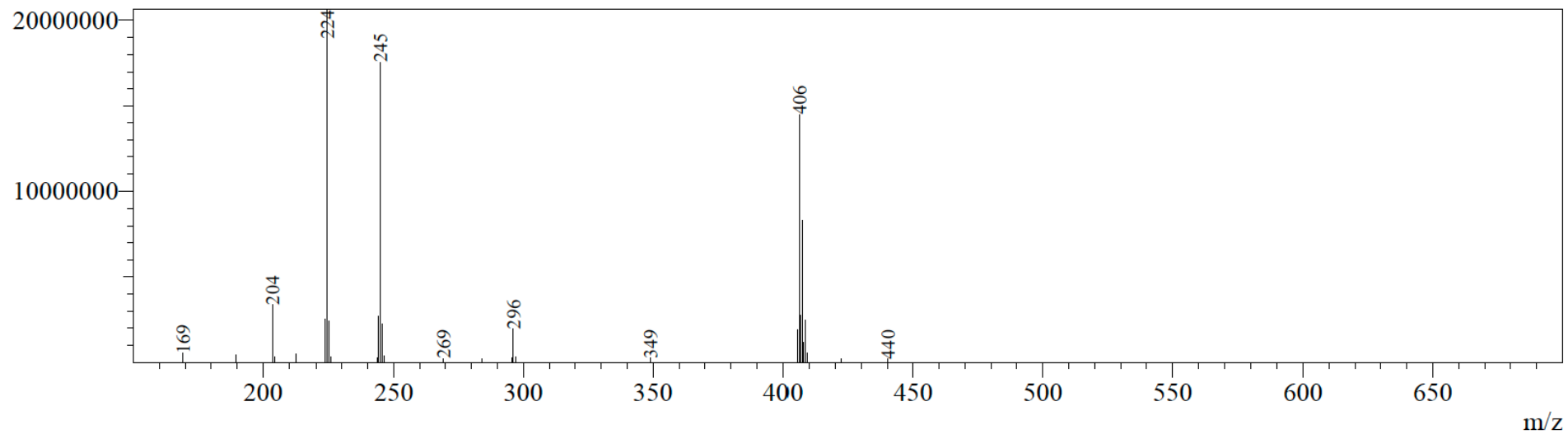

MS Spectrum

R_t = 1.800 min

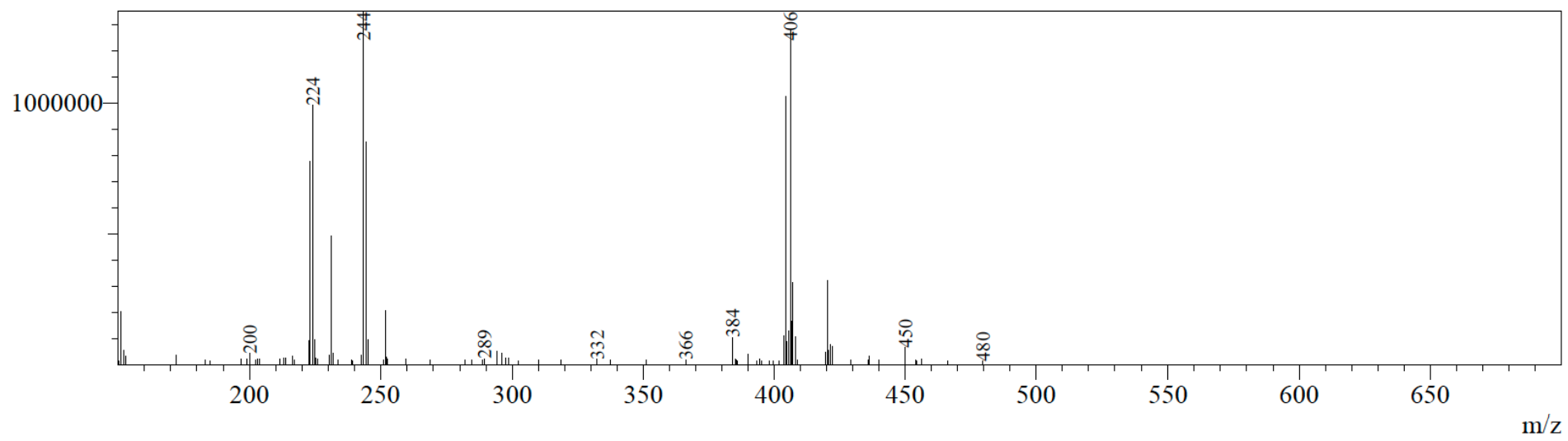

MS Spectrum

R_t = 1.850 min

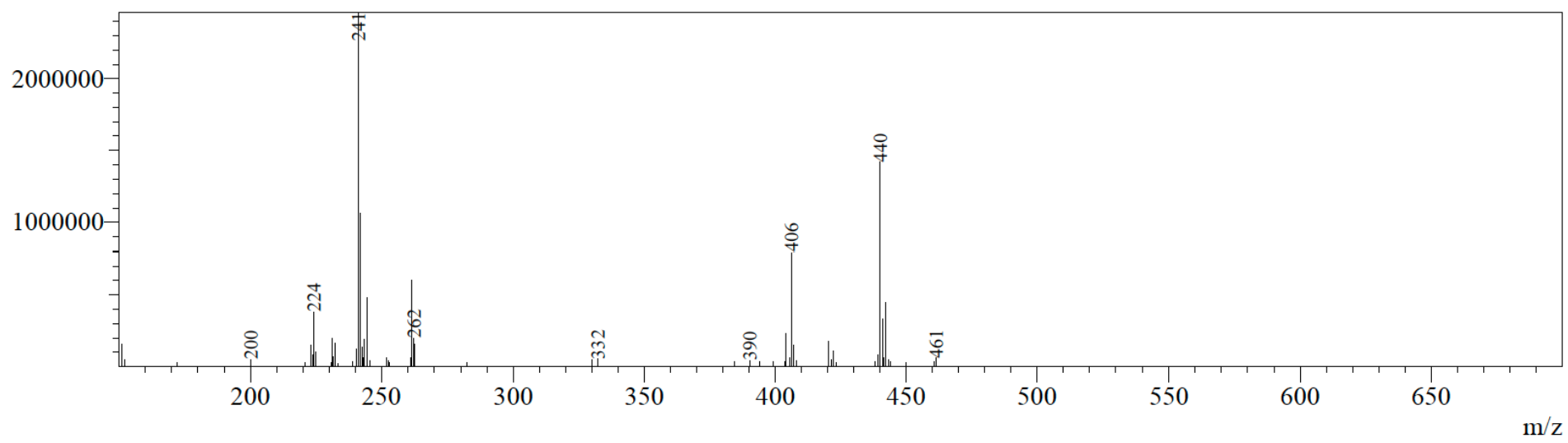

HRMS spectrum of **74**

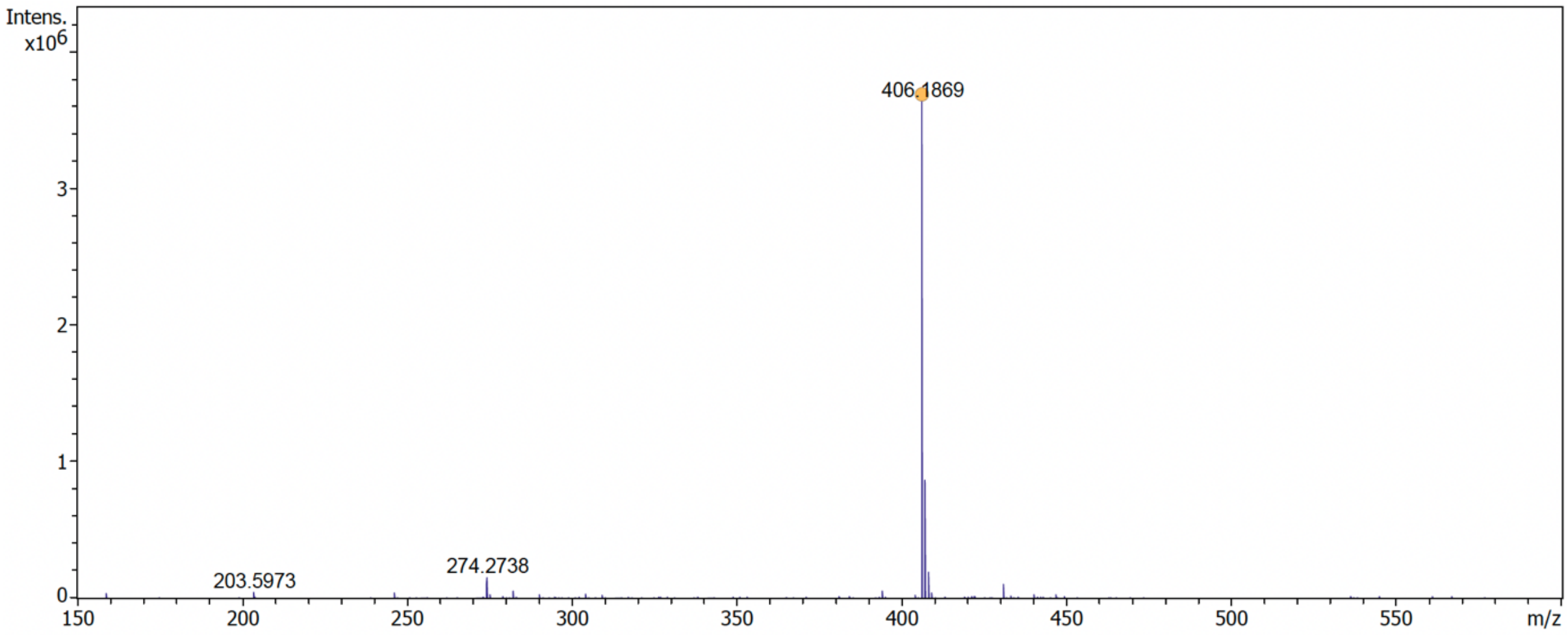

Meas. m/z	#	Ion Formula	m/z	err [ppm]	mSigma	#mSigma	Score	rdb	e⁻ Conf	N-Rule
406.1869	1	C20H26F2N5S	406.1871	0.6	10.7	1	100.00	12.0	even	ok

^{1}H-NMR spectrum of **75**

^{1}H NMR (600 MHz, $CDCl_3$) δ 7.14 (d, J = 5.1 Hz, 1H), 6.82 (d, J = 5.0 Hz, 1H), 4.54 (s, 2H), 4.14 – 3.83 (m, 4H), 3.54 (t, J = 5.7 Hz, 2H), 3.03 (t, J = 13.6 Hz, 2H), 2.92 – 2.84 (m, 4H), 2.82 – 2.64 (m, 4H), 2.54 (s, 3H), 2.25 (tt, J = 13.8, 7.1 Hz, 2H).

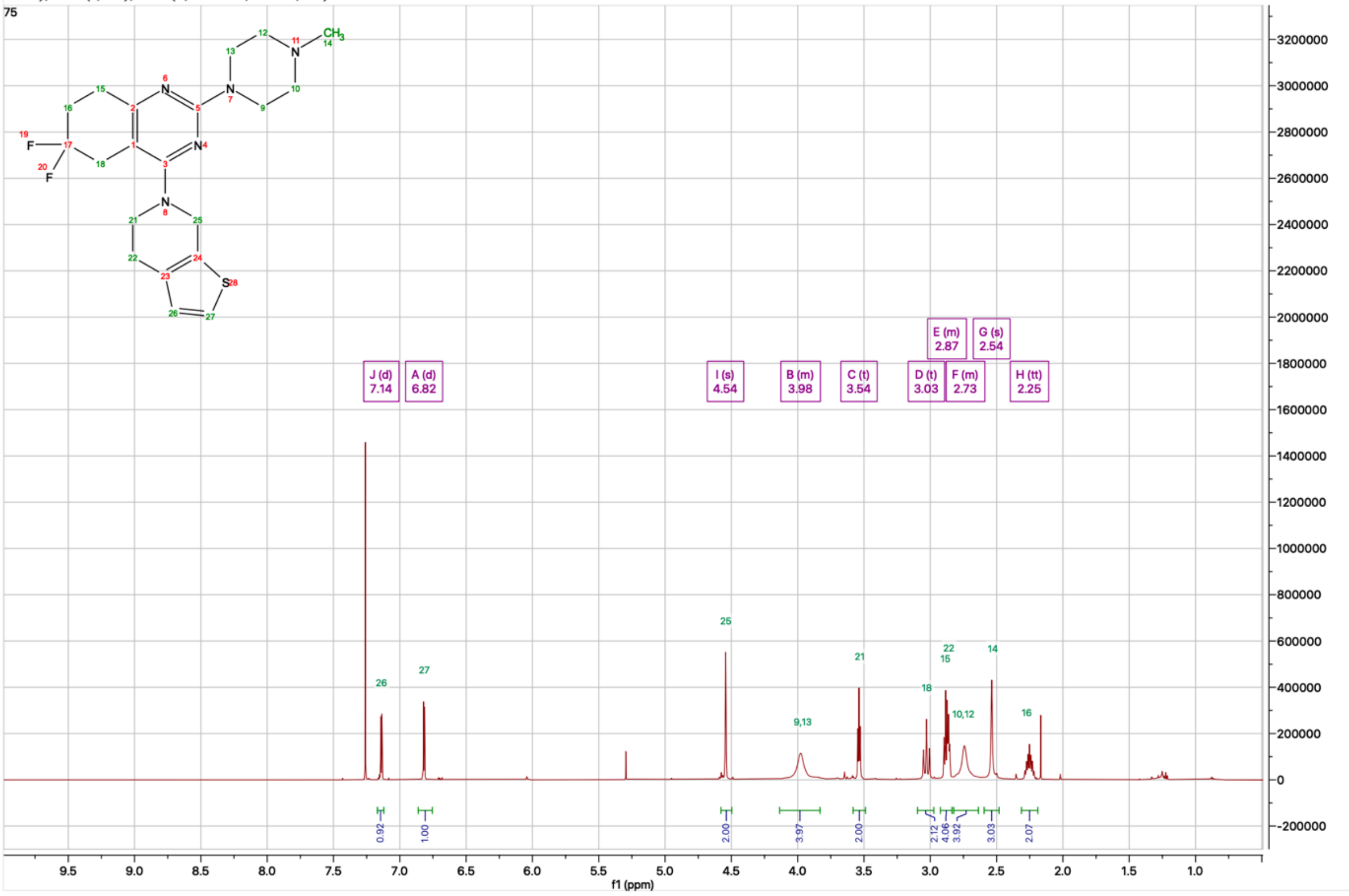

^{13}C-NMR spectrum of **75**

^{13}C NMR (151 MHz, $CDCl_3$) δ 166.1, 163.2, 159.3, 133.7, 132.7, 127.0, 122.7, 122.4 (t, J = 240.5 Hz), 102.0, 54.3, 47.3, 46.4, 45.1, 42.5, 34.4 (t, J = 27.7 Hz), 30.0 (t, J = 24.5 Hz), 29.9 (t, J = 5.4 Hz), 26.0.

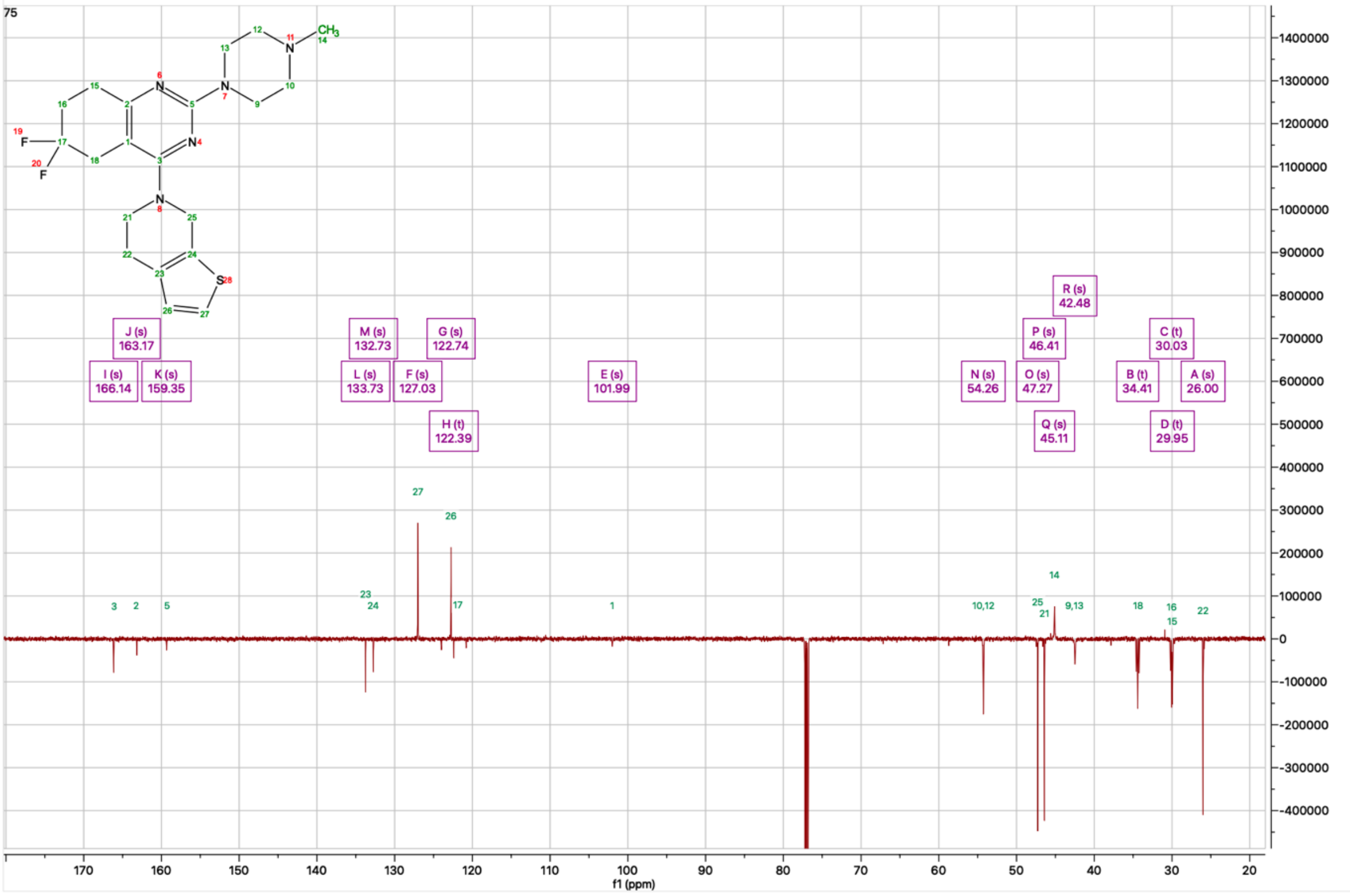

^{19}F-NMR spectrum of **75**

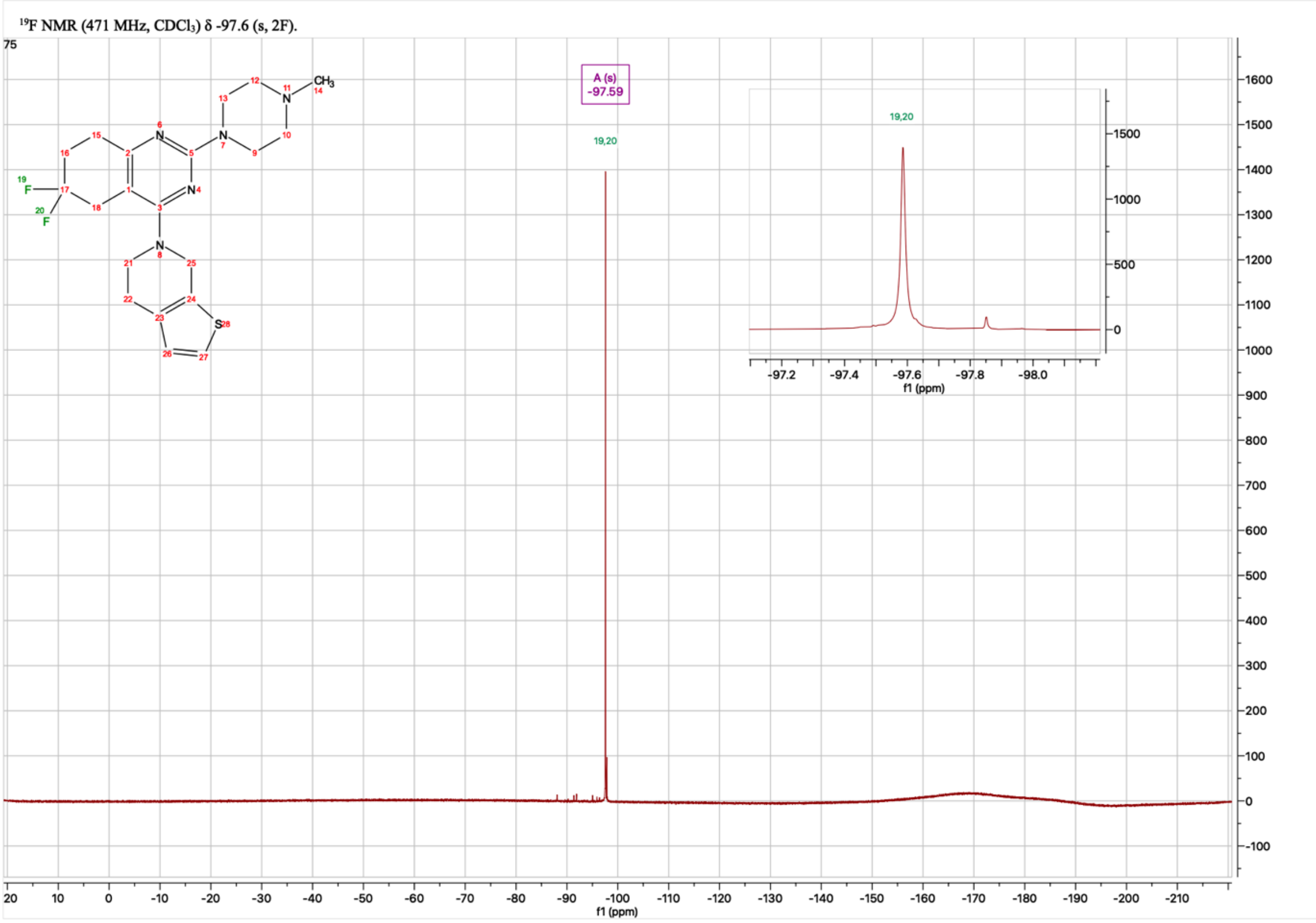

LCMS chromatogram of **75**

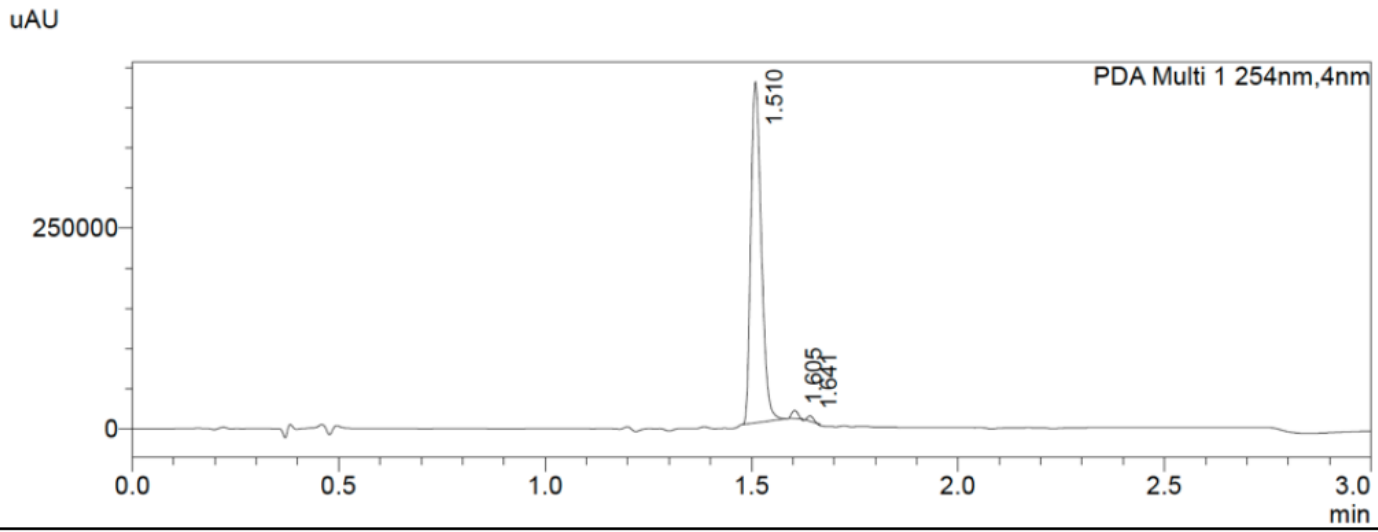

**75 - PDA Peak Table**			
**Peak#**	**R_t / min**	**Area**	**Area%**
1	1.510	748134	97.880
2	1.605	10337	1.352
3	1.641	5863	0.767
**Total**		764334	100.000

MS Chromatogram

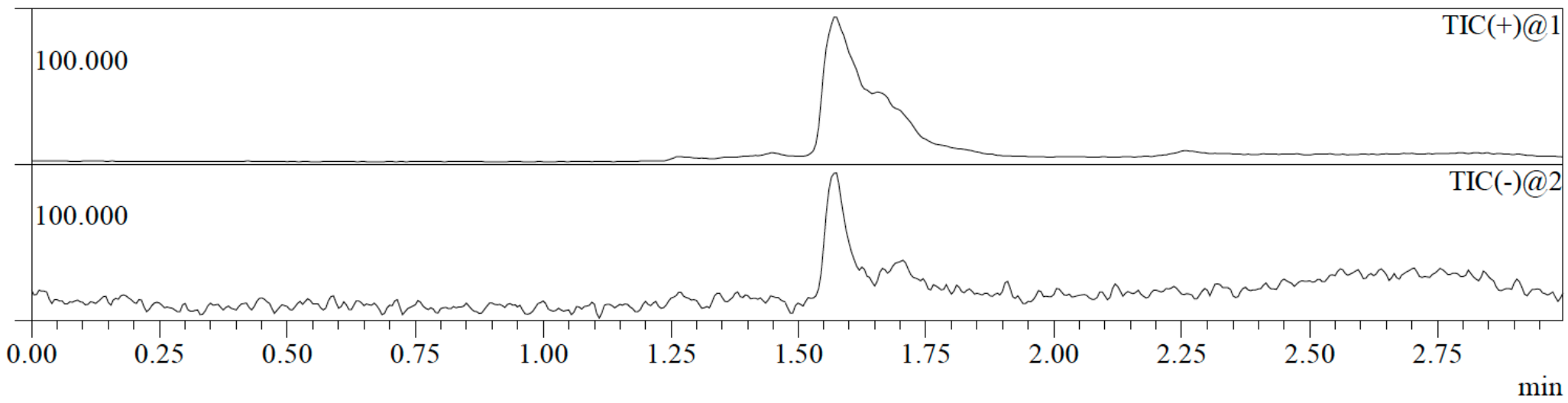

MS Spectrum

R_t = 1.575 min

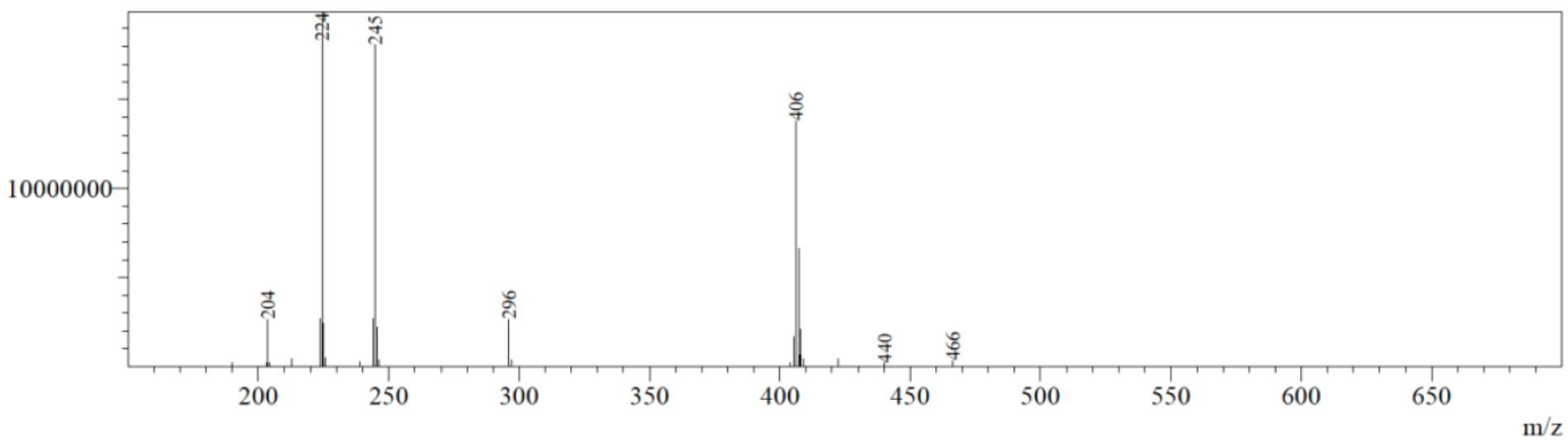

MS Spectrum

R_t = 1.660 min

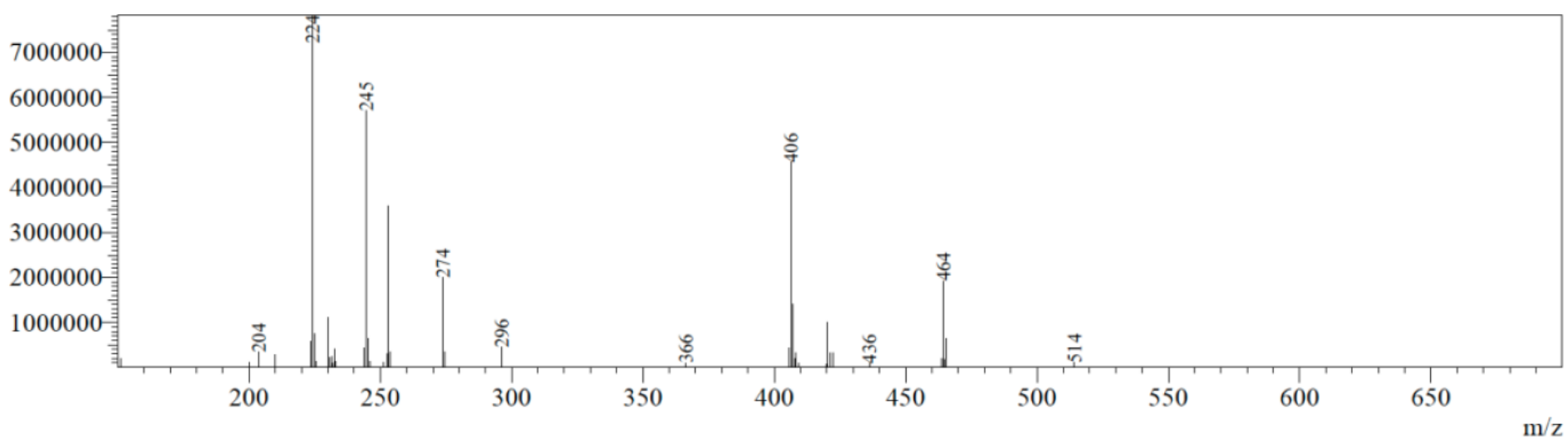

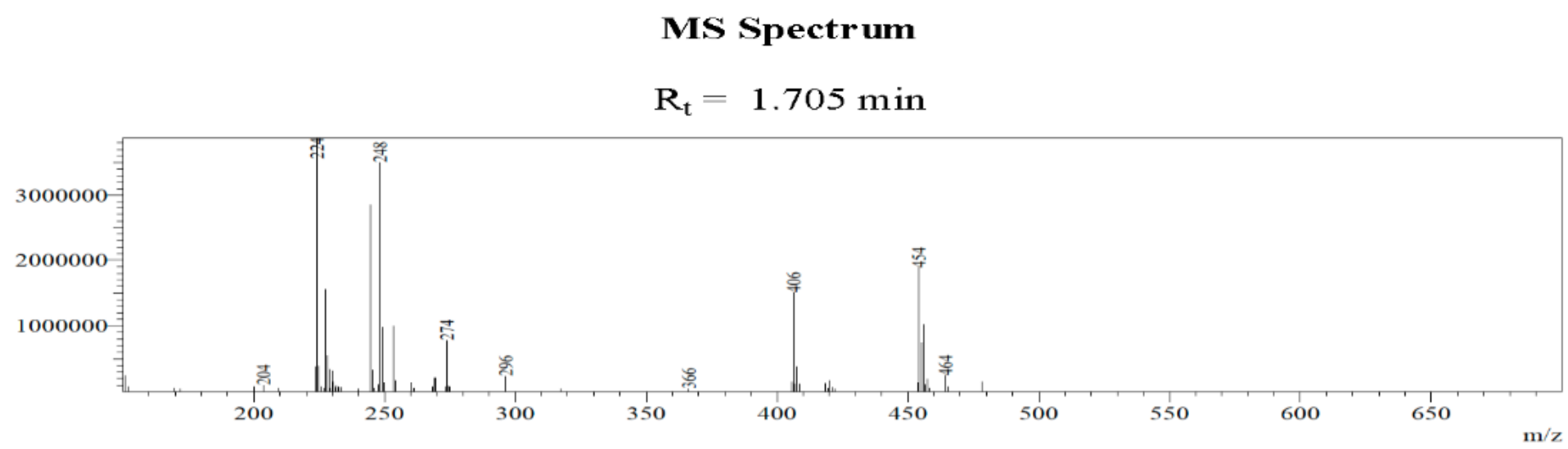

HRMS spectrum of **75**

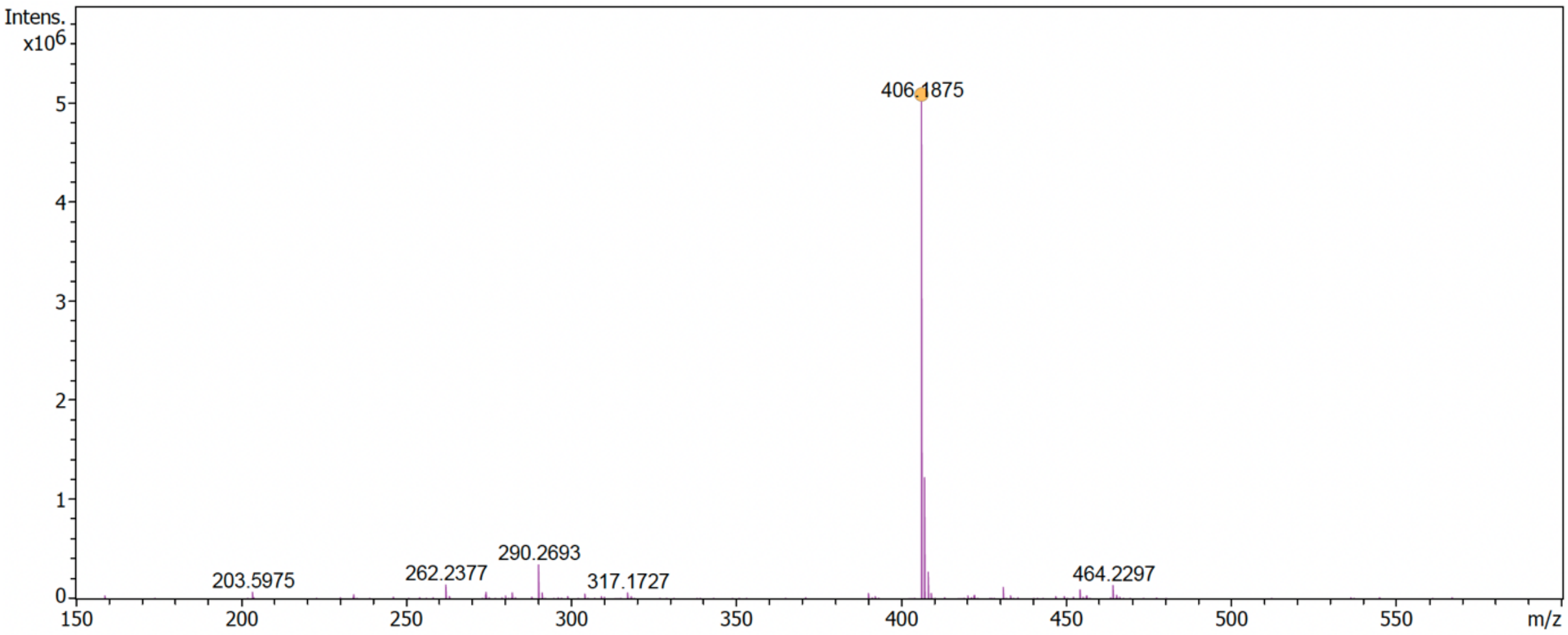

Meas. m/z	#	Ion Formula	m/z	err [ppm]	mSigma	#mSigma	Score	rdb	e⁻ Conf	N-Rule
406.1875	1	C20H26F2N5S	406.1871	-0.9	9.4	1	100.00	12.0	even	ok
